# Co-dependency for MET and FGFR1 in basal triple-negative breast cancers

**DOI:** 10.1038/s41523-021-00238-4

**Published:** 2021-03-26

**Authors:** Vanessa Y. C. Sung, Jennifer F. Knight, Radia M. Johnson, Yaakov E. Stern, Sadiq M. Saleh, Paul Savage, Anie Monast, Dongmei Zuo, Stéphanie Duhamel, Morag Park

**Affiliations:** 1 Rosalind and Morris Goodman Cancer Research Centre, McGill University, Montreal, QC Canada; 2 Department of Biochemistry, McGill University, Montreal, QC Canada; 3 Department of Bioinformatics and Computational Biology, Genentech Inc, South San Francisco, CA USA; 4 Department of Medicine, McGill University, Montreal, QC Canada; 5 Department of Oncology, McGill University, Montreal, QC Canada

**Keywords:** Breast cancer, Prognostic markers

## Abstract

Triple-negative breast cancer (TNBC) is a heterogeneous disease that lacks both effective patient stratification strategies and therapeutic targets. Whilst elevated levels of the MET receptor tyrosine kinase are associated with TNBCs and predict poor clinical outcome, the functional role of MET in TNBC is still poorly understood. In this study, we utilise an established Met-dependent transgenic mouse model of TNBC, human cell lines and patient-derived xenografts to investigate the role of MET in TNBC tumorigenesis. We find that in TNBCs with mesenchymal signatures, MET participates in a compensatory interplay with FGFR1 to regulate tumour-initiating cells (TICs). We demonstrate a requirement for the scaffold protein FRS2 downstream from both Met and FGFR1 and find that dual inhibition of MET and FGFR1 signalling results in TIC depletion, hindering tumour progression. Importantly, basal breast cancers that display elevated MET and FGFR1 signatures are associated with poor relapse-free survival. Our results support a role for MET and FGFR1 as potential co-targets for anti-TIC therapies in TNBC.

## Introduction

Triple-negative breast cancer (TNBC) is a heterogeneous subgroup of aggressive breast cancers, accounting for ~15% of all invasive breast cancers^1^. Clinically, patients with TNBC have fewer therapeutic options due to the lack of HER2 amplification and negative oestrogen and progesterone receptor (ER and PR, respectively) status. Patients with TNBC are at high risk of local and metastatic recurrence within the first 5 years post treatment and have poor overall survival^1,2^. The main challenge in treating TNBC is the paucity of patient stratification strategies capable of informing therapeutic decisions. Conventionally, TNBC is largely divided into the basal-like and claudin-low molecular subtypes of breast cancer^3,4^. While more recent efforts to further stratify patients have identified four to seven subtypes based on gene expression and mutational spectra, with the exception of a subtype enriched for the androgen receptor, the clinical relevance of each of these subtypes remains to be fully established^5–8^. Consequently, treatment for TNBC still typically involves combinations of anthracycline and taxane-based chemotherapies along with surgery and in some cases radiotherapy, with no clear consensus on new therapeutic regimens^1,2^.

A growing body of evidence suggests that disease relapse in breast cancer is attributable to the survival of a subpopulation of tumour cells with stem-like properties, termed tumour-initiating cells (TICs) or cancer stem cells^9–12^. TICs are capable of self-renewal and regenerate the heterogeneity of the original tumour following transplantation^13^, display resistance to conventional therapies^9,14–16^ and are postulated to seed local recurrence and distant metastases^17–20^. As a result, therapies that debulk tumours by indiscriminately eradicating highly proliferating cells, but do not target TICs, are now recognised as unlikely to cure patients^21,22^. Signalling pathways that promote TICs, as well as inhibitory compounds that target them, have therefore been the subject of intense research focus^23,24^. Highly tumourigenic and stem-like properties observed in TIC populations can be enhanced by the process of epithelial to mesenchymal transition (EMT)^25,26^, a cellular programme whereby epithelial cells decrease cell–cell junctions, lose apical–basal polarity, and display increased migratory and invasive capacities associated with mesenchymal cells. EMT is orchestrated by a restricted number of transcription factors namely those in the *SNAI*, *TWIST*, and *ZEB* families^27,28^. Indeed, breast TICs possess a mesenchymal gene expression signature supportive of mesenchymal and stem-like properties observed in breast cancer cells undergoing EMT such as elevated expression of *ALDH* and *CD44* and low expression of *CD24*^9,11,29^. The level of expression of these markers is associated with poor clinical outcome in TNBC patients^11^.

MET is a receptor tyrosine kinase (RTK) that, when bound by its ligand hepatocyte growth factor (HGF), coordinates a programme of invasive growth that broadly overlaps with the process of EMT, both in cancer and during the physiological process in development^30^. MET also regulates a variety of distinct biological processes characteristically exploited by tumours, including cell scattering, epithelial remodelling, cell proliferation and cell survival^31^. Elevated levels of MET protein are detected in 15–20% of all breast cancers and are associated with poor outcome across subtypes and within the TNBC subclass itself^32–35^. Despite these correlations, the functional role of MET in TNBC remains poorly understood. We have previously developed a murine model in which mammary gland expression of *Met* (MMTV-*Met*^*mt*^)^34^ and loss of the tumour suppressor gene *Trp53* (MMTV-*Met*^*mt*^*;Trp53fl/+;Cre*) synergise to promote tumours displaying a spindloid, mesenchymal pathology associated with the molecular and pathological features of claudin-low breast cancer^36^. Cross-species gene expression analysis revealed that spindloid tumours were characterised by mesenchymal properties and high expression of EMT and stem cell gene signatures, reminiscent of the basal B molecular subtype of breast cancer^4,36,37^. In this murine model of basal breast cancer, the endogenous *MET* locus becomes amplified and Met kinase activity is required to sustain both the EMT phenotype and proliferation of spindloid tumour cells^36^.

Fibroblast growth factor receptors (FGFRs) are a family of four RTKs (FGFR1-4) that are activated by fibroblast growth factors (FGFs) and regulate predominantly cell survival, proliferation and differentiation, and are implicated in many cancers^38^. Amplification of the genomic locus of *FGFR1* at chromosomal region 8p11-12 occurs in ∼10% of breast cancers^39^. While in ER + breast cancers, *FGFR1* amplification is associated with poor prognosis^39,40^ and resistance to endocrine therapy^39^, the role of FGFR1 in TNBCs remains poorly understood.

In this study, we assay TIC properties to directly investigate the role of Met in tumour initiation and identify FGFR1 signalling as a key convergent pathway with Met for the maintenance of TICs. We find that simultaneous inhibition of Met and FGFR1 activity abrogates properties of stemness and reduces TICs, hindering tumour progression. Importantly, we show that both human TNBC cell lines and TNBC patient-derived xenografts (PDXs) with co-expression of MET and FGFR1 are highly sensitive to dual-MET-FGFR1 inhibition in TIC assays. Finally, we find that human TNBCs with mesenchymal characteristics are significantly enriched for *HGF* and *FGFR1*, and that co-expression predicts poor prognosis among basal breast cancer patients. These studies identify MET and FGFR1 as co-regulators of TIC properties in basal breast cancer and provide a new understanding of treatment modalities for highly mesenchymal TNBC tumours.

## Results

### MMTV-*Met*^*mt*^;*Trp53fl*/+;*Cre* spindloid tumour cells are enriched in tumour-initiating cells

Spindloid tumour cells from the MMTV-*Met*^*mt*^;*Trp53fl*/+;*Cre* murine basal-like mammary tumour model are associated with increased mesenchymal status and tumourigenicity^36^. Unsupervised clustering of gene expression data from three independent MMTV-*Met*^*mt*^*;Trp53fl/+;Cre*-derived spindloid and non-spindloid tumours revealed that spindloid tumours displayed a gene expression profiling distinct from the non-spindloid tumours (Supplementary Fig. 1a). Analysis of Molecular Signatures DB (MSigDB) revealed that spindloid tumours were predominantly linked to stemness, EMT, and invasive gene signatures, while differentiation, *Cdh1* (E-Cadherin), and cell junction gene signatures were enhanced in non-spindloid tumours (Supplementary Fig. 1b, c). Accordingly, increased levels of key regulatory genes implicated in the promotion of EMT and stemness (*Cd44*, *Mybl2* and *Hmga2*) were elevated in the spindloid tumours, while higher levels of cell junction markers (*Cldn1, -7* and *-8)* and differentiation genes (*Gdpd2* and *Ogn*)^9,11,29^ are elevated in non-spindloid tumours (Supplementary Fig. 1d).

**Fig. 1:**
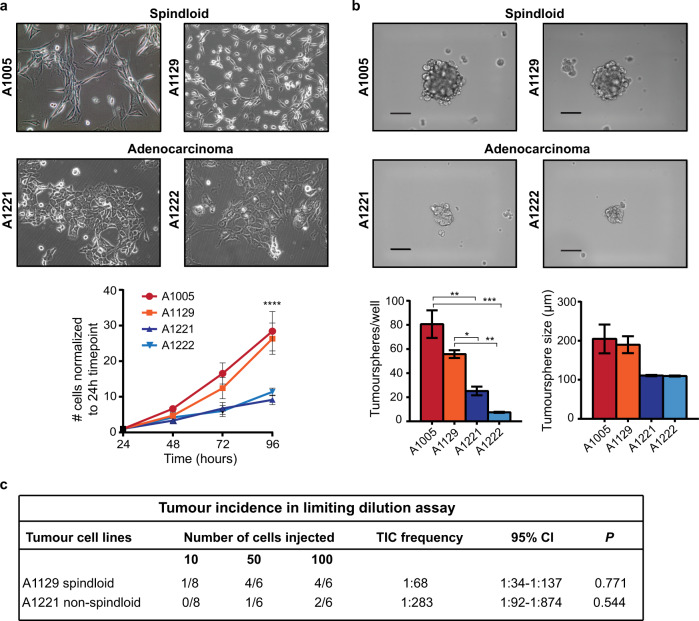
MMTV-*Met*^*mt*^*;Trp53fl*/+;*Cre* spindloid tumour-derived cells have higher sphere-forming efficiencies (SFE) than non-spindloid cells. **a** Representative image of MMTV-*Met*^*mt*^*;Trp53fl*/+;*Cre* spindloid cell lines A1005 and A1129 and adenocarcinoma (non-spindloid) cell lines A1221 and A1222 in adherent culture are shown (top). The proliferative rates of the spindloid (A1005, A1129) and non-spindloid (A1221, A1222) cell lines were assessed for 96 h by Trypan blue assay (bottom) (*n* = 3). **b** Representative images of tumourspheres formed from MMTV-*Met*^*mt*^*;Trp53fl*/+;*Cre* tumour cell lines (top). Tumourspheres were assessed on day 5 for quantity and size using AxioVision (Carl Zeiss) (bottom). Scale bar: 100 μm (*n* = 3). **c** Cells were injected into the mammary fat pads of female athymic mice at the indicated numbers. Tumour-initiating capacity was calculated using Extreme Limiting Dilution Analysis online software (http://bioinf.wehi.edu.au/software/elda/) 12.5 weeks post-inoculation (10 injected cells (*n* = 8); 50 injected cells (*n* = 6); 100 injected cells (*n* = 6)). Student’s *t* test; *P ≤ 0.05; **P ≤ 0.01; ***P ≤ 0.001; ****P ≤ 0.0001. Error bars indicate SEM.

To evaluate if this is reflective of a higher tumour-initiating cell population, we compared TIC populations from spindloid tumours with those from non-spindloid tumours from the same murine model utilising a range of established assays. Cell lines derived from spindloid tumours (A1005 and A1129) were nearly three times more proliferative than cell lines derived from non-spindloid tumours (A1221 and A1222) (Fig. 1a). To functionally test TIC capacity, we employed a tumoursphere assay commonly used to enrich for putative TICs based on their stem-like ability to survive and propagate in vitro as spheres in suspension^41^. We found that spindloid cell lines (A1005 and A1129) formed a higher number of and larger tumourspheres when compared to non-spindloid cell lines (A1221 and A1222), hence supporting the idea that spindloid cell lines possess higher proportions of TICs that form tumourspheres with greater proliferative capacity (Fig. 1b). Since limiting dilution assays performed in vivo remain the gold-standard functional readout for TIC frequency, we tested this by injecting spindloid (A1129) and non-spindloid (A1221) single cells at limiting dilutions (100, 50 and 10 cells) into the 4th mammary fat pads of female athymic mice. As predicted, the TIC frequency of the mesenchymal A1129 cells was four times higher than that of A1221 cells (1:68 and 1:283, respectively, *P* = 0.023) (Fig. 1c), providing further support that MMTV-*Met*^*mt*^*;Trp53fl/+;Cre* spindloid tumour cells are enriched for TIC.

### Dual inhibition of Met and FGFR targets TICs from spindloid tumours in vitro

**Fig. 2:**
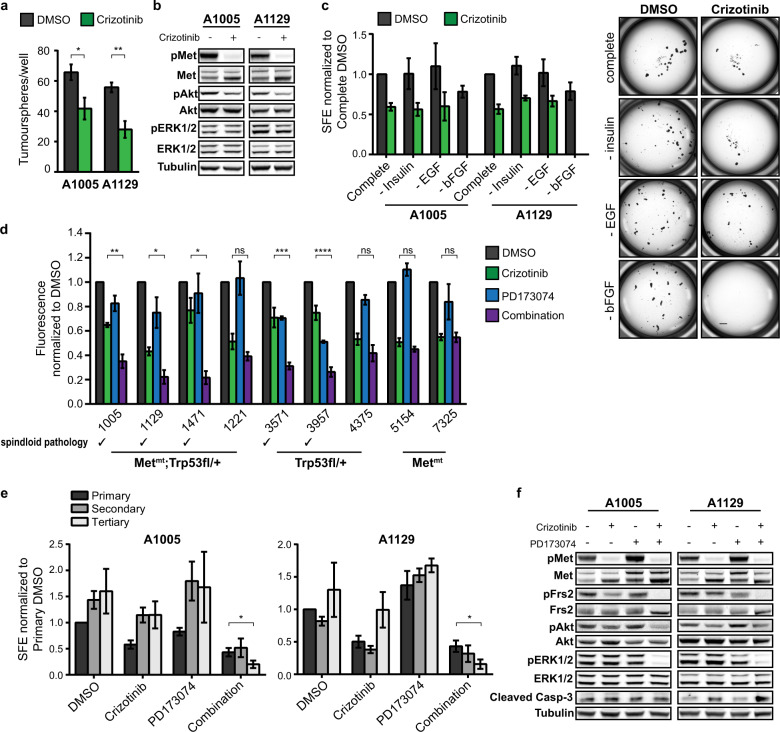
Dual inhibition of Met and FGFR signalling abrogates tumoursphere formation of spindloid tumour cells in vitro. **a** A1005 and A1129 tumourspheres were cultured in the presence or absence of Crizotinib (1 μM) and counted after 5 days (*n* = 3). **b** The activation of Met and its downstream signalling effectors in control and Crizotinib-treated tumourspheres were assessed by western blot analysis (*n* = 3). **c** A1005 and A1129 tumourspheres were cultured in media lacking insulin, EGF (20 ng/ml), or bFGF (20 ng/ml), and in the presence or absence of Crizotinib (left). Representative images of tumourspheres in growth factor-depleted media are shown (right) (*n* = 3). Scale bar: 500 μm. **d** A panel of MMTV-*Met*^*mt*^*;Trp53fl*/+;*Cre*, and MMTV-*Met*^*mt*^ cells were seeded in 96-well low-attachment plates, treated with 1 μM Crizotinib and/or 1 μM PD173074, and tumoursphere proliferation was detected by Cyquant proliferation assay (Invitrogen) (*n* = 3). **e** A1005 and A1129 tumourspheres were allowed to form over 48 h, and subsequently treated with Crizotinib and/or PD173074 for 72 h. Primary tumourspheres were enzymatically and mechanically dissociated into single cells and serially passaged to assess self-renewal capacity (*n* = 3). **f** Western blot analysis was used to detect cell death and signalling changes in tumourspheres treated with Crizotinib and/or PD173074 for 72 h (*n* = 3). Student’s *t* test; *P ≤ 0.05; **P ≤ 0.01; ***P ≤ 0.001; ****P ≤ 0.0001. Error bars indicate SEM.

The EMT phenotype and proliferative capacity of MMTV-*Met*^*mt*^*;Trp53fl/+;Cre* spindloid cells on both plastic and in soft agar are dependent on Met kinase activity^36^. To determine whether active Met signalling was also required to maintain TIC populations in these cells, tumourspheres derived from A1005 and A1129 cells were cultured in the presence of the small-molecule Met inhibitor Crizotinib for 5 days. Met inhibition significantly decreased but did not completely abrogate tumoursphere formation (Fig. 2a). When downstream signalling pathways were examined, we observed that following Met inhibition, Akt showed a decrease in phosphorylation, whereas ERK1/2 phosphorylation remained unaffected (Fig. 2b). The ability of RTKs, including Met, to engage in compensatory signalling is well-documented^42^, hence we hypothesised that loss of Met signalling may be compensated by growth factors present in the sphere culture medium, namely, epidermal growth factor (EGF), basic fibroblast growth factor (bFGF) and insulin^43^. To test this, tumoursphere assays were performed with sequential removal of individual growth factors. Strikingly, in the absence of bFGF, treatment with Crizotinib abrogated tumoursphere formation of both A1005 and A1129 cell lines (Fig. 2c). This difference supports that signalling through the FGFR pathway can compensate for loss of Met signalling in response to Crizotinib to sustain TICs derived from MMTV-*Met*^*mt*^*;Trp53fl/+;Cre* spindloid cells.

To determine if the signalling interaction between Met and FGFR was specific to MMTV-*Met*^*mt*^*;Trp53fl/+;Cre* spindloid tumours, or was ubiquitous among different breast cancer models, we tested a panel of cell lines derived from MMTV-*Met*^*mt*^*;Trp53fl/+;Cre*, *Trp53fl/+;Cre* and MMTV-*Met*^*mt*^ tumours. Tumourspheres were cultured in the presence of DMSO, Crizotinib alone, small-molecule FGFR inhibitor, PD173074, alone, or both inhibitors in combination. Tumoursphere proliferation, measured by Cyquant proliferation assays after 7 days in culture, revealed that all spindloid tumour cell lines, including those derived from *Trp53fl/+;Cre* tumours (which spontaneously amplified the endogenous murine *Met* locus^36^), showed a significant reduction in sphere proliferation when treated with both inhibitors when compared to either inhibitor alone (Fig. 2d). In contrast, non-spindloid tumour cells lines that retain expression of the MMTV-*Met*^*mt*^ transgene responded only to a Met inhibitor and are dependent on this oncogenic Met.

A key functional characteristic of TICs is the ability to self-renew, which can be assayed in vitro by assessing the capacity of tumourspheres to maintain sphere-forming efficiency (SFE) over multiple rounds of passage. Since spindloid tumourspheres fail to form in the presence of Met and FGFR inhibitors in combination, A1005 and A1129 tumourspheres were allowed to establish over 2 days before inhibitors were added for a further 3 days. On day 5, tumoursphere numbers in each condition were determined, tumourspheres were enzymatically and mechanically dissociated into single cells, re-plated, and the treatment with inhibitors was repeated. Strikingly, Met and FGFR inhibitor combination-treated tumourspheres exhibited a decrease in SFE by the tertiary passage that was not observed in any other conditions (Fig. 2e). Under these conditions, Met and FGFR co-inhibition resulted in a loss of ERK1/2 phosphorylation in both cell lines tested, as well as elevated levels of the apoptosis marker, cleaved Caspase-3, in A1129 tumourspheres (Fig. 2f). Interestingly, compensatory signalling between Met and FGFR correlated with the tyrosine phosphorylation of Frs2, a large scaffold protein known to couple FGFR1 with downstream signalling pathways, notably the ERK1/2 MAPK through recruitment of the adaptor protein Grb2^44^, but is poorly described downstream of Met. Under TIC conditions, Met and FGFR can both independently promote tyrosine phosphorylation of Frs2, enabling its function as a scaffold protein integrating each RTK with downstream signalling pathways. Notably, tyrosine phosphorylation of Frs2 is lost only under dual inhibition of Met and FGFR, resulting in loss of ERK1/2 phosphorylation. These findings support a network in which Met and FGFR signalling cooperate to sustain proliferation, self-renewal, and survival of TICs derived from spindloid, basal-like breast tumours through maintenance of an activated ERK1/2 pathway.

### FGFR1 is preferentially expressed in spindloid tumour cells and maintains tumoursphere formation when Met is inhibited

**Fig. 3:**
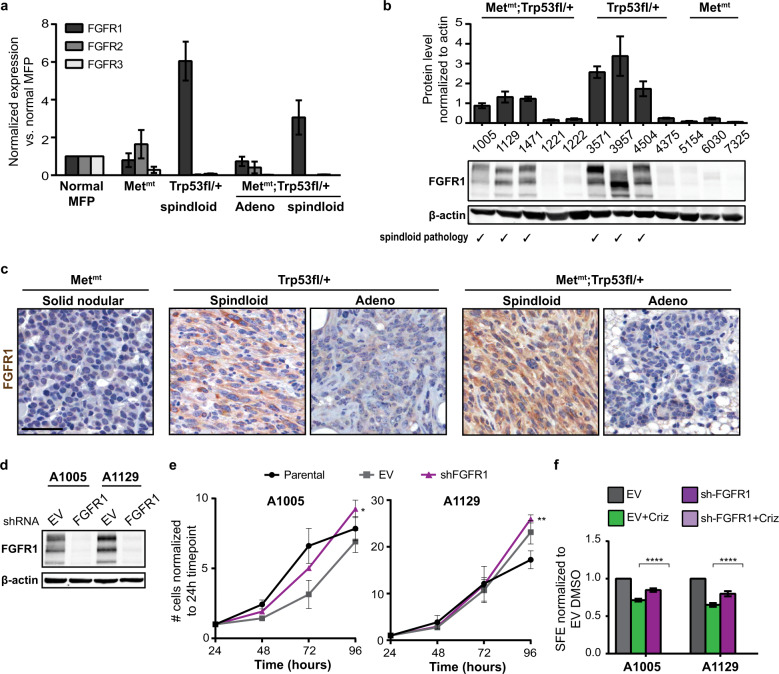
FGFR1 is preferentially expressed in spindloid tumours and compensates for loss of Met signalling to sustain tumourspheres. **a** RNA extracted from MMTV-*Met*^*mt*^, *Trp53fl*/+;*Cre* and MMTV-*Met*^*mt*^*;Trp53fl*/+;*Cre* tumour cells was used to assess relative expression levels of FGFR1, 2 and 3 using quantitative RT-PCR. **b** Western blot analysis to validate FGFR1 expression at the protein level. The pathology of each tumour-derived cell line tested (*n* = 3 for MMTV-*Met*^*mt*^, *n* = 4 for *Trp53fl/*+;*Cre* and *n* = 5 for MMTV-*Met*^*mt*^*;Trp53fl*/+;*Cre* tumours) is indicated. **c** Paraffin-embedded sections of corresponding tumours were analysed by immunohistochemistry for FGFR1 expression (*n* = 3). Representative images are shown. Scale bar: 50 μm. **d** A1005 and A1129 cells were transfected with empty vector or shRNA against FGFR1, and efficient knockdown of FGFR1 was verified by western blot analysis (*n* = 3). **e** The effect of FGFR1 knockdown on proliferation in adherent condition was assessed for 96 h by Trypan blue assay (*n* = 3). **f** The effect of FGFR1 knockdown on tumoursphere formation in the presence or absence of 1 μM Crizotinib was assessed after 5 days of culture. Student’s *t* test; *P ≤ 0.05; **P ≤ 0.01. Error bars indicate SEM.

We found *FGFR1* transcript to be selectively elevated in cells from spindloid tumours when compared to all other tumour types and normal mammary gland, while *FGFR2* and *FGFR3* were not modulated (Fig. 3a) and *FGRF4* was undetectable (not shown). The increase of FGFR1 expression in spindloid tumours was confirmed at the protein level (Fig. 3b). Consistent with our findings in tumour-derived cell lines, MMTV-*Met*^*mt*^*;Trp53fl/+;Cre* and *Trp53fl/+;Cre* mammary tumours with spindloid pathology showed stronger immunohistochemical staining for FGFR1 when compared to tumours with non-spindloid pathologies (Fig. 3c). To directly test the requirement for FGFR1 in spindloid tumoursphere formation, FGFR1 expression was silenced using shRNA in spindloid tumour-derived mesenchymal A1005 and A1129 cell lines (Fig. 3d). Whereas loss of FGFR1 did not inhibit 2D proliferation following Met inhibition by Crizotinib (Fig. 3e), tumoursphere formation was abrogated in the presence of Crizotinib (Fig. 3f) demonstrating that loss of FGFR1 sensitised tumourspheres from mesenchymal cell lines to Met inhibition. Likewise, shRNA mediated knockdown of Frs2 failed to inhibit 2D proliferation but abrogated tumoursphere formation of both A1005 and A1129 cell lines in the presence of Crizotinib (Supplementary Fig. 2a–c). These data demonstrate that the Frs2 scaffold protein is required downstream of FGFR1 for maintenance of TIC capacity of spindloid MMTV-*Met*^*mt*^*;Trp53fl/+;Cre* in the absence of Met activation.

### Co-inhibition of Met and FGFR signalling in TICs impairs EMT, stemness and proliferation, and induces a programme of cell differentiation

**Fig. 4:**
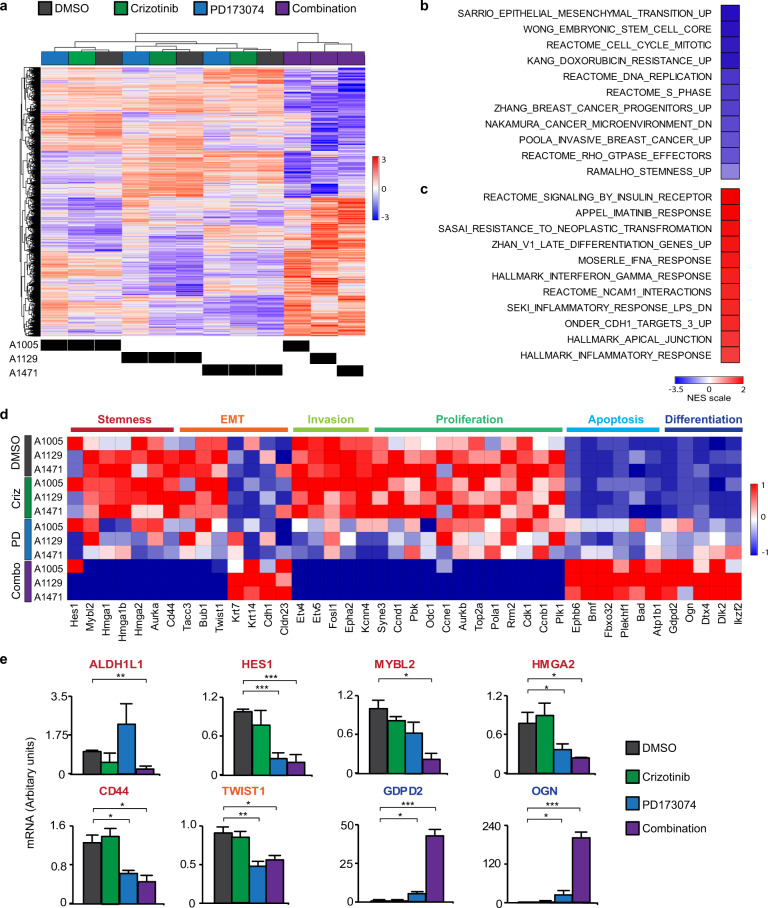
Co-inhibition of Met and FGFR1 signalling in TICs abrogates EMT, stemness and proliferation, and induces cell differentiation. **a** Tumourspheres generated from three independent MMTV-*Met*^*mt*^;*Trp53fl*/+;*Cre* spindloid tumour cell lines were treated for 24 h with DMSO, Crizotinib, PD173074, or a combination of both agents. RNA was extracted from treated tumourspheres and subjected to RNA-Sequencing. Unsupervised hierarchical clustering of all genes that were differentially expressed in each pairwise comparisons tested with adjusted *P* value ≤0.05 (BH method). **b**, **c** Heatmap showing changes in the normalised enrichment score (NES) for the most enriched pathways in tumourspheres treated for 24 h with DMSO, Crizotinib, PD173074 and a combination of both agents (1 μM for both inhibitors) (FDR *P* ≤ 0.05). The top-up and downregulated gene signatures of Hallmark, genetic and chemical perturbations and Reactome pathways. **d** Heatmap representation of the relative expression of known phenotypic markers for each indicated cellular process. **e** Quantitative RT-PCR for the indicated genes. *P ≤ 0.05; **P ≤ 0.01; ***P ≤ 0.001. Error bars indicate SEM.

To understand the events that occur in TICs upon inhibition of Met and FGFR1, we performed gene expression profiling by RNA-Sequencing of RNA prepared from tumourspheres from three independent MMTV-*Met*^*mt*^*;Trp53fl/+;Cre*-derived spindloid tumour cell lines (A1005, A1129 and A1471) following treatment with Met or FGFR inhibitor alone (Crizotinib or PD173074, respectively), or in combination for 24 h. All three cell lines treated with inhibitor combinations displayed a gene expression signature distinct from that of cells treated with either inhibitor alone (Fig. 4a). By contrast, and consistent with the moderate effect of Met inhibition alone on tumoursphere formation (Fig. 2a, d), there were no statistically significant differences in gene expression between DMSO and Crizotinib alone-treated cells (Fig. 4a). Notably, the gene expression signature of tumourspheres treated with FGFR inhibitor, PD173074, alone exhibited a partial overlap with expression profiles observed in tumourspheres treated with a combination of Met and FGFR inhibitors; however, no significantly modulated gene signatures were observed.

To gain further insight into these differences, we performed gene set enrichment analysis (GSEA) on tumourspheres subjected to dual-MET-FGFR inhibition and found negative enrichment indicating loss of gene signatures associated with EMT, stemness, breast cancer progenitors, and Met signalling when compared to DMSO-treated control tumourspheres (Fig. 4b, d). Reduced expression of key regulatory genes implicated in the promotion of EMT (*Twist1*) and stemness (*Aldh1h1, Hes1, Mybl2*, *Hmga2* and *Cd44*) were confirmed independently by qPCR (Fig. 4e). Interestingly, the HES1 pathway is essential for the maintenance of TIC in breast cancer cells and has been associated with metastasis and multidrug resistance^45^. Cancer microenvironment, invasiveness, and Rho GTPase gene signatures were also negatively enriched indicating loss in the combination-treated tumourspheres, in accordance with the reversion of mesenchymal and invasive properties in the treated cells. Reciprocally, GSEA showed enhancement for gene signatures associated with interactions with the adhesion mediator NCAM1, the cell junction protein CDH1, and Integrin surface adhesion, which provides mechanical strength and influences the differentiation state of cells in contact with the extracellular matrix^46^ (Fig. 4c). An increase in cell differentiation gene signatures was also observed in combination-treated tumourspheres (Fig. 4c, d), with significantly elevated expression of the differentiation genes *Gdpd2* and *Ogn*, confirmed by qPCR (Fig. 4e). In accordance with the increased levels of cleaved Caspase-3 observed in combination-treated tumourspheres (Fig. 2f), a significant increase of the cell death regulators *Bmf* and *Bad* genes was observed (Fig. 4d). Notably, qPCR analyses revealed that the expression of the key EMT/Stemness regulators, *Twist1*, *Cd44, Hes1* and *Hmga2* were significantly modulated in PD173074-treated tumourspheres (Fig. 4e). Overall, the observed changes in gene expression are consistent with a reversion of EMT and stem-like features and support the induction of a differentiation programme in *Met*^*mt*^*;Trp53fl/+;Cre* spindloid cells upon loss of Met and FGFR signalling, resulting in a reduced capacity to survive and propagate under TIC conditions.

### Dual inhibition of Met and FGFR signalling reduces spindloid tumour initiation and impairs progression of established spindloid tumours in vivo

**Fig. 5:**
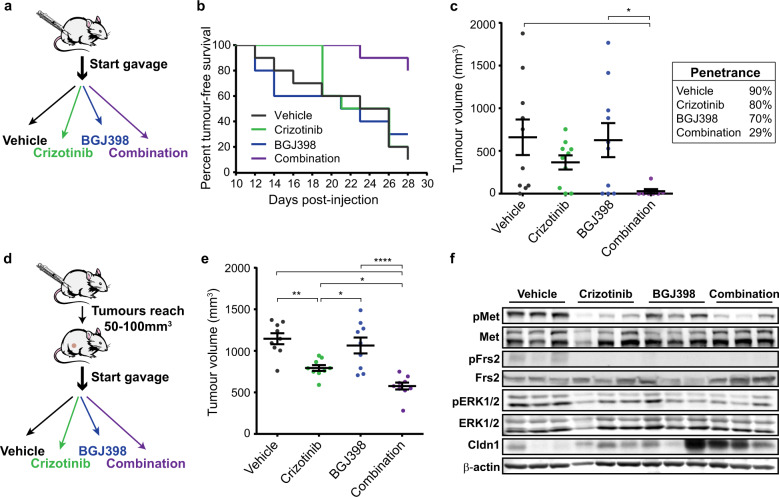
Co-inhibition of Met and FGFR1 signalling in vivo reduces tumour-initiating potential of MMTV-*Met*^*mt*^;*Trp53fl/+*;*Cre* spindloid cells and delays progression of established tumours. **a** Female athymic mice were injected with A1129 cells in the 4th mammary fad pad and treated daily with vehicle control, Crizotinib alone (50 mg/kg), BGJ398 alone (30 mg/kg), or the two agents in combination by oral gavage. **b** Kaplan–Meier curves of the percentage of tumour-free A1129-injected mice in the indicated treatment groups. Mice were sacrificed after 28 days and their tumours collected. **c** Tumour volumes on day of sacrifice (left) and percent penetrance in each treatment group (right) (vehicle (*n* = 10), Crizotinib (*n* = 10), BGJ398 (*n* = 10), and combination (*n* = 7)). **d** Female athymic mice were injected with A1129 cells in the 4th mammary fat pad and tumours were allowed to reach 50–100 mm^3^ in volume before randomisation into treatment groups. **e** Tumour volumes after 11 days of drug treatment (*n* = 9). **f** Tumour lysates were prepared from three mice per treatment group, and western blot analysis was performed for the indicated antibodies. Student’s *t* test; *P ≤ 0.05; **P ≤ 0.01; ****P ≤ 0.0001. Error bars indicate SEM.

To investigate whether co-targeting Met and FGFR in vivo could suppress tumour-initiation, female athymic mice were injected with A1129 tumour cells and randomised into four groups, receiving either vehicle control, Crizotinib, the orally available FGFR inhibitor BGJ398, or both agents in combination by daily gavage (Fig. 5a). Mice receiving combination therapy showed prolonged event-free survival when compared to all other treatment arms (Fig. 5b). Treatment with Crizotinib alone decreased overall tumour volume, though not reaching statistical significance (Fig. 5c). Strikingly, whereas BGJ398 alone did not affect tumour burden, combination therapy with Crizotinib strongly reduced tumour penetrance. These results are consistent with in vitro data and support that tumour initiation of spindloid tumour cells is combinatorially dependent on Met and FGFR signalling in vivo.

To evaluate the potential of Met and FGFR co-inhibition as a therapeutic strategy, female athymic mice were orthotopically injected with spindloid A1129 tumour cells and randomised into 4 treatment groups and subjected to treatment when tumours reached 50–100 mm^3^ (Fig. 5d). Whereas combination treatment failed to induce regression of established tumours, it did impair progression more effectively than either treatment arm alone (Fig. 5e). Western blot analysis confirmed that Met and FGFR co-inhibition resulted in the loss of Met, Frs2 and ERK1/2 phosphorylation, whereas Crizotinib alone exerts a limited effect on ERK1/2 phosphorylation. Notably, following Crizotinib treatment the expression of the tight junction protein Claudin-1 was elevated, supporting our previous observations that activation of Met signalling decreases Claudin-1 levels and induces cell–cell junction disassembly^36^ (Fig. 5f). Taken together, our findings support that Met and FGFR signalling cooperate to support TIC capacity in murine basal-like mammary tumours with spindloid pathology, and that co-inhibition of both Met and FGFR receptors suppresses tumour initiation and impairs progression in vivo.

### Dual inhibition of MET and FGFR signalling targets TICs in basal B breast cancer cell lines

**Fig. 6:**
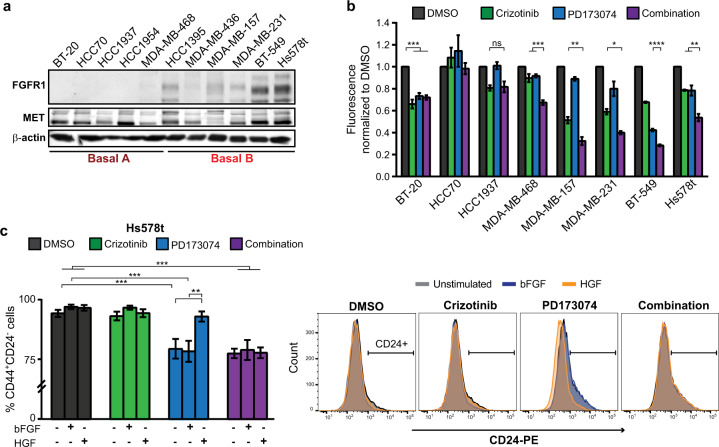
Co-inhibition of MET and FGFR signalling targets TICs in basal B breast cancer cell lines. **a** Western blot analysis was performed to validate MET and FGFR1 protein levels across a panel of TNBC cell lines (*n* = 3). **b** The indicated TNBC cell lines, comprised of a mix of basal A and basal B cell lines, were cultured as tumourspheres in the presence of HGF (50 ng/ml) and bFGF (20 ng/ml) and treated with 1 μM Crizotinib and/or 1 μM PD173074. Tumoursphere proliferation was detected by Cyquant proliferation assay (*n* = 3). **c** Hs578t cells were treated with Crizotinib and/or PD173074 for 2 h, followed by 30 min stimulation with HGF or bFGF. The proportion of CD44^+^CD24^-^ cells was determined by flow cytometry 72 h later (*n* = 3). Student’s *t* test; *P ≤ 0.05; **P ≤ 0.01; ***P ≤ 0.001; ****P ≤ 0.0001. Error bars indicate SEM.

We next sought to establish if TICs derived from human breast cancers were specifically dependent on FGFR1 and MET signalling. FGFR signalling is enriched in human-derived TNBC cell lines, several of which display autocrine bFGF signalling^47^. Among TNBC-derived cell lines used to interrogate human breast cancer biology, “Basal B” breast cancer cell lines are the most representative of claudin-low breast cancer^4,48,49^. Utilising gene expression data from the publicly available database, Cancer Cell Line Encyclopedia (CCLE), we found that basal B cell lines exhibited the highest expression of FGFR1 when compared to luminal, HER2, and basal A cell lines (corresponding to ER + , HER2 amplified and basal-like breast cancers, respectively). In contrast, FGFR2 and FGFR3 were not significantly correlated with any subgroup of breast cancer cell lines, whereas FGFR4 expression was elevated in HER2 and luminal cell lines (Supplementary Fig. 3a–d). Western blot analysis confirmed that FGFR1 expression is preferentially elevated in basal B cell lines (Fig. 6a).

To establish whether dual inhibition of MET and FGFR signalling results in reduced tumourigenicity, a panel of TNBC cell lines (four basal A and four basal B) were cultured as tumourspheres in the presence of DMSO, Crizotinib, PD173074, or both inhibitors in combination. In addition to bFGF, a regular component of tumoursphere medium, tumourspheres were also cultured in the presence of HGF to ensure MET activation. Measurement of tumoursphere proliferation revealed that tumourspheres from all four basal B cell lines consistently exhibited a greater decrease in proliferation under conditions of MET and FGFR co-inhibition when compared to all other conditions, while in contrast, basal A tumourspheres varied in their sensitivity (Fig. 6b).

In human breast cancer, TICs are often enriched within a population of cells characterised by the cell surface marker profile CD44^+^CD24^−/low^. Reflecting their strong overlap with mesenchymal/stem gene expression signatures, basal B cell lines typically have high percentages of CD44^+^CD24^−/low^ cells^23^. To investigate whether the CD44^+^CD24^−/low^ phenotype is regulated by MET or FGFR signalling, basal B cell lines Hs578t and BT-549 were cultured in the presence of HGF and/or bFGF and treated with Crizotinib and/or PD173074 for 3 days. In both cell lines, FGFR inhibition led to TIC depletion, which was rescued upon activation of MET by HGF. However, MET inhibition alone did not affect the CD44^+^CD24^−/low^ population (Fig. 6c and Supplementary Fig. S4a, b). When downstream signalling changes that occur in basal B tumourspheres upon MET and FGFR inhibition were interrogated, co-inhibition of MET and FGFR signalling abrogated tyrosine phosphorylation of FRS2, resulting in loss of ERK1/2 activation (Supplementary Fig. 4c) consistent with our observations in murine claudin-low-like breast cancer. Taken together, these results support that MET and FGFR signalling are key pathways involved in the regulation of human basal B-derived TICs.

### MET and FGFR1 are co-expressed in patient-derived basal B-like xenografts and are required for TIC capacity

**Fig. 7:**
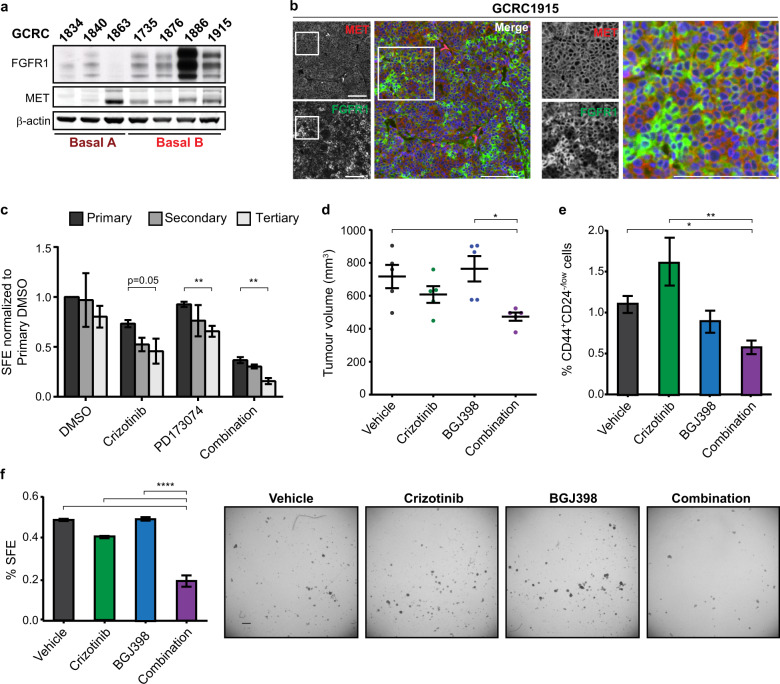
Basal B TNBC PDX tumours that co-express MET and FGFR1 are susceptible to TIC depletion via dual inhibition of MET and FGFR signalling. **a** Cell lysates were generated from mammary PDX tumour fragments and used to determine MET and FGFR1 expression by western blot (*n* = 3). **b** Immunofluorescence was performed on GCRC1915 PDX tumour sections to validate co-expression of MET and FGFR1. Scale bars: 100 μm. **c** GCRC1915 PDX was dissociated into single cells and cultured as tumourspheres in the presence of Crizotinib and/or PD173074 (1 μM for both inhibitors). Tumourspheres were subsequently serially passaged to evaluate self-renewal capacity (*n* = 3). **d** Female NSG-hHGFki mice were orthotopically injected with GCRC1915 cells, and tumours were permitted to progress to 50–100 mm^3^ in volume. Tumour-bearing mice were treated with vehicle control, Crizotinib (50 mg/kg), BGJ398 (30 mg/kg) or both agents in combination (*n* = 5). **e**, **f** Tumour volumes after 17 days of treatment are shown. Treated tumours were dissociated into single cells and evaluated for CD44^+^CD24^−/low^ populations by flow cytometry as well as cultured as tumourspheres and counted after 7 days of culture (*n* = 4). Scale bar: 200 μm. Student’s *t* test; *P ≤ 0.05; **P ≤ 0.01; ****P ≤ 0.0001. Error bars indicate SEM.

Patient-derived xenografts (PDXs) are primary patient tumour fragments that are serially transplanted in immunocompromised mice that are considered to recapitulate properties of the original patient tumours^50,51^. While some clonal divergence has been detected^52^, PDXs are robust tools for studying human cancer. Interrogating a panel of PDXs established from TNBC patients^53^, we found that the basal B-like PDXs, which are associated with increased stemness and EMT markers including *CD44*, *ALDH1A1*, *TWIST1/2* and *ZEB1/2*, and reduced expression of claudins^4,48,49^ (Supplementary Fig. 5a), co-expressed MET and FGFR1 proteins (Fig. 7a). Interestingly, *HGF* and *FGFR1* gene expression were also significantly increased in the basal B PDXs, as well as downstream effectors of the MET, FGFR1-FRS2 and ERK1/2 signalling pathways (Supplementary Fig. 5b). Unbiased GSEA analysis further confirmed that the basal B PDXs, expressing a higher level of FGFR1, are positively associated with stem cells and negative cell differentiation genes signatures and are correlated with increased expression of the stem cell markers *ALDH1A1* and *CD44* (Supplementary Fig. 5c, d). Immunofluorescence of FFPE sections derived from PDX tumours (GCRC1886 and GCRC1915) revealed that MET and FGFR1 are not mutually exclusive and that both RTKs are expressed in the same tumour cells (Fig. 7a and Supplementary Fig. S6a). The PDX GCRC1863, with low expression of FGFR1 and absence of Met and FGFR1 co-localisation in the tumour, was included to validate staining specificity (Supplementary Fig. 6a).

To assess the role of MET and FGFR signalling in putative TICs derived from PDXs, the GCRC1915 PDX tumour was collected, digested into single cells and cultured as tumourspheres in media containing DMSO, Crizotinib, PD173074, or both inhibitors in combination. A reduction in SFE was observed in both Crizotinib and combination-treated tumourspheres, with the latter exhibiting a larger decrease (Fig. 7c). Serial passaging revealed that MET-FGFR co-inhibition had the most deleterious effect on SFE.

To investigate how the GCRC1915 PDX would respond to MET and FGFR inhibitors in vivo, single cells were transplanted into mice carrying insertion of the human *HGF* gene in the murine locus (NSG-hHGFki), ensuring that both FGFR and MET signalling pathways can be activated. Combination treatment with both Crizotinib and BGJ398 by oral gavage resulted in a moderate but significant reduction in tumour burden compared to all other treatment arms (Fig. 7d). Treated GCRC1915 PDX tumours were collected at the endpoint and digested into single cells for flow cytometry analysis of TIC markers. We observed a 1.9-fold depletion in the proportion of the CD44^+^CD24^−/low^ TIC population in combination-treated tumours, but not in those treated with either inhibitor alone (Fig. 7e and Supplementary Fig. S6b). Consistent with TIC depletion, when single cells from treated tumours were cultured as tumourspheres, cells from combination-treated tumours resulted in a marked decrease in SFE compared to all other groups (Fig. 7f). Together, these in vitro and in vivo findings using PDX models provide strong support that MET and FGFR signalling can be combinatorially targeted to deplete TICs in human TNBC.

### Patient tumours enriched for TIC/EMT signatures are also enriched for MET and FGFR1 signalling pathways

**Fig. 8:**
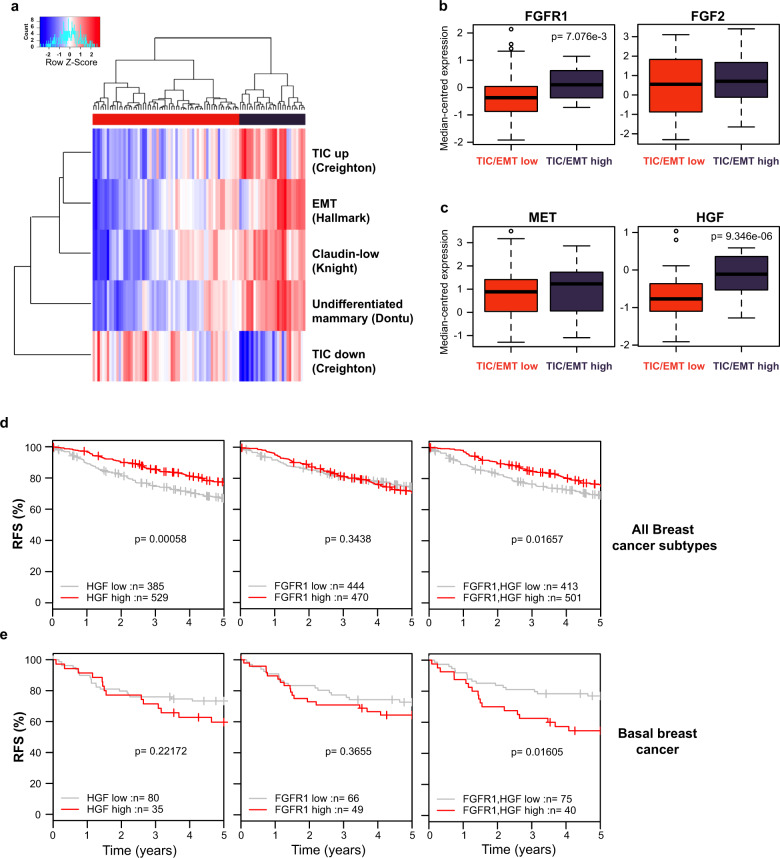
HGF and FGFR1 are co-expressed in highly mesenchymal TNBC tumours and together are associated with poor outcome. **a** Gene expression profiles from triple-negative tumours from the TCGA patient cohort were subjected to ssGSEA for the indicated tumour-initiating cell and mesenchymal gene signatures and stratified into TIC/EMT high and low groups. **b**, **c** Box plots of median-centred expression of the indicated genes in TIC/EMT high and low patient groups. **d**, **e** Relapse-free survival (RFS) analysis across (**d**) all breast subtypes and (**e**) basal breast cancers split by expression HGF and/or FGFR1 expression.

We sought to identify patients who would potentially benefit clinically from combination therapies of MET and FGFR inhibitors. Using single-sample gene set enrichment analysis (ssGSEA), TNBC patients from The Cancer Genome Atlas (TCGA)^54^ dataset of invasive breast carcinomas were clustered into TIC/EMT high and TIC/EMT low cohorts, using published gene signatures for claudin-low breast cancer^36^, TIC^9^, EMT^4^ and undifferentiated mammary epithelial cells^41^ (Fig. 8a). Accordingly, expression of the TIC-related gene *ALDH1A1* was enriched in the TIC/EMT high patients, as were EMT-related genes *SNAI2*, *ZEB1*, *ZEB2*, *TWIST1* and *VIM* (Supplementary Fig. 7a). Comparing expression of genes encoding FGFR1 and MET, as well as their ligands FGF2 and HGF respectively, between TIC/EMT high and low patients revealed that *FGFR1* and *HGF* were significantly increased in the TIC/EMT high group, whereas *FGF2* and *MET* were not significantly different (Fig. 8b, c). Despite this, using GSEA, TIC/EMT high patients scored higher for gene signatures associated with both MET^55^ and FGFR1^56^ signalling activation (Supplementary Fig. 7b), further supporting that TNBCs with mesenchymal features were enriched for components of MET and FGFR1 signalling.

Since highly mesenchymal TNBCs are associated with elevated expression of both *FGFR1* and *HGF*, we assessed whether these two genes in combination could predict outcome in breast cancer patients. Analysis of publicly available breast cancer expression profiles revealed that high expression of both *FGFR1* and *HGF* were associated with poor relapse-free survival (RFS) specifically in basal tumours (Fig. 8d, e). Collectively, these data support the combination targeting of MET and FGFR1 as a feasible therapeutic option for basal B TNBCs.

## Discussion

Increasing evidence supports that TICs can promote intratumoural heterogeneity given their capacity to give rise to multiple different cell types within a single tumour^57,58^ and that this is a key driver of disease relapse. Indeed, targeting TICs is thought to be essential for optimal cancer treatment as their self-renewal, plasticity, and tumourigenic properties are associated with disease recurrence and resistance to standard-care therapies^9,10,12,14–16,57,58^. Triple-negative breast cancer (TNBC) is a heterogeneous disease that lacks both effective patient stratification and therapeutic targets, and where strategies to target TICs are still poorly defined. Here, we identify a co-dependency between the MET and FGFR RTKs for self-renewal of TICs and tumourigenesis of mesenchymal TNBC. This was established by functional studies using transgenic mouse models of TNBC, human TNBC cell lines and PDXs from patients with TNBC. These multiple complementary preclinical and clinical models, together with quantitative biochemical data, and gene expression and TCGA data analyses, support a model whereby MET cooperates with FGFR to regulate TICs, and where dual inhibition of MET and FGFR1-FRS2 signalling results in TIC depletion, hindering tumour progression in TNBC.

A role for MET signalling in TIC biology has been documented in several cancers including glioblastoma, prostate, pancreatic and breast cancers^59–66^, where MET acts as a functional marker of TICs, facilitating their survival under stress such as hypoxia, as well as conferring therapeutic resistance and metastatic potential^59–62,66–68^. This is consistent with our observation that MMTV-*Met*^*mt*^*;Trp53fl/+;Cre* mouse mammary tumours with a spindloid pathology are highly enriched in TIC populations that are dependent on Met signalling for survival, proliferation and self-renewal. FGFR signalling is essential for normal mammary gland development and promotes the growth of TNBC cell lines^47^. FGFR1-3 inhibition with AZD4547 has been reported to selectively target mammary stem cells and TIC populations in an ErbB2-overexpressing breast cancer mouse model^69^. However, while FGFR1 is an independent prognostic marker of survival in TNBC patients^70^, the contribution of FGFR1 signalling in TIC renewal in these tumours remains to established.

The ability of MET and FGFR1 signalling to compensate for one another in support of tumour growth has been identified in various human cancers. In FGFR1-amplified lung cancer cell lines, MET-amplification is a mechanism of resistance to FGFR-targeting drugs^71–74^ and conversely, FGFR1-dependent resistance to MET inhibitors is observed in leukaemia, kidney and prostate cancers^75–77^. Resistance is typically associated with amplification of MET or FGFR1, supporting the ability of MET and FGFR to activate similar key signalling pathways required for tumour progression. Despite extensive research into the therapeutic applications of MET and FGFR inhibitors, studies exploring the potential benefit of targeting both receptors in TNBC are lacking.

Here, we report that MET or FGFR-dependent TIC renewal in TNBC requires tyrosine phosphorylation of the downstream scaffold protein FRS2. FRS2 phosphorylation generates multiple binding motifs for tyrosine phosphatase Shp2 and adaptor protein Grb2, recruiting additional scaffold proteins such as GAB1, and is required for maintenance of ERK1/2 signalling^78–80^. This unexpected crosstalk between these RTKs for FRS2 phosphorylation and TNBC TICs is further supported by in vivo studies where tumour initiation was inhibited only upon combination therapy with Met and FGFR inhibitors. Accumulating evidence highlights the strict requirement of ERK1/2 signalling in the maintenance of TIC properties^81–83^. As both Met and FGFR are robust activators of ERK1/2 signalling, it is improbable that there are unique pathways downstream of ERK1/2 that regulate TIC/stemness properties that cannot be activated by either MET or FGFR alone. Yet transcriptional changes in response to MET and FGFR inhibition are distinct, implying that alternate pathways can be activated and/or the amplitude of the signal may alter transcriptional responses. Whereas Met inhibition alone exerted a limited effect on FRS2 phosphorylation in tumourspheres, Crizotinib was sufficient to reduce FRS2 phosphorylation in tumours to a level similar to FGFR inhibitor.

The specific sensitivity of mesenchymal TNBCs to MET and FGFR inhibitors reflects the dual expression of these RTKs. Although MET is elevated in most basal breast cancer cell lines, we found that both FGFR1 protein and mRNA levels are preferentially enriched in basal B cell lines. Consistent with MET and FGFR1 signalling crosstalk in TNBCs, following FGFR inhibition, basal B cell lines underwent reduction of CD44^+^CD24^−/low^ TIC populations, a process that can be prevented by HGF stimulation, further highlighting the role of MET activity in the maintenance of TICs. Notably, in these TNBC cells, tyrosine phosphorylation of FRS2 can occur via HGF-dependent activation of MET. Although FRS2 tyrosine phosphorylation has been identified in a MET-amplified gastric cancer cell line^84^, this is the first study to establish FRS2 as a common downstream signal in response to ligand activation of non-amplified MET. FRS2 contains a PTB domain for binding to RTKs, including FGFR, RET, ALK and the neurotrophin receptor^82,85,86^. MET can form complexes with multiple RTKs^42,87^ leading to crosstalk downstream from RTKs. We may envision that proximity of MET and FGFR complexes is enriched in highly mesenchymal and invasive cells, subsequently facilitating FRS2 phosphorylation downstream of MET.

We found that TIC/EMT-enriched breast cancers were elevated in gene signatures associated with both MET and FGFR1 signalling pathway activation. Consistent with basal B PDXs, the TIC/EMT high patient group was enriched for co-expression of *FGFR1* and *HGF*. Importantly, we found that co-expression of *HGF* and *FGFR1* predicts poor relapse-free survival specifically among basal breast cancers, providing further support that FGFR1 and MET pathways contribute to the tumourigenicity of highly mesenchymal breast cancers. Accordingly, we found that combined inhibition of MET and FGFR in a basal B PDX resulted in a decrease in sphere-forming and CD44^+^CD24^−/low^ populations, indicative of a loss of TICs and reduction in overall tumour burden. The finding that co-inhibition of MET and FGFR signalling reduces but does not completely abrogate sphere formation and tumour growth in human cell lines and PDXs may suggest the involvement of other compensatory pathways, such as EGFR, which has been associated with mesenchymal TNBCs^88^. We can also envision that MET and FGFR1 inhibition targets a specific subgroup of TICs. This is consistent with data where MET was specifically correlated with ALDH1A3 and CD133 in breast cancers^66^. In addition, phenotypically and functionally distinct TIC populations can exist within a single tumour. Several studies have also documented the spontaneous conversion between TIC and non-TIC states, demonstrating a robust capacity for plasticity^58,89^. These complexities have implications for TIC-targeting therapies and warrant further study. Taken together, our data support that MET and FGFR1-FRS2 pathways cooperate to promote a population of mesenchymal-like TICs in TNBCs and, consistent with other potential therapies targeting mesenchymal-like TIC populations^90,91^, the clinical benefits of inhibiting these receptors are likely maximised if paired with tumour de-bulking by a chemotherapeutic agent.

In summary, we have established that MET and FGFR1 are co-expressed in highly mesenchymal TNBCs and that they co-regulate TICs therein through a compensatory interplay. We demonstrate for the first time to our knowledge, the ability of non-amplified MET to independently phosphorylate FRS2. Finally, the prognostic power of *HGF* and *FGFR1* co-expression in basal breast cancers offers a new strategy for stratifying patients, as well as potential anti-TIC therapeutic targets that warrant further investigation.

## Methods

### Cell culture

Primary mouse cell lines were established by dissociation of MMTV-*Met*^*mt*^, *Trp53fl/+;Cre*, and MMTV-*Met*^*mt*^*;Trp53fl/+;Cre* mammary tumours as previously described^34,36^. Cells were cultured in DMEM (Gibco) supplemented with 5% serum, epidermal growth factor (5 ng/ml), insulin (5 μg/ml), bovine pituitary extract (35 μg/ml) and hydrocortisone (1 μg/ml). BT-20, HCC70, HCC1937, HCC1954, HCC1395, MDA-MB-468, MDA-MB-436, MDA-MB-157, MDA-MB-231, BT-549 and Hs578T were from ATCC and cultured according to ATCC recommendations. All cells were grown at 37 °C and 5% CO_2_.

### Patient-derived xenografts (PDXs)

PDXs have been previously described^53^. Briefly, all human participants provided informed consent for this study, and tissue was collected at McGill University Health Center in accordance with the protocols approved by the research ethics board (SUR-99-780). All animal studies linked to this protocol were approved by the McGill University Animal Care Committee (2014–7514). The Biobank protocol (05-006) and the protocol to generate PDX from biobank tissues (14–168) were both approved by the Jewish General Hospital ethics committee.

### Antibodies and reagents

Commercial antibodies used include Met (R&D System), Claudin-1 (Thermo Fisher), FRS2 (Santa Cruz) and β-actin (Sigma). Antibodies against phospho-Met (Y1234/1235), FGFR1, Akt, phospho-Akt (S473), ERK1/2, phospho-ERK1/2 (T202/Y204), phospho-Frs2 (Y196), phospho-Frs2 (Y436), β-tubulin and cleaved-Caspase-3 were from Cell Signaling. CD24-PE and CD44-FITC for flow cytometry were from BD Biosciences. Antibodies dilutions are provided in Supplementary Table 4.

### Lentiviral infection

Knockdown of *FGFR1* and *Frs2* in A1005 and A1129 cells were carried out using shRNAs cloned into pLKO.1 lentiviral vectors shRNAs (Sigma-Aldrich). The following clones were used for *FGFR1*: TRCN0000023295 (Sequence: CCGGCCTGGAGCATCATA ATGGATTCTCGAGAATCCATTATGATGCTCCAGGTTTTT) and *Frs2*: TRCN0000097281 (sequence: CCGGCCGACAGTCTTTAACTTTGATCTCGAGATCAAAGTTAAAGACTGTCGGTTTTTG) HEK293T cells were transfected using FuGENE HD (Promega) to produce lentivirus. Packaging vectors used were pRSV-Rev, pHCMV-VSVg and pMDLg/pRRE. Media containing viral particles was collected and passed through a 0.45-μm filter. Infected A1005 and A1129 cells with stable knockdown were selected under Puromycin (2 μg/ml) and the drug efflux inhibitor Cyclosporin A (2.5 μM).

### Tumoursphere-formation assays

Single cells were seeded in six-well ultra-low attachments plates (Corning) in 2 ml serum-free DMEM/F12 supplemented with 1× B27, 10 μg/ml insulin (Gibco), 20 ng/ml EGF (BP Bioscience), 20 ng/ml bFGF (StemRD), 10 μg/ml heparin (StemCell Technologies) and 0.5 μg/ml hydrocortisone (Wisent). Tumoursphere number and size were determined after 5–7 days of culture using the software AxioVision (Carl Zeiss). To serially passage, tumourspheres were enzymatically and mechanically dissociated in 0.05% Trypsin-EDTA (Gibco), passed through a 25-G needle, and re-seeded as single cells. All experiments were performed in triplicate.

### Tumoursphere proliferation assays

The proliferation of tumourspheres was determined using the CyQUANT Cell Proliferation Assay Kit (Invitrogen) according to the manufacturer’s instructions with some modifications. Briefly, single cells were seeded in 96-well ultra-low-attachment plates (Corning) and cultured for 5–7 days. Plates were collected on the first and last days of culture and centrifuged at 2000 rpm for 20 min to pellet the tumourspheres. Plates were then inverted to remove media, frozen at −80 °C for a minimum of 24 h, and thawed at room temperature when ready to quantify. The CyQUANT dye/lysis buffer solution was added to all wells, and each plate was analysed using a VarioSkan plate reader (Thermo Scientific). Proliferation was expressed as a ratio of fluorescence on the last day to the first day of culture. All experiments were performed in triplicate.

### Western blot analysis

Tumour-derived cell lines were lysed in 1% Triton lysis buffer (150 mM NaCl, 50 mM HEPES, 1 mM EDTA, 1.5 mM MgCl2, 1% Triton X-100, 4% glycerol), and snap-frozen mammary tumours were lysed as previously described^23^. Whole-cell lysates were resolved by SDS/PAGE and transferred to PVDF membranes. Membranes were blocked with Li-COR Blocking Buffer (Li-COR Biosciences) and probed with primary antibodies overnight at 4 °C. After TBS-Tween washes, membranes were incubated with infrared-conjugated (Li-COR Biosciences) or HRP-conjugated (Cell Signaling and GE Healthcare) secondary antibodies for 1 h at room temperature for signal detection by Odyssey IR Imaging System (Li-COR Biosciences) or enhanced chemiluminescence (Amersham Biosciences) respectively. All blots derive from the same experiment and were processed in parallel.

### Quantitative RT-PCR

RNA was isolated using the RNAeasy Mini Kit (Qiagen) according to the manufacturer’s instructions. cDNA was synthesised using the Transcriptor First Strand cDNA Synthesis Kit (Roche) according to the manufacturer’s instructions. qPCR reactions were performed using SYBR Green I Master on a LightCycler480 (Roche). Primers sequences (Integrated DNA Technologies) are provided in Supplementary Table 5. Normalisation was done using both *Hprt* and *Gapdh* genes for the FGFR transcript quantification and RNA-Seq gene expression validation. For RNA-Seq gene expression validation, the data presented represent the merge of three different cell lines for the DMSO, PD173074 and Crizotinib-treated tumourspheres and two cell lines for the combination-treated tumourspheres.

### Immunohistochemistry

Tumour tissues were formalin-fixed and paraffin-embedded in a previous study^36^, and sections were cut at 4 μm. Sections were deparaffinized xylene and re-hydrated in ethanol, followed by antigen retrieval in Tris-EDTA at boiling temperature. Slides were cooled and blocked with Power Block (BioGenex) and incubated overnight with primary antibody at 4 °C. Rinse slides in distilled water then incubated in 3% H_2_O_2_ for 30 min. SignalStain Boost (Cell Signaling) was used as the secondary antibody, and the SignalStain DAB substrate kit (Cell Signaling) was used to detect signal prior to counterstaining with Harris’ hematoxylin. Finally, slides were dehydrated, mounted, and scanned using Aperio-XT slide scanner (Aperio).

### RNA sequencing

A1005, A1129 and A1471 tumourspheres treated with inhibitors as indicated were collected, and the total RNA was extracted using the AllPrep DNA/RNA Mini Kit (Qiagen) according to the manufacturer’s instructions. High RNA quality was verified using the Bioanalyzer RNA 6000 Nano assay (Agilent). The samples were sequenced using a NextSeq500 sequencer (Illumina) by the Genomics Core Facility of the Institute for Research in Immunology and Cancer, Université de Montréal. Reads were mapped to mouse genome version mm10 using Spliced Transcripts Alignment to a Reference (STAR). Reads counts were normalised using mean-centred and log-transformed. Differentially expressed genes among groups were identified using the R packages DESeq2^92^ and Lima. After unpaired analysis, only genes with False Discovery Rate (FDR) < 0.05, and log2 fold change ≥1.5 were considered. Hierarchical clustering of differentially expressed genes was used to represent the results (R package ggplot2). The gene set enrichment analysis was performed using the Molecular Signature-DB 7.1 and the GSEA software version 4.10^93^ with the complete set of normalised input values, using the Hallmark, canonical pathway gene sets (chemical and genetic perturbations, BioCarta, Reactome and Kegg), and Gene ontology gene sets (Biological process and cellular component). Heatmaps were constructed using Partek software. For all statistical analysis, differences were considered statistically significant if the adjusted *P* values calculated by Student’s *t* test with Bonferroni correction were <0.05. RNA-Sequencing of the tumoursphere (GSE162272). The microarray of the MMTV-*Met*^*mt*^;*Trp53fl*/+;*Cre* tumours (GSE41601) were previously published^36^. RNA-Sequencing of the PDXs (GSE142767) were previously published^94^.

### In vivo limiting dilution assay

Mice were housed in accordance with McGill University Animal Ethics Committee guidelines. A1129 and A1221 cells were resuspended in sterile PBS and injected at decreasing numbers (100, 50 and 10 cells) into the 4th mammary fat pad of female athymic mice aged between 6 and 8 weeks (Taconic Farms). The study was conducted over 12.5 weeks, during which mice were palpated every 2–3 days monitor for tumour outgrowth. TIC frequency was calculated using the Extreme Limiting Dilution Analysis (ELDA) online software available at http://bioinf.wehi.edu.au/software/elda/.

### In vivo inhibitor treatments

For the tumour-initiation study, 100,000 A1129 cells were resuspended in sterile PBS and injected into the 4th mammary fat pad of female athymic mice (Taconic Farms, Inc.). On the day of injection, mice were randomised into four groups (*n* = 10), with each group receiving vehicle control, Crizotinib (50 mg/kg/d, p.o.), BGJ398 (30 mg/kg/d, p.o.), or both drugs in combination. For the tumour progression study, mice were injected with MMTV-*Met*^*mt*^*;Trp53fl/+;Cre* cells (A1129) as above. Once tumours reached 50–100 mm^3^, mice were randomised into four treatment groups (*n* = 10) and gavaged daily as above. Tumour diameters were measured every 2 days with callipers, and tumour volume (mm^3^) was calculated by the following formula: (length × 4.18879)/2 × (width/2)^2^. PDX GCRC1915 tumours were transplanted as fragments into the 4th mammary fat pad of female NSG mice with *HGF*^*tm1.1(HGF)Aveo*^ “humanised” knock-in allele (NSG-hHGFki) (https://www.jax.org/strain/014553). Once tumours reached 50–100 mm^3^, mice were randomised into four treatment groups (*n* = 5) and gavaged daily as above. Tumour growth was monitored and volumes calculated as above.

### Flow cytometry

Single cells were stained with fluorophore-conjugated antibodies in 100 μl of PBS with 2% FBS for 30 min on ice protected from light. Cells were washed twice then resuspended in PBS with 2% FBS, and viability dye 7-AAD (eBioscience) was added to each sample. Multi-colour cell sorting was performed on a FACS CantoII (BD Biosciences) and data analysis was performed using FlowJo (Tree Star Inc.). An example of gating is presented in Supplementary Fig. S8.

### Immunofluorescence

Tumour tissues were formalin-fixed and paraffin-embedded, and sections were cut at 4 μm. Sections were deparaffinized in xylene and re-hydrated in ethanol, followed by citrate buffer (pH 6) at boiling temperature. Slides were cooled and blocked with 2% BSA and incubated overnight with primary antibody at 4 °C. Slides were rinsed in distilled water, treated with 3% H_2_O_2_ for 30 min, incubated with secondary antibody for 45 min at room temperature, and counterstained with 0.5 ng/ml DAPI. Tyramide signal amplification (Thermo Fisher) was used according to the manufacturer’s instructions. Finally, slides were imaged with an LSM800 confocal microscope and analysed using Zen software (Zeiss).

### Tumour dissociation

Tumours were minced using razor blades and transferred to a 50-ml conical tube containing tissue digestion media (DMEM + 2.5 FBS with collagenase IV). Tubes were placed in rotating ovens and to dissociate over 2–3 h at 37 °C. The suspension was centrifuged to pellet the epithelial and fibroblast cell content. The pellet was then resuspended in warm PBS, and the epithelial organoids/cells were allowed to gravity sediment for ~5 min. The stromal cell-rich supernatant was removed, and the epithelial content washed in warm PBS then pelleted. The pellet was then trypsinized in 0.05% Trypsin-EDTA and passed through a 40-μm cell strainer to generate a single-cell suspension. Murine stromal cells were further removed using a Mouse Cell Depletion Kit (Miltenyi) according to the manufacturer’s instructions.

### Analysis of gene expression data

The R package GSVA^95^ was used to analyse data from TNBC patients of the TCGA dataset^41^. In order to enrich for patients with high expression of TIC or EMT gene signatures, ssGSEA was applied using the indicated published gene signatures. Welch two-sample *t* test was used to compare the expression of single genes, as well as MET and FGFR1 activation signatures between TIC/EMT high and TIC/EMT low patient cohorts.

The gene expression-based outcome for breast cancer online (GOBO) tool (http://co.bmc.lu.se/gobo/) was used to determine the association of HGF, FGFR1, EGF and EGFR expression with relapse-free survival across a large set of breast cancers analysed by Affymetrix U133A arrays^96^.

### Statistical analysis

Quantitative data are presented as means ± SEM. Statistical significance was assessed using a two-tailed Student’s *t* test, and ordinary one-way ANOVA with Tukey’s correction for multiple comparisons, unless otherwise indicated, using Prism software. Significance is as follows: *P* > 0.05, not significant (ns); ^∗^*P* ≤ 0.05; ^∗∗^*P* ≤ 0.01; ^∗∗∗^*P* ≤ 0.001; ^∗∗∗∗^*P* ≤ 0.0001. Data distribution was assumed to be normal, but this was not formally tested. *P* values and the number of experiments used for quantification and statistical analysis are indicated in the corresponding figure legends.

### Reporting summary

Further information on research design is available in the Nature Research Reporting Summary linked to this article.

## Supplementary information

Supplementary Information

Reporting Summary Checklist

## Data Availability

Processed RNA-sequencing datasets generated during the study are available in Gene expression Omnibus: https://identifiers.org/geo:GSE162272^97^. The raw RNA-sequencing data are available in Sequence Read Archive: https://identifiers.org/ncbi/insdc.sra:SRP294504^98^. All other datasets generated and analysed during the study (including tumoursphere-formation assays, tumoursphere proliferation assays, immunohistochemistry data, quantitative RT-PCR, in vivo inhibitor treatments (including tumour volume calculations), flow cytometry data and immunofluorescence data) are publicly available in the figshare repository: 10.6084/m9.figshare.13519181^99^. The publicly available TCGA data analysed during the study are available in cBioPortal for Cancer Genomics: https://identifiers.org/cbioportal:brca_tcga_pub^100^. Microarray data from the MMTV-*Met*^*mt*^;*Trp53fl*/+;*Cre* tumours analysed during the study, are available in Gene Expression Omnibus: https://identifiers.org/geo:GSE41601^101^. RNA-sequencing data from breast cancer pairs of primary tumours and PDXs, analysed during the study, are also available in Gene Expression Omnibus: https://identifiers.org/geo:GSE142767^102^. Uncropped western blots are part of the supplementary files that accompany the article^97–102^.
