# Supplementary material for: Co-dependency for MET and FGFR1 in basal triple-negative breast cancers
Source: NPJ Breast Cancer. 2021 Mar 26;7:36. doi: 10.1038/s41523-021-00238-4 (PMC7997957; doi:10.1038/s41523-021-00238-4)
Supplement: Supplementary file 1 — Supplementary Information [file 41523_2021_238_MOESM1_ESM.pdf]

Supplementary Information

Supplemental Figures and Tables:

Supplementary Figure 1. MMTV-*Met*^{mt}*;Trp53fl*/+;*Cre* spindloid tumour-derived are enriched in mesenchymal and stem cell gene signatures. **a** Unsupervised hierarchical clustering of 3 independent MMTV-*Met*^{mt};*Trp53fl*/+;*Cre* tumours with spindloid or non-spindloid pathology from microarray analysis (GSE41601). **b-c** Significantly modulated genes in spindloid tumours compared to non-spindloid tumours were subjected to MSigDB (FDR<0.05, log2fold change ≥1.5). Graphs showing significant enrichment of genes signatures associated with positive regulation of stem cells and negative regulation of differentiation. **d** Heatmap representation of the relative expression of known phenotypic markers for each indicated cellular process.

Supplementary Figure 2. Frs2 is required to facilitate compensatory signaling between Met and FGFR1. **a** A1005 and A1129 cells were transfected with empty vector or shRNA against Frs2, and efficient knockdown of Frs2 was verified by Western blot analysis. **b** The effect of Frs2 knockdown on proliferation in adherent condition was assessed for 96h by Trypan blue assay. **c** The effect of Frs2 knockdown on tumoursphere formation in the presence or absence of 1µM Crizotinib was assessed after 5 days of culture. Student's t-test; **p≤ 0.01. Error bars indicate SEM.

Supplementary Figure 3. Human Basal B breast cancer cell lines are enriched in expression of FGFR1. **a-d** mRNA z-scores of MET, FGFR1, 2, 3 and 4 were acquired from the Cancer Cell Line Encyclopedia, and co-expression of MET and each FGFR was compared among human breast cancer cell lines. Student's t-test; *p≤ 0.05; **p≤ 0.01; ***p≤ 0.001.

Supplementary Figure 4. MET and FGFR signaling regulate TICs in Basal B breast cancer cell lines. **a** Scatter plots of CD44 and CD24 expression as detected by flow cytometry in Hs578t cells treated with Crizotinib and/or PD173074 (1µM for both) and stimulated with HGF (50ng/ml) or bFGF (20ng/ml). **b** BT549 cells were treated for 2h, followed by 30min stimulation with HGF or bFGF. The proportion of $CD44^{+}CD24^{-}$ cells was determined by flow cytometry 72h later. **c** Protein lysates from BT549 and Hs578t tumourspheres treated with Crizotinib and/or PD173074 for 2h, followed by 30min stimulation with HGF or bFGF, were analyzed by Western blot. Student's t-test; ***p≤ 0.001, ****p≤ 0.0001. Error bars indicate SEM.

Supplementary Figure 5. Basal B TNBC PDXs are enriched in expression of HGF and FGFR1-FRS2. **a** Unsupervised hierarchical clustering of a published claudin-low (Basal B) breast cancer gene expression signature stratified the PDXs into Basal A and Basal B subtypes from RNA-Seq analysis (GSE142767). **b** Basal B PDXs correlate with increased HGF, FGFR1, and ERK1/2 signaling pathways. **c** Gene expression profiles from Basal B (high FGFR1) and Basal A PDXs (low FGFR1) were subjected to GSEA analysis. Basal B PDXs show significant enrichment of genes signature associated with positive regulation

of stem cells and negative regulation of cell differentiation. Selected enrichment plots for the indicate gene signatures are shown. **d** Heatmap representation of relative expression of known phenotypic markers for each indicated cellular process.

Supplementary Figure 6. MET and FGFR signaling regulate TICs in PDXs. **a** Immunofluorescence was performed on GCRC 1886 and GCRC1863 tumour sections to validate not every PDX has MET and FGFR1 co-expression in the same cell. Scale bars: 100μm. **b** Scatter plot of CD44 and CD24 expression as detected by flow cytometry in GCRC1915 xenografts treated with Crizotinib and/or BGJ398.

Supplementary Figure 7. Highly mesenchymal TNBCs are enriched for gene signatures associated with MET and FGFR1 signaling activation. **a** Box plots of median-centred expression of the indicated genes in TIC/EMT high and low patient groups. **b** Box plots of ssGSEA enrichment scores for MET and FGFR1 activation gene signatures in TIC/EMT high and low patient groups.

Supplementary Figure 8. Gating strategy for evaluating CD24 and CD44 positive cells by flow cytometry in cell lines and dissociated tumours.

Supplementary Figure 9. Raw data for the uncropped blots included in each figure and supplementary figure.

Supplementary Table 1. List of the most significant differentially expressed genes in Crizotinib versus DMSO treated tumourspheres

Supplementary Table 2. List of the most significant differentially expressed genes in PD173074 versus DMSO treated tumourspheres

Supplementary Table 3. List of the most significant differentially expressed genes in Combo versus DMSO treated tumourspheres

Supplementary Table 4. Antibody dilutions

Supplementary Table 5. Primers sequences

Supplementary Figure 1

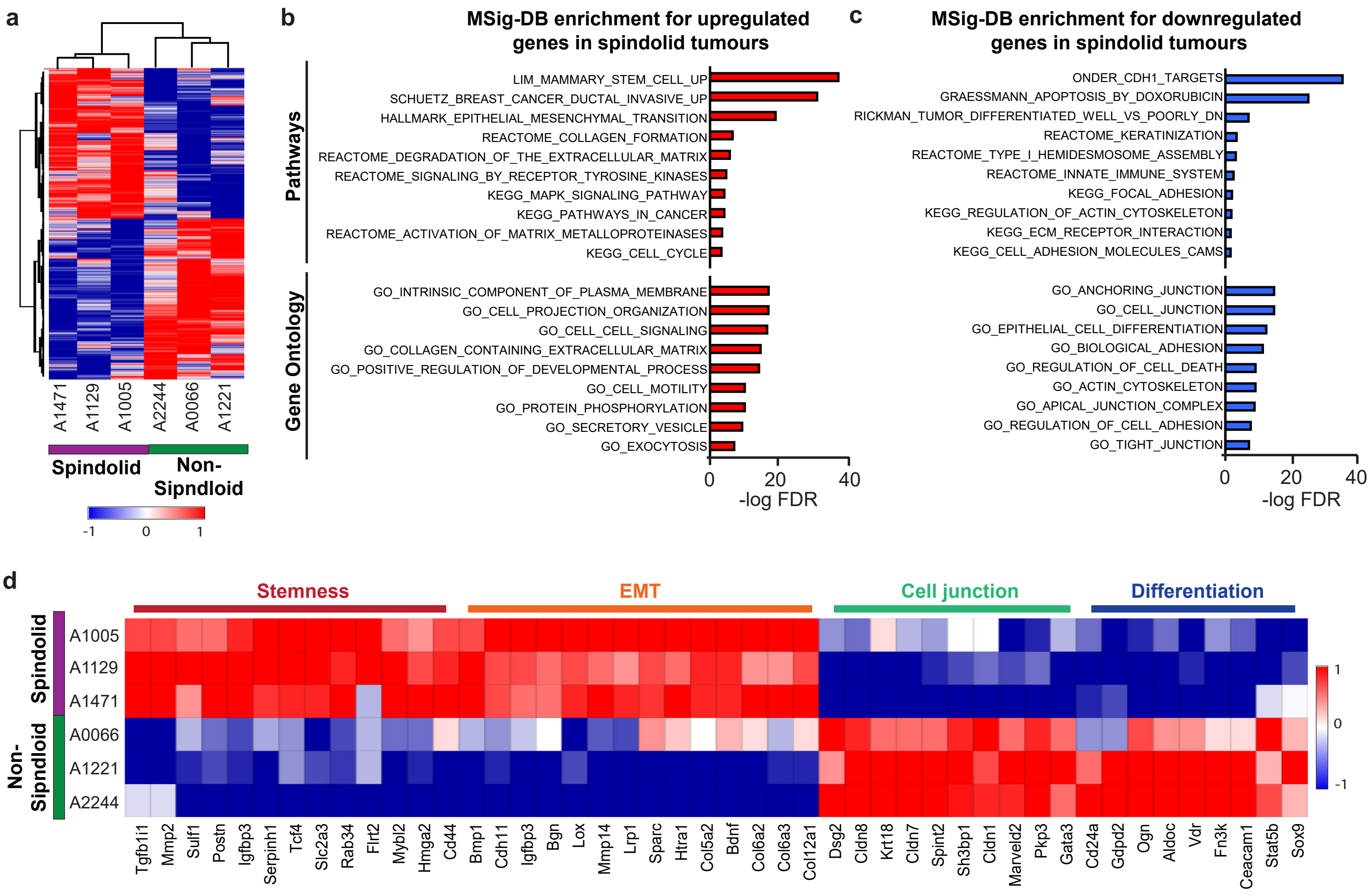

Supplementary Figure 1. MMTV-*Met*^{mt};*Trp53fl/+;Cre* spindloid tumour-derived are enriched in mesenchymal and stem cell gene signatures. a Unsupervised hierarchical clustering of 3 independent MMTV-*Met*^{mt};*Trp53fl/+;Cre* tumours with spindloid or non-spindloid pathology from microarray analysis (GSE41601). b-c Significantly modulated genes in spindloid tumours compared to non-spindloid tumours were subjected to MSigDB (FDR<0.05, log2fold change ≥1.5). Graphs showing significant enrichment of genes signatures associated with positive regulation of stem cells and negative regulation of differentiation. d Heatmap representation of the relative expression of known phenotypic markers for each indicated cellular process.

Supplementary Figure 2

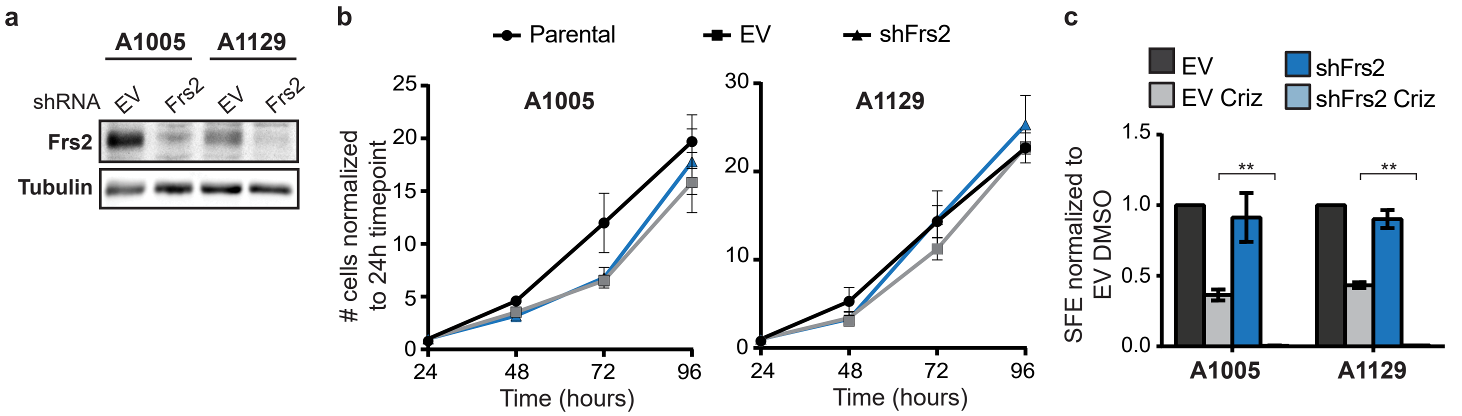

Supplementary Figure 2. Frs2 is required to facilitate compensatory signaling between Met and FGFR1. a A1005 and A1129 cells were transfected with empty vector or shRNA against Frs2, and efficient knockdown of Frs2 was verified by Western blot analysis. b The effect of Frs2 knockdown on proliferation in adherent condition was assessed for 96h by Trypan blue assay. c The effect of Frs2 knockdown on tumoursphere formation in the presence or absence of 1μ M Crizotinib was assessed after 5 days of culture. Student's t-test; **p≤ 0.01. Error bars indicate SEM.

Supplementary Figure 3

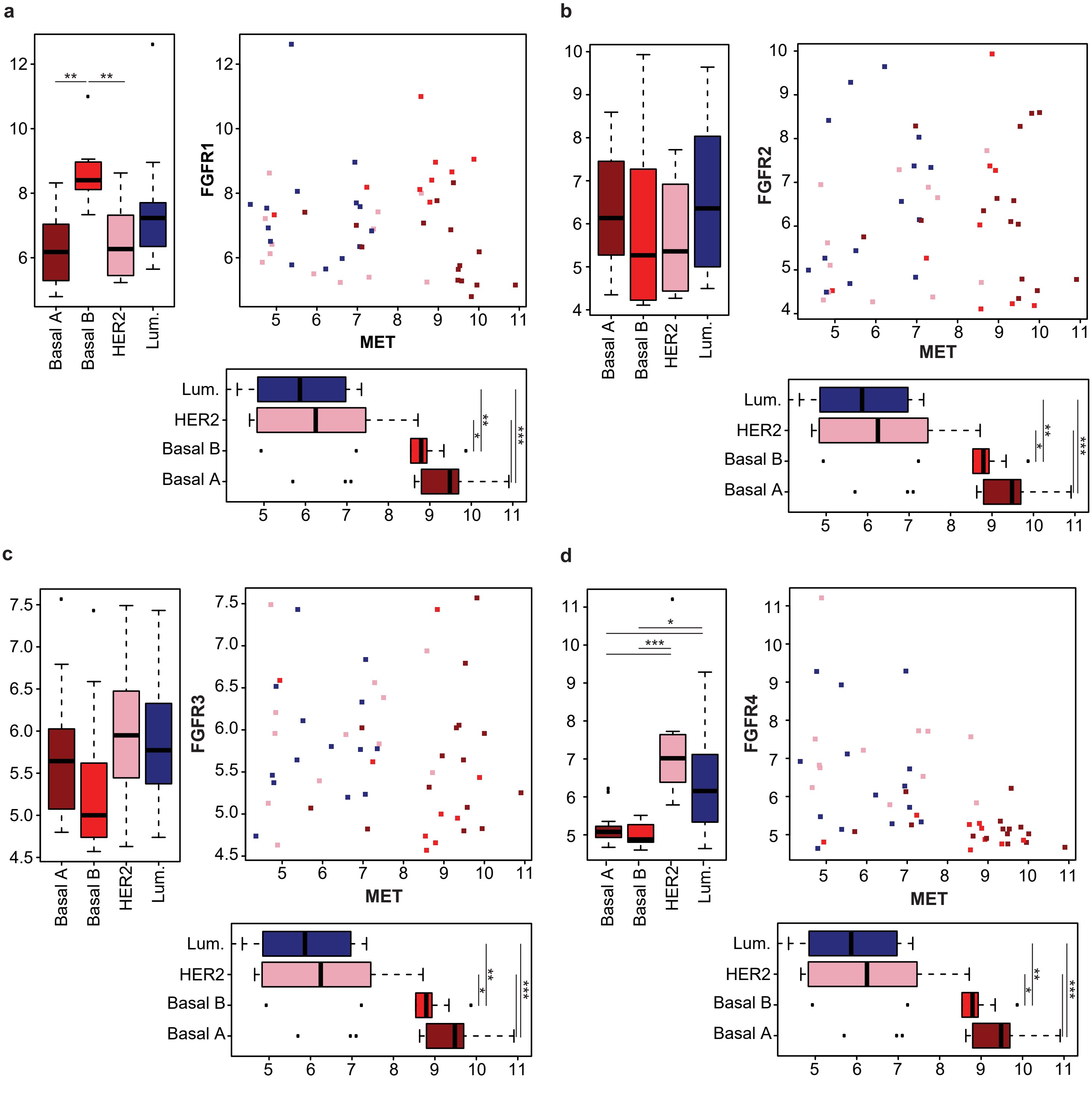

Supplementary Figure 3. Human Basal B breast cancer cell lines are enriched in expression of FGFR1. a-d mRNA z-scores of MET, FGFR1, 2, 3 and 4 were acquired from the Cancer Cell Line Encyclopedia, and co-expression of MET and each FGFR was compared among human breast cancer cell lines. Student's t-test; *p≤ 0.05; **p≤ 0.01; ***p≤ 0.001.

Supplementary Figure 4

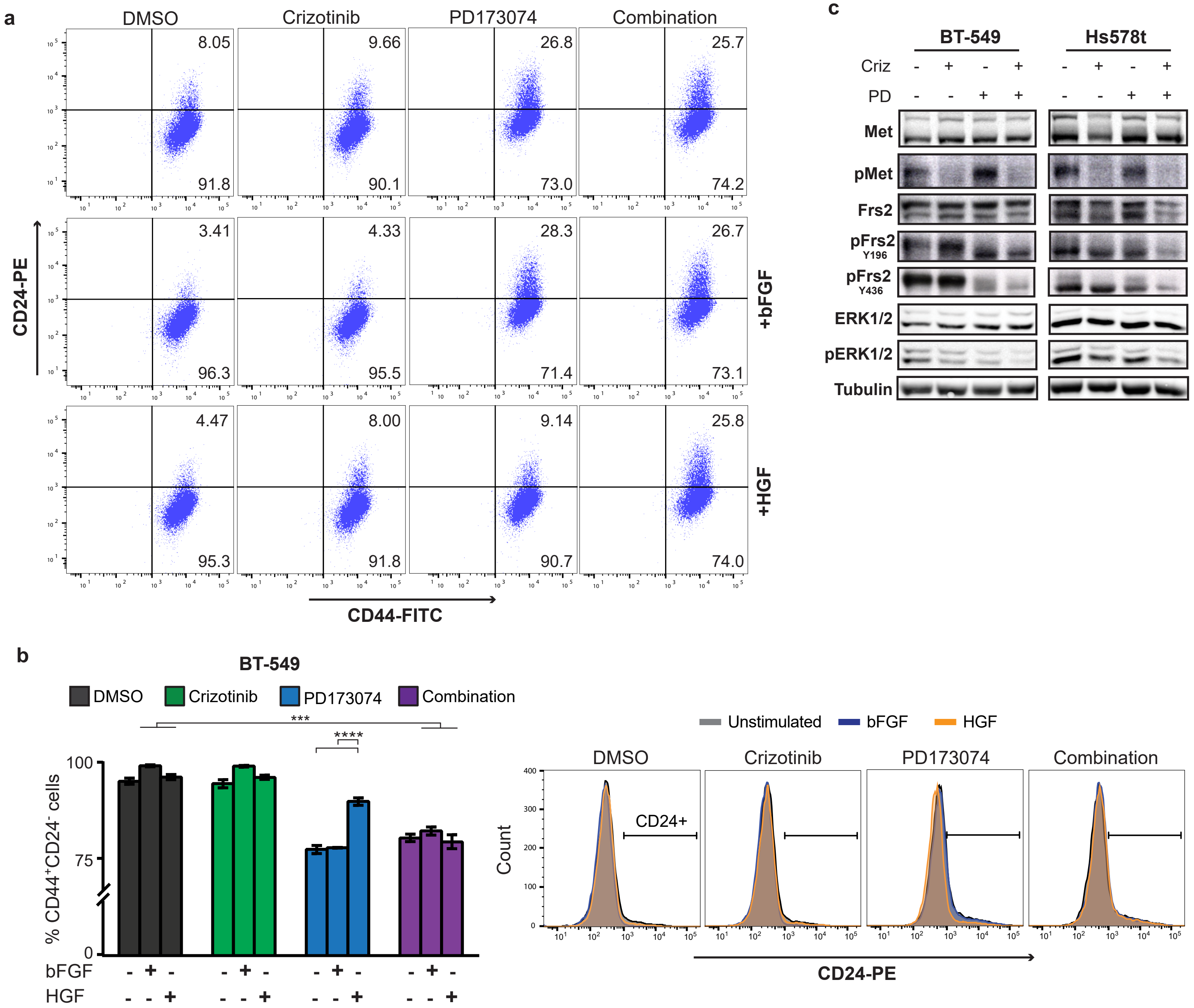

Supplementary Figure 4. MET and FGFR signaling regulate TICs in Basal B breast cancer cell lines. a Scatter plots of CD44 and CD24 expression as detected by flow cytometry in Hs578t cells treated with Crizotinib and/or PD173074 (1µM for both) and stimulated with HGF (50ng/ml) or bFGF (20ng/ml). b BT549 cells were treated for 2h, followed by 30min stimulation with HGF or bFGF. The proportion of CD44+CD24- cells was determined by flow cytometry 72h later. c Protein lysates from BT549 and Hs578t tumourspheres treated with Crizotinib and/or PD173074 for 2h, followed by 30min stimulation with HGF or bFGF, were analyzed by Western blot. Student's t-test; ***p≤ 0.001, ****p≤ 0.0001. Error bars indicate SEM.

Supplementary Figure 5

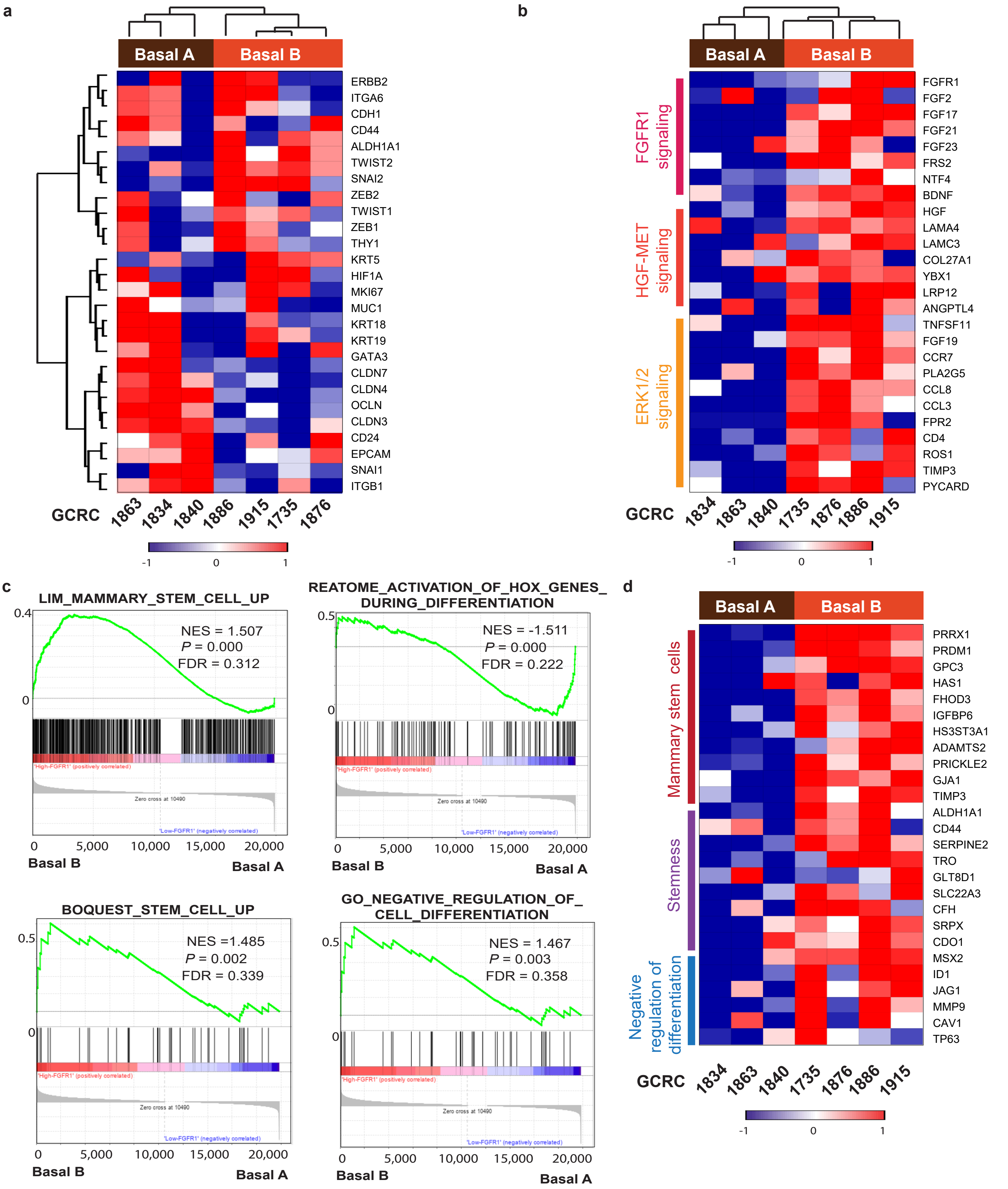

Supplementary Figure 5. Basal B TNBC PDXs are enriched in expression of HGF and FGFR1-FRS2. a Unsupervised hierarchical clustering of a published claudin-low (Basal B) breast cancer gene expression signature stratified the PDXs into Basal A and Basal B subtypes from RNA-Seq analysis (GSE142767). b Basal B PDXs correlate with increased HGF, FGFR1, and ERK1/2 signaling pathways. c Gene expression profiles from Basal B (high FGFR1) and Basal A PDXs (low FGFR1) were subjected to GSEA analysis. Basal B PDXs show significant enrichment of genes signature associated with positive regulation of stem cells and negative regulation of cell differentiation. Selected enrichment plots for the indicate gene signatures are shown. d Heatmap representation of relative expression of known phenotypic markers for each indicated cellular process.

Supplementary Figure 6

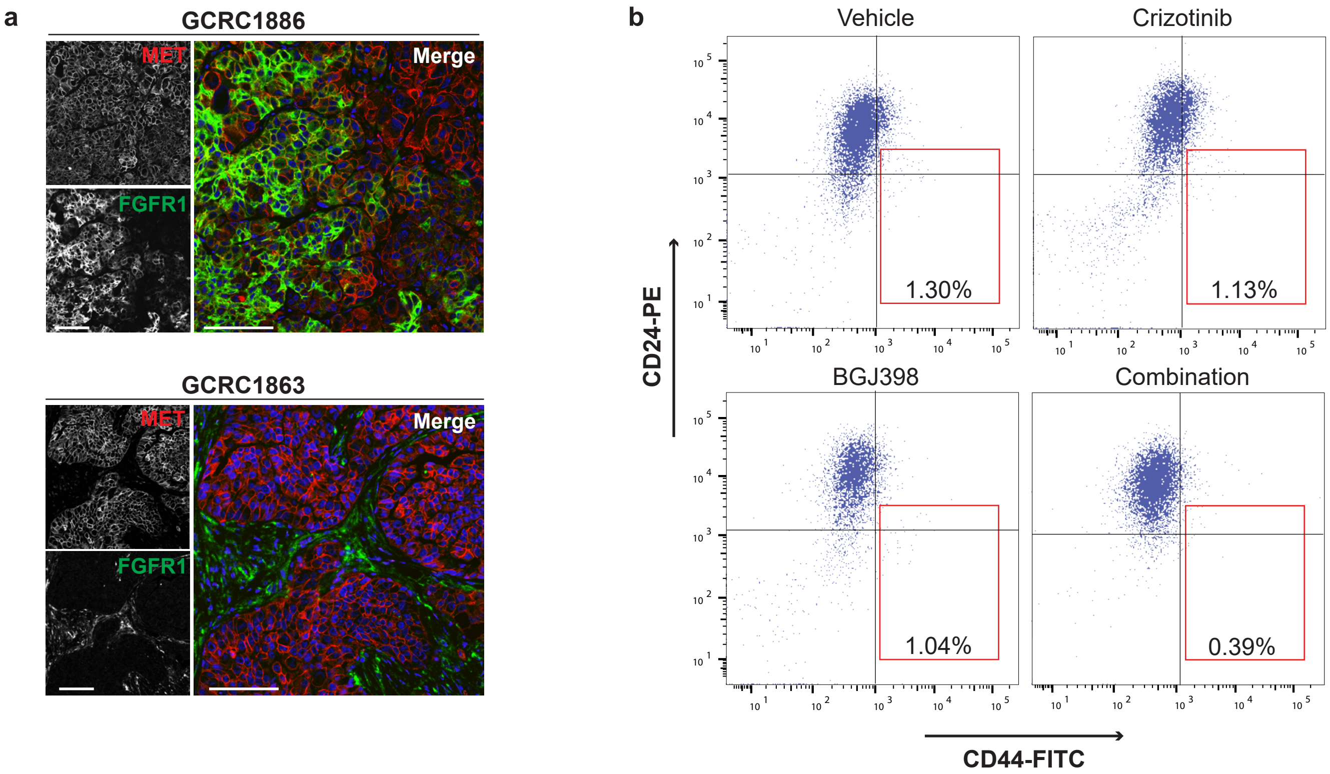

Supplementary Figure 6. MET and FGFR signaling regulate TICs in PDXs. a Immunofluorescence was performed on GCRC 1886 and GCRC1863 tumour sections to validate not every PDX has MET and FGFR1 co-expression in the same cell. Scale bars: 100µm. b Scatter plot of CD44 and CD24 expression as detected by flow cytometry in GCRC1915 xenografts treated with Crizotinib and/or BGJ398.

Supplementary Figure 7

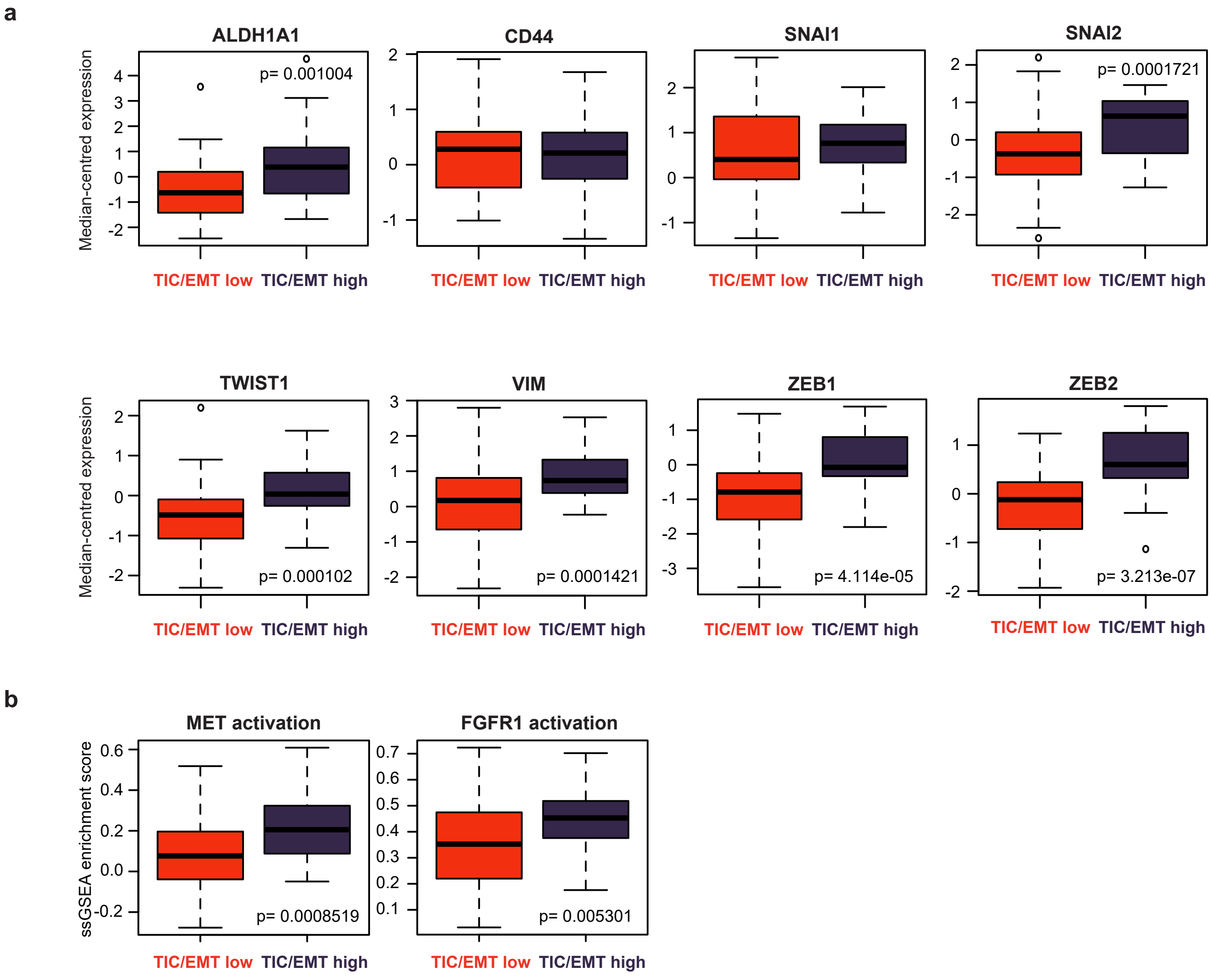

Supplementary Figure 7. Highly mesenchymal TNBCs are enriched for gene signatures associated with MET and FGFR1 signaling activation. a Box plots of median-centred expression of the indicated genes in TIC/EMT high and low patient groups. b Box plots of ssGSEA enrichment scores for MET and FGFR1 activation gene signatures in TIC/EMT high and low patient groups.

Supplementary Figure 8

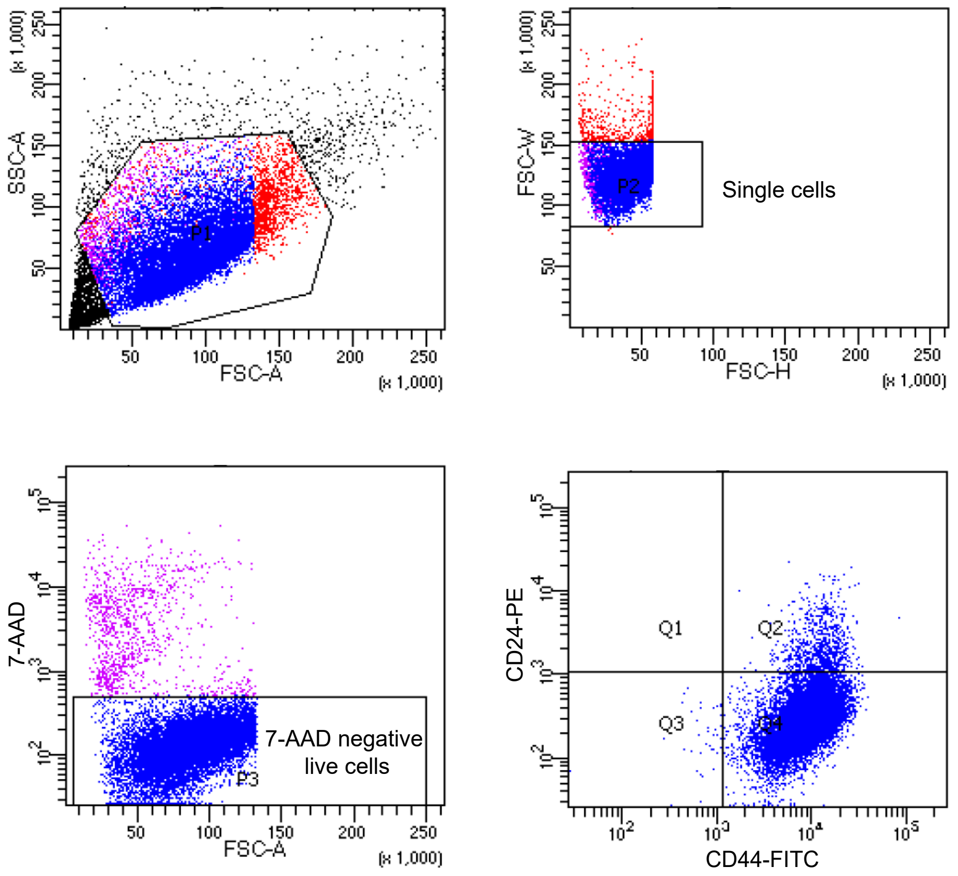

Supplementary Figure 8. Gating strategy for evaluating CD24 and CD44 positive cells by flow cytometry in cell lines and dissociated tumours.

Supplementary Figure 9.

Figure 2B

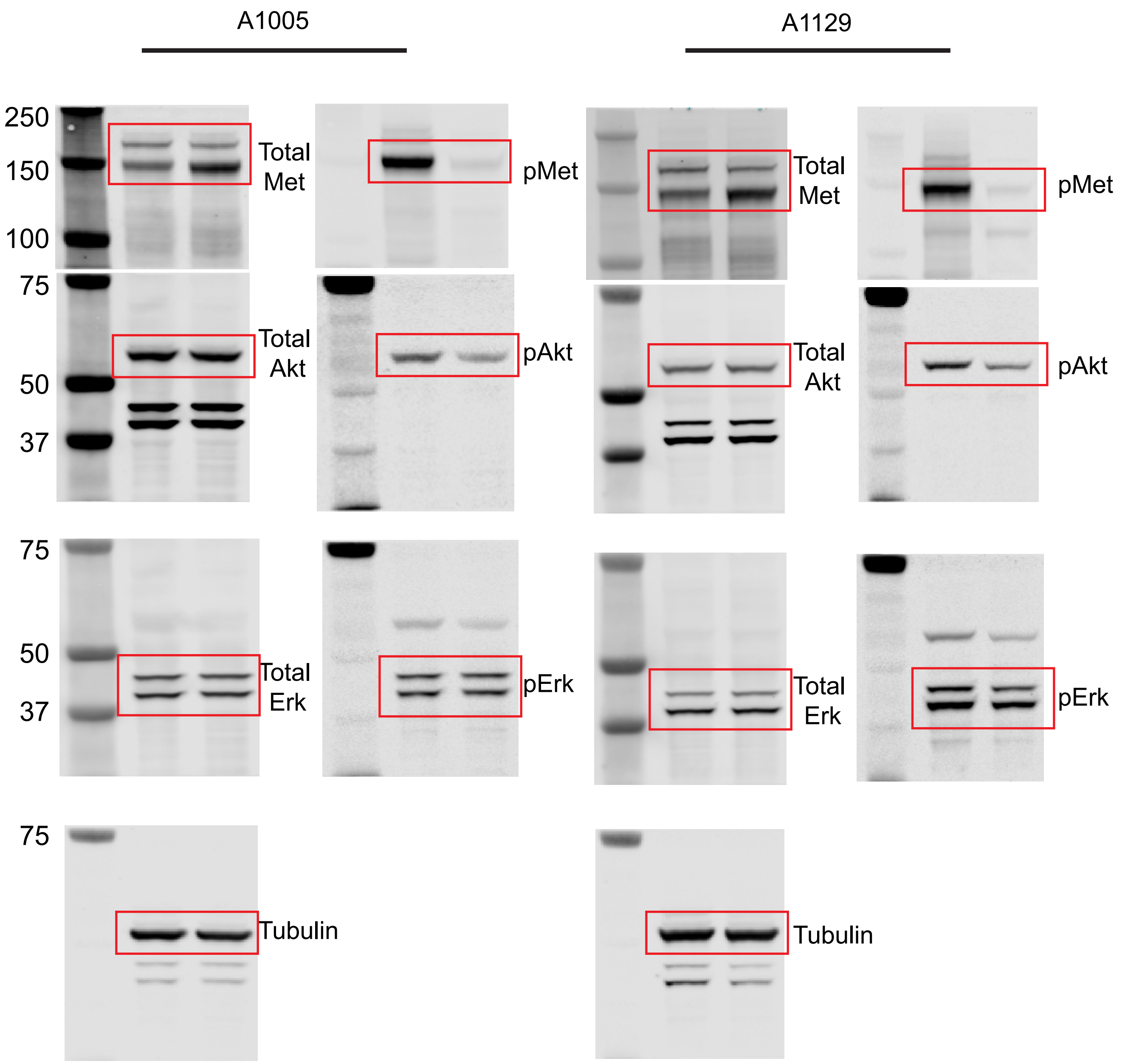

Supplementary Figure 9. Raw data for the uncropped blots included in each figure and supplementary figure.

Un-cropped images of all blots from Fig. 2b.

Figure 2F

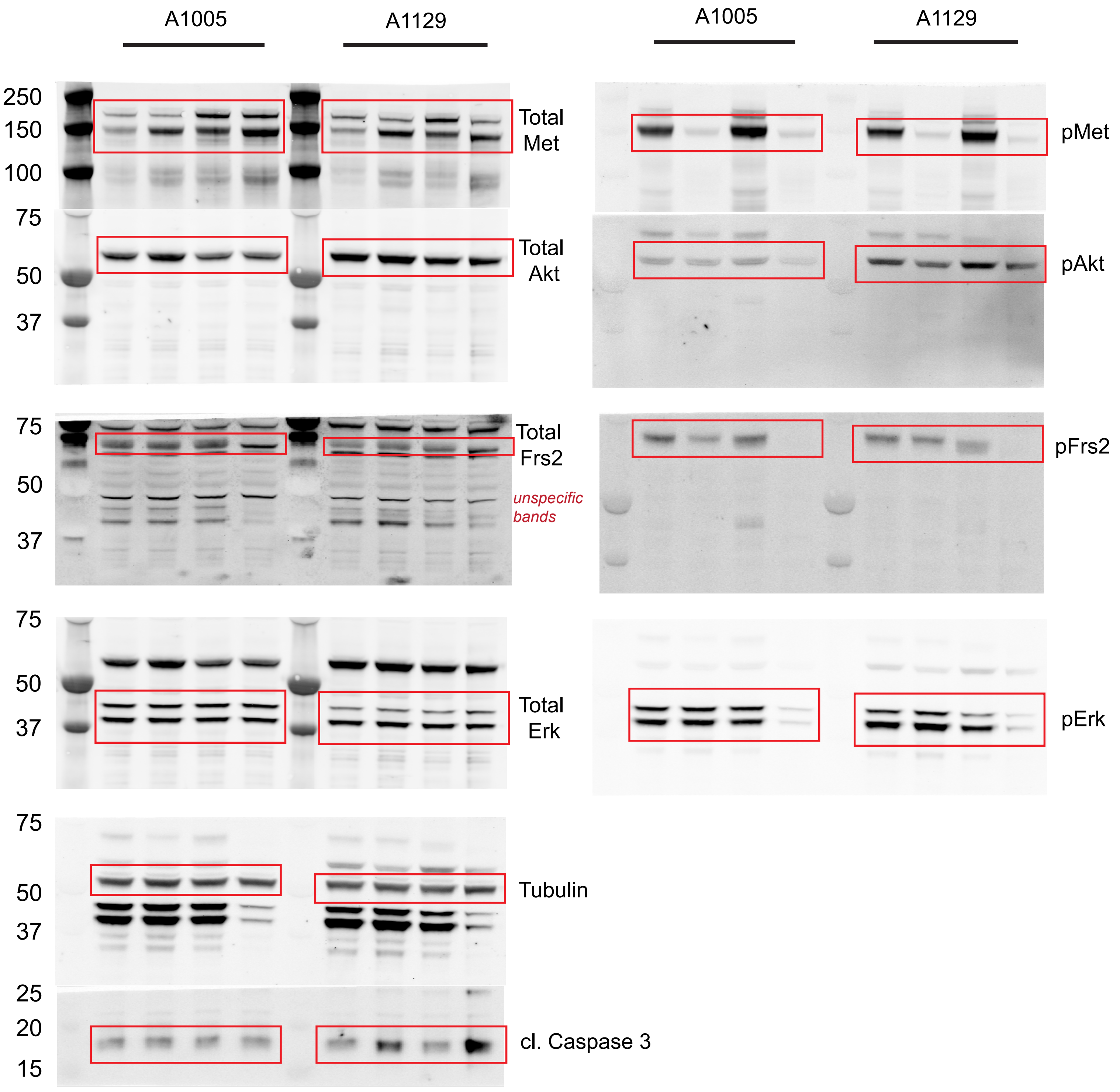

Un-cropped images of all blots from Fig. 2f.

Figure 3B

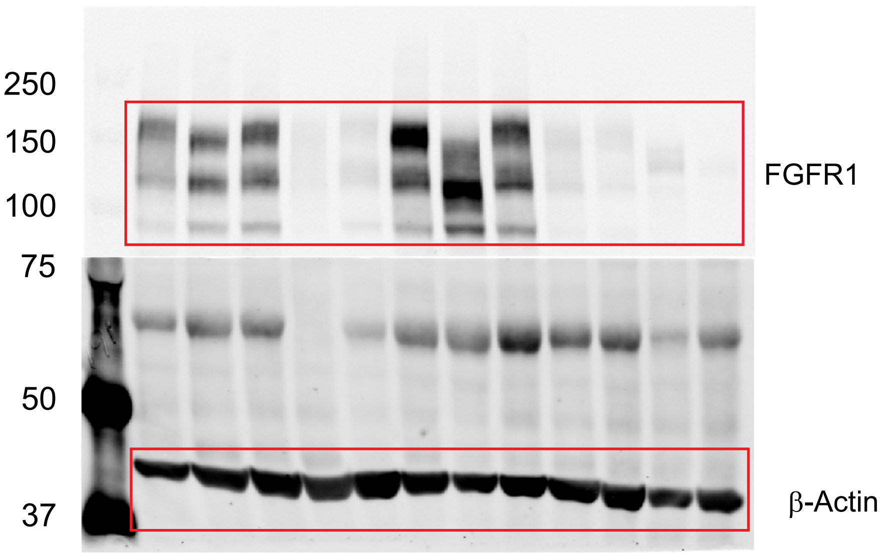

Un-cropped images of all blots from Fig. 3b.

Figure 3D

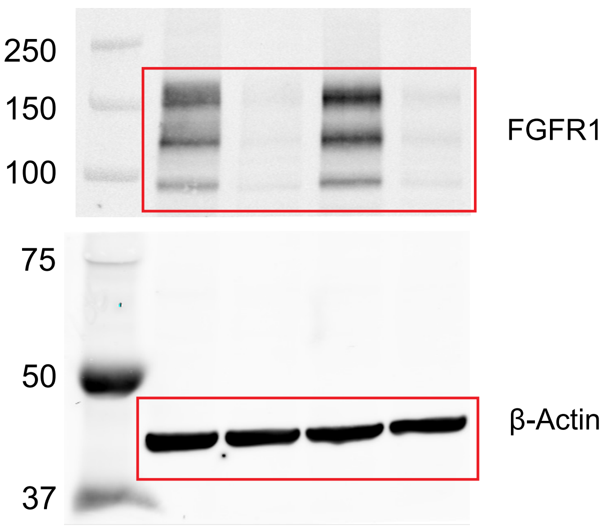

Un-cropped images of all blots from Fig. 3d.

Figure 5F

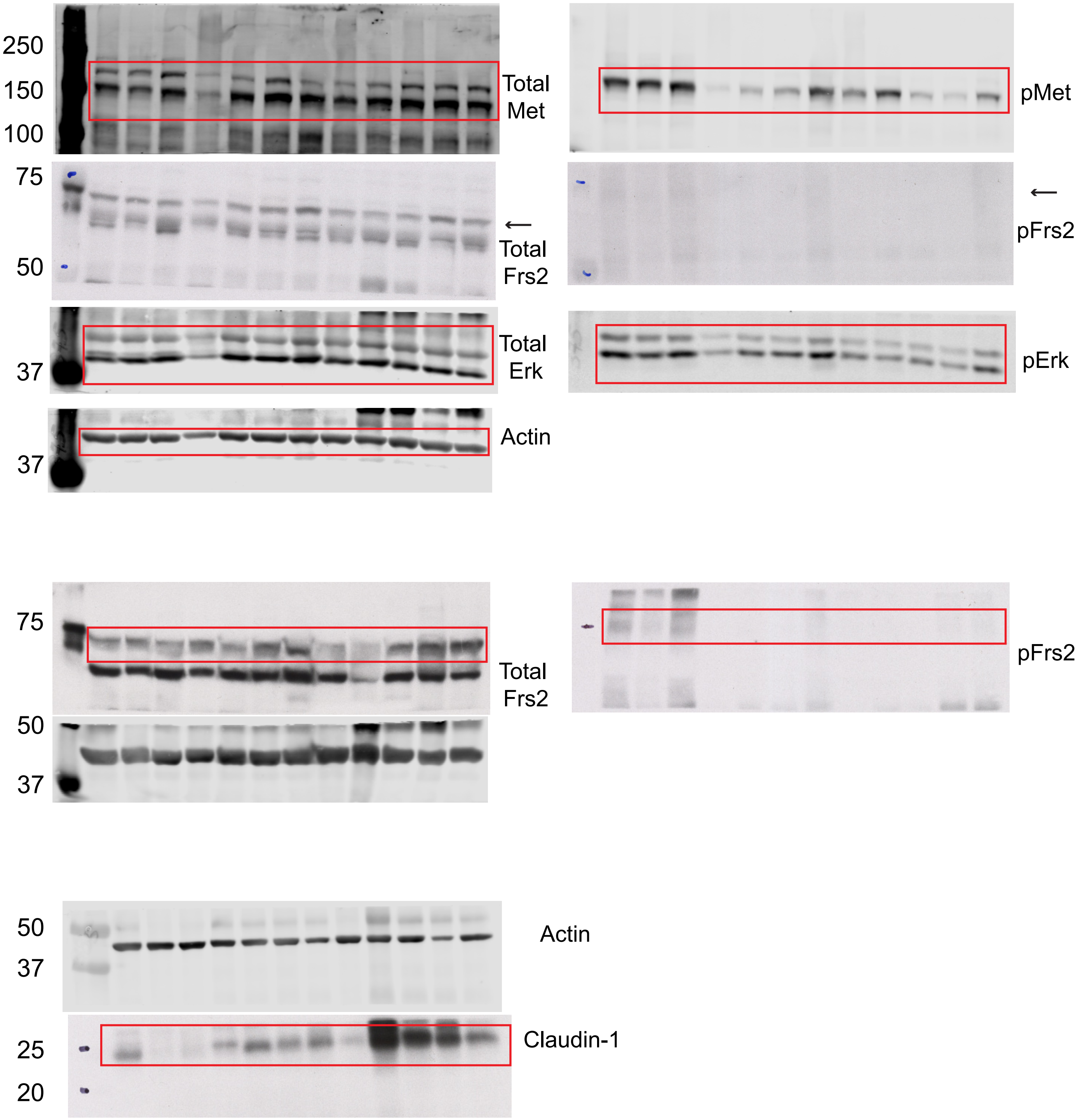

Un-cropped images of all blots from Fig. 5f.

Figure 6A

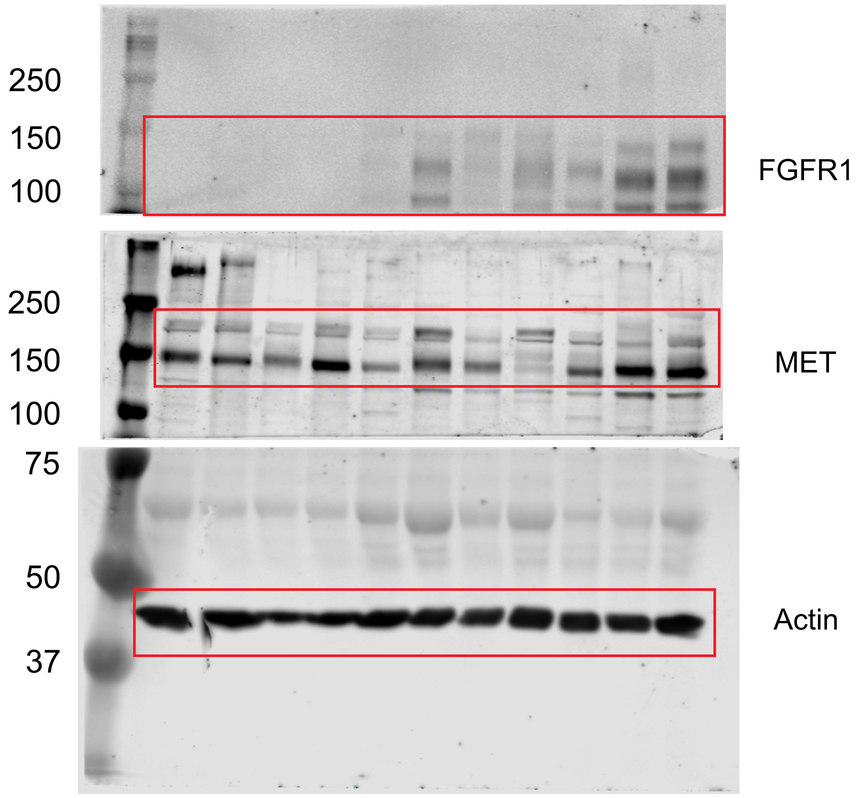

Un-cropped images of all blots from Fig. 6a.

Figure 7A

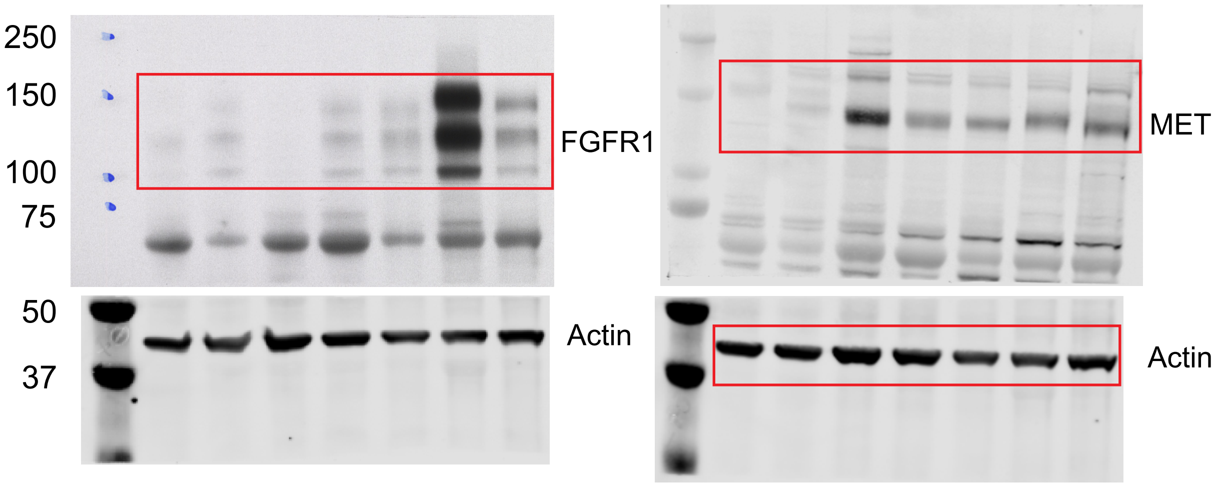

Un-cropped images of all blots from Fig. 7a.

Figure S2A

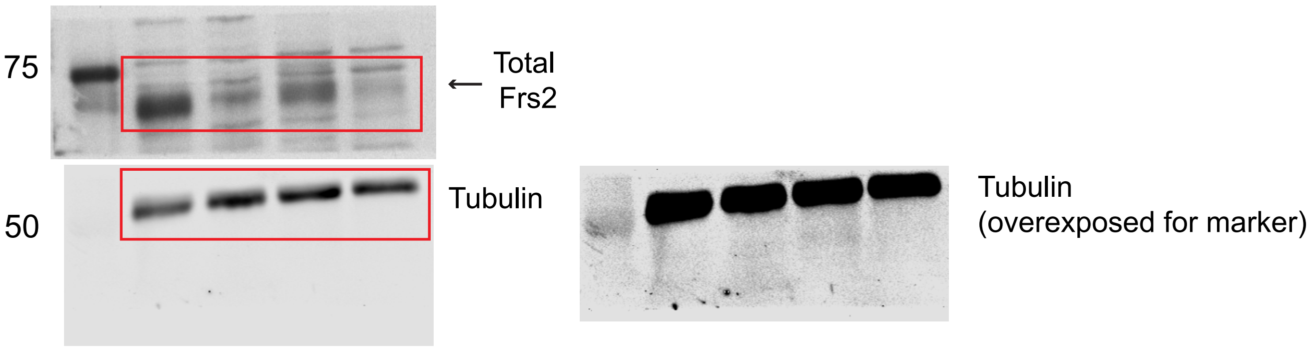

Un-cropped images of all blots from Fig. S2a.

Figure S4C

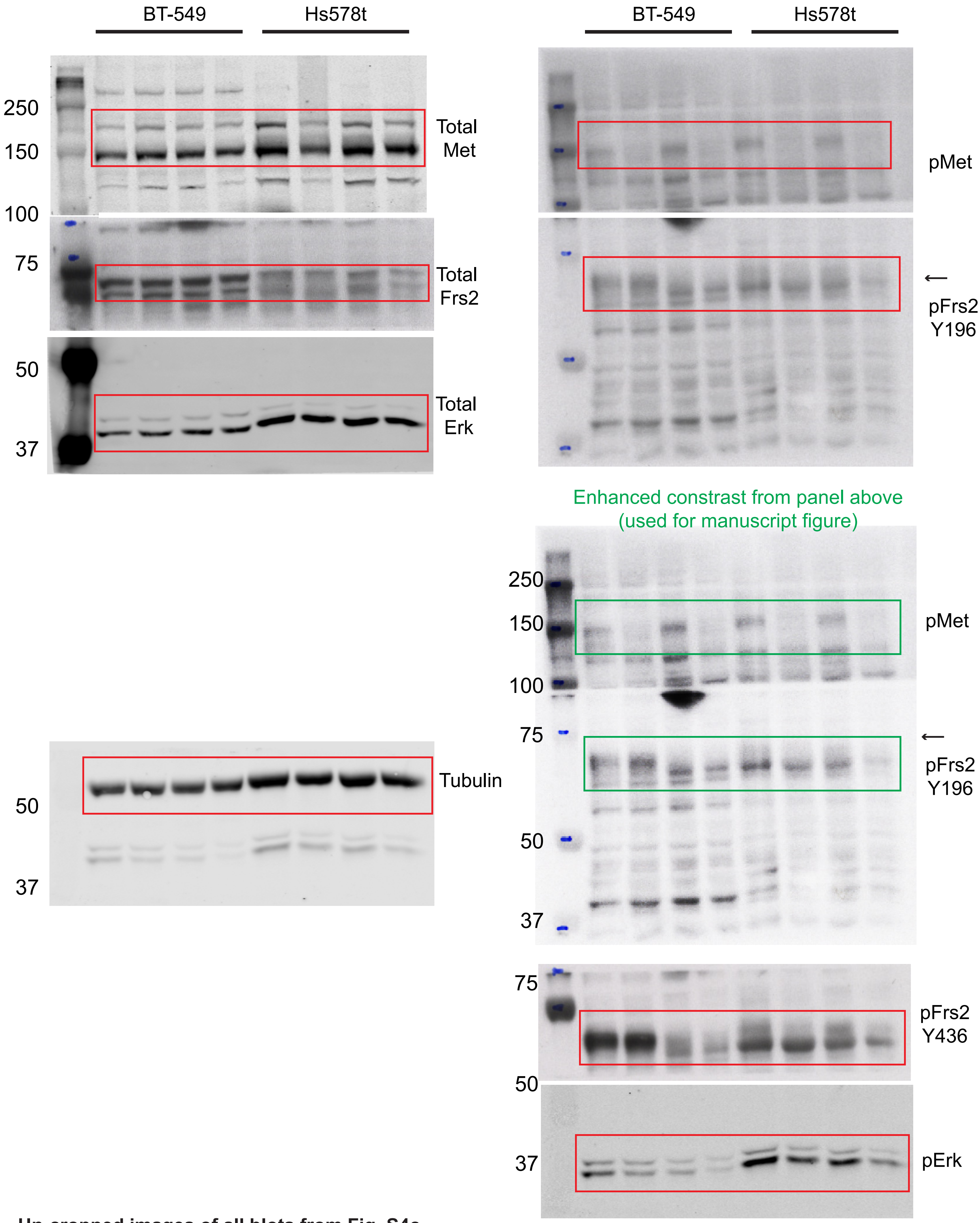

Un-cropped images of all blots from Fig. S4c.

Supplementary Table 1. List of the most significant differentially expressed genes in Crizotinib versus DMSO treated tumourspheres

GeneID	logFC	AveExpr	t	P.Value	adj.P.Val	B
AC127256.1	-2.6674	-3.2483	-4.3208	0.001	1	-4.5445
Duox2	-2.5484	-3.4688	-4.32	0.001	1	-4.5446
Tgtp1	-3.7095	-2.3942	-4.2998	0.001	1	-4.5458
Tgtp2	-3.6299	-2.32	-4.2489	0.0011	1	-4.5462
Gm5865	2.0935	-1.7707	4.1251	0.0014	1	-4.5186
Gm6093	-3.2539	-1.0396	-4.0742	0.0016	1	-4.5428
Gm16279	2.4423	-1.929	4.0027	0.0018	1	-4.5329
Hspa1l	-2.0501	-3.3399	-3.8501	0.0023	1	-4.5497
Gm12356	1.343	-0.9862	3.7822	0.0026	1	-4.4907
Wwtr1	0.3418	8.1451	3.7196	0.0029	1	-3.3083
Spc24	-0.4054	4.3535	-3.6809	0.0032	1	-3.6267
5330438D12Rik	1.4383	-0.4569	3.67	0.0032	1	-4.5006
Gm36527	2.1142	-2.6678	3.6623	0.0033	1	-4.541
Iigp1	-4.8314	1.0321	-3.6383	0.0034	1	-4.5335
Olfr56	-2.9492	-2.713	-3.626	0.0035	1	-4.5527
Gm9670	2.2164	-2.8877	3.5633	0.0039	1	-4.5506
Gm10241	-1.3682	-1.1437	-3.5575	0.004	1	-4.5039
M6pr-ps	-1.8215	-2.6405	-3.4843	0.0045	1	-4.5443
Rpl36-ps9	-1.6016	-2.5378	-3.463	0.0047	1	-4.5355
mt-Ta	2.2788	-2.7704	3.4483	0.0048	1	-4.5484
Slc23a3	2.2047	-2.7039	3.4435	0.0049	1	-4.5547
Malat1	0.6686	8.8452	3.4189	0.0051	1	-3.442
E030037K01Rik	-2.2419	-2.7674	-3.3468	0.0058	1	-4.5563
Gm43598	2.4984	-2.4368	3.337	0.006	1	-4.554
Gm20430	2.0539	-3.0548	3.3069	0.0063	1	-4.5567
Gm11870	1.6671	-2.4879	3.2767	0.0067	1	-4.548
Gm43133	-2.1255	-3.238	-3.2663	0.0068	1	-4.5582
Gm10735	-2.572	-3.1231	-3.2657	0.0068	1	-4.5577
Il24	-2.7693	0.3567	-3.2627	0.0068	1	-4.4613
Hrk	2.07	-1.9039	3.2496	0.007	1	-4.5377
Gm15283	-1.5693	-1.279	-3.2384	0.0071	1	-4.5266
Snord17	0.9651	-1.1206	3.199	0.0077	1	-4.4896
Zcwpw2	-2.012	-2.2964	-3.194	0.0078	1	-4.5527
Gm26826	2.005	-2.1456	3.1935	0.0078	1	-4.5493
Inca1	1.0351	0.339	3.1849	0.0079	1	-4.4665
Bckdha	0.3715	5.6159	3.1807	0.0079	1	-3.7089
Adgrf5	1.9495	-2.5388	3.1608	0.0082	1	-4.5415
Gm45838	1.7902	-3.2543	3.1503	0.0084	1	-4.557
Zc3h11a	0.473	4.0219	3.1436	0.0085	1	-3.9767
Gm43533	1.7885	-2.6091	3.1277	0.0088	1	-4.5596
Gm8606	-1.6835	-1.6577	-3.0997	0.0092	1	-4.5355
Gm16098	1.6688	-2.3734	3.0881	0.0094	1	-4.5548
BC006965	1.5646	-1.7454	3.0831	0.0095	1	-4.5384
C920021L13Rik	2.2662	-2.1276	3.0506	0.0101	1	-4.5529
Gm9988	1.9902	-2.6554	3.0495	0.0101	1	-4.5497
Gm49745	1.659	-2.7772	3.0464	0.0102	1	-4.5555
Gm26935	2.1975	-2.5106	3.0355	0.0104	1	-4.5608
Tmem236	1.9221	-2.0078	3.0217	0.0107	1	-4.5427
Oaz3	-1.8806	-1.969	-3.0111	0.0109	1	-4.54

Gm42998	1.8706	-2.4237	3.0085	0.0109	1	-4.5469
Gm3839	-1.8691	-1.9429	-3.0084	0.0109	1	-4.5401
Gm7466	-1.1091	-0.6401	-2.9937	0.0112	1	-4.4958
Nmb	1.9039	-1.8624	2.9831	0.0115	1	-4.5511
Gm27219	1.3807	-1.8945	2.9816	0.0115	1	-4.5313
Gm7667	-1.8934	-2.7199	-2.9645	0.0119	1	-4.5569
Maged2	0.3311	6.4991	2.9587	0.012	1	-3.7133
Selenok-ps1	2.2087	-2.9124	2.9514	0.0122	1	-4.5621
AW047730	1.4996	-1.9973	2.9479	0.0122	1	-4.5544
Gm12925	1.6595	-2.5448	2.942	0.0124	1	-4.5626
Gm43654	1.6571	-2.0307	2.9334	0.0126	1	-4.5442
Gm37303	2.0798	-2.0826	2.9228	0.0128	1	-4.5524
Fabp4	0.9708	3.6018	2.9193	0.0129	1	-3.9535
Tpi-rs4	-1.1085	-0.0961	-2.9163	0.013	1	-4.4815
Klhl10	-1.7987	-1.4368	-2.9058	0.0132	1	-4.5465
Zfp383	-0.495	2.2458	-2.9022	0.0133	1	-4.3104
Gm11663	-0.6556	1.0569	-2.8812	0.0138	1	-4.4166
Gm7862	-0.4396	2.8171	-2.8634	0.0143	1	-4.2282
Dock9	0.3958	7.0632	2.8376	0.015	1	-3.7408
Hist1h3b	-1.9794	-2.3904	-2.8341	0.0151	1	-4.5606
Bcl2l15	1.5411	-2.4003	2.8266	0.0153	1	-4.5548
Hist1h2bp	1.8685	-2.5558	2.8207	0.0155	1	-4.5595
Gm48194	1.7773	-1.5489	2.8158	0.0156	1	-4.5481
Mir703	1.2616	2.0493	2.8058	0.0159	1	-4.3273
Tpi-rs10	-1.8238	-2.2139	-2.8004	0.0161	1	-4.546
Gm6474	-1.0931	-0.6811	-2.7879	0.0165	1	-4.5135
Gm38247	-1.3397	-2.4822	-2.7846	0.0166	1	-4.5546
Best2	1.7972	-3.0198	2.7783	0.0168	1	-4.5633
Clec2l	1.6324	-1.7058	2.7741	0.0169	1	-4.5475
Gbp10	-2.9342	-0.9697	-2.7738	0.0169	1	-4.5654
Hyal3	-1.2909	-1.794	-2.7714	0.017	1	-4.5492
Snora23	1.0859	-1.8544	2.7611	0.0173	1	-4.5304
Dhrs9	0.4478	4.3528	2.7558	0.0175	1	-3.9847
Gm42918	1.9175	-2.7985	2.752	0.0176	1	-4.5654
5830428M24Rik	1.8752	-2.1887	2.7438	0.0179	1	-4.5534
Rufy4	0.8911	0.0467	2.7395	0.018	1	-4.4348
Rps15a-ps5	-0.9877	-0.2864	-2.7394	0.018	1	-4.4908
Gm42748	1.5205	-1.314	2.735	0.0182	1	-4.5322
Tia1	0.2626	6.1419	2.7322	0.0182	1	-3.8448
Kdsr	0.2742	5.4817	2.7239	0.0185	1	-3.9009
mt-Rnr2	0.4278	11.0045	2.7202	0.0187	1	-3.7742
Ankrd7	2.0977	-2.9955	2.7189	0.0187	1	-4.5626
Dnah17	1.8792	-1.5015	2.7142	0.0189	1	-4.553
Gm47573	-1.6423	-2.4921	-2.7122	0.0189	1	-4.5557
Pdcd4	0.3697	5.5511	2.7028	0.0193	1	-3.9097
Gm44243	1.3123	-1.6702	2.6926	0.0196	1	-4.5443
Sirt6	-0.3214	3.6184	-2.691	0.0197	1	-4.1746
B130021K23Rik	1.3416	-1.8841	2.6862	0.0199	1	-4.5373
AC132253.6	0.6279	0.6422	2.6829	0.02	1	-4.444
Gm7353	1.0061	-0.3548	2.6814	0.02	1	-4.5087
Gm15787	1.2675	-0.7547	2.6808	0.0201	1	-4.5256
1700034P13Rik	-1.2579	-2.0204	-2.676	0.0202	1	-4.5453

Gm6366	-1.617	-2.8177	-2.6719	0.0204	1	-4.5567
Inka1	0.7935	0.2368	2.6705	0.0204	1	-4.4821
Ifi47	-3.2164	-0.0529	-2.6701	0.0205	1	-4.5592
2900009J06Rik	-1.431	-0.92	-2.6688	0.0205	1	-4.5362
Add2	0.6442	0.3622	2.6684	0.0205	1	-4.383
Gm9522	-1.8143	-2.108	-2.6676	0.0206	1	-4.5528
Higd1a	-0.3969	5.7687	-2.647	0.0214	1	-3.903
Gm16020	-1.0397	-1.2084	-2.6448	0.0214	1	-4.5335
Gm5652	-1.2773	-1.0414	-2.6445	0.0215	1	-4.5267
Apol8	-1.7672	-1.5089	-2.6341	0.0219	1	-4.5519
Nck2	0.2554	5.9087	2.6259	0.0222	1	-3.9006
1700029J07Rik	1.0133	-0.9573	2.623	0.0223	1	-4.528
Gm6263	1.6146	-2.1166	2.6181	0.0225	1	-4.5592
Snord15a	-1.4977	-2.6483	-2.6181	0.0225	1	-4.5665
9330104G04Rik	0.9128	0.5136	2.6177	0.0225	1	-4.4945
Exoc3l2	1.6229	-2.9454	2.6123	0.0228	1	-4.5579
Gm7859	1.5936	-2.4681	2.6117	0.0228	1	-4.5584
Gm43759	1.7267	-2.1525	2.6051	0.0231	1	-4.5584
Rps6-ps1	1.4051	-2.1496	2.605	0.0231	1	-4.5518
Gm5340	-1.7107	-2.1759	-2.6045	0.0231	1	-4.5629
Gm10169	-0.7666	0.6361	-2.6043	0.0231	1	-4.4629
Gm42783	0.9244	-0.5664	2.6023	0.0232	1	-4.5058
Mmd	0.4933	6.385	2.602	0.0232	1	-3.8771
Gm45627	-1.75	-1.8748	-2.5986	0.0233	1	-4.5515
BC023105	-2.0044	-2.9653	-2.5934	0.0236	1	-4.5688
Gm12359	-0.9197	-0.9505	-2.5909	0.0237	1	-4.5219
Cspg4	2.1759	-2.8523	2.5897	0.0237	1	-4.5587
Gm7285	1.2225	-1.4722	2.5892	0.0238	1	-4.544
Gm16288	-1.9624	-1.8557	-2.5849	0.0239	1	-4.5514
Ccdc173	-0.7543	1.4835	-2.5835	0.024	1	-4.4452
Onecut2	0.6781	0.5552	2.5786	0.0242	1	-4.4706
Gm4800	-1.1666	-1.9954	-2.5759	0.0243	1	-4.5471
AC131065.2	-1.032	-0.6595	-2.5748	0.0244	1	-4.523
Rpl19-ps6	-1.1486	-0.6102	-2.5739	0.0244	1	-4.522
Tha1	-0.672	0.9339	-2.5709	0.0246	1	-4.4525
Gm17193	-1.4922	-2.8479	-2.5703	0.0246	1	-4.5648
Gm5853	-1.3566	-1.8687	-2.567	0.0247	1	-4.5405
Rap1gap	0.5635	3.3962	2.5663	0.0248	1	-4.1148
Gm23419	-2.1468	-2.9994	-2.566	0.0248	1	-4.5686
Gm48958	1.5709	-2.6948	2.5581	0.0252	1	-4.5624
Gm18541	-1.253	-2.2278	-2.5539	0.0253	1	-4.5513
Mpeg1	-2.5853	-2.3083	-2.5472	0.0257	1	-4.5691
Gm15609	2.0729	-4.225	2.5464	0.0257	1	-4.5695
Slc25a2	1.0539	-1.9752	2.5414	0.0259	1	-4.5502
0610040B10Rik	1.1265	-0.5979	2.5398	0.026	1	-4.5331
Gm36401	0.6431	0.301	2.5296	0.0265	1	-4.4571
Gm17825	-1.783	-2.8682	-2.5279	0.0266	1	-4.5612
Gm4654	-0.8824	-0.4951	-2.5231	0.0268	1	-4.5192
AC125101.1	-1.9934	-1.8561	-2.5223	0.0269	1	-4.5598
Naaladl1	0.7784	-0.3705	2.5212	0.0269	1	-4.4912
Gm7192	-1.6382	-3.1464	-2.519	0.027	1	-4.5681
Ecsit	-0.2648	4.6852	-2.5081	0.0276	1	-4.0587

Gm26645	-1.724	-1.7022	-2.5041	0.0278	1	-4.5575
Gm8349	-1.6337	-1.5592	-2.5037	0.0278	1	-4.5543
Acot9	-0.2821	6.7312	-2.4991	0.028	1	-3.9335
Syne1	0.4108	5.7112	2.4973	0.0281	1	-3.9597
Gm9349	-2.0752	-3.1395	-2.4972	0.0281	1	-4.5704
Gdf9	-1.365	-1.8619	-2.4961	0.0282	1	-4.5583
Itgb1bp1	-0.2266	5.009	-2.4859	0.0287	1	-4.0464
Rbm42	1.1961	-1.6212	2.4857	0.0287	1	-4.5473
Gm4459	-0.89	-1.2585	-2.4852	0.0287	1	-4.5343
Synj2	0.2828	6.1101	2.4797	0.029	1	-3.95
Gm20521	-1.1403	-2.1355	-2.4793	0.0291	1	-4.5542
Pi16	2.2232	-0.7946	2.4776	0.0292	1	-4.5332
mt-Nd6	0.5155	5.3592	2.4772	0.0292	1	-4.0069
Whamm	-0.2821	3.7989	-2.475	0.0293	1	-4.2175
Gm21972	-1.7111	-2.211	-2.4705	0.0295	1	-4.5592
Zfp951	0.6663	0.7859	2.4684	0.0296	1	-4.4659
Fmnl2	0.355	7.3325	2.4683	0.0297	1	-3.9323
Rnf182	0.7939	2.3263	2.4678	0.0297	1	-4.3336
Pgam1	-0.2589	7.835	-2.466	0.0298	1	-3.9288
Mbtd1	0.3251	5.8162	2.4641	0.0299	1	-3.9913
9530027J09Rik	1.1858	-2.0034	2.461	0.0301	1	-4.5562
Gm20257	1.4365	-1.0417	2.4609	0.0301	1	-4.5421
Med27	-0.3015	3.8965	-2.4608	0.0301	1	-4.1995
Pcolce	-0.3754	8.388	-2.4603	0.0301	1	-3.9345
Gm43039	2.0448	-2.5072	2.4591	0.0302	1	-4.5642
Snord73a	0.9997	-1.8678	2.4564	0.0303	1	-4.5514
Scarna13	1.9079	-2.8208	2.451	0.0306	1	-4.5678
Prkacb	0.2108	7.2334	2.4509	0.0306	1	-3.9453
Gm4778	2.3082	-3.5066	2.4499	0.0307	1	-4.5709
Pdcd2l	-0.3094	3.7944	-2.4449	0.0309	1	-4.1934
Mettl27	0.5083	3.475	2.4439	0.031	1	-4.2747
Snord72	1.2247	-1.6315	2.4404	0.0312	1	-4.5407
Spink2	1.668	-2.7986	2.4398	0.0312	1	-4.56
Polr3gl	0.488	3.4262	2.4375	0.0314	1	-4.2666
Gm5575	-1.5968	-2.6343	-2.4366	0.0314	1	-4.5651
Gm3534	-1.2742	-1.9752	-2.4352	0.0315	1	-4.5531
Gm27365	1.4085	-2.6393	2.4335	0.0316	1	-4.5652
Tcp11l2	0.5812	4.5957	2.4328	0.0316	1	-4.1655
B930094E09Rik	-1.7774	-1.4452	-2.4257	0.0321	1	-4.5562
Pcdhgc4	0.7873	0.0749	2.4241	0.0321	1	-4.5119
Gm44710	-0.8939	-0.7428	-2.4231	0.0322	1	-4.5356
D630003M21Rik	1.4626	-0.6553	2.4222	0.0323	1	-4.5586
Gm8228	-1.011	-1.5892	-2.4183	0.0325	1	-4.5434
Gm6822	1.2753	-2.2189	2.4167	0.0326	1	-4.555
Pcmtd2	0.446	5.2158	2.4139	0.0328	1	-4.0709
AI480526	0.6866	1.3179	2.4131	0.0328	1	-4.4603
Syde1	-0.2963	4.9704	-2.4128	0.0328	1	-4.0818
Gm44081	1.4167	-2.9529	2.4047	0.0333	1	-4.5643
Gm13216	-0.7298	-0.3751	-2.4009	0.0335	1	-4.4984
Mthfd2	-0.3757	6.4301	-2.3995	0.0336	1	-3.9756
Gm37660	0.6448	0.4397	2.3991	0.0336	1	-4.5005
Gm12663	-0.5296	1.3442	-2.3977	0.0337	1	-4.4479

Ankrd61	1.4453	-1.1724	2.3972	0.0338	1	-4.5527
Firre	0.5565	4.1553	2.3939	0.034	1	-4.1877
Hist1h3f	-1.5382	-2.0617	-2.3898	0.0342	1	-4.556
Gm28043	0.7524	-0.2132	2.389	0.0343	1	-4.5094
Gm32031	0.8193	-0.8058	2.3865	0.0344	1	-4.5304
2610044O15Rik8	0.3211	3.4695	2.3831	0.0346	1	-4.27
Gm14130	-0.9005	-0.6967	-2.3821	0.0347	1	-4.5233
Zfp217	0.2421	6.0144	2.3807	0.0348	1	-4.0035
Gm14305	1.9283	-2.2595	2.3787	0.0349	1	-4.566
Asns	-0.3221	7.1447	-2.3749	0.0352	1	-3.9812
Gnptg	-0.3858	3.6849	-2.3723	0.0353	1	-4.274
Gm13410	1.4338	-1.4933	2.3709	0.0354	1	-4.5539
AC110913.1	-1.1252	-1.9053	-2.3708	0.0354	1	-4.5553
Gm43379	1.3091	-1.7526	2.3707	0.0354	1	-4.5626
Gm28536	-1.3969	-2.7008	-2.3704	0.0354	1	-4.5644
Atxn3	0.27	5.4085	2.3689	0.0355	1	-4.0573
Plp1	1.0121	-0.5514	2.3683	0.0356	1	-4.5251
Gm12355	-0.7559	-1.055	-2.3663	0.0357	1	-4.5334
Zfp335os	0.6284	1.1581	2.3639	0.0359	1	-4.4479
Synpo	0.3939	6.6357	2.3627	0.0359	1	-4.002
Gm12435	-0.7098	-0.7464	-2.3603	0.0361	1	-4.5235
Hist1h2af	-1.4613	-2.13	-2.3583	0.0362	1	-4.5597
2900089D17Rik	0.9413	-0.5352	2.3568	0.0363	1	-4.5394
Usp17le	1.3597	-2.1009	2.3565	0.0364	1	-4.5547
Lmntd2	0.9898	-0.9518	2.3565	0.0364	1	-4.5349
Ccnd2	-1.8779	-2.3706	-2.3561	0.0364	1	-4.5672
E130102H24Rik	0.9645	-0.6517	2.3519	0.0367	1	-4.5354
Plcd4	1.9448	-3.2785	2.3442	0.0372	1	-4.5731
Gm6311	-0.8826	-0.628	-2.3428	0.0373	1	-4.5277
Man1a	0.4811	6.368	2.3407	0.0374	1	-4.0247
Rasa1	0.2396	6.9931	2.3365	0.0377	1	-4.0066
A530013C23Rik	-0.8526	-0.6085	-2.3289	0.0382	1	-4.5411
Hist1h2al	1.0856	0.4132	2.326	0.0384	1	-4.5286
Gm8983	1.2286	-1.6095	2.3258	0.0384	1	-4.5523
Junos	0.4811	1.5744	2.3232	0.0386	1	-4.3818
Gm7803	1.6108	-2.0136	2.3189	0.0389	1	-4.5575
Nfat5	0.3599	7.765	2.3166	0.0391	1	-4.008
Grhpr	-0.387	4.5991	-2.3158	0.0391	1	-4.1502
mt-Nd5	0.3034	10.2284	2.314	0.0393	1	-3.9965
Gm5835	-0.529	2.0352	-2.3123	0.0394	1	-4.3979
Icam5	0.8365	1.2077	2.309	0.0396	1	-4.4742
AI463229	1.0095	-1.5548	2.3026	0.0401	1	-4.5426
Adm2	-1.3563	-1.9636	-2.3011	0.0402	1	-4.5526
Creb3l2	0.3299	6.846	2.2961	0.0406	1	-4.0286
Gm47694	0.787	-0.2086	2.296	0.0406	1	-4.5186
Slc2a9	0.8223	0.5795	2.2945	0.0407	1	-4.4923
2510017J16Rik	1.6285	-2.4952	2.2882	0.0411	1	-4.5656
Gm45833	-1.0264	-1.7128	-2.2876	0.0412	1	-4.5467
Gm26947	-0.7097	-0.7426	-2.2865	0.0413	1	-4.5136
Nanp	0.8328	-0.9818	2.2862	0.0413	1	-4.5409
Abca1	1.1397	3.7681	2.2836	0.0415	1	-4.3606
Gm9899	1.48	-3.5093	2.2832	0.0415	1	-4.5714

Gm21817	1.6629	-1.9281	2.2823	0.0416	1	-4.5597
Btn2a2	1.3925	-2.3988	2.2807	0.0417	1	-4.5661
Rps2-ps11	1.1966	-2.1052	2.2803	0.0417	1	-4.5608
Gm16159	1.6267	-2.8451	2.2797	0.0418	1	-4.5683
1810059H22Rik	0.7354	0.2436	2.2767	0.042	1	-4.5102
Gm20337	-0.7883	-0.8389	-2.2738	0.0422	1	-4.5405
Wdr60	0.254	5.4793	2.2734	0.0423	1	-4.0909
Npr3	-1.6735	-1.3882	-2.2719	0.0424	1	-4.5593
Tmem165	0.2162	6.6669	2.2705	0.0425	1	-4.0489
Sash1	0.4392	5.2683	2.2692	0.0426	1	-4.1158
Dennd2d	1.6352	-2.8431	2.2681	0.0427	1	-4.5715
Npl	1.2927	-0.8746	2.2579	0.0435	1	-4.5464
Mrps26	-0.2399	5.5676	-2.2577	0.0435	1	-4.0925
Gm26384	1.0181	-0.5304	2.2574	0.0435	1	-4.5244
Gpr135	0.6467	1.2032	2.2547	0.0437	1	-4.4827
Gm5487	0.9077	-0.4791	2.2518	0.0439	1	-4.5256
Gm13886	1.6413	-2.4205	2.2517	0.0439	1	-4.5652
Gm29346	-1.3706	-3.1766	-2.2509	0.044	1	-4.5757
Cul9	1.6297	-2.6204	2.2478	0.0442	1	-4.573
Asprv1	1.67	-3.2753	2.2468	0.0443	1	-4.5743
Gm17655	1.6102	-2.2016	2.2462	0.0444	1	-4.5682
Gm43071	1.4017	-1.2562	2.2456	0.0444	1	-4.5546
Ccnd3	-0.2452	6.5603	-2.2441	0.0446	1	-4.061
Gabbr1	0.4819	3.8886	2.2439	0.0446	1	-4.2805
Gm15998	0.9498	-0.014	2.2433	0.0446	1	-4.5256
Asb5	1.6927	-2.8269	2.2421	0.0447	1	-4.5744
Gm47863	-1.5748	-1.5991	-2.232	0.0455	1	-4.5635
Nfatc2	1.0804	0.1232	2.23	0.0457	1	-4.5227
Gch1	0.5533	3.4584	2.2296	0.0457	1	-4.2454
Tpi1	-0.3035	9.8634	-2.2266	0.046	1	-4.0439
Hist2h3c2	1.8265	-0.8229	2.2258	0.046	1	-4.5629
Gm38414	1.5783	-1.841	2.2257	0.046	1	-4.5588
1110003F10Rik	-1.3974	-2.7734	-2.2246	0.0461	1	-4.5675
Thnsl2	1.935	-2.6458	2.2238	0.0462	1	-4.5746
Gm9701	1.8318	-3.3158	2.2212	0.0464	1	-4.5734
Gm12415	1.7735	-2.969	2.2181	0.0467	1	-4.5715
Gm6187	-1.3491	-2.6726	-2.2168	0.0468	1	-4.5645
Foxd2	-1.332	-2.1483	-2.2141	0.047	1	-4.5655
Gm12070	-1.3891	-2.5013	-2.2136	0.0471	1	-4.5655
Gm37121	1.463	-1.3703	2.2125	0.0471	1	-4.5589
Gm10382	0.76	-0.888	2.2116	0.0472	1	-4.5441
Gm26695	1.6878	-2.7837	2.2079	0.0475	1	-4.5718
Cox5a	-0.2035	7.3848	-2.2075	0.0476	1	-4.071
Gm26890	1.0662	-1.341	2.2074	0.0476	1	-4.5556
Gm7079	-0.7449	-1.4448	-2.2062	0.0477	1	-4.5421
Fcnaos	-1.3356	-3.0111	-2.2018	0.0481	1	-4.5756
Gm10705	-0.5426	0.9179	-2.2017	0.0481	1	-4.4861
Gm11572	-1.4614	-2.6102	-2.201	0.0481	1	-4.5691
Gm26514	-1.3394	-2.4421	-2.1998	0.0482	1	-4.5641
6330415G19Rik	0.9911	-1.5792	2.1993	0.0483	1	-4.5571
Dnah10	1.2903	-2.7562	2.1986	0.0483	1	-4.5723
Gm17110	1.2376	-1.7443	2.1964	0.0485	1	-4.5617

Gm44899	1.597	-2.173	2.1955	0.0486	1	-4.5707
Khdc4	0.2748	6.051	2.1952	0.0486	1	-4.099
Gm26202	1.6623	-2.5356	2.1938	0.0488	1	-4.5639
Gm8141	-0.9966	-0.8337	-2.1936	0.0488	1	-4.5386
Pcdhb8	-1.736	-3.2925	-2.1928	0.0488	1	-4.576
Fzr1	-0.235	5.5728	-2.1927	0.0489	1	-4.1194
Grin1	1.9794	-3.3608	2.1925	0.0489	1	-4.5751
Gm43088	1.2791	-2.7492	2.1924	0.0489	1	-4.5672
Asap2	0.3766	5.8115	2.192	0.0489	1	-4.1093
Apoh	2.1395	-3.2824	2.192	0.0489	1	-4.5751
Psph	-0.3743	5.122	-2.1898	0.0491	1	-4.1349
Gm43597	0.45	2.1664	2.188	0.0493	1	-4.4139
Gm48455	-0.5808	-0.163	-2.1871	0.0493	1	-4.5037
Gm4864	-0.4697	1.7989	-2.1852	0.0495	1	-4.4353
Yipf2	0.3134	4.2163	2.1847	0.0496	1	-4.2661
Casq2	1.6202	-2.0199	2.1843	0.0496	1	-4.5724
Pygm	0.8847	-0.4048	2.1824	0.0498	1	-4.5317

Supplementary Table 2. List of the most significant differentially expressed genes in PD173074 versus DMSO treated tumourspheres

GeneID	logFC	AveExpr	t	P.Value	adj.P.Val	B
Gdpd2	3.2595	4.6212	9.7991	0	0.0051	6.3816
Etv5	-1.0635	4.5905	-9.5702	0	0.0051	6.5822
Gas1	0.8539	6.8662	7.9101	0	0.0248	4.6593
C3	2.2648	8.8358	6.9208	0	0.062	3.3244
Etv4	-1.0637	6.0795	-6.7846	0	0.062	3.1393
Fas	2.2955	4.0401	6.659	0	0.062	2.9589
Foxc1	1.3984	6.2336	6.572	0	0.062	2.856
Rin2	0.8284	6.9121	6.4317	0	0.062	2.6324
Dlk2	1.5355	3.1153	6.3897	0	0.062	2.559
Dusp6	-1.0634	5.9731	-6.373	0	0.062	2.5413
Cp	1.5123	10.2055	6.2294	0	0.0698	2.3059
Steap4	3.157	3.4761	6.0873	0.0001	0.0792	2.0987
P2rx5	2.6746	0.9362	5.9027	0.0001	0.0813	0.9599
Gm13216	-2.5156	-0.3751	-5.8901	0.0001	0.0813	0.4904
Pck2	-0.7583	5.7077	-5.8685	0.0001	0.0813	1.8028
Ptprn	-1.2432	6.8171	-5.8579	0.0001	0.0813	1.7564
Pik3r1	1.1976	6.9304	5.8434	0.0001	0.0813	1.7381
Ipo13	0.8103	6.01	5.803	0.0001	0.0813	1.7005
Cfh	4.4954	1.8006	5.7568	0.0001	0.0813	0.7888
Ubald2	-0.6213	5.1815	-5.705	0.0001	0.0813	1.5706
Irf8	-1.1473	3.9514	-5.6819	0.0001	0.0813	1.5978
Pcolce	0.8587	8.388	5.6764	0.0001	0.0813	1.455
Phactr1	2.4131	3.3555	5.6508	0.0001	0.0813	1.5273
Add2	-1.9929	0.3622	-5.5939	0.0001	0.0815	0.8801
Dock9	-0.7891	7.0632	-5.593	0.0001	0.0815	1.334
Psd3	1.336	3.6009	5.5716	0.0001	0.0815	1.4578
Parp9	1.644	4.5789	5.54	0.0001	0.0815	1.3895
Enah	0.6763	6.6578	5.5247	0.0001	0.0815	1.2324
Irgm1	1.2989	4.0601	5.4885	0.0001	0.0834	1.3206
Susd6	0.9028	7.0456	5.4224	0.0002	0.0882	1.0616
Rgma	1.1274	4.326	5.4125	0.0002	0.0882	1.1987
Bcl9l	0.7896	6.7939	5.3739	0.0002	0.0902	0.9887
Ptgs1	-0.7295	8.1672	-5.3042	0.0002	0.0902	0.8526
Wwtr1	-0.4883	8.1451	-5.2828	0.0002	0.0902	0.8188
Col6a1	1.1028	8.0906	5.2804	0.0002	0.0902	0.8162
Gm7079	-2.2925	-1.4448	-5.2713	0.0002	0.0902	-0.4435
Bmf	3.433	3.393	5.2241	0.0002	0.0902	0.8
Cbr2	1.817	5.0056	5.2059	0.0002	0.0902	0.8499
Plekha4	3.4665	-1.7184	5.2002	0.0002	0.0902	-0.9429
Msrb3	1.3793	3.8746	5.1645	0.0002	0.0902	0.833
Pld3	0.9597	5.1223	5.1492	0.0002	0.0902	0.722
E130308A19Rik	0.8659	3.8838	5.137	0.0002	0.0902	0.7763
Pimreg	-0.4385	5.668	-5.106	0.0003	0.0902	0.556
Akap13	0.5537	5.8748	5.0872	0.0003	0.0902	0.5473
Mnd1	-0.6308	4.064	-5.0856	0.0003	0.0902	0.6411
Irf1	1.054	6.3444	5.0843	0.0003	0.0902	0.524
Agt	2.9741	2.6383	5.0808	0.0003	0.0902	0.6047
Parp14	2.0594	3.919	5.0772	0.0003	0.0902	0.7004
Tent5a	2.0997	2.6235	5.0767	0.0003	0.0902	0.6544

Il6st	0.6352	8.4579	5.0739	0.0003	0.0902	0.4693
Gm49774	-1.6059	2.8266	-5.0713	0.0003	0.0902	0.6953
D630003M21Rik	3.1144	-0.6553	5.0657	0.0003	0.0902	-0.7398
Clcn4	0.6366	5.1776	5.0609	0.0003	0.0902	0.5491
Fat1	0.6538	6.2489	5.0572	0.0003	0.0902	0.4822
Ndst3	-1.2667	1.6811	-5.0487	0.0003	0.0902	0.649
Wls	1.0177	7.953	5.001	0.0003	0.0947	0.3531
Arsj	0.9499	1.9735	4.9978	0.0003	0.0947	0.5632
Ints4	0.5214	5.8241	4.9772	0.0003	0.0964	0.3661
Hpcal1	-0.6592	5.7405	-4.9588	0.0003	0.0964	0.3278
Phldb2	2.364	4.5744	4.9567	0.0003	0.0964	0.4957
Tlr2	1.6363	4.3431	4.9161	0.0004	0.1012	0.424
Rasa4	-1.1272	3.8026	-4.9082	0.0004	0.1012	0.4034
Trp53inp2	1.645	5.5606	4.8886	0.0004	0.1017	0.2862
Ar	1.2901	4.8674	4.8841	0.0004	0.1017	0.3094
Samhd1	0.389	6.7123	4.8757	0.0004	0.1017	0.1547
Kcnn4	-1.029	5.8699	-4.8681	0.0004	0.1017	0.1479
Fut8	-0.6333	6.2033	-4.8589	0.0004	0.1018	0.139
Rbms3	1.48	1.4671	4.8454	0.0004	0.102	0.2259
Lrrk2	-1.1641	6.5076	-4.84	0.0004	0.102	0.1005
Slc22a15	-0.7913	3.8192	-4.8282	0.0004	0.1026	0.2618
Gm26699	-1.8351	-1.6471	-4.815	0.0004	0.1029	-0.6722
Pik3r3	1.2813	3.88	4.8055	0.0004	0.1029	0.261
Gm15867	1.665	-0.4896	4.8017	0.0004	0.1029	-0.39
Herc6	0.8824	3.331	4.781	0.0005	0.1052	0.2311
Magi3	0.7559	5.4684	4.757	0.0005	0.1081	0.0152
Mtor	0.5212	6.1852	4.7316	0.0005	0.1114	-0.0704
Gadd45b	1.198	3.8647	4.7235	0.0005	0.1115	0.1202
Adgrl2	0.6435	7.1735	4.7104	0.0005	0.1125	-0.1329
Rab30	0.8043	2.9193	4.6928	0.0005	0.114	0.0995
AI429214	1.6647	1.3067	4.6881	0.0005	0.114	-0.0461
Ssbp3	0.4667	6.1411	4.6552	0.0006	0.1177	-0.2005
Ch25h	3.6441	-3.1558	4.6549	0.0006	0.1177	-1.617
Lamb2	0.9708	6.2319	4.6127	0.0006	0.1225	-0.2573
Dtx3l	1.6874	4.3311	4.6038	0.0006	0.1225	-0.0956
Rasgef1a	2.1581	1.0911	4.5959	0.0006	0.1225	-0.2608
Sbno2	0.6459	6.0903	4.5878	0.0006	0.1225	-0.3217
Hmga2	-0.6143	7.7505	-4.571	0.0006	0.1225	-0.3913
Fxyd5	-1.2499	4.2208	-4.5566	0.0007	0.1225	-0.2533
Bnc2	1.6337	0.3015	4.5547	0.0007	0.1225	-0.4192
Arhgap28	0.6349	5.598	4.5499	0.0007	0.1225	-0.3507
5330438D12Rik	1.7769	-0.4569	4.5468	0.0007	0.1225	-0.7723
Mapk8ip1	0.7513	4.0029	4.5437	0.0007	0.1225	-0.1968
Gbp2	2.4629	5.4695	4.5373	0.0007	0.1225	-0.3096
Gm26722	1.6847	0.3675	4.5353	0.0007	0.1225	-0.4468
Rap1gap	-1.2484	3.3962	-4.5193	0.0007	0.1225	-0.1976
H2-T23	1.7686	2.5916	4.5175	0.0007	0.1225	-0.1791
Spry2	-0.6543	4.6193	-4.5134	0.0007	0.1225	-0.3758
Sphk1	2.6501	4.0811	4.5108	0.0007	0.1225	-0.2008
Gnao1	0.7245	6.5588	4.51	0.0007	0.1225	-0.4657
P2rx4	0.6958	4.8011	4.5072	0.0007	0.1225	-0.3409
Cdon	1.2896	4.3229	4.5007	0.0007	0.1225	-0.2737

Clic1	-0.6645	8.321	-4.5007	0.0007	0.1225	-0.5153
Spns2	1.5992	3.2667	4.5	0.0007	0.1225	-0.2079
Itm2b	0.887	8.5069	4.4759	0.0008	0.1254	-0.5568
Lipa	0.7975	6.3276	4.4736	0.0008	0.1254	-0.5153
Hcfc1r1	0.9748	5.1826	4.4699	0.0008	0.1254	-0.4336
Gm2a	0.9004	5.2242	4.4549	0.0008	0.1258	-0.4596
Tram2	0.578	4.836	4.4525	0.0008	0.1258	-0.4489
mt-Nd5	0.583	10.2284	4.4494	0.0008	0.1258	-0.6152
Parp12	0.921	4.7793	4.4469	0.0008	0.1258	-0.454
Naaladl2	3.4808	-0.3406	4.3907	0.0009	0.1349	-1.2051
Pcdhga6	1.3037	0.4236	4.39	0.0009	0.1349	-0.5752
Htra1	0.7929	8.2013	4.3886	0.0009	0.1349	-0.709
Glp1r	-2.0815	3.7701	-4.3801	0.0009	0.1349	-0.4847
Pi4k2b	-0.5844	6.5375	-4.3775	0.0009	0.1349	-0.7075
Slc2a6	1.294	4.2593	4.3721	0.0009	0.1349	-0.4767
Parvb	-0.6573	6.4683	-4.3698	0.0009	0.1349	-0.7235
Lamp2	0.612	8.7815	4.3667	0.0009	0.1349	-0.7531
Col6a2	1.5745	5.325	4.3475	0.001	0.1383	-0.6713
Tapbp	1.0429	7.8517	4.3342	0.001	0.1404	-0.7998
A330074K22Rik	1.9279	2.3486	4.3202	0.001	0.1425	-0.5051
Wnt4	2.9293	0.5694	4.3142	0.001	0.1425	-0.8053
Cxcr3	-2.9914	-1.3106	-4.3097	0.001	0.1425	-1.2566
Msh5	-1.4304	1.0265	-4.3074	0.001	0.1425	-0.5701
Sync	2.3846	1.8062	4.2897	0.0011	0.145	-0.6194
Ddx58	0.6845	5.4203	4.2882	0.0011	0.145	-0.783
Akr1c14	2.9888	1.2554	4.2768	0.0011	0.1458	-0.7599
Arhgap18	-0.8677	5.6507	-4.2761	0.0011	0.1458	-0.8531
Lactb	0.8447	4.0295	4.2626	0.0011	0.1473	-0.6825
Gm14513	-2.0627	-1.0499	-4.2607	0.0011	0.1473	-1.2211
4930471E19Rik	-0.9704	2.3079	-4.2573	0.0011	0.1473	-0.6033
Gm16685	4.9898	-1.6466	4.242	0.0012	0.1502	-2.0236
Igfbp7	1.7542	5.2706	4.2257	0.0012	0.1503	-0.85
Asns	-0.5741	7.1447	-4.2231	0.0012	0.1503	-0.9956
Snx33	0.6654	4.8934	4.2197	0.0012	0.1503	-0.8462
Vasn	0.5041	6.9064	4.2188	0.0012	0.1503	-0.9925
Egr1	2.0225	4.4426	4.216	0.0012	0.1503	-0.8555
Dusp1	1.2089	4.9433	4.2144	0.0012	0.1503	-0.855
Abcd4	-0.6616	4.5234	-4.2124	0.0012	0.1503	-0.8569
Gpsm1	0.5944	4.8225	4.2069	0.0012	0.1507	-0.867
Eif4ebp1	-0.4345	7.2154	-4.1962	0.0013	0.1508	-1.0427
Itgb8	2.4977	2.5447	4.1962	0.0013	0.1508	-0.6938
Cnn2	0.8097	7.2669	4.1947	0.0013	0.1508	-1.04
Samd9l	1.9721	1.5756	4.1782	0.0013	0.1533	-0.7709
Trim21	1.0711	2.1066	4.1721	0.0013	0.1533	-0.7238
Ube2t	-0.5504	4.0755	-4.168	0.0013	0.1533	-0.9119
Atxn1	0.8135	4.3356	4.1654	0.0013	0.1533	-0.8847
Klf3	0.7046	6.5166	4.1635	0.0013	0.1533	-1.0737
Gprc5c	2.8662	-2.3963	4.1621	0.0013	0.1533	-1.9591
Cars	-0.5102	6.5935	-4.1546	0.0013	0.154	-1.1076
Tgfb2	1.9399	3.007	4.1485	0.0014	0.154	-0.7664
Gm27021	2.3131	-3.2753	4.1482	0.0014	0.154	-1.9001
Mxd4	0.855	6.7497	4.1332	0.0014	0.1571	-1.1267

Bmpr2	0.4178	7.1043	4.1206	0.0014	0.1571	-1.1715
Gm43737	0.7865	2.5434	4.1177	0.0014	0.1571	-0.8157
Inava	0.9291	2.6254	4.1143	0.0014	0.1571	-0.8345
Hmces	-0.4834	4.5123	-4.1122	0.0015	0.1571	-1.0584
Gm17181	-2.6501	-2.6716	-4.1111	0.0015	0.1571	-1.994
E130201H02Rik	-2.1667	-2.3186	-4.1071	0.0015	0.1571	-1.7646
Gm14853	-2.372	-1.9682	-4.1036	0.0015	0.1571	-1.6414
Arel1	0.4465	5.743	4.1026	0.0015	0.1571	-1.1438
Auts2	0.7496	4.9448	4.1015	0.0015	0.1571	-1.0652
Irf2bpl	0.6471	6.7621	4.0929	0.0015	0.1577	-1.2118
Junb	0.8087	7.5767	4.0895	0.0015	0.1577	-1.2355
Tor4a	-0.675	5.6772	-4.0889	0.0015	0.1577	-1.1805
Syne3	-1.0194	4.5567	-4.0807	0.0015	0.1582	-1.1375
Ids	0.5951	5.2409	4.0805	0.0015	0.1582	-1.1435
Clec2d	1.3265	2.2486	4.0674	0.0016	0.161	-0.8917
Srgap1	1.9484	2.4311	4.0625	0.0016	0.1612	-0.901
Lipg	-0.915	4.511	-4.0599	0.0016	0.1612	-1.14
Ier3	-0.7314	8.5462	-4.0492	0.0016	0.1629	-1.323
Sulf2	1.3338	6.4946	4.0476	0.0016	0.1629	-1.2682
Vps8	0.7851	3.9875	4.0399	0.0017	0.1642	-1.07
App	0.5987	8.7532	4.0319	0.0017	0.1655	-1.3503
Eif2ak2	0.4384	5.4859	4.0262	0.0017	0.1655	-1.2746
Amy1	2.2565	-1.8865	4.0258	0.0017	0.1655	-1.9525
Pam	-0.3774	7.6453	-4.0197	0.0017	0.1655	-1.3624
Zfp672	0.5556	3.5689	4.0158	0.0017	0.1655	-1.0661
Nmi	1.1517	3.1068	4.0128	0.0017	0.1655	-1.0174
Vwa5a	1.3923	5.3721	4.0119	0.0017	0.1655	-1.2278
Sh3bgrl3	-0.4115	5.87	-4.0079	0.0017	0.1655	-1.3343
Slc25a48	1.6644	4.727	4.0074	0.0018	0.1655	-1.1814
Nrarp	-2.7376	1.9943	-4.001	0.0018	0.1664	-1.0019
Padi4	-1.0119	5.7746	-3.9867	0.0018	0.1684	-1.3733
Capg	-0.6231	7.6839	-3.9843	0.0018	0.1684	-1.4251
Efl1	-0.4847	5.5989	-3.9797	0.0018	0.1684	-1.3761
Me3	3.5646	-0.6104	3.9742	0.0019	0.1684	-1.7211
Dusp4	-1.1801	5.9321	-3.9718	0.0019	0.1684	-1.415
Aig1	-0.5964	4.3027	-3.9675	0.0019	0.1684	-1.2715
Dysf	1.325	3.0913	3.9659	0.0019	0.1684	-1.0962
Mme	4.3549	3.5312	3.9652	0.0019	0.1684	-1.0598
Met	0.69	12.1627	3.9631	0.0019	0.1684	-1.4888
Tax1bp3	0.7968	5.1229	3.9574	0.0019	0.1684	-1.331
mt-Cytb	0.3758	11.3058	3.9545	0.0019	0.1684	-1.5019
Dtx4	1.306	6.8608	3.9533	0.0019	0.1684	-1.4502
Zc2hc1a	0.7548	4.8315	3.953	0.0019	0.1684	-1.3087
Rabepk	-0.4504	4.7904	-3.9473	0.002	0.1684	-1.3664
Grhl2	-1.5203	1.3978	-3.9463	0.002	0.1684	-1.0863
Axin2	1.6526	1.9253	3.9446	0.002	0.1684	-1.0891
Skap2	0.7898	5.1792	3.9398	0.002	0.1684	-1.3823
Ubtd2	0.5913	4.2097	3.9388	0.002	0.1684	-1.2912
Lxn	0.8386	6.1775	3.935	0.002	0.1684	-1.464
Creb3l2	-0.572	6.846	-3.9347	0.002	0.1684	-1.4989
Slco4a1	-1.1541	5.6971	-3.9341	0.002	0.1684	-1.4711
Acap3	0.3596	5.2641	3.9246	0.002	0.1705	-1.4515

Ltbp3	-0.5711	8.0843	-3.9169	0.0021	0.1721	-1.5526
Tapbpl	1.121	3.4732	3.9114	0.0021	0.1723	-1.2206
Glul	0.8264	7.9823	3.909	0.0021	0.1723	-1.5624
Sarm1	1.9052	-0.4716	3.9012	0.0021	0.1723	-1.535
Gm12925	2.1885	-2.5448	3.9006	0.0021	0.1723	-2.1656
Kansl1l	0.6192	4.4142	3.8995	0.0021	0.1723	-1.3553
Cst3	0.7868	8.1938	3.8986	0.0021	0.1723	-1.5837
Gm6257	-1.8448	-2.3298	-3.8944	0.0021	0.1723	-1.8851
AC149090.1	0.4883	6.1871	3.8909	0.0022	0.1723	-1.5482
B4galt7	-0.4445	5.0388	-3.8901	0.0022	0.1723	-1.4737
Gm12482	-1.7509	-1.114	-3.8899	0.0022	0.1723	-1.5615
Prune2	0.9945	6.9121	3.886	0.0022	0.1724	-1.5836
Emp1	-0.5728	8.6562	-3.8819	0.0022	0.1724	-1.6244
Fndc7	1.4547	1.2945	3.881	0.0022	0.1724	-1.2076
Dclk1	1.6374	7.4176	3.8782	0.0022	0.1724	-1.6055
Gm43533	2.2094	-2.6091	3.8767	0.0022	0.1724	-2.1859
Etv1	-0.6813	5.77	-3.8682	0.0023	0.1731	-1.6058
Tpp1	0.5417	6.9115	3.8652	0.0023	0.1731	-1.623
St5	1.0699	5.5931	3.8651	0.0023	0.1731	-1.5336
Pigz	2.5998	-2.4265	3.8606	0.0023	0.1731	-2.2025
mt-Nd4	0.3978	9.65	3.8597	0.0023	0.1731	-1.6678
Hspa1l	-2.0486	-3.3399	-3.856	0.0023	0.1731	-2.2061
Gm28875	-1.4958	1.0799	-3.8555	0.0023	0.1731	-1.2327
Timp2	0.9198	9.7267	3.8548	0.0023	0.1731	-1.6764
Zbtb20	0.9368	5.4806	3.8506	0.0023	0.1737	-1.5549
Rnd3	1.0324	4.6184	3.8462	0.0023	0.1737	-1.4957
Nfkbia	0.5598	8.0493	3.8459	0.0023	0.1737	-1.6803
Marcks	0.9103	6.3218	3.8382	0.0024	0.1754	-1.6505
Gm13398	2.5716	-2.8831	3.8349	0.0024	0.1754	-2.2333
Eya2	-2.1539	1.8039	-3.8335	0.0024	0.1754	-1.2836
Tnfaip3	1.2113	4.8464	3.8292	0.0024	0.176	-1.5159
Lima1	0.5599	6.8516	3.8267	0.0024	0.176	-1.6941
Rc3h1	0.3852	6.3139	3.818	0.0025	0.1781	-1.6967
4930546K05Rik	-2.1261	-1.7644	-3.8126	0.0025	0.1783	-1.8121
Ubxn2b	0.634	3.3669	3.8114	0.0025	0.1783	-1.4136
Ramp3	-1.3631	3.9284	-3.8088	0.0025	0.1783	-1.4946
Pkn3	-0.7832	3.1648	-3.8055	0.0025	0.1783	-1.4453
Wdr81	0.5233	5.612	3.8043	0.0025	0.1783	-1.6534
Rundc3a	-0.8976	4.1861	-3.8036	0.0025	0.1783	-1.4912
Bcl3	0.7519	4.9419	3.791	0.0026	0.1817	-1.6438
Rsu1	0.5075	5.948	3.7804	0.0026	0.1817	-1.737
Synj2	-0.4393	6.1101	-3.7761	0.0027	0.1817	-1.7631
Erbin	0.5261	8.0805	3.7761	0.0027	0.1817	-1.8058
D830050J10Rik	1.4953	-0.6532	3.7754	0.0027	0.1817	-1.6157
Acadm	0.5739	6.4241	3.7753	0.0027	0.1817	-1.7662
AW047730	1.9108	-1.9973	3.7751	0.0027	0.1817	-2.078
Gns	0.3878	6.9383	3.7721	0.0027	0.1817	-1.7937
Krt18	-1.0604	3.6476	-3.7703	0.0027	0.1817	-1.4969
Mfsd11	0.4862	5.3258	3.7697	0.0027	0.1817	-1.7041
Hsd17b10	-0.4387	6.7338	-3.7689	0.0027	0.1817	-1.8002
Nuak2	1.0972	3.0949	3.7644	0.0027	0.1824	-1.442
Aplp2	0.4932	9.2321	3.7624	0.0027	0.1824	-1.8419

Col27a1	1.3542	-0.1521	3.7525	0.0028	0.1841	-1.5495
Cks1b	-0.3787	7.4558	-3.7508	0.0028	0.1841	-1.8511
Scrn1	1.2868	4.9222	3.7493	0.0028	0.1841	-1.66
Gm12976	-2.0047	-2.0617	-3.7475	0.0028	0.1841	-1.9678
Tinagl1	-0.9819	7.5961	-3.7469	0.0028	0.1841	-1.8556
Gba	0.4969	6.2332	3.7423	0.0028	0.1843	-1.8216
Nckap5	3.9053	-0.6001	3.7395	0.0028	0.1843	-2.1404
Gm12359	-1.4397	-0.9505	-3.7368	0.0029	0.1843	-1.686
Rps23-ps2	-1.0297	0.8265	-3.7366	0.0029	0.1843	-1.436
Ccnd1	-0.8439	8.4099	-3.7358	0.0029	0.1843	-1.8934
Trim12c	1.1689	3.2189	3.7267	0.0029	0.1867	-1.5146
Ctsa	0.5302	8.0657	3.7198	0.0029	0.1883	-1.9066
Shf	-0.7525	4.124	-3.7117	0.003	0.1904	-1.6702
Xaf1	2.1585	3.0956	3.7095	0.003	0.1905	-1.4972
Hnrnpa1l2-ps2	-1.846	-0.9446	-3.707	0.003	0.1906	-1.8055
Grn	0.5602	9.191	3.7052	0.003	0.1906	-1.9448
Lgals9	2.0597	3.6368	3.7015	0.003	0.1912	-1.5704
Lurap1l	0.7651	4.4287	3.6888	0.0031	0.1937	-1.7199
Map1lc3a	0.634	5.6305	3.688	0.0031	0.1937	-1.8703
Gm43379	2.0192	-1.7526	3.6864	0.0031	0.1937	-2.0616
Rgs16	-1.3578	5.9679	-3.6863	0.0031	0.1937	-1.9183
Ano1	-0.7186	6.5637	-3.6786	0.0032	0.1958	-1.9611
Trim54	-0.8424	3.2559	-3.6724	0.0032	0.1973	-1.6329
Uba7	3.0113	0.6125	3.6577	0.0033	0.2016	-1.6949
Cav1	0.8073	9.4534	3.6568	0.0033	0.2016	-2.0369
Gm5946	-1.9786	-2.602	-3.6467	0.0034	0.2036	-2.205
Haus1	-0.3931	5.0381	-3.6455	0.0034	0.2036	-1.9484
Lims1	0.6042	6.2844	3.6454	0.0034	0.2036	-2.0037
Gabarap	0.4312	7.6033	3.64	0.0034	0.2043	-2.0462
Dnah8	-1.5255	-1.2439	-3.6397	0.0034	0.2043	-1.8256
Mrgprf	1.3592	3.6601	3.6331	0.0035	0.205	-1.7049
Relb	0.4932	5.2926	3.6302	0.0035	0.205	-1.9498
Fer1l6	-1.5097	0.8558	-3.6288	0.0035	0.205	-1.5961
Ephb6	2.1675	3.3111	3.6267	0.0035	0.205	-1.6248
Slc15a2	0.8794	3.6048	3.6261	0.0035	0.205	-1.724
Nub1	0.4303	6.9367	3.6259	0.0035	0.205	-2.0612
Spry4	-1.1129	2.251	-3.6248	0.0035	0.205	-1.7473
Prkg2	-0.9878	5.5084	-3.6194	0.0035	0.2062	-2.0472
Rab32	1.6217	4.2649	3.6179	0.0035	0.2062	-1.7963
Plscr2	0.9136	3.644	3.6098	0.0036	0.2067	-1.7757
Tgfbrap1	0.4251	5.2742	3.6084	0.0036	0.2067	-1.9986
Itga3	-0.6857	8.6778	-3.6059	0.0036	0.2067	-2.1239
Aldoart2	-2.3398	-2.074	-3.6022	0.0037	0.2067	-2.249
Fam78b	-0.8667	2.5344	-3.5978	0.0037	0.2067	-1.7568
Gm9260	-1.2437	3.4169	-3.5976	0.0037	0.2067	-1.8298
Gm14279	-1.7414	-1.1548	-3.5913	0.0037	0.2067	-1.9824
Ext1	0.6536	6.6868	3.591	0.0037	0.2067	-2.1237
Il15	1.5937	0.1556	3.5902	0.0037	0.2067	-1.7539
Lbp	2.73	4.5436	3.5873	0.0038	0.2067	-1.8206
Gm47034	2.6526	-2.1659	3.5872	0.0038	0.2067	-2.3286
Pltp	1.5612	2.5808	3.5839	0.0038	0.2067	-1.6851
Irs1	1.3791	3.0471	3.5817	0.0038	0.2067	-1.7132

Gm15730	-1.8469	-1.4339	-3.5798	0.0038	0.2067	-2.0317
Nuak1	0.4595	4.5078	3.5796	0.0038	0.2067	-1.9364
Fam53b	0.8277	4.4908	3.5794	0.0038	0.2067	-1.922
Dtnbp1	0.5338	6.1674	3.5782	0.0038	0.2067	-2.1238
Pqlc2	0.6494	2.2766	3.5767	0.0038	0.2067	-1.7084
Add3	0.7779	5.394	3.5758	0.0038	0.2067	-2.0581
Rps24-ps2	-2.3713	-2.1052	-3.5739	0.0038	0.2067	-2.3653
Tle3	0.3042	6.3534	3.5736	0.0038	0.2067	-2.1422
Card19	-0.6278	5.3025	-3.5735	0.0038	0.2067	-2.0754
Gm5873	-1.4543	-1.5302	-3.5726	0.0039	0.2067	-1.9881
Neu1	0.5904	5.0887	3.5725	0.0039	0.2067	-2.0175
Faim2	2.7054	-2.2087	3.5709	0.0039	0.2067	-2.377
2310022B05Rik	0.9114	7.2406	3.5706	0.0039	0.2067	-2.168
Rps6ka1	-0.3749	6.5337	-3.5619	0.0039	0.2092	-2.1771
Nrip1	-0.7209	6.7384	-3.5599	0.0039	0.2092	-2.1809
Gm6745	-1.8835	-1.4933	-3.5588	0.004	0.2092	-2.1078
2610008E11Rik	0.5623	3.4354	3.5488	0.004	0.2123	-1.8541
Csgalnact1	-1.2067	4.2725	-3.5475	0.004	0.2123	-2.0118
Gm29292	3.3533	-1.5007	3.5453	0.0041	0.2126	-2.3072
Mob3b	1.1873	3.1914	3.5407	0.0041	0.2137	-1.8442
Ssbp2	0.6404	4.703	3.5387	0.0041	0.2138	-2.047
Gm45833	-1.7867	-1.7128	-3.5296	0.0042	0.2161	-2.091
Ehd4	-0.4653	6.4746	-3.5295	0.0042	0.2161	-2.232
Tcn2	1.9672	4.7617	3.5264	0.0042	0.2167	-1.9673
Arvcf	1.2338	3.0635	3.5216	0.0042	0.2178	-1.8386
Arhgef1	-0.3755	6.4627	-3.5205	0.0042	0.2178	-2.245
Gm32591	2.554	-2.3264	3.5182	0.0043	0.2181	-2.5023
Mcc	1.3041	0.6492	3.5148	0.0043	0.2183	-1.7904
Serpinb9	1.1065	3.8732	3.5125	0.0043	0.2183	-1.968
Gm15710	-1.09	1.2322	-3.5124	0.0043	0.2183	-1.7793
Mthfd2	-0.551	6.4301	-3.5112	0.0043	0.2183	-2.2752
Tnfrsf23	-0.6809	5.1946	-3.4964	0.0044	0.2236	-2.2156
Gm6560	-0.4702	3.2895	-3.4935	0.0045	0.2236	-1.9884
Sema6b	-0.5686	3.7561	-3.4919	0.0045	0.2236	-2.0942
Antxr1	0.8454	6.5552	3.4918	0.0045	0.2236	-2.2901
Fbxo32	1.6687	3.9626	3.487	0.0045	0.2249	-1.921
Fig4	0.4499	4.7431	3.4835	0.0045	0.2252	-2.1698
Wdtc1	0.5109	6.0568	3.4832	0.0045	0.2252	-2.2853
Cgn	1.8589	1.5392	3.4778	0.0046	0.2254	-1.8351
Trib3	-0.7387	4.5182	-3.4776	0.0046	0.2254	-2.1964
Pear1	-1.9103	0.2477	-3.476	0.0046	0.2254	-1.8967
Stk11ip	0.4667	4.2479	3.4754	0.0046	0.2254	-2.1023
Tlr6	1.9623	1.6881	3.4748	0.0046	0.2254	-1.839
Irgm2	1.7463	2.2323	3.4715	0.0046	0.2262	-1.8719
Gbp10	2.2245	-0.9697	3.4674	0.0047	0.2273	-1.9805
Slc26a2	0.6686	5.5682	3.465	0.0047	0.2276	-2.2749
Cmas	-0.3448	5.5757	-3.4621	0.0047	0.2282	-2.3023
Commd9	0.5224	4.4138	3.4566	0.0048	0.2299	-2.1601
Cryzl1	-0.3722	5.4566	-3.4513	0.0048	0.2305	-2.3147
Gm13091	2.3865	-2.5778	3.4509	0.0048	0.2305	-2.456
Ncam1	1.0873	5.6953	3.4506	0.0048	0.2305	-2.3
Peak1	-0.5254	8.3824	-3.447	0.0049	0.2314	-2.4132

Atf6	-0.4075	6.6045	-3.4437	0.0049	0.2321	-2.39
Scx	0.7764	3.9367	3.4392	0.0049	0.2334	-2.1056
Plxnb1	1.8855	3.6094	3.4373	0.0049	0.2334	-1.9873
Farp1	0.3431	6.2747	3.4363	0.005	0.2334	-2.3902
Foxj1	1.9473	-2.2947	3.4326	0.005	0.2343	-2.4732
Duox2	-2.0114	-3.4688	-3.4279	0.005	0.2346	-2.5851
Casp12	1.736	5.2563	3.4259	0.0051	0.2346	-2.2514
Hexa	0.5789	6.7116	3.4253	0.0051	0.2346	-2.4145
Gm28513	-3.2072	-2.8178	-3.421	0.0051	0.2346	-2.6005
Angptl2	-0.5704	7.4786	-3.4194	0.0051	0.2346	-2.4527
Idnk	0.8275	3.7899	3.419	0.0051	0.2346	-2.1278
Osmr	1.2863	4.3876	3.4161	0.0051	0.2346	-2.2267
Ebp	0.415	5.9975	3.4158	0.0051	0.2346	-2.4076
Crebbp	0.392	6.1933	3.4152	0.0052	0.2346	-2.4232
Rpa3	-0.4805	4.8743	-3.4124	0.0052	0.2346	-2.347
Parp3	0.6212	5.0421	3.4123	0.0052	0.2346	-2.3107
Pkp2	0.7145	4.2439	3.4113	0.0052	0.2346	-2.2251
Ccn2	1.0795	3.6728	3.4086	0.0052	0.2346	-2.1512
Aldh3a1	1.6539	5.8874	3.4078	0.0052	0.2346	-2.4048
Zfp119a	0.8269	1.6185	3.407	0.0052	0.2346	-1.9594
Wdr54	-0.5467	2.8754	-3.4045	0.0053	0.2346	-2.0829
1700120C14Rik	-0.8269	0.838	-3.404	0.0053	0.2346	-1.9531
Bcl9	0.4143	5.9787	3.4038	0.0053	0.2346	-2.4243
Ankrd55	1.2248	1.4217	3.4033	0.0053	0.2346	-1.9531
Adamts6	1.1768	2.1697	3.3981	0.0053	0.2346	-1.9976
Pkd1	0.4124	6.4165	3.3979	0.0053	0.2346	-2.4606
Syne1	-0.5844	5.7112	-3.3979	0.0053	0.2346	-2.4128
Egr2	1.596	1.0037	3.3976	0.0053	0.2346	-1.965
Arhgef40	-0.4246	6.4681	-3.3975	0.0053	0.2346	-2.466
3110021N24Rik	-2.4612	-2.733	-3.3956	0.0053	0.2346	-2.6131
Ifngr1	0.5429	5.8112	3.3948	0.0053	0.2346	-2.4335
Chac1	-0.6262	3.1158	-3.3933	0.0054	0.2346	-2.1453
Cdh23	-2.3748	-2.8194	-3.388	0.0054	0.2355	-2.62
Pgm5	1.79	-3.3087	3.3873	0.0054	0.2355	-2.6506
Ano6	0.359	7.494	3.3862	0.0054	0.2355	-2.5089
Dars2	-0.3785	4.5849	-3.3853	0.0054	0.2355	-2.3584
Ppp2r3a	0.6529	6.3454	3.3832	0.0055	0.2355	-2.4842
Ube2s	-0.3432	6.0022	-3.383	0.0055	0.2355	-2.4932
Coro2a	-0.7775	5.6061	-3.3742	0.0056	0.237	-2.4724
Glns-ps1	0.9204	2.4243	3.3731	0.0056	0.237	-2.051
Gm11518	-2.7215	-3.2916	-3.3715	0.0056	0.237	-2.6435
Oaf	-0.72	7.9027	-3.3708	0.0056	0.237	-2.548
Mroh1	0.5348	6.364	3.3698	0.0056	0.237	-2.5013
Zfp108	1.7111	0.1136	3.3689	0.0056	0.237	-2.0635
Lars2	0.5762	9.8765	3.3686	0.0056	0.237	-2.5656
Angptl7	3.5363	-2.1675	3.3665	0.0056	0.237	-2.6681
Ackr3	1.0191	5.3278	3.3656	0.0056	0.237	-2.4544
Rad18	-0.362	5.5654	-3.3649	0.0057	0.237	-2.5068
Gm49395	-1.9121	-2.306	-3.3649	0.0057	0.237	-2.439
Ogn	6.5216	1.4366	3.3612	0.0057	0.2375	-2.4284
Gm7993	-0.9714	5.1938	-3.3604	0.0057	0.2375	-2.4804
Tmem19	0.7514	5.1423	3.3598	0.0057	0.2375	-2.4171

Padi2	-1.6167	4.9254	-3.3571	0.0057	0.238	-2.3831
Itih2	1.6692	2.3405	3.3561	0.0057	0.238	-2.079
Pisd-ps1	0.9144	1.1268	3.3514	0.0058	0.2395	-2.0341
Epha2	-0.8529	6.1685	-3.3495	0.0058	0.2396	-2.5716
Rufy4	-1.4537	0.0467	-3.348	0.0058	0.2396	-2.0925
Cdk1	-0.3221	6.8158	-3.3467	0.0058	0.2396	-2.5817
Tmco4	0.6977	3.1748	3.3456	0.0059	0.2396	-2.1876
Clcn7	0.387	5.4217	3.3436	0.0059	0.2396	-2.4876
Asb13	0.368	4.6979	3.3434	0.0059	0.2396	-2.4225
Gm4784	-1.8927	-1.3885	-3.3421	0.0059	0.2396	-2.3615
Zmiz1	0.5027	7.801	3.3405	0.0059	0.2397	-2.5971
Thbs1	2.4916	5.6899	3.339	0.0059	0.2399	-2.4251
Plk3	-0.7931	4.7817	-3.3345	0.006	0.2408	-2.4875
Cipc	0.5124	5.1665	3.3343	0.006	0.2408	-2.4806
Nfkbie	1.1552	4.874	3.3328	0.006	0.2409	-2.4042
Bahcc1	0.4751	6.2185	3.3257	0.0061	0.2432	-2.5863
Apol9a	1.9173	2.8591	3.3246	0.0061	0.2432	-2.1412
Setd7	0.3824	7.7887	3.3201	0.0061	0.2432	-2.6355
Hist1h4k	1.4415	-0.932	3.3194	0.0061	0.2432	-2.2031
Frmd6	0.4258	6.6088	3.3189	0.0062	0.2432	-2.6175
Rfx5	0.449	4.9363	3.3189	0.0062	0.2432	-2.4719
Nfkb1	0.3449	7.562	3.318	0.0062	0.2432	-2.6383
Kansl1	0.3393	6.0627	3.3177	0.0062	0.2432	-2.5897
Crispld2	1.5058	4.571	3.3153	0.0062	0.2435	-2.3913
Ripk2	0.5664	4.3998	3.3145	0.0062	0.2435	-2.4146
Psph	-0.5737	5.122	-3.3102	0.0063	0.2438	-2.5625
Uap1l1	0.6501	6.2737	3.3097	0.0063	0.2438	-2.6029
Synrg	0.599	5.5803	3.3096	0.0063	0.2438	-2.5536
Dgkd	-0.4997	7.0457	-3.3089	0.0063	0.2438	-2.6474
Nfib	0.3121	7.0331	3.3072	0.0063	0.2441	-2.6493
Kank2	0.5919	6.514	3.305	0.0063	0.2444	-2.6294
Ccn4	1.8731	2.9033	3.304	0.0063	0.2444	-2.2054
Gm9115	-1.1909	-0.127	-3.3025	0.0063	0.2444	-2.1265
Mgat3	0.6614	6.0879	3.3015	0.0064	0.2444	-2.6148
Hist2h3c2	2.7323	-0.8229	3.299	0.0064	0.2445	-2.4358
Pdgfra	0.8672	7.1235	3.2988	0.0064	0.2445	-2.6635
Pqlc3	-0.4975	4.2639	-3.2951	0.0064	0.2455	-2.4547
Tmem176b	0.8739	7.6443	3.2942	0.0064	0.2455	-2.6791
Slc11a2	0.4575	6.6423	3.2928	0.0065	0.2456	-2.6652
Gmnn	-0.3306	5.5136	-3.2884	0.0065	0.2471	-2.6479
Tsc22d3	0.8002	6.9777	3.2865	0.0065	0.2474	-2.6736
Zfp263	0.4719	5.1067	3.2841	0.0066	0.248	-2.5702
Clvs1	-2.4168	-2.0469	-3.2789	0.0066	0.2498	-2.5248
Tmem44	2.5512	1.339	3.2759	0.0067	0.2504	-2.1746
Odc1	-0.9882	9.0937	-3.2744	0.0067	0.2504	-2.7363
9330188P03Rik	-0.97	2.7005	-3.274	0.0067	0.2504	-2.2884
Rnf183	-1.4155	1.9725	-3.2717	0.0067	0.2504	-2.225
Gpt2	-0.557	5.759	-3.2698	0.0067	0.2504	-2.6765
Il13ra1	0.8413	6.7998	3.2697	0.0067	0.2504	-2.7142
Frk	1.0268	3.9655	3.2693	0.0067	0.2504	-2.4095
Sema3f	0.8631	4.6209	3.2645	0.0068	0.251	-2.5116
Gnaz	1.4985	-0.1402	3.2644	0.0068	0.251	-2.2264

Tspan17	0.7666	4.2794	3.2637	0.0068	0.251	-2.4613
Tgm2	1.288	7.7703	3.2626	0.0068	0.251	-2.7347
Fn3krp	0.4415	4.1753	3.2623	0.0068	0.251	-2.5004
Fam118b	0.414	4.562	3.2595	0.0069	0.251	-2.546
Tor1aip1	0.3695	6.426	3.2571	0.0069	0.251	-2.7225
Spred2	-0.6026	5.7767	-3.2556	0.0069	0.251	-2.724
Parvg	-1.384	-1.0307	-3.2553	0.0069	0.251	-2.3158
Gm45237	-1.8164	-2.315	-3.2542	0.0069	0.251	-2.5747
Znfx1	0.7724	4.6271	3.2538	0.0069	0.251	-2.538
Asb5	2.4445	-2.8269	3.2537	0.0069	0.251	-2.7456
Vcan	0.9679	8.4002	3.2531	0.0069	0.251	-2.7653
9930004E17Rik	2.1267	-2.7665	3.2506	0.007	0.2513	-2.7101
Gm20559	1.8137	-2.1486	3.2503	0.007	0.2513	-2.532
Syt11	1.0751	3.7153	3.2478	0.007	0.2514	-2.4026
Sparc	1.0546	9.1075	3.2477	0.007	0.2514	-2.7808
Spred3	-0.6931	4.1979	-3.2447	0.0071	0.252	-2.5868
Parva	0.447	7.1711	3.2443	0.0071	0.252	-2.7639
Gm7867	-2.1842	-1.752	-3.2378	0.0071	0.2537	-2.5902
Nrep	4.8349	2.8427	3.2377	0.0071	0.2537	-2.2385
Gm27326	-1.5741	-1.3895	-3.2373	0.0072	0.2537	-2.4
Nptxr	-0.6356	6.1108	-3.2359	0.0072	0.2537	-2.7563
Trps1	0.3832	7.4014	3.2328	0.0072	0.2537	-2.7884
Rhbdl3	2.5637	-1.4789	3.2327	0.0072	0.2537	-2.5619
Gm26947	-1.0639	-0.7426	-3.2324	0.0072	0.2537	-2.2459
Procr	-0.569	4.2395	-3.2307	0.0072	0.2537	-2.5929
S1pr1	2.3452	1.9751	3.2305	0.0072	0.2537	-2.2402
Car11	1.9685	-2.0482	3.226	0.0073	0.2551	-2.5873
Acp2	0.3528	5.2708	3.2249	0.0073	0.2551	-2.6858
Nacc2	1.0664	4.4409	3.2243	0.0073	0.2551	-2.538
Prkca	-0.3172	6.3234	-3.2232	0.0073	0.2551	-2.7907
Chchd1	-0.3774	5.912	-3.2219	0.0074	0.2552	-2.7735
Nrbp2	1.721	1.7636	3.2196	0.0074	0.2557	-2.2424
D5Ertd579e	0.4408	5.7192	3.2188	0.0074	0.2557	-2.7474
Gm26826	2.0201	-2.1456	3.2176	0.0074	0.2557	-2.6414
Glmp	0.3503	7.3337	3.2151	0.0075	0.2564	-2.8179
Cd9	-0.4769	8.6494	-3.2085	0.0075	0.2577	-2.8513
Extl1	1.364	1.4434	3.208	0.0076	0.2577	-2.2601
4933427E11Rik	1.9954	-2.9124	3.2073	0.0076	0.2577	-2.7529
Gm7278	-1.002	0.3187	-3.2072	0.0076	0.2577	-2.2597
Dag1	0.3304	8.4884	3.207	0.0076	0.2577	-2.8506
Sult1a1	2.0813	-2.5553	3.2059	0.0076	0.2577	-2.7902
Filip1l	0.9283	5.1417	3.2025	0.0076	0.2577	-2.7135
Enc1	0.7923	5.3672	3.202	0.0076	0.2577	-2.7476
Nfix	0.5158	7.3106	3.2018	0.0076	0.2577	-2.8439
Gm29054	-1.9722	-2.6405	-3.2016	0.0076	0.2577	-2.6591
Mmp14	0.8852	7.4641	3.1976	0.0077	0.2592	-2.8517
1700065D16Rik	1.3166	-1.9252	3.1948	0.0077	0.26	-2.539
Cnnm2	1.1094	2.8691	3.1929	0.0078	0.2604	-2.3973
Gm13862	-1.9857	-1.4887	-3.1908	0.0078	0.2609	-2.5607
Sapcd2	-0.4055	3.7726	-3.1889	0.0078	0.2613	-2.6742
Gbp7	1.7665	4.0038	3.1807	0.0079	0.2638	-2.5617
Tmem107	-0.4362	3.7244	-3.179	0.008	0.2638	-2.6213

Ets2	0.6136	6.8912	3.1788	0.008	0.2638	-2.8793
Egflam	-3.0109	-2.249	-3.1788	0.008	0.2638	-2.8237
Ccdc77	-0.3403	4.3519	-3.1785	0.008	0.2638	-2.7031
Atp6v0a2	0.3736	6.0861	3.1765	0.008	0.2643	-2.8488
Tent5b	2.2626	2.9773	3.1731	0.0081	0.2654	-2.3763
Itga6	-0.6807	7.3611	-3.1706	0.0081	0.2662	-2.905
Zfp217	-0.3276	6.0144	-3.1677	0.0081	0.2668	-2.8692
Gm12185	2.0035	-3.4569	3.1672	0.0081	0.2668	-2.6945
Omd	3.9363	-1.9155	3.1651	0.0082	0.2668	-2.8738
Washc2	0.3706	7.2474	3.1647	0.0082	0.2668	-2.9075
Fbn1	1.4508	0.976	3.1622	0.0082	0.2668	-2.355
Hspd1-ps3	-0.4925	2.0687	-3.1616	0.0082	0.2668	-2.432
Vps13c	0.486	6.1798	3.161	0.0082	0.2668	-2.8799
Lrch3	0.357	5.4998	3.1605	0.0082	0.2668	-2.8379
Casq2	2.3315	-2.0199	3.1596	0.0083	0.2668	-2.7466
Mmp16	1.6722	0.6207	3.1591	0.0083	0.2668	-2.335
Aff1	0.376	6.3575	3.1572	0.0083	0.2669	-2.8978
Hist1h2bf	2.3987	-2.9195	3.1569	0.0083	0.2669	-2.8017
Gm4544	1.2099	1.0072	3.1535	0.0084	0.2681	-2.3434
Cblb	0.5671	6.2141	3.1486	0.0084	0.27	-2.9093
Abca9	2.9274	-1.3801	3.1463	0.0085	0.2702	-2.6804
Junos	-0.7512	1.5744	-3.1454	0.0085	0.2702	-2.4084
Ap3d1	0.3305	6.5093	3.1452	0.0085	0.2702	-2.9259
Erap1	0.6825	6.557	3.1425	0.0085	0.2709	-2.9346
Ehbp1l1	-0.3604	6.3947	-3.1419	0.0085	0.2709	-2.9377
Zbtb43	0.3797	4.8348	3.1327	0.0087	0.2735	-2.8133
Gm24245	0.8413	2.4235	3.1323	0.0087	0.2735	-2.4839
Cdca3	-0.3723	5.7786	-3.1312	0.0087	0.2735	-2.9497
Arhgef17	0.7838	7.2758	3.1311	0.0087	0.2735	-2.9707
Rhou	1.336	6.5268	3.1306	0.0087	0.2735	-2.9335
Tlr3	1.0472	1.9464	3.1299	0.0087	0.2735	-2.4364
Insyn1	0.9943	1.6512	3.1299	0.0087	0.2735	-2.4203
Rab3il1	2.1728	1.3461	3.1279	0.0088	0.274	-2.3846
Gdf5	2.7973	-3.2118	3.1268	0.0088	0.274	-2.8721
P2rx6	2.2748	-1.9758	3.1246	0.0088	0.2746	-2.7511
Wdpcp	0.7179	2.2039	3.1226	0.0088	0.2748	-2.4608
Ppm1j	-1.0426	-0.9478	-3.1222	0.0089	0.2748	-2.4465
Vgf	-1.2559	-0.4376	-3.1177	0.0089	0.2762	-2.4322
Hist1h2al	1.4444	0.4132	3.1176	0.0089	0.2762	-2.4288
Nmb	1.9859	-1.8624	3.1132	0.009	0.2777	-2.7241
Fermt3	-0.9931	0.6019	-3.1128	0.009	0.2777	-2.4108
Tnn	3.4159	-1.1444	3.111	0.009	0.2778	-2.7533
Gm16793	2.2772	-2.6572	3.1107	0.009	0.2778	-2.8802
Rubcn	0.3635	5.561	3.1093	0.0091	0.2778	-2.9392
Abca1	1.5398	3.7681	3.1075	0.0091	0.2778	-2.5796
Gipc3	-1.3448	-0.5878	-3.1068	0.0091	0.2778	-2.4712
Macc1	1.8525	0.921	3.1067	0.0091	0.2778	-2.4163
Cpeb4	0.7178	5.2385	3.1059	0.0091	0.2778	-2.8847
Cdyl2	-0.566	3.7851	-3.1044	0.0092	0.2781	-2.7242
Gm29340	-2.2864	-3.1324	-3.0984	0.0093	0.2793	-2.8925
Slc46a1	0.7113	2.1963	3.0984	0.0093	0.2793	-2.4988
Tns3	0.6402	8.0041	3.0974	0.0093	0.2793	-3.0437

Rev3l	0.6093	5.7669	3.0932	0.0093	0.2793	-2.9754
Fuca2	0.7395	3.6498	3.0915	0.0094	0.2793	-2.6684
Twist1	-0.313	6.4905	-3.0915	0.0094	0.2793	-3.034
Snord73a	1.2476	-1.8678	3.0904	0.0094	0.2793	-2.6076
Tbc1d5	0.2841	5.4158	3.0897	0.0094	0.2793	-2.959
Snta1	1.0368	4.6164	3.0889	0.0094	0.2793	-2.8116
Parpbp	-0.4235	4.0355	-3.0886	0.0094	0.2793	-2.8676
Uaca	-0.4699	6.7343	-3.0879	0.0094	0.2793	-3.046
Lrig2	0.3839	4.7208	3.0873	0.0094	0.2793	-2.8709
Asap1	0.3187	7.4745	3.0872	0.0094	0.2793	-3.0558
Adck1	0.5684	3.9866	3.0863	0.0095	0.2793	-2.7677
Gdnf	1.2837	2.7994	3.0856	0.0095	0.2793	-2.5855
Gm42418	0.5522	12.7267	3.0853	0.0095	0.2793	-3.0914
Zwilch	-0.2893	5.3028	-3.0849	0.0095	0.2793	-3.0037
Gm5835	-0.7265	2.0352	-3.0843	0.0095	0.2793	-2.5411
Rps27-ps1	-1.8602	-1.9137	-3.0838	0.0095	0.2793	-2.6989
Gm11100	-1.8544	-2.5084	-3.0833	0.0095	0.2793	-2.7844
Tmem176a	1.0364	7	3.0823	0.0095	0.2793	-3.0524
Notch3	1.5365	0.5403	3.0811	0.0096	0.2795	-2.4548
Kcnj2	2.7976	1.5439	3.0798	0.0096	0.2797	-2.4568
Gm15032	-1.9496	-1.8985	-3.0773	0.0096	0.2801	-2.7439
Gbp2b	2.5783	-0.0384	3.0767	0.0096	0.2801	-2.5076
Gm14130	-1.2317	-0.6967	-3.076	0.0096	0.2801	-2.5038
Parp10	0.7695	4.8077	3.0753	0.0097	0.2801	-2.8885
Trp53inp1	1.6071	4.7126	3.0735	0.0097	0.2802	-2.7695
4933406C10Rik	-2.2728	-2.8269	-3.0734	0.0097	0.2802	-2.8833
Gm24187	0.7784	5.2281	3.0709	0.0097	0.281	-2.9684
Tnfrsf22	-0.5833	4.559	-3.0695	0.0098	0.2813	-2.9165
Tcf7l1	0.6816	3.5448	3.0685	0.0098	0.2813	-2.7101
Tmem140	3.6403	0.934	3.0667	0.0098	0.2818	-2.5732
Gas7	1.1882	7.5669	3.0634	0.0099	0.2827	-3.0984
Map3k6	-0.8263	4.1955	-3.0632	0.0099	0.2827	-2.8611
Mars	-0.3205	6.6743	-3.0605	0.0099	0.2836	-3.0967
Sh3kbp1	-0.357	7.294	-3.0562	0.01	0.2854	-3.1135
4931406P16Rik	0.4055	5.9811	3.0542	0.01	0.286	-3.0579
Rnf213	0.5662	7.0234	3.0527	0.0101	0.2863	-3.1088
Gng7	1.174	0.4936	3.0496	0.0101	0.2875	-2.5043
Gm43592	-1.5331	-2.2204	-3.0472	0.0102	0.2879	-2.7451
Gm16098	1.6469	-2.3734	3.047	0.0102	0.2879	-2.865
Lrrc58	0.4282	6.9277	3.0447	0.0102	0.2885	-3.1222
C1ra	3.0799	1.9263	3.0442	0.0102	0.2885	-2.514
Lbr	-0.4129	6.8892	-3.0413	0.0103	0.2891	-3.1379
Evi2a	-1.1241	2.4025	-3.0407	0.0103	0.2891	-2.6414
Gbp3	2.0083	3.6167	3.0404	0.0103	0.2891	-2.73
Tgfbr3	0.4047	7.1885	3.0373	0.0104	0.2898	-3.1452
Loxl3	-0.7386	4.5894	-3.0358	0.0104	0.2898	-2.9696
Epb41l2	0.5571	6.8483	3.0353	0.0104	0.2898	-3.1375
Gm10224	-1.1376	-0.7848	-3.0352	0.0104	0.2898	-2.5839
Zfp36l1	0.5921	6.5588	3.0348	0.0104	0.2898	-3.1276
Gm5654	-1.3731	-0.5803	-3.0332	0.0104	0.2902	-2.6026
Rps18-ps1	-0.9646	0.4845	-3.0297	0.0105	0.2916	-2.5336
Ifit3	1.7447	0.4591	3.0234	0.0106	0.2939	-2.5443

Arhgap31	0.6004	5.3874	3.023	0.0106	0.2939	-3.0738
Rab40b	1.6746	1.4329	3.0228	0.0106	0.2939	-2.5512
Glb1l	0.6338	2.0045	3.022	0.0107	0.2939	-2.619
Rpl36-ps3	-1.572	-1.772	-3.0204	0.0107	0.2943	-2.6822
Arid5b	0.3754	6.2898	3.0193	0.0107	0.2944	-3.1484
Sptlc1	0.2841	5.4823	3.0174	0.0108	0.295	-3.101
C1qtnf6	1.0826	5.8804	3.0143	0.0108	0.296	-3.1041
Atoh8	2.7395	-0.1566	3.0138	0.0108	0.296	-2.6848
Pld1	1.1441	4.7977	3.0125	0.0109	0.2962	-2.9648
Tgfa	-0.9871	3.6581	-3.0096	0.0109	0.2974	-2.8863
Mgst1	1.4052	5.7616	3.0075	0.011	0.2978	-3.095
Pcdhb16	0.813	1.4423	3.0063	0.011	0.2978	-2.5893
Cks2	-0.3071	5.8769	-3.0056	0.011	0.2978	-3.178
Dvl3	0.3957	5.6809	3.0055	0.011	0.2978	-3.1282
Rpl36-ps12	-0.9335	1.0119	-3.0034	0.011	0.298	-2.589
Nr1d1	-0.5391	4.4393	-3.0033	0.011	0.298	-3.0128
Sohlh1	3.4741	-1.3215	3.0019	0.0111	0.2984	-2.8165
Adamtsl4	1.0111	5.0485	3.0008	0.0111	0.2984	-3.0458
Vps33a	0.3638	6.2057	2.9999	0.0111	0.2984	-3.1771
Zfp784	1.4463	0.269	2.9986	0.0111	0.2984	-2.5917
Gm2614	-1.3366	-0.8953	-2.9984	0.0111	0.2984	-2.6457
Narf	0.3585	5.8934	2.9976	0.0112	0.2984	-3.1605
2310043P16Rik	2.2447	-1.5321	2.9964	0.0112	0.2986	-2.8511
Prss23	2.8741	3.299	2.9927	0.0113	0.3002	-2.682
Pycr1	-0.5361	3.9848	-2.9903	0.0113	0.3008	-2.968
Arhgap19	-0.4118	5.3287	-2.99	0.0113	0.3008	-3.1839
Nckap1l	-1.2145	0.3213	-2.9886	0.0113	0.3011	-2.6017
Gm4800	-1.4024	-1.9954	-2.9865	0.0114	0.3018	-2.7612
Rpl30-ps1	-1.2165	0.262	-2.9853	0.0114	0.302	-2.6029
Bcar1	0.3532	6.6385	2.9839	0.0114	0.302	-3.2274
Pgf	-1.5157	-0.4717	-2.9834	0.0115	0.302	-2.6092
Myl6	0.4409	7.6817	2.9821	0.0115	0.302	-3.2493
Gm12356	1.0674	-0.9862	2.9812	0.0115	0.302	-2.6725
Dusp5	-0.5447	3.7973	-2.9812	0.0115	0.302	-3.035
Gm5513	-0.8812	-0.1689	-2.9797	0.0115	0.3023	-2.6145
Brpf3	0.2893	5.5688	2.979	0.0116	0.3023	-3.1686
Pdlim5	0.6891	7.7654	2.9766	0.0116	0.3029	-3.2583
Hagh	-0.4337	5.8632	-2.9762	0.0116	0.3029	-3.2009
Alox8	-1.6958	-2.4911	-2.9747	0.0116	0.3034	-2.9028
Enpp5	2.2351	1.2514	2.9721	0.0117	0.3043	-2.6212
Fabp4	-1.2505	3.6018	-2.9714	0.0117	0.3043	-2.8935
D430020J02Rik	0.7484	2.7783	2.9703	0.0117	0.3044	-2.7887
Nelfb	-0.2915	6.1219	-2.9695	0.0118	0.3045	-3.2383
Fosl2	0.5841	6.4174	2.9683	0.0118	0.3047	-3.2428
1810058I24Rik	0.6153	4.9396	2.967	0.0118	0.3049	-3.0926
Irgq	0.5691	5.005	2.9647	0.0119	0.3058	-3.119
Gm27365	1.7059	-2.6393	2.9624	0.0119	0.3066	-2.9142
Nfatc1	0.5265	4.8605	2.9614	0.0119	0.3067	-3.1226
D630036H23Rik	2.3148	-3.2512	2.9606	0.012	0.3067	-3.0247
Gm5814	-1.1897	-1.9428	-2.9588	0.012	0.3069	-2.7647
Ror1	1.4644	2.4678	2.9579	0.012	0.3069	-2.7446
Trim17	2.0715	-3.1535	2.9568	0.012	0.3069	-3.0422

Mvp	0.5222	6.7126	2.9564	0.012	0.3069	-3.2724
Hmga1b	-0.7096	8.1156	-2.9562	0.012	0.3069	-3.3093
Car7	1.4169	-1.0565	2.9546	0.0121	0.3074	-2.7047
Sectm1a	2.4345	-1.971	2.9529	0.0121	0.3074	-2.8836
Trim34b	1.4597	-0.7669	2.952	0.0121	0.3074	-2.7079
Spin1	0.321	7.1728	2.9514	0.0122	0.3074	-3.298
C630043F03Rik	0.8937	1.1694	2.9507	0.0122	0.3074	-2.6896
CR555305.3	-1.2388	-0.7399	-2.9504	0.0122	0.3074	-2.6865
Ddit3	-0.5691	4.0218	-2.9499	0.0122	0.3074	-3.0378
Tbc1d24	0.4288	4.3474	2.947	0.0123	0.3086	-3.0695
Mfsd1	0.3459	8.0318	2.946	0.0123	0.3087	-3.3189
Micall2	-0.3809	5.9698	-2.9416	0.0124	0.31	-3.2767
Pvr	-0.426	5.9019	-2.9407	0.0124	0.31	-3.2832
Tmem63b	0.3305	6.4046	2.9405	0.0124	0.31	-3.2969
Gm12583	-1.6928	-1.9126	-2.9402	0.0124	0.31	-2.822
Hexb	0.6419	6.1318	2.9394	0.0124	0.31	-3.2726
Atp6v0d1	0.5763	6.3336	2.939	0.0124	0.31	-3.2829
Ajuba	0.9054	5.2547	2.9378	0.0125	0.3102	-3.1947
Gm33347	-1.3867	-1.9575	-2.9356	0.0125	0.3108	-2.78
Aurka	-0.3763	6.2699	-2.9352	0.0125	0.3108	-3.3202
Syngap1	0.9141	2.1182	2.9338	0.0126	0.3108	-2.7653
Gm11772	1.6104	-1.5566	2.933	0.0126	0.3108	-2.8152
Gm16538	-1.861	-2.5972	-2.9325	0.0126	0.3108	-2.9108
Tubb4b-ps2	-1.5498	-1.9239	-2.9322	0.0126	0.3108	-2.8146
March2	0.5158	4.3122	2.9306	0.0126	0.3112	-3.0804
Zcchc24	0.3681	6.9849	2.9299	0.0127	0.3112	-3.3342
Phip	0.3623	7.3659	2.9285	0.0127	0.3113	-3.3417
Pls3	-0.2811	8.5997	-2.9283	0.0127	0.3113	-3.3607
Zbtb33	0.4184	5.5977	2.9271	0.0127	0.3115	-3.271
Nek6	0.5083	6.4369	2.9258	0.0127	0.3118	-3.3306
H2-T10	0.7311	3.4411	2.9242	0.0128	0.3118	-2.9574
Rapgef3	-0.9593	4.9015	-2.9232	0.0128	0.3118	-3.2644
Sgk2	-1.4388	1.6051	-2.9228	0.0128	0.3118	-2.7744
Elmsan1	0.2827	5.8914	2.9228	0.0128	0.3118	-3.3126
Ap5z1	0.4094	3.8435	2.9221	0.0128	0.3118	-3.0125
Ubtd1	-0.5452	5.913	-2.9195	0.0129	0.3126	-3.3191
AW146154	0.8519	1.6507	2.9178	0.0129	0.3126	-2.7487
Gbp9	2.7175	2.4435	2.9172	0.013	0.3126	-2.753
Lrrfip1	-0.2986	7.3889	-2.9169	0.013	0.3126	-3.3678
Nlrp12	-2.0116	-2.8791	-2.9168	0.013	0.3126	-2.997
Gm13981	-1.8144	-2.0566	-2.9159	0.013	0.3127	-2.9
Ypel2	0.5574	3.9013	2.9127	0.0131	0.3141	-3.0278
Snx22	-0.6118	2.3171	-2.9099	0.0131	0.3149	-2.8384
Ddx60	2.2393	-0.2607	2.9095	0.0131	0.3149	-2.7525
Ifi47	1.879	-0.0529	2.9092	0.0131	0.3149	-2.7185
Golt1b	-0.2809	6.4952	-2.9059	0.0132	0.3156	-3.3689
AC150274.1	-1.4148	-1.5298	-2.9053	0.0132	0.3156	-2.8098
Irf9	0.7804	3.5473	2.9052	0.0132	0.3156	-3.0081
Tcf7l2	0.65	2.9558	2.9036	0.0133	0.3156	-2.9225
Tspan11	0.4325	6.2645	2.9022	0.0133	0.3156	-3.3609
Slc29a3	0.6872	3.8173	2.9022	0.0133	0.3156	-3.0433
Gm49064	0.8887	-0.1835	2.902	0.0133	0.3156	-2.7346

Fam229b	0.902	0.6747	2.9019	0.0133	0.3156	-2.7334
Trafd1	0.4623	4.6917	2.8995	0.0134	0.3166	-3.1893
Nedd9	0.9401	4.4522	2.8987	0.0134	0.3166	-3.1768
Prr15l	3.262	-0.1785	2.895	0.0135	0.3182	-2.8893
Ube2e2	0.4868	4.9082	2.8944	0.0135	0.3182	-3.2422
Fbf1	-0.3492	6.4644	-2.8938	0.0135	0.3182	-3.388
Urgcp	-0.3803	6.776	-2.8921	0.0136	0.3185	-3.3988
Gm49891	-1.0467	-0.4814	-2.8912	0.0136	0.3185	-2.7477
Sgms2	-0.5866	5.4807	-2.8911	0.0136	0.3185	-3.3443
Nbeal1	0.5085	5.2108	2.8881	0.0137	0.3196	-3.2818
Cdkn2d	-0.4734	4.5649	-2.8877	0.0137	0.3196	-3.2802
Pdgfc	2.3093	4.1048	2.8853	0.0137	0.3206	-2.9971
Apol9b	1.4786	2.1542	2.8842	0.0138	0.3209	-2.853
Ankrd12	0.4374	5.6367	2.8826	0.0138	0.3214	-3.3408
Wnt10b	1.3116	2.7995	2.8785	0.0139	0.3234	-2.9276
Ankrd61	1.7284	-1.1724	2.8758	0.014	0.3245	-2.8707
Maf	0.922	5.3422	2.8741	0.014	0.325	-3.3072
Tesk1	0.4311	4.8058	2.8731	0.0141	0.325	-3.2719
Dagla	-0.609	4.7286	-2.8723	0.0141	0.325	-3.2953
Ifnar2	0.6085	5.0452	2.8713	0.0141	0.325	-3.3091
Gm13387	-1.8164	0.5573	-2.8708	0.0141	0.325	-2.8012
Iigp1	1.319	1.0321	2.8707	0.0141	0.325	-2.9691
Atp1b1	2.0627	1.209	2.8695	0.0142	0.3253	-2.7823
Rita1	-0.3822	3.6836	-2.867	0.0142	0.3264	-3.1542
Os9	0.4695	5.9469	2.8645	0.0143	0.3271	-3.4012
BC001981	-1.4277	-1.085	-2.8643	0.0143	0.3271	-2.8472
Amotl2	0.9774	5.5807	2.8624	0.0143	0.3278	-3.367
Atp11a	0.3964	6.5919	2.8618	0.0144	0.3278	-3.4422
Cideb	2.371	-1.6248	2.8592	0.0144	0.329	-3.0023
Gramd4	-0.4239	7.0389	-2.8584	0.0144	0.329	-3.465
Kcnt1	2.9879	-1.1919	2.8544	0.0146	0.331	-2.9899
Ptgfrn	0.5907	7.1783	2.8503	0.0147	0.3331	-3.4781
Rhebl1	-0.4684	3.6713	-2.849	0.0147	0.3334	-3.1723
5830432E09Rik	-1.4616	-2.0431	-2.8474	0.0147	0.3334	-2.8914
Zxdc	0.4095	5.0773	2.8471	0.0147	0.3334	-3.3478
Ash1l	0.3808	7.3444	2.8469	0.0148	0.3334	-3.4894
Ribc1	-0.5237	2.1343	-2.8415	0.0149	0.3361	-2.9618
Slc35e3	0.41	4.1179	2.8412	0.0149	0.3361	-3.2222
Hist1h4j	1.1857	-0.9704	2.8387	0.015	0.3371	-2.8616
Echdc2	-0.4537	3.706	-2.8381	0.015	0.3371	-3.1786
Gm13410	1.7093	-1.4933	2.8373	0.015	0.3372	-2.9205
Gch1	-0.822	3.4584	-2.8362	0.0151	0.3374	-3.1291
Ccdc88c	0.5202	5.1799	2.835	0.0151	0.3374	-3.3656
Lamp1	0.3284	9.6495	2.8349	0.0151	0.3374	-3.5357
Mgst2	-0.4033	5.5805	-2.832	0.0152	0.3384	-3.4424
Tmem39a	-0.4352	5.3702	-2.8319	0.0152	0.3384	-3.4355
Stard13	0.8516	4.0439	2.8297	0.0152	0.3393	-3.2137
Washc5	0.3801	7.6927	2.8289	0.0153	0.3394	-3.5272
Unc13a	-0.9798	2.8206	-2.826	0.0153	0.3406	-3.0724
Cnn3	0.2749	8.5805	2.8248	0.0154	0.3406	-3.5461
Ctbs	-0.4266	4.0735	-2.8247	0.0154	0.3406	-3.2791
C130089K02Rik	-1.7335	-2.347	-2.8235	0.0154	0.3406	-3.0962

Tgfbi	1.2149	6.8152	2.8233	0.0154	0.3406	-3.5084
mt-Nd2	0.4595	10.0049	2.8228	0.0154	0.3406	-3.5595
Gm14167	-1.3781	-1.1327	-2.8217	0.0155	0.3408	-2.9011
Gm8181	-1.8687	-2.4438	-2.82	0.0155	0.3408	-3.0989
Gm5586	-0.9182	-0.1045	-2.8194	0.0155	0.3408	-2.8501
Enpp2	1.5529	6.2161	2.8191	0.0155	0.3408	-3.472
Rac3	1.059	1.8225	2.8191	0.0155	0.3408	-2.9424
Rps4x-ps	-0.8207	0.3101	-2.8164	0.0156	0.3413	-2.8624
Nt5c2	0.3602	5.1265	2.8161	0.0156	0.3413	-3.4042
Trim69	-1.4934	-1.2878	-2.8148	0.0157	0.3413	-2.9186
Mafb	1.1577	2.8427	2.814	0.0157	0.3413	-3.0666
Nr2f2	0.6847	6.4901	2.814	0.0157	0.3413	-3.5263
Gm37240	1.3786	-1.1098	2.8115	0.0158	0.3413	-2.9232
Dynlt1f	-0.3999	3.9212	-2.8114	0.0158	0.3413	-3.294
Gm7407	-2.2862	-2.8489	-2.8092	0.0158	0.3413	-3.1086
C230038L03Rik	-1.2166	-2.2372	-2.8084	0.0158	0.3413	-2.9967
Gm33378	1.2217	-1.5614	2.8058	0.0159	0.3413	-2.9528
P3h2	0.9334	1.9763	2.8052	0.0159	0.3413	-2.9798
Vwa1	1.1479	2.7048	2.805	0.0159	0.3413	-3.0733
Trim56	0.4643	6.269	2.8038	0.016	0.3413	-3.5303
Zkscan1	0.3834	5.8261	2.8037	0.016	0.3413	-3.4988
Klhdc2	-0.2482	6.9888	-2.8037	0.016	0.3413	-3.5625
Pip4k2c	0.3382	5.6648	2.8032	0.016	0.3413	-3.4946
Vps50	0.2901	6.4958	2.8027	0.016	0.3413	-3.5488
Brdt	0.7362	1.8261	2.8027	0.016	0.3413	-2.9751
2010204K13Rik	-0.6336	3.1222	-2.8026	0.016	0.3413	-3.2265
Snx18	0.4919	6.8414	2.8021	0.016	0.3413	-3.555
Zfp628	0.403	3.8999	2.8019	0.016	0.3413	-3.27
Mxra8	0.5503	5.9811	2.8016	0.016	0.3413	-3.5028
Tex2	-0.5212	6.0152	-2.8011	0.0161	0.3413	-3.5388
Als2	0.3742	5.5144	2.7999	0.0161	0.3413	-3.4847
Hs6st1	0.4859	4.6792	2.7997	0.0161	0.3413	-3.382
Lipt2	-0.6316	2.414	-2.7995	0.0161	0.3413	-3.1133
Irf7	1.9242	0.8558	2.7989	0.0161	0.3413	-2.8975
Ehf	2.7231	-1.7483	2.7989	0.0161	0.3413	-3.0593
Nipal3	1.2705	2.0442	2.7986	0.0161	0.3413	-2.9381
Plcd4	2.3039	-3.2785	2.7973	0.0162	0.3417	-3.1918
Ogfr	0.5204	6.3752	2.7948	0.0162	0.3418	-3.5544
Mid1	1.1534	2.7435	2.7947	0.0163	0.3418	-3.0728
Lrrc8d	0.2762	6.3638	2.7932	0.0163	0.3418	-3.5646
Prr16	2.9917	-2.9772	2.793	0.0163	0.3418	-3.1941
Adora1	-2.0244	-2.9931	-2.7928	0.0163	0.3418	-3.1263
Cyld	0.4657	6.008	2.7927	0.0163	0.3418	-3.5419
Casp3	-0.5344	8.7802	-2.7917	0.0163	0.3418	-3.6095
Ip6k1	0.2611	6.2058	2.7915	0.0164	0.3418	-3.5493
Frs2	0.3333	5.5476	2.7912	0.0164	0.3418	-3.5069
Ppp4r2	0.2259	7.4003	2.7872	0.0165	0.3431	-3.599
Pdgfrb	2.8937	2.9373	2.7869	0.0165	0.3431	-2.9987
Cd44	-0.6546	8.4118	-2.7862	0.0165	0.3431	-3.618
Birc5	-0.2384	6.3221	-2.786	0.0165	0.3431	-3.5942
Magi1	-0.4173	5.6137	-2.7859	0.0165	0.3431	-3.5321
Dnah17	1.9276	-1.5015	2.7843	0.0166	0.3433	-3.0497

Ube2l6	0.8914	3.9011	2.7842	0.0166	0.3433	-3.2846
Hspa9-ps1	-1.1698	-0.6805	-2.7824	0.0166	0.344	-2.9149
Gab1	0.6988	4.1334	2.7818	0.0166	0.344	-3.2929
Cfap53	1.937	-2.2059	2.7811	0.0167	0.3441	-3.0733
Jund	0.709	7.4903	2.7801	0.0167	0.3443	-3.6084
Rin3	0.7714	6.3696	2.7783	0.0168	0.3449	-3.5725
Tug1	0.2792	7.2653	2.7774	0.0168	0.3449	-3.6124
Ttpal	0.3459	5.7114	2.7766	0.0168	0.3449	-3.5503
Aurkb	-0.305	5.6959	-2.7766	0.0168	0.3449	-3.5937
Stat3	0.4594	7.453	2.7736	0.0169	0.3451	-3.6224
Sh2b3	-0.8139	5.6874	-2.7724	0.0169	0.3451	-3.5674
Tead3	0.9263	3.3847	2.7713	0.017	0.3451	-3.2049
Cers6	0.4601	5.6192	2.7711	0.017	0.3451	-3.5545
Rbm5	0.2192	6.401	2.7709	0.017	0.3451	-3.6006
Gm15454	-2.278	-2.6302	-2.7706	0.017	0.3451	-3.1643
Tmem106b	0.7086	7.9741	2.7705	0.017	0.3451	-3.6338
Odf2	-0.2798	6.1872	-2.7703	0.017	0.3451	-3.6019
Fras1	1.8552	-1.2524	2.7702	0.017	0.3451	-3.022
Rap2b	0.3455	6.1519	2.7699	0.017	0.3451	-3.5946
Slc40a1	2.7824	-1.7671	2.7664	0.0171	0.3469	-3.1461
Tmem150a	0.7813	3.726	2.765	0.0172	0.347	-3.2705
Wdfy1	0.4063	5.3657	2.7645	0.0172	0.347	-3.5305
Shmt2	-0.3438	7.0876	-2.764	0.0172	0.347	-3.6427
Shisa4	0.6398	4.7238	2.7627	0.0172	0.347	-3.4364
Pcdhb17	0.875	1.8501	2.7625	0.0173	0.347	-3.0184
Gm9755	-0.9411	-0.41	-2.7623	0.0173	0.347	-2.9343
Psmb10	0.7968	5.4349	2.7613	0.0173	0.347	-3.545
Bphl	0.5675	3.7968	2.7608	0.0173	0.347	-3.2937
Kif26b	1.438	4.0803	2.7606	0.0173	0.347	-3.3008
Gm5300	2.0272	-0.5629	2.7588	0.0174	0.3478	-2.9912
Vgll4	0.4556	6.1913	2.7572	0.0174	0.3482	-3.6099
Ppp1r12c	-0.2505	5.6509	-2.7562	0.0175	0.3482	-3.5901
Cenpq	-0.2849	4.5202	-2.7561	0.0175	0.3482	-3.523
Rpl13a-ps1	-1.0309	-1.3647	-2.7545	0.0175	0.3488	-3.0019
Scmh1	-0.3637	4.4867	-2.7537	0.0175	0.3488	-3.467
Spp1	-0.6797	10.9872	-2.7534	0.0175	0.3488	-3.6892
Tbccd1	0.3588	4.0345	2.7521	0.0176	0.349	-3.3745
Hmga1	-0.7501	7.76	-2.7518	0.0176	0.349	-3.6734
Trim34a	1.5435	-0.9632	2.7507	0.0176	0.3492	-2.9921
Arid1a	0.3197	7.021	2.7503	0.0176	0.3492	-3.6576
Zfp213	0.3374	3.798	2.7492	0.0177	0.3495	-3.3655
Tnip1	0.4235	6.4787	2.7484	0.0177	0.3496	-3.6381
Ifngr2	0.4297	5.7987	2.7467	0.0178	0.3496	-3.5971
Btc	-1.9572	0.6925	-2.7454	0.0178	0.3496	-2.9651
Cenpa	-0.4366	6.4361	-2.7454	0.0178	0.3496	-3.6655
Myd88	0.3404	5.2752	2.7453	0.0178	0.3496	-3.578
Igsf8	-0.4362	7.0377	-2.7452	0.0178	0.3496	-3.6694
Zbtb16	0.8921	4.3748	2.7436	0.0179	0.3501	-3.3879
2210408I21Rik	0.6132	2.4931	2.7432	0.0179	0.3501	-3.1505
Tagap	-0.6862	0.9597	-2.7427	0.0179	0.3501	-2.9932
Zc3h11a	0.4141	4.0219	2.7398	0.018	0.3506	-3.3997
Gtf2h3	-0.3052	5.0797	-2.7398	0.018	0.3506	-3.5682

Hacd4	1.3988	0.0692	2.7395	0.018	0.3506	-2.9674
Rpl32l	-1.3719	0.402	-2.7394	0.018	0.3506	-2.9717
Arsb	0.4248	5.7559	2.7368	0.0181	0.3513	-3.6318
Col4a5	0.9767	8.0306	2.7365	0.0181	0.3513	-3.6938
Gm8253	-0.9795	0.0287	-2.7365	0.0181	0.3513	-2.9728
Pyroxd2	1.9006	0.9936	2.7355	0.0181	0.3515	-2.9763
Wdr38	-0.7618	2.2102	-2.7335	0.0182	0.3515	-3.1496
Atp6v1b2	0.297	7.1736	2.7332	0.0182	0.3515	-3.6896
Stat5a	0.948	2.9352	2.7328	0.0182	0.3515	-3.1994
AC129773.1	-1.0317	-0.5981	-2.7322	0.0182	0.3515	-2.9805
Sh3gl3	-0.9832	3.1142	-2.7321	0.0182	0.3515	-3.2866
Aox3	3.6879	-1.437	2.732	0.0183	0.3515	-3.1959
Sh2d5	-1.3334	4.7725	-2.7308	0.0183	0.3519	-3.5455
Gm23935	0.6373	9.4029	2.729	0.0184	0.3521	-3.7248
Hipk3	0.4194	6.2696	2.7284	0.0184	0.3521	-3.6728
Cpq	0.9593	4.9141	2.7277	0.0184	0.3521	-3.5094
Gm8623	-1.5396	-1.2313	-2.7276	0.0184	0.3521	-3.0227
H3f3a-ps1	0.6997	1.3823	2.7275	0.0184	0.3521	-3.0567
Ptx3	2.9093	1.4096	2.725	0.0185	0.3533	-3.0073
Gm44899	1.9748	-2.173	2.7241	0.0185	0.3533	-3.1765
Scarb2	0.4055	6.0111	2.7239	0.0185	0.3533	-3.6584
Itpr3	-0.4665	7.6255	-2.7222	0.0186	0.3534	-3.7186
Gm47814	0.8768	-0.3256	2.7219	0.0186	0.3534	-2.9931
Gm32849	-2.7185	-2.8857	-2.7214	0.0186	0.3534	-3.2034
Cryab	2.1593	4.6046	2.7212	0.0186	0.3534	-3.354
Lfng	0.8322	3.449	2.7205	0.0186	0.3534	-3.3143
Atp6v1d	0.3443	5.7795	2.7196	0.0187	0.3534	-3.6486
Gm13470	1.3532	0.2419	2.7194	0.0187	0.3534	-2.9981
Hsdl2	0.5108	3.7856	2.7171	0.0188	0.3546	-3.3884
Clic4	0.4329	8.0474	2.7159	0.0188	0.355	-3.7347
Reep3	0.339	6.713	2.715	0.0188	0.3552	-3.7139
Eef2k	0.4648	4.8295	2.714	0.0189	0.3554	-3.5595
Ammecr1l	0.2964	5.7131	2.7126	0.0189	0.356	-3.6644
Zfp934	0.6238	1.6879	2.712	0.0189	0.356	-3.1444
Ak3	1.0472	4.0592	2.7111	0.019	0.3562	-3.3893
Ciao2b	-0.256	5.2792	-2.7105	0.019	0.3562	-3.6403
AC134576.3	0.9686	1.5157	2.7099	0.019	0.3562	-3.0814
Nat2	0.7742	1.6817	2.7077	0.0191	0.3572	-3.1125
Il2rb	1.2275	0.9969	2.7065	0.0191	0.3573	-3.0522
Pmepa1	0.9704	4.4914	2.7064	0.0191	0.3573	-3.4603
2310001H17Rik	1.6394	-0.1318	2.7045	0.0192	0.3582	-3.0248
Gm47175	-0.837	0.5821	-2.7024	0.0193	0.3591	-3.0639
Gm11249	-1.0121	0.2392	-2.702	0.0193	0.3591	-3.029
Kifc5b	-0.4239	3.8496	-2.6998	0.0194	0.36	-3.5142
Stxbp3	0.3821	6.1705	2.6993	0.0194	0.36	-3.7184
Ube4a	0.3473	6.4607	2.6989	0.0194	0.36	-3.7293
Map2k3	-0.4502	6.9323	-2.698	0.0194	0.3601	-3.7536
Zfp36l1-ps	1.5399	-1.0031	2.6975	0.0195	0.3601	-3.0675
4930513N10Rik	0.8922	0.3741	2.6964	0.0195	0.3604	-3.0383
Cmss1	-0.3517	4.3647	-2.6955	0.0195	0.3607	-3.5889
Ldlrap1	-0.4995	4.8186	-2.6941	0.0196	0.3612	-3.6524
Glt8d2	2.8406	-0.6052	2.6927	0.0196	0.3618	-3.094

Meiob	-1.4201	-1.8343	-2.6877	0.0198	0.3636	-3.1022
Rbm4b	0.5088	3.5395	2.6863	0.0199	0.3636	-3.3958
AI182371	-1.7957	-2.6343	-2.686	0.0199	0.3636	-3.2115
Zfp597	0.4056	3.8184	2.6856	0.0199	0.3636	-3.4747
Gprc5a	-0.5597	5.6192	-2.6848	0.0199	0.3636	-3.7211
Tsen2	-0.4255	3.8716	-2.6842	0.0199	0.3636	-3.5065
Cep350	0.4179	5.788	2.684	0.0199	0.3636	-3.7136
Fuca1	0.4512	4.9832	2.6835	0.02	0.3636	-3.6184
Lsm3	-0.2923	5.7497	-2.683	0.02	0.3636	-3.7468
Msantd1	1.5172	-1.6758	2.6821	0.02	0.3636	-3.1369
Sptlc2	0.2919	6.5858	2.6812	0.02	0.3636	-3.7701
Dcxr	0.8864	2.7841	2.6804	0.0201	0.3636	-3.2593
Vps53	0.2973	5.4387	2.6795	0.0201	0.3636	-3.6908
Spire2	-0.4252	3.268	-2.6794	0.0201	0.3636	-3.4456
Rbmx2	-0.3656	4.4021	-2.6789	0.0201	0.3636	-3.6184
Farp2	0.3524	4.6987	2.6785	0.0201	0.3636	-3.619
Gsap	1.0403	3.744	2.6772	0.0202	0.3636	-3.3978
Parp4	0.3492	5.3219	2.6769	0.0202	0.3636	-3.6747
Calr3	-0.5685	2.56	-2.6764	0.0202	0.3636	-3.2954
Flcn	0.3479	5.4462	2.6758	0.0202	0.3636	-3.6911
Palld	0.4554	6.1731	2.6752	0.0203	0.3636	-3.7546
Echs1	-0.4222	6.24	-2.6749	0.0203	0.3636	-3.7729
Arhgef10	0.8896	4.0099	2.6743	0.0203	0.3636	-3.4611
Stat1	0.7362	3.7404	2.6741	0.0203	0.3636	-3.4671
Tgds	-0.3941	4.1246	-2.6741	0.0203	0.3636	-3.5619
Crnde	-0.7184	1.7075	-2.6733	0.0203	0.3636	-3.1871
Ifitm2	0.323	7.2705	2.6728	0.0204	0.3636	-3.8004
Gm31323	1.7516	-3.0609	2.6727	0.0204	0.3636	-3.3093
Pttg1	-0.4598	5.3328	-2.6727	0.0204	0.3636	-3.7347
Ctns	0.9462	4.6649	2.6713	0.0204	0.3636	-3.5464
Plxna1	0.4178	7.9567	2.6711	0.0204	0.3636	-3.811
Man2b2	1.123	3.6642	2.6711	0.0204	0.3636	-3.3944
Lgr6	-1.6238	2.9608	-2.67	0.0205	0.3636	-3.411
Nfil3	0.3144	6.3168	2.6695	0.0205	0.3636	-3.7845
Ruvbl2	-0.3069	6.9584	-2.6688	0.0205	0.3636	-3.8084
Dchs2	2.8186	-3.3072	2.6686	0.0205	0.3636	-3.3149
Nrp1	0.938	2.7357	2.6677	0.0206	0.3636	-3.3134
Gars	-0.2846	8.4389	-2.6675	0.0206	0.3636	-3.8291
Mrps35	-0.2634	5.7353	-2.6672	0.0206	0.3636	-3.7593
Cdca8	-0.2561	5.7631	-2.6657	0.0206	0.3636	-3.7886
Gse1	0.5684	5.3937	2.665	0.0207	0.3636	-3.6714
Piezo2	-1.7249	0.9447	-2.6637	0.0207	0.3636	-3.1386
Hlx	0.594	2.415	2.6627	0.0207	0.3636	-3.278
Adamts5	1.8822	-0.1683	2.6627	0.0207	0.3636	-3.0908
Cpeb3	0.9551	0.9808	2.662	0.0208	0.3636	-3.1089
F8	1.2547	0.8982	2.6619	0.0208	0.3636	-3.0931
Limk2	-0.2728	5.7359	-2.6617	0.0208	0.3636	-3.7542
Phyh	1.2886	3.9156	2.6616	0.0208	0.3636	-3.4523
Bbs12	-0.5715	4.4038	-2.6603	0.0208	0.3636	-3.6563
Aldh6a1	0.6237	4.3831	2.6602	0.0208	0.3636	-3.5411
Natd1	0.4194	4.8431	2.6602	0.0208	0.3636	-3.6322
Nup37	-0.3766	5.1395	-2.66	0.0208	0.3636	-3.7408

Anxa7	-0.294	6.7113	-2.6599	0.0209	0.3636	-3.8128
Pdgfrl	3.4929	1.122	2.6594	0.0209	0.3636	-3.087
Kbtbd7	0.6619	2.6852	2.656	0.021	0.364	-3.3136
Elk3	-0.3905	6.6099	-2.6557	0.021	0.364	-3.8192
Psme2	0.5863	7.0092	2.6553	0.021	0.364	-3.8254
Krt7	1.3921	0.5854	2.6552	0.021	0.364	-3.0997
Ccdc88b	1.3053	-1.5143	2.6549	0.021	0.364	-3.1523
Gm8129	-0.9308	-0.4172	-2.6546	0.0211	0.364	-3.0902
Gm16061	-1.2678	-1.6134	-2.6536	0.0211	0.364	-3.1208
Imp3	-0.3121	5.5908	-2.6531	0.0211	0.364	-3.7708
Cct7	-0.246	9.1018	-2.6528	0.0211	0.364	-3.8604
Yipf1	0.3556	4.0983	2.6525	0.0211	0.364	-3.555
AC133509.1	-0.5028	3.2662	-2.6524	0.0211	0.364	-3.4223
C230035I16Rik	0.8507	-0.3855	2.6523	0.0211	0.364	-3.0935
Gm26935	1.9229	-2.5106	2.6517	0.0212	0.3641	-3.3282
Zfand5	0.3907	7.3945	2.6505	0.0212	0.3645	-3.839
Ifnar1	0.2658	6.5293	2.6499	0.0212	0.3645	-3.82
Snrk	0.3564	4.3881	2.6494	0.0213	0.3645	-3.6019
Ets1	0.405	7.3583	2.6476	0.0213	0.3653	-3.8451
Sertad4	0.7404	4.168	2.6472	0.0213	0.3653	-3.5717
Gm28536	-1.6145	-2.7008	-2.6464	0.0214	0.3654	-3.2561
Arpc5	0.2239	7.6271	2.6445	0.0215	0.3663	-3.8563
Apbb2	0.3935	6.3558	2.6439	0.0215	0.3664	-3.8223
Dusp2	-0.9212	2.5583	-2.6431	0.0215	0.3665	-3.407
Gm5525	-0.8712	-0.3009	-2.6424	0.0215	0.3667	-3.1092
Nhsl2	2.7921	-1.4236	2.6413	0.0216	0.3667	-3.2484
Gm6436	-1.2886	-0.6102	-2.6408	0.0216	0.3667	-3.1141
Pcdhgb2	1.8311	-1.7346	2.6407	0.0216	0.3667	-3.2146
Gm10635	2.3528	-2.656	2.6402	0.0216	0.3667	-3.2991
Kdm7a	-0.379	6.1293	-2.6387	0.0217	0.3671	-3.8269
AI413582	-0.3577	4.6064	-2.6371	0.0217	0.3671	-3.6861
Banf1	-0.301	7.1471	-2.637	0.0218	0.3671	-3.8662
H2-T22	0.9305	1.9539	2.6356	0.0218	0.3671	-3.2406
Nr1h3	1.484	1.1732	2.6348	0.0218	0.3671	-3.1536
St14	1.6705	-0.9814	2.6346	0.0218	0.3671	-3.1506
Gm26910	1.1157	-0.7178	2.6345	0.0219	0.3671	-3.1291
Ttc17	0.335	4.7702	2.6344	0.0219	0.3671	-3.6825
Porcn	0.878	2.7482	2.6339	0.0219	0.3671	-3.3364
Gm5239	-0.9974	-0.5369	-2.6334	0.0219	0.3671	-3.1234
Mtap	-0.2387	7.773	-2.6334	0.0219	0.3671	-3.8811
Gm45053	-0.865	-0.467	-2.633	0.0219	0.3671	-3.1211
Slc6a2	3.8251	-0.2349	2.6323	0.0219	0.3671	-3.1806
Polm	-0.3495	4.6783	-2.6318	0.022	0.3671	-3.7174
Appl2	-0.4577	4.7948	-2.6316	0.022	0.3671	-3.6952
Fam214a	0.7653	3.8138	2.6305	0.022	0.3675	-3.4729
Exosc8	-0.3337	6.3675	-2.6298	0.022	0.3677	-3.8684
Rhob	0.5997	7.3051	2.6287	0.0221	0.3677	-3.8772
Gm13622	-1.4335	-1.4284	-2.6282	0.0221	0.3677	-3.1673
Dock8	-0.7695	3.5918	-2.6281	0.0221	0.3677	-3.5032
Plet1	-2.4723	0.7821	-2.6274	0.0221	0.3678	-3.1355
C1qtnf1	1.4211	4.9952	2.6262	0.0222	0.3681	-3.6563
Pld2	0.3774	4.8303	2.6258	0.0222	0.3681	-3.7142

Mpp6	-0.3352	6.9977	-2.6252	0.0222	0.3681	-3.8872
Peg13	0.6086	2.7733	2.6249	0.0222	0.3681	-3.3762
Hax1	-0.3154	6.4792	-2.6232	0.0223	0.369	-3.8727
AC133601.1	-1.6388	-2.2931	-2.6216	0.0224	0.3695	-3.1964
Plek2	-1.1121	0.2098	-2.6213	0.0224	0.3695	-3.1598
Ngef	-0.8968	1.9213	-2.6201	0.0224	0.3699	-3.3799
Gm6035	-1.7726	-2.851	-2.6194	0.0225	0.3699	-3.2839
Mafk	-0.316	4.6744	-2.6178	0.0225	0.3699	-3.7294
Map2k3os	-0.7023	1.9335	-2.6175	0.0226	0.3699	-3.3253
Nfkbiz	0.5433	6.0292	2.6174	0.0226	0.3699	-3.863
Sdhb	-0.3169	6.8589	-2.617	0.0226	0.3699	-3.8938
Lmna	-0.3677	9.6573	-2.6167	0.0226	0.3699	-3.9268
Gm16510	-1.2279	-2.6414	-2.6158	0.0226	0.3699	-3.2308
Ccdc14	-0.3506	3.4516	-2.6156	0.0226	0.3699	-3.5628
Manea	0.5493	4.3031	2.6155	0.0226	0.3699	-3.6478
Tipin	-0.3009	5.8012	-2.6133	0.0227	0.3704	-3.8794
Rabggtb	-0.2554	6.6725	-2.6128	0.0227	0.3704	-3.8992
Rpl35-ps1	-1.3278	-1.6368	-2.6122	0.0228	0.3704	-3.1794
5033406O09Rik	-1.5392	-2.2885	-2.6106	0.0228	0.3704	-3.2462
mt-Nd1	0.3536	11.1774	2.6105	0.0228	0.3704	-3.9427
AC132253.4	-1.165	-0.7722	-2.6104	0.0228	0.3704	-3.1543
Gm35190	2.1591	-3.5026	2.61	0.0229	0.3704	-3.3764
Sh3rf1	0.4754	4.3202	2.6098	0.0229	0.3704	-3.6592
Ercc4	0.4857	3.5818	2.6094	0.0229	0.3704	-3.5427
Uvssa	0.4538	4.0636	2.609	0.0229	0.3704	-3.6094
Ston2	1.4215	2.0653	2.6087	0.0229	0.3704	-3.3025
AC122119.2	-0.9362	0.2215	-2.6081	0.0229	0.3704	-3.1656
Mcur1	0.3408	5.485	2.6078	0.023	0.3704	-3.8199
Gm7206	-1.7548	-1.3404	-2.607	0.023	0.3704	-3.2122
Thap2	0.3871	5.0472	2.6067	0.023	0.3704	-3.7804
Gm16755	-0.9398	-0.4703	-2.6061	0.023	0.3704	-3.1606
Bco2	2.1276	-1.233	2.606	0.023	0.3704	-3.2238
Psap	0.3849	9.5805	2.6056	0.023	0.3704	-3.9444
Naalad2	1.446	-3.0808	2.603	0.0232	0.3706	-3.3611
Zeb2	0.4638	5.5229	2.6024	0.0232	0.3706	-3.847
Mtg1	-0.3651	3.6912	-2.6024	0.0232	0.3706	-3.6031
Lrrc28	0.4453	2.9787	2.6024	0.0232	0.3706	-3.484
Gfra1	2.607	-1.1163	2.6023	0.0232	0.3706	-3.2368
Klhl24	0.706	6.543	2.6011	0.0232	0.3706	-3.888
C77080	-0.3078	6.9988	-2.6006	0.0233	0.3706	-3.9235
Ehd1	-0.2542	8.0649	-2.6005	0.0233	0.3706	-3.944
Gm44261	-1.7063	-2.0463	-2.6002	0.0233	0.3706	-3.2536
Snx19	0.2963	5.5062	2.6002	0.0233	0.3706	-3.8392
Tubb2a	0.8088	4.457	2.599	0.0233	0.3711	-3.693
Masp1	4.1492	1.9431	2.5952	0.0235	0.3734	-3.1803
Ppp1r35	-0.3406	3.2641	-2.5942	0.0235	0.3735	-3.5484
Grhl1	0.9277	0.0417	2.5935	0.0236	0.3735	-3.1831
Unc5b	-0.7501	6.4705	-2.5927	0.0236	0.3735	-3.9303
Ppp1r15a	-0.2654	5.011	-2.5927	0.0236	0.3735	-3.8111
Gm2962	-1.4565	-1.9864	-2.5925	0.0236	0.3735	-3.2358
Slc9a5	-0.6451	3.7459	-2.592	0.0236	0.3735	-3.6485
Gm34934	-0.7389	0.9062	-2.5899	0.0237	0.3744	-3.2367

Medag	0.9606	4.8664	2.5897	0.0237	0.3744	-3.8076
1700037H04Rik	-0.3641	5.3808	-2.5891	0.0238	0.3745	-3.8697
Cavin2	2.875	2.3074	2.5886	0.0238	0.3745	-3.2543
Gstcd	-0.3107	5.4476	-2.5875	0.0238	0.3749	-3.896
Wdsub1	0.4087	3.7884	2.5863	0.0239	0.3753	-3.6191
Rab36	1.0012	0.242	2.5859	0.0239	0.3753	-3.1954
mt-Rnr2	0.4065	11.0045	2.5845	0.024	0.376	-3.9875
Lsm5	-0.2852	5.0219	-2.5835	0.024	0.376	-3.8577
Gm30339	1.5998	-2.4225	2.5834	0.024	0.376	-3.2676
Pgap3	0.4877	2.8265	2.5826	0.024	0.3762	-3.4639
2510016D11Rik	-1.0016	1.3261	-2.5815	0.0241	0.3764	-3.31
Gm17709	-1.0076	0.2654	-2.5812	0.0241	0.3764	-3.2346
Klc4	0.7402	3.9288	2.5809	0.0241	0.3764	-3.5897
Fam189a2	1.7919	-0.7701	2.5794	0.0242	0.3768	-3.2243
Klrg2	-0.853	4.3192	-2.5792	0.0242	0.3768	-3.7125
Sesn1	0.747	5.1887	2.5787	0.0242	0.3768	-3.8195
Gm14388	-1.3368	-1.8484	-2.5784	0.0242	0.3768	-3.2488
Vipas39	0.3652	5.0934	2.5774	0.0243	0.3771	-3.8224
Tppp3	1.372	-0.0604	2.5768	0.0243	0.3771	-3.2007
Map3k14	0.5441	5.0151	2.5764	0.0243	0.3771	-3.8173
Mir1291	1.2768	-2.0888	2.574	0.0244	0.3781	-3.2772
5930403N24Rik	1.2935	-2.0466	2.574	0.0244	0.3781	-3.29
Gm17241	-0.9512	-0.0454	-2.5734	0.0245	0.3782	-3.2079
Fzd8	0.5903	3.1205	2.5724	0.0245	0.3785	-3.5383
Muc1	2.0042	-1.0497	2.5701	0.0246	0.3795	-3.2609
D630033O11Rik	-2.0766	-2.28	-2.57	0.0246	0.3795	-3.3051
Pknox2	2.2792	-2.1143	2.5686	0.0247	0.3802	-3.3374
Dzip1	0.5972	3.916	2.5657	0.0248	0.3819	-3.6468
Cdc42bpa	0.2886	6.7617	2.5635	0.0249	0.3822	-3.9841
Lrrc15	1.5081	-2.4671	2.5635	0.0249	0.3822	-3.3456
Med16	0.3461	4.3247	2.5634	0.0249	0.3822	-3.7613
Atp2a3	-1.8773	-2.0181	-2.5633	0.0249	0.3822	-3.3291
Fn3k	1.9466	-1.2157	2.5616	0.025	0.3825	-3.244
Rasgef1b	1.358	-0.6587	2.5608	0.025	0.3825	-3.2361
Aamdc	0.4155	2.8058	2.56	0.0251	0.3825	-3.4741
Pdk2	0.8908	3.1129	2.5598	0.0251	0.3825	-3.4885
Mindy4	0.485	2.9245	2.5591	0.0251	0.3825	-3.5359
Efr3b	1.0879	-1.2101	2.559	0.0251	0.3825	-3.2292
Cldn15	1.534	-1.992	2.5589	0.0251	0.3825	-3.3049
Ntn4	1.856	0.2433	2.5589	0.0251	0.3825	-3.2262
Foxo3	0.4538	7.1896	2.558	0.0252	0.3825	-4.0015
Trim66	-1.0954	0.9893	-2.5573	0.0252	0.3825	-3.3564
Gm4459	-0.921	-1.2585	-2.5572	0.0252	0.3825	-3.2337
Gm7008	-0.6254	1.0459	-2.557	0.0252	0.3825	-3.3217
Mcoln1	0.408	5.0766	2.5566	0.0252	0.3825	-3.847
Rnf185	0.2638	5.5201	2.5556	0.0253	0.3826	-3.9151
Pik3cd	-0.852	0.6814	-2.5554	0.0253	0.3826	-3.2743
Ifi27	0.7115	4.7522	2.5543	0.0253	0.3828	-3.8102
Gm9512	-0.6524	1.2976	-2.5541	0.0253	0.3828	-3.3438
Clip4	1.5295	2.3322	2.5535	0.0254	0.3828	-3.3967
mt-Co1	0.316	12.3276	2.5532	0.0254	0.3828	-4.0463
Bmp2k	0.3425	4.8971	2.5518	0.0254	0.3831	-3.8744

Igtp	1.2954	2.4724	2.5514	0.0255	0.3831	-3.483
Gamt	0.7257	3.0902	2.5511	0.0255	0.3831	-3.5613
Timm29	0.2857	5.9364	2.5509	0.0255	0.3831	-3.9675
8430423G03Rik	-1.6202	-2.959	-2.5498	0.0255	0.3835	-3.3387
Ostm1	0.4886	4.7813	2.5491	0.0256	0.3835	-3.8184
Reep4	-0.3063	5.7295	-2.5487	0.0256	0.3835	-3.9764
Kif22	-0.3081	6.0146	-2.5484	0.0256	0.3835	-4.0034
Crebl2	0.8758	2.9559	2.5452	0.0258	0.3854	-3.5014
Xpr1	0.3199	6.1375	2.5445	0.0258	0.3854	-3.9932
Hist1h2bc	0.8427	4.2628	2.5439	0.0258	0.3854	-3.7288
Tk1	-0.4724	5.7133	-2.5433	0.0258	0.3854	-4.0166
Wdfy2	0.312	4.7045	2.5429	0.0259	0.3854	-3.8453
Flvcr1	0.2988	5.113	2.5425	0.0259	0.3854	-3.9166
Zfp646	0.2909	5.5799	2.5424	0.0259	0.3854	-3.9424
Ccsap	-0.5874	3.0041	-2.5419	0.0259	0.3855	-3.6478
Atp6ap1	0.2777	7.7832	2.54	0.026	0.3857	-4.0415
Gstm1	0.5715	7.0439	2.539	0.0261	0.3857	-4.0264
Gm12543	1.9014	-2.2285	2.5387	0.0261	0.3857	-3.3558
Gm7476	-0.992	0.3837	-2.5381	0.0261	0.3857	-3.2838
Noxred1	1.6208	-1.9252	2.5377	0.0261	0.3857	-3.3291
Cap1	-0.3061	8.5688	-2.5372	0.0261	0.3857	-4.0583
Anxa11	-0.3927	5.9669	-2.5365	0.0262	0.3857	-4.0045
Rbm3os	-0.9183	-1.343	-2.5364	0.0262	0.3857	-3.2694
Zfp456	0.8727	0.6814	2.5358	0.0262	0.3857	-3.2904
Gm37660	0.6798	0.4397	2.5356	0.0262	0.3857	-3.2814
Reps2	1.7984	0.4567	2.5353	0.0262	0.3857	-3.2596
Prkag2	0.3411	4.6012	2.5352	0.0262	0.3857	-3.8548
Spint1	-1.2783	0.9909	-2.5351	0.0262	0.3857	-3.3435
Gm43118	-1.2588	-1.4563	-2.5348	0.0263	0.3857	-3.2795
Tns1	0.8388	6.1075	2.5345	0.0263	0.3857	-3.9672
Ccdc18	-0.5375	3.7325	-2.5309	0.0264	0.388	-3.7589
Snhg9	-1.2342	-0.8975	-2.5284	0.0266	0.3885	-3.2686
Ablim1	2.11	0.8259	2.528	0.0266	0.3885	-3.2827
Rpl35a-ps5	-1.9604	-3.1432	-2.528	0.0266	0.3885	-3.4504
Acyp1	-0.4897	3.5186	-2.5276	0.0266	0.3885	-3.6798
Fam43a	0.8015	2.6621	2.5272	0.0266	0.3885	-3.5206
Zfp862-ps	0.4945	4.0381	2.5269	0.0266	0.3885	-3.7612
Rpl36-ps9	-1.0823	-2.5378	-2.5265	0.0267	0.3885	-3.3132
Gm44878	1.107	-1.606	2.5264	0.0267	0.3885	-3.3021
Kcnk2	-0.5147	3.4869	-2.524	0.0268	0.3896	-3.7558
Slc9a2	-1.1581	2.8883	-2.5237	0.0268	0.3896	-3.6398
Cep55	-0.4316	5.5587	-2.523	0.0268	0.3896	-4.0314
Crebrf	0.6318	4.6453	2.523	0.0268	0.3896	-3.8165
Btg3	-0.487	3.5617	-2.5207	0.0269	0.39	-3.7393
Zbtb1	0.2941	5.7692	2.5206	0.0269	0.39	-4.0044
Atg4a	0.4794	4.0179	2.5205	0.027	0.39	-3.7625
Bin1	-0.289	7.5333	-2.52	0.027	0.39	-4.0776
Cyp1b1	2.9138	2.5063	2.5196	0.027	0.39	-3.3839
Vsig10l	1.0828	-0.0245	2.5193	0.027	0.39	-3.2838
Gm8773	1.1766	-0.9187	2.5181	0.0271	0.39	-3.2847
Sesn3	0.8577	6.592	2.518	0.0271	0.39	-4.0379
Gm10988	-2.0397	-3.1928	-2.518	0.0271	0.39	-3.4599

Def8	0.2699	5.2985	2.517	0.0271	0.39	-3.9739
Gm45718	0.9635	-1.7108	2.5166	0.0271	0.39	-3.3159
Mapt	1.4166	-1.3298	2.5165	0.0272	0.39	-3.3085
Ctsl	-0.2506	10.1406	-2.5165	0.0272	0.39	-4.1045
Cd274	0.7601	1.0111	2.5153	0.0272	0.3902	-3.3663
Sestd1	-0.2761	7.023	-2.5153	0.0272	0.3902	-4.075
Ip6k3	1.2108	-0.0988	2.5127	0.0273	0.3915	-3.2902
Gm8960	-0.7969	0.6091	-2.5125	0.0274	0.3915	-3.3407
Lsm6	-0.3052	5.8635	-2.5121	0.0274	0.3915	-4.0537
Lpp	0.6168	6.4583	2.5116	0.0274	0.3916	-4.0605
Arl6ip4	-0.2695	5.1239	-2.5103	0.0275	0.3918	-3.9824
Tnfaip6	2.0127	0.4014	2.51	0.0275	0.3918	-3.3078
Ucp2	-0.3456	7.3698	-2.5093	0.0275	0.3918	-4.0908
Inpp4b	-0.8541	3.5859	-2.5092	0.0275	0.3918	-3.7725
Polr2e	-0.2833	6.6799	-2.5081	0.0276	0.3918	-4.0811
Vps41	0.3542	6.4951	2.5081	0.0276	0.3918	-4.0666
Zfp68	0.3392	5.5554	2.5077	0.0276	0.3918	-4.0036
Greb1l	0.9229	2.2035	2.5077	0.0276	0.3918	-3.5018
Ptgdr2	2.5317	-2.834	2.5064	0.0277	0.3924	-3.471
Tia1	0.241	6.1419	2.5051	0.0277	0.393	-4.0566
Abat	2.4792	-0.1729	2.5032	0.0278	0.3932	-3.3195
2610002M06Rik	0.3061	5.4094	2.5031	0.0278	0.3932	-4.0089
Gm16409	-1.4642	-1.6094	-2.5022	0.0279	0.3932	-3.3386
Gstp-ps	-0.5115	1.5359	-2.502	0.0279	0.3932	-3.4551
Wbp1l	0.3393	6.7522	2.5018	0.0279	0.3932	-4.0863
Fmn1	0.8383	4.1952	2.5012	0.0279	0.3932	-3.8086
Sdc2	0.6153	6.6155	2.5005	0.028	0.3932	-4.0816
Cenpj	0.404	4.4435	2.5003	0.028	0.3932	-3.8895
Aprt	-0.3879	7.1659	-2.5001	0.028	0.3932	-4.1077
Dnm3	-1.281	-1.7257	-2.4997	0.028	0.3932	-3.3309
Kif23	-0.3716	6.6459	-2.4996	0.028	0.3932	-4.1045
Galk1	-0.3536	6.693	-2.499	0.028	0.3932	-4.1026
Gm7589	-0.7916	0.0457	-2.4989	0.028	0.3932	-3.3325
Polk	-0.3873	4.2907	-2.4985	0.0281	0.3932	-3.8845
Lats2	0.5293	3.935	2.4972	0.0281	0.3936	-3.7937
Cnbd2	0.5511	2.2573	2.4969	0.0281	0.3936	-3.5304
Papss1	-0.2106	7.0062	-2.4966	0.0282	0.3936	-4.107
5430425K12Rik	-0.856	-0.7248	-2.4956	0.0282	0.3938	-3.3317
Gm9104	1.6649	-2.4487	2.4955	0.0282	0.3938	-3.4044
Castor1	-0.385	3.0199	-2.4945	0.0283	0.3942	-3.677
Cbwd1	-0.3337	4.2384	-2.4926	0.0284	0.395	-3.894
Tmem184c	0.2995	5.8796	2.4926	0.0284	0.395	-4.0629
Ctr9	0.2427	6.3494	2.4907	0.0285	0.3959	-4.0958
Ccnb2	-0.4044	6.3783	-2.4901	0.0285	0.3959	-4.1139
Atp2c1	0.2368	6.3246	2.4899	0.0285	0.3959	-4.0972
Fem1a	0.2665	5.7206	2.489	0.0285	0.396	-4.0693
Alpk1	0.9109	2.1723	2.4889	0.0286	0.396	-3.5018
Gm38375	1.4295	-2.726	2.4879	0.0286	0.3961	-3.4059
Traf6	0.3772	5.1195	2.4879	0.0286	0.3961	-3.9932
Gm12732	-1.0326	-0.4699	-2.4873	0.0286	0.3962	-3.329
C1rb	1.8699	-1.7095	2.4868	0.0287	0.3962	-3.3867
Plxdc1	2.3062	0.6504	2.4843	0.0288	0.3977	-3.3293

Zfp629	0.3373	3.761	2.4836	0.0288	0.3979	-3.7866
Ptpn14	0.2491	6.2182	2.4833	0.0289	0.3979	-4.0992
Tgfbr2	0.3499	6.0418	2.4815	0.0289	0.3988	-4.0915
Gm17096	1.8541	-2.5423	2.4794	0.0291	0.3995	-3.4218
Coq8b	0.525	3.4694	2.4792	0.0291	0.3995	-3.7327
Csf1	0.8909	7.0743	2.479	0.0291	0.3995	-4.1371
Mtmr11	-0.3918	4.6951	-2.4789	0.0291	0.3995	-3.9746
Kidins220	0.4484	6.245	2.4777	0.0291	0.4	-4.1056
Gm32914	1.9749	-2.6034	2.4759	0.0292	0.401	-3.5012
Slc41a3	1.116	0.3551	2.4752	0.0293	0.4012	-3.3672
Rai14	-0.5879	7.8596	-2.4732	0.0294	0.4021	-4.1637
Gcnt2	0.8255	4.726	2.4728	0.0294	0.4021	-4.0006
Gm12854	0.763	1.2745	2.4721	0.0295	0.4021	-3.4571
Gm13194	-2.0796	-2.1675	-2.4712	0.0295	0.4021	-3.4306
Gm17259	-1.0953	-0.533	-2.4712	0.0295	0.4021	-3.3495
Rbpj	-0.3817	6.806	-2.4707	0.0295	0.4021	-4.1524
Sema7a	-1.1559	4.6936	-2.4705	0.0295	0.4021	-4.0183
Zfp820	0.5788	1.6375	2.4698	0.0296	0.4021	-3.5116
Car13	0.6762	5.6757	2.469	0.0296	0.4021	-4.0784
Helz2	0.549	4.0265	2.4688	0.0296	0.4021	-3.8674
Eaf1	-0.2847	6.7831	-2.4685	0.0296	0.4021	-4.155
Meis2	0.4224	4.5465	2.4681	0.0297	0.4021	-3.9475
Atg12	0.2296	6.3151	2.468	0.0297	0.4021	-4.1323
Gm43598	1.8498	-2.4368	2.4679	0.0297	0.4021	-3.4925
AC116451.2	-1.8145	-2.1433	-2.4675	0.0297	0.4021	-3.4482
Top2a	-0.3267	9.3103	-2.4652	0.0298	0.4035	-4.1933
Gm11914	-0.7823	0.786	-2.4646	0.0299	0.4036	-3.455
Gm6365	-2.0625	-3.0617	-2.4635	0.0299	0.4037	-3.5136
Map1lc3b	0.307	7.1849	2.4633	0.0299	0.4037	-4.1624
Ptms	0.3433	8.3203	2.4622	0.03	0.4037	-4.1846
Itga5	-0.4146	7.8439	-2.462	0.03	0.4037	-4.1819
Gm6210	-0.7748	1.6141	-2.4619	0.03	0.4037	-3.5186
Paox	-0.656	3.2066	-2.4614	0.03	0.4037	-3.7553
Tsku	0.5401	4.7477	2.461	0.0301	0.4037	-4.035
Dpep2	-2.2235	-2.2253	-2.4608	0.0301	0.4037	-3.4985
M1ap	-0.8	0.1477	-2.4606	0.0301	0.4037	-3.4003
Lcat	0.839	-0.0871	2.4601	0.0301	0.4038	-3.3899
Bcl2l2	-0.4168	4.5836	-2.4594	0.0301	0.404	-3.9957
Gm42517	1.3162	-0.8217	2.4567	0.0303	0.4054	-3.3678
H3f3a	0.2331	7.6204	2.4566	0.0303	0.4054	-4.1838
Gm9517	1.6262	-2.8031	2.4559	0.0303	0.4056	-3.5056
Aatk	-1.7117	-0.6895	-2.4546	0.0304	0.4058	-3.371
Epc1	0.3697	5.6342	2.4544	0.0304	0.4058	-4.0987
Gm3219	-1.9926	-2.1679	-2.4542	0.0304	0.4058	-3.4408
Pacs2	0.4518	5.3203	2.4532	0.0305	0.4058	-4.052
Irx1	0.3672	6.6663	2.4529	0.0305	0.4058	-4.1691
Rps15a-ps5	-0.8678	-0.2864	-2.4529	0.0305	0.4058	-3.3927
Gm6634	-0.6758	2.163	-2.4527	0.0305	0.4058	-3.6394
Gm48357	1.313	-2.113	2.4507	0.0306	0.4066	-3.4141
Slc4a4	1.0629	4.4315	2.4507	0.0306	0.4066	-3.9208
Zfp513	0.2912	4.7386	2.45	0.0307	0.4066	-4.0162
Slc25a44	0.3071	5.6867	2.4498	0.0307	0.4066	-4.1224

Rps7-ps2	-1.471	-1.7936	-2.4482	0.0308	0.4066	-3.3912
Tpi-rs4	-0.8973	-0.0961	-2.4482	0.0308	0.4066	-3.4025
Zfp24	0.2947	5.9799	2.4478	0.0308	0.4066	-4.1548
Ldlrad4	0.6487	2.8779	2.4473	0.0308	0.4066	-3.6854
Gm2534	-1.6357	-1.7856	-2.4469	0.0308	0.4066	-3.4127
Chd9	0.4487	5.5791	2.4467	0.0308	0.4066	-4.1185
Wdr7	0.6068	5.15	2.4466	0.0309	0.4066	-4.0511
Cdc25b	-0.4705	6.1197	-2.4462	0.0309	0.4066	-4.1808
Stxbp4	0.25	4.7485	2.4461	0.0309	0.4066	-4.0212
Hpx	1.6823	-1.8496	2.4441	0.031	0.4074	-3.4555
Iffo2	-0.4563	5.6066	-2.4435	0.031	0.4074	-4.1416
Ifit3b	2.7981	-1.6699	2.4433	0.031	0.4074	-3.4302
Gm44986	-1.6078	-2.785	-2.4429	0.0311	0.4074	-3.5206
Tut7	0.3273	6.8384	2.4424	0.0311	0.4074	-4.1939
Neurl2	0.5515	2.0301	2.4424	0.0311	0.4074	-3.564
Ift122	0.3777	4.4465	2.4421	0.0311	0.4074	-3.9577
Arhgap45	-1.5446	0.6366	-2.4415	0.0311	0.4075	-3.4371
Paip2	-0.2563	7.391	-2.4403	0.0312	0.4075	-4.2094
Arhgef18	0.415	5.2721	2.4397	0.0312	0.4075	-4.0844
Wdr92	-0.2998	4.9305	-2.4393	0.0313	0.4075	-4.0795
Kirrel3	1.9791	-1.5553	2.4392	0.0313	0.4075	-3.4321
Tert	-0.4602	2.8332	-2.4389	0.0313	0.4075	-3.7329
Trio	0.3418	6.5617	2.4388	0.0313	0.4075	-4.1938
Zfp157	0.368	3.736	2.4387	0.0313	0.4075	-3.8545
Lockd	-0.5511	2.6592	-2.4378	0.0314	0.4075	-3.7196
Ermp1	-0.4542	6.8129	-2.4377	0.0314	0.4075	-4.2059
Gm19619	-1.2129	-3.0516	-2.4371	0.0314	0.4075	-3.4662
Caprin1	0.216	9.2098	2.437	0.0314	0.4075	-4.2389
Dlat	-0.2412	6.6184	-2.4366	0.0314	0.4075	-4.2044
Ankrd27	-0.2421	5.4441	-2.4353	0.0315	0.408	-4.147
Cenpv	-0.414	4.0524	-2.4351	0.0315	0.408	-3.9525
Tcirg1	0.3536	5.9549	2.4346	0.0315	0.4081	-4.1661
Gm9616	-0.4536	2.7978	-2.4339	0.0316	0.4082	-3.7348
Tspan14	-0.291	6.0515	-2.4332	0.0316	0.4082	-4.1828
6330403L08Rik	0.3838	2.7554	2.4332	0.0316	0.4082	-3.7092
Gm11714	-1.9173	-1.9536	-2.4326	0.0317	0.4083	-3.4357
Acox3	0.6726	3.877	2.432	0.0317	0.4085	-3.858
Rnps1	-0.2534	5.7215	-2.4316	0.0317	0.4085	-4.1793
Fth-ps2	-1.261	-0.6319	-2.4303	0.0318	0.4091	-3.403
N4bp3	-0.4142	3.5496	-2.4295	0.0318	0.4091	-3.939
Cpt1a	1.1603	4.4258	2.4292	0.0318	0.4091	-3.8997
Szt2	0.3809	4.7019	2.4291	0.0319	0.4091	-4.0213
Rtl5	1.968	-0.0894	2.4276	0.0319	0.4099	-3.4075
Zeb2os	1.8871	-0.4685	2.4271	0.032	0.4099	-3.4072
C920021L13Rik	1.8055	-2.1276	2.4259	0.032	0.4105	-3.4981
Mtmr4	0.2521	6.3888	2.4256	0.0321	0.4105	-4.211
Tpd52-ps	1.7421	-3.0929	2.4249	0.0321	0.4105	-3.5127
Gm17893	-2.2737	-3.3399	-2.4246	0.0321	0.4105	-3.5513
Lpin1	-0.5224	5.4541	-2.4239	0.0322	0.4105	-4.1351
Gm43661	-0.8406	-0.2885	-2.4239	0.0322	0.4105	-3.4326
Gm14026	-0.9603	-1.5833	-2.423	0.0322	0.4108	-3.429
2810408I11Rik	-0.4374	2.3857	-2.4222	0.0323	0.4108	-3.7646

Cacul1	0.2835	6.3062	2.4217	0.0323	0.4108	-4.2116
Vps11	0.2598	6.4512	2.4215	0.0323	0.4108	-4.2149
Catsperd	1.4048	-2.0706	2.4215	0.0323	0.4108	-3.4552
Tgif1	-0.4445	6.7248	-2.42	0.0324	0.4115	-4.2326
Fxr2	-0.2616	7.1584	-2.4198	0.0324	0.4115	-4.2425
Nkap	-0.2476	4.9149	-2.4194	0.0324	0.4115	-4.1113
Myorg	0.8684	3.0963	2.4187	0.0325	0.4117	-3.7559
Ccdc102a	0.3832	3.7207	2.4181	0.0325	0.4117	-3.8928
Tom1	0.6235	3.4764	2.4176	0.0325	0.4117	-3.8215
Cdc25c	-0.4575	5.0444	-2.4166	0.0326	0.4117	-4.1679
Vamp1	0.5265	2.4267	2.4165	0.0326	0.4117	-3.6757
Slc22a5	0.3976	2.7939	2.4163	0.0326	0.4117	-3.7446
Gm49338	0.505	3.2051	2.4162	0.0326	0.4117	-3.8037
Zfp780b	0.3442	3.2876	2.414	0.0327	0.4129	-3.837
Smurf1	-0.2582	6.5328	-2.4138	0.0328	0.4129	-4.2409
Spata9	1.7704	-2.4297	2.4131	0.0328	0.4132	-3.4871
Cdh24	0.5946	2.1037	2.4108	0.0329	0.4142	-3.6555
Notch2	0.5149	7.5667	2.4106	0.0329	0.4142	-4.2597
Pnrc1	0.3112	7.0567	2.4105	0.033	0.4142	-4.2524
A230103J11Rik	-1.0273	-1.3625	-2.4093	0.033	0.4145	-3.4347
Gm49066	-1.3741	-1.1646	-2.4089	0.0331	0.4145	-3.4364
Tln2	0.7742	4.7235	2.4088	0.0331	0.4145	-4.047
Bpifb4	-1.1696	1.1908	-2.4083	0.0331	0.4145	-3.5351
Gpatch2l	0.3011	4.6755	2.4082	0.0331	0.4145	-4.077
Mss51	-0.909	0.0307	-2.4071	0.0332	0.4147	-3.477
Bend5	-1.5254	-2.5983	-2.4071	0.0332	0.4147	-3.4777
Dlg4	-0.4369	5.2513	-2.4067	0.0332	0.4147	-4.1669
Serpinb6b	2.865	-0.3139	2.4051	0.0333	0.4156	-3.4448
Fbxo36	0.6391	2.3636	2.4046	0.0333	0.4156	-3.6696
Lgmn	0.3455	7.0232	2.4039	0.0334	0.4156	-4.2633
Inpp5j	2.2193	-2.5965	2.4034	0.0334	0.4156	-3.5565
Sms-ps	-0.5577	1.5141	-2.4034	0.0334	0.4156	-3.6391
Gm13292	-1.0425	-0.9633	-2.4028	0.0334	0.4156	-3.4417
Sh3rf3	2.0856	-1.4812	2.4026	0.0334	0.4156	-3.4557
Sh3bp2	0.6184	5.4331	2.4017	0.0335	0.4161	-4.1561
Stap2	1.1158	2.547	2.4012	0.0335	0.4161	-3.6605
Erich2os	-0.8327	-0.679	-2.4009	0.0335	0.4161	-3.4548
Gm26797	1.7207	-1.9928	2.3998	0.0336	0.4163	-3.4964
Gpr135	0.6872	1.2032	2.3997	0.0336	0.4163	-3.5416
Slc4a8	-0.5711	2.9013	-2.3995	0.0336	0.4163	-3.8636
Saxo2	-1.016	-0.9979	-2.3986	0.0337	0.4163	-3.4455
Fbxo10	0.5303	3.9762	2.3981	0.0337	0.4163	-3.9495
Zfp84	0.3747	3.5746	2.3981	0.0337	0.4163	-3.9121
Fhod3	0.9243	2.6139	2.3978	0.0337	0.4163	-3.7223
Gm35330	2.4197	-1.6839	2.3961	0.0338	0.4172	-3.4899
Ptpn11	0.2116	7.5952	2.3959	0.0338	0.4172	-4.2922
Tm4sf4	-1.4844	-3.2785	-2.3951	0.0339	0.4174	-3.5699
Nol4l	-0.7481	4.1465	-2.3947	0.0339	0.4174	-4.0012
Acsl1	0.5403	4.8351	2.3939	0.034	0.4174	-4.1528
Twsg1	0.2957	6.9736	2.3938	0.034	0.4174	-4.2823
Rpl36a-ps2	-0.4762	1.8511	-2.3936	0.034	0.4174	-3.6591
04-sept	2.0324	-1.9095	2.3917	0.0341	0.4186	-3.4963

Gm43268	1.0142	-0.8811	2.3892	0.0343	0.4202	-3.4583
Cenpw	-0.3166	3.6999	-2.3885	0.0343	0.4204	-4.0407
Kif1b	0.3557	6.6632	2.3881	0.0343	0.4204	-4.2828
Tex30	-0.2492	4.876	-2.3871	0.0344	0.4209	-4.1903
Lctl	2.7052	-0.63	2.3864	0.0344	0.4209	-3.4968
Dstyk	0.4973	5.0536	2.3856	0.0345	0.4209	-4.1373
Daam2	4.433	-0.8124	2.3851	0.0345	0.4209	-3.5417
Arsk	0.3477	3.4586	2.3848	0.0345	0.4209	-3.8984
Apc2	1.6461	-1.5484	2.3847	0.0345	0.4209	-3.4992
Sh3bp4	0.5368	5.4193	2.3844	0.0346	0.4209	-4.2141
Ppfibp1	-0.2121	8.4499	-2.3843	0.0346	0.4209	-4.3228
Gm5822	-0.5901	1.611	-2.3839	0.0346	0.4209	-3.6319
AC126944.1	-1.4762	-2.7804	-2.3833	0.0346	0.4209	-3.5593
Aqp8	-2.06	-1.0029	-2.3832	0.0346	0.4209	-3.4678
Zscan26	0.239	5.3641	2.3825	0.0347	0.4211	-4.2096
Gm6288	1.919	-1.6425	2.3812	0.0348	0.4218	-3.5035
Ppp1r2-ps4	-2.1241	-3.0143	-2.3807	0.0348	0.4219	-3.5796
Zfp809	0.449	3.3818	2.3803	0.0348	0.4219	-3.9069
Gm12100	2.0416	-3.4047	2.3797	0.0349	0.4221	-3.6026
Dusp10	0.5844	3.8821	2.3792	0.0349	0.4222	-3.9873
Sufu	0.2979	5.6214	2.3785	0.0349	0.4224	-4.2409
Ap5b1	0.6058	1.9165	2.3771	0.035	0.4228	-3.6846
Gm47205	1.1192	-0.9967	2.3763	0.0351	0.4228	-3.4784
Tedc1	-0.3052	4.0146	-2.3762	0.0351	0.4228	-4.0855
Coro1a	1.2078	-0.6214	2.3746	0.0352	0.4228	-3.4774
9430062P05Rik	-1.1476	-1.1688	-2.3745	0.0352	0.4228	-3.4836
B130046B21Rik	-1.0025	-1.2613	-2.3738	0.0352	0.4228	-3.4789
Tpk1	0.5429	1.7196	2.3734	0.0353	0.4228	-3.645
Phldb3	0.7333	1.4439	2.3733	0.0353	0.4228	-3.6224
Kdm5a	0.2022	6.9557	2.3728	0.0353	0.4228	-4.3174
1110003F10Rik	-1.516	-2.7734	-2.3728	0.0353	0.4228	-3.5418
Atp6v0b	0.3193	6.3682	2.3724	0.0353	0.4228	-4.2955
Adamts17	2.3196	-2.1441	2.3724	0.0353	0.4228	-3.5669
Gm10335	-1.5938	-2.2846	-2.3722	0.0353	0.4228	-3.5345
2900076A07Rik	-0.5537	1.0928	-2.3719	0.0354	0.4228	-3.6042
Tpcn1	-0.6193	6.9768	-2.3716	0.0354	0.4228	-4.3216
Ncapd3	-0.283	5.8884	-2.3707	0.0354	0.4228	-4.3019
Fos	1.5228	1.51	2.3699	0.0355	0.4228	-3.6385
Dcaf6	0.3685	6.0306	2.3693	0.0355	0.4228	-4.278
Papolg	0.3034	4.6069	2.3693	0.0355	0.4228	-4.1211
Fetub	-0.8979	2.9948	-2.3691	0.0355	0.4228	-3.8697
Mrtfb	0.3497	5.2756	2.369	0.0355	0.4228	-4.2141
Gm10509	0.972	-0.2761	2.3686	0.0356	0.4228	-3.4991
Sri	0.2448	6.4547	2.3684	0.0356	0.4228	-4.3121
9830144P21Rik	-1.4054	-2.1596	-2.3683	0.0356	0.4228	-3.5581
Dner	-0.9232	-1.4453	-2.3681	0.0356	0.4228	-3.4866
Gm48194	1.4993	-1.5489	2.3681	0.0356	0.4228	-3.5197
Zfyve1	0.5225	5.5371	2.3655	0.0358	0.4241	-4.2174
Pold4	-0.6711	4.4545	-2.3651	0.0358	0.4241	-4.1091
Layn	-0.387	5.2573	-2.3649	0.0358	0.4241	-4.2485
Mmp1a	-2.1875	-2.1062	-2.3648	0.0358	0.4241	-3.5276
Pmp22	0.5583	6.0502	2.3642	0.0359	0.4243	-4.2832

Gm6594	-0.6938	0.1101	-2.3619	0.036	0.4254	-3.5485
Hacd3	0.2051	6.3677	2.3617	0.036	0.4254	-4.3219
Nqo2	-0.3839	5.1142	-2.3616	0.036	0.4254	-4.2513
Gm48338	-1.5949	-2.5837	-2.3608	0.0361	0.4257	-3.5545
AC127331.2	-0.5558	1.0998	-2.3592	0.0362	0.4267	-3.6283
1810062G17Rik	-1.6099	-2.4409	-2.3583	0.0362	0.427	-3.5673
Xdh	0.5506	7.6939	2.3581	0.0363	0.427	-4.3562
Gm9670	1.4765	-2.8877	2.3576	0.0363	0.4271	-3.6063
Gm16477	-1.1749	-2.2148	-2.3565	0.0364	0.4273	-3.5279
Gm5497	-1.4705	-2.2833	-2.3564	0.0364	0.4273	-3.5527
Fam193a	0.3576	5.6189	2.3562	0.0364	0.4273	-4.2759
Arhgap21	0.2512	6.6035	2.3557	0.0364	0.4273	-4.3399
Thnsl2	2.0467	-2.6458	2.3549	0.0365	0.4274	-3.6202
Gm5652	-1.1039	-1.0414	-2.3548	0.0365	0.4274	-3.5052
Gm7600	-1.166	-0.5997	-2.3507	0.0367	0.4304	-3.5148
Gm6768	1.1601	-0.6698	2.3503	0.0368	0.4304	-3.5106
Ctdsp2	0.371	6.8937	2.3493	0.0368	0.4305	-4.3524
Psme1	0.4311	6.7085	2.349	0.0369	0.4305	-4.3511
Mocs1	0.5353	4.3828	2.3487	0.0369	0.4305	-4.1073
Gbp6	1.5842	-2.3849	2.3483	0.0369	0.4305	-3.5604
S100a6	-0.2543	11.2381	-2.3481	0.0369	0.4305	-4.4003
Zswim8	0.331	6.8949	2.3478	0.0369	0.4305	-4.3562
Exosc7	-0.3757	5.0281	-2.3476	0.037	0.4305	-4.2701
Lrrc73	0.7049	0.9778	2.3462	0.037	0.4313	-3.6335
D030056L22Rik	-0.2538	5.1564	-2.3447	0.0371	0.4321	-4.2848
Pcdhb7	1.4695	-0.5355	2.3442	0.0372	0.4321	-3.5185
Nos2	1.4871	3.166	2.3439	0.0372	0.4321	-3.9679
Kctd21	0.5504	2.4504	2.3435	0.0372	0.4321	-3.7901
Ripor3	-1.7044	-0.4075	-2.3433	0.0372	0.4321	-3.5273
Gm44658	1.5064	-2.2668	2.3428	0.0373	0.4322	-3.5543
4632415L05Rik	0.459	5.1402	2.3418	0.0373	0.4324	-4.24
Gm11574	-1.7314	-2.8994	-2.3416	0.0373	0.4324	-3.6206
Tbc1d4	0.5377	6.0455	2.3414	0.0374	0.4324	-4.3301
Rtp4	2.2561	0.3915	2.3392	0.0375	0.433	-3.553
Gm43984	1.8814	-3.221	2.3387	0.0375	0.433	-3.6369
Gm2412	-1.672	-2.7189	-2.3382	0.0376	0.433	-3.5888
Zfp14	0.9783	-0.8	2.3377	0.0376	0.433	-3.5269
Gm35066	-0.9708	-0.8657	-2.3376	0.0376	0.433	-3.5295
Apon	1.3602	-2.2889	2.3376	0.0376	0.433	-3.5607
Plekhf1	1.321	3.6034	2.3367	0.0377	0.433	-3.8991
Exoc3	0.1914	5.7579	2.3365	0.0377	0.433	-4.3252
Laptm4a	0.3565	8.5773	2.3365	0.0377	0.433	-4.4046
Zfp282	0.3727	4.919	2.3359	0.0377	0.433	-4.2285
Slc35f6	0.3446	4.9377	2.3357	0.0378	0.433	-4.2323
M6pr-ps	-1.1054	-2.6405	-2.3354	0.0378	0.433	-3.5583
Gm42609	-0.9533	-0.5354	-2.3348	0.0378	0.433	-3.567
Tigd2	-0.4231	5.1337	-2.3345	0.0378	0.433	-4.2601
Mrps10	-0.2573	5.3044	-2.334	0.0379	0.433	-4.306
Gm16638	1.6786	-0.6651	2.3336	0.0379	0.433	-3.5323
Golga7b	1.5496	-1.9326	2.3335	0.0379	0.433	-3.5534
Dnah2	-1.8393	-1.8734	-2.3334	0.0379	0.433	-3.5471
Smox	-0.7636	5.0278	-2.3326	0.038	0.433	-4.2515

Wnt10a	1.3321	5.3382	2.3325	0.038	0.433	-4.2893
Gpr179	1.5234	-1.2729	2.3321	0.038	0.433	-3.5425
Gm5432	0.942	-0.1469	2.3309	0.0381	0.433	-3.5721
Gpx8	0.3349	5.6448	2.3309	0.0381	0.433	-4.3276
Gm16861	0.7461	-0.3546	2.3306	0.0381	0.433	-3.5481
Ddx6	0.2054	8.2071	2.3299	0.0382	0.433	-4.413
Resf1	0.3002	7.3812	2.3299	0.0382	0.433	-4.3979
Mmd	-0.4487	6.385	-2.3298	0.0382	0.433	-4.3757
Gm45592	1.1802	-2.0701	2.3295	0.0382	0.433	-3.5713
Tnfsf10	2.5951	-2.5003	2.3291	0.0382	0.433	-3.6214
Hp	3.2138	3.1642	2.329	0.0382	0.433	-3.7774
Nup88	-0.2154	7.0687	-2.3288	0.0382	0.433	-4.3986
2900042K21Rik	-1.6103	-2.4079	-2.3287	0.0382	0.433	-3.57
Pfkfb2	0.6308	2.3078	2.3285	0.0382	0.433	-3.7855
2700081O15Rik	0.4668	5.5967	2.3275	0.0383	0.4334	-4.3168
Plekhh2	0.5077	5.303	2.3272	0.0383	0.4334	-4.2835
Gm46336	-1.0405	-1.8093	-2.3257	0.0384	0.4343	-3.5479
3300005D01Rik	-0.3065	4.9588	-2.3252	0.0385	0.4345	-4.2852
Gm37494	0.4217	3.7416	2.3241	0.0386	0.435	-4.0449
Gm43328	-1.0182	-1.1261	-2.3237	0.0386	0.435	-3.5447
Mbd1	0.366	6.2502	2.3234	0.0386	0.435	-4.3729
Vegfd	1.1858	3.2367	2.3204	0.0388	0.4367	-3.9961
Rtkn2	-0.5682	2.6831	-2.3203	0.0388	0.4367	-3.9665
B9d1os	1.0915	-1.5825	2.3199	0.0389	0.4367	-3.5534
Clec2l	1.3707	-1.7058	2.3199	0.0389	0.4367	-3.5715
Gm29346	1.2247	-3.1766	2.3181	0.039	0.4378	-3.6129
Nup107	-0.2859	6.3979	-2.3173	0.039	0.4378	-4.4102
Tesmin	1.1965	-1.5441	2.3167	0.0391	0.4378	-3.5651
1700020I14Rik	0.3307	6.0123	2.3167	0.0391	0.4378	-4.3704
Poc1a	-0.2763	3.755	-2.3164	0.0391	0.4378	-4.1429
Apobec1	0.6626	2.4768	2.3163	0.0391	0.4378	-3.8739
1110025M09Rik	0.9378	-0.9867	2.3154	0.0392	0.4382	-3.5554
Rnf34	0.2543	4.9365	2.3141	0.0393	0.439	-4.28
Ivl	-1.4467	-0.6581	-2.3137	0.0393	0.439	-3.5818
E330032C10Rik	-1.8459	-1.9308	-2.3132	0.0393	0.4391	-3.5657
Rtn4r	1.9754	-1.3729	2.3126	0.0394	0.4393	-3.5854
Gm43011	-0.9841	-1.6438	-2.3108	0.0395	0.44	-3.5622
Gm13456	-0.3429	4.5404	-2.3108	0.0395	0.44	-4.2188
Zfp558	0.9501	-0.0314	2.3103	0.0395	0.44	-3.5795
Schip1	-0.8767	1.3742	-2.31	0.0396	0.44	-3.759
Wwc2	0.3369	6.2205	2.3098	0.0396	0.44	-4.4015
Gm16238	-1.0894	0.3217	-2.3095	0.0396	0.44	-3.6213
Gtf2e2	-0.2578	6.3039	-2.3085	0.0397	0.4405	-4.4181
Gm18085	-1.6515	-4.0929	-2.3078	0.0397	0.4405	-3.6808
Pxylp1	0.65	3.5932	2.3073	0.0397	0.4405	-4.0519
Sh3pxd2b	0.5034	7.6224	2.3071	0.0398	0.4405	-4.445
Dennd6b	-0.5922	2.3121	-2.307	0.0398	0.4405	-3.8546
4933412E12Rik	0.636	2.7591	2.3068	0.0398	0.4405	-3.9051
Stard3nl	-0.232	5.3612	-2.3063	0.0398	0.4406	-4.3494
Fcgr4	1.8782	-0.996	2.3049	0.0399	0.4413	-3.5737
Sh3tc2	0.5819	5.4437	2.3046	0.0399	0.4413	-4.3195
Sigirr	-0.9974	3.194	-2.3043	0.04	0.4413	-4.0119

Gm16279	1.4112	-1.929	2.3038	0.04	0.4414	-3.6171
Rpl5-ps1	-1.516	-1.4969	-2.3036	0.04	0.4414	-3.5764
Tsc1	0.2637	5.6457	2.3031	0.04	0.4415	-4.366
Trim62	1.1107	0.1431	2.3023	0.0401	0.4416	-3.59
Krt16	1.6991	-0.6495	2.3019	0.0401	0.4416	-3.5729
Eml6	0.5774	2.3065	2.3016	0.0402	0.4416	-3.8449
Gm6142	-1.1545	-1.0469	-2.3014	0.0402	0.4416	-3.5736
Pdia6	-0.3199	9.0488	-2.3009	0.0402	0.4416	-4.472
Kat6b	0.4377	5.0736	2.3006	0.0402	0.4416	-4.29
Gm11476	1.4969	-2.3197	2.3005	0.0402	0.4416	-3.5977
mt-Tt	0.7537	0.1862	2.3002	0.0403	0.4416	-3.6186
Acbd4	-0.4009	4.3574	-2.2997	0.0403	0.4416	-4.2208
Rtn4rl1	1.2391	-1.9347	2.2986	0.0404	0.4418	-3.5869
Sms	-0.3144	6.7634	-2.2985	0.0404	0.4418	-4.4476
Snrnp27	-0.2631	5.8757	-2.298	0.0404	0.4418	-4.4086
4930556M19Rik	-0.8384	1.6852	-2.2979	0.0404	0.4418	-3.7441
Chst2	0.6857	4.4586	2.2978	0.0404	0.4418	-4.2665
Mlst8	0.3002	4.4313	2.2969	0.0405	0.4419	-4.2487
Ddr1	0.7375	6.224	2.2969	0.0405	0.4419	-4.4005
Zfp52	-0.4935	4.8393	-2.2957	0.0406	0.4426	-4.3144
Nes	-0.5138	5.1213	-2.2933	0.0408	0.4443	-4.3803
Coro1b	0.2962	6.7817	2.2925	0.0408	0.4445	-4.448
Mycbp2	0.2574	6.4826	2.2921	0.0409	0.4445	-4.4415
Gm6767	-0.6784	1.5479	-2.2917	0.0409	0.4445	-3.7705
Pea15a	-0.322	6.5418	-2.2916	0.0409	0.4445	-4.4497
Chst11	0.4139	5.4206	2.2908	0.041	0.4446	-4.3758
Cd300lb	-1.2785	2.0183	-2.2907	0.041	0.4446	-3.8373
Gm15998	0.969	-0.014	2.2903	0.041	0.4447	-3.615
Snx11	0.3433	3.7162	2.289	0.0411	0.4447	-4.1158
Cd38	-0.8084	2.8366	-2.2888	0.0411	0.4447	-3.9223
Gm12435	-0.6867	-0.7464	-2.2886	0.0411	0.4447	-3.6098
Gm46378	-2.0074	-2.3523	-2.2883	0.0411	0.4447	-3.632
Kctd1	0.4141	2.8378	2.2882	0.0411	0.4447	-3.9462
Yjefn3	0.8233	-0.3024	2.2881	0.0411	0.4447	-3.6018
Cd99l2	0.3024	6.1781	2.2876	0.0412	0.4447	-4.4319
Bbs4	-0.3592	2.7525	-2.2875	0.0412	0.4447	-3.9735
Pbxip1	0.7957	5.7462	2.2861	0.0413	0.4451	-4.3749
Wnt9a	1.4794	0.7819	2.286	0.0413	0.4451	-3.6583
Zfp316	0.4474	3.3949	2.2856	0.0413	0.4451	-4.0363
Gm47509	-1.4408	1.0289	-2.2851	0.0414	0.4451	-3.7026
9130023H24Rik	0.4887	2.1427	2.2851	0.0414	0.4451	-3.8663
Spata24	-0.6181	1.2179	-2.2849	0.0414	0.4451	-3.7905
Rhoq	0.5427	6.4275	2.2841	0.0414	0.4453	-4.45
Akap17b	0.6916	2.0725	2.284	0.0415	0.4453	-3.858
Tjap1	0.3173	6.0951	2.283	0.0415	0.4458	-4.4331
Zbtb4	0.3053	3.7795	2.281	0.0417	0.4466	-4.1411
Arrb1	0.4235	6.2913	2.281	0.0417	0.4466	-4.4598
Gm15964	-1.7404	-1.6263	-2.281	0.0417	0.4466	-3.6145
Sbds	-0.2032	5.7444	-2.2799	0.0418	0.4472	-4.4256
P4htm	1.678	-3.0919	2.2785	0.0419	0.4474	-3.6974
Hectd1	-0.2777	8.5559	-2.2784	0.0419	0.4474	-4.5045
Lurap1	0.5815	1.1164	2.2783	0.0419	0.4474	-3.7351

Zfp26	0.3224	4.3713	2.2778	0.0419	0.4474	-4.2583
Gm6366	-1.3299	-2.8177	-2.2777	0.0419	0.4474	-3.6296
Caprin2	-0.3342	3.5542	-2.277	0.042	0.4474	-4.153
Stat2	0.6786	3.4489	2.2763	0.042	0.4474	-4.0604
Rpl17-ps3	-1.101	-1.054	-2.2761	0.042	0.4474	-3.6082
Gm48455	-0.6061	-0.163	-2.2751	0.0421	0.4474	-3.694
Gm37553	1.6898	-2.6009	2.2749	0.0421	0.4474	-3.6471
Anxa1	0.3677	9.5024	2.2749	0.0421	0.4474	-4.5174
Prrc1	-0.2972	5.5902	-2.2748	0.0421	0.4474	-4.4197
Nans	-0.1895	6.6063	-2.2748	0.0422	0.4474	-4.482
Copz2	0.5481	4.2425	2.2747	0.0422	0.4474	-4.2011
Gm12250	0.9211	-0.0808	2.2745	0.0422	0.4474	-3.6633
Plekha3	-0.2411	5.7811	-2.2737	0.0422	0.4477	-4.445
Trak1	0.3226	6.1929	2.2726	0.0423	0.4483	-4.4547
Esam	1.4916	-1.2022	2.2723	0.0423	0.4483	-3.6128
Gm17638	-0.6945	1.3587	-2.2706	0.0425	0.4492	-3.7759
0610040B10Rik	1.0112	-0.5979	2.2706	0.0425	0.4492	-3.6143
Pianp	1.6811	-3.3158	2.2689	0.0426	0.4502	-3.7157
Snn	0.5043	5.692	2.2679	0.0427	0.4502	-4.4109
9330161L09Rik	-1.4457	-2.1798	-2.2677	0.0427	0.4502	-3.6458
Bach1	0.3051	5.8865	2.2675	0.0427	0.4502	-4.4375
Mtmr1	0.3354	5.1883	2.2673	0.0427	0.4502	-4.3911
Gm49502	0.8626	-0.7285	2.267	0.0427	0.4502	-3.6272
Rplp0-ps1	-1.0185	0.8923	-2.2669	0.0428	0.4502	-3.7277
Podxl2	1.4745	2.6607	2.2652	0.0429	0.4513	-3.8864
Lpar6	0.4721	3.0303	2.2643	0.043	0.4514	-4.0405
Atp6v0e2	0.6962	3.1206	2.2643	0.043	0.4514	-3.9931
Gm8186	-0.2474	4.7863	-2.2641	0.043	0.4514	-4.3661
Zfp503	0.3602	5.9264	2.2625	0.0431	0.4516	-4.4675
Slco2a1	-0.7836	7.6786	-2.2622	0.0431	0.4516	-4.5266
Adam33	-1.9769	-2.9115	-2.2622	0.0431	0.4516	-3.6689
Ccl17	2.2845	-2.7269	2.2618	0.0431	0.4516	-3.7113
Gm9432	-1.4096	-2.3401	-2.2614	0.0432	0.4516	-3.6476
Slc10a3	0.365	4.0517	2.2609	0.0432	0.4516	-4.2186
Gm9794	-0.3819	2.8711	-2.2608	0.0432	0.4516	-4.0287
Gm6382	-1.2208	-1.588	-2.2605	0.0433	0.4516	-3.6285
Gm37131	-0.7935	-0.0818	-2.2601	0.0433	0.4516	-3.6791
4933431E20Rik	0.8194	3.0658	2.2599	0.0433	0.4516	-3.9986
Ccnyl1	0.2168	4.7042	2.2597	0.0433	0.4516	-4.3586
Gm49326	-1.282	-1.1377	-2.2595	0.0433	0.4516	-3.6272
Gm26514	-1.3785	-2.4421	-2.2595	0.0433	0.4516	-3.6443
Mlkl	-0.3423	5.3542	-2.2588	0.0434	0.4518	-4.454
Zfp202	0.4062	3.0067	2.2576	0.0435	0.4525	-4.0615
Gm36839	-1.0594	-1.6649	-2.2519	0.0439	0.4558	-3.6373
BC067074	1.0334	0.4901	2.2515	0.044	0.4558	-3.7089
Nudc	-0.2151	7.46	-2.2513	0.044	0.4558	-4.5381
Wwc1	-0.6102	5.2417	-2.251	0.044	0.4558	-4.4377
Gpr156	1.2554	-2.7724	2.2508	0.044	0.4558	-3.6844
F830016B08Rik	1.8513	-2.3801	2.2506	0.044	0.4558	-3.6485
Tpm1	0.3607	8.8777	2.2505	0.044	0.4558	-4.5544
Tmem218	0.4315	2.6884	2.25	0.0441	0.4558	-4.0005
Gm7308	-0.8894	-0.4196	-2.2499	0.0441	0.4558	-3.6629

Tmod3	0.2482	7.2593	2.2496	0.0441	0.4558	-4.5338
Glyctk	0.8214	0.7743	2.2495	0.0441	0.4558	-3.7478
Ssc5d	1.7587	0.5967	2.2494	0.0441	0.4558	-3.6778
Oas1b	1.25	-0.5332	2.2487	0.0442	0.4558	-3.6531
Prnp	-0.345	5.9993	-2.2486	0.0442	0.4558	-4.4869
Tmem106a	0.8894	3.0042	2.2476	0.0443	0.4558	-4.0283
Nt5e	-0.5708	6.7713	-2.2474	0.0443	0.4558	-4.5254
Inca1	0.739	0.339	2.2469	0.0443	0.4558	-3.6802
Pgrmc1	0.2634	6.6965	2.2468	0.0443	0.4558	-4.5264
Txk	-1.2615	-0.085	-2.2467	0.0443	0.4558	-3.709
Creb1	0.2078	6.1215	2.2463	0.0444	0.4558	-4.51
Cdh17	1.2439	-0.3867	2.2463	0.0444	0.4558	-3.6575
Gm9910	-1.6913	-1.7302	-2.2458	0.0444	0.4558	-3.6526
Zfp384	0.1903	5.9662	2.2455	0.0444	0.4558	-4.4979
Gm45030	-1.4402	-2.8211	-2.2451	0.0445	0.4558	-3.6717
Pif1	-0.3227	3.7691	-2.245	0.0445	0.4558	-4.3175
Atm	0.3114	6.0113	2.2446	0.0445	0.4558	-4.5047
Tcp11l2	0.5377	4.5957	2.2445	0.0445	0.4558	-4.2799
Insc	2.1459	-2.5433	2.2436	0.0446	0.4558	-3.7292
5930430L01Rik	0.4199	3.1378	2.2436	0.0446	0.4558	-4.0822
Zkscan3	0.2556	5.3696	2.2436	0.0446	0.4558	-4.4311
Gm26738	-1.3429	-2.7087	-2.2432	0.0446	0.4559	-3.6687
March8	0.1804	6.5232	2.2428	0.0447	0.456	-4.5243
2810405F17Rik	-1.6973	-2.4275	-2.242	0.0447	0.456	-3.7042
Atxn7l3b	0.2653	6.6223	2.242	0.0447	0.456	-4.5295
Gm6263	1.3889	-2.1166	2.2418	0.0447	0.456	-3.6851
Scarna2	1.437	-0.8948	2.2412	0.0448	0.4561	-3.6507
Nfe2l1	0.2671	8.6502	2.241	0.0448	0.4561	-4.5671
Gm14848	-1.8268	-2.795	-2.2404	0.0448	0.4563	-3.7222
Hrct1	1.6044	0.0568	2.2397	0.0449	0.4566	-3.6737
Ampd3	1.2324	5.645	2.2379	0.045	0.4578	-4.4494
Ifit1bl2	1.6683	-1.7534	2.2373	0.0451	0.458	-3.6649
Sybu	1.8941	1.3477	2.2364	0.0452	0.4583	-3.7436
Gm14586	-0.5332	1.6251	-2.2364	0.0452	0.4583	-3.8791
Mtmr7	1.1696	-1.0752	2.2361	0.0452	0.4583	-3.6569
Snrpg	-0.2861	6.4176	-2.2357	0.0452	0.4583	-4.5432
Artn	1.2546	2.6203	2.2352	0.0453	0.4584	-3.985
Marcksl1	0.4609	7.6383	2.2345	0.0453	0.4585	-4.5627
Hes6	0.3137	4.5067	2.2337	0.0454	0.4585	-4.3303
Gm9703	-2.3922	-3.0577	-2.2333	0.0454	0.4585	-3.7393
Gale	-0.6742	4.7281	-2.2331	0.0454	0.4585	-4.39
S100a14	-1.7979	-3.2977	-2.2331	0.0454	0.4585	-3.7292
Gm14387	-2.0507	-3.1211	-2.2325	0.0455	0.4585	-3.7196
1700087I21Rik	1.5602	-2.3967	2.2325	0.0455	0.4585	-3.684
Zfp651	0.5567	3.8293	2.2325	0.0455	0.4585	-4.191
Snhg6	-0.3178	4.1873	-2.2321	0.0455	0.4586	-4.3155
Sars	-0.2247	8.2813	-2.2307	0.0456	0.4594	-4.5825
Fam168a	0.2375	6.3187	2.2303	0.0457	0.4595	-4.541
Upp1	-0.6588	3.9981	-2.2297	0.0457	0.4597	-4.4287
Gm26890	1.0746	-1.341	2.227	0.0459	0.4616	-3.6686
Gm15495	-1.6622	-3.9608	-2.2268	0.046	0.4616	-3.7583
Kifap3	0.2537	6.0619	2.226	0.046	0.4618	-4.5301

Cldn6	-0.8167	-1.3442	-2.2259	0.046	0.4618	-3.6699
Bcl2l11	0.4251	5.3536	2.2256	0.0461	0.4618	-4.4367
Uchl4	-0.9046	-1.5047	-2.224	0.0462	0.4627	-3.6756
Scn2a	0.7707	2.1839	2.2237	0.0462	0.4627	-3.9425
Dip2b	0.3746	6.1616	2.2231	0.0463	0.4627	-4.5369
4930548H24Rik	-1.7619	-3.3341	-2.222	0.0463	0.4627	-3.7407
Mrip-ps	0.6159	0.1932	2.222	0.0464	0.4627	-3.7507
Arl10	-0.3344	2.6106	-2.2217	0.0464	0.4627	-4.0598
Gm15694	1.4273	-2.325	2.2214	0.0464	0.4627	-3.6851
Gpr75	2.015	-2.426	2.2213	0.0464	0.4627	-3.7244
Plekhm2	0.3224	5.2151	2.2211	0.0464	0.4627	-4.4579
Sh3bgrl	0.4198	7.4854	2.221	0.0464	0.4627	-4.5847
Farsb	-0.3121	6.8186	-2.2204	0.0465	0.4627	-4.5809
Cops3	-0.2188	6.6236	-2.2196	0.0465	0.4627	-4.5748
Gm26619	-0.5595	3.1908	-2.2189	0.0466	0.4627	-4.1433
Gm22748	-1.065	-1.0037	-2.2188	0.0466	0.4627	-3.6975
Scnn1a	0.8515	0.7662	2.2188	0.0466	0.4627	-3.7899
Zfp426	0.2765	4.0036	2.2185	0.0466	0.4627	-4.2882
Gm5857	1.1384	-1.5576	2.2184	0.0467	0.4627	-3.6809
Siglec15	-1.2815	0.3276	-2.2184	0.0467	0.4627	-3.7839
Lrsam1	0.3359	4.2341	2.2184	0.0467	0.4627	-4.3071
Xkr5	-1.7013	-1.3615	-2.2177	0.0467	0.463	-3.68
Nars	-0.3577	8.7595	-2.2154	0.0469	0.4646	-4.6147
Flna	0.3777	10.6692	2.2141	0.047	0.4654	-4.625
Fnip1	0.3901	6.1132	2.2115	0.0472	0.4672	-4.5504
9330102E08Rik	0.6032	-0.0917	2.2109	0.0473	0.4672	-3.7287
Begain	1.7958	-2.7562	2.2108	0.0473	0.4672	-3.7359
Gm43682	0.5481	0.538	2.2104	0.0473	0.4672	-3.7882
Pex19	-0.1982	6.3447	-2.21	0.0474	0.4672	-4.5785
D930036K23Rik	1.116	-1.2504	2.2089	0.0475	0.4672	-3.6913
Gm43681	0.6626	-0.3266	2.2086	0.0475	0.4672	-3.7158
Pold3	-0.2186	4.8514	-2.2084	0.0475	0.4672	-4.4863
Gpr161	0.3624	5.0489	2.2082	0.0475	0.4672	-4.4614
Areg	-0.8267	2.4635	-2.2078	0.0476	0.4672	-4.2454
Phf20l1	0.2896	5.9953	2.2077	0.0476	0.4672	-4.5598
Gm37233	0.9227	1.2726	2.2069	0.0476	0.4672	-3.8398
Ghr	1.2234	2.5596	2.2065	0.0477	0.4672	-3.9948
Psen1	0.2371	6.2797	2.2065	0.0477	0.4672	-4.5775
AI480526	0.6301	1.3179	2.2064	0.0477	0.4672	-3.8494
Cox7a1	1.2194	-0.7906	2.2062	0.0477	0.4672	-3.7017
Rpl28-ps3	-0.7408	0.4853	-2.2062	0.0477	0.4672	-3.8051
Pmf1	-0.2258	5.9496	-2.2059	0.0477	0.4672	-4.5858
Gm12231	-0.9004	-0.4853	-2.2057	0.0477	0.4672	-3.712
Snrpd2	-0.2952	6.4956	-2.2056	0.0477	0.4672	-4.5965
Scube3	2.3155	-0.0623	2.2052	0.0478	0.4673	-3.7083
Arsg	0.6705	1.3078	2.2039	0.0479	0.4676	-3.8462
Mad2l2	0.2777	4.0633	2.2036	0.0479	0.4676	-4.3259
Hyls1	-0.344	4.0707	-2.2032	0.0479	0.4676	-4.3565
Nxph3	-1.8317	-1.3835	-2.2031	0.0479	0.4676	-3.6983
Gm7962	0.8491	-0.9648	2.203	0.048	0.4676	-3.7143
Abca8b	0.7767	2.6332	2.2029	0.048	0.4676	-4.0357
Zscan30	1.3696	-0.8959	2.2024	0.048	0.4678	-3.7026

Gm43579	-0.7121	0.0208	-2.2017	0.0481	0.4681	-3.7426
Bmp4	2.0493	-0.161	2.2012	0.0481	0.4683	-3.7084
Selenom	0.8902	4.474	2.1999	0.0482	0.469	-4.3474
Cacnb3	0.6777	5.4161	2.1997	0.0482	0.469	-4.5036
Trip6	0.303	5.6307	2.1988	0.0483	0.4695	-4.547
K230015D01Rik	0.6368	0.5579	2.1968	0.0485	0.4699	-3.783
Zbtb6	0.292	4.8773	2.1966	0.0485	0.4699	-4.4695
Ubd	4.1637	-2.3449	2.1964	0.0485	0.4699	-3.788
Myh9	0.3986	9.8442	2.1963	0.0485	0.4699	-4.6515
Ythdc2	0.2941	3.9662	2.1962	0.0485	0.4699	-4.3395
Gm10120	-1.0783	-1.571	-2.1961	0.0486	0.4699	-3.7083
Ep400	0.3454	7.2304	2.1961	0.0486	0.4699	-4.6226
Slc4a11	-0.7378	2.06	-2.1948	0.0487	0.4707	-4.0248
Rsph3b	0.477	2.2183	2.194	0.0487	0.4711	-4.0166
Ift74	0.3106	5.023	2.1935	0.0488	0.4712	-4.4837
Gm13431	-1.5624	-1.3879	-2.1933	0.0488	0.4712	-3.7118
Dnajc8	-0.1876	6.6893	-2.193	0.0488	0.4712	-4.6201
Gm5555	-0.9603	-2.0333	-2.1914	0.049	0.4717	-3.7187
Slc49a4	0.3983	4.5812	2.1913	0.049	0.4717	-4.3912
Olfml3	1.2256	4.0602	2.1908	0.049	0.4717	-4.3049
Msln	0.9343	5.398	2.1906	0.049	0.4717	-4.5291
Pfdn4	-0.3327	5.4149	-2.1894	0.0491	0.4717	-4.5646
Gm48804	1.5072	-3.7001	2.1892	0.0492	0.4717	-3.7946
Usp25	0.3063	5.7375	2.1891	0.0492	0.4717	-4.575
Ptpn18	1.6859	-2.7653	2.1888	0.0492	0.4717	-3.7652
Mef2b	1.1018	-0.7612	2.1887	0.0492	0.4717	-3.7199
Cish	1.0054	0.2306	2.1881	0.0493	0.4717	-3.8406
Nr2c1	0.2636	4.0315	2.188	0.0493	0.4717	-4.3354
Zfp39	0.4506	2.1287	2.1872	0.0493	0.4717	-4.0232
Ifi35	0.4725	4.7775	2.1865	0.0494	0.4717	-4.4589
Tspan12	1.2014	1.1168	2.1862	0.0494	0.4717	-3.8412
Ttyh2	0.326	5.141	2.1861	0.0494	0.4717	-4.5049
Rnf38	0.3668	5.3118	2.1852	0.0495	0.4717	-4.531
Chpt1	0.5212	4.3692	2.1851	0.0495	0.4717	-4.3949
Abhd4	0.3399	6.336	2.1845	0.0496	0.4717	-4.6047
Ap1s1	-0.2195	6.9285	-2.1839	0.0496	0.4717	-4.6414
Gm9134	-1.5931	-2.4376	-2.1838	0.0496	0.4717	-3.7393
Qrfp	-2.0022	-3.7324	-2.1837	0.0496	0.4717	-3.7938
Neurl1a	1.0931	0.9614	2.183	0.0497	0.4717	-3.8167
Peli3	1.7105	-0.2755	2.1829	0.0497	0.4717	-3.7274
Ulk1	0.4498	5.9173	2.1829	0.0497	0.4717	-4.5794
Psca	2.1491	-2.694	2.1827	0.0497	0.4717	-3.7888
Gm27960	0.9395	0.3312	2.1826	0.0497	0.4717	-3.846
Gm4705	-0.8566	-1.8009	-2.1824	0.0498	0.4717	-3.7239
Wdfy3	0.3787	6.6787	2.1823	0.0498	0.4717	-4.629
Gm28404	-1.9166	-3.1788	-2.1815	0.0498	0.4717	-3.7661
Rragc	0.241	6.9687	2.1815	0.0498	0.4717	-4.641
Eif1-ps1	-1.2808	-1.3485	-2.1814	0.0499	0.4717	-3.7251
Stx2	0.2439	4.737	2.1812	0.0499	0.4717	-4.4531
Gm8784	-1.7536	-2.2172	-2.1809	0.0499	0.4717	-3.7466
Gm12481	0.5703	0.9301	2.1807	0.0499	0.4717	-3.865
Cd14	-0.5914	4.4018	-2.1807	0.0499	0.4717	-4.4416

Gm10222	1.3435	-2.6312	2.1804	0.0499	0.4717	-3.7697
Itln1	1.0279	-0.3849	2.1804	0.0499	0.4717	-3.7499
Sohlh2	1.4823	-3.7361	2.1804	0.0499	0.4717	-3.8029

Supplementary Table 3. List of the most significant differentially expressed genes in Combo versus DMSO treated tumourspheres

GeneID	logFC	AveExpr	t	P.Value	adj.P.Val	B
Birc5	-2.7252	6.3221	-27.4062	0	0	17.923
Pimreg	-2.4535	5.668	-23.5728	0	0	16.3073
Cdk1	-2.2829	6.8158	-22.2961	0	0	15.9389
Hmga2	-2.843	7.7505	-20.3312	0	0	14.9545
Ccnd1	-5.2848	8.4099	-20.2525	0	0	14.8548
Etv5	-5.021	4.5905	-20.0796	0	0	12.8923
Mcm3	-1.911	7.5772	-20.0519	0	0	14.8077
Aurkb	-2.7738	5.6959	-19.831	0	0	14.488
Cdca8	-2.1969	5.7631	-19.6013	0	0	14.45
Top2a	-2.5267	9.3103	-18.8383	0	0	14.1143
Etv4	-4.9322	6.0795	-18.8343	0	0	13.5805
Kif15	-2.846	5.5538	-18.617	0	0	13.773
Zwilch	-2.1051	5.3028	-18.4808	0	0	13.756
Mybl2	-3.0538	5.7895	-18.2064	0	0	13.5643
Cks1b	-1.8645	7.4558	-18.1131	0	0	13.6645
Dtl	-1.8718	5.9391	-17.7699	0	0	13.4103
Lig1	-1.8816	6.3574	-17.7278	0	0	13.4055
Cenpi	-2.2989	5.4404	-17.7249	0	0	13.3092
Uhrf1	-2.6032	6.9895	-17.656	0	0	13.3629
Tubb4b	-2.0104	7.9197	-17.6085	0	0	13.3457
Trip13	-2.356	5.766	-17.5712	0	0	13.2509
Kpna2	-1.3626	7.3026	-17.4468	0	0	13.2399
Melk	-2.4095	5.0966	-17.3309	0	0	12.9983
Rrm2	-3.1659	7.0058	-17.2594	0	0	13.0982
Dusp6	-5.1139	5.9731	-17.0466	0	0	12.48
Gmnn	-1.983	5.5136	-17.0408	0	0	12.9247
Cks2	-1.9416	5.8769	-16.9697	0	0	12.8993
Mcm5	-2.1574	7.0038	-16.8592	0	0	12.8475
Nsl1	-2.25	4.1811	-16.8076	0	0	12.4816
Clspn	-2.3382	5.2858	-16.7267	0	0	12.6583
Hjurp	-1.7327	6.4564	-16.5725	0	0	12.651
Cdt1	-1.8695	5.8526	-16.4085	0	0	12.526
Dhfr	-2.2964	5.887	-16.3995	0	0	12.5078
Cdca3	-2.2694	5.7786	-16.3662	0	0	12.4844
Chaf1a	-1.6591	6.6913	-16.225	0	0	12.4128
Lyar	-2.1119	6.8199	-16.0754	0	0	12.3073
Pclaf	-3.3626	6.3448	-15.8745	0	0	12.1273
Cenpq	-2.1715	4.5202	-15.8694	0	0	11.9962
Espl1	-2.2944	6.1709	-15.8466	0	0	12.1364
Tmpo	-1.2983	7.7569	-15.8456	0	0	12.1415
Hmgb2	-2.0688	7.5459	-15.8191	0	0	12.1235
Bub1	-2.4839	6.1696	-15.729	0	0	12.0504
Rad18	-1.9304	5.5654	-15.6418	0	0	11.9818
Aurka	-2.2104	6.2699	-15.6392	0	0	11.9929
Pmf1	-1.7489	5.9496	-15.623	0	0	11.9808
Kif22	-2.1211	6.0146	-15.6103	0	0	11.969
Spc24	-2.2822	4.3535	-15.3562	0	0	11.6131
Ccnf	-1.9789	5.6937	-15.3311	0	0	11.7604
Shmt2	-1.9656	7.0876	-15.313	0	0	11.7531

Plk1	-2.4106	6.3982	-15.2891	0	0	11.7374
Lmnb1	-1.8844	7.1403	-15.2054	0	0	11.6725
Rangap1	-1.6104	7.5804	-15.1667	0	0	11.6403
Kif23	-2.4412	6.6459	-15.0836	0	0	11.585
Cdc20	-2.1474	6.8117	-15.0674	0	0	11.5713
Fam111a	-2.3801	7.3701	-15.0617	0	0	11.5645
Tipin	-1.9561	5.8012	-15.0552	0	0	11.5614
Sgo1	-2.2733	5.4117	-14.8323	0	0	11.3753
Rfc5	-1.7645	5.9284	-14.8002	0	0	11.3708
Ier3	-2.7065	8.5462	-14.7344	0	0	11.3063
Hirip3	-2.2763	5.0086	-14.7289	0	0	11.271
Ube2s	-1.5895	6.0022	-14.6709	0	0	11.2708
Incenp	-1.9984	6.7313	-14.6694	0	0	11.2664
Ndc80	-1.845	5.5316	-14.6654	0	0	11.2644
Arhgap19	-2.627	5.3287	-14.6364	0	0	11.2086
Rad51	-2.257	5.2007	-14.6243	0	0	11.2056
Mpp6	-1.9331	6.9977	-14.5962	0	0	11.2063
Cdc6	-2.5495	5.2842	-14.5655	0	0	11.1502
Exo1	-2.2025	5.1549	-14.557	0	0	11.1512
Gdpd2	4.848	4.6212	14.4737	0	0	10.944
Sapcd2	-3.03	3.7726	-14.451	0	0	10.649
Smc2	-1.836	7.1185	-14.4448	0	0	11.0853
Spdl1	-1.6537	4.9812	-14.4294	0	0	11.0731
Kntc1	-2.4649	5.3741	-14.3801	0	0	11.0254
Mcm2	-1.3791	7.3894	-14.3609	0	0	11.0146
Lbr	-2.0267	6.8892	-14.2928	0	0	10.9677
Chek1	-1.942	4.6182	-14.2908	0	0	10.926
Pcna	-1.5072	7.5822	-14.2851	0	0	10.9529
Rin2	1.8174	6.9121	14.2261	0	0	10.9073
Nuf2	-2.4959	5.6363	-14.2187	0	0	10.9124
Exosc8	-1.9308	6.3675	-14.2137	0	0	10.9101
Prim1	-1.8845	5.2403	-14.2033	0	0	10.9
Mcm6	-1.6312	7.8302	-14.1696	0	0	10.8586
Mthfd2	-2.4245	6.4301	-14.126	0	0	10.842
Nasp	-1.6405	7.0183	-14.1247	0	0	10.8284
Ncaph	-2.0343	5.8802	-13.9452	0	0	10.701
Sgo2a	-1.9897	6.0755	-13.9432	0	0	10.6973
Cenpa	-2.4487	6.4361	-13.8963	0	0	10.6579
Asf1b	-2.2213	5.2454	-13.8911	0	0	10.6489
Aunip	-2.4031	3.4006	-13.8729	0	0	10.3008
Ndc1	-1.6862	6.3207	-13.8606	0	0	10.6241
Mad2l1	-1.872	6.168	-13.8588	0	0	10.6268
Spc25	-2.1809	4.6708	-13.8257	0	0	10.5541
Anp32e	-1.5923	7.9972	-13.7805	0	0	10.5375
Spag5	-2.0612	5.8486	-13.7644	0	0	10.5545
Ncapg	-2.7095	6.6482	-13.7443	0	0	10.5336
Kif11	-2.2589	6.7028	-13.6699	0	0	10.4659
Tk1	-4.0544	5.7133	-13.6552	0	0	10.38
Shcbp1	-2.5263	5.2977	-13.6028	0	0	10.41
BC030867	-2.2501	4.2849	-13.5972	0	0	10.3308
Rfc4	-1.5694	5.1521	-13.5339	0	0	10.3669
Kif18b	-2.0483	5.3906	-13.5084	0	0	10.3455

Blm	-1.8953	5.2181	-13.484	0	0	10.3244
Chtf18	-1.9193	4.7648	-13.465	0	0	10.2944
Pold1	-1.5708	5.5827	-13.4569	0	0	10.2999
Fignl1	-2.1717	5.2403	-13.3794	0	0	10.2354
E2f8	-3.694	5.7787	-13.3662	0	0	10.1915
Foxm1	-1.9389	6.7378	-13.3537	0	0	10.1942
Fosl1	-3.3977	7.3538	-13.3324	0	0	10.1826
Ncapg2	-1.9566	6.3887	-13.2807	0	0	10.1408
Topbp1	-1.3242	7.6861	-13.2426	0	0	10.0818
Ccna2	-2.516	7.6249	-13.1705	0	0	10.0264
Etv1	-2.9983	5.77	-13.1045	0	0	10.0053
Ncapd3	-1.7101	5.8884	-13.0918	0	0	9.9847
Ska3	-2.0803	4.8801	-13.0883	0	0	9.9857
Eps8	-2.5294	7.9181	-13.054	0	0	9.9206
Cip2a	-2.1185	6.5635	-12.9888	0	0	9.886
Nuak1	1.5973	4.5078	12.9256	0	0	9.8554
Ruvbl2	-1.5179	6.9584	-12.916	0	0	9.8033
Nup107	-1.6782	6.3979	-12.8963	0	0	9.7999
Mcm10	-2.2212	5.4251	-12.8784	0	0	9.8148
Ccnb1	-2.2698	6.9303	-12.875	0	0	9.778
Ube2c	-2.1625	7.0339	-12.8681	0	0	9.7668
Pola1	-2.389	6.561	-12.8304	0	0	9.7518
Wdr81	1.7132	5.612	12.7626	0	0	9.6905
BC055324	-1.7693	5.1969	-12.728	0	0	9.6841
Kcnn4	-3.8727	5.8699	-12.6946	0	0	9.642
Mthfd1l	-1.7405	6.363	-12.6787	0	0	9.608
Prc1	-1.8448	6.99	-12.6451	0	0	9.5646
Nucks1	-1.507	7.9715	-12.5836	0	0	9.4946
Tex30	-1.5036	4.876	-12.5374	0	0	9.5175
Kif2c	-2.1182	5.7402	-12.5303	0	0	9.5023
Parpbp	-2.3864	4.0355	-12.4528	0	0	9.3687
Lsm3	-1.4592	5.7497	-12.4459	0	0	9.4109
Spred2	-2.9663	5.7767	-12.406	0	0	9.4016
Ncapd2	-1.7765	6.8892	-12.3857	0	0	9.3295
Gtse1	-2.272	5.0264	-12.3772	0	0	9.3748
Nup160	-1.7526	6.4218	-12.3514	0	0	9.3113
Rad54b	-2.0992	2.9796	-12.3213	0	0	9.0906
Spry2	-2.3453	4.6193	-12.306	0	0	9.2973
Emp1	-1.8298	8.6562	-12.2897	0	0	9.2198
Cep55	-2.6861	5.5587	-12.2851	0	0	9.2934
Pif1	-2.6719	3.7691	-12.2843	0	0	9.1493
Ehd1	-1.2032	8.0649	-12.2195	0	0	9.1566
Racgap1	-1.8161	7.4784	-12.186	0	0	9.1334
Dck	-1.3357	5.5757	-12.186	0	0	9.1796
Rad51ap1	-2.5383	4.1599	-12.1784	0	0	9.1226
Nup85	-1.6637	6.9265	-12.1605	0	0	9.1179
Fancd2	-2.3844	4.743	-12.1293	0	0	9.1448
Tacc3	-1.9418	6.5944	-12.1268	0	0	9.099
Mcm4	-1.4803	7.4588	-12.1095	0	0	9.0603
Kif14	-2.5137	4.8515	-12.0911	0	0	9.1079
Parvb	-1.9115	6.4683	-12.0269	0	0	9.0075
Mnd1	-1.7808	4.064	-11.9994	0	0	9.0245

Oip5	-2.1383	4.3368	-11.9965	0	0	9.0147
Lamb2	2.4919	6.2319	11.9918	0	0	8.9714
Spp1	-2.9646	10.9872	-11.9447	0	0	8.8863
Acadm	1.7932	6.4241	11.9434	0	0	8.9125
Atad2	-1.6888	6.9254	-11.9422	0	0	8.9129
Gstcd	-1.5997	5.4476	-11.9385	0	0	8.958
Dnajc9	-1.5451	5.7224	-11.9163	0	0	8.9235
Asns	-1.6439	7.1447	-11.9022	0	0	8.8658
Haus6	-1.4373	5.5935	-11.8864	0	0	8.8997
Hmmr	-2.4454	6.6623	-11.8559	0	0	8.8529
Tfrc	-1.2241	8.4671	-11.8435	0	0	8.7972
Rbl1	-1.5751	5.4242	-11.8193	0	0	8.8491
Cdca5	-2.0242	4.8617	-11.8123	0	0	8.8634
Gabarap	1.3885	7.6033	11.8071	0	0	8.7644
Rcc1	-1.7297	6.4363	-11.7743	0	0	8.7676
Sass6	-1.443	4.4362	-11.7563	0	0	8.8115
Gns	1.1993	6.9383	11.7561	0	0	8.7221
Nek2	-1.9465	6.3462	-11.7298	0	0	8.7306
Snx33	1.7942	4.8934	11.696	0	0	8.7334
Cinp	-1.2948	5.5934	-11.6807	0	0	8.6987
Haus1	-1.3869	5.0381	-11.6803	0	0	8.7217
Grn	1.755	9.191	11.6455	0	0	8.5984
Suv39h2	-2.1567	4.4634	-11.6201	0	0	8.6771
Ccnb2	-2.0235	6.3783	-11.6115	0	0	8.6143
Neu1	1.859	5.0887	11.5817	0	0	8.6153
Ipo5	-1.2816	8.414	-11.5811	0	0	8.5414
Prkca	-1.1727	6.3234	-11.5765	0	0	8.5661
Rfc2	-1.161	5.7774	-11.5663	0	0	8.5749
Ipo13	1.5894	6.01	11.4963	0	0	8.4921
Tubb6	-2.6408	8.1187	-11.4958	0	0	8.4656
Fancb	-2.5242	4.0048	-11.4559	0	0	8.4775
Selenoh	-1.2956	5.9943	-11.4505	0	0	8.4563
Klf16	-1.1307	5.0008	-11.4311	0	0	8.482
Mxd4	2.3368	6.7497	11.4276	0	0	8.4082
Epha2	-4.4291	6.1685	-11.4222	0	0	8.4954
Nup93	-0.9717	6.8965	-11.4186	0	0	8.3955
Washc2	1.326	7.2474	11.4178	0	0	8.3858
Cenpw	-1.9817	3.6999	-11.4013	0	0	8.4438
Cenpn	-1.8076	4.102	-11.3834	0	0	8.4534
Gins1	-2.1428	3.6187	-11.3761	0	0	8.3935
Nup62	-1.1303	6.3342	-11.3735	0	0	8.3664
Pld3	2.0806	5.1223	11.3664	0	0	8.4128
Ercc6l	-1.9929	5.6271	-11.3604	0	0	8.4077
Cenpf	-2.2417	7.1689	-11.3473	0	0	8.3354
Rgma	2.323	4.326	11.2752	0	0	8.3551
Mis18bp1	-2.1208	4.8659	-11.273	0	0	8.3536
Ybx3	-1.136	9.3096	-11.2553	0	0	8.2105
Cenpu	-2.1553	3.6265	-11.2409	0	0	8.2681
Fen1	-1.4656	6.139	-11.2256	0	0	8.2329
Cdc45	-1.9562	5.6015	-11.2205	0	0	8.2672
Pbk	-3.6245	5.5266	-11.214	0	0	8.2951
E2f7	-2.1619	4.3274	-11.1835	0	0	8.2637

C3	3.6394	8.8358	11.1507	0	0	8.1084
Hmga1b	-2.7398	8.1156	-11.1495	0	0	8.1184
Iqgap3	-2.1192	5.8488	-11.1475	0	0	8.1893
Smc4	-1.6549	8.6502	-11.1474	0	0	8.1062
Glmp	1.2054	7.3337	11.1435	0	0	8.1084
Lsm6	-1.4434	5.8635	-11.1356	0	0	8.1501
Timeless	-1.3168	5.0823	-11.1329	0	0	8.1913
Ctsa	1.5717	8.0657	11.0944	0	0	8.0524
Pkmyt1	-1.5734	4.2629	-11.0877	0	0	8.1752
Ints4	1.1451	5.8241	11.0779	0	0	8.0752
Tmem97	-1.5376	6.314	-11.0648	0	0	8.0631
Nap1l1	-0.8864	8.7006	-11.0622	0	0	8.0166
Mars	-1.1781	6.6743	-11.0548	0	0	8.0326
Cenpl	-1.3219	4.2199	-11.0519	0	0	8.1394
Kif4	-1.6354	6.3135	-11.049	0	0	8.0502
Anln	-2.1549	8.1186	-11.0382	0	0	8.0008
Lrr1	-2.7319	2.3172	-11.0379	0	0	7.6735
Cars	-1.3854	6.5935	-11.0143	0	0	7.9957
Plk4	-2.3421	5.762	-11.0074	0	0	8.0602
Troap	-2.0653	4.7465	-10.9954	0	0	8.0824
Ckap2l	-1.9761	5.9815	-10.9809	0	0	8.0064
Rps6ka1	-1.1788	6.5337	-10.9597	0	0	7.9399
Bcl9l	1.5972	6.7939	10.9479	0	0	7.9164
Pole2	-2.4028	3.9255	-10.9401	0	0	8.0021
Tpp1	1.5196	6.9115	10.9374	0	0	7.9027
Dlk2	2.6622	3.1153	10.9307	0	0	7.966
Dek	-1.566	7.864	-10.9221	0	0	7.8817
Aamdc	1.6974	2.8058	10.9179	0	0	7.9729
Ssbp3	1.0834	6.1411	10.915	0	0	7.8956
Kif20a	-1.8121	7.5407	-10.8723	0	0	7.8354
Pold3	-1.1825	4.8514	-10.8721	0	0	7.9293
Arel1	1.1618	5.743	10.86	0	0	7.8543
Cenpe	-2.053	7.5909	-10.8596	0	0	7.8239
Cdca2	-2.1423	5.4921	-10.8583	0	0	7.9144
Fbxo5	-2.8471	4.305	-10.8403	0	0	7.9021
AC149090.1	1.3434	6.1871	10.8363	0	0	7.8153
Hexa	1.81	6.7116	10.8293	0	0	7.795
Hmces	-1.4332	4.5123	-10.8217	0	0	7.8993
Knl1	-2.5283	5.8143	-10.8008	0	0	7.8569
Gas1	1.1594	6.8662	10.7747	0	0	7.7354
Xrcc2	-1.7133	3.8903	-10.7731	0	0	7.8621
Gm2a	2.1355	5.2242	10.7726	0	0	7.8058
Anp32b	-1.2558	8.3287	-10.7669	0	0	7.7136
Umps	-1.365	6.439	-10.7511	0	0	7.7319
Gpsm1	1.479	4.8225	10.7506	0	0	7.7954
Nemp1	-1.619	4.4563	-10.7046	0	0	7.7909
H2afz	-1.1042	8.559	-10.7039	0	0	7.6449
Acp2	1.1386	5.2708	10.6985	0	0	7.7076
P2rx4	1.6088	4.8011	10.6833	0	0	7.7295
Mroh1	1.6699	6.364	10.657	0	0	7.6228
Eif4ebp1	-1.1114	7.2154	-10.6539	0	0	7.6044
Vars	-1.4846	7.4384	-10.6357	0	0	7.585

D030056L22Rik	-1.2599	5.1564	-10.6351	0	0	7.6726
Ttk	-1.9801	5.5345	-10.6127	0	0	7.6553
2610008E11Rik	1.6243	3.4354	10.5901	0	0	7.6809
Eme1	-1.8228	3.7243	-10.5869	0	0	7.6709
Mapk8ip1	1.7098	4.0029	10.5812	0	0	7.6667
Lipa	1.8647	6.3276	10.5684	0	0	7.5323
Phf19	-2.2765	4.316	-10.5666	0	0	7.6571
Ska1	-2.4551	4.2726	-10.5533	0	0	7.6383
Wdhd1	-2.0155	5.5755	-10.5514	0	0	7.5919
Kansl1l	1.627	4.4142	10.5509	0	0	7.6148
Suv39h1	-1.2943	6.6837	-10.5501	0	0	7.5097
Rrm1	-1.4404	8.0163	-10.5334	0	0	7.4692
Slc2a6	3.115	4.2593	10.5315	0	0	7.6206
Tuba1c	-1.596	7.3211	-10.5071	0	0	7.4517
Twist1	-1.0858	6.4905	-10.498	0	0	7.4557
Nup205	-1.3425	7.7609	-10.4933	0	0	7.4289
Tpx2	-1.9556	7.4944	-10.4832	0	0	7.426
Ube2t	-1.6292	4.0755	-10.4826	0	0	7.5707
Ccdc34	-1.1847	5.3067	-10.478	0	0	7.493
Rpa2	-1.1981	5.5953	-10.4735	0	0	7.4746
Ap5z1	1.4048	3.8435	10.442	0	0	7.5243
Pask	-1.6904	4.0846	-10.4289	0	0	7.5172
Slfn9	-1.6921	5.8187	-10.427	0	0	7.4335
Kifc1	-1.8795	5.1862	-10.4048	0	0	7.4518
Lrrfip1	-1.071	7.3889	-10.3838	0	0	7.3135
Bub1b	-1.8632	7.1716	-10.3825	0	0	7.3238
Fas	3.633	4.0401	10.3675	0	0	7.4451
Cdca7	-1.6874	5.8695	-10.3673	0	0	7.3615
Pa2g4	-1.4365	8.1785	-10.3658	0	0	7.2871
Sema6b	-2.293	3.7561	-10.3498	0	0	7.4229
Cdca4	-1.3151	5.9063	-10.3266	0	0	7.302
Mtap	-0.9412	7.773	-10.3262	0	0	7.2462
Uap1l1	2	6.2737	10.3259	0	0	7.2727
Mms22l	-1.6272	4.6087	-10.3227	0	0	7.3902
Rps6ka4	-1.1977	5.3872	-10.3114	0	0	7.3092
Trp53inp2	3.4432	5.5606	10.3101	0	0	7.3285
Gba	1.3442	6.2332	10.2393	0	0	7.1755
Pnpla2	1.5909	5.3043	10.2333	0	0	7.214
Adgrl2	1.3885	7.1735	10.2291	0	0	7.1443
Foxc1	2.1616	6.2336	10.2173	0	0	7.1646
Dusp5	-3.1184	3.7973	-10.1991	0	0	7.1998
Reep4	-1.3007	5.7295	-10.1768	0	0	7.1478
Sh3kbp1	-1.1975	7.294	-10.1568	0	0	7.0677
Cmss1	-1.5654	4.3647	-10.1545	0	0	7.2189
Map1lc3b	1.2545	7.1849	10.1501	0	0	7.0561
Cit	-1.6004	5.1201	-10.1376	0	0	7.1611
Cdc7	-2.3126	4.5285	-10.1364	0	0	7.2129
Traip	-1.6199	4.1515	-10.1357	0	0	7.2104
Shmt1	-1.6653	6.2114	-10.1323	0	0	7.0864
Hspd1	-1.3214	9.8463	-10.1178	0	0	7.0034
Irf8	-2.9767	3.9514	-10.1164	0	0	7.1675
11-sept	-1.2524	8.341	-10.0978	0	0	6.9909

Ifrd2	-2.0576	5.6215	-10.0824	0	0	7.0844
Sms	-1.4117	6.7634	-10.0584	0	0	6.9734
Nup37	-1.6239	5.1395	-10.0518	0	0	7.0642
Diaph3	-1.7635	6.3068	-10.0441	0	0	6.9867
Ticrr	-2.0169	4.4432	-10.0394	0	0	7.1081
Gins2	-1.3983	4.0947	-10.0297	0	0	7.0944
Ptgs1	-1.3856	8.1672	-10.025	0	0	6.9107
Cct7	-0.9314	9.1018	-10.0156	0	0	6.8921
Gm43737	1.8748	2.5434	10.0145	0	0	7.0498
Hpf1	-1.2281	5.4517	-10.0128	0	0	6.9804
Atad5	-1.7027	4.9235	-9.9943	0	0	7.0232
Odc1	-3.0622	9.0937	-9.9912	0	0	6.872
Rad54l	-1.9005	4.2295	-9.9834	0	0	7.05
Ubald2	-1.1353	5.1815	-9.9765	0	0	6.9528
Galk1	-1.4464	6.693	-9.9641	0	0	6.8681
Cenph	-1.6508	4.4909	-9.9536	0	0	7.0022
Tfap4	-1.1965	4.7097	-9.9531	0	0	6.9726
Orc1	-1.5209	3.9426	-9.9288	0	0	6.9937
Lin9	-1.1504	4.9384	-9.9247	0	0	6.9232
Lsm5	-1.1937	5.0219	-9.9233	0	0	6.9118
Nup43	-1.6967	4.6664	-9.9141	0	0	6.9499
Skp2	-1.7311	5.3051	-9.912	0	0	6.9051
Cbr2	3.446	5.0056	9.9041	0	0	6.9254
Ptprn	-2.1788	6.8171	-9.903	0	0	6.8087
Usp1	-1.57	6.7024	-9.9029	0	0	6.8063
Itm2b	1.9524	8.5069	9.8915	0	0	6.7558
Aplp2	1.2904	9.2321	9.8677	0	0	6.7237
Fam83d	-1.9771	4.3985	-9.8619	0	0	6.9146
Ddx58	1.5437	5.4203	9.8588	0	0	6.7967
Cdk2	-1.0779	5.422	-9.8522	0	0	6.7991
Wls	1.9888	7.953	9.8226	0	0	6.682
Tfdp1	-1.108	7.5563	-9.781	0	0	6.6428
Poc1b	-1.23	4.4	-9.7788	0	0	6.7996
Ip6k1	0.9034	6.2058	9.7741	0	0	6.6551
Lurap1l	1.9785	4.4287	9.7729	0	0	6.7837
Prr11	-2.167	6.279	-9.7622	0	0	6.6842
Pold2	-1.3541	6.9241	-9.7591	0	0	6.6293
Fanci	-1.9499	4.0432	-9.7545	0	0	6.8074
Pola2	-1.4066	6.2145	-9.7471	0	0	6.6434
Mtor	1.0636	6.1852	9.737	0	0	6.6154
Pik3r1	1.9835	6.9304	9.7367	0	0	6.5995
Hnrnpa2b1	-1.0358	10.0904	-9.734	0	0	6.5691
Cenpk	-2.0496	2.934	-9.7319	0	0	6.7344
Haspin	-1.4962	4.4713	-9.7242	0	0	6.7449
Exosc2	-1.4946	5.8707	-9.7191	0	0	6.6361
Ckap5	-1.0827	7.6844	-9.7176	0	0	6.568
Ckap2	-1.8789	5.734	-9.7012	0	0	6.6435
Depdc1b	-2.4403	3.7754	-9.688	0	0	6.7301
Banf1	-1.116	7.1471	-9.6858	0	0	6.538
Sqstm1	1.1774	9.0123	9.6764	0	0	6.5057
Ptges	-2.5432	8.2832	-9.6629	0	0	6.5052
Pole	-2.1878	5.6474	-9.6512	0	0	6.6115

Sae1	-0.8519	7.4856	-9.6399	0	0	6.48
Gse1	1.9941	5.3937	9.6318	0	0	6.5508
Zmynd19	-1.1387	6.0996	-9.6051	0	0	6.4807
Kifc5b	-1.8971	3.8496	-9.5851	0	0	6.6239
Cd44	-2.2832	8.4118	-9.5842	0	0	6.4106
Mfsd11	1.2074	5.3258	9.5741	0	0	6.4732
Eaf1	-1.1206	6.7831	-9.5659	0	0	6.4085
Hmga1	-2.6796	7.76	-9.5456	0	0	6.3804
Gadd45b	2.4067	3.8647	9.5297	0	0	6.5538
Klf3	1.5977	6.5166	9.523	0	0	6.3611
Tlr2	3.174	4.3431	9.5009	0	0	6.5159
Tardbp	-1.1425	8.0392	-9.4989	0	0	6.3109
Fat1	1.2177	6.2489	9.4921	0	0	6.3339
Dock9	-1.3522	7.0632	-9.4916	0	0	6.3173
Ssrp1	-1.0223	8.5012	-9.4868	0	0	6.2917
Hnrnpd	-1.1496	7.9469	-9.4843	0	0	6.295
Clcn7	1.073	5.4217	9.4803	0	0	6.3557
Relb	1.2558	5.2926	9.4623	0	0	6.3477
Vrk1	-1.2446	5.5947	-9.4539	0	0	6.3389
Kif20b	-2.0027	6.3523	-9.4519	0	0	6.3186
Ap3d1	0.9845	6.5093	9.4476	0	0	6.2685
Arhgap11a	-1.7824	6.3366	-9.4309	0	0	6.2858
Tram2	1.1936	4.836	9.4066	0	0	6.3177
Cst3	1.8877	8.1938	9.4028	0	0	6.1936
App	1.3899	8.7532	9.3883	0	0	6.1718
Hcfc1r1	2.012	5.1826	9.3779	0	0	6.2749
mt-Nd4	0.9642	9.65	9.3694	0	0	6.145
Abcb1b	-0.9573	6.4008	-9.3638	0	0	6.1837
Map1lc3a	1.5797	5.6305	9.363	0	0	6.2152
Ppa1	-1.5036	6.7328	-9.3592	0	0	6.1747
Gemin6	-1.5661	4.4244	-9.3547	0	0	6.3353
Tsc22d3	2.2522	6.9777	9.3514	0	0	6.1494
Ebp	1.1213	5.9975	9.3482	0	0	6.1681
Ahcy	-1.2123	5.6105	-9.3412	0	0	6.2021
Cdc25c	-2.1779	5.0444	-9.3411	0	0	6.2951
Tyms	-1.9259	5.8314	-9.3398	0	0	6.2229
Pik3r3	2.4884	3.88	9.3327	0	0	6.3378
Lamb1	-1.8699	8.9375	-9.3244	0	0	6.0997
Poc1a	-1.27	3.755	-9.3206	0	0	6.3186
Ypel2	1.7228	3.9013	9.316	0	0	6.2982
Nub1	1.0982	6.9367	9.3146	0	0	6.1031
Hat1	-1.7233	6.3121	-9.3118	0	0	6.1446
Synrg	1.6496	5.5803	9.3048	0	0	6.148
Scx	2.0524	3.9367	9.303	0	0	6.2825
Sirt2	1.0268	5.8583	9.2952	0	0	6.1094
Pacs2	1.6591	5.3203	9.2905	0	0	6.1464
Mxd3	-2.2768	3.1434	-9.2885	0	0	6.2737
Bin1	-1.072	7.5333	-9.284	0	0	6.0628
Enoph1	-1.137	5.1242	-9.2832	0	0	6.1709
Ranbp1	-1.2808	7.4524	-9.2782	0	0	6.0586
Bard1	-2.0952	3.7396	-9.2565	0	0	6.2623
Tax1bp3	1.8246	5.1229	9.2549	0	0	6.1304

Neil3	-2.6356	2.6771	-9.2502	0	0	6.1327
Lamp2	1.2902	8.7815	9.2287	0	0	5.9821
Recql4	-2.0965	4.1537	-9.2134	0	0	6.2079
4930579G24Rik	-1.0632	4.6967	-9.21	0	0	6.1254
Snrpa1	-1.6019	5.9445	-9.205	0	0	6.0336
Gars	-0.9863	8.4389	-9.2046	0	0	5.9573
Farsb	-1.3185	6.8186	-9.2011	0	0	5.9794
Trafd1	1.4158	4.6917	9.1878	0	0	6.0742
Cdc25b	-1.9169	6.1197	-9.1847	0	0	6.0099
Atp6v1f	0.8472	6.077	9.1816	0	0	5.9655
Msrb3	2.4482	3.8746	9.1605	0	0	6.1412
Mfsd2a	-2.2324	3.8041	-9.1597	0	0	6.1534
Fuca2	2.1418	3.6498	9.149	0	0	6.1265
Rbpj	-1.4467	6.806	-9.1436	0	0	5.9143
Mxra8	1.7637	5.9811	9.1432	0	0	5.9342
Tedc1	-1.3505	4.0146	-9.1407	0	0	6.1001
Ulbp1	-1.4561	5.9193	-9.1328	0	0	5.9481
Cdkn2d	-1.7826	4.5649	-9.1251	0	0	6.0568
Atp6v0d1	1.7664	6.3336	9.1245	0	0	5.8947
Rnf126	-1.1426	6.1542	-9.1156	0	0	5.8996
Lrrc40	-0.8885	6.0128	-9.1064	0	0	5.8925
2810408I11Rik	-2.2957	2.3857	-9.1035	0	0	5.998
Zfp637	1.7807	4.8345	9.1034	0	0	5.9653
Atp6v1d	1.1306	5.7795	9.0956	0	0	5.877
Cse1l	-1.4157	7.6976	-9.0745	0	0	5.8119
Dtymk	-1.208	6.1227	-9.0696	0	0	5.8495
Parp3	1.6087	5.0421	9.0695	0	0	5.9072
E130308A19Rik	1.5015	3.8838	9.0595	0	0	6.0004
Hells	-1.9318	6.8089	-9.0528	0	0	5.8196
Vipas39	1.2416	5.0934	9.0399	0	0	5.8569
Fkbp4	-0.8979	8.34	-9.0311	0	0	5.7486
Mcm7	-1.3173	7.6231	-9.0282	0	0	5.7555
Lxn	1.9028	6.1775	9.0244	0	0	5.7834
Ncl	-1.1201	10.8141	-9.0192	0	0	5.7216
Polq	-2.1644	4.0479	-9.018	0	0	5.9869
Rnf185	0.9098	5.5201	9.0161	0	0	5.7948
Kif18a	-2.0995	4.5757	-9.0156	0	0	5.9581
Fam53b	2.0361	4.4908	9.0097	0	0	5.9004
Rsu1	1.1939	5.948	9.0093	0	0	5.7687
Akap13	0.9704	5.8748	9.0033	0	0	5.7664
Gsr	-1.6537	7.2048	-9.0016	0	0	5.734
Bckdha	1.0301	5.6159	8.9941	0	0	5.7668
Hist1h2ak	-3.235	1.174	-8.9884	0	0	5.3189
Lmnb2	-1.5329	4.8776	-8.9818	0	0	5.8586
Prkg2	-3.7958	5.5084	-8.9762	0	0	5.9284
Zfyve1	1.9259	5.5371	8.9646	0	0	5.7476
Mcm8	-1.4596	3.9202	-8.9583	0	0	5.9003
Nup54	-1.0494	6.0536	-8.9456	0	0	5.6973
Podxl	-1.1028	6.9003	-8.9379	0	0	5.6583
Ect2	-2.2091	6.7268	-8.9337	0	0	5.6883
Pttg1	-1.7284	5.3328	-8.9289	0	0	5.7553
Lima1	1.2971	6.8516	8.9268	0	0	5.6369

Ppat	-1.2895	6.6524	-8.9267	0	0	5.6564
Ccdc77	-1.0317	4.3519	-8.9244	0	0	5.8053
4931406P16Rik	1.1666	5.9811	8.9181	0	0	5.6553
Cdon	2.5416	4.3229	8.9108	0	0	5.8214
Il6st	1.1122	8.4579	8.9062	0	0	5.593
Hspa14	-0.9612	6.382	-8.9002	0	0	5.6245
Nxt1	-0.9968	4.8909	-8.897	0	0	5.7242
Prim2	-1.0712	5.2121	-8.8962	0	0	5.6976
Spout1	-1.0214	5.3607	-8.8924	0	0	5.6788
Rbmx2	-1.375	4.4021	-8.8816	0	0	5.7631
Peak1	-1.3615	8.3824	-8.879	0	0	5.5636
Cnot6	-0.8624	8.1977	-8.8789	0	0	5.5642
Mlkl	-1.4832	5.3542	-8.8785	0	0	5.6805
Gm6682	-1.8816	3.8592	-8.876	0	0	5.8194
Mtbp	-1.494	5.3531	-8.8731	0	0	5.6751
Ddx11	-1.3872	4.707	-8.8677	0	0	5.7303
Rnf141	-1.071	4.3998	-8.8664	0	0	5.733
mt-Cytb	0.8406	11.3058	8.8529	0	0	5.5153
H3f3a	0.8351	7.6204	8.8485	0	0	5.5292
Bora	-2.0122	4.6505	-8.8475	0	0	5.7453
Bcl9	1.0626	5.9787	8.8461	0	0	5.5662
Slc19a1	-1.1493	4.9875	-8.8378	0	0	5.6547
Mfsd1	1.0325	8.0318	8.8358	0	0	5.5093
Natd1	1.3465	4.8431	8.8341	0	0	5.6362
Nudc	-0.8483	7.46	-8.8315	0	0	5.5132
Ldlrap1	-2.0231	4.8186	-8.8308	0	0	5.708
Vps11	0.9373	6.4512	8.8171	0	0	5.5103
Ndst3	-4.6377	1.6811	-8.8156	0	0	4.8979
Ccnd3	-0.9787	6.5603	-8.8142	0	0	5.5115
Arhgap18	-1.9998	5.6507	-8.8127	0	0	5.6028
Mthfd1	-1.3327	7.2636	-8.8033	0	0	5.4853
Auts2	1.5756	4.9448	8.8027	0	0	5.595
Ilf2	-0.9269	7.4208	-8.7923	0	0.0001	5.4658
Impdh2	-1.0563	8.3109	-8.7755	0	0.0001	5.4349
Csf3	-5.4095	5.8023	-8.7598	0	0.0001	5.6885
Snapin	0.7624	5.8501	8.7547	0	0.0001	5.4527
Parp4	1.1121	5.3219	8.7525	0	0.0001	5.4892
P2rx5	4.2174	0.9362	8.7501	0	0.0001	4.9969
Zc2hc1a	1.6329	4.8315	8.7419	0	0.0001	5.5362
Uck2	-1.0931	7.853	-8.7369	0	0.0001	5.3922
St5	2.3819	5.5931	8.7272	0	0.0001	5.4647
Pop1	-1.5168	6.0634	-8.7201	0	0.0001	5.4372
Gar1	-1.3806	6.1101	-8.7196	0	0.0001	5.4261
Flcn	1.1038	5.4462	8.6984	0	0.0001	5.4126
N4bp3	-1.8968	3.5496	-8.6878	0	0.0001	5.6056
Nav3	-1.2568	4.4774	-8.6874	0	0.0001	5.5256
Parp9	2.57	4.5789	8.6867	0	0.0001	5.5274
Rnps1	-0.9365	5.7215	-8.6861	0	0.0001	5.3901
Orc6	-1.3903	5.8499	-8.6835	0	0.0001	5.3983
Ano6	0.9158	7.494	8.6818	0	0.0001	5.3235
Mcoln1	1.3394	5.0766	8.6619	0	0.0001	5.4017
March2	1.4733	4.3122	8.6597	0	0.0001	5.4712

Mkrn1	1.1323	5.4622	8.6546	0	0.0001	5.3536
Ifngr2	1.3281	5.7987	8.6498	0	0.0001	5.3313
Palb2	-1.529	3.2322	-8.6476	0	0.0001	5.5612
Xpot	-0.9002	7.4918	-8.6451	0	0.0001	5.281
Htra1	1.5542	8.2013	8.6339	0	0.0001	5.2554
Pqlc2	1.5239	2.2766	8.6302	0	0.0001	5.5402
Kank2	1.5294	6.514	8.6218	0	0.0001	5.2683
4930471E19Rik	-2.8892	2.3079	-8.6101	0	0.0001	5.3913
Clcn4	1.065	5.1776	8.5954	0	0.0001	5.3102
Tprgl	0.8388	5.3194	8.5922	0	0.0001	5.2827
Hus1	-1.0492	4.8499	-8.5822	0	0.0001	5.3408
Fam214a	2.4494	3.8138	8.5705	0	0.0001	5.4403
Ran	-1.2193	9.0938	-8.5682	0	0.0001	5.1681
Bmf	5.8745	3.393	8.5647	0	0.0001	5.3806
Bpnt1	-1.1415	5.5685	-8.5645	0	0.0001	5.2613
Eif2s2	-1.0749	7.9575	-8.5625	0	0.0001	5.1708
Aprt	-1.3443	7.1659	-8.5569	0	0.0001	5.177
Syne3	-3.3714	4.5567	-8.5472	0	0.0001	5.4413
Lsm2	-1.0583	5.7403	-8.5376	0	0.0001	5.2077
Tspan17	1.9553	4.2794	8.5332	0	0.0001	5.3358
Ankrd27	-0.8817	5.4441	-8.5257	0	0.0001	5.211
Rabggtb	-0.841	6.6725	-8.519	0	0.0001	5.1352
Eef1e1	-1.4947	5.966	-8.5172	0	0.0001	5.1859
Pcnt	-1.1363	5.861	-8.5045	0	0.0001	5.1647
Ube2q2	-0.9198	6.0342	-8.4944	0	0.0001	5.1354
Zfp672	1.1428	3.5689	8.4903	0	0.0001	5.3313
Cbx3	-0.694	6.5579	-8.4902	0	0.0001	5.1021
Ift122	1.2637	4.4465	8.4895	0	0.0001	5.2417
Fbxl20	1.246	5.8401	8.4887	0	0.0001	5.1274
Pi4k2b	-1.1515	6.5375	-8.4885	0	0.0001	5.1057
Serbp1	-0.8909	9.6715	-8.4847	0	0.0001	5.0576
Gtf2e2	-0.9674	6.3039	-8.482	0	0.0001	5.1031
Sipa1l3	-1.108	6.8284	-8.4745	0	0.0001	5.0802
Cnn2	1.6249	7.2669	8.4722	0	0.0001	5.0609
Bzw2	-0.7189	7.2993	-8.4638	0	0.0001	5.0526
Bcat1	-2.4906	5.9094	-8.4538	0	0.0001	5.16
Tuba1b	-1.8612	7.4907	-8.453	0	0.0001	5.0446
Lactb	1.6439	4.0295	8.4526	0	0.0001	5.2535
E2f4	-1.1737	6.5413	-8.4486	0	0.0001	5.0563
1810058I24Rik	1.7007	4.9396	8.4387	0	0.0001	5.1432
Fbxo32	4.1502	3.9626	8.436	0	0.0001	5.3088
Efl1	-1.066	5.5989	-8.4355	0	0.0001	5.0885
Glul	1.7711	7.9823	8.4225	0	0.0001	4.9891
Dkc1	-1.2469	7.4431	-8.4171	0	0.0001	4.9945
Dna2	-2.1838	3.9357	-8.4032	0	0.0001	5.2601
Dtx4	2.7491	6.8608	8.3996	0	0.0001	4.9867
Eif4e	-1.0042	7.7335	-8.3914	0	0.0001	4.9556
Psap	1.2363	9.5805	8.3873	0	0.0001	4.9317
Cdr2	-1.3882	4.7151	-8.3863	0	0.0001	5.1283
Nt5c2	1.0428	5.1265	8.3776	0	0.0001	5.0342
Tmem107	-1.2966	3.7244	-8.3592	0	0.0001	5.1757
Tcp1	-0.8692	9.3258	-8.3565	0	0.0001	4.8945

Wdtc1	1.2108	6.0568	8.348	0	0.0001	4.9345
Tcof1	-1.6081	7.3773	-8.3463	0	0.0001	4.9073
Ddx21	-1.0017	8.041	-8.344	0	0.0001	4.8907
Nfe2l1	0.9912	8.6502	8.3431	0	0.0001	4.8796
Susd6	1.3829	7.0456	8.3422	0	0.0001	4.8986
mt-Nd5	1.0914	10.2284	8.3385	0	0.0001	4.8667
Fam72a	-1.3634	2.6267	-8.3347	0	0.0001	5.1903
Fam78b	-3.3882	2.5344	-8.3321	0	0.0001	5.0475
Spred3	-2.4559	4.1979	-8.3294	0	0.0001	5.165
Gnao1	1.3291	6.5588	8.326	0	0.0001	4.8904
Mpv17	0.8548	5.838	8.3216	0	0.0001	4.9084
Slco4a1	-3.1476	5.6971	-8.3204	0	0.0001	5.061
Tnfrsf12a	-1.1717	7.5829	-8.3201	0	0.0001	4.8653
Cdv3	-0.9453	8.1778	-8.316	0	0.0001	4.8524
Pcolce	1.2548	8.388	8.31	0	0.0001	4.8393
Grk6	-1.0725	6.7778	-8.308	0	0.0001	4.8662
Pkn3	-2.1943	3.1648	-8.3075	0	0.0001	5.1579
Eri1	-0.9837	6.925	-8.3073	0	0.0001	4.8611
Nsun2	-1.2517	8.6356	-8.3066	0	0.0001	4.8364
Gen1	-1.8231	4.8204	-8.3029	0	0.0001	5.0494
Ldha	-1.0303	10.6239	-8.301	0	0.0001	4.8174
Phactr1	3.6406	3.3555	8.3	0	0.0001	5.1485
Cnot9	-0.9025	6.8726	-8.2965	0	0.0001	4.8461
Syncrip	-0.8925	8.2251	-8.2944	0	0.0001	4.8237
Ivns1abp	-0.8791	7.7771	-8.2927	0	0.0001	4.8269
Esco2	-2.6206	4.2976	-8.2917	0	0.0001	5.1255
Ifnar1	0.8252	6.5293	8.29	0	0.0001	4.8395
Tubg1	-0.9659	6.2357	-8.2883	0	0.0001	4.8587
Tcf19	-1.7884	4.6543	-8.2845	0	0.0001	5.0313
Naf1	-1.4286	5.4818	-8.2837	0	0.0001	4.9314
Rmi2	-3.6451	1.9355	-8.2696	0	0.0001	4.7701
Mcph1	-0.9682	5.6467	-8.2685	0	0.0001	4.8708
Vps53	0.8973	5.4387	8.2658	0	0.0001	4.8596
Hes6	1.1167	4.5067	8.2593	0	0.0001	4.9348
Ddias	-1.6979	3.7576	-8.2554	0	0.0001	5.0682
Timm9	-1.1854	4.8359	-8.2542	0	0.0001	4.9397
G2e3	-1.1794	5.677	-8.2479	0	0.0001	4.8538
Tapbp	1.9745	7.8517	8.2478	0	0.0001	4.7646
Alyref	-1.1481	7.4376	-8.2476	0	0.0001	4.7735
Oaf	-1.7785	7.9027	-8.2439	0	0.0001	4.7644
Slc49a4	1.4402	4.5812	8.2415	0	0.0001	4.9172
Nomo1	-0.9352	8.0245	-8.241	0	0.0001	4.7562
Inpp5k	1.1417	4.7631	8.2403	0	0.0001	4.8918
Pfdn6	-0.9451	5.6691	-8.2367	0	0.0001	4.8251
Zkscan3	0.9142	5.3696	8.2348	0	0.0001	4.826
Rfx5	1.0846	4.9363	8.2307	0	0.0001	4.8678
Irgm1	1.936	4.0601	8.2306	0	0.0001	4.9786
Eif5a	-1.1925	10.194	-8.2243	0	0.0001	4.719
Cycs	-1.1739	6.3018	-8.2212	0	0.0001	4.7719
Cnppd1	1.0641	5.8669	8.2159	0	0.0001	4.7707
Hnrnpa3	-0.906	9.1501	-8.2151	0	0.0001	4.7117
Gm14150	-1.7042	4.695	-8.2137	0	0.0001	4.939

Nans	-0.6899	6.6063	-8.212	0	0.0001	4.7401
Lamp1	0.9496	9.6495	8.2098	0	0.0001	4.7007
Zbtb20	1.9664	5.4806	8.2093	0	0.0001	4.8104
Usp31	-1.0359	5.7348	-8.2081	0	0.0001	4.79
Ar	2.1449	4.8674	8.2015	0	0.0001	4.8759
Dazap1	-0.8414	6.9054	-8.2008	0	0.0001	4.72
Tcf7l1	1.7737	3.5448	8.1984	0	0.0001	4.9758
Krr1	-0.8174	5.8316	-8.1984	0	0.0001	4.7639
Slc7a1	-1.2615	6.9642	-8.1921	0	0.0001	4.7126
Polr2h	-0.9791	4.9585	-8.1876	0	0.0001	4.8258
Cenps	-1.53	2.8097	-8.1831	0	0.0001	5.0063
Snrpe	-0.8607	6.7015	-8.1811	0	0.0001	4.6995
Mki67	-2.0934	8.1103	-8.1806	0	0.0001	4.6826
Dusp4	-2.9671	5.9321	-8.179	0	0.0001	4.8408
Aldh6a1	1.8573	4.3831	8.1755	0	0.0001	4.8723
Scarb2	1.1996	6.0111	8.1722	0	0.0001	4.7058
Coro1b	1.0467	6.7817	8.1651	0	0.0001	4.6701
March8	0.6519	6.5232	8.1609	0	0.0001	4.6712
Phldb2	3.9119	4.5744	8.144	0	0.0001	4.9003
Prkar2a	-0.9686	7.0933	-8.1437	0	0.0001	4.6435
Nup133	-1.0625	6.551	-8.1411	0	0.0001	4.656
Txn1	-0.8764	8.6542	-8.1403	0	0.0001	4.6171
Lsm11	-1.0843	4.1448	-8.1362	0	0.0001	4.8531
Prpf4	-0.7772	5.5342	-8.1321	0	0.0001	4.6997
Irs1	3.2046	3.0471	8.1244	0	0.0001	4.935
Psd3	1.9415	3.6009	8.1218	0	0.0001	4.8898
Fut8	-1.0762	6.2033	-8.1195	0	0.0001	4.6408
Cacybp	-1.1458	7.5977	-8.1168	0	0.0001	4.6001
Mvp	1.4204	6.7126	8.1126	0	0.0001	4.6049
Angptl2	-1.3637	7.4786	-8.1082	0	0.0001	4.5907
Mastl	-1.732	5.1811	-8.107	0	0.0001	4.744
Rrp1b	-1.2814	5.9936	-8.0998	0	0.0001	4.6392
Impa2	-1.3891	4.7327	-8.0926	0	0.0001	4.7459
Zscan21	1.4291	4.1755	8.0805	0	0.0001	4.7459
Fuca1	1.3183	4.9832	8.0804	0	0.0001	4.6634
Snrpd1	-1.2653	6.988	-8.0767	0	0.0001	4.5593
Erbin	1.1203	8.0805	8.0729	0	0.0001	4.5316
Rnpepl1	0.7974	5.4965	8.0713	0	0.0001	4.6005
Tnip1	1.2315	6.4787	8.0703	0	0.0001	4.5553
Map2k3	-1.369	6.9323	-8.0678	0	0.0001	4.5502
Bad	0.8667	4.4611	8.0544	0	0.0001	4.6731
Stk11ip	1.0498	4.2479	8.0517	0	0.0001	4.7057
Pgm5	4.3408	-3.3087	8.049	0	0.0001	2.7961
Dlgap5	-1.7736	5.9952	-8.0459	0	0.0001	4.5861
Kcnk2	-2.1972	3.4869	-8.0449	0	0.0001	4.8313
Abhd4	1.2355	6.336	8.0423	0	0.0001	4.5239
Klhl24	2.1527	6.543	8.035	0	0.0001	4.5156
Steap4	4.2494	3.4761	8.035	0	0.0001	4.8246
Narf	0.9473	5.8934	8.0315	0	0.0001	4.5257
Pwp1	-0.8612	6.068	-8.0273	0	0.0001	4.5248
4930503L19Rik	-1.1839	5.1198	-8.0272	0	0.0001	4.6133
Spire2	-1.51	3.268	-8.0242	0	0.0001	4.7919

Pvt1	-1.0821	8.1364	-8.0193	0	0.0001	4.4634
Kansl1	0.81	6.0627	8.0038	0	0.0001	4.4818
Rbm14	-0.7344	5.9062	-8.0033	0	0.0001	4.501
Golt1b	-0.7818	6.4952	-8.0009	0	0.0001	4.4667
Exosc7	-1.426	5.0281	-7.9927	0	0.0001	4.5864
Dimt1	-1.2305	4.8242	-7.9903	0	0.0001	4.5991
Aspm	-1.667	6.6114	-7.9898	0	0.0001	4.4721
Dag1	0.8208	8.4884	7.9893	0	0.0001	4.416
Gm26722	3.0616	0.3675	7.9874	0	0.0001	4.4336
Klc4	2.2349	3.9288	7.9843	0	0.0001	4.6845
Rpf1	-0.7689	6.0707	-7.9789	0	0.0001	4.4564
B3galnt2	-1.1247	6.2378	-7.9784	0	0.0001	4.4557
Slc2a1	-1.6554	7.3761	-7.9718	0	0.0001	4.4133
Kpnb1	-1.0064	9.7197	-7.9685	0	0.0001	4.3823
Eif2s1	-1.0386	7.7709	-7.9664	0	0.0001	4.397
Pcdhb16	2.1198	1.4423	7.9615	0	0.0001	4.6879
Impdh1	-1.2649	6.0039	-7.9607	0	0.0001	4.4518
Nusap1	-1.7631	6.076	-7.9514	0	0.0001	4.456
Nup35	-1.4847	4.1353	-7.9483	0	0.0001	4.6316
Timp2	1.8921	9.7267	7.9458	0	0.0001	4.3506
Ppil1	-1.4022	5.8391	-7.9454	0	0.0001	4.4495
Nop56	-1.3153	7.6906	-7.9453	0	0.0001	4.3716
Ptp4a2	-0.7679	8.0363	-7.9443	0	0.0001	4.3628
Ubash3b	-2.4279	1.9795	-7.942	0	0.0001	4.6448
Scrn1	2.6913	4.9222	7.9301	0	0.0001	4.516
Slc15a2	1.8901	3.6048	7.927	0	0.0001	4.6368
Mtfr2	-1.8772	3.3913	-7.924	0	0.0001	4.6763
Lgmn	1.1302	7.0232	7.9215	0	0.0001	4.3422
Fxyd5	-3.1826	4.2208	-7.9204	0	0.0001	4.6684
Parp14	3.2496	3.919	7.9187	0	0.0001	4.6388
Galc	1.4342	4.8744	7.9166	0	0.0001	4.4607
Slc20a2	-0.9987	6.5952	-7.906	0	0.0001	4.3407
Slc31a2	1.5715	3.0942	7.9058	0	0.0001	4.6292
Slco2a1	-2.8528	7.6786	-7.9037	0	0.0001	4.337
Ddx39	-1.4339	6.8076	-7.9013	0	0.0001	4.3345
Ssr1	-1.145	8.889	-7.8943	0	0.0001	4.2885
Aff1	0.9323	6.3575	7.8929	0	0.0001	4.3211
Tmem109	-0.9019	6.1691	-7.8921	0	0.0001	4.3395
Ptges3	-1.1655	7.5008	-7.8903	0	0.0001	4.2993
Herc6	1.4331	3.331	7.8873	0	0.0001	4.5929
S1pr2	1.1187	6.6347	7.8856	0	0.0001	4.3023
Bcl2l11	1.4618	5.3536	7.8851	0	0.0001	4.3806
Coro1c	-1.0015	7.8561	-7.8802	0	0.0001	4.2797
Pfas	-1.509	6.1531	-7.8791	0	0.0001	4.3464
Kif24	-1.5883	3.0773	-7.8732	0	0.0001	4.6167
Calcoco1	2.1145	5.8719	7.8729	0	0.0001	4.3333
Dpy30	-1.1063	5.4742	-7.8723	0	0.0001	4.3656
Rbm5	0.6178	6.401	7.8638	0	0.0001	4.2792
Wbp11	-0.8347	7.2318	-7.8637	0	0.0001	4.2659
Mphosph6	-1.0994	4.8999	-7.8631	0	0.0001	4.4107
Pgp	-0.9488	5.7366	-7.857	0	0.0001	4.3187
Gpn3	-0.9125	5.3004	-7.852	0	0.0001	4.3482

Cp	1.9039	10.2055	7.8476	0	0.0001	4.2165
Slc29a3	1.8049	3.8173	7.8281	0	0.0001	4.4716
Ing5	-1.1192	5.1212	-7.8233	0	0.0001	4.3406
Tomm70a	-0.9283	8.0856	-7.8191	0	0.0001	4.1943
A330074K22Rik	3.657	2.3486	7.8139	0	0.0001	4.5176
Wdr36	-1.2186	7.4526	-7.8136	0	0.0001	4.1968
Ankrd12	1.163	5.6367	7.8118	0	0.0001	4.2506
Lrig2	0.9447	4.7208	7.8087	0	0.0001	4.3261
Nolc1	-1.2896	7.961	-7.8079	0	0.0001	4.182
Pms2	-1.0389	4.5804	-7.8049	0	0.0001	4.3642
Ripk2	1.2967	4.3998	7.8031	0	0.0001	4.3583
Tgfbrap1	0.9021	5.2742	7.8019	0	0.0001	4.2618
Slc26a2	1.4797	5.5682	7.8003	0	0.0001	4.246
Decr2	1.2733	4.6115	7.7947	0	0.0001	4.3235
Zgrf1	-1.2711	3.6504	-7.7935	0	0.0001	4.4634
Zfp948	-1.4993	4.246	-7.7928	0	0.0001	4.4213
Atp6v0b	1.0385	6.3682	7.7927	0	0.0001	4.1846
Skap2	1.5313	5.1792	7.7719	0	0.0001	4.2413
Snx18	1.3532	6.8414	7.7709	0	0.0001	4.1436
Wbp1l	1.0444	6.7522	7.7599	0	0.0001	4.1296
Cct6a	-1.005	9.0615	-7.7578	0	0.0001	4.1013
Arf6	-0.6546	7.4436	-7.7577	0	0.0001	4.1179
Atic	-1.0805	7.8638	-7.7558	0	0.0001	4.1113
Rpa3	-1.1692	4.8743	-7.747	0	0.0001	4.2532
Os9	1.2497	5.9469	7.7329	0	0.0001	4.1233
Sox4	2.0513	8.6709	7.731	0	0.0001	4.0661
Mrpl12	-1.0247	6.8874	-7.7278	0	0.0001	4.0898
Ankrd37	-1.612	3.2487	-7.7234	0	0.0001	4.4106
Irgq	1.4431	5.005	7.7141	0	0.0001	4.1762
Ahctf1	-0.7683	7.5829	-7.7115	0	0.0001	4.0535
Pnrc1	0.9884	7.0567	7.707	0	0.0001	4.0515
Tomm40	-1.4246	6.74	-7.6953	0	0.0001	4.0573
Brip1	-1.7444	4.4862	-7.6932	0	0.0001	4.2776
Ebna1bp2	-1.1431	6.6465	-7.6915	0	0.0001	4.0498
Ttc17	0.9484	4.7702	7.6865	0	0.0001	4.1545
Fbl	-1.2692	6.6291	-7.6856	0	0.0001	4.0453
Pgam1	-0.8102	7.835	-7.6855	0	0.0001	4.0139
Timp1	-1.3234	6.4097	-7.6802	0	0.0001	4.0523
Ruvbl1	-1.0787	6.7553	-7.6786	0	0.0001	4.0273
Stx2	0.8307	4.737	7.6735	0	0.0001	4.1369
Clec2d	2.5307	2.2486	7.6731	0	0.0001	4.3701
Snrpb	-0.7955	8.0482	-7.6693	0	0.0001	3.989
Nes	-2.0792	5.1213	-7.6643	0	0.0001	4.1861
Vwa5a	2.6316	5.3721	7.6587	0	0.0001	4.1183
Pafah2	1.2969	3.7911	7.6557	0	0.0001	4.2257
Dync2li1	0.9662	4.5857	7.6523	0	0.0001	4.1227
Cad	-1.6976	7.8938	-7.6499	0	0.0001	3.9691
Gas2	-1.5798	1.8703	-7.648	0	0.0001	4.3279
Slc25a5	-1.2653	8.9123	-7.6454	0	0.0001	3.9491
Plscr3	1.4604	5.7367	7.6439	0	0.0001	4.0182
Ostm1	1.4199	4.7813	7.6432	0	0.0001	4.1051
Cct8	-0.8256	8.8428	-7.6421	0	0.0001	3.9437

Znfx1	1.7728	4.6271	7.6418	0	0.0001	4.1315
Atg14	1.5575	4.2832	7.6337	0	0.0001	4.152
Ugdh	-0.8857	6.8341	-7.6284	0	0.0001	3.9524
Lsm4	-1.066	6.1592	-7.6283	0	0.0001	3.9814
Slc22a15	-1.361	3.8192	-7.6253	0	0.0001	4.2231
Isy1	-0.7099	5.4347	-7.6252	0	0.0001	4.0228
Myl6	1.1213	7.6817	7.6251	0	0.0001	3.929
Mtmr3	0.8227	7.1674	7.623	0	0.0001	3.9337
Sulf2	2.4918	6.4946	7.6224	0	0.0001	3.9658
Irf1	1.5722	6.3444	7.6219	0	0.0001	3.9604
Washc1	0.8527	5.6114	7.621	0	0.0001	3.9831
Dstyk	1.5406	5.0536	7.6197	0	0.0001	4.0439
Dctpp1	-1.5533	5.4753	-7.6061	0	0.0001	4.0308
Magi3	1.1947	5.4684	7.6038	0	0.0001	3.9825
Polr1b	-1.027	6.1276	-7.6021	0	0.0001	3.9531
Bphl	1.5128	3.7968	7.6007	0	0.0001	4.1633
Polr2c	-0.6867	6.2301	-7.6004	0	0.0001	3.9337
Snrnp40	-1.1015	5.9399	-7.5898	0	0.0001	3.9443
Uba2	-1.0594	7.5573	-7.5898	0	0.0001	3.8873
Tent5a	3.2339	2.6235	7.5853	0	0.0001	4.2567
Depdc1a	-2.6877	4.1273	-7.5837	0	0.0001	4.2307
Yipf2	1.045	4.2163	7.583	0	0.0001	4.0798
Lrp8	-2.3317	6.0483	-7.5808	0	0.0001	3.9903
Mcmbp	-0.8359	6.6028	-7.5806	0	0.0001	3.8948
Nedd1	-0.8067	5.2824	-7.5791	0	0.0001	3.9761
Crim1	1.0678	5.6383	7.5726	0	0.0001	3.9228
Padi4	-2.1266	5.7746	-7.5722	0	0.0001	3.98
Yars	-0.9068	6.7802	-7.564	0	0.0001	3.8642
Ptms	1.0502	8.3203	7.5611	0	0.0001	3.8337
Terf1	-1.003	4.7451	-7.56	0	0.0001	4.0176
Zfp316	1.4225	3.3949	7.5567	0	0.0001	4.1415
Stx5a	0.7681	6.0352	7.5561	0	0.0001	3.8732
Hexb	1.6289	6.1318	7.551	0	0.0001	3.8691
Gm4737	-1.1728	5.0713	-7.5459	0	0.0001	3.9714
Tmem19	1.6522	5.1423	7.5427	0	0.0001	3.9353
Plxna1	1.1732	7.9567	7.5396	0	0.0001	3.8081
Timm50	-0.857	5.5769	-7.5368	0	0.0001	3.8918
Wdr18	-0.7498	5.7042	-7.5366	0	0.0001	3.876
Rabepk	-0.894	4.7904	-7.5355	0	0.0001	3.9595
Crlf3	-0.93	5.6082	-7.5331	0	0.0001	3.8836
Rbms3	2.3191	1.4671	7.5302	0	0.0001	4.1544
Spred1	-1.9328	6.7765	-7.5277	0	0.0001	3.8414
Pidd1	-1.8772	2.2019	-7.5244	0	0.0002	4.1701
Atp6ap1	0.8186	7.7832	7.5219	0	0.0002	3.785
Set	-1.1654	9.3353	-7.5201	0	0.0002	3.772
Ap1s1	-0.7603	6.9285	-7.5157	0	0.0002	3.7932
Plaur	-3.1534	5.5197	-7.5153	0	0.0002	4.0352
Tdp1	-1.0992	4.8455	-7.513	0	0.0002	3.9405
Irf2bpl	1.1805	6.7621	7.5074	0	0.0002	3.7833
Pkd1	0.9047	6.4165	7.5064	0	0.0002	3.7913
Trip4	0.7363	5.4888	7.5063	0	0.0002	3.8331
Utp11	-1.1705	6.3839	-7.5029	0	0.0002	3.8001

Pdk2	2.5978	3.1129	7.5013	0	0.0002	4.1261
Vps8	1.4286	3.9875	7.5005	0	0.0002	4.0064
Uxs1	-0.7659	6.3229	-7.4978	0	0.0002	3.7882
Gm7993	-2.9063	5.1938	-7.4955	0	0.0002	4.0166
Bach1	0.9906	5.8865	7.4898	0	0.0002	3.7925
Spns2	2.6927	3.2667	7.4878	0	0.0002	4.1065
Bbs12	-2.0157	4.4038	-7.4833	0	0.0002	4.0173
Phf10	-0.8019	6.5756	-7.4809	0	0.0002	3.7542
Slc19a2	-1.1325	5.1162	-7.4805	0	0.0002	3.8722
Srpk1	-0.7117	7.0021	-7.4781	0	0.0002	3.7397
4933404O12Rik	-1.2293	4.3985	-7.4707	0	0.0002	3.9526
Amy1	4.4736	-1.8865	7.4614	0	0.0002	2.479
1810055G02Rik	-0.8567	5.3697	-7.4604	0	0.0002	3.8035
Hbp1	1.3679	6.8003	7.4585	0	0.0002	3.7133
AI429214	2.6972	1.3067	7.4523	0	0.0002	4.022
Etaa1	-1.3196	4.4448	-7.4516	0	0.0002	3.9292
Mrgprf	2.7998	3.6601	7.4462	0	0.0002	4.0143
Zswim8	1.0419	6.8949	7.4443	0	0.0002	3.6902
Il11ra1	1.4486	3.3649	7.4405	0	0.0002	3.9806
Ptbp1	-0.7951	8.9642	-7.4398	0	0.0002	3.6613
Zfp579	1.2849	2.774	7.4325	0	0.0002	4.0231
2010204K13Rik	-2.2991	3.1222	-7.4288	0	0.0002	4.051
Sfxn5	-1.7595	3.4321	-7.4275	0	0.0002	4.0159
Lipt2	-2.2294	2.414	-7.4213	0	0.0002	4.044
Frmd4a	-1.0137	6.9033	-7.4209	0	0.0002	3.6657
Ctdsp2	1.1615	6.8937	7.4131	0	0.0002	3.647
Cstf2	-1.1367	6.928	-7.4072	0	0.0002	3.6477
Larp1	-0.7994	8.6094	-7.4034	0	0.0002	3.6133
Lipg	-1.9864	4.511	-7.3954	0	0.0002	3.8744
Rpp40	-1.096	3.8733	-7.391	0	0.0002	3.8888
Mycbp	-1.3624	4.1132	-7.39	0	0.0002	3.8772
Tarbp1	-1.3266	4.9127	-7.3885	0	0.0002	3.7909
Zkscan1	0.9937	5.8261	7.3802	0	0.0002	3.6436
Dtx3l	2.707	4.3311	7.3782	0	0.0002	3.864
Atp6v0e2	2.2314	3.1206	7.3761	0	0.0002	3.9475
Wdr5	-0.8342	6.5891	-7.3716	0	0.0002	3.6022
Vgll4	1.2047	6.1913	7.3703	0	0.0002	3.6087
Uaca	-1.1351	6.7343	-7.3639	0	0.0002	3.5884
Ehd4	-0.9836	6.4746	-7.3562	0	0.0002	3.5861
Yipf1	0.9523	4.0983	7.354	0	0.0002	3.7697
Ddah2	1.3366	5.3869	7.3505	0	0.0002	3.628
Snta1	2.4247	4.6164	7.3475	0	0.0002	3.7488
Slc1a5	-1.5428	6.2071	-7.3411	0	0.0002	3.5963
Sinhcaf	-0.8829	4.4922	-7.341	0	0.0002	3.7363
Snx19	0.8202	5.5062	7.3378	0	0.0002	3.5989
Tapbpl	2.0896	3.4732	7.3377	0	0.0002	3.8641
Mzt1	-0.9829	5.4647	-7.3377	0	0.0002	3.6298
Orc2	-1.1082	6.4336	-7.3359	0	0.0002	3.5657
Dlgap4	0.9779	5.8974	7.3338	0	0.0002	3.568
Srgap1	3.6872	2.4311	7.3306	0	0.0002	3.9227
Gtf2a2	-0.9277	5.7122	-7.3285	0	0.0002	3.5895
Plk3	-2.1324	4.7817	-7.3226	0	0.0002	3.7549

Eri2	-1.0466	4.5278	-7.3219	0	0.0002	3.7206
Mdm1	-1.0231	3.9827	-7.3212	0	0.0002	3.7732
Klf5	-1.5019	5.5855	-7.3183	0	0.0002	3.6196
Ppp2r2a	-1.1047	7.2639	-7.3182	0	0.0002	3.5118
Cnbp	-1.035	9.0437	-7.316	0	0.0002	3.4865
Nacc2	2.3825	4.4409	7.3101	0	0.0002	3.7247
Sumf1	0.8002	5.9986	7.31	0	0.0002	3.5301
Mttp	1.2271	4.6525	7.3099	0	0.0002	3.6542
Hmgb1	-0.9471	7.6291	-7.309	0	0.0002	3.4917
Dis3	-1.1921	7.0272	-7.3064	0	0.0002	3.5027
Tctn2	1.0114	3.8579	7.3051	0	0.0002	3.7371
Add3	1.563	5.394	7.3043	0	0.0002	3.5753
Cenpc1	-0.7573	5.5414	-7.3022	0	0.0002	3.5642
Glb1l	1.4869	2.0045	7.3011	0	0.0002	3.8873
Wdfy1	1.0499	5.3657	7.2997	0	0.0002	3.5589
Jund	1.8471	7.4903	7.2994	0	0.0002	3.4754
Enah	0.8909	6.6578	7.299	0	0.0002	3.4921
Mrpl42	-1.0494	5.6564	-7.295	0	0.0002	3.5498
Pdss1	-2.079	3.6794	-7.2926	0	0.0002	3.84
Arvcf	2.5729	3.0635	7.2897	0	0.0002	3.8477
Zfp961	-1.3154	4.1932	-7.2858	0	0.0002	3.7238
Porcn	2.4073	2.7482	7.2839	0	0.0002	3.8453
Arhgap28	1.0051	5.598	7.2796	0	0.0002	3.5186
Fam234a	1.2374	5.6831	7.277	0	0.0002	3.506
Hnrnpu	-0.7277	9.9446	-7.2766	0	0.0002	3.4246
Ddx27	-0.8049	6.9897	-7.2732	0	0.0002	3.4513
Vps41	1.0179	6.4951	7.2682	0	0.0002	3.4515
Col6a1	1.5143	8.0906	7.267	0	0.0002	3.4236
Dvl3	0.9416	5.6809	7.2666	0	0.0002	3.4893
Ccne2	-2.3923	4.7235	-7.2661	0	0.0002	3.739
Ids	1.0422	5.2409	7.259	0	0.0002	3.5201
Arpc2	-0.7289	8.3512	-7.2565	0	0.0002	3.4069
Gm49774	-3.4528	2.8266	-7.2532	0	0.0002	3.8103
Rgp1	0.9928	4.8615	7.2469	0	0.0002	3.5275
Antxr1	1.7404	6.5552	7.2461	0	0.0002	3.4228
Fam49b	-1.3349	6.6318	-7.2411	0	0.0002	3.424
Slco3a1	-1.2059	5.2903	-7.2404	0	0.0002	3.5276
Stoml2	-0.8795	6.6425	-7.2403	0	0.0002	3.4142
Trib3	-1.7943	4.5182	-7.2385	0	0.0002	3.6334
Ttf2	-0.8236	5.8563	-7.2382	0	0.0002	3.4495
Fam136a	-0.9221	5.7906	-7.2337	0	0.0002	3.4493
Nptxr	-1.4846	6.1108	-7.2324	0	0.0002	3.4441
Ankrd26	-1.1189	4.9402	-7.2275	0	0.0002	3.5381
Tbccd1	0.9112	4.0345	7.2262	0	0.0002	3.5968
Noc2l	-1.0806	6.7935	-7.2208	0	0.0002	3.3845
Oaz2	0.6042	5.8831	7.2163	0	0.0002	3.4
Ssbp2	1.2765	4.703	7.2146	0	0.0002	3.519
Casp3	-1.3885	8.7802	-7.2142	0	0.0002	3.3445
Sync	4.2887	1.8062	7.2065	0	0.0002	3.6846
Nup188	-0.9087	7.5216	-7.2064	0	0.0002	3.3473
Med18	-1.3126	3.7043	-7.2062	0	0.0002	3.6666
Shisa4	1.6226	4.7238	7.2039	0	0.0002	3.5024

Aig1	-1.1616	4.3027	-7.2031	0	0.0002	3.5731
Homer1	-1.0681	5.684	-7.203	0	0.0002	3.4247
Tube1	-1.6792	2.3362	-7.1974	0	0.0002	3.7528
Nuak2	2.0847	3.0949	7.1874	0	0.0002	3.6883
Selenoi	-0.9044	6.3118	-7.1816	0	0.0002	3.3459
Sgms2	-1.596	5.4807	-7.1787	0	0.0002	3.4353
Naa25	-1.0246	6.8583	-7.1784	0	0.0002	3.3221
Aida	-0.7445	6.3667	-7.1781	0	0.0002	3.3353
Pltp	3.2281	2.5808	7.1747	0	0.0002	3.7227
Ttc37	-0.814	6.5825	-7.1724	0	0.0002	3.3182
Prps1	-1.2163	6.6964	-7.1668	0	0.0002	3.3129
Brpf3	0.6849	5.5688	7.1655	0	0.0002	3.3518
Tgm2	2.808	7.7703	7.1633	0	0.0002	3.2801
Srsf2	-1.0415	8.1581	-7.1627	0	0.0002	3.2768
Clic1	-1.0596	8.321	-7.1594	0	0.0002	3.2685
Ap3m2	1.0136	3.4197	7.1523	0	0.0002	3.5696
Fzr1	-0.789	5.5728	-7.1501	0	0.0002	3.3401
Bub3	-0.6329	6.9757	-7.1399	0	0.0002	3.2594
Wwtr1	-0.6603	8.1451	-7.1389	0	0.0002	3.2405
Hspa9	-0.9148	9.9698	-7.1352	0	0.0002	3.2216
Hspd1-ps3	-1.2325	2.0687	-7.1342	0	0.0002	3.6667
Acad11	0.9921	4.731	7.133	0	0.0002	3.3827
Mlycd	1.715	2.5902	7.1329	0	0.0002	3.6357
Kat6b	1.3173	5.0736	7.132	0	0.0002	3.3539
Samhd1	0.5668	6.7123	7.1237	0	0.0002	3.2381
U2af1	-1.0615	6.878	-7.1236	0	0.0002	3.2422
Vps18	0.793	5.4835	7.1174	0	0.0002	3.2878
Slc25a37	-1.506	6.0342	-7.116	0	0.0002	3.2901
Ddx46	-0.7957	7.5114	-7.1152	0	0.0002	3.2154
Dcaf6	1.0908	6.0306	7.1114	0	0.0002	3.247
H2-T23	2.8441	2.5916	7.1107	0	0.0002	3.6362
Mogs	-1.1537	6.8278	-7.1095	0	0.0002	3.2251
Trappc9	0.8504	5.3816	7.1078	0	0.0002	3.2799
Spsb2	1.6646	3.9368	7.1069	0	0.0002	3.4582
Zfp646	0.798	5.5799	7.1053	0	0.0002	3.2661
Dlst	-0.7225	7.6316	-7.1046	0	0.0002	3.1978
Psph	-1.3169	5.122	-7.1033	0	0.0002	3.3297
Dars2	-0.8259	4.5849	-7.103	0	0.0002	3.3705
Cipc	1.0704	5.1665	7.1029	0	0.0002	3.3002
Sspn	1.5463	5.1399	7.1006	0	0.0002	3.3051
Tufm	-0.7244	6.5677	-7.096	0	0.0002	3.2061
Pdlim5	1.6321	7.7654	7.0949	0	0.0002	3.1792
Tex264	0.7388	5.5212	7.0938	0	0.0002	3.2472
Lnpk	-0.8061	4.6312	-7.0933	0	0.0002	3.368
1700052K11Rik	1.2535	3.2491	7.0932	0	0.0002	3.5101
Lrrk2	-1.7504	6.5076	-7.0926	0	0.0002	3.2235
Crebrf	1.7244	4.6453	7.092	0	0.0002	3.374
Dnajc2	-1.0366	7.3143	-7.0872	0	0.0002	3.1795
Slc46a1	1.5852	2.1963	7.0829	0	0.0002	3.5921
Wars	-0.6484	6.6633	-7.0794	0	0.0002	3.1788
Trim21	1.8013	2.1066	7.0792	0	0.0002	3.5901
Wdsub1	1.0801	3.7884	7.0789	0	0.0002	3.4218

Cdkn3	-1.8294	3.6494	-7.0769	0	0.0002	3.5182
Hpcal1	-0.9527	5.7405	-7.0767	0	0.0002	3.2216
Arhgef39	-2.1725	2.4735	-7.0766	0	0.0002	3.5928
Sult1a1	4.9706	-2.5553	7.0754	0	0.0002	1.9591
Gm5454	-3.1292	1.1095	-7.074	0	0.0002	3.3468
Snrk	0.922	4.3881	7.0687	0	0.0002	3.3355
Atp10a	-1.5424	4.6642	-7.0634	0	0.0002	3.3705
Dnph1	-1.5496	4.2265	-7.0627	0	0.0002	3.4146
Kctd1	1.2264	2.8378	7.0597	0	0.0002	3.5078
Dusp1	2.0013	4.9433	7.0583	0	0.0002	3.2929
Pnpt1	-0.8016	5.8564	-7.0553	0	0.0002	3.1859
Ago1	0.7075	6.2625	7.0552	0	0.0002	3.1516
Rad21	-0.789	8.8217	-7.0551	0	0.0002	3.1128
Xpo1	-1.369	8.4317	-7.0543	0	0.0002	3.118
Gab1	1.7257	4.1334	7.0512	0	0.0002	3.3769
Trak1	0.9883	6.1929	7.0429	0	0.0002	3.139
Ckap4	-0.7198	7.7304	-7.0415	0	0.0002	3.1053
Palld	1.1841	6.1731	7.0319	0	0.0002	3.1248
Mmp14	1.9327	7.4641	7.0313	0	0.0002	3.0905
Riok2	-0.7526	6.0684	-7.0261	0	0.0002	3.129
Glns-ps1	1.8879	2.4243	7.0227	0	0.0002	3.5041
Fbxo36	1.8116	2.3636	7.0148	0	0.0002	3.4911
Esf1	-0.8819	6.5557	-7.0136	0	0.0002	3.0897
Tns1	2.2853	6.1075	7.0133	0	0.0002	3.1257
Arhgap22	-1.3549	6.0124	-7.0078	0	0.0002	3.1268
Ccsap	-2.1096	3.0041	-7.0075	0	0.0002	3.481
Notch2	1.4844	7.5667	7.0018	0	0.0002	3.0466
Uchl5	-1.1397	6.5361	-6.9999	0	0.0002	3.0769
Pck2	-0.9077	5.7077	-6.9997	0	0.0002	3.1063
Usp35	1.275	2.7184	6.9977	0	0.0002	3.4269
Ganc	1.2386	2.685	6.9976	0	0.0002	3.4249
Eif1ad	-0.8548	5.9378	-6.9975	0	0.0002	3.097
Wdr90	-1.6009	4.04	-6.9961	0	0.0002	3.3532
Cnn3	0.679	8.5805	6.9938	0	0.0002	3.0228
Vps13c	1.0648	6.1798	6.99	0	0.0003	3.0648
Rabgap1	0.6182	6.7153	6.9877	0	0.0003	3.0396
Rtel1	-1.6421	5.6489	-6.9833	0	0.0003	3.1425
Fbxo25	0.9052	4.6403	6.9832	0	0.0003	3.1775
Zscan2	1.6254	2.765	6.9797	0	0.0003	3.4106
Eif2b3	-1.316	5.1521	-6.9766	0	0.0003	3.1564
Glb1	0.8102	5.5784	6.9756	0	0.0003	3.0736
Wdr4	-0.8355	5.105	-6.9749	0	0.0003	3.1399
Tcp11l2	1.6141	4.5957	6.9749	0	0.0003	3.2086
Msh6	-0.9075	7.1902	-6.9738	0	0.0003	3.0162
Ddx18	-1.0679	6.9695	-6.9732	0	0.0003	3.0221
Nme1	-0.9818	6.9025	-6.9726	0	0.0003	3.0201
Cth	-1.6236	3.981	-6.9696	0	0.0003	3.3142
Commd9	1.027	4.4138	6.966	0	0.0003	3.1944
Gm4204	-0.8389	4.1283	-6.9644	0	0.0003	3.2367
Ndufs2	0.7898	7.8132	6.9643	0	0.0003	2.9873
Ptges3-ps	-1.1672	3.2969	-6.9615	0	0.0003	3.3514
Plscr2	1.7341	3.644	6.9604	0	0.0003	3.3012

Tbc1d31	-1.3035	5.2357	-6.9591	0	0.0003	3.1199
Cep85	-0.6307	5.3536	-6.9552	0	0.0003	3.0772
6330403L08Rik	1.055	2.7554	6.9546	0	0.0003	3.3607
Tmub2	0.9769	5.1348	6.9543	0	0.0003	3.0802
Gstm1	1.5522	7.0439	6.9542	0	0.0003	2.9853
Dgkd	-1.0577	7.0457	-6.9537	0	0.0003	2.9895
Tra2b	-0.8292	6.8253	-6.952	0	0.0003	2.9927
Ccdc88c	1.2457	5.1799	6.9509	0	0.0003	3.091
Dzip1	1.5685	3.916	6.9478	0	0.0003	3.2429
Gnai2	0.6336	8.8769	6.9439	0	0.0003	2.9472
Gm20667	-2.3787	0.701	-6.9422	0	0.0003	3.222
Syt11	2.2828	3.7153	6.9402	0	0.0003	3.287
Zfp629	0.9092	3.761	6.9401	0	0.0003	3.2288
Vps35l	1.0226	6.3719	6.9378	0	0.0003	2.9775
Mrpl28	-0.7484	6.4376	-6.9361	0	0.0003	2.9775
Rin3	1.905	6.3696	6.9347	0	0.0003	2.9818
Cnih1	-0.6323	6.8335	-6.9318	0	0.0003	2.9596
Rasgef1a	3.3854	1.0911	6.9295	0	0.0003	3.3031
Mrps22	-1.1001	5.319	-6.924	0	0.0003	3.0504
Odf2	-0.7082	6.1872	-6.9228	0	0.0003	2.9691
Serpinb9	2.1583	3.8732	6.9223	0	0.0003	3.2295
Snn	1.5068	5.692	6.919	0	0.0003	2.9955
Lsm12	-0.8929	6.7799	-6.9177	0	0.0003	2.9431
Tef	1.2573	5.4104	6.9091	0	0.0003	2.9966
Pip4k2c	0.8204	5.6648	6.9054	0	0.0003	2.9654
Ccdc137	-1.3292	5.3985	-6.9038	0	0.0003	3.0362
Knstrn	-1.5861	6.2815	-6.9026	0	0.0003	2.9609
Neurl2	1.5084	2.0301	6.9025	0	0.0003	3.3502
Tcta	1.2745	2.9902	6.9001	0	0.0003	3.2632
Adck1	1.2348	3.9866	6.8983	0	0.0003	3.1444
Kdelr2	-0.7446	7.096	-6.8978	0	0.0003	2.9049
Mphosph10	-0.8785	5.8767	-6.8957	0	0.0003	2.9554
Srsf7	-1.1906	7.2256	-6.8936	0	0.0003	2.9005
Dynlt1f	-1.0629	3.9212	-6.8932	0	0.0003	3.1735
Zfp113	0.9613	3.5906	6.8909	0	0.0003	3.1761
Trappc12	0.8604	5.4242	6.8888	0	0.0003	2.9579
Tcn2	3.8557	4.7617	6.8845	0	0.0003	3.1606
Noct	-1.5242	5.6284	-6.8841	0	0.0003	2.9955
Psmd3	-0.9193	8.1059	-6.8837	0	0.0003	2.8691
Dnajb2	1.1735	4.9256	6.8811	0	0.0003	2.9923
Kmt5a	-0.7863	7.7588	-6.878	0	0.0003	2.8651
Gm48455	-2.6248	-0.163	-6.8739	0	0.0003	2.975
Sh3bp2	1.7298	5.4331	6.8731	0	0.0003	2.9505
Dscc1	-1.7831	3	-6.8705	0	0.0003	3.2873
Pgam5	-0.8347	6.0438	-6.8668	0	0.0003	2.9007
Wdpcp	1.5393	2.2039	6.8653	0	0.0003	3.2914
Mettl27	1.3783	3.475	6.8641	0	0.0003	3.1671
Atraid	1.0799	4.877	6.8624	0	0.0003	2.9728
E2f6	-0.7343	5.1171	-6.8613	0	0.0003	2.9689
Dpy19l1	-0.6917	7.0377	-6.8609	0	0.0003	2.8513
Tbc1d24	0.9702	4.3474	6.8605	0	0.0003	3.0415
Prune2	1.7448	6.9121	6.86	0	0.0003	2.8535

Cops3	-0.6812	6.6236	-6.8588	0	0.0003	2.857
Parp2	-1.2135	4.8897	-6.8574	0	0.0003	3.011
Gja1	-2.0675	6.609	-6.857	0	0.0003	2.8948
Fbxw5	0.688	5.3849	6.8563	0	0.0003	2.911
Mcur1	0.8783	5.485	6.8517	0	0.0003	2.9025
Plekhm1	0.9768	5.4241	6.8511	0	0.0003	2.9062
Clns1a	-0.854	6.6809	-6.8433	0	0.0003	2.8364
Abhd2	-0.7542	8.9809	-6.8414	0	0.0003	2.7974
Nsun5	-1.054	4.5636	-6.8398	0	0.0003	3.0186
Ppp5c	-1.0091	6.4031	-6.8386	0	0.0003	2.8415
Erh	-0.9163	6.6022	-6.8372	0	0.0003	2.8301
Nfyb	-0.6642	4.7234	-6.8334	0	0.0003	2.966
Add2	-2.9375	0.3622	-6.8312	0	0.0003	2.9903
Slc25a45	1.4603	2.4886	6.8311	0	0.0003	3.2116
Epc1	1.0097	5.6342	6.8306	0	0.0003	2.8617
Ppih	-0.9928	4.4955	-6.8262	0	0.0003	2.9978
Cenpm	-1.942	4.2183	-6.8257	0	0.0003	3.1119
Fanca	-1.8362	4.4911	-6.8251	0	0.0003	3.068
Tinf2	-0.7412	4.8944	-6.8242	0	0.0003	2.9317
Upp1	-4.8773	3.9981	-6.8219	0	0.0003	3.2312
Snx30	1.4578	4.3612	6.8162	0	0.0003	2.9845
Grk2	-0.8321	6.4316	-6.8125	0	0.0003	2.797
Tjap1	0.9355	6.0951	6.8106	0	0.0003	2.801
Abce1	-1.1149	8.5859	-6.8096	0	0.0003	2.7551
Fbxw9	0.9613	3.2689	6.809	0	0.0003	3.0957
Atp6v1b2	0.7365	7.1736	6.8084	0	0.0003	2.7668
Dbf4	-1.1589	6.8342	-6.8076	0	0.0003	2.7827
Ephb6	4.2565	3.3111	6.8076	0	0.0003	3.2184
Etfdh	0.9345	6.4406	6.8072	0	0.0003	2.7815
Cbx1	-0.6765	6.9699	-6.8046	0	0.0003	2.7685
Naglu	1.0614	5.1338	6.8002	0	0.0003	2.8549
Nup155	-0.8662	6.6959	-6.7999	0	0.0003	2.7739
Lin54	-1.1952	5.1486	-6.7973	0	0.0003	2.8985
Tfam	-0.8387	5.5152	-6.7966	0	0.0003	2.8356
Ifrd1	-0.9905	7.3955	-6.7959	0	0.0003	2.7491
Ska2	-0.853	5.592	-6.7922	0	0.0003	2.8183
Haus4	-1.0732	4.1995	-6.792	0	0.0003	3.0013
Nqo2	-1.1836	5.1142	-6.7917	0	0.0003	2.8779
Prep	-0.7091	7.3845	-6.7907	0	0.0003	2.7413
Tecpr1	1.2462	4.626	6.7907	0	0.0003	2.9036
Cat	1.044	5.8499	6.7905	0	0.0003	2.7853
Eprs	-0.776	8.8289	-6.7895	0	0.0003	2.7212
Eef1g	-0.7737	9.449	-6.7864	0	0.0003	2.7119
Nat10	-1.0001	5.8489	-6.7857	0	0.0003	2.801
Pigz	4.9349	-2.4265	6.7825	0	0.0003	1.7276
Fig4	0.8582	4.7431	6.7812	0	0.0003	2.8702
Acox3	1.8235	3.877	6.7811	0	0.0003	3.0156
Pcdhb17	2.1218	1.8501	6.7804	0	0.0003	3.1904
Nudc-ps1	-1.0656	3.5219	-6.7801	0	0.0003	3.0538
Nfkbie	2.3182	4.874	6.7786	0	0.0003	2.9076
Tnfrsf23	-1.4277	5.1946	-6.7775	0	0.0003	2.871
Ctns	2.3562	4.6649	6.7774	0	0.0003	2.9351

Cep152	-1.2898	3.7508	-6.7708	0	0.0003	3.0424
Pycr2	-0.8851	6.5288	-6.7633	0	0.0003	2.7228
Mis18a	-1.3001	4.1549	-6.7623	0	0.0003	2.967
Mctp2	-4.0011	1.0109	-6.7605	0	0.0003	2.7762
Gm9008	-0.8812	3.5782	-6.76	0	0.0003	3.0131
Smpd1	1.1988	5.1808	6.7588	0	0.0003	2.7925
Tm4sf1	-1.46	8.2977	-6.7576	0	0.0003	2.682
Snx27	-0.5894	7.5028	-6.7548	0	0.0003	2.6849
Rpn1	-0.6914	8.624	-6.7535	0	0.0003	2.6691
L2hgdh	-0.7997	5.0103	-6.7512	0	0.0003	2.8234
Ppid	-1.252	7.9203	-6.7455	0	0.0003	2.6682
Pmepa1	2.3772	4.4914	6.7433	0	0.0003	2.9071
Nop58	-1.3618	8.0814	-6.7429	0	0.0003	2.6627
Trim12c	2.1058	3.2189	6.7427	0	0.0003	3.0534
Idnk	1.601	3.7899	6.7426	0	0.0003	2.969
Msrb1	0.8773	4.9746	6.7419	0	0.0003	2.7808
Atp11a	0.9272	6.5919	6.7388	0	0.0003	2.677
Cgas	-1.8554	4.0052	-6.7379	0	0.0003	3.0101
Lzts2	1.6724	7.248	6.7378	0	0.0003	2.6598
Siah1a	0.9904	4.4657	6.7369	0	0.0003	2.837
mt-Rnr2	1.0578	11.0045	6.7329	0	0.0003	2.6257
Mast4	-1.3277	6.3687	-6.7323	0	0.0003	2.6974
Atxn1	1.2924	4.3356	6.7306	0	0.0003	2.8766
Junos	-2.0002	1.5744	-6.7304	0	0.0003	3.1104
Gm49336	-0.9378	5.9964	-6.7281	0	0.0003	2.7051
Snrpd2	-0.913	6.4956	-6.7279	0	0.0003	2.6691
Ipo7	-0.9594	8.5344	-6.7275	0	0.0003	2.6324
Zfp68	0.8932	5.5554	6.7256	0	0.0003	2.7084
Fzd2	1.5868	5.0421	6.7249	0	0.0003	2.7614
Eif4h	-0.6433	8.7597	-6.7211	0	0.0003	2.6193
Slf1	-1.5103	4.8648	-6.7196	0	0.0003	2.8426
Cpsf6	-0.6312	7.007	-6.7189	0	0.0003	2.6407
Trim56	1.1021	6.269	6.7168	0	0.0003	2.6568
Gm13398	4.7327	-2.8831	6.7133	0	0.0003	1.7407
Gart	-0.8956	7.3316	-6.7114	0	0.0003	2.6244
Nagk	1.445	4.0302	6.7082	0	0.0003	2.8589
Rbpj-ps3	-1.3359	4.8368	-6.7041	0	0.0003	2.8089
Mgat3	1.3296	6.0879	6.7013	0	0.0003	2.6462
Wdr12	-1.0689	6.0598	-6.7006	0	0.0003	2.66
Tbl3	-0.9985	6.1578	-6.7002	0	0.0003	2.649
Nmi	1.9109	3.1068	6.6969	0	0.0003	2.9983
Marcks	1.5777	6.3218	6.6969	0	0.0003	2.6305
Uvssa	1.1261	4.0636	6.6951	0	0.0003	2.8421
Rrp9	-1.2624	6.1914	-6.6945	0	0.0003	2.6471
Arsk	0.9404	3.4586	6.6917	0	0.0003	2.9091
Smc3	-0.8204	7.5364	-6.6917	0	0.0003	2.5908
Fam118b	0.8306	4.562	6.6917	0	0.0003	2.7624
Sphk1	3.9866	4.0811	6.6916	0	0.0003	2.979
Pcmtd1	1.2628	5.235	6.6908	0	0.0003	2.6913
Wdr43	-0.946	7.5679	-6.6883	0	0.0003	2.5866
Rsl1d1	-0.95	8.4639	-6.6843	0	0.0003	2.5683
Dclk1	2.8042	7.4176	6.6826	0	0.0003	2.5811

Sh3tc2	1.6456	5.4437	6.6755	0	0.0003	2.658
Rad9a	-0.8532	4.752	-6.6754	0	0.0003	2.7354
Ccndbp1	0.9157	4.9823	6.6734	0	0.0003	2.6806
Alg1	1.0234	4.1903	6.6727	0	0.0003	2.7777
Zfp14	2.7919	-0.8	6.665	0	0.0003	2.6834
Urb1	-1.0242	5.0613	-6.6646	0	0.0003	2.6989
Chchd4	-1.0958	5.3274	-6.6641	0	0.0003	2.6726
Lrsam1	0.9737	4.2341	6.6636	0	0.0003	2.7641
Rpp30	-1.0039	5.0277	-6.6619	0	0.0003	2.6942
9330104G04Rik	2.3086	0.5136	6.6598	0	0.0003	2.9544
Cep72	-1.347	2.4543	-6.6591	0	0.0003	3.0009
Slc36a1	1.294	5.1233	6.658	0	0.0003	2.6513
1600014C10Rik	1.1605	3.4044	6.6575	0	0.0003	2.863
Zfp128	1.4213	1.6646	6.6564	0	0.0003	3.0146
Pbdc1	-0.6546	6.2318	-6.656	0	0.0003	2.5694
Ilvbl	1.1177	3.7076	6.6527	0	0.0003	2.8125
Tnfaip3	2.0803	4.8464	6.65	0	0.0003	2.7166
3300005D01Rik	-0.9255	4.9588	-6.6466	0	0.0003	2.6745
Ypel5	1.152	5.8032	6.6462	0	0.0003	2.5697
Trim27	-0.9308	6.6828	-6.646	0	0.0003	2.5434
Eif4g1	-0.6702	9.9427	-6.6446	0	0.0003	2.497
Chchd1	-0.7904	5.912	-6.644	0	0.0003	2.5673
Srsf1	-0.9525	8.5019	-6.6401	0	0.0003	2.5021
Ywhah	-0.8029	7.1452	-6.6392	0	0.0003	2.5179
Fam83g	-1.1983	6.0608	-6.6331	0	0.0003	2.5635
Vps33a	0.7978	6.2057	6.6319	0	0.0003	2.5299
Zfp212	0.6467	4.5226	6.6318	0	0.0003	2.6707
Gm9825	-0.9115	4.7252	-6.6307	0	0.0003	2.6815
Srm	-1.4719	6.8388	-6.6286	0	0.0003	2.521
Gm34590	1.9792	0.1046	6.6284	0	0.0003	2.9207
Papola	-0.7568	8.1737	-6.628	0	0.0003	2.4864
Eif4a3	-0.5667	7.6357	-6.6263	0	0.0003	2.4896
Snrnp70	-0.7079	7.9297	-6.6251	0	0.0003	2.4845
Exosc3	-1.2031	4.9525	-6.6251	0	0.0003	2.6588
Plgrkt	1.3053	4.7456	6.623	0	0.0004	2.6398
Trmt11	-0.8697	4.0511	-6.622	0	0.0004	2.7551
Dnajc18	0.8709	5.2493	6.6207	0	0.0004	2.5747
Slbp	-0.9545	6.3831	-6.6164	0	0.0004	2.5114
Pisd-ps1	1.7801	1.1268	6.6148	0	0.0004	2.9596
D630003M21Ril	4.259	-0.6553	6.6098	0	0.0004	2.1809
Emg1	-0.7215	7.0838	-6.6057	0	0.0004	2.4681
Nsd2	-1.0299	7.8571	-6.6038	0	0.0004	2.4549
Srsf9	-0.8908	7.0377	-6.6013	0	0.0004	2.4636
Piga	-1.1606	4.7939	-6.5998	0	0.0004	2.6414
Otud4	-1.1348	7.3398	-6.5988	0	0.0004	2.4573
Clpb	-0.6919	5.4529	-6.597	0	0.0004	2.5356
Rpf2	-1.3343	5.7488	-6.5969	0	0.0004	2.5403
Nup153	-0.8162	7.3143	-6.5968	0	0.0004	2.4529
Plxnb1	3.7256	3.6094	6.5961	0	0.0004	2.8805
Agt	3.9887	2.6383	6.5954	0	0.0004	2.937
Nop16	-1.2731	6.2017	-6.5938	0	0.0004	2.498
Vasn	0.7849	6.9064	6.5906	0	0.0004	2.4461

Tmco4	1.3417	3.1748	6.5881	0	0.0004	2.8064
Pcdhga6	1.9539	0.4236	6.5869	0	0.0004	2.8653
Cct2	-0.5447	9.0299	-6.5837	0	0.0004	2.4095
Dctn1	0.5629	7.593	6.5826	0	0.0004	2.4214
Sf3a2	-0.6948	5.987	-6.5824	0	0.0004	2.474
Papolg	0.8181	4.6069	6.5813	0	0.0004	2.5896
Pcmt1	-0.6144	5.7932	-6.5797	0	0.0004	2.4813
Procr	-1.265	4.2395	-6.5709	0	0.0004	2.6657
Malat1	1.2812	8.8452	6.5672	0	0.0004	2.3859
Cdk8	-0.7771	5.7297	-6.5666	0	0.0004	2.4739
Usp20	1.0026	5.1424	6.5642	0	0.0004	2.5037
Npm1	-0.7796	10.9549	-6.5573	0	0.0004	2.3612
Zfp367	-1.4369	4.964	-6.5562	0	0.0004	2.5838
Hbegf	-2.051	4.7973	-6.5559	0	0.0004	2.654
Ppif	-1.1139	5.2089	-6.5556	0	0.0004	2.52
Arhgef18	1.0891	5.2721	6.5553	0	0.0004	2.4812
Lrrc59	-1.1499	8.7441	-6.5523	0	0.0004	2.3661
Rapgef3	-3.4298	4.9015	-6.5483	0	0.0004	2.7695
Plekha3	-0.7087	5.7811	-6.5472	0	0.0004	2.4351
Kri1	-0.7527	5.3376	-6.5466	0	0.0004	2.4732
Eef1d	-0.7548	7.9598	-6.5456	0	0.0004	2.3634
Chmp1b	0.9806	5.4177	6.5435	0	0.0004	2.4461
Rab11fip2	0.9615	3.3684	6.5391	0	0.0004	2.6856
Slk	-0.9927	7.6085	-6.5383	0	0.0004	2.359
Dcxr	2.1364	2.7841	6.5346	0	0.0004	2.7923
Cdkn2b	1.0859	5.8889	6.5337	0	0.0004	2.392
Nfkb2	0.7069	7.3943	6.5326	0	0.0004	2.3479
Foxn3	1.2298	5.713	6.5277	0	0.0004	2.4041
Tgfb2	3.1318	3.007	6.527	0	0.0004	2.8083
Fam122b	-0.9478	3.6128	-6.5252	0	0.0004	2.6733
Fbxo10	1.3954	3.9762	6.5221	0	0.0004	2.6018
Alad	-0.8553	4.8979	-6.5212	0	0.0004	2.4887
Tle3	0.5517	6.3534	6.511	0	0.0004	2.3394
Alg13	-1.2235	5.2479	-6.5067	0	0.0004	2.4538
Cd99l2	0.8515	6.1781	6.5061	0	0.0004	2.3383
Nrm	-1.6898	3.5298	-6.5056	0	0.0004	2.7084
Hist1h2al	3.1058	0.4132	6.5045	0	0.0004	2.657
Iars	-0.9721	7.9486	-6.5033	0	0.0004	2.2998
Dtnbp1	0.963	6.1674	6.4984	0	0.0004	2.3286
Tmem175	1.0367	4.0845	6.4921	0	0.0004	2.5255
Zmiz1	0.9732	7.801	6.4921	0	0.0004	2.2815
St3gal5	1.1071	5.706	6.4914	0	0.0004	2.3465
H2-T10	1.5842	3.4411	6.4908	0	0.0004	2.6321
Swap70	0.8681	5.5846	6.4886	0	0.0004	2.3451
Nap1l4	-0.6105	7.3093	-6.487	0	0.0004	2.2831
Fbxo6	1.1762	4.5121	6.4861	0	0.0004	2.4572
Ddx20	-0.9664	6.3105	-6.4799	0	0.0004	2.3075
Pik3r4	0.8274	5.4003	6.4789	0	0.0004	2.3459
Il1rap	-1.2417	6.1358	-6.4778	0	0.0004	2.3262
Nipal3	3.0166	2.0442	6.4776	0	0.0004	2.7727
Snrpg	-0.8398	6.4176	-6.4774	0	0.0004	2.2922
Fam50a	0.6653	5.953	6.4753	0	0.0004	2.2994

Copg2	0.6793	5.7396	6.473	0	0.0004	2.3097
Ptpn14	0.6438	6.2182	6.4722	0	0.0004	2.2854
Igsf8	-1.0366	7.0377	-6.4722	0	0.0004	2.2685
Phf21a	0.8037	5.7816	6.4716	0	0.0004	2.3067
Parva	0.8877	7.1711	6.4702	0	0.0004	2.2566
Slc4a8	-1.9659	2.9013	-6.469	0	0.0004	2.7217
Mrps25	-0.8188	5.5059	-6.4672	0	0.0004	2.3371
Pam	-0.6079	7.6453	-6.4656	0	0.0004	2.2448
Polr3g	-1.1803	4.8313	-6.4602	0	0.0004	2.4276
Nubp1	-0.8208	5.5672	-6.4601	0	0.0004	2.3261
Dnajc21	-1.1507	5.8436	-6.4595	0	0.0004	2.311
Zfp157	0.9412	3.736	6.4578	0	0.0004	2.5256
Idh3a	-0.9543	7.0101	-6.4541	0	0.0004	2.2413
Pfdn4	-1.0301	5.4149	-6.4536	0	0.0004	2.3341
Ctsd	1.4007	8.7459	6.4522	0	0.0004	2.21
Snrpd3	-0.7802	6.6298	-6.4505	0	0.0004	2.2433
Strap	-0.6186	8.3327	-6.4497	0	0.0004	2.2121
Stard7	-0.8036	6.4771	-6.4492	0	0.0004	2.2497
Mib2	0.981	4.2603	6.4482	0	0.0004	2.4265
Siah1b	-1.3336	4.2717	-6.4479	0	0.0004	2.5064
Rasa4	-1.5882	3.8026	-6.4474	0	0.0004	2.5603
Cpt1a	3.0533	4.4258	6.4458	0	0.0004	2.5011
Gbp2	3.4911	5.4695	6.4454	0	0.0004	2.3737
Slc7a5	-1.172	7.5004	-6.443	0	0.0004	2.2149
BC005537	-0.573	7.9648	-6.4415	0	0.0004	2.2039
Rhou	2.7237	6.5268	6.4405	0	0.0004	2.2441
Amdhd2	1.2306	4.5721	6.4404	0	0.0004	2.3758
Tom1	1.6156	3.4764	6.4376	0	0.0004	2.5547
Ctsb	0.825	9.6206	6.4356	0	0.0004	2.1787
Mgme1	-0.8468	4.4431	-6.429	0	0.0004	2.413
Nrbp2	3.6166	1.7636	6.4238	0	0.0004	2.6867
2810025M15Rik	-0.9285	4.1163	-6.4234	0	0.0004	2.4456
Mrps5	-0.7451	5.6306	-6.423	0	0.0004	2.2552
Rangrf	-1.332	3.9823	-6.4224	0	0.0004	2.4982
Sft2d2	0.9124	6.3729	6.4208	0	0.0004	2.2006
C1qtnf6	2.2792	5.8804	6.4206	0	0.0004	2.2503
Ndrg3	0.8276	6.0034	6.4159	0	0.0004	2.2082
Slc39a14	-1.5759	7.4131	-6.4135	0	0.0004	2.1777
Vdac1	-0.4764	8.5086	-6.4117	0	0.0004	2.1507
Clcc1	-0.7999	5.6095	-6.4108	0	0.0004	2.2442
Ano1	-1.2722	6.5637	-6.4091	0	0.0004	2.1899
Atp6v0e	0.7926	5.985	6.4063	0	0.0004	2.1946
Extl1	2.7807	1.4434	6.4022	0	0.0004	2.6608
Lhfpl2	-0.9133	5.8887	-6.4014	0	0.0004	2.2124
Zfp839	0.9225	3.4612	6.3983	0	0.0004	2.466
Kctd21	1.4527	2.4504	6.3977	0	0.0004	2.5987
Bcas3	1.5354	5.0341	6.3957	0	0.0004	2.2761
Cyb561d1	1.0204	2.6382	6.3949	0	0.0004	2.5636
Ubxn6	0.6042	5.9486	6.3945	0	0.0004	2.1763
Eno1	-0.8552	9.6893	-6.3944	0	0.0004	2.116
Cfh	5.1218	1.8006	6.3925	0	0.0004	2.4975
Snrpf	-1.0794	6.0962	-6.3923	0	0.0004	2.186

2310022B05Rik	1.6224	7.2406	6.3915	0	0.0004	2.1345
Srr	1.1145	4.5174	6.389	0	0.0004	2.315
Itpripl1	-1.3879	4.4758	-6.389	0	0.0004	2.3922
Tnpo3	-0.614	7.653	-6.3886	0	0.0004	2.1268
Ehbp1l1	-0.7391	6.3947	-6.3874	0	0.0004	2.1528
Cct3	-0.8332	9.3473	-6.3872	0	0.0004	2.1071
Ddr1	2.0233	6.224	6.3863	0	0.0004	2.1661
Tpm3	-0.7574	9.0691	-6.3854	0	0.0004	2.106
Smg9	-0.8464	4.5189	-6.3829	0	0.0004	2.3271
Htr1d	3.4386	-3.7282	6.3822	0	0.0004	1.5661
Vamp1	1.342	2.4267	6.3809	0	0.0005	2.5706
Mrps10	-0.7234	5.3044	-6.3803	0	0.0005	2.219
Gm32591	5.0198	-2.3264	6.3784	0	0.0005	1.3765
Nars	-1.0333	8.7595	-6.3758	0	0.0005	2.0941
Tubb5	-0.9396	10.3009	-6.3731	0	0.0005	2.0808
Zfand5	0.9346	7.3945	6.3716	0	0.0005	2.1021
Donson	-1.1765	3.8672	-6.3696	0	0.0005	2.425
Ybx1	-0.5472	9.6606	-6.3685	0	0.0005	2.0759
Parp10	1.5599	4.8077	6.3682	0	0.0005	2.2641
Samd9l	3.1036	1.5756	6.3673	0	0.0005	2.6087
AW554918	-1.0685	4.0108	-6.3669	0	0.0005	2.3866
C1qbp	-0.8272	7.9169	-6.3669	0	0.0005	2.0899
Nufip1	-0.7538	5.3612	-6.3653	0	0.0005	2.1957
Cln5	0.9884	4.851	6.3636	0	0.0005	2.2265
Pkm	-0.611	11.8843	-6.3602	0	0.0005	2.0559
Dcaf1	-0.7481	6.4977	-6.3597	0	0.0005	2.1089
Gspt1	-0.8029	8.2344	-6.3592	0	0.0005	2.0746
Zfp362	1.485	2.8069	6.3577	0	0.0005	2.4976
Ccdc14	-0.9138	3.4516	-6.355	0	0.0005	2.4282
Wdr76	-1.3937	4.169	-6.3541	0	0.0005	2.3804
Pik3ip1	2.7465	4.2197	6.3513	0	0.0005	2.3637
Mcc	2.3717	0.6492	6.3508	0	0.0005	2.5759
Pafah1b2	-0.6025	7.6799	-6.349	0	0.0005	2.0653
Thop1	-0.9126	6.235	-6.3488	0	0.0005	2.1072
Brca2	-1.4113	4.9549	-6.3465	0	0.0005	2.2648
Mylip	2.0807	4.4015	6.3445	0	0.0005	2.2986
Haus5	-1.6376	1.7525	-6.3443	0	0.0005	2.5823
Bnc2	2.2973	0.3015	6.3434	0	0.0005	2.5085
Ube4a	0.8107	6.4607	6.3423	0	0.0005	2.0776
Gaa	1.1455	7.776	6.3409	0	0.0005	2.0478
Fubp1	-0.817	7.1774	-6.3406	0	0.0005	2.0623
Rsl1	1.2618	1.6792	6.3399	0	0.0005	2.5624
Zmym1	-0.7484	4.6319	-6.3388	0	0.0005	2.2458
Pla2g12a	-0.9555	6.0657	-6.3371	0	0.0005	2.0985
Gm4735	-0.8776	6.1825	-6.3342	0	0.0005	2.0851
Nt5c3	-0.7439	5.1415	-6.334	0	0.0005	2.1741
Smyd5	-1.021	6.0638	-6.3322	0	0.0005	2.0973
Dyrk1b	1.625	4.2183	6.3322	0	0.0005	2.2907
Kif2a	-0.6642	6.6722	-6.3297	0	0.0005	2.0561
Trmt6	-0.818	5.8286	-6.3291	0	0.0005	2.1021
Snap29	0.7453	4.9676	6.3291	0	0.0005	2.1564
Jmjd6	-0.8397	6.6936	-6.3267	0	0.0005	2.0508

Smcr8	0.701	6.3735	6.3253	0	0.0005	2.0522
Itga6	-1.3684	7.3611	-6.3235	0	0.0005	2.0342
Rit1	0.8783	4.6593	6.3229	0	0.0005	2.1771
Pet100	1.0241	3.5008	6.3172	0	0.0005	2.3333
Cnnm2	2.1882	2.8691	6.3162	0	0.0005	2.4647
Gtpbp4	-0.7667	7.4412	-6.3123	0	0.0005	2.0126
Unc5b	-1.9228	6.4705	-6.3051	0	0.0005	2.0571
Csad	1.4765	4.8131	6.302	0	0.0005	2.1525
5430425K12Rik	-3.7541	-0.7248	-6.3	0	0.0005	1.8107
Hnrnpf	-0.5645	7.5437	-6.2993	0	0.0005	1.9899
Ulk1	1.2773	5.9173	6.2991	0	0.0005	2.04
Fosl2	1.2304	6.4174	6.2991	0	0.0005	2.0125
Zfp358	1.3227	4.8854	6.2984	0	0.0005	2.1311
2700099C18Rik	-1.509	2.3959	-6.2971	0	0.0005	2.4883
Mybbp1a	-1.0388	9.0424	-6.2967	0	0.0005	1.9697
Gprc5c	4.6055	-2.3963	6.2961	0	0.0005	1.3505
Cpeb4	1.4278	5.2385	6.2937	0	0.0005	2.1015
Tug1	0.6302	7.2653	6.2921	0	0.0005	1.9802
Uchl3	-1.0429	5.718	-6.2912	0	0.0005	2.0578
Asap1	0.647	7.4745	6.2892	0	0.0005	1.9725
Tomm5	-0.9503	5.5404	-6.2869	0	0.0005	2.0674
Trp53inp1	3.2907	4.7126	6.2862	0	0.0005	2.2711
Atad3a	-0.8922	6.1613	-6.2828	0	0.0005	2.0113
Zfp46	0.9868	3.8721	6.2808	0	0.0005	2.2279
Mad1l1	-0.7849	5.3835	-6.2807	0	0.0005	2.0637
Rab30	1.0629	2.9193	6.2786	0	0.0005	2.3699
Nop2	-0.9857	7.412	-6.2774	0	0.0005	1.9602
Elavl1	-0.6933	7.1133	-6.277	0	0.0005	1.9633
Acp1	-0.6806	5.6362	-6.2738	0	0.0005	2.0221
Gm15867	2.1856	-0.4896	6.2728	0	0.0005	2.2854
Kidins220	1.1237	6.245	6.2706	0	0.0005	1.9758
Mcee	0.8941	3.3734	6.2696	0	0.0005	2.2868
Nhp2	-1.2427	7.1584	-6.2693	0	0.0005	1.9546
Xpo4	-1.3028	6.4379	-6.2671	0	0.0005	1.9853
Tor4a	-1.0571	5.6772	-6.2656	0	0.0005	2.0158
K230015D01Rik	1.7629	0.5579	6.263	0	0.0005	2.4549
Lims1	1.0318	6.2844	6.2629	0	0.0005	1.9636
Bysl	-0.8149	5.6413	-6.2599	0	0.0005	2.0096
Phex	-2.186	4.8634	-6.2582	0	0.0005	2.199
Pvr	-0.9265	5.9019	-6.2577	0	0.0005	1.9815
Rgmb	1.1737	4.9692	6.2576	0	0.0005	2.0581
Eed	-0.933	5.7236	-6.255	0	0.0005	1.9998
Ankle1	-2.9618	2.3288	-6.2525	0	0.0005	2.4477
Xpo5	-0.7528	7.1591	-6.2505	0	0.0005	1.9214
Cib1	0.657	4.8947	6.2499	0	0.0005	2.0433
Steap1	-1.3102	5.288	-6.249	0	0.0005	2.0548
Pld2	0.8762	4.8303	6.249	0	0.0005	2.053
Zfand4	-1.3349	4.1041	-6.2478	0	0.0005	2.2148
Rbbp8	-0.9939	5.7424	-6.2455	0	0.0005	1.9842
Senp1	-0.933	6.4502	-6.2452	0	0.0005	1.9373
Marc2	0.6993	5.5956	6.2443	0	0.0005	1.9664
Polr2d	-0.7615	5.4347	-6.2441	0	0.0005	1.9964

Rrp8	-0.9302	5.7207	-6.2408	0	0.0005	1.9772
Rcc1l	-1.0179	4.5144	-6.2407	0	0.0005	2.1188
Rap2c	-1.0035	6.4879	-6.2389	0	0.0005	1.9305
Sarm1	3.144	-0.4716	6.2388	0	0.0005	2.1319
Adam15	0.5496	7.2364	6.2357	0	0.0005	1.8925
Igfbp7	2.5758	5.2706	6.2344	0	0.0005	2.0479
Sparc	2.0195	9.1075	6.234	0	0.0005	1.8693
Wdr3	-0.7243	7.2783	-6.2329	0	0.0005	1.8917
Eapp	0.7226	4.7861	6.2326	0	0.0005	2.0249
E2f1	-0.856	5.4796	-6.2292	0	0.0005	1.9776
Col27a1	2.2558	-0.1521	6.229	0	0.0005	2.3158
Sesn3	2.099	6.592	6.2276	0	0.0005	1.9056
Coro2a	-1.5183	5.6061	-6.2267	0	0.0005	1.984
Mnt	0.6841	5.8866	6.2259	0	0.0005	1.921
Zfp318	0.9426	5.0047	6.2228	0	0.0005	1.9959
Stk36	1.3373	2.5005	6.2222	0	0.0005	2.3213
Retreg2	0.7659	6.5976	6.2211	0	0.0005	1.8821
Pdcd4	0.8372	5.5511	6.2209	0	0.0005	1.9383
Msn	-0.736	9.4761	-6.2204	0	0.0005	1.8467
Parp12	1.2741	4.7793	6.2203	0	0.0005	2.0347
Sema3f	1.6137	4.6209	6.2185	0	0.0005	2.0717
Haghl	0.8558	4.0179	6.2171	0	0.0005	2.0934
Tial1	-0.9441	6.3161	-6.2151	0	0.0005	1.8971
Mettl9	-0.6277	6.4411	-6.2116	0	0.0005	1.8783
Tmem260	1.1575	3.4288	6.2114	0	0.0005	2.1982
Tor3a	1.4567	4.7868	6.2099	0	0.0005	2.002
Mmachc	-0.835	3.9369	-6.208	0	0.0005	2.1392
Pld1	2.3259	4.7977	6.2072	0	0.0005	2.0565
Add1	0.7862	7.5725	6.2067	0	0.0005	1.8411
Gtf2f2	-0.7667	5.5005	-6.2059	0	0.0005	1.9332
Slc38a7	1.5667	4.3706	6.2038	0	0.0005	2.0703
Stip1	-0.83	8.7035	-6.1993	0	0.0005	1.8193
Sac3d1	-0.8059	4.1923	-6.199	0	0.0005	2.0859
Sdhaf4	0.9649	3.8881	6.1957	0	0.0005	2.0944
Tnfrsf22	-1.2884	4.559	-6.1932	0	0.0005	2.061
Pgs1	-0.8825	6.6169	-6.1913	0	0.0005	1.8446
Utp18	-0.7978	6.5477	-6.1896	0	0.0005	1.8431
Zfp174	1.2277	1.3464	6.1875	0	0.0005	2.3504
Ino80e	-0.8813	5.9735	-6.1868	0	0.0005	1.8715
Dhx15	-0.8093	8.3198	-6.1866	0	0.0005	1.8043
Kdm4b	0.8135	5.9835	6.1862	0	0.0005	1.8526
Chd1	-0.6321	7.3334	-6.1852	0	0.0005	1.8159
Rbms1	-0.9735	7.7246	-6.1849	0	0.0005	1.8101
Naa10	-0.8349	5.7997	-6.1842	0	0.0005	1.8766
Pbxip1	2.1152	5.7462	6.1831	0	0.0005	1.887
Spry4	-6.1661	2.251	-6.1805	0	0.0006	1.8723
Wdr75	-0.9572	6.7176	-6.18	0	0.0006	1.8249
Itgb8	3.8362	2.5447	6.1782	0	0.0006	2.3427
Mrpl18	-0.6528	6.2142	-6.1781	0	0.0006	1.835
Sf3a3	-0.6626	6.7777	-6.1768	0	0.0006	1.813
Sdad1	-0.7847	6.3757	-6.1738	0	0.0006	1.8264
D5Ertd579e	0.8355	5.7192	6.1726	0	0.0006	1.8514

Fam133b	-1.109	5.5352	-6.1687	0	0.0006	1.8879
Stc1	-1.9084	4.0235	-6.1673	0	0.0006	2.147
Pnn	-0.9848	6.67	-6.1672	0	0.0006	1.8073
Mief2	1.116	3.0026	6.1671	0	0.0006	2.1784
Nagpa	0.719	4.6499	6.1669	0	0.0006	1.952
Atp6v1e1	0.7628	6.7096	6.1664	0	0.0006	1.7927
Gm26947	-2.7098	-0.7426	-6.1637	0	0.0006	2.0162
Ddx56	-0.8146	6.6661	-6.1633	0	0.0006	1.7969
Ccdc18	-1.5223	3.7325	-6.1632	0	0.0006	2.1467
Smpd4	-0.7364	5.5282	-6.1625	0	0.0006	1.8648
Maf	1.9456	5.3422	6.1613	0	0.0006	1.8921
Atl1	1.8055	1.3146	6.1569	0	0.0006	2.3143
Psmb3	-0.5885	7.1641	-6.1569	0	0.0006	1.7719
Gnpnat1	-1.1225	4.9441	-6.1548	0	0.0006	1.9392
Lclat1	-0.9941	5.1928	-6.1543	0	0.0006	1.9036
Psmg2	-0.7312	5.7557	-6.1527	0.0001	0.0006	1.8277
Ipo11	-0.8366	6.4553	-6.1499	0.0001	0.0006	1.7861
Fbxl12os	1.6367	0.7713	6.1489	0.0001	0.0006	2.3029
Als2	0.8076	5.5144	6.1402	0.0001	0.0006	1.8156
Zfp36l2	-0.7747	7.9411	-6.1398	0.0001	0.0006	1.736
G3bp1	-0.7478	8.7494	-6.1361	0.0001	0.0006	1.7198
Wdr7	1.4864	5.15	6.1355	0.0001	0.0006	1.8533
Ccne1	-1.6201	4.2519	-6.1354	0.0001	0.0006	2.0591
Suz12	-0.8194	7.3177	-6.135	0.0001	0.0006	1.738
Chfr	1.3599	5.6986	6.1338	0.0001	0.0006	1.7962
Gm42793	-2.313	1.1108	-6.1336	0.0001	0.0006	2.2542
Glrx5	-0.7628	6.2816	-6.1294	0.0001	0.0006	1.7581
Clta	0.5542	7.3558	6.1272	0.0001	0.0006	1.7194
Ccdc102a	0.9392	3.7207	6.1259	0.0001	0.0006	2.0159
Hsdl2	1.1179	3.7856	6.1253	0.0001	0.0006	2.0096
Polh	-0.8631	4.6106	-6.1245	0.0001	0.0006	1.9195
Marcksl1	1.2544	7.6383	6.1197	0.0001	0.0006	1.7035
U2surp	-0.6698	7.2716	-6.1194	0.0001	0.0006	1.7124
Rragc	0.6728	6.9687	6.1192	0.0001	0.0006	1.7135
Phb2	-0.6664	8.164	-6.1188	0.0001	0.0006	1.6982
Srsf3	-0.8491	8.0057	-6.1159	0.0001	0.0006	1.6973
Eef2k	1.0226	4.8295	6.1155	0.0001	0.0006	1.8445
Palm	0.8073	5.8073	6.1081	0.0001	0.0006	1.7363
Stat2	1.7718	3.4489	6.1025	0.0001	0.0006	2.0409
Hnrnpk	-0.539	9.7095	-6.1017	0.0001	0.0006	1.6578
Cntrob	-0.8254	4.0475	-6.1004	0.0001	0.0006	1.9522
Pink1	1.7396	4.5185	6.0996	0.0001	0.0006	1.895
Sh3bgrl2	-0.7472	5.3545	-6.0983	0.0001	0.0006	1.786
Pcgf5	-0.7583	5.2936	-6.0956	0.0001	0.0006	1.7858
Baz2b	1.0422	5.2846	6.095	0.0001	0.0006	1.7679
Jkamp	0.8378	4.8894	6.093	0.0001	0.0006	1.7964
Oxnad1	-1.1081	4.646	-6.0929	0.0001	0.0006	1.8735
Naa15	-0.9548	8.2427	-6.0917	0.0001	0.0006	1.656
9330102E08Rik	1.6121	-0.0917	6.0902	0.0001	0.0006	2.1854
Ptpn9	-1.0901	6.0537	-6.0881	0.0001	0.0006	1.7171
Abca1	3.0645	3.7681	6.0868	0.0001	0.0006	2.0953
Itpa	-0.6638	5.7684	-6.0851	0.0001	0.0006	1.7183

Akr1c14	4.5324	1.2554	6.0847	0.0001	0.0006	2.1447
Zcchc8	-0.6695	5.78	-6.0842	0.0001	0.0006	1.7169
Eif3j1	-0.8188	4.943	-6.0841	0.0001	0.0006	1.8144
Gm9260	-3.1843	3.4169	-6.0791	0.0001	0.0006	2.1736
Grwd1	-1.252	5.756	-6.0768	0.0001	0.0006	1.7308
Gm13092	-1.0568	3.2091	-6.0758	0.0001	0.0006	2.0424
Dcaf8	0.7122	7.0814	6.0725	0.0001	0.0006	1.6376
Pnpo	0.7125	4.1814	6.0721	0.0001	0.0006	1.8579
Ube2h	0.8539	6.5204	6.0717	0.0001	0.0006	1.6494
Zbtb16	1.9338	4.3748	6.0664	0.0001	0.0006	1.8825
Ogdhl	1.5038	3.5258	6.0659	0.0001	0.0006	1.9697
Siae	1.2722	4.8648	6.0645	0.0001	0.0006	1.7697
Zbtb24	0.9983	3.6467	6.0643	0.0001	0.0006	1.9271
Blmh	-0.6078	6.4312	-6.063	0.0001	0.0006	1.6428
Slc39a3	1.1838	3.7086	6.0602	0.0001	0.0006	1.9171
Get4	-0.6396	5.8882	-6.06	0.0001	0.0006	1.6683
Itga5	-1.0245	7.8439	-6.056	0.0001	0.0006	1.6043
Tesk1	0.8887	4.8058	6.0555	0.0001	0.0006	1.7562
Mutyh	-1.439	1.6515	-6.0546	0.0001	0.0006	2.1608
Amd1	-0.8971	6.0471	-6.054	0.0001	0.0006	1.6578
Ncam1	1.8885	5.6953	6.0538	0.0001	0.0006	1.6918
Lyst	1.1784	5.0122	6.0533	0.0001	0.0006	1.7343
Tbc1d5	0.5491	5.4158	6.0512	0.0001	0.0006	1.6813
Kat2a	-0.7943	7.1771	-6.0492	0.0001	0.0006	1.6037
Zfp651	1.4641	3.8293	6.0492	0.0001	0.0006	1.9076
Slc22a5	0.9611	2.7939	6.0472	0.0001	0.0006	2.0179
Vamp8	1.0624	5.7775	6.0453	0.0001	0.0006	1.6408
Wnt7b	-2.9839	6.0045	-6.0442	0.0001	0.0006	1.802
Hilpda	-0.9722	4.0844	-6.0434	0.0001	0.0006	1.8548
Ramp3	-2.9425	3.9284	-6.0394	0.0001	0.0006	2.0451
Hikeshi	-0.8026	5.4779	-6.0389	0.0001	0.0006	1.6728
Atxn7l3b	0.7104	6.6223	6.0387	0.0001	0.0006	1.5945
Rrp15	-0.9233	6.0486	-6.0325	0.0001	0.0006	1.6232
Taf5	-1.1574	4.2602	-6.0322	0.0001	0.0006	1.8449
Ssr3	-0.5805	9.5576	-6.0321	0.0001	0.0006	1.5481
Orc5	-0.755	4.9227	-6.0292	0.0001	0.0007	1.7226
Atp6v0a1	0.7624	5.7623	6.0263	0.0001	0.0007	1.6142
Ak2	-1.3045	6.9095	-6.0262	0.0001	0.0007	1.5797
Csk	-0.7027	6.5946	-6.022	0.0001	0.0007	1.5741
Szt2	0.9183	4.7019	6.0204	0.0001	0.0007	1.7233
Ift46	0.6243	6.2836	6.0179	0.0001	0.0007	1.5707
Naa50	-1.0446	7.8246	-6.0169	0.0001	0.0007	1.5436
Bmpr2	0.6085	7.1043	6.0164	0.0001	0.0007	1.5496
Akt1	-0.6424	8.3389	-6.0142	0.0001	0.0007	1.5297
Kifap3	0.6784	6.0619	6.0127	0.0001	0.0007	1.5737
Utp20	-0.7726	6.7053	-6.0124	0.0001	0.0007	1.5584
Dnaaf5	-0.8226	5.0771	-6.0108	0.0001	0.0007	1.6714
Ppt1	0.8521	6.4671	6.0096	0.0001	0.0007	1.5522
Polr2e	-0.6829	6.6799	-6.0093	0.0001	0.0007	1.5504
5330438D12Rik	2.3673	-0.4569	6.007	0.0001	0.0007	1.8889
Tbrg4	-0.966	6.6631	-6.0065	0.0001	0.0007	1.5523
Tmtc4	1.028	4.4033	6.0063	0.0001	0.0007	1.738

1110012L19Rik	0.6876	4.4942	6.0062	0.0001	0.0007	1.7082
Lrig1	-0.7856	8.2221	-6.0057	0.0001	0.0007	1.5185
Gcc1	0.9367	4.2613	6.0045	0.0001	0.0007	1.7451
Rbp2	-1.4374	2.6584	-6.0022	0.0001	0.0007	2.017
Dnajc8	-0.5155	6.6893	-6.0016	0.0001	0.0007	1.5357
Haus3	-1.1755	5.4202	-5.9955	0.0001	0.0007	1.6389
Lgals9	3.3896	3.6368	5.995	0.0001	0.0007	1.9304
Wipf1	-1.1554	5.0658	-5.9933	0.0001	0.0007	1.6715
Rbm8a	-0.5969	7.6093	-5.9918	0.0001	0.0007	1.503
Snhg15	-1.1973	3.8828	-5.9907	0.0001	0.0007	1.8341
Zfyve26	1.4654	5.4543	5.9899	0.0001	0.0007	1.5911
Arsg	1.7748	1.3078	5.9891	0.0001	0.0007	2.0698
Tex2	-1.1481	6.0152	-5.9881	0.0001	0.0007	1.5588
Chaf1b	-1.0429	5.2497	-5.988	0.0001	0.0007	1.6312
Pmp22	1.3957	6.0502	5.9877	0.0001	0.0007	1.5435
Slc35f5	0.7526	5.3906	5.9855	0.0001	0.0007	1.5803
Mon1b	0.8342	4.7389	5.9848	0.0001	0.0007	1.6441
Nbeal1	1.0331	5.2108	5.9846	0.0001	0.0007	1.61
Lamtor4	0.9294	4.4987	5.9828	0.0001	0.0007	1.678
Mbd1	0.9336	6.2502	5.9822	0.0001	0.0007	1.5191
Tinagl1	-1.577	7.5961	-5.9807	0.0001	0.0007	1.4895
Adamtsl4	1.9814	5.0485	5.9782	0.0001	0.0007	1.6355
Tmem59	0.7408	7.4927	5.9774	0.0001	0.0007	1.4794
Gmps	-0.7026	8.1113	-5.9725	0.0001	0.0007	1.4665
Igbp1	0.7042	6.5431	5.9705	0.0001	0.0007	1.4862
Tnrc18	1.0375	7.8438	5.9704	0.0001	0.0007	1.4634
Afp	-1.8411	2.3458	-5.9694	0.0001	0.0007	2.0149
Trps1	0.7051	7.4014	5.9689	0.0001	0.0007	1.4684
Crebl2	2.0258	2.9559	5.9683	0.0001	0.0007	1.93
Anxa1	0.9631	9.5024	5.968	0.0001	0.0007	1.4447
B3galt4	1.2553	1.848	5.9672	0.0001	0.0007	1.9967
Grina	1.2886	7.6326	5.9671	0.0001	0.0007	1.4603
Stap2	2.7991	2.547	5.9657	0.0001	0.0007	1.9901
Ercc4	1.0772	3.5818	5.9657	0.0001	0.0007	1.7884
Smchd1	-0.7724	7.2101	-5.9653	0.0001	0.0007	1.47
Dand5	1.2966	1.6137	5.9651	0.0001	0.0007	2.0146
Ppp2r3a	1.144	6.3454	5.9646	0.0001	0.0007	1.4893
Smndc1	-0.6826	6.0547	-5.9646	0.0001	0.0007	1.5092
Pip4p1	0.5545	5.4088	5.9621	0.0001	0.0007	1.5353
Ifit1bl1	4.2779	-3.1928	5.9607	0.0001	0.0007	1.1007
Apbb2	0.8807	6.3558	5.9604	0.0001	0.0007	1.4799
Nutf2	-0.8493	5.336	-5.9549	0.0001	0.0007	1.5531
Abhd17c	-0.9178	5.5658	-5.9548	0.0001	0.0007	1.5402
Tmem39b	-0.878	3.8015	-5.9548	0.0001	0.0007	1.7737
Zwint	-0.6067	7.231	-5.952	0.0001	0.0007	1.4461
Bsdc1	0.8811	5.0153	5.9506	0.0001	0.0007	1.5579
Atp6v0a2	0.6946	6.0861	5.9506	0.0001	0.0007	1.4779
1700120C14Rik	-1.6571	0.838	-5.9505	0.0001	0.0007	2.0163
1700037H04Rik	-0.8636	5.3808	-5.9501	0.0001	0.0007	1.5427
Cebpz	-0.6331	7.5095	-5.9499	0.0001	0.0007	1.4389
Vcpkmt	-1.2059	4.5334	-5.946	0.0001	0.0007	1.6737
Bnc1	-1.7706	5.0024	-5.9453	0.0001	0.0007	1.6478

Yrdc	-1.0799	5.5063	-5.9442	0.0001	0.0007	1.5317
Dcaf15	-1.0503	4.969	-5.9413	0.0001	0.0007	1.5943
Xpo7	-0.6265	7.2605	-5.9399	0.0001	0.0007	1.4265
Ifngr1	0.9404	5.8112	5.9388	0.0001	0.0007	1.4754
Gm8186	-0.6695	4.7863	-5.9382	0.0001	0.0007	1.5803
Tmem176b	1.5672	7.6443	5.9366	0.0001	0.0007	1.4122
Kctd10	0.8895	7.5972	5.9362	0.0001	0.0007	1.4114
Cep192	-0.9814	6.6341	-5.9362	0.0001	0.0007	1.4422
Rbmxl1	-0.6781	5.8204	-5.9337	0.0001	0.0007	1.4752
Peg13	1.3365	2.7733	5.931	0.0001	0.0007	1.8575
Svbp	1.026	3.2283	5.9281	0.0001	0.0007	1.7705
Top1mt	-0.782	4.0771	-5.9263	0.0001	0.0007	1.6686
Hmgb3	-0.8829	5.907	-5.9258	0.0001	0.0007	1.4596
Hs6st1	1.0046	4.6792	5.9254	0.0001	0.0007	1.5683
Gk	-1.2692	4.9704	-5.9252	0.0001	0.0007	1.5857
Rif1	-0.9848	6.5908	-5.9244	0.0001	0.0007	1.4253
Pelp1	-0.7279	6.3396	-5.9219	0.0001	0.0007	1.4246
Farp1	0.5883	6.2747	5.9209	0.0001	0.0007	1.4172
Large1	1.5817	6.2349	5.9206	0.0001	0.0007	1.4251
Stil	-1.2318	4.5784	-5.9199	0.0001	0.0007	1.6288
Herc3	1.1572	5.1674	5.9183	0.0001	0.0007	1.5033
Isg20l2	-0.7414	6.8288	-5.914	0.0001	0.0007	1.396
Usp36	-0.7463	6.1671	-5.9115	0.0001	0.0007	1.4201
Mocs1	1.3062	4.3828	5.9104	0.0001	0.0007	1.592
Pdia6	-0.8233	9.0488	-5.9093	0.0001	0.0007	1.3547
Ube2n	-0.6841	5.9844	-5.9088	0.0001	0.0007	1.4235
Kdm4a	0.7975	6.5011	5.9086	0.0001	0.0007	1.3888
Zpr1	-1.0494	6.8259	-5.9078	0.0001	0.0007	1.3867
Pdxp	-1.1772	3.8908	-5.9072	0.0001	0.0007	1.6948
Col4a5	2.0962	8.0306	5.9057	0.0001	0.0007	1.3585
Rpia	-0.7703	4.7092	-5.9057	0.0001	0.0007	1.5522
Asb13	0.6387	4.6979	5.905	0.0001	0.0007	1.5238
Hist3h2a	1.9553	1.253	5.9047	0.0001	0.0007	1.9468
Maged1	0.5542	8.8882	5.9046	0.0001	0.0007	1.3467
Eif5	-0.8089	8.7114	-5.9033	0.0001	0.0007	1.3487
Nol10	-0.8714	6.2853	-5.9032	0.0001	0.0007	1.4037
Prmt5	-0.925	6.747	-5.9018	0.0001	0.0007	1.3807
Pwwp3a	-0.6433	5.139	-5.9017	0.0001	0.0007	1.4899
Ppp1r2	-0.6183	6.0458	-5.9002	0.0001	0.0007	1.4035
Rab3il1	4.4344	1.3461	5.8967	0.0001	0.0007	1.9097
Stard3	0.9512	4.6198	5.8959	0.0001	0.0007	1.5231
Pmpca	-0.6045	7.2309	-5.895	0.0001	0.0007	1.3542
Nrip1	-1.2071	6.7384	-5.8924	0.0001	0.0008	1.3686
Aldh18a1	-1.0214	6.7341	-5.8877	0.0001	0.0008	1.356
Atpaf2	-0.6961	5.0608	-5.8867	0.0001	0.0008	1.4719
Mrpl50	-0.6766	5.3652	-5.8854	0.0001	0.0008	1.4386
Ezh1	0.7465	4.9585	5.882	0.0001	0.0008	1.4524
Fam171a2	1.5909	3.8187	5.8812	0.0001	0.0008	1.644
Abcf2	-0.6883	7.7057	-5.8805	0.0001	0.0008	1.3239
Hmgn1	-0.5395	8.6558	-5.88	0.0001	0.0008	1.3107
Tsc1	0.6629	5.6457	5.8771	0.0001	0.0008	1.3842
Rtkn2	-1.7468	2.6831	-5.8763	0.0001	0.0008	1.827

Foxn2	-0.8518	5.5101	-5.8752	0.0001	0.0008	1.4202
Tbc1d1	-0.7256	6.099	-5.8738	0.0001	0.0008	1.3593
Gm7278	-2.3667	0.3187	-5.8737	0.0001	0.0008	1.8293
Rpn2	-0.5678	8.6258	-5.8695	0.0001	0.0008	1.2937
Atp6v0c	0.6164	5.2671	5.869	0.0001	0.0008	1.3987
Enkd1	-1.1446	4.1284	-5.8676	0.0001	0.0008	1.5993
Hspa2	1.2959	3.9036	5.8672	0.0001	0.0008	1.5931
Comt	0.8865	6.2683	5.8668	0.0001	0.0008	1.3307
Dip2b	0.9781	6.1616	5.8659	0.0001	0.0008	1.3359
Tmem44	5.0122	1.339	5.8656	0.0001	0.0008	1.832
Rtn4r	5.6803	-1.3729	5.8654	0.0001	0.0008	1.2608
Slc25a20	1.1052	4.0998	5.8613	0.0001	0.0008	1.5415
Metap1	-0.5558	6.8141	-5.8607	0.0001	0.0008	1.308
Pfkfb2	1.541	2.3078	5.8596	0.0001	0.0008	1.806
Sdf2	0.7607	5.3455	5.8588	0.0001	0.0008	1.3764
Hectd3	1.0422	4.4262	5.8578	0.0001	0.0008	1.4869
Il15	2.6427	0.1556	5.8574	0.0001	0.0008	1.8244
Vsig10l	2.525	-0.0245	5.8562	0.0001	0.0008	1.8303
Ubtd1	-1.1265	5.913	-5.8553	0.0001	0.0008	1.352
Saraf	0.7544	6.7511	5.854	0.0001	0.0008	1.2937
Zscan29	1.0473	4.0289	5.8532	0.0001	0.0008	1.5379
Twistnb	-0.8096	5.632	-5.8508	0.0001	0.0008	1.364
Rab11b	0.732	5.7035	5.85	0.0001	0.0008	1.3342
Dagla	-1.3613	4.7286	-5.8451	0.0001	0.0008	1.4912
Psip1	-0.7498	7.4534	-5.8437	0.0001	0.0008	1.2692
Irf9	1.538	3.5473	5.8392	0.0001	0.0008	1.6155
Rai14	-1.3973	7.8596	-5.8377	0.0001	0.0008	1.2545
Nop9	-0.6351	5.9889	-5.8373	0.0001	0.0008	1.3067
Eftud2	-0.7033	8.0627	-5.8357	0.0001	0.0008	1.2464
Rpl7l1	-0.6572	7.4661	-5.8354	0.0001	0.0008	1.2544
Numbl	1.6843	4.2917	5.8352	0.0001	0.0008	1.4896
Irx1	0.8678	6.6663	5.8317	0.0001	0.0008	1.2621
Ppp3cb	-0.9094	6.5826	-5.8313	0.0001	0.0008	1.2725
10-sept	-1.1059	5.6721	-5.831	0.0001	0.0008	1.3338
Ppme1	-0.5012	6.255	-5.8307	0.0001	0.0008	1.2755
Slc25a24	-0.5069	7.7869	-5.83	0.0001	0.0008	1.2405
Flad1	0.7372	4.6115	5.8295	0.0001	0.0008	1.4131
Rita1	-0.815	3.6836	-5.8285	0.0001	0.0008	1.5703
Ulk2	1.0467	5.4982	5.8282	0.0001	0.0008	1.317
Pdap1	-0.7277	7.648	-5.8244	0.0001	0.0008	1.2335
Lars	-0.8322	8.2119	-5.8234	0.0001	0.0008	1.2243
Pex26	0.7875	3.8431	5.8231	0.0001	0.0008	1.5206
Csgalnact1	-2.6409	4.2725	-5.8225	0.0001	0.0008	1.6527
Map3k14	1.2012	5.0151	5.8223	0.0001	0.0008	1.3626
Cntrl	-1.0844	5.6676	-5.8218	0.0001	0.0008	1.3223
Laptm4a	0.8858	8.5773	5.8213	0.0001	0.0008	1.2148
Mpv17l2	0.736	4.3843	5.8206	0.0001	0.0008	1.4208
Ampd2	-0.7676	6.5487	-5.8204	0.0001	0.0008	1.2526
Socs5	-1.0627	6.0469	-5.8195	0.0001	0.0008	1.2917
Jpt2	-0.8442	6.9016	-5.8179	0.0001	0.0008	1.2401
Hmgn5	-1.1636	5.3563	-5.8179	0.0001	0.0008	1.3525
Copz2	1.3575	4.2425	5.8175	0.0001	0.0008	1.4696

Ngef	-3.3366	1.9213	-5.817	0.0001	0.0008	1.814
Lgals8	0.8727	5.8319	5.8169	0.0001	0.0008	1.272
Smim1	1.8663	3.8564	5.8152	0.0001	0.0008	1.5343
Acadvl	0.5077	6.2818	5.8144	0.0001	0.0008	1.2433
Fnip1	1.0135	6.1132	5.8122	0.0001	0.0008	1.2537
Nsmce4a	-0.6063	6.2521	-5.8117	0.0001	0.0008	1.2486
Zdhhc13	-0.6898	4.6332	-5.8109	0.0001	0.0008	1.4102
Rbbp7	-0.8113	8.7471	-5.8087	0.0001	0.0008	1.1951
Atg4a	1.0727	4.0179	5.8078	0.0001	0.0008	1.4772
Osbpl5	0.9883	4.3525	5.8077	0.0001	0.0008	1.4185
Zscan20	0.9385	2.6094	5.8061	0.0001	0.0008	1.6618
Psmd12	-0.7036	7.5953	-5.8058	0.0001	0.0008	1.204
Dcaf11	0.807	5.0723	5.805	0.0001	0.0008	1.3155
Pno1	-0.8514	6.3636	-5.8047	0.0001	0.0008	1.2369
Wwp2	0.5949	6.21	5.8046	0.0001	0.0008	1.2309
B2m	1.1122	8.5906	5.8038	0.0001	0.0008	1.1858
Gemin2	-0.8065	4.2436	-5.8034	0.0001	0.0008	1.4581
Eif1ax	-0.8382	7.7833	-5.8019	0.0001	0.0008	1.1959
Creb3	0.7454	4.7907	5.8006	0.0001	0.0008	1.3388
Pitpnb	-0.6541	7.2723	-5.798	0.0001	0.0008	1.1973
Abcd4	-0.9317	4.5234	-5.7978	0.0001	0.0008	1.3987
Cct4	-0.5291	8.603	-5.7952	0.0001	0.0008	1.1733
Rbm4b	1.0673	3.5395	5.7944	0.0001	0.0008	1.5319
Pus7	-1.2062	6.9528	-5.7927	0.0001	0.0008	1.2026
Mak16	-1.0281	6.7984	-5.7919	0.0001	0.0008	1.2022
Mrpl15	-0.6264	6.1827	-5.7911	0.0001	0.0008	1.2179
Inpp5e	1.0553	4.6444	5.7907	0.0001	0.0008	1.3567
Erlec1	0.7184	5.5153	5.7904	0.0001	0.0008	1.2536
Npc2	0.9049	8.4876	5.7902	0.0001	0.0008	1.1647
H2afx	-1.1208	6.4697	-5.7874	0.0001	0.0008	1.2114
Smg5	-0.8194	7.4056	-5.7867	0.0001	0.0008	1.1776
Rab28	-0.8404	5.4736	-5.7837	0.0001	0.0008	1.2635
Gm6560	-0.8056	3.2895	-5.783	0.0001	0.0008	1.545
Rpa1	-0.6726	7.6131	-5.78	0.0001	0.0008	1.1622
Srrt	-0.6719	7.5796	-5.7771	0.0001	0.0009	1.1581
Pls3	-0.5551	8.5997	-5.775	0.0001	0.0009	1.1407
Xrcc1	-0.633	5.5966	-5.7741	0.0001	0.0009	1.2328
Gm3226	-1.9065	1.9032	-5.7739	0.0001	0.0009	1.7472
Arhgef10	1.8851	4.0099	5.7739	0.0001	0.0009	1.4639
Cep83	-0.8938	5.11	-5.7737	0.0001	0.0009	1.2932
Mien1	0.9604	4.9241	5.7731	0.0001	0.0009	1.2771
Chek2	-0.8905	4.4384	-5.7725	0.0001	0.0009	1.3861
Rgs16	-2.2984	5.9679	-5.7714	0.0001	0.0009	1.2631
Tspan31	0.6169	6.2985	5.7694	0.0001	0.0009	1.1703
Vmn1r43	-1.7977	2.7581	-5.7667	0.0001	0.0009	1.6707
Ddx39b	-1.0375	7.7892	-5.7661	0.0001	0.0009	1.138
Sesn1	1.6362	5.1887	5.7649	0.0001	0.0009	1.2661
Tnfaip2	-2.6678	8.1327	-5.7647	0.0001	0.0009	1.143
Arhgef17	1.4351	7.2758	5.7643	0.0001	0.0009	1.1392
Bcdin3d	-1.0825	3.2539	-5.7643	0.0001	0.0009	1.5598
Zxdc	0.813	5.0773	5.7624	0.0001	0.0009	1.2601
Tcirg1	0.8275	5.9549	5.7621	0.0001	0.0009	1.1777

Drg2	-0.582	6.4429	-5.7617	0.0001	0.0009	1.1578
Agrn	0.8354	6.9806	5.7586	0.0001	0.0009	1.1349
Man2b2	2.4076	3.6642	5.7547	0.0001	0.0009	1.5094
Memo1	-0.6682	6.4615	-5.7538	0.0001	0.0009	1.147
Usp45	-0.6296	5.5787	-5.7535	0.0001	0.0009	1.2033
Pank4	-0.7859	4.1812	-5.7535	0.0001	0.0009	1.386
Smim14	1.0621	5.4393	5.7527	0.0001	0.0009	1.1995
Cd81	1.021	6.8037	5.75	0.0001	0.0009	1.1244
Ahdc1	1.0112	5.7606	5.7481	0.0001	0.0009	1.1721
Casp12	2.8965	5.2563	5.7464	0.0001	0.0009	1.3017
Resf1	0.737	7.3812	5.7445	0.0001	0.0009	1.1053
Hint1	-0.6905	8.0465	-5.7415	0.0001	0.0009	1.0927
Snx9	-0.5851	7.7814	-5.7393	0.0001	0.0009	1.0933
Gemin5	-0.8583	6.7984	-5.7388	0.0001	0.0009	1.1145
Nfix	0.9208	7.3106	5.738	0.0001	0.0009	1.0954
Tmem150a	1.5899	3.726	5.7378	0.0001	0.0009	1.4365
Bri3	0.8315	5.718	5.7358	0.0001	0.0009	1.1452
Ifi27	1.5606	4.7522	5.735	0.0001	0.0009	1.2648
Sms-ps	-1.5502	1.5141	-5.7344	0.0001	0.0009	1.6878
Hspe1	-0.7801	7.4398	-5.7338	0.0001	0.0009	1.0898
Tspyl1	0.5521	5.7596	5.7338	0.0001	0.0009	1.1403
Slc35e3	0.8059	4.1179	5.732	0.0001	0.0009	1.3397
Ttc39b	-1.6768	5.0374	-5.7254	0.0001	0.0009	1.2885
Gle1	-0.7421	6.018	-5.7252	0.0001	0.0009	1.1258
Ubqln4	-0.6476	7.2948	-5.7247	0.0001	0.0009	1.0779
Eno1b	-0.7974	6.9889	-5.7236	0.0001	0.0009	1.0814
Gadd45gip1	-0.7495	5.6082	-5.7225	0.0001	0.0009	1.1483
Tmed5	-0.9207	6.9329	-5.7218	0.0001	0.0009	1.0847
Atf4	-1.0079	7.9907	-5.7195	0.0001	0.0009	1.058
Crispld2	2.5829	4.571	5.717	0.0001	0.0009	1.3171
Rab5b	0.7187	5.967	5.7166	0.0001	0.0009	1.1009
Tmem104	0.8564	3.8833	5.7163	0.0001	0.0009	1.3383
Aaas	-0.8734	5.7755	-5.7145	0.0001	0.0009	1.1253
Cgn	3.1571	1.5392	5.711	0.0001	0.0009	1.665
Cbx5	-0.998	8.1733	-5.7104	0.0001	0.0009	1.0422
Siva1	-0.7955	5.0067	-5.7101	0.0001	0.0009	1.1971
Prmt7	-1.1751	5.9252	-5.7061	0.0001	0.0009	1.1143
Abcd1	1.4191	4.296	5.7054	0.0001	0.0009	1.2744
Nmd3	-0.7562	6.9842	-5.7051	0.0001	0.0009	1.0523
Vegfa	-1.0751	6.6379	-5.7049	0.0001	0.0009	1.0637
Ypel3	1.6327	4.3062	5.7034	0.0001	0.0009	1.2864
Rnf213	1.0524	7.0234	5.7007	0.0001	0.0009	1.0404
Anapc15	-0.9484	4.0988	-5.7003	0.0001	0.0009	1.3204
Rab32	2.5533	4.2649	5.6981	0.0001	0.0009	1.3533
Nlgn2	0.8815	5.1312	5.6973	0.0001	0.0009	1.1404
Atrnl1	0.9423	6.0752	5.6945	0.0001	0.0009	1.0621
Abca3	0.9753	6.3745	5.6944	0.0001	0.0009	1.0469
Stat5a	1.9487	2.9352	5.6932	0.0001	0.0009	1.4873
Orc3	-0.5845	5.5978	-5.6912	0.0001	0.0009	1.0965
Ankrd55	2.0413	1.4217	5.6878	0.0001	0.0009	1.6195
Dlat	-0.5654	6.6184	-5.6849	0.0001	0.0009	1.0251
2310057M21Rik	-0.8603	3.963	-5.684	0.0001	0.0009	1.3128

Pex11g	1.4888	1.8269	5.6832	0.0001	0.0009	1.5717
Gm9115	-2.7078	-0.127	-5.6799	0.0001	0.001	1.4784
Wnt4	4.0416	0.5694	5.6786	0.0001	0.001	1.5501
Gnptg	0.8288	3.6849	5.677	0.0001	0.001	1.2769
Smc1a	-0.7007	7.9969	-5.6757	0.0001	0.001	0.9858
Nob1	-0.7167	6.0981	-5.673	0.0001	0.001	1.032
Setd1b	1.3756	5.0905	5.6696	0.0001	0.001	1.118
Sfxn3	1.0176	6.3815	5.6693	0.0001	0.001	1.0052
Pkp2	1.166	4.2439	5.6675	0.0001	0.001	1.221
mt-Co1	0.7008	12.3276	5.6664	0.0001	0.001	0.9464
Sbk1	1.7134	3.685	5.6663	0.0001	0.001	1.332
Fndc7	2.1291	1.2945	5.6634	0.0001	0.001	1.5895
Iah1	0.9677	4.2589	5.6614	0.0001	0.001	1.195
Tbc1d9b	0.7168	6.5536	5.6612	0.0001	0.001	0.9843
1700020I14Rik	0.799	6.0123	5.6602	0.0001	0.001	1.0088
Arrdc3	-1.1188	6.4099	-5.6578	0.0001	0.001	1.0012
Slc25a48	2.3397	4.727	5.6576	0.0001	0.001	1.1949
Zw10	-0.7671	5.4179	-5.6573	0.0001	0.001	1.0684
Wrap53	-0.8165	3.9067	-5.6564	0.0001	0.001	1.2666
Flrt3	-1.5318	3.5875	-5.656	0.0001	0.001	1.3981
Diexf	-0.757	5.0271	-5.6555	0.0001	0.001	1.1102
Sephs2	-0.7676	5.6643	-5.655	0.0001	0.001	1.0381
Slc16a3	-2.1697	5.7381	-5.6544	0.0001	0.001	1.1045
Sel1l	1.0053	7.2176	5.6534	0.0001	0.001	0.9575
Gga2	0.8869	5.5228	5.6524	0.0001	0.001	1.027
Inca1	1.8188	0.339	5.651	0.0001	0.001	1.5639
Ergic3	0.7129	6.7948	5.6509	0.0001	0.001	0.9616
Cd9	-0.8415	8.6494	-5.6503	0.0001	0.001	0.9365
Rnh1	0.709	7.824	5.6484	0.0001	0.001	0.9398
Fbf1	-0.6865	6.4644	-5.648	0.0001	0.001	0.9729
Arid5b	0.698	6.2898	5.6468	0.0001	0.001	0.972
Lysmd4	1.4838	2.7081	5.6464	0.0001	0.001	1.4097
Plp2	-0.6664	7.1834	-5.646	0.0001	0.001	0.9496
Gnpda1	1.4884	4.5658	5.6449	0.0001	0.001	1.1434
H2-K1	0.9002	8.2266	5.6433	0.0001	0.001	0.9267
Grhl2	-2.9049	1.3978	-5.6378	0.0001	0.001	1.5527
Akr1b10	1.0695	3.1848	5.6371	0.0001	0.001	1.3188
Tex10	-0.6807	6.0551	-5.6358	0.0001	0.001	0.9753
Wdr47	0.8627	4.0108	5.6353	0.0001	0.001	1.1856
Usp10	-0.8168	6.8857	-5.6347	0.0001	0.001	0.9399
Lrwd1	-0.7597	5.4366	-5.6345	0.0001	0.001	1.0224
Tead3	1.8546	3.3847	5.6335	0.0001	0.001	1.3238
4933431E20Rik	2.0048	3.0658	5.6322	0.0001	0.001	1.3765
Itln1	2.6746	-0.3849	5.6313	0.0001	0.001	1.4962
Mtrex	-0.6315	7.544	-5.6284	0.0001	0.001	0.9147
Me2	-0.9994	6.8923	-5.6269	0.0001	0.001	0.9271
Dusp2	-3.2294	2.5583	-5.6255	0.0001	0.001	1.5363
Akip1	1.1031	3.8515	5.6247	0.0001	0.001	1.2023
Rubcn	0.65	5.561	5.6238	0.0001	0.001	0.976
Pbx1	0.7403	7.1305	5.623	0.0001	0.001	0.909
Mrc2	-0.925	8.2578	-5.6208	0.0001	0.001	0.8934
Myorg	1.989	3.0963	5.6198	0.0001	0.001	1.3448

Rnaseh2c	-0.892	4.4924	-5.6197	0.0001	0.001	1.1182
Pgpep1	1.9708	4.9341	5.6191	0.0001	0.001	1.0655
Plekhm2	0.7983	5.2151	5.6185	0.0001	0.001	1.0005
Sptssa	-0.7098	5.9465	-5.6176	0.0001	0.001	0.9541
Ccnb1-ps	-3.6944	-1.2199	-5.6169	0.0001	0.001	0.9183
Tln2	1.7632	4.7235	5.6161	0.0001	0.001	1.0837
Hmox1	1.0431	6.1022	5.6156	0.0001	0.001	0.9273
Tdp2	-0.7984	5.7443	-5.6138	0.0001	0.001	0.9656
Fkbp3	-0.9391	6.7977	-5.6109	0.0001	0.001	0.9039
Mrps27	-0.6726	5.7156	-5.6102	0.0001	0.001	0.957
Ncstn	0.523	6.9472	5.6064	0.0001	0.001	0.885
Prkra	0.7755	4.9579	5.6046	0.0001	0.001	1.0002
Plekhf1	3.213	3.6034	5.6012	0.0001	0.001	1.3261
Ttc23	0.7775	4.097	5.6008	0.0001	0.001	1.1116
Nifk	-0.7595	6.8338	-5.6004	0.0001	0.001	0.8849
Anxa11	-0.8844	5.9669	-5.6003	0.0001	0.001	0.9241
Irs2	1.6572	4.557	5.6003	0.0001	0.001	1.0849
Bud23	-0.6283	5.1909	-5.5969	0.0001	0.001	0.9817
Btd	0.9868	4.7134	5.5964	0.0001	0.001	1.0264
Pcna-ps2	-1.9558	0.0407	-5.5962	0.0001	0.001	1.4406
Rasgef1b	3.043	-0.6587	5.5961	0.0001	0.001	1.3391
Pwp2	-0.7277	5.5126	-5.5945	0.0001	0.0011	0.9566
Coq8b	1.1489	3.4694	5.5943	0.0001	0.0011	1.2236
Tpst1	-0.9358	5.5894	-5.5936	0.0001	0.0011	0.9509
Ano10	0.8984	5.653	5.5923	0.0001	0.0011	0.9179
Mfsd4b4	0.9756	3.0261	5.5922	0.0001	0.0011	1.2801
Kbtbd6	-2.211	0.1746	-5.5921	0.0001	0.0011	1.4374
Hdgf	-0.664	9.6699	-5.5912	0.0001	0.0011	0.831
Tamm41	-0.6864	4.8304	-5.5908	0.0001	0.0011	1.0193
Polr1e	-0.7171	4.7932	-5.5908	0.0001	0.0011	1.031
Ajuba	1.6967	5.2547	5.5907	0.0001	0.0011	0.9733
Ets1	0.8515	7.3583	5.5906	0.0001	0.0011	0.8518
Exoc3	0.4528	5.7579	5.5875	0.0001	0.0011	0.9003
Cfap298	-1.1979	4.5557	-5.5872	0.0001	0.0011	1.0826
Rbm10	-0.4951	6.5205	-5.5852	0.0001	0.0011	0.8656
Tent4a	-0.7012	6.0672	-5.5841	0.0001	0.0011	0.8889
S100a11	0.7864	7.5635	5.5835	0.0001	0.0011	0.8359
Adam11	1.7571	0.7917	5.5835	0.0001	0.0011	1.4716
Lfng	1.6747	3.449	5.583	0.0001	0.0011	1.2185
Ppan	-1.0737	6.3186	-5.5796	0.0001	0.0011	0.8778
Klhdc8b	1.931	0.632	5.5757	0.0001	0.0011	1.4635
Cep350	0.857	5.788	5.5738	0.0001	0.0011	0.8825
Acot13	0.8793	5.4253	5.5713	0.0001	0.0011	0.8994
Irak4	1.1687	4.1136	5.5701	0.0001	0.0011	1.0716
Inava	1.2433	2.6254	5.5686	0.0001	0.0011	1.2995
Sgpl1	0.781	6.5254	5.5672	0.0001	0.0011	0.8312
Tns3	1.1466	8.0041	5.5668	0.0001	0.0011	0.8041
Yaf2	-1.011	5.6569	-5.565	0.0001	0.0011	0.9011
Insl6	2.1611	1.4617	5.5644	0.0001	0.0011	1.4129
2210016L21Rik	0.6958	4.3984	5.5642	0.0001	0.0011	1.0107
Tm2d2	0.8979	5.6245	5.5641	0.0001	0.0011	0.8731
Ywhae	-0.6373	9.6574	-5.5641	0.0001	0.0011	0.786

Tubb2a	1.6937	4.457	5.5637	0.0001	0.0011	1.0238
Nup88	-0.5157	7.0687	-5.5628	0.0001	0.0011	0.8132
Polm	-0.7658	4.6783	-5.5624	0.0001	0.0011	0.99
Serinc1	0.5731	7.5497	5.5622	0.0001	0.0011	0.8025
Tpk1	1.2339	1.7196	5.5619	0.0001	0.0011	1.3887
Ankmy2	0.7733	4.4763	5.5599	0.0001	0.0011	0.992
Eef1akmt4	-0.9393	3.1356	-5.5596	0.0001	0.0011	1.2365
Gm28875	-3.1104	1.0799	-5.5583	0.0001	0.0011	1.4115
Ezh2	-0.6004	6.8968	-5.5565	0.0001	0.0011	0.8081
Impdh2-ps	-1.1804	2.2395	-5.5562	0.0001	0.0011	1.3474
Dhodh	-0.8712	4.2649	-5.5549	0.0001	0.0011	1.0532
Frk	1.724	3.9655	5.5544	0.0001	0.0011	1.1169
Htra2	-1.1118	5.9529	-5.5531	0.0001	0.0011	0.8545
Crbn	0.775	5.2364	5.5511	0.0001	0.0011	0.8881
Polr1d	-0.6261	6.63	-5.5497	0.0001	0.0011	0.8031
Cacul1	0.6453	6.3062	5.5488	0.0001	0.0011	0.8088
Nip7	-0.7928	6.2735	-5.5455	0.0001	0.0011	0.8147
Nacc1	-0.5555	7.3978	-5.5452	0.0001	0.0011	0.7794
Tsfm	-0.9462	4.2607	-5.545	0.0001	0.0011	1.0327
Hs1bp3	1.0809	5.2149	5.5419	0.0001	0.0011	0.8708
Uap1	-0.8406	6.1996	-5.5411	0.0001	0.0011	0.8116
Gm12925	3.1516	-2.5448	5.5406	0.0001	0.0011	0.7209
Adck2	1.1994	3.0215	5.5395	0.0001	0.0011	1.1854
Tmem127	0.5482	6.0634	5.5393	0.0001	0.0011	0.8021
Ak3	2.1185	4.0592	5.5385	0.0001	0.0011	1.0966
Rttn	-0.9775	4.3036	-5.5362	0.0001	0.0011	1.0226
Neurl1a	2.8078	0.9614	5.536	0.0001	0.0011	1.4043
Ube2j2	-0.5979	5.6155	-5.5351	0.0001	0.0011	0.8368
Narfl	0.86	4.5174	5.5337	0.0001	0.0011	0.9453
Prelid3b	-0.6496	6.1151	-5.5335	0.0001	0.0011	0.7991
Relt	-1.5369	2.0305	-5.5329	0.0001	0.0011	1.3557
Nr2c1	0.6467	4.0315	5.5321	0.0001	0.0011	1.0154
Nudcd2	-0.709	6.785	-5.5309	0.0001	0.0011	0.7693
Akr7a5	0.6229	4.6565	5.5304	0.0001	0.0011	0.9156
Mybl1	-1.2305	3.4314	-5.5301	0.0001	0.0011	1.1738
Baiap2	1.0894	7.3757	5.5273	0.0001	0.0011	0.7467
Nckap5	6.3821	-0.6001	5.5265	0.0001	0.0011	0.7213
Elf1	0.7091	5.973	5.5249	0.0001	0.0011	0.7839
Gm9855	-0.6417	4.0262	-5.5246	0.0001	0.0011	1.0334
Stard9	1.1034	4.5369	5.5234	0.0001	0.0011	0.9392
Egln3	-2.1792	7.3354	-5.5221	0.0001	0.0011	0.7595
Ip6k2	0.7139	5.2921	5.5215	0.0001	0.0011	0.8326
Snap47	0.7148	5.8211	5.5206	0.0001	0.0011	0.7854
Eml6	1.3415	2.3065	5.5201	0.0001	0.0011	1.2586
Wdr6	0.9861	7.2466	5.5193	0.0001	0.0011	0.7349
Cryab	4.4188	4.6046	5.5192	0.0001	0.0011	1.0955
Gm15470	-3.5696	-0.9834	-5.5187	0.0001	0.0011	0.9288
Prdm2	0.7387	6.035	5.5178	0.0001	0.0011	0.7712
Poc5	-0.5977	4.7772	-5.5175	0.0001	0.0011	0.8991
Dnaaf2	-1.1089	2.7469	-5.515	0.0001	0.0011	1.2327
Mapk6	-0.8646	8.4083	-5.5099	0.0001	0.0012	0.7072
Ccdc28b	1.1461	1.8192	5.506	0.0001	0.0012	1.2802

Mob3b	1.8372	3.1914	5.5053	0.0001	0.0012	1.146
Noa1	-0.8105	6.2037	-5.5045	0.0001	0.0012	0.7518
Psme3	-0.6278	7.505	-5.5038	0.0001	0.0012	0.7092
Plin3	1.0156	6.7208	5.5038	0.0001	0.0012	0.7194
Las1l	-0.6218	6.4897	-5.503	0.0001	0.0012	0.7332
Gpt2	-0.9544	5.759	-5.5029	0.0001	0.0012	0.7712
Hist1h2bc	1.7842	4.2628	5.5025	0.0001	0.0012	0.9805
Gcat	-1.3344	4.1133	-5.5	0.0001	0.0012	1.0175
Carm1	-0.7171	7.461	-5.4994	0.0001	0.0012	0.7028
Adsl	-0.8258	6.6526	-5.4981	0.0001	0.0012	0.7191
F8	2.6255	0.8982	5.4974	0.0001	0.0012	1.3455
Pcyt1b	-0.9065	6.2181	-5.4974	0.0001	0.0012	0.7388
Gm6634	-1.872	2.163	-5.4909	0.0001	0.0012	1.2937
Eif2b1	-0.5518	5.8251	-5.4908	0.0001	0.0012	0.7467
Setd7	0.6308	7.7887	5.4906	0.0001	0.0012	0.6799
Mrto4	-0.9732	6.2869	-5.4897	0.0001	0.0012	0.7258
Faap24	-0.9302	3.3979	-5.4886	0.0001	0.0012	1.0884
Megf8	0.5844	6.6991	5.4879	0.0001	0.0012	0.6939
Pole3	-0.6492	5.7323	-5.484	0.0001	0.0012	0.7446
Zbtb4	0.7117	3.7795	5.481	0.0001	0.0012	0.9686
Srd5a3	0.713	4.9003	5.4801	0.0001	0.0012	0.805
Iffo2	-1.0647	5.6066	-5.4794	0.0001	0.0012	0.7643
Nudt9	-0.8598	6.5173	-5.4792	0.0001	0.0012	0.6926
Hprt	-0.8319	6.9388	-5.4784	0.0001	0.0012	0.6787
Ssx2ip	-0.9186	5.545	-5.4783	0.0001	0.0012	0.7614
Cpq	1.8932	4.9141	5.4778	0.0001	0.0012	0.8444
Sigmar1	-0.6654	6.1448	-5.4775	0.0001	0.0012	0.7073
Gm21596	-1.1945	2.1398	-5.4753	0.0001	0.0012	1.2269
Dhrs4	0.7874	3.6395	5.4748	0.0001	0.0012	0.978
Smurf1	-0.5884	6.5328	-5.4717	0.0001	0.0012	0.6757
Gm4353	-1.3034	2.0677	-5.4714	0.0001	0.0012	1.2425
Sh2b3	-1.7372	5.6874	-5.471	0.0001	0.0012	0.7786
Ubn1	0.7722	6.8654	5.4703	0.0001	0.0012	0.6613
Fus	-0.5226	8.4226	-5.4693	0.0001	0.0012	0.6378
Drp2	-1.9047	0.1199	-5.464	0.0001	0.0012	1.2643
Pias1	1.0673	5.6466	5.4637	0.0001	0.0012	0.7105
Dbr1	-0.6702	5.1926	-5.4607	0.0001	0.0012	0.7648
Srsf4	-0.5497	6.077	-5.4606	0.0001	0.0012	0.6783
Ptcd3	-0.7579	6.9487	-5.4582	0.0001	0.0012	0.6449
Kras	-0.6946	6.6657	-5.458	0.0001	0.0012	0.6511
Gprc5a	-1.1834	5.6192	-5.4555	0.0001	0.0012	0.722
Vps52	0.4516	6.0211	5.4554	0.0001	0.0012	0.6643
Xaf1	3.2536	3.0956	5.4535	0.0001	0.0012	1.1648
Azin1	-0.8465	6.967	-5.4533	0.0001	0.0012	0.636
Gm26699	-2.1664	-1.6471	-5.452	0.0001	0.0012	1.0488
Fancm	-0.823	4.8549	-5.4516	0.0001	0.0012	0.8024
Ecsit	-0.5887	4.6852	-5.4497	0.0001	0.0012	0.7926
Sgf29	-0.8865	3.7814	-5.4481	0.0002	0.0012	0.9483
Ftl1	1.0184	10.5265	5.4472	0.0002	0.0012	0.5866
Pigp	0.8084	3.7243	5.4456	0.0002	0.0012	0.9266
Enox2	0.6549	4.7029	5.4444	0.0002	0.0013	0.7657
Cdc25a	-0.7256	5.2712	-5.4419	0.0002	0.0013	0.7272

Ltv1	-0.7064	5.1179	-5.4419	0.0002	0.0013	0.735
Lrch3	0.6076	5.4998	5.4414	0.0002	0.0013	0.6823
Gtf3a	-0.6558	5.9955	-5.4389	0.0002	0.0013	0.648
Dctn3	0.5764	4.9994	5.4387	0.0002	0.0013	0.7184
2810414N06Rik	1.4452	0.6629	5.4381	0.0002	0.0013	1.253
Ppig	-0.5839	7.0394	-5.4377	0.0002	0.0013	0.6065
Rnf167	0.7226	4.6542	5.4362	0.0002	0.0013	0.7603
Psma3	-0.6769	7.6576	-5.4359	0.0002	0.0013	0.5927
01-sept	2.7641	0.8825	5.4354	0.0002	0.0013	1.2525
Ube2r2	0.4903	7.3309	5.4338	0.0002	0.0013	0.5912
Eif3b	-0.7678	8.9422	-5.433	0.0002	0.0013	0.5726
Cnih4	-0.6143	6.6397	-5.432	0.0002	0.0013	0.6072
Gem	1.1239	3.3928	5.4308	0.0002	0.0013	0.9509
Magoh	-0.647	5.9173	-5.4296	0.0002	0.0013	0.6408
Tut7	0.7239	6.8384	5.4278	0.0002	0.0013	0.5911
Dner	-2.9812	-1.4453	-5.4261	0.0002	0.0013	0.8368
Zrsr1	1.3882	4.8273	5.4247	0.0002	0.0013	0.7384
Utp4	-0.9995	7.0713	-5.4227	0.0002	0.0013	0.585
Stambpl1	-0.9656	5.2288	-5.4207	0.0002	0.0013	0.7021
Pccb	0.7545	4.4385	5.4192	0.0002	0.0013	0.758
Zbtb43	0.6458	4.8348	5.4185	0.0002	0.0013	0.7178
Cmc2	-0.8166	4.3156	-5.4178	0.0002	0.0013	0.8108
Gm49064	1.6329	-0.1835	5.4165	0.0002	0.0013	1.2079
5430416N02Rik	-0.853	3.6463	-5.4151	0.0002	0.0013	0.9159
Rrs1	-0.8609	6.6425	-5.4151	0.0002	0.0013	0.5845
Tmem256	0.6303	4.8288	5.4142	0.0002	0.0013	0.6969
Ube2l6	1.7007	3.9011	5.4127	0.0002	0.0013	0.86
AW146154	1.5526	1.6507	5.4125	0.0002	0.0013	1.1737
Cenpp	-1.4878	3.5773	-5.4118	0.0002	0.0013	0.9875
Vat1	0.8575	8.2889	5.4109	0.0002	0.0013	0.5388
Rin1	-1.3439	5.6367	-5.4097	0.0002	0.0013	0.6606
Washc5	0.7242	7.6927	5.4068	0.0002	0.0013	0.5405
Pgap3	0.9903	2.8265	5.4054	0.0002	0.0013	1.0033
Gpr146	1.3161	3.6672	5.4053	0.0002	0.0013	0.8623
Zfp52	-1.2697	4.8393	-5.4053	0.0002	0.0013	0.7559
Thyn1	-0.9792	5.6597	-5.4037	0.0002	0.0013	0.6252
Mtss1l	1.9111	4.3281	5.4035	0.0002	0.0013	0.8035
Atp6v0c-ps2	0.5903	5.4095	5.4033	0.0002	0.0013	0.619
Nfkbia	0.7847	8.0493	5.4017	0.0002	0.0013	0.5279
Nisch	0.7602	8.6803	5.3999	0.0002	0.0013	0.5176
Rasa3	-0.6246	6.7846	-5.3979	0.0002	0.0013	0.5461
Gm32914	4.5925	-2.6034	5.3964	0.0002	0.0013	0.4595
Foxk2	-0.5157	7.4007	-5.3954	0.0002	0.0013	0.529
Micall1	0.6263	6.3234	5.395	0.0002	0.0013	0.5497
Ssb	-0.8318	7.9837	-5.3939	0.0002	0.0013	0.5184
Btbd9	0.7207	4.5594	5.3914	0.0002	0.0013	0.7012
Mrps26	-0.5817	5.5676	-5.3913	0.0002	0.0013	0.5979
Steap3	1.4676	7.0128	5.3911	0.0002	0.0013	0.5262
Retreg3	0.5192	6.2231	5.3906	0.0002	0.0013	0.5474
Ubc	0.5839	7.1129	5.3904	0.0002	0.0013	0.5207
Haus8	-1.0077	4.0971	-5.39	0.0002	0.0013	0.8124
Slc9a5	-1.5575	3.7459	-5.3898	0.0002	0.0013	0.9179

Zfp30	1.3268	2.2401	5.3891	0.0002	0.0013	1.0557
Lmna	-0.758	9.6573	-5.3889	0.0002	0.0013	0.4933
Thoc3	-0.794	6.314	-5.3872	0.0002	0.0013	0.5479
Psma7	-0.5781	8.1908	-5.3867	0.0002	0.0013	0.5018
Zdhhc21	-1.1325	4.5113	-5.3863	0.0002	0.0013	0.7672
Mta1	-0.5506	7.1824	-5.3851	0.0002	0.0013	0.5147
Tmem183a	-0.5747	6.8732	-5.3836	0.0002	0.0013	0.5195
Gramd4	-0.8014	7.0389	-5.3815	0.0002	0.0013	0.5134
Gadd45g	0.8526	3.9508	5.3803	0.0002	0.0013	0.7743
Glp1r	-4.9836	3.7701	-5.3797	0.0002	0.0013	1.1593
Tmem9b	0.8562	4.5123	5.3797	0.0002	0.0013	0.6922
AC134576.3	1.8962	1.5157	5.3791	0.0002	0.0013	1.126
P2rx6	4.1299	-1.9758	5.3783	0.0002	0.0013	0.6521
Ddx31	-0.7985	4.7538	-5.3776	0.0002	0.0013	0.688
Eif3j2	-0.8109	5.0583	-5.3775	0.0002	0.0013	0.6471
Npm3	-1.0866	6.2029	-5.377	0.0002	0.0013	0.5456
Tgds	-0.83	4.1246	-5.3746	0.0002	0.0014	0.7652
Nudt21	-0.5993	6.2891	-5.3728	0.0002	0.0014	0.5218
Caprin2	-0.8359	3.5542	-5.3726	0.0002	0.0014	0.8506
Lmbrd1	1.1224	5.2131	5.3719	0.0002	0.0014	0.5949
Gsap	2.0658	3.744	5.3717	0.0002	0.0014	0.8703
D430042O09Rik	0.723	4.6859	5.3713	0.0002	0.0014	0.6448
Spata5l1	-1.0293	2.7415	-5.3686	0.0002	0.0014	0.978
Rassf1	-0.5189	5.7913	-5.3685	0.0002	0.0014	0.5411
Gm16685	6.8103	-1.6466	5.3682	0.0002	0.0014	0.2784
Ccdc58	-0.9248	4.881	-5.3672	0.0002	0.0014	0.647
Wrnip1	-0.8008	6.3294	-5.3655	0.0002	0.0014	0.5115
Aimp2	-0.824	5.8114	-5.3647	0.0002	0.0014	0.544
Mdh2	-0.5927	8.5613	-5.3642	0.0002	0.0014	0.4597
Zfp784	2.6232	0.269	5.3636	0.0002	0.0014	1.1278
Cul7	0.6971	6.3697	5.3632	0.0002	0.0014	0.4953
Cyhr1	0.6578	6.4219	5.3628	0.0002	0.0014	0.4918
1700056E22Rik	1.4338	0.121	5.3624	0.0002	0.0014	1.1404
Psmd7	-0.5624	7.7828	-5.3623	0.0002	0.0014	0.4667
Gm20632	2.021	-0.0334	5.3611	0.0002	0.0014	1.1292
Sh3rf1	0.9525	4.3202	5.3608	0.0002	0.0014	0.6996
Commd3	0.6526	5.7692	5.3595	0.0002	0.0014	0.5166
Rfc3	-0.6332	5.6365	-5.3545	0.0002	0.0014	0.537
Rhob	1.2151	7.3051	5.3542	0.0002	0.0014	0.4573
Timm13	-0.7293	5.8579	-5.3532	0.0002	0.0014	0.517
Gm33347	-3.5535	-1.9575	-5.3514	0.0002	0.0014	0.5142
Egln2	0.5991	5.6066	5.351	0.0002	0.0014	0.518
Cpeb3	1.897	0.9808	5.3497	0.0002	0.0014	1.1158
Gpatch2	0.6977	4.4851	5.3492	0.0002	0.0014	0.6477
Cs	-0.4639	7.9236	-5.3486	0.0002	0.0014	0.4414
Prkab1	-0.5256	5.4994	-5.3484	0.0002	0.0014	0.5364
2900089D17Rik	2.1134	-0.5352	5.3482	0.0002	0.0014	1.0601
Trim62	2.5912	0.1431	5.347	0.0002	0.0014	1.1014
Lif	-2.2055	5.4823	-5.3469	0.0002	0.0014	0.6476
Npm3-ps1	-1.0776	3.535	-5.3444	0.0002	0.0014	0.83
Acad12	1.7995	1.7733	5.3443	0.0002	0.0014	1.0372
Rcor2	-1.0987	5.9547	-5.3439	0.0002	0.0014	0.5064

Eloc	-0.5887	6.5676	-5.3421	0.0002	0.0014	0.4579
Tlr6	3.114	1.6881	5.3406	0.0002	0.0014	1.0961
Ung	-1.8596	4.0529	-5.3395	0.0002	0.0014	0.8356
9330188P03Rik	-1.8397	2.7005	-5.3384	0.0002	0.0014	0.988
Fam43a	1.6572	2.6621	5.338	0.0002	0.0014	0.9321
Pias3	0.7127	5.2249	5.3353	0.0002	0.0014	0.5288
Hccs	-0.9463	5.5369	-5.329	0.0002	0.0014	0.5224
Cdca7l	-0.9085	6.0413	-5.329	0.0002	0.0014	0.4731
Epb41l2	0.9737	6.8483	5.3285	0.0002	0.0014	0.4241
Pfkp	-0.9976	7.3119	-5.3284	0.0002	0.0014	0.4178
Tmem39a	-0.8398	5.3702	-5.3271	0.0002	0.0014	0.5176
Chac1	-1.0341	3.1158	-5.3269	0.0002	0.0014	0.8377
D830050J10Rik	2.1141	-0.6532	5.3268	0.0002	0.0014	1.0206
Uevld	-0.7964	4.2622	-5.3262	0.0002	0.0014	0.6691
Smarcc2	0.6029	6.7806	5.3259	0.0002	0.0014	0.4204
Parp1	-0.7012	8.0273	-5.3254	0.0002	0.0014	0.4019
Gpx4	0.7911	7.5981	5.3254	0.0002	0.0014	0.4038
Cited2	2.0959	6.5062	5.3226	0.0002	0.0014	0.4318
Odf2l	-0.7182	4.9231	-5.3205	0.0002	0.0014	0.5622
Gm8203	-1.1867	1.5767	-5.3204	0.0002	0.0014	1.0356
Ctxn1	1.3063	5.5236	5.3183	0.0002	0.0014	0.4746
Scrib	-0.4469	6.9844	-5.318	0.0002	0.0014	0.4057
Homez	0.8315	3.0114	5.3175	0.0002	0.0014	0.8168
Plekha4	3.5724	-1.7184	5.3172	0.0002	0.0014	0.6295
Zfp184	1.3148	3.1197	5.3168	0.0002	0.0014	0.827
Ak4	-1.1648	5.8496	-5.3163	0.0002	0.0014	0.4759
Dctd	-1.2209	4.7348	-5.3157	0.0002	0.0014	0.6159
Aen	-0.9381	4.9434	-5.3154	0.0002	0.0014	0.5733
07-sept	-0.7371	8.1621	-5.3131	0.0002	0.0015	0.3797
Nedd4	0.525	10.9286	5.3122	0.0002	0.0015	0.3583
Xpc	0.794	5.0617	5.3112	0.0002	0.0015	0.5013
Alpk1	1.9164	2.1723	5.3108	0.0002	0.0015	0.9638
Rsbn1	-0.8049	5.4811	-5.3086	0.0002	0.0015	0.481
Atp8b3	2.9182	0.4432	5.3069	0.0002	0.0015	1.0513
Cep76	-1.1437	5.3868	-5.3061	0.0002	0.0015	0.5019
Gtf2h2	-0.8527	5.2461	-5.3057	0.0002	0.0015	0.5023
Pigk	0.6302	5.6472	5.3051	0.0002	0.0015	0.4336
Stk40	-0.9769	4.3375	-5.3049	0.0002	0.0015	0.631
Mccc1	0.754	4.5915	5.3044	0.0002	0.0015	0.5457
Axin2	2.2367	1.9253	5.3026	0.0002	0.0015	0.9931
Atp5b	-0.481	10.0206	-5.3011	0.0002	0.0015	0.343
Wdfy2	0.6365	4.7045	5.2999	0.0002	0.0015	0.5318
Fam98a	-0.6472	6.1095	-5.2997	0.0002	0.0015	0.4098
Lonp2	0.7576	5.5474	5.2992	0.0002	0.0015	0.4332
Efna4	1.3496	3.8339	5.2987	0.0002	0.0015	0.6672
Timm8a1	-1.4656	3.7321	-5.2971	0.0002	0.0015	0.7712
Ypel1	3.0745	2.3335	5.297	0.0002	0.0015	0.979
Galnt10	0.7995	5.7728	5.2968	0.0002	0.0015	0.4155
Hnrnpab	-0.6669	10.0279	-5.2967	0.0002	0.0015	0.3358
Psmg3	-0.6365	4.4025	-5.2965	0.0002	0.0015	0.586
Fpgs	-0.8175	5.0402	-5.2963	0.0002	0.0015	0.5131
Psmd14	-0.8302	7.2273	-5.296	0.0002	0.0015	0.365

Nckap1l	-3.1959	0.3213	-5.2956	0.0002	0.0015	0.9542
Snx8	0.9165	5.0179	5.2954	0.0002	0.0015	0.4835
Atp6v1g1	0.6614	6.2038	5.2935	0.0002	0.0015	0.3832
Trnp1	2.4413	-0.6158	5.2922	0.0002	0.0015	0.988
Rad23b	-0.6164	8.3697	-5.2922	0.0002	0.0015	0.341
Fgfr1op2	-0.4915	6.5338	-5.2914	0.0002	0.0015	0.3725
Foxj1	3.0476	-2.2947	5.2911	0.0002	0.0015	0.5662
Egln1	-0.7265	7.15	-5.2897	0.0002	0.0015	0.3557
Snhg1	-1.0502	5.7797	-5.2897	0.0002	0.0015	0.4274
Atp11b	-0.788	7.0871	-5.2893	0.0002	0.0015	0.3574
Eif2ak2	0.5717	5.4859	5.2885	0.0002	0.0015	0.425
Snx29	1.8236	1.9762	5.2882	0.0002	0.0015	0.9372
Kif1b	0.7834	6.6632	5.2881	0.0002	0.0015	0.3597
Ppp1r18	-0.6996	7.1355	-5.2876	0.0002	0.0015	0.3517
Wincr1	-2.7263	2.5484	-5.2874	0.0002	0.0015	0.995
Fam120a	-0.6911	8.9041	-5.2874	0.0002	0.0015	0.3273
Rap1gap	-1.5211	3.3962	-5.2864	0.0002	0.0015	0.7878
Tcf7l2	1.1548	2.9558	5.2851	0.0002	0.0015	0.8039
Zfp280c	-1.0574	5.3425	-5.285	0.0002	0.0015	0.472
Prdx3	-0.639	5.8907	-5.2812	0.0002	0.0015	0.388
Habp4	0.8958	5.2179	5.2811	0.0002	0.0015	0.4408
Snu13	-0.8339	6.6208	-5.2793	0.0002	0.0015	0.3543
Fam32a	0.6315	6.5194	5.277	0.0002	0.0015	0.3435
Gnl3	-0.8307	7.3041	-5.2762	0.0002	0.0015	0.331
Tango2	0.993	3.4834	5.2753	0.0002	0.0015	0.6684
Dok1	-1.5411	5.4498	-5.2737	0.0002	0.0015	0.4662
Anapc1	-0.484	8.167	-5.2724	0.0002	0.0015	0.3096
Gm4739	-2.8366	-0.3467	-5.2692	0.0002	0.0015	0.8699
Tmem106b	1.3419	7.9741	5.2687	0.0002	0.0015	0.3045
Gatad1	-0.7344	6.9345	-5.2685	0.0002	0.0015	0.324
Wdr77	-0.9296	6.545	-5.2683	0.0002	0.0015	0.3417
Trim26	0.9835	4.7838	5.268	0.0002	0.0015	0.4716
Afg3l2	-0.6015	7.0106	-5.2678	0.0002	0.0015	0.3203
Cog2	0.5964	5.048	5.2675	0.0002	0.0015	0.4275
Gsn	1.3657	8.4806	5.265	0.0002	0.0015	0.2916
Tcerg1	-0.7814	7.4861	-5.265	0.0002	0.0015	0.3084
Gsk3b	0.687	6.9097	5.2639	0.0002	0.0015	0.3121
Gcsh	-0.9092	6.285	-5.2631	0.0002	0.0015	0.3418
Zfp598	-0.5647	6.2352	-5.2628	0.0002	0.0015	0.3386
Mgst1	2.4379	5.7616	5.2604	0.0002	0.0015	0.4013
Gm10282	-1.4413	1.1403	-5.2561	0.0002	0.0015	0.9631
Cmc1	0.7098	4.5669	5.2544	0.0002	0.0016	0.4637
Ogfr	0.9722	6.3752	5.2537	0.0002	0.0016	0.3132
Znrf3	-0.871	5.0243	-5.2502	0.0002	0.0016	0.4401
Kcnn1	2.805	0.8021	5.2498	0.0002	0.0016	0.9695
Rrn3	-0.5183	6.919	-5.2492	0.0002	0.0016	0.2911
Herpud2	0.6425	5.2408	5.2476	0.0002	0.0016	0.3786
Tnpo1	-0.668	8.591	-5.2467	0.0002	0.0016	0.2619
Calm3	-0.5676	7.9258	-5.2463	0.0002	0.0016	0.2684
Noc3l	-0.7167	6.0824	-5.2439	0.0002	0.0016	0.3193
Cbfb	-0.6751	7.4359	-5.2435	0.0002	0.0016	0.2729
Tsr1	-0.9435	7.066	-5.2434	0.0002	0.0016	0.2814

Flna	0.8934	10.6692	5.2425	0.0002	0.0016	0.2405
Inpp4b	-2.4758	3.5859	-5.2414	0.0002	0.0016	0.8026
Plekhg5	1.5775	3.4894	5.2404	0.0002	0.0016	0.6487
Esd	-1.0495	8.4876	-5.2395	0.0002	0.0016	0.2506
H2-T22	1.8227	1.9539	5.2375	0.0002	0.0016	0.8644
Atp9b	0.7082	5.9863	5.2364	0.0002	0.0016	0.3004
Guf1	-0.6654	4.6966	-5.2345	0.0002	0.0016	0.4408
Ccs	0.7006	4.7474	5.2344	0.0002	0.0016	0.4071
Stard13	1.545	4.0439	5.2326	0.0002	0.0016	0.5501
Gpr108	0.8049	5.4852	5.2309	0.0002	0.0016	0.3254
Arfgef2	0.9478	6.0333	5.2308	0.0002	0.0016	0.289
Supv3l1	-0.5677	5.0587	-5.2308	0.0002	0.0016	0.3841
Mgat1	0.5347	6	5.2299	0.0002	0.0016	0.2849
Agpat3	0.836	6.445	5.229	0.0002	0.0016	0.2643
Nbr1	0.6613	7.6653	5.2289	0.0002	0.0016	0.2396
Adamts14	-1.367	4.7088	-5.2288	0.0002	0.0016	0.4826
Wdr13	0.5786	5.2064	5.228	0.0002	0.0016	0.3465
Fer1l6	-3.041	0.8558	-5.228	0.0002	0.0016	0.9168
Pcdhb20	1.8114	0.0792	5.2274	0.0002	0.0016	0.936
Noxo1	1.8203	0.1218	5.2273	0.0002	0.0016	0.9352
Znhit6	-0.6989	5.2322	-5.2264	0.0002	0.0016	0.3622
Kcmf1	-0.4312	7.0902	-5.2258	0.0002	0.0016	0.2464
Gm4544	1.9944	1.0072	5.2228	0.0002	0.0016	0.919
Zbtb1	0.6026	5.7692	5.2222	0.0002	0.0016	0.2907
Elk3	-0.7731	6.6099	-5.2218	0.0002	0.0016	0.2537
Arhgap23	1.5927	5.4011	5.2206	0.0002	0.0016	0.3336
Mri1	-0.8971	4.0821	-5.2196	0.0002	0.0016	0.5253
Wbp1	0.9469	5.1484	5.2191	0.0002	0.0016	0.3366
Zfp36l1	1.0127	6.5588	5.2171	0.0002	0.0016	0.2456
Ppp1ca	-0.5236	8.7318	-5.2166	0.0002	0.0016	0.2076
Ikbkg	0.7075	6.3342	5.2152	0.0002	0.0016	0.2466
Haus2	-0.6479	4.9665	-5.2127	0.0002	0.0016	0.3687
Nelfa	-0.5749	5.6952	-5.2109	0.0002	0.0016	0.2879
Gas2l3	-1.2271	4.9894	-5.2107	0.0002	0.0016	0.3995
Hnrnpll	-0.6772	6.9924	-5.2091	0.0002	0.0016	0.2222
Tnfsf13	2.8365	-1.0796	5.2088	0.0002	0.0016	0.7906
Gm47175	-1.9837	0.5821	-5.2071	0.0002	0.0016	0.9037
Cep57l1	-0.8664	3.5082	-5.2058	0.0002	0.0016	0.6017
Scarb1	-0.5238	7.4227	-5.2042	0.0002	0.0017	0.2047
Fmn1	1.7068	4.1952	5.2016	0.0002	0.0017	0.4726
Rap1a	-0.6687	6.5514	-5.1988	0.0002	0.0017	0.2169
Hint2	1.0428	1.8671	5.1978	0.0002	0.0017	0.7627
Zer1	0.7875	4.5877	5.1971	0.0002	0.0017	0.374
Dnpep	0.722	6.144	5.1963	0.0002	0.0017	0.2191
Fam216a	-0.6679	5.5163	-5.1945	0.0002	0.0017	0.2769
Crebbp	0.5933	6.1933	5.1931	0.0002	0.0017	0.217
Gbe1	-0.717	6.143	-5.1931	0.0002	0.0017	0.2245
Oat	-0.5578	6.7631	-5.1929	0.0002	0.0017	0.198
Atg12	0.4806	6.3151	5.1929	0.0002	0.0017	0.2099
Cdkn2aipnl	-1.0371	6.7103	-5.1927	0.0002	0.0017	0.2067
Agtrap	1.0695	4.5718	5.1891	0.0002	0.0017	0.3642
Gm11175	1.713	2.2833	5.1871	0.0002	0.0017	0.7057

Pithd1	-0.7803	4.7321	-5.1849	0.0002	0.0017	0.3575
Dars	-0.6003	7.3738	-5.1839	0.0002	0.0017	0.1708
Hps1	0.8424	4.921	5.1837	0.0002	0.0017	0.2973
Ldb1	0.6834	6.9391	5.1836	0.0002	0.0017	0.1751
Tpgs1	0.8659	3.9505	5.1833	0.0002	0.0017	0.4452
Gba2	1.2302	2.6985	5.1832	0.0002	0.0017	0.6574
Tmem192	0.6411	4.4975	5.1825	0.0002	0.0017	0.3505
Areg	-5.5772	2.4635	-5.181	0.0002	0.0017	0.789
Rnf115	0.6046	6.207	5.1807	0.0002	0.0017	0.1926
D430040D24Rik	1.7216	0.3857	5.1801	0.0002	0.0017	0.86
Celf1	-0.5306	7.7682	-5.1788	0.0002	0.0017	0.1564
Pheta1	1.079	4.0688	5.1787	0.0002	0.0017	0.4197
Wdr19	1.073	4.6215	5.1772	0.0002	0.0017	0.3351
Nfx1	-0.5641	6.6581	-5.1752	0.0002	0.0017	0.1726
Zfp647	1.0176	2.7225	5.1751	0.0002	0.0017	0.6356
Ftsj3	-0.7019	7.6172	-5.174	0.0002	0.0017	0.1506
Tor1aip1	0.5847	6.426	5.1737	0.0002	0.0017	0.173
AW047730	2.6371	-1.9973	5.1721	0.0002	0.0017	0.4917
Xrcc6	-0.7799	6.365	-5.1705	0.0002	0.0017	0.1763
Nudt5	-0.899	6.3542	-5.1692	0.0002	0.0017	0.1806
Ppp1r9b	0.5741	6.8533	5.1682	0.0002	0.0017	0.1501
Hsp90b1	-0.7139	10.0988	-5.1672	0.0002	0.0017	0.1148
Me3	4.906	-0.6104	5.1655	0.0002	0.0017	0.5404
Fbxl17	0.6168	5.1357	5.1637	0.0002	0.0017	0.2476
Fhl3	-1.6499	5.7776	-5.1621	0.0002	0.0017	0.2524
Wdr55	-0.6647	5.7886	-5.162	0.0002	0.0017	0.1997
Filip1l	1.4774	5.1417	5.1619	0.0002	0.0017	0.2603
Sfpq	-0.6449	8.3788	-5.1614	0.0002	0.0017	0.1182
9130023H24Rik	1.0693	2.1427	5.1591	0.0002	0.0017	0.6754
Gins4	-0.5452	5.8197	-5.1587	0.0002	0.0017	0.1854
Tgfbi	2.2029	6.8152	5.1571	0.0002	0.0018	0.1422
Man2b1	1.2377	5.4264	5.1527	0.0002	0.0018	0.2049
Map2k3os	-1.5978	1.9335	-5.1516	0.0002	0.0018	0.7511
Ppm1l	0.919	6.3043	5.1513	0.0002	0.0018	0.1413
Dnase2a	1.0605	3.7165	5.1505	0.0002	0.0018	0.4299
Mir17hg	-1.5779	1.4341	-5.1494	0.0002	0.0018	0.7866
Gm47813	-2.8918	-0.567	-5.1465	0.0002	0.0018	0.6316
Acaa2	0.9003	5.3346	5.1465	0.0002	0.0018	0.1952
Atf1	-0.8825	6.5105	-5.1463	0.0002	0.0018	0.1324
Tnip2	0.8834	3.4888	5.1444	0.0002	0.0018	0.4526
Stxbp4	0.5157	4.7485	5.1441	0.0002	0.0018	0.2608
Ssh3	0.9552	4.0764	5.1438	0.0002	0.0018	0.3656
Pop5	-0.8727	5.0958	-5.1423	0.0002	0.0018	0.2447
Mrps35	-0.5118	5.7353	-5.142	0.0002	0.0018	0.1577
Ccdc173	1.2598	1.4835	5.1403	0.0002	0.0018	0.6925
Dysf	1.7121	3.0913	5.1402	0.0002	0.0018	0.5744
Tpd52l2	0.6592	6.6633	5.1386	0.0002	0.0018	0.1036
Zfp87	0.8013	3.5562	5.1362	0.0002	0.0018	0.4192
Ccpg1	1.4469	2.7051	5.1357	0.0002	0.0018	0.5959
Fam107a	4.6249	-1.5082	5.1338	0.0003	0.0018	0.4757
Pcdhb9	2.9421	-1.2245	5.1337	0.0003	0.0018	0.668
Shroom3	2.0086	3.2446	5.1337	0.0003	0.0018	0.5377

Gmfb	-0.6296	7.2075	-5.1334	0.0003	0.0018	0.088
Ltbp3	-0.749	8.0843	-5.1332	0.0003	0.0018	0.073
Phb	-0.6621	6.9788	-5.1322	0.0003	0.0018	0.0897
Carnmt1	-0.8699	3.8276	-5.1303	0.0003	0.0018	0.4234
Pin1	-0.7839	6.0795	-5.1294	0.0003	0.0018	0.1239
Pnrc2	0.5109	6.2403	5.1269	0.0003	0.0018	0.0972
Aph1c	2.7699	-0.3419	5.1255	0.0003	0.0018	0.7511
Gps1	-0.5965	6.9042	-5.1246	0.0003	0.0018	0.0787
Tars	-0.8005	7.4584	-5.1246	0.0003	0.0018	0.0682
Utrn	0.8617	6.482	5.124	0.0003	0.0018	0.0866
Sdc2	1.2524	6.6155	5.1238	0.0003	0.0018	0.0845
Tifa	1.8238	2.6823	5.1226	0.0003	0.0018	0.574
Spa17	1.801	0.686	5.1224	0.0003	0.0018	0.7631
Naalad2	2.8397	-3.0808	5.1219	0.0003	0.0018	0.3079
Mrtfb	0.7427	5.2756	5.1218	0.0003	0.0018	0.1643
Sgsh	1.222	3.8306	5.1213	0.0003	0.0018	0.3661
Nol11	-0.6472	6.7708	-5.1208	0.0003	0.0018	0.0763
Cyb5r1	-0.5111	5.8054	-5.1203	0.0003	0.0018	0.1209
Mre11a	-1.2188	6.4996	-5.119	0.0003	0.0018	0.0953
Map3k6	-1.535	4.1955	-5.1186	0.0003	0.0018	0.3918
Cpxm1	1.4743	0.8256	5.1184	0.0003	0.0018	0.7477
Kdm5a	0.4347	6.9557	5.1179	0.0003	0.0018	0.063
Hmgn2	-1.1987	6.0896	-5.115	0.0003	0.0018	0.1148
Phldb3	1.5426	1.4439	5.1134	0.0003	0.0018	0.6912
Tmem11	-0.6794	5.6169	-5.1127	0.0003	0.0018	0.127
Rcan1	0.702	4.3019	5.1114	0.0003	0.0019	0.2587
Dnajc15	-0.7726	4.4593	-5.1101	0.0003	0.0019	0.2638
Ech1	0.7549	5.3525	5.11	0.0003	0.0019	0.1284
Mrpl20	-0.5779	5.7349	-5.1095	0.0003	0.0019	0.1065
Kiz	0.6232	4.8573	5.1074	0.0003	0.0019	0.1816
Ube3b	0.6566	6.7756	5.1069	0.0003	0.0019	0.0464
Ahnak2	2.4137	5.3175	5.1057	0.0003	0.0019	0.174
Zfp217	-0.5308	6.0144	-5.1055	0.0003	0.0019	0.0812
Gm17709	-2.8598	0.2654	-5.1052	0.0003	0.0019	0.6985
Tmem63b	0.5712	6.4046	5.1046	0.0003	0.0019	0.0552
Stx18	0.6396	4.4903	5.1045	0.0003	0.0019	0.2174
Polk	-0.8268	4.2907	-5.1043	0.0003	0.0019	0.2909
Rab2b	0.7708	3.3884	5.1026	0.0003	0.0019	0.3969
Npdc1	1.2617	5.0712	5.102	0.0003	0.0019	0.1647
Gm47034	3.9364	-2.1659	5.1018	0.0003	0.0019	0.292
Zfp930	-0.8491	4.3602	-5.1014	0.0003	0.0019	0.2816
Rbak	0.9269	3.3744	5.1002	0.0003	0.0019	0.3992
Polr3h	-0.9452	5.728	-5.1	0.0003	0.0019	0.1093
H2afy	-0.6083	7.4448	-5.0996	0.0003	0.0019	0.0252
Dpf1	-2.2631	2.2941	-5.0961	0.0003	0.0019	0.6822
Akap8	-0.5537	6.9281	-5.0958	0.0003	0.0019	0.0294
Tsr2	-0.9474	5.283	-5.0956	0.0003	0.0019	0.1502
Dnah8	-2.4647	-1.2439	-5.0943	0.0003	0.0019	0.572
Ssh1	0.8486	4.8327	5.0933	0.0003	0.0019	0.161
Nkap	-0.5303	4.9149	-5.0918	0.0003	0.0019	0.1594
Serinc3	0.9749	7.4309	5.0915	0.0003	0.0019	0.0083
Rmnd5a	0.6374	6.9314	5.0893	0.0003	0.0019	0.0138

Ube2k	-0.5466	7.1782	-5.0883	0.0003	0.0019	0.01
Stx4a	0.5101	5.6994	5.0874	0.0003	0.0019	0.0599
Dnajc28	1.3862	1.6989	5.0873	0.0003	0.0019	0.6177
Pkn1	0.9266	6.0196	5.0859	0.0003	0.0019	0.0405
Ak6	-0.6858	6.0641	-5.0842	0.0003	0.0019	0.0445
Ndufaf6	-0.9178	2.7886	-5.0835	0.0003	0.0019	0.4929
Ip6k3	2.4629	-0.0988	5.0829	0.0003	0.0019	0.6947
Ang2	2.7501	-0.0721	5.0822	0.0003	0.0019	0.6877
Fam227a	2.0767	-0.5853	5.0816	0.0003	0.0019	0.6849
Necap1	-0.4721	5.881	-5.0815	0.0003	0.0019	0.0473
Slc35f6	0.7343	4.9377	5.0815	0.0003	0.0019	0.1302
Fhl2	-1.0108	5.6043	-5.0813	0.0003	0.0019	0.0872
Dut	-1.6958	6.7728	-5.0796	0.0003	0.0019	0.0265
Gm42609	-3.0422	-0.5354	-5.0788	0.0003	0.0019	0.5716
Phf6	-0.6589	6.7795	-5.0777	0.0003	0.0019	0.003
Bcar1	0.5988	6.6385	5.0776	0.0003	0.0019	0.0001
Hid1	2.5254	4.1509	5.0757	0.0003	0.0019	0.3261
Echdc1	0.8527	5.423	5.0746	0.0003	0.0019	0.0653
Ero1l	-1.7273	8.2222	-5.0741	0.0003	0.0019	-0.0272
Gm43034	1.6055	0.7242	5.0741	0.0003	0.0019	0.6815
Acot7	-0.6038	6.4417	-5.074	0.0003	0.0019	0.0042
Amotl2	1.7139	5.5807	5.0739	0.0003	0.0019	0.0691
Nt5dc3	-0.6915	5.1492	-5.0735	0.0003	0.0019	0.1154
Bhlhe41	1.8884	4.576	5.0721	0.0003	0.0019	0.2002
Selenom	2.0116	4.474	5.0717	0.0003	0.0019	0.2218
Psme1	0.9256	6.7085	5.071	0.0003	0.0019	-0.0129
Syngap1	1.5562	2.1182	5.0707	0.0003	0.0019	0.5732
Bdp1	-0.6228	6.3308	-5.0676	0.0003	0.0019	0.0032
Herc2	0.9389	6.4951	5.0663	0.0003	0.002	-0.0116
Pfkfb1	-1.1054	2.2335	-5.0654	0.0003	0.002	0.561
Slc11a2	0.7015	6.6423	5.0653	0.0003	0.002	-0.0208
Apeh	0.4159	5.8856	5.0652	0.0003	0.002	0.0102
Ntn1	-1.0292	6.2873	-5.0646	0.0003	0.002	0.0086
Cpsf4	-0.7527	5.0954	-5.0641	0.0003	0.002	0.1052
Rab13	1.1737	4.359	5.0629	0.0003	0.002	0.1914
Tlr3	1.6756	1.9464	5.0627	0.0003	0.002	0.5641
Rnaseh2b	-0.9224	3.7894	-5.0605	0.0003	0.002	0.3169
Gnpat	-0.6159	6.7944	-5.0599	0.0003	0.002	-0.0305
Trim66	-3.9879	0.9893	-5.0577	0.0003	0.002	0.6075
Ccpg1os	1.1984	2.0035	5.0573	0.0003	0.002	0.5196
Cluh	-0.6193	7.2557	-5.0567	0.0003	0.002	-0.0447
Atp7a	0.7229	5.5329	5.0555	0.0003	0.002	0.0225
Mmp11	2.532	3.9455	5.0537	0.0003	0.002	0.3077
Supt16	-0.8227	7.8831	-5.0521	0.0003	0.002	-0.0621
Gm36401	-1.7563	0.301	-5.0508	0.0003	0.002	0.6546
Gskip	0.7201	4.5482	5.0508	0.0003	0.002	0.1166
Itfg1	0.6593	6.2052	5.0506	0.0003	0.002	-0.031
Trnt1	-0.5822	6.1592	-5.0506	0.0003	0.002	-0.0187
Rnd2	0.8977	3.6233	5.0499	0.0003	0.002	0.2571
Gm39473	-3.3798	0.0814	-5.0493	0.0003	0.002	0.5275
Ndufab1	-0.6891	5.6512	-5.0491	0.0003	0.002	0.0115
Tgfbr2	0.7057	6.0418	5.0476	0.0003	0.002	-0.0252

Apol9a	2.9754	2.8591	5.046	0.0003	0.002	0.5077
Slu7	0.54	6.1451	5.044	0.0003	0.002	-0.0412
Ppp1r14b	-0.7202	7.5187	-5.043	0.0003	0.002	-0.0737
Vapa	-0.5534	7.5947	-5.0418	0.0003	0.002	-0.0771
Dctn2	0.5361	6.8467	5.0413	0.0003	0.002	-0.0686
Arid1b	0.7781	6.3238	5.0405	0.0003	0.002	-0.052
Tbc1d17	0.6533	5.0547	5.04	0.0003	0.002	0.0357
Ccser2	0.6802	6.4373	5.0393	0.0003	0.002	-0.0593
Arhgef6	3.3291	-2.2065	5.0372	0.0003	0.002	0.349
Ext2	0.458	6.7909	5.0369	0.0003	0.002	-0.0746
Tmem176a	1.6844	7	5.0366	0.0003	0.002	-0.0761
Sord	1.2507	5.8933	5.0356	0.0003	0.002	-0.0358
Nicn1	0.7591	4.4243	5.0351	0.0003	0.002	0.1136
Ammecr1l	0.5446	5.7131	5.0337	0.0003	0.002	-0.0297
Pramef8	0.6582	4.6672	5.0336	0.0003	0.002	0.0759
Kdm5b	0.6864	6.2607	5.033	0.0003	0.002	-0.0633
Sf3b4	-0.5247	6.815	-5.0322	0.0003	0.002	-0.0784
Rnf168	-0.7368	5.4679	-5.0312	0.0003	0.002	0.0092
Tsen2	-0.8325	3.8716	-5.0309	0.0003	0.002	0.2188
Gm12856	-3.6607	-1.8655	-5.0298	0.0003	0.002	0.141
Fh1	-0.4644	6.8548	-5.0276	0.0003	0.002	-0.0892
Ube2e2	0.8312	4.9082	5.0272	0.0003	0.002	0.0533
Cbwd1	-0.696	4.2384	-5.0269	0.0003	0.002	0.1508
Myc	-0.9241	8.7173	-5.0229	0.0003	0.0021	-0.1234
D1Ertd622e	-0.7859	5.4413	-5.0224	0.0003	0.0021	-0.0017
Gak	0.5296	6.5942	5.0212	0.0003	0.0021	-0.0968
Psmc2	-0.4721	7.9249	-5.0212	0.0003	0.0021	-0.1185
Ptbp3	-0.6482	6.9031	-5.0207	0.0003	0.0021	-0.0978
Bag4	-0.9277	5.4126	-5.0201	0.0003	0.0021	0.0063
Myg1	-0.8111	5.7413	-5.0198	0.0003	0.0021	-0.0434
Rfwd3	-0.7281	6.9263	-5.0194	0.0003	0.0021	-0.1011
Cxcr3	-4.861	-1.3106	-5.0179	0.0003	0.0021	0.049
Gm37494	0.8848	3.7416	5.0172	0.0003	0.0021	0.2147
Golgb1	0.7271	6.8203	5.0168	0.0003	0.0021	-0.109
Tceal9	0.6054	7.2806	5.0168	0.0003	0.0021	-0.1185
Cyp4f17	1.5933	1.5206	5.0163	0.0003	0.0021	0.5261
Stx11	1.6173	3.5055	5.0154	0.0003	0.0021	0.2593
Phyh	2.4181	3.9156	5.0139	0.0003	0.0021	0.2469
Gm5596	-2.4219	-1.0062	-5.0125	0.0003	0.0021	0.4366
Spns1	0.6257	5.5647	5.0122	0.0003	0.0021	-0.0571
Grcc10	0.8361	4.676	5.0118	0.0003	0.0021	0.0335
Rnf146	0.5449	5.0346	5.0105	0.0003	0.0021	-0.0036
Gm19619	-2.8919	-3.0516	-5.01	0.0003	0.0021	0.132
Pcsk4	-1.2094	2.1429	-5.01	0.0003	0.0021	0.4797
Ints11	-0.713	6.1976	-5.0096	0.0003	0.0021	-0.0946
Slc4a7	-0.7807	6.3395	-5.009	0.0003	0.0021	-0.0963
Prmt1	-0.9036	7.7773	-5.0081	0.0003	0.0021	-0.137
Gm20559	2.826	-2.1486	5.0062	0.0003	0.0021	0.3562
Mplkip	-0.6277	4.5063	-5.0056	0.0003	0.0021	0.074
Mettl21a	0.5178	4.3607	5.0047	0.0003	0.0021	0.0711
Tmem209	-0.5383	5.7793	-5.0044	0.0003	0.0021	-0.0722
Naa16	-0.8756	5.0577	-5.0026	0.0003	0.0021	0.0138

Cd274	1.4775	1.0111	5.0024	0.0003	0.0021	0.5388
Leo1	-0.5417	5.632	-5.0019	0.0003	0.0021	-0.0664
Pds5a	-0.5525	7.8382	-5.0002	0.0003	0.0021	-0.1523
Jak2	1.0222	5.9971	4.9994	0.0003	0.0021	-0.1044
Urb2	-0.9104	6.0753	-4.9976	0.0003	0.0021	-0.095
Zkscan5	0.9781	3.3475	4.997	0.0003	0.0021	0.2263
Zbtb37	0.7624	3.9431	4.9957	0.0003	0.0021	0.1283
Stk38	0.4331	6.5726	4.9948	0.0003	0.0021	-0.1409
Slc38a6	0.8323	3.3247	4.9948	0.0003	0.0021	0.2254
Apol9b	2.5926	2.1542	4.9948	0.0003	0.0021	0.4561
Bop1	-0.7006	6.8852	-4.9943	0.0003	0.0021	-0.1442
Mpc2	0.7343	5.0333	4.9912	0.0003	0.0021	-0.0429
Pak1ip1	-0.6465	6.7831	-4.9903	0.0003	0.0021	-0.15
Gon7	-0.6714	3.4057	-4.9898	0.0003	0.0021	0.2321
Snx10	0.7985	5.5908	4.9864	0.0003	0.0021	-0.1046
Tmcc1	0.5832	4.6088	4.9864	0.0003	0.0021	0.0094
Golph3l	0.831	5.411	4.9855	0.0003	0.0022	-0.089
Abraxas1	-0.8764	3.8291	-4.9844	0.0003	0.0022	0.1595
Bccip	-0.6131	5.9874	-4.9836	0.0003	0.0022	-0.125
Fn3krp	0.6634	4.1753	4.9833	0.0003	0.0022	0.0669
Yjefn3	1.759	-0.3024	4.9804	0.0003	0.0022	0.5486
Cyc1	-0.672	7.2427	-4.9802	0.0003	0.0022	-0.1782
Naaladl2	4.0562	-0.3406	4.9788	0.0003	0.0022	0.3815
Scamp2	0.7282	5.3722	4.9758	0.0003	0.0022	-0.1021
Gm13461	-1.3143	1.0497	-4.9752	0.0003	0.0022	0.516
Paxip1	-0.7393	5.969	-4.9751	0.0003	0.0022	-0.1335
Fis1	0.4743	5.7358	4.9733	0.0003	0.0022	-0.1424
Usf1	0.7305	6.289	4.9727	0.0003	0.0022	-0.1701
Got2	-0.729	7.9765	-4.9711	0.0003	0.0022	-0.2048
Chic2	-0.7034	5.9719	-4.9699	0.0003	0.0022	-0.1454
Krt7	2.6502	0.5854	4.9698	0.0003	0.0022	0.5363
Cacnb3	1.5023	5.4161	4.9692	0.0003	0.0022	-0.104
Ermp1	-0.9331	6.8129	-4.9688	0.0003	0.0022	-0.185
Kbtbd7	1.208	2.6852	4.9674	0.0003	0.0022	0.3052
Fam185a	-1.0735	4.686	-4.9672	0.0003	0.0022	0.014
Arl6ip4	-0.5406	5.1239	-4.9668	0.0003	0.0022	-0.0867
Gm47205	2.3226	-0.9967	4.9666	0.0003	0.0022	0.4717
Fam131b	-1.8966	2.1344	-4.9652	0.0003	0.0022	0.4438
Carns1	1.6449	0.5578	4.962	0.0003	0.0022	0.4962
Zfp558	2.019	-0.0314	4.9618	0.0003	0.0022	0.5244
Stat3	0.8188	7.453	4.9613	0.0003	0.0022	-0.2174
Phip	0.612	7.3659	4.961	0.0003	0.0022	-0.2155
Vmac	1.2588	2.8493	4.9602	0.0003	0.0022	0.2614
Map3k12	0.6124	4.8057	4.9591	0.0003	0.0022	-0.0676
Hspb1	1.6084	3.8609	4.9585	0.0003	0.0022	0.0862
Bid	0.7132	5.7516	4.9565	0.0003	0.0022	-0.1653
Olfm1	-1.2904	6.9453	-4.9553	0.0003	0.0022	-0.2054
Mtfr1	-0.7923	6.5805	-4.9549	0.0003	0.0022	-0.2025
Gm15943	2.8441	-4.0074	4.9549	0.0003	0.0022	0.0654
Rpl36al	-0.6128	5.922	-4.954	0.0003	0.0022	-0.1757
Rnf103	0.6107	4.8658	4.9538	0.0003	0.0022	-0.0853
5830432E09Rik	-3.4733	-2.0431	-4.9533	0.0003	0.0022	0.0961

5930430L01Rik	0.8991	3.1378	4.953	0.0003	0.0022	0.1941
Baz1a	-0.6429	6.9699	-4.9527	0.0003	0.0022	-0.2191
Iqgap2	-2.0822	3.1868	-4.9523	0.0003	0.0022	0.3245
Gm45716	-2.3525	2.859	-4.9523	0.0003	0.0022	0.3869
Commd1	-0.8412	4.8313	-4.9519	0.0003	0.0022	-0.0531
Tulp4	0.5731	6.6062	4.9506	0.0003	0.0022	-0.2182
Socs1	-1.4188	3.1476	-4.9504	0.0003	0.0022	0.2746
Stat1	1.3339	3.7404	4.9495	0.0003	0.0022	0.0859
Ap1ar	-0.4314	6.2151	-4.9492	0.0003	0.0022	-0.2016
Msh5	-1.7077	1.0265	-4.9484	0.0003	0.0022	0.48
Pxmp4	1.0997	4.0064	4.9478	0.0003	0.0023	0.0377
Rac1	-0.46	8.8032	-4.9478	0.0003	0.0023	-0.2563
Cers2	-0.4604	7.8786	-4.9474	0.0003	0.0023	-0.2453
Angptl7	5.6635	-2.1675	4.9468	0.0003	0.0023	-0.1104
Ist1	0.4738	6.4338	4.9458	0.0003	0.0023	-0.2226
Plbd2	0.8152	6.6247	4.9452	0.0003	0.0023	-0.2287
Ncs1	-0.6336	6.083	-4.9445	0.0003	0.0023	-0.1964
Setd6	-1.0514	4.0658	-4.9421	0.0003	0.0023	0.0733
Acad10	1.3249	2.3181	4.9413	0.0003	0.0023	0.3106
Pigyl	0.8317	3.0088	4.9408	0.0003	0.0023	0.1808
Tcea1	-0.5563	6.7581	-4.9403	0.0003	0.0023	-0.2355
Edem2	0.6692	5.7584	4.9396	0.0003	0.0023	-0.1997
Rnf135	1.3247	2.7549	4.9375	0.0003	0.0023	0.228
Clic4	0.7849	8.0474	4.9371	0.0003	0.0023	-0.268
Ptgfrn	1.0188	7.1783	4.9366	0.0003	0.0023	-0.2543
Dyrk3	-1.7513	4.4251	-4.9364	0.0003	0.0023	0.063
Rassf8	-0.8559	6.1298	-4.9357	0.0003	0.0023	-0.2097
Rnf220	-0.5498	5.4758	-4.9343	0.0003	0.0023	-0.1714
Fam214b	1.3042	4.1086	4.9341	0.0003	0.0023	-0.0028
Eml1	-0.9068	6.1739	-4.9327	0.0004	0.0023	-0.218
Mllt11	-0.8408	4.1687	-4.9314	0.0004	0.0023	0.008
Abca8b	1.6986	2.6332	4.9285	0.0004	0.0023	0.2612
Birc2	1.4971	5.562	4.9282	0.0004	0.0023	-0.1863
Zfp563	1.0683	2.4187	4.9266	0.0004	0.0023	0.255
Ppm1g	-0.8133	7.7789	-4.9258	0.0004	0.0023	-0.28
Abcc1	-0.7296	7.1416	-4.9257	0.0004	0.0023	-0.2701
Ubl7	0.7441	5.6195	4.9244	0.0004	0.0023	-0.213
Rilpl1	1.0577	4.6837	4.9241	0.0004	0.0023	-0.1081
Ldlrad4	1.2709	2.8779	4.9235	0.0004	0.0023	0.2062
Krt14	2.7009	-3.3964	4.9223	0.0004	0.0023	0.1049
Wdr35	0.6863	5.2706	4.9221	0.0004	0.0023	-0.188
Zkscan14	1.0972	2.7853	4.9219	0.0004	0.0023	0.1941
Stambp	0.7636	4.7634	4.9192	0.0004	0.0023	-0.1374
Smarca2	0.9918	6.2577	4.9191	0.0004	0.0023	-0.2583
Hsd17b12	-0.8218	7.3432	-4.9176	0.0004	0.0023	-0.2873
Ppwd1	-0.7062	5.2525	-4.9145	0.0004	0.0023	-0.1688
Rad51b	-0.9919	2.1455	-4.9133	0.0004	0.0024	0.301
Sh3pxd2a	0.9409	7.5836	4.9125	0.0004	0.0024	-0.305
Psma1	-0.6749	7.3368	-4.9125	0.0004	0.0024	-0.2977
Adamts6	1.6888	2.1697	4.9106	0.0004	0.0024	0.2966
Uba3	-0.4062	7.2039	-4.9105	0.0004	0.0024	-0.2998
Gm43533	2.8136	-2.6091	4.9091	0.0004	0.0024	0.0116

Ywhag	-0.5336	8.6962	-4.9082	0.0004	0.0024	-0.3241
Isoc2b	2.0267	0.0073	4.9081	0.0004	0.0024	0.4422
Gm14636	-2.4843	-0.4013	-4.9061	0.0004	0.0024	0.3688
Usp19	0.4441	7.5088	4.9057	0.0004	0.0024	-0.3146
Trim34b	2.4457	-0.7669	4.9054	0.0004	0.0024	0.3998
Lyplal1	1.0698	3.9658	4.9052	0.0004	0.0024	-0.0214
Pdgfc	3.9924	4.1048	4.905	0.0004	0.0024	0.147
Bag2	-0.6851	5.9859	-4.9019	0.0004	0.0024	-0.2663
Ncbp2	-0.5308	6.3324	-4.9017	0.0004	0.0024	-0.2902
Six5	0.8493	3.1153	4.8994	0.0004	0.0024	0.097
Cops6	-0.4284	7.0845	-4.8988	0.0004	0.0024	-0.318
Nde1	-0.5971	6.2213	-4.8985	0.0004	0.0024	-0.2865
Uqcc3	0.9508	3.9609	4.8984	0.0004	0.0024	-0.0318
Rev3l	0.956	5.7669	4.8983	0.0004	0.0024	-0.2594
Plpp2	-0.5252	5.9022	-4.8982	0.0004	0.0024	-0.2695
Rnaseh2a	-0.7343	5.5025	-4.8976	0.0004	0.0024	-0.2322
Msantd3	-0.9836	4.2809	-4.8973	0.0004	0.0024	-0.055
Clcf1	-1.5223	4.6152	-4.8952	0.0004	0.0024	-0.0625
Frg2f1	1.7934	0.0761	4.8948	0.0004	0.0024	0.4204
Gpr107	0.4992	6.3068	4.8935	0.0004	0.0024	-0.3096
Cald1	1.1982	8.5018	4.8925	0.0004	0.0024	-0.3509
Lgr4	1.2598	4.5085	4.8915	0.0004	0.0024	-0.1237
Exoc4	0.5987	5.9428	4.8911	0.0004	0.0024	-0.295
Dnhd1	2.4575	0.1447	4.8899	0.0004	0.0024	0.4108
Utp3	-0.4653	6.601	-4.8896	0.0004	0.0024	-0.3218
Txnl1	-0.6284	7.7832	-4.8874	0.0004	0.0024	-0.3482
9230112E08Rik	1.1773	2.11	4.8872	0.0004	0.0024	0.2291
Tia1	0.467	6.1419	4.8867	0.0004	0.0024	-0.3088
Ptcd2	-0.651	5.4584	-4.8858	0.0004	0.0024	-0.2472
Piezo1	-0.5493	7.195	-4.8836	0.0004	0.0024	-0.3446
Dennd1a	0.555	4.6833	4.8824	0.0004	0.0024	-0.1873
Apex2	-0.7744	4.6048	-4.8818	0.0004	0.0024	-0.1389
Fkbp2	-0.52	5.1768	-4.8817	0.0004	0.0024	-0.2344
Utp15	-0.6796	6.4061	-4.8807	0.0004	0.0024	-0.3245
Plcl1	2.3374	0.9994	4.8804	0.0004	0.0024	0.3769
Vps45	0.5124	4.7161	4.8803	0.0004	0.0024	-0.2003
Nelfb	-0.4809	6.1219	-4.8797	0.0004	0.0025	-0.3208
Lrrc49	0.5727	4.3087	4.8767	0.0004	0.0025	-0.1518
Hnrnpr	-0.4427	6.8298	-4.8738	0.0004	0.0025	-0.3546
Mras	0.8781	5.6101	4.8734	0.0004	0.0025	-0.3037
Mospd1	0.9265	4.2222	4.8724	0.0004	0.0025	-0.1231
Phf5a	-0.874	5.8913	-4.8708	0.0004	0.0025	-0.306
Pea15a	-0.689	6.5418	-4.8689	0.0004	0.0025	-0.3534
Ncbp1	-0.5291	7.0767	-4.8669	0.0004	0.0025	-0.372
Nadk2	-0.8391	5.3924	-4.8668	0.0004	0.0025	-0.2633
Gm5620	-1.6032	0.5473	-4.8651	0.0004	0.0025	0.3736
Psmd11	-0.6113	7.7161	-4.8645	0.0004	0.0025	-0.3878
Spata24	-1.5148	1.2179	-4.8623	0.0004	0.0025	0.3245
Tgfa	-1.8935	3.6581	-4.8617	0.0004	0.0025	0.0734
3110082I17Rik	-1.0234	4.2017	-4.8617	0.0004	0.0025	-0.1019
mt-Rnr1	0.9231	9.3947	4.8602	0.0004	0.0025	-0.4144
Rnf26	-0.7605	2.9213	-4.8599	0.0004	0.0025	0.0957

Dusp7	-0.7069	6.3806	-4.856	0.0004	0.0025	-0.3659
Babam2	0.6005	5.6674	4.8544	0.0004	0.0025	-0.3406
Nle1	-0.9179	4.6644	-4.8543	0.0004	0.0025	-0.185
Tk2	0.6906	3.9493	4.851	0.0004	0.0026	-0.1319
Fam98b	-0.5796	5.5954	-4.8473	0.0004	0.0026	-0.3339
Plscr1	-0.8945	6.7796	-4.8465	0.0004	0.0026	-0.3969
Sec61a2	-0.6318	5.2365	-4.8463	0.0004	0.0026	-0.2934
Gm16861	1.5159	-0.3546	4.8445	0.0004	0.0026	0.3423
Rpap3	-0.9128	5.8489	-4.8444	0.0004	0.0026	-0.3493
Zfp106	-0.5362	8.5792	-4.8438	0.0004	0.0026	-0.4353
Hsd17b11	0.7558	3.7	4.8429	0.0004	0.0026	-0.1046
Endov	0.5712	4.0456	4.8424	0.0004	0.0026	-0.1594
Usp14	-0.7874	7.3761	-4.8398	0.0004	0.0026	-0.4235
Gabarapl1	0.9052	7.0011	4.8394	0.0004	0.0026	-0.4234
Phldb1	0.8585	6.8943	4.8379	0.0004	0.0026	-0.4237
Unc45a	0.6192	5.4038	4.8377	0.0004	0.0026	-0.3488
Gm26735	1.8335	1.9303	4.8371	0.0004	0.0026	0.1711
Mcts1	-0.6594	5.361	-4.8366	0.0004	0.0026	-0.3268
Sdhb	-0.5872	6.8589	-4.8364	0.0004	0.0026	-0.4223
Nf2	0.5977	7.6101	4.8358	0.0004	0.0026	-0.4391
Gpatch4	-0.8155	5.4551	-4.8325	0.0004	0.0026	-0.3341
Rfc1	-0.5587	7.313	-4.8323	0.0004	0.0026	-0.437
1700066M21Rik	-1.0977	4.0838	-4.8319	0.0004	0.0026	-0.1109
4930442G10Rik	-2.8136	-2.1978	-4.8306	0.0004	0.0026	-0.0381
Gm9531	-1.4669	1.5654	-4.8274	0.0004	0.0026	0.2468
Cables1	1.9979	1.0243	4.8273	0.0004	0.0026	0.2635
Pigs	0.4999	6.0758	4.8271	0.0004	0.0026	-0.415
Coro1a	2.4617	-0.6214	4.8257	0.0004	0.0026	0.2876
Ifitm10	1.3214	-0.3242	4.8255	0.0004	0.0026	0.3121
Foxd1	1.4841	3.7304	4.8253	0.0004	0.0026	-0.089
Mospd3	0.9454	3.805	4.8253	0.0004	0.0026	-0.137
Pxdc1	-1.1268	5.061	-4.8251	0.0004	0.0026	-0.2895
4632415L05Rik	0.9273	5.1402	4.8215	0.0004	0.0027	-0.3366
Ctbp2	-0.4526	6.1097	-4.8212	0.0004	0.0027	-0.42
Twist2	-0.8264	4.7572	-4.8196	0.0004	0.0027	-0.2623
Ubtd2	0.7157	4.2097	4.8191	0.0004	0.0027	-0.2099
Gm10638	4.9572	-1.3229	4.8188	0.0004	0.0027	0.0723
Cog4	0.9585	6.4456	4.8186	0.0004	0.0027	-0.4447
Gm5300	3.7223	-0.5629	4.8181	0.0004	0.0027	0.2154
Snhg17	-0.9258	4.655	-4.818	0.0004	0.0027	-0.2502
Slc39a11	0.5276	4.8801	4.8178	0.0004	0.0027	-0.3284
Tyro3	-0.547	4.3432	-4.8177	0.0004	0.0027	-0.2192
Rprd1b	-0.6905	5.6733	-4.8172	0.0004	0.0027	-0.3898
Slc41a3	2.1629	0.3551	4.8164	0.0004	0.0027	0.291
Abtb2	0.6945	4.0606	4.8155	0.0004	0.0027	-0.2068
Zdhhc3	-0.4411	6.759	-4.8149	0.0004	0.0027	-0.4574
Fancf	-1.0185	2.4921	-4.8146	0.0004	0.0027	0.0879
Itpr3	-0.8272	7.6255	-4.8141	0.0004	0.0027	-0.4734
S100a1	1.2439	4.8469	4.8139	0.0004	0.0027	-0.3131
Ifit2	1.5745	4.5764	4.8137	0.0004	0.0027	-0.2619
Mecr	-0.4844	4.6332	-4.8123	0.0004	0.0027	-0.2924
Znhit3	-0.6644	4.1836	-4.8122	0.0004	0.0027	-0.2071

Arl6ip6	-0.731	4.947	-4.8119	0.0004	0.0027	-0.312
Ptpra	0.6046	7.105	4.8106	0.0004	0.0027	-0.4753
Nlrx1	1.4674	0.9583	4.8075	0.0004	0.0027	0.2279
Iqsec2	1.1459	4.1283	4.8068	0.0004	0.0027	-0.2139
Pcyox1	0.5125	7.2787	4.8066	0.0004	0.0027	-0.4847
Fbxo48	-2.2696	0.4497	-4.8058	0.0004	0.0027	0.28
Slc9a9	2.0327	-0.2673	4.8048	0.0004	0.0027	0.2806
Zfp862-ps	0.9167	4.0381	4.8041	0.0004	0.0027	-0.2096
Smc6	-0.788	7.5562	-4.8034	0.0004	0.0027	-0.491
Pds5b	-0.595	7.0908	-4.8032	0.0004	0.0027	-0.4826
Tcaf1	1.1657	5.8101	4.8029	0.0004	0.0027	-0.436
Vcan	1.4255	8.4002	4.8012	0.0004	0.0027	-0.5092
Ubxn2b	0.789	3.3669	4.8006	0.0004	0.0027	-0.0989
Zfp553	0.823	5.0901	4.7997	0.0004	0.0027	-0.3794
Sh3d19	1.7318	6.2246	4.7993	0.0004	0.0027	-0.4551
Mob1a	-0.4076	7.8371	-4.7983	0.0004	0.0027	-0.5059
Tmem86a	2.1631	3.2864	4.7981	0.0004	0.0027	-0.0304
Rassf2	2.4667	1.6233	4.798	0.0004	0.0027	0.1862
Med4	-0.5349	5.245	-4.7973	0.0004	0.0027	-0.3848
Sema7a	-3.5146	4.6936	-4.7964	0.0004	0.0027	-0.0043
Slc25a44	0.5945	5.6867	4.7954	0.0004	0.0027	-0.4425
Fbxl14	-0.6588	5.5852	-4.795	0.0004	0.0027	-0.4199
Trmt10c	-0.7356	5.07	-4.7938	0.0004	0.0027	-0.3576
Zfp326	-0.4765	5.5797	-4.7936	0.0004	0.0027	-0.4258
Anxa4	0.9618	7.9466	4.7935	0.0004	0.0027	-0.5187
Rfxank	0.6627	3.9797	4.7932	0.0004	0.0027	-0.2454
Wdfy3	0.8272	6.6787	4.7932	0.0004	0.0027	-0.4935
Stn1	-0.6001	5.4161	-4.7904	0.0004	0.0028	-0.4153
Gm16845	1.617	0.5406	4.7896	0.0004	0.0028	0.2346
Rars	-0.5174	7.9579	-4.7894	0.0004	0.0028	-0.5234
Phc2	-0.6389	7.6018	-4.7883	0.0004	0.0028	-0.5197
Coq2	-0.9401	4.2834	-4.7878	0.0004	0.0028	-0.2536
Bloc1s2	-0.5145	5.0767	-4.787	0.0004	0.0028	-0.3901
Ccdc28a	0.8752	2.7429	4.7869	0.0004	0.0028	-0.05
Zfp93	1.002	2.8102	4.7857	0.0004	0.0028	-0.0455
Tpm3-rs7	-0.8519	2.0017	-4.7826	0.0005	0.0028	0.079
Cdc123	-0.4449	6.7226	-4.7822	0.0005	0.0028	-0.5134
Ltbp1	-0.903	7.0799	-4.7814	0.0005	0.0028	-0.5196
Hyls1	-0.7792	4.0707	-4.7811	0.0005	0.0028	-0.2423
Chst12	0.706	4.6831	4.7807	0.0005	0.0028	-0.3657
Zfp280b	-0.5275	4.3999	-4.7794	0.0005	0.0028	-0.2993
Pyroxd2	3.4822	0.9936	4.7785	0.0005	0.0028	0.2382
Pspc1	-0.71	5.401	-4.778	0.0005	0.0028	-0.4285
Rnf215	0.6927	3.9887	4.7767	0.0005	0.0028	-0.2541
Jag2	-1.4751	4.9755	-4.7765	0.0005	0.0028	-0.3262
AI506816	-1.5229	2.2929	-4.7763	0.0005	0.0028	0.0977
Zc3h14	-0.468	7.5561	-4.776	0.0005	0.0028	-0.541
Nol7	-0.7225	6.4091	-4.7757	0.0005	0.0028	-0.5115
Vwa8	1.0099	5.8441	4.7756	0.0005	0.0028	-0.4859
Cdc14a	-0.6868	4.2208	-4.7745	0.0005	0.0028	-0.2814
Ift172	0.8833	4.8607	4.7744	0.0005	0.0028	-0.3958
Riok1	-0.5095	5.4632	-4.7722	0.0005	0.0028	-0.4506

Gchfr	-1.1514	5.0089	-4.772	0.0005	0.0028	-0.3646
Fbxw4	0.8023	3.6413	4.7672	0.0005	0.0028	-0.2289
Sarnp	-0.4347	6.1172	-4.7671	0.0005	0.0028	-0.5178
Gm13778	2.5718	-2.5913	4.7666	0.0005	0.0028	-0.0229
Pcmtd2	0.8651	5.2158	4.7653	0.0005	0.0028	-0.4442
Slc27a1	0.8682	5.385	4.7649	0.0005	0.0028	-0.4721
Bcl3	0.9369	4.9419	4.7648	0.0005	0.0028	-0.4165
Pak4	-0.6699	5.2778	-4.7648	0.0005	0.0028	-0.4432
Gm3320	-2.9603	-1.0218	-4.7645	0.0005	0.0028	0.0353
Kdsr	0.4744	5.4817	4.7639	0.0005	0.0028	-0.4797
Plod2	-1.2884	7.3236	-4.7639	0.0005	0.0028	-0.5537
B930095G15Rik	1.1961	0.9978	4.7636	0.0005	0.0028	0.1546
Rad50	-0.5253	6.1942	-4.7627	0.0005	0.0028	-0.5242
Lbp	3.6412	4.5436	4.7615	0.0005	0.0029	-0.1753
Ppil2	0.4754	5.9958	4.7613	0.0005	0.0029	-0.5279
Ecd	-0.4587	6.3999	-4.7602	0.0005	0.0029	-0.5409
Dnaja3	-0.5164	6.1284	-4.7576	0.0005	0.0029	-0.5333
Ripor1	0.6646	5.7087	4.7566	0.0005	0.0029	-0.5156
Nhlrc3	0.6376	4.8652	4.7566	0.0005	0.0029	-0.43
Traf6	0.7089	5.1195	4.7565	0.0005	0.0029	-0.4526
U2af2	-0.5334	7.9323	-4.7557	0.0005	0.0029	-0.5821
Xrcc3	-1.0547	2.3931	-4.7556	0.0005	0.0029	0.0022
Cadm1	-1.1298	6.8947	-4.7553	0.0005	0.0029	-0.5546
Gm13577	-1.2436	1.6946	-4.7538	0.0005	0.0029	0.1006
Vps37c	0.6297	4.9464	4.7534	0.0005	0.0029	-0.4464
Mfsd6	1.3196	4.4477	4.7506	0.0005	0.0029	-0.3562
Nudcd1	-0.6921	6.1797	-4.7502	0.0005	0.0029	-0.5421
Vps50	0.4899	6.4958	4.7499	0.0005	0.0029	-0.5657
Cep135	-0.8121	3.9746	-4.7459	0.0005	0.0029	-0.2707
Enc1	1.1625	5.3672	4.7453	0.0005	0.0029	-0.4954
Rab36	1.8174	0.242	4.7447	0.0005	0.0029	0.1812
Mme	5.2808	3.5312	4.7446	0.0005	0.0029	0.0988
Btg3	-0.9764	3.5617	-4.7437	0.0005	0.0029	-0.2107
Gnb5	0.7033	4.1236	4.7413	0.0005	0.0029	-0.3409
Gm9968	2.6913	-2.893	4.7399	0.0005	0.0029	-0.1037
2310043P16Rik	3.6889	-1.5321	4.7382	0.0005	0.0029	-0.0687
Nop14	-0.6979	6.9522	-4.7375	0.0005	0.0029	-0.5965
Inpp4a	0.6958	4.0193	4.7366	0.0005	0.003	-0.3345
Timm23	-0.683	5.5373	-4.7366	0.0005	0.003	-0.514
Pitpna	-0.5541	7.7421	-4.7359	0.0005	0.003	-0.6142
Tmem126b	0.6608	3.7312	4.7358	0.0005	0.003	-0.2959
Apobec3	1.1704	5.0262	4.735	0.0005	0.003	-0.4853
Gm8615	1.3287	2.4569	4.7334	0.0005	0.003	-0.072
Gfm1	-0.5418	6.9853	-4.7315	0.0005	0.003	-0.6088
Vasp	-0.4756	7.2714	-4.729	0.0005	0.003	-0.6195
Prpf3	-0.5771	6.6569	-4.7283	0.0005	0.003	-0.6052
2700081O15Rik	0.9349	5.5967	4.7282	0.0005	0.003	-0.5483
Gm17491	-1.4297	2.0183	-4.7282	0.0005	0.003	0.0333
Gm26606	1.5447	-0.615	4.7279	0.0005	0.003	0.1591
Rbm28	-0.5373	6.5647	-4.7262	0.0005	0.003	-0.6054
Sltm	-0.6529	6.9757	-4.7261	0.0005	0.003	-0.617
Spg21	0.5871	6.2959	4.7247	0.0005	0.003	-0.6042

Atp1b1	3.561	1.209	4.7247	0.0005	0.003	0.1402
Pus1	-0.7107	5.9137	-4.724	0.0005	0.003	-0.5697
Zc3h15	-0.6525	7.1028	-4.7234	0.0005	0.003	-0.6247
Cdk2ap1	-0.5682	5.4086	-4.7232	0.0005	0.003	-0.5336
Gm19557	-2.8379	-0.564	-4.722	0.0005	0.003	0.0756
Gjb4	1.5968	3.777	4.7219	0.0005	0.003	-0.278
Mrpl22	-0.5496	5.36	-4.7192	0.0005	0.003	-0.5394
Gm22748	-3.3355	-1.0037	-4.7191	0.0005	0.003	-0.0369
Gm29292	4.7181	-1.5007	4.7188	0.0005	0.003	-0.1447
Gpatch2l	0.5786	4.6755	4.7186	0.0005	0.003	-0.4676
Casp8ap2	-0.691	5.8188	-4.716	0.0005	0.003	-0.574
Mxd1	0.8663	4.6567	4.7149	0.0005	0.003	-0.4639
Ccng1	0.6514	6.5965	4.7147	0.0005	0.003	-0.6319
Exosc1	-0.907	5.1678	-4.7143	0.0005	0.003	-0.5006
Gm6055	1.2583	0.5419	4.713	0.0005	0.003	0.1017
Simc1	-0.6471	4.6703	-4.7119	0.0005	0.003	-0.4493
Sephs1	-0.5011	6.5041	-4.7094	0.0005	0.0031	-0.6322
Krt76	-3.5773	4.4918	-4.7083	0.0005	0.0031	-0.0935
Mapre3	0.9998	3.7891	4.7081	0.0005	0.0031	-0.3531
Pcdhb22	1.9099	1.4513	4.708	0.0005	0.0031	0.0354
Smarcd3	1.1626	3.5933	4.7075	0.0005	0.0031	-0.2993
Pigc	0.5748	4.2858	4.7051	0.0005	0.0031	-0.4406
Pxn	-0.4616	7.4894	-4.7049	0.0005	0.0031	-0.6649
Zfp746	0.4526	4.9508	4.7044	0.0005	0.0031	-0.5319
Dusp9	-1.714	6.0482	-4.7018	0.0005	0.0031	-0.5796
Phka2	0.8304	5.2425	4.7008	0.0005	0.0031	-0.567
Fam160a1	2.1195	3.6784	4.7007	0.0005	0.0031	-0.2699
Naa80	0.9966	3.2532	4.7004	0.0005	0.0031	-0.2811
Invs	0.9518	3.7024	4.7001	0.0005	0.0031	-0.3464
Eif3i	-0.5531	7.451	-4.6985	0.0005	0.0031	-0.676
Sugct	2.2067	-0.2304	4.6982	0.0005	0.0031	0.1128
Lonp1	-0.4897	7.6706	-4.6966	0.0005	0.0031	-0.6839
Scp2	0.8029	5.7389	4.6942	0.0005	0.0031	-0.626
Tent5b	3.4549	2.9773	4.6941	0.0005	0.0031	-0.0484
Rbms2	0.8654	5.6641	4.6938	0.0005	0.0031	-0.6224
Dnajb9	0.7141	4.8527	4.6938	0.0005	0.0031	-0.5263
Utp6	-0.6669	6.8066	-4.6922	0.0005	0.0031	-0.6715
Kif3a	0.4287	5.7496	4.6914	0.0005	0.0031	-0.6348
Zranb2	-0.6866	7.1083	-4.6909	0.0005	0.0031	-0.6812
Spata2l	1.55	1.1052	4.69	0.0005	0.0031	0.018
Mrpl3	-0.3984	6.2006	-4.6899	0.0005	0.0031	-0.6586
Frs2	0.5539	5.5476	4.688	0.0005	0.0031	-0.6166
Nrep	7.2783	2.8427	4.6874	0.0005	0.0031	0.0959
Lhfp	1.0741	5.8532	4.6873	0.0005	0.0031	-0.6456
Echs1	-0.7465	6.24	-4.6873	0.0005	0.0031	-0.6607
Ccp110	-0.749	4.8454	-4.6859	0.0005	0.0032	-0.5121
Plekhh2	1.0041	5.303	4.6858	0.0005	0.0032	-0.5929
Ppp1r8	-0.6017	6.2981	-4.6849	0.0005	0.0032	-0.6677
Gpr75	4.5539	-2.426	4.6845	0.0005	0.0032	-0.2524
Larp4	-0.715	7.5332	-4.6842	0.0005	0.0032	-0.7007
Plppr3	2.9307	2.8873	4.6832	0.0005	0.0032	-0.1278
Parl	-0.5852	5.4461	-4.6812	0.0005	0.0032	-0.6128

Serpinb8	2.8657	-0.775	4.6809	0.0005	0.0032	0.0608
Zbtb11os1	1.7457	0.903	4.6786	0.0005	0.0032	0.0311
Grpel1	-0.4894	6.4146	-4.6783	0.0005	0.0032	-0.6889
Fcgr4	4.0644	-0.996	4.6769	0.0005	0.0032	-0.0174
Egr3	-4.4559	2.3749	-4.6758	0.0005	0.0032	0.0713
Mrps17	-0.4281	5.4353	-4.6757	0.0005	0.0032	-0.6281
Calu	-0.4826	9.1896	-4.6742	0.0005	0.0032	-0.7404
Zfp369	0.636	4.242	4.6729	0.0005	0.0032	-0.4849
Ccdc171	0.9684	1.8875	4.6728	0.0005	0.0032	-0.1135
Srsf10	-0.6833	7.0018	-4.6725	0.0005	0.0032	-0.7103
Fermt3	-1.6765	0.6019	-4.6725	0.0005	0.0032	0.0546
Pabpc4	-0.6763	7.2098	-4.6725	0.0005	0.0032	-0.7171
Proser2	-1.2771	3.581	-4.6689	0.0005	0.0032	-0.3103
Nr1d1	-0.8646	4.4393	-4.6667	0.0006	0.0032	-0.4967
Lrrc8c	-0.7104	5.6638	-4.6667	0.0006	0.0032	-0.6485
Dnal1	0.5529	3.8541	4.6662	0.0006	0.0032	-0.4433
Orai3	0.5541	4.4761	4.6649	0.0006	0.0032	-0.5402
Fbxl15	0.6349	3.352	4.6642	0.0006	0.0032	-0.3598
Slc35a3	-0.5214	5.8912	-4.6594	0.0006	0.0033	-0.6868
Mrpl52	-0.7681	5.124	-4.6592	0.0006	0.0033	-0.6086
Rhof	1.9112	0.5462	4.6575	0.0006	0.0033	0.0247
Ppm1j	-1.708	-0.9478	-4.6574	0.0006	0.0033	0.0246
Marveld1	0.4719	5.2558	4.6567	0.0006	0.0033	-0.6501
Zfp850	-1.0752	3.5813	-4.6558	0.0006	0.0033	-0.3324
R3hdm4	0.5641	5.4431	4.6549	0.0006	0.0033	-0.6741
Prkd2	0.7436	3.7856	4.6528	0.0006	0.0033	-0.4487
Gm18736	-1.104	2.2149	-4.6508	0.0006	0.0033	-0.1605
Rsph3b	0.9802	2.2183	4.6498	0.0006	0.0033	-0.1895
Gtf2h3	-0.5228	5.0797	-4.6497	0.0006	0.0033	-0.6352
Hscb	1.0145	3.4482	4.6494	0.0006	0.0033	-0.3865
Zfp386	0.7804	4.9792	4.6485	0.0006	0.0033	-0.6244
Sorbs3	1.7428	4.4667	4.6481	0.0006	0.0033	-0.5127
Wdr46	-0.7185	6.2523	-4.6479	0.0006	0.0033	-0.7289
D430020J02Rik	1.149	2.7783	4.6447	0.0006	0.0033	-0.255
Uba7	3.9853	0.6125	4.6437	0.0006	0.0033	0.022
Trim24	0.9544	5.8573	4.6435	0.0006	0.0033	-0.718
Smn1	-0.6173	5.9963	-4.6421	0.0006	0.0033	-0.725
Pcdhb19	2.3212	-0.6736	4.6419	0.0006	0.0033	0.0133
Loxl3	-1.1877	4.5894	-4.6412	0.0006	0.0034	-0.5417
Rela	0.4903	6.5987	4.6404	0.0006	0.0034	-0.7645
Rbm12	-0.5944	4.7438	-4.6402	0.0006	0.0034	-0.5867
Gm48488	1.3523	0.0993	4.6392	0.0006	0.0034	0.0099
Ncaph2	-0.8038	7.7938	-4.6387	0.0006	0.0034	-0.7868
Grk4	1.9537	0.5322	4.6382	0.0006	0.0034	-0.0068
Ccdc88b	2.269	-1.5143	4.6374	0.0006	0.0034	-0.0566
Brix1	-0.8773	6.6138	-4.6367	0.0006	0.0034	-0.7602
Ifi30	1.171	5.8808	4.6351	0.0006	0.0034	-0.741
Cdk6	-0.7825	7.6811	-4.6348	0.0006	0.0034	-0.7905
Pprc1	-0.8607	6.6054	-4.6339	0.0006	0.0034	-0.7645
Noc4l	-0.7568	5.9092	-4.6337	0.0006	0.0034	-0.7272
Dctn4	0.4653	6.9036	4.6336	0.0006	0.0034	-0.7837
Mtx3	-0.5074	4.5513	-4.6321	0.0006	0.0034	-0.5779

Prpf19	-0.5601	7.8194	-4.632	0.0006	0.0034	-0.7996
Imp3	-0.5498	5.5908	-4.6318	0.0006	0.0034	-0.7176
Prelid1	-0.4995	8.451	-4.6317	0.0006	0.0034	-0.8092
Otud6b	-0.5525	6.0587	-4.6316	0.0006	0.0034	-0.746
Khsrp	-0.4285	7.9501	-4.6295	0.0006	0.0034	-0.8058
Sall2	1.0883	2.7316	4.6276	0.0006	0.0034	-0.3
Tars2	-0.6727	5.7066	-4.6273	0.0006	0.0034	-0.7292
Lrrc15	2.7335	-2.4671	4.6272	0.0006	0.0034	-0.2123
Slc30a4	-0.6196	8.3017	-4.6249	0.0006	0.0034	-0.8187
Zfyve27	0.6475	5.0004	4.6245	0.0006	0.0034	-0.6826
Bst2	1.3909	4.9165	4.6233	0.0006	0.0034	-0.6566
Snhg6	-0.6795	4.1873	-4.6232	0.0006	0.0034	-0.5349
Gm6069	3.4172	-3.5004	4.6214	0.0006	0.0034	-0.3629
Vamp5	1.9198	3.2866	4.62	0.0006	0.0034	-0.3965
Klf9	0.9158	6.9892	4.6198	0.0006	0.0034	-0.8091
Adat1	-0.8231	3.6033	-4.6194	0.0006	0.0034	-0.4363
Gm6505	-0.9624	2.2675	-4.619	0.0006	0.0034	-0.2068
Grb14	2.2135	-0.138	4.6184	0.0006	0.0035	-0.013
Rpl7a	-0.5065	9.9117	-4.6176	0.0006	0.0035	-0.8452
Rad23a	-0.702	6.2149	-4.6164	0.0006	0.0035	-0.7849
Ssbp1	-0.5175	5.7023	-4.6158	0.0006	0.0035	-0.7557
Tbc1d4	1.0504	6.0455	4.6155	0.0006	0.0035	-0.7803
Idh1	1.0279	8.0007	4.6154	0.0006	0.0035	-0.8347
Serpinb9b	2.1675	0.0351	4.6152	0.0006	0.0035	-0.0274
Fam57a	-0.5549	4.4209	-4.6115	0.0006	0.0035	-0.5996
Ttc39c	1.0481	5.6839	4.6105	0.0006	0.0035	-0.7663
Cenpt	-0.7599	4.6138	-4.61	0.0006	0.0035	-0.6238
Sh3bp5l	0.523	5.3721	4.609	0.0006	0.0035	-0.7456
Ctps2	0.4636	6.3512	4.6088	0.0006	0.0035	-0.8113
Siah2	-0.9479	5.1209	-4.6069	0.0006	0.0035	-0.6906
Trip6	0.627	5.6307	4.6069	0.0006	0.0035	-0.7753
Plau	-2.0231	5.8624	-4.6067	0.0006	0.0035	-0.7164
Ccnc	-0.8054	4.2763	-4.606	0.0006	0.0035	-0.5598
Gnl1	-0.4903	6.5757	-4.6056	0.0006	0.0035	-0.821
Gm6969	0.5779	4.6508	4.6048	0.0006	0.0035	-0.6711
Uros	0.8564	2.8078	4.6036	0.0006	0.0035	-0.3641
Nab1	-0.5763	7.3339	-4.6033	0.0006	0.0035	-0.8415
Eci1	0.51	5.2436	4.6029	0.0006	0.0035	-0.7465
Gm49338	0.9368	3.2051	4.6019	0.0006	0.0035	-0.4158
Aarsd1	-1.1448	2.1452	-4.6012	0.0006	0.0035	-0.2036
Denr	-0.6272	6.7168	-4.601	0.0006	0.0035	-0.8325
Gm48529	1.2281	0.4776	4.5989	0.0006	0.0035	-0.075
Mettl7a1	1.6637	2.2586	4.5988	0.0006	0.0035	-0.2615
Lrrc58	0.6449	6.9277	4.5984	0.0006	0.0035	-0.8444
Usp15	-0.5296	6.4629	-4.5975	0.0006	0.0035	-0.8318
Urgcp	-0.606	6.776	-4.5975	0.0006	0.0035	-0.8416
Repin1	0.6489	4.6073	4.5971	0.0006	0.0035	-0.6753
Ubb	0.4971	8.1696	4.5932	0.0006	0.0036	-0.8765
Mpp7	1.239	4.2702	4.5924	0.0006	0.0036	-0.6081
Slc23a3	2.9648	-2.7039	4.5917	0.0006	0.0036	-0.3845
Manea	0.9441	4.3031	4.5897	0.0006	0.0036	-0.6234
Snrnp25	-0.6239	4.34	-4.5895	0.0006	0.0036	-0.6363

Samd5	-1.2874	2.9218	-4.5895	0.0006	0.0036	-0.3297
Pigw	-0.8321	3.6246	-4.5894	0.0006	0.0036	-0.4905
Ibtk	-0.5635	6.1425	-4.5886	0.0006	0.0036	-0.8312
Zfp12	0.5588	4.5678	4.5859	0.0006	0.0036	-0.6903
Ints7	-0.4864	6.5499	-4.5852	0.0006	0.0036	-0.8565
Prss23	4.555	3.299	4.585	0.0006	0.0036	-0.2365
Cblb	0.822	6.2141	4.5843	0.0006	0.0036	-0.8457
Mipol1	-0.929	3.4298	-4.5837	0.0006	0.0036	-0.4512
Rnf183	-2.4426	1.9725	-4.5832	0.0006	0.0036	-0.1635
Tada2a	-0.7016	5.111	-4.5821	0.0006	0.0036	-0.7451
Tmem138	-0.6619	4.0558	-4.5809	0.0006	0.0036	-0.59
Glt28d2	1.975	0.4334	4.58	0.0006	0.0036	-0.1037
Tpra1	0.833	4.7971	4.5797	0.0006	0.0036	-0.7377
Chmp1a	0.5152	6.5428	4.5797	0.0006	0.0036	-0.8706
Zfp263	0.6509	5.1067	4.5794	0.0006	0.0036	-0.7581
Usp2	2.0311	0.7924	4.5794	0.0006	0.0036	-0.112
Gm37233	1.8853	1.2726	4.5791	0.0006	0.0036	-0.1583
Mcrip1	0.459	5.5329	4.579	0.0006	0.0036	-0.8182
Atp23	-0.8823	3.3904	-4.5764	0.0006	0.0036	-0.4645
4930546K05Rik	-2.8451	-1.7644	-4.576	0.0006	0.0036	-0.2408
Mier2	-0.6807	4.5909	-4.5744	0.0006	0.0037	-0.6845
Ajm1	2.1469	0.7769	4.5736	0.0006	0.0037	-0.1306
Nol12	-0.7783	5.226	-4.573	0.0006	0.0037	-0.7722
Dcbld1	-0.8171	3.498	-4.5723	0.0006	0.0037	-0.5052
Cmtm3	1.4635	5.8443	4.5716	0.0006	0.0037	-0.8429
Fchsd1	1.6121	3.234	4.571	0.0006	0.0037	-0.4753
Dclre1b	-0.4923	4.53	-4.5707	0.0006	0.0037	-0.6924
Pik3c3	0.7185	5.5814	4.5702	0.0006	0.0037	-0.8329
Wdr45	0.9124	5.4692	4.5684	0.0007	0.0037	-0.8269
Plpp6	1.5089	2.1898	4.5678	0.0007	0.0037	-0.3241
Tchp	0.787	3.342	4.5676	0.0007	0.0037	-0.5334
Pip4k2a	0.6694	5.1174	4.5674	0.0007	0.0037	-0.7918
Gnl2	-0.4994	6.4457	-4.5673	0.0007	0.0037	-0.8839
Txndc15	0.4984	6.2579	4.5664	0.0007	0.0037	-0.8825
Wnt10b	2.0834	2.7995	4.5661	0.0007	0.0037	-0.3555
Tns4	-2.1576	2.9459	-4.5659	0.0007	0.0037	-0.298
Lrrc61	1.5657	2.6322	4.5657	0.0007	0.0037	-0.3764
Hhipl1	-1.6449	5.5711	-4.5652	0.0007	0.0037	-0.7616
Grsf1	-0.4625	7.3035	-4.5651	0.0007	0.0037	-0.9107
Zfp229	0.8368	2.7077	4.5644	0.0007	0.0037	-0.4152
Numa1	0.8594	7.596	4.5628	0.0007	0.0037	-0.9224
Gm48804	3.0697	-3.7001	4.5615	0.0007	0.0037	-0.4227
B4galt4	1.4998	1.6862	4.5604	0.0007	0.0037	-0.2629
4933412E12Rik	1.2238	2.7591	4.5601	0.0007	0.0037	-0.4096
Vdac2	-0.3965	8.2888	-4.5599	0.0007	0.0037	-0.9353
Surf6	-0.5147	5.1763	-4.5571	0.0007	0.0037	-0.8028
Sestd1	-0.5009	7.023	-4.5563	0.0007	0.0037	-0.9216
Proser1	0.6542	5.6311	4.5547	0.0007	0.0037	-0.864
Rasa1	-0.4702	6.9931	-4.5542	0.0007	0.0037	-0.9232
Gdpd1	1.2544	4.8473	4.5537	0.0007	0.0038	-0.7644
Atoh8	4.4273	-0.1566	4.5537	0.0007	0.0038	-0.1948
Pcgf2	0.4241	5.1856	4.5507	0.0007	0.0038	-0.8295

Jup	1.7603	5.4766	4.5503	0.0007	0.0038	-0.8269
Foxp1	0.9929	7.0185	4.5501	0.0007	0.0038	-0.9348
Lrrc56	1.4666	0.8384	4.5497	0.0007	0.0038	-0.1902
Celf4	-1.9597	2.3429	-4.549	0.0007	0.0038	-0.2733
Nek9	0.5548	7.0239	4.5479	0.0007	0.0038	-0.9388
Xpo6	-0.4864	6.8202	-4.5478	0.0007	0.0038	-0.9301
Shpk	2.4622	1.6746	4.5465	0.0007	0.0038	-0.2343
Itm2c	0.6336	7.2338	4.5454	0.0007	0.0038	-0.9477
Rc3h1	0.4575	6.3139	4.5452	0.0007	0.0038	-0.9203
Ccdc141	1.1862	1.2066	4.5451	0.0007	0.0038	-0.2379
Mrpl19	-0.5835	5.365	-4.5447	0.0007	0.0038	-0.848
Sec61a1	-0.4026	8.6103	-4.5435	0.0007	0.0038	-0.9682
Apc2	3.2127	-1.5484	4.5434	0.0007	0.0038	-0.2512
Gm37305	0.9646	1.831	4.5425	0.0007	0.0038	-0.3278
Hnrnpl	-0.3813	8.6826	-4.5424	0.0007	0.0038	-0.9709
Mrpl47	-0.7273	4.9821	-4.542	0.0007	0.0038	-0.8031
Hdac5	0.8022	6.7849	4.5417	0.0007	0.0038	-0.9467
Cant1	0.4393	6.2833	4.5416	0.0007	0.0038	-0.9299
Parp16	-0.804	4.4578	-4.5416	0.0007	0.0038	-0.7258
Sssca1	-1.1491	1.9312	-4.5413	0.0007	0.0038	-0.2963
Usp12	-0.5923	6.1912	-4.5411	0.0007	0.0038	-0.9153
Car13	1.2285	5.6757	4.5392	0.0007	0.0038	-0.8861
Tspan11	0.6734	6.2645	4.5391	0.0007	0.0038	-0.9317
Gm28229	-1.6563	0.209	-4.5377	0.0007	0.0038	-0.1414
Rps2	-0.4842	9.7428	-4.5373	0.0007	0.0038	-0.9878
Rhbdl3	3.7427	-1.4789	4.5367	0.0007	0.0038	-0.3106
Gng7	1.7348	0.4936	4.5365	0.0007	0.0038	-0.1627
Thap2	0.663	5.0472	4.5342	0.0007	0.0038	-0.839
Pag1	1.98	1.2037	4.5332	0.0007	0.0038	-0.2204
A430005L14Rik	-0.7179	4.6005	-4.5309	0.0007	0.0039	-0.7637
Arglu1	-0.666	6.2409	-4.5294	0.0007	0.0039	-0.9366
Slc35e1	-0.6094	6.4808	-4.5289	0.0007	0.0039	-0.9522
Sf3b5	-0.6481	5.178	-4.5289	0.0007	0.0039	-0.8523
Mfge8	0.5751	8.2467	4.5278	0.0007	0.0039	-0.9943
Cnbd2	0.9741	2.2573	4.5273	0.0007	0.0039	-0.3995
Cdk5rap3	0.4965	5.7655	4.5271	0.0007	0.0039	-0.9273
Psmb10	1.2911	5.4349	4.5259	0.0007	0.0039	-0.8896
Stxbp3	0.6372	6.1705	4.5259	0.0007	0.0039	-0.9499
Bcl7c	-0.5754	5.1142	-4.5256	0.0007	0.0039	-0.8476
Hpx	3.1755	-1.8496	4.5248	0.0007	0.0039	-0.326
Cyb561	-1.7095	5.1552	-4.5238	0.0007	0.0039	-0.7827
Cactin	-0.5333	5.2732	-4.5229	0.0007	0.0039	-0.8736
Snrpc	-0.4747	6.0238	-4.5228	0.0007	0.0039	-0.9439
Fam189a2	3.2433	-0.7701	4.5228	0.0007	0.0039	-0.2111
Gabbr1	0.9456	3.8886	4.5228	0.0007	0.0039	-0.6647
Ttc3	0.3777	7.3522	4.5216	0.0007	0.0039	-0.9916
Il2rb	2.0457	0.9969	4.5201	0.0007	0.0039	-0.2357
Zfp81	-0.6895	3.7068	-4.52	0.0007	0.0039	-0.6341
Ap3s2	0.5272	5.2963	4.5181	0.0007	0.0039	-0.9017
Lmbrd2	-0.8463	5.9877	-4.5158	0.0007	0.0039	-0.941
Pgf	-3.0804	-0.4717	-4.5154	0.0007	0.0039	-0.2141
Dtwd1	-1.1977	4.0362	-4.5151	0.0007	0.0039	-0.6481

Ctso	1.5749	3.0934	4.5147	0.0007	0.0039	-0.5277
Cyba	1.5841	2.7564	4.5133	0.0007	0.004	-0.4925
Mbnl3	-1.2138	4.5952	-4.5128	0.0007	0.004	-0.7303
Mtmr9	0.4941	5.2809	4.5123	0.0007	0.004	-0.9097
Pcdh18	2.4827	4.5629	4.5114	0.0007	0.004	-0.7203
Metap2	-0.5143	7.8068	-4.5114	0.0007	0.004	-1.0146
Dnajb5	1.0672	3.5511	4.5092	0.0007	0.004	-0.6413
Rbm15b	0.5407	6.9167	4.5082	0.0007	0.004	-1.008
Gm5841	-1.5877	0.4062	-4.5074	0.0007	0.004	-0.1946
Wdr89	-0.7435	4.0136	-4.507	0.0007	0.004	-0.7181
Kif13b	0.8278	5.0494	4.5069	0.0007	0.004	-0.8915
Gm37240	2.2069	-1.1098	4.5062	0.0007	0.004	-0.219
Syf2	0.6135	5.2207	4.5052	0.0007	0.004	-0.917
Rgs17	-1.1688	3.6653	-4.505	0.0007	0.004	-0.6072
Psen2	2.226	1.0115	4.5045	0.0007	0.004	-0.2343
Msh3	-0.6984	5.309	-4.5044	0.0007	0.004	-0.898
Trim34a	2.5596	-0.9632	4.5031	0.0007	0.004	-0.2202
Wdr92	-0.5622	4.9305	-4.5024	0.0007	0.004	-0.8762
Dbnl	0.435	7.124	4.5024	0.0007	0.004	-1.0224
Bfar	0.5737	5.0726	4.5015	0.0007	0.004	-0.9052
Optn	1.2782	4.197	4.4974	0.0007	0.004	-0.7844
Pym1	-0.6331	4.2599	-4.4962	0.0007	0.004	-0.7795
Ifit3	2.6423	0.4591	4.4956	0.0007	0.004	-0.2224
Arsa	0.771	6.2517	4.4954	0.0007	0.004	-1.0123
Slc10a3	0.7079	4.0517	4.4952	0.0007	0.004	-0.761
Heatr1	-0.7632	7.3478	-4.4952	0.0007	0.004	-1.0337
Hars	-0.6313	7.3859	-4.4948	0.0007	0.004	-1.0375
Enpp2	2.46	6.2161	4.4925	0.0007	0.0041	-0.9761
Oard1	-0.6945	4.2944	-4.4914	0.0007	0.0041	-0.7964
Gamt	1.2515	3.0902	4.4903	0.0007	0.0041	-0.5907
Smco4	-1.2407	3.4307	-4.4896	0.0007	0.0041	-0.5973
Anapc2	0.4672	6.8794	4.4886	0.0007	0.0041	-1.0428
Scamp3	0.4867	6.2751	4.4876	0.0007	0.0041	-1.0263
Morc2a	0.5452	7.3806	4.4875	0.0007	0.0041	-1.0532
Nupr1	1.5109	6.0432	4.4864	0.0008	0.0041	-1.0168
Farsa	-0.5754	6.7473	-4.486	0.0008	0.0041	-1.0398
Nrf1	-0.4613	5.591	-4.4849	0.0008	0.0041	-0.9778
Gpx8	0.6374	5.6448	4.4848	0.0008	0.0041	-0.9948
Spg11	0.5821	5.4999	4.4844	0.0008	0.0041	-0.9803
Smim8	0.86	2.6841	4.4832	0.0008	0.0041	-0.5772
Zfp330	-0.3983	5.5773	-4.4832	0.0008	0.0041	-0.9824
Trim59	-0.7525	7.2926	-4.4832	0.0008	0.0041	-1.0545
Adamts5	3.2806	-0.1683	4.483	0.0008	0.0041	-0.2391
Ccdc157	0.5571	4.4098	4.4829	0.0008	0.0041	-0.8514
Mrps24	-0.5558	5.757	-4.482	0.0008	0.0041	-0.9986
Gm14963	-1.7485	-0.0774	-4.4785	0.0008	0.0041	-0.2365
4933407K13Rik	0.9941	2.2379	4.4782	0.0008	0.0041	-0.4706
Bri3bp	-0.5818	6.6559	-4.4782	0.0008	0.0041	-1.05
Yae1d1	-0.6501	4.9061	-4.4781	0.0008	0.0041	-0.8978
Kbtbd2	-0.3772	6.3269	-4.4773	0.0008	0.0041	-1.0416
Hsph1	-1.0164	8.3072	-4.4769	0.0008	0.0041	-1.082
Ubqln1	-0.5267	8.0947	-4.4761	0.0008	0.0041	-1.0817

Peli3	3.6799	-0.2755	4.4756	0.0008	0.0042	-0.2589
Qsox2	-0.5822	4.6277	-4.4754	0.0008	0.0042	-0.8845
Gm7964	-0.4694	4.9408	-4.4753	0.0008	0.0042	-0.9274
E2f3	-0.7279	5.6817	-4.4752	0.0008	0.0042	-0.9892
Zfp729a	0.8316	4.0938	4.4747	0.0008	0.0042	-0.7987
Psme4	1.2283	8.5631	4.4723	0.0008	0.0042	-1.0965
Pcdhb21	1.8379	0.297	4.4705	0.0008	0.0042	-0.2811
Sf3a1	-0.44	7.4388	-4.4702	0.0008	0.0042	-1.0828
Pigf	-0.8111	3.7673	-4.4702	0.0008	0.0042	-0.725
Gfod1	-0.908	6.2486	-4.4699	0.0008	0.0042	-1.039
Zfp810	1.6385	1.7705	4.4676	0.0008	0.0042	-0.4276
Sdhd	-0.4291	7.4519	-4.4662	0.0008	0.0042	-1.0904
Glyr1	0.4008	7.3034	4.466	0.0008	0.0042	-1.0907
Ppp4c	-0.4232	6.6412	-4.4659	0.0008	0.0042	-1.0745
Nr2c2ap	-0.5324	4.6329	-4.4655	0.0008	0.0042	-0.8935
Meis2	0.7489	4.5465	4.4655	0.0008	0.0042	-0.8901
Abca2	1.3126	5.8886	4.4647	0.0008	0.0042	-1.0375
C1rb	3.4726	-1.7095	4.4645	0.0008	0.0042	-0.3822
Ppp2r2d	-0.5679	5.6214	-4.4622	0.0008	0.0042	-1.0182
Trappc10	0.583	5.672	4.4622	0.0008	0.0042	-1.038
Lsm14a	-0.3966	7.0903	-4.462	0.0008	0.0042	-1.0916
Nepro	-0.6777	4.4847	-4.4619	0.0008	0.0042	-0.8535
Ptgr2	0.5566	5.1796	4.4616	0.0008	0.0042	-0.9946
Sec16a	0.6317	6.9603	4.4613	0.0008	0.0042	-1.0926
Lctl	5.674	-0.63	4.4612	0.0008	0.0042	-0.4376
Gtf3c2	-0.6174	5.8988	-4.4608	0.0008	0.0042	-1.0409
Mapt	2.5214	-1.3298	4.4597	0.0008	0.0042	-0.3011
Senp7	0.5283	4.8056	4.4592	0.0008	0.0042	-0.9481
Cenpx	-0.8774	4.3832	-4.4576	0.0008	0.0042	-0.8453
Cox5a	-0.4114	7.3848	-4.4571	0.0008	0.0042	-1.1066
Wdr74	-0.807	5.6128	-4.457	0.0008	0.0042	-1.0162
Slc35a5	0.7738	3.5467	4.4569	0.0008	0.0042	-0.7503
Id1	-1.8106	5.1147	-4.4549	0.0008	0.0043	-0.8818
Morrbid	-1.4282	1.9524	-4.4548	0.0008	0.0043	-0.4124
Tsc22d2	1.1921	6.4064	4.453	0.0008	0.0043	-1.0887
Zfat	0.6699	3.8606	4.4524	0.0008	0.0043	-0.8098
Kif26b	2.3124	4.0803	4.4518	0.0008	0.0043	-0.7525
Thbs1	3.3148	5.6899	4.4495	0.0008	0.0043	-0.9296
Copg1	-0.4375	8.1381	-4.4467	0.0008	0.0043	-1.1363
Tnrc6c	0.6416	6.1435	4.4463	0.0008	0.0043	-1.0907
Sec14l5	3.1194	-3.1389	4.4461	0.0008	0.0043	-0.5763
Ctsz	0.455	7.4671	4.445	0.0008	0.0043	-1.1317
Kti12	-0.7947	5.3995	-4.4438	0.0008	0.0043	-1.015
Tdg	-0.4764	4.4988	-4.4428	0.0008	0.0043	-0.9149
Eif1a	-0.6441	7.6031	-4.4421	0.0008	0.0043	-1.1344
Traf3ip1	-0.5977	4.5292	-4.4417	0.0008	0.0043	-0.9177
Ocel1	1.0568	3.0514	4.4416	0.0008	0.0043	-0.6932
Tmem201	-0.667	5.1155	-4.441	0.0008	0.0043	-0.9938
Insyn1	1.3956	1.6512	4.4404	0.0008	0.0043	-0.4618
Rab11fip3	0.6116	5.5565	4.4402	0.0008	0.0043	-1.063
Col6a2	1.6059	5.325	4.4402	0.0008	0.0043	-1.0057
Apon	2.5849	-2.2889	4.4401	0.0008	0.0043	-0.3983

Myef2	-1.019	5.9664	-4.4399	0.0008	0.0043	-1.0711
Slc17a5	0.7528	4.4843	4.4394	0.0008	0.0043	-0.9338
Slc9a2	-3.003	2.8883	-4.439	0.0008	0.0044	-0.4354
Samd14	1.3575	4.1001	4.4381	0.0008	0.0044	-0.8523
Ado	-0.785	5.6177	-4.4374	0.0008	0.0044	-1.0486
Csrp2	-0.752	3.7331	-4.4367	0.0008	0.0044	-0.8005
Nbeal2	-1.1396	4.6551	-4.434	0.0008	0.0044	-0.903
Tyk2	0.7034	5.3531	4.4321	0.0008	0.0044	-1.0579
Podxl2	2.9575	2.6607	4.4317	0.0008	0.0044	-0.5141
Wbp2	0.5886	7.4675	4.431	0.0008	0.0044	-1.1574
Cyth1	0.6439	4.9354	4.4303	0.0008	0.0044	-1.0143
Klhl26	0.7279	4.4788	4.4294	0.0008	0.0044	-0.9541
Zfp407	0.4554	5.2234	4.4291	0.0008	0.0044	-1.0498
Tspan2	1.8053	4.2179	4.4287	0.0008	0.0044	-0.8481
Taf3	-0.4084	5.3016	-4.4276	0.0008	0.0044	-1.0559
Maco1	0.3983	6.164	4.4274	0.0008	0.0044	-1.1277
Dtna	2.0669	-0.2146	4.4273	0.0008	0.0044	-0.3256
Rsl24d1	-0.6282	6.219	-4.4269	0.0008	0.0044	-1.1218
4931406C07Rik	1.0016	5.4916	4.4262	0.0008	0.0044	-1.0805
Mmd	-0.8625	6.385	-4.4223	0.0008	0.0045	-1.132
Ccn2	1.3892	3.6728	4.4211	0.0008	0.0045	-0.8041
2810433D01Rik	3.6809	-3.3384	4.4206	0.0008	0.0045	-0.6173
Ciao2a	-0.4697	6.0156	-4.4198	0.0008	0.0045	-1.1276
Vamp4	-0.6761	5.3084	-4.4192	0.0008	0.0045	-1.057
Ccdc85c	-0.5706	6.4841	-4.4186	0.0008	0.0045	-1.1497
Gm16001	1.2753	1.4592	4.418	0.0008	0.0045	-0.4737
Npc1	0.8856	6.3231	4.4173	0.0008	0.0045	-1.1528
Greb1l	1.5996	2.2035	4.4168	0.0008	0.0045	-0.5737
Gbp6	3.0187	-2.3849	4.416	0.0008	0.0045	-0.4681
Wwc1	-1.2716	5.2417	-4.4155	0.0008	0.0045	-1.0303
Lurap1	1.0973	1.1164	4.4144	0.0009	0.0045	-0.4462
Trim17	3.0792	-3.1535	4.4144	0.0009	0.0045	-0.6119
Ttyh3	1.5972	5.9086	4.4103	0.0009	0.0045	-1.1289
2310001H17Rik	2.7285	-0.1318	4.4064	0.0009	0.0046	-0.3502
Esyt2	0.6575	7.7878	4.4063	0.0009	0.0046	-1.2055
Thsd1	1.5612	-0.2257	4.406	0.0009	0.0046	-0.3569
Rasa2	-1.1304	5.0003	-4.4058	0.0009	0.0046	-1.0149
Trappc2l	0.4965	4.5287	4.4053	0.0009	0.0046	-1.0159
Acsl4	-0.8765	8.5376	-4.4048	0.0009	0.0046	-1.2146
Upf1	-0.5619	6.9443	-4.4041	0.0009	0.0046	-1.191
Ugcg	-1.0012	5.1201	-4.403	0.0009	0.0046	-1.0426
Wdr45b	-0.4259	6.7748	-4.402	0.0009	0.0046	-1.1926
Gm15428	-3.9067	-0.3242	-4.402	0.0009	0.0046	-0.4778
Polr3d	-0.6453	4.8806	-4.4019	0.0009	0.0046	-1.0345
Gstm7	2.5369	2.2149	4.4019	0.0009	0.0046	-0.545
Sart3	-0.501	6.4348	-4.4013	0.0009	0.0046	-1.1811
Snhg4	-1.2555	3.4318	-4.4008	0.0009	0.0046	-0.7502
Ap5b1	1.0953	1.9165	4.4008	0.0009	0.0046	-0.5755
4930558J18Rik	-2.4392	0.2958	-4.4007	0.0009	0.0046	-0.3592
Fndc3a	-0.7134	7.4294	-4.4001	0.0009	0.0046	-1.2076
Zfp738	0.6239	3.4099	4.3988	0.0009	0.0046	-0.834
Sri	0.4529	6.4547	4.3984	0.0009	0.0046	-1.1925

Gm19412	1.5136	0.8858	4.3981	0.0009	0.0046	-0.4545
Lats2	0.911	3.935	4.3963	0.0009	0.0046	-0.9079
Gm16364	1.6705	-1.2516	4.3954	0.0009	0.0046	-0.3755
Acaa1a	0.945	4.6611	4.3949	0.0009	0.0046	-1.0407
Gsdme	-2.3804	2.6257	-4.3948	0.0009	0.0046	-0.5282
Nol9	-0.7462	6.0971	-4.3948	0.0009	0.0046	-1.167
Gm10432	2.7464	-3.5093	4.3947	0.0009	0.0046	-0.6329
Smad6	1.6372	1.4586	4.3946	0.0009	0.0046	-0.5197
Vps13d	0.8081	5.0959	4.394	0.0009	0.0046	-1.0939
BC067074	1.9981	0.4901	4.3934	0.0009	0.0046	-0.4122
Gm12346	-0.8847	2.3918	-4.3933	0.0009	0.0046	-0.6452
Pgm2	-0.5188	5.8016	-4.3931	0.0009	0.0046	-1.1616
Decr1	0.827	4.796	4.3931	0.0009	0.0046	-1.0605
Sohlh2	2.9146	-3.7361	4.3922	0.0009	0.0046	-0.6387
Nhsl1	1.794	5.1062	4.3914	0.0009	0.0046	-1.0927
Dhps	-0.6748	4.7109	-4.3896	0.0009	0.0046	-1.0397
Tmem106a	1.7082	3.0042	4.3893	0.0009	0.0047	-0.7292
Usp6nl	-0.4828	6.2315	-4.3873	0.0009	0.0047	-1.1969
Pcca	0.5316	4.1872	4.3869	0.0009	0.0047	-0.9965
Zfhx3	1.1632	5.8217	4.3857	0.0009	0.0047	-1.1763
Scly	1.4135	3.7833	4.3854	0.0009	0.0047	-0.9001
Snx11	0.6417	3.7162	4.3846	0.0009	0.0047	-0.901
Eme2	0.9797	3.2864	4.3843	0.0009	0.0047	-0.8249
Dlg4	-0.8162	5.2513	-4.383	0.0009	0.0047	-1.1134
Zfp282	0.6867	4.919	4.3825	0.0009	0.0047	-1.0935
Unc119	0.7323	3.9045	4.3819	0.0009	0.0047	-0.9365
Mea1	0.5113	5.6002	4.3819	0.0009	0.0047	-1.1777
Srrm1	-0.5307	7.4395	-4.3805	0.0009	0.0047	-1.2438
Nrarp	-4.2226	1.9943	-4.3799	0.0009	0.0047	-0.4024
Casq2	3.2961	-2.0199	4.3796	0.0009	0.0047	-0.5935
Gm20412	3.0294	-1.2021	4.3794	0.0009	0.0047	-0.4262
Srprb	-0.5321	5.2914	-4.378	0.0009	0.0047	-1.1375
Ctr9	0.4248	6.3494	4.3776	0.0009	0.0047	-1.2254
Scn2a	1.4835	2.1839	4.3765	0.0009	0.0047	-0.6315
Vcam1	2.7857	4.4187	4.3765	0.0009	0.0047	-0.9519
Gm28809	-2.1024	1.2047	-4.3765	0.0009	0.0047	-0.4336
Mdc1	-0.6706	6.246	-4.3764	0.0009	0.0047	-1.2112
Lrmda	1.4224	0.1427	4.3746	0.0009	0.0047	-0.4284
Tm2d3	0.6251	3.6182	4.3729	0.0009	0.0048	-0.925
Hsd3b7	0.9169	2.172	4.3727	0.0009	0.0048	-0.6731
Slc35g1	-1.2374	5.029	-4.3722	0.0009	0.0048	-1.0628
Tbcd	0.4447	6.3207	4.3707	0.0009	0.0048	-1.2379
Irf4	2.9678	-3.21	4.3704	0.0009	0.0048	-0.67
Ftl1-ps1	1.3615	0.6928	4.3701	0.0009	0.0048	-0.4834
Trim3	0.7781	5.1403	4.3663	0.0009	0.0048	-1.16
Tsga10	0.9431	1.8453	4.3655	0.0009	0.0048	-0.6349
Stoml1	1.236	2.701	4.3652	0.0009	0.0048	-0.7574
Parm1	2.8413	1.208	4.365	0.0009	0.0048	-0.5098
H2-T-ps	1.786	2.4422	4.364	0.0009	0.0048	-0.6892
Mllt1	-0.5763	6.8437	-4.3628	0.0009	0.0048	-1.2634
Slc4a11	-1.7446	2.06	-4.3628	0.0009	0.0048	-0.5677
Emilin1	1.6981	5.6224	4.3626	0.0009	0.0048	-1.1851

Fras1	2.9897	-1.2524	4.3621	0.0009	0.0048	-0.4842
Gm9770	1.1543	2.0833	4.362	0.0009	0.0048	-0.6741
Cox18	-0.704	4.2924	-4.3609	0.0009	0.0048	-1.0202
Mrpl49	-0.4683	5.999	-4.3599	0.0009	0.0048	-1.2354
Layn	-0.7276	5.2573	-4.3599	0.0009	0.0048	-1.1705
Dolk	0.5125	4.1988	4.3598	0.0009	0.0048	-1.036
Tep1	0.4871	4.5549	4.3594	0.0009	0.0048	-1.0907
Ier3ip1	-0.5124	5.5016	-4.3592	0.0009	0.0048	-1.1972
5730414N17Rik	2.0956	-1.6933	4.3585	0.0009	0.0048	-0.46
Clasrp	-0.5767	4.5642	-4.3582	0.0009	0.0048	-1.0746
Irgm2	2.2075	2.2323	4.358	0.0009	0.0048	-0.635
Cpped1	0.7032	5.1138	4.3579	0.0009	0.0048	-1.1666
Asph	-0.6914	7.384	-4.3568	0.0009	0.0049	-1.2849
Gorab	-0.7078	4.8782	-4.3566	0.0009	0.0049	-1.1151
Zc3h8	-0.9891	2.8894	-4.356	0.0009	0.0049	-0.7676
Myo6	1.2789	5.468	4.3545	0.0009	0.0049	-1.1936
Eva1c	-1.8356	2.9615	-4.3538	0.0009	0.0049	-0.6943
Dnmt1	-0.7951	8.1535	-4.3538	0.0009	0.0049	-1.3021
Med14	-0.619	6.6243	-4.3514	0.001	0.0049	-1.2743
Ubap2	-0.486	7.2597	-4.3509	0.001	0.0049	-1.2938
Tnk2	-0.6223	6.3479	-4.3499	0.001	0.0049	-1.2692
Zbed3	-0.6539	4.6574	-4.3498	0.001	0.0049	-1.1064
Nkrf	-0.9715	4.6516	-4.3485	0.001	0.0049	-1.0671
Gm13375	1.048	1.9271	4.348	0.001	0.0049	-0.6602
Nme2	-0.4081	6.8108	-4.3477	0.001	0.0049	-1.2915
Matr3	-0.5122	7.5734	-4.3477	0.001	0.0049	-1.3042
Zrsr2	-0.6265	6.0542	-4.3473	0.001	0.0049	-1.2588
Gm5812	-1.3462	1.2056	-4.3471	0.001	0.0049	-0.5352
Mrpl13	-0.7907	6.5939	-4.3466	0.001	0.0049	-1.2846
Eif2s3x	-0.5071	6.9262	-4.3462	0.001	0.0049	-1.2957
Zfp108	2.2218	0.1136	4.3456	0.001	0.0049	-0.4517
Tmem241	0.7598	3.2351	4.3456	0.001	0.0049	-0.8879
Sh2d5	-2.7812	4.7725	-4.3455	0.001	0.0049	-0.9146
Lgi2	-2.1522	0.7442	-4.3445	0.001	0.0049	-0.4678
Plxdc1	4.3441	0.6504	4.344	0.001	0.0049	-0.4526
4930488L21Rik	2.7651	-2.03	4.3436	0.001	0.0049	-0.5662
Ifi35	0.9186	4.7775	4.3425	0.001	0.0049	-1.1488
Oacyl	3.1714	-3.2244	4.342	0.001	0.0049	-0.7114
Atg13	0.6358	5.6261	4.3419	0.001	0.0049	-1.2488
Psmd5	-0.5119	6.9994	-4.3412	0.001	0.0049	-1.307
Tradd	0.5148	4.3214	4.3411	0.001	0.0049	-1.087
Cd1d1	1.5464	4.2278	4.341	0.001	0.0049	-1.0495
Cwc25	0.478	4.9853	4.3409	0.001	0.0049	-1.1827
Stag1	-0.6331	7.4171	-4.3401	0.001	0.005	-1.3159
Tmem184c	0.5174	5.8796	4.3395	0.001	0.005	-1.2673
Mocs3	0.7179	2.9286	4.3394	0.001	0.005	-0.8608
Gm10110	-0.7828	2.7342	-4.3393	0.001	0.005	-0.7841
Tcf7	-0.968	3.9881	-4.3389	0.001	0.005	-0.9728
Crls1	-0.6391	4.8885	-4.3387	0.001	0.005	-1.1569
Tbc1d20	0.4884	6.6431	4.3386	0.001	0.005	-1.306
Tmem140	5.5866	0.934	4.3383	0.001	0.005	-0.4871
Phf14	0.5533	5.9468	4.3379	0.001	0.005	-1.2784

Clasp1	0.5197	6.7858	4.3372	0.001	0.005	-1.3124
Stx8	0.7658	4.2308	4.3371	0.001	0.005	-1.0802
Ift22	0.5535	4.0375	4.3368	0.001	0.005	-1.0452
Nmt2	-0.4694	6.4987	-4.3365	0.001	0.005	-1.3013
Ror1	2.1581	2.4678	4.3348	0.001	0.005	-0.6937
Lix1l	0.434	7.2511	4.3347	0.001	0.005	-1.3269
Cdadc1	0.6014	4.191	4.334	0.001	0.005	-1.072
Paxbp1	-0.4263	5.4521	-4.3333	0.001	0.005	-1.2356
Larp7	-0.4874	6.7091	-4.3321	0.001	0.005	-1.3154
Zfp84	0.6614	3.5746	4.33	0.001	0.005	-0.9747
Ogg1	-0.8753	3.8396	-4.33	0.001	0.005	-0.9891
Dnajc30	0.6519	3.5576	4.3281	0.001	0.005	-0.991
Szrd1	-0.5707	6.6684	-4.3273	0.001	0.005	-1.3214
Aebp2	-0.4509	7.1177	-4.3273	0.001	0.005	-1.334
Mbd3	-0.6032	6.7569	-4.3273	0.001	0.005	-1.3248
Mbtps2	-0.5785	6.0607	-4.3252	0.001	0.005	-1.2968
Zbtb5	0.8373	3.9275	4.3244	0.001	0.0051	-1.0458
Pycr1	-0.7912	3.9848	-4.3241	0.001	0.0051	-1.0412
Bms1	-0.5398	7.5221	-4.3238	0.001	0.0051	-1.3478
Txnl4b	0.5586	3.3978	4.3213	0.001	0.0051	-0.9707
4632404H12Rik	1.032	2.3075	4.3202	0.001	0.0051	-0.7864
Zmat5	0.7081	4.25	4.32	0.001	0.0051	-1.1243
Cpsf1	-0.4285	7.1719	-4.32	0.001	0.0051	-1.3492
5730409E04Rik	1.4528	3.2432	4.3199	0.001	0.0051	-0.897
Usp39	-0.374	6.4635	-4.3195	0.001	0.0051	-1.3311
Ring1	0.8691	3.006	4.3186	0.001	0.0051	-0.9027
Zfp456	1.4627	0.6814	4.3183	0.001	0.0051	-0.5463
Mul1	0.5252	4.3194	4.3177	0.001	0.0051	-1.1268
Fam193a	0.6479	5.6189	4.3174	0.001	0.0051	-1.2878
Parp11	0.7882	3.225	4.317	0.001	0.0051	-0.9478
Luc7l2	-0.5854	7.2093	-4.3168	0.001	0.0051	-1.354
Tedc2	-0.667	3.7743	-4.3148	0.001	0.0051	-1.0164
Hlx	0.9419	2.415	4.3146	0.001	0.0051	-0.795
Cyb5a	0.6013	6.2692	4.3138	0.001	0.0051	-1.339
Ddx10	-0.4926	6.4769	-4.3129	0.001	0.0051	-1.3425
Rusc1	1.0082	1.914	4.3118	0.001	0.0051	-0.7523
Hlcs	0.739	3.6365	4.3117	0.001	0.0051	-1.023
Coq5	-0.4868	4.9366	-4.3115	0.001	0.0051	-1.2204
Gm44432	2.998	-3.4688	4.3111	0.001	0.0051	-0.7457
Kcnt1	4.871	-1.1919	4.3099	0.001	0.0051	-0.6617
Gm26692	-2.6103	-2.7018	-4.3096	0.001	0.0051	-0.7358
Mon2	0.4574	6.395	4.3095	0.001	0.0051	-1.35
Kmt5b	0.6258	5.9234	4.3094	0.001	0.0051	-1.3257
Itgb5	1.4886	6.3607	4.3087	0.001	0.0052	-1.3448
Ahsa1	-0.5789	7.3228	-4.3078	0.001	0.0052	-1.3736
Gnb1	-0.3456	8.8237	-4.3059	0.001	0.0052	-1.3987
Gdi1	0.4297	6.7133	4.3054	0.001	0.0052	-1.3696
Ptpn21	0.6495	5.6933	4.3052	0.001	0.0052	-1.3209
Serf2	0.5007	7.0813	4.3049	0.001	0.0052	-1.3785
Plek2	-2.2689	0.2098	-4.3038	0.001	0.0052	-0.5148
C1qtnf1	2.3097	4.9952	4.3037	0.001	0.0052	-1.1519
Kalrn	3.6051	2.7091	4.3031	0.001	0.0052	-0.7149

Mitf	-0.6814	4.2052	-4.3031	0.001	0.0052	-1.1087
Afg3l1	-0.5342	6.4094	-4.3015	0.001	0.0052	-1.3629
Znrf2	-0.5093	5.4806	-4.3014	0.001	0.0052	-1.2978
Efcab11	-1.0419	1.0943	-4.3009	0.001	0.0052	-0.6197
Insc	4.3526	-2.5433	4.3006	0.001	0.0052	-0.8136
Psme2	0.9461	7.0092	4.3005	0.001	0.0052	-1.384
Sgk2	-2.9672	1.6051	-4.3	0.001	0.0052	-0.5541
Ints13	-0.5487	6.2714	-4.2984	0.001	0.0052	-1.3596
Cyld	0.7124	6.008	4.298	0.001	0.0052	-1.3503
Rps2-ps10	-0.4348	7.2654	-4.2979	0.001	0.0052	-1.3916
Gsk3a	-0.3861	6.4421	-4.2977	0.001	0.0052	-1.3712
Senp2	-0.4172	6.3134	-4.297	0.001	0.0052	-1.3665
Hsd17b4	0.6024	6.7153	4.2966	0.001	0.0052	-1.3854
Maged2	0.4794	6.4991	4.296	0.001	0.0052	-1.3785
Fnip2	0.6952	5.8479	4.2959	0.001	0.0052	-1.3427
Adcy9	2.089	2.5376	4.2954	0.001	0.0052	-0.8306
Paxx	1.4865	2.266	4.2949	0.0011	0.0052	-0.8125
Zc4h2	1.1995	3.3854	4.291	0.0011	0.0053	-0.9983
Itpkc	0.6232	3.6538	4.2902	0.0011	0.0053	-1.0667
Kdelc1	-0.5501	5.9336	-4.289	0.0011	0.0053	-1.3574
5930403N24Rik	2.1382	-2.0466	4.2889	0.0011	0.0053	-0.612
Elovl6	-1.0882	7.7025	-4.2879	0.0011	0.0053	-1.4134
Ints10	-0.5167	5.9761	-4.2871	0.0011	0.0053	-1.3636
Cops8	-0.4243	6.4923	-4.2851	0.0011	0.0053	-1.3954
Fdxr	-0.6095	3.4526	-4.2837	0.0011	0.0053	-1.0322
Coro6	2.5617	1.3368	4.2824	0.0011	0.0053	-0.6566
Ppp1r12c	-0.3899	5.6509	-4.2806	0.0011	0.0054	-1.356
Tead2	1.04	6.5562	4.2805	0.0011	0.0054	-1.4084
4930506C21Rik	2.9898	-2.9864	4.2798	0.0011	0.0054	-0.7895
Seh1l	-0.6543	7.3963	-4.2795	0.0011	0.0054	-1.4247
Gm5602	1.9795	-2.2446	4.2789	0.0011	0.0054	-0.6052
N4bp2l1	1.6202	1.9193	4.2786	0.0011	0.0054	-0.7707
Isca1	-0.605	5.2041	-4.2781	0.0011	0.0054	-1.3041
Pes1	-0.4682	7.7794	-4.2771	0.0011	0.0054	-1.4371
Col9a3	2.9629	-2.5506	4.2767	0.0011	0.0054	-0.7048
Gm12100	3.6923	-3.4047	4.2745	0.0011	0.0054	-0.8064
Fmc1	-0.8332	3.4593	-4.2745	0.0011	0.0054	-1.0303
Gm47662	2.9356	-3.0645	4.2734	0.0011	0.0054	-0.7303
Prpf31	-0.6443	5.9581	-4.2733	0.0011	0.0054	-1.3811
Adrb2	1.6959	0.6373	4.2711	0.0011	0.0054	-0.6338
Mmp16	2.2795	0.6207	4.271	0.0011	0.0054	-0.6163
Rtf2	0.5587	6.0537	4.2707	0.0011	0.0054	-1.4062
Nudt7	1.1139	2.0208	4.2696	0.0011	0.0054	-0.8294
Adamts4	2.8086	5.935	4.2689	0.0011	0.0054	-1.3601
Dhrs13	-0.9182	3.6949	-4.2688	0.0011	0.0054	-1.0513
Usp32	0.6523	6.349	4.2674	0.0011	0.0055	-1.4255
Gm14928	-2.6747	-2.3883	-4.2657	0.0011	0.0055	-0.7694
Tspan15	1.7525	4.5647	4.2652	0.0011	0.0055	-1.2254
Kif9	2.0816	-0.9546	4.2649	0.0011	0.0055	-0.5752
Prl6a1	-2.9758	-3.0144	-4.2644	0.0011	0.0055	-0.8054
Frmd8	0.8106	6.7802	4.2643	0.0011	0.0055	-1.4447
Dtx3	0.8027	4.06	4.2627	0.0011	0.0055	-1.1756

Arl8a	0.4397	6.0589	4.2625	0.0011	0.0055	-1.4243
Serac1	0.7621	2.882	4.2623	0.0011	0.0055	-0.9857
Tmc6	0.5904	4.7361	4.2623	0.0011	0.0055	-1.3013
Psen1	0.4556	6.2797	4.2615	0.0011	0.0055	-1.4327
Arrdc2	1.1755	4.1757	4.2599	0.0011	0.0055	-1.2009
Fam120b	0.5483	5.3494	4.2599	0.0011	0.0055	-1.377
Pawr	0.7254	4.5473	4.2597	0.0011	0.0055	-1.2778
Shq1	-0.7845	4.6029	-4.2594	0.0011	0.0055	-1.2409
Rapgef2	0.7759	5.6567	4.2587	0.0011	0.0055	-1.4001
Zfp511	-0.6213	4.1439	-4.2586	0.0011	0.0055	-1.1819
Tspan9	0.5505	7.095	4.2579	0.0011	0.0055	-1.4638
Caskin2	0.6275	4.9416	4.2573	0.0011	0.0055	-1.3288
Eya2	-6.1825	1.8039	-4.2546	0.0011	0.0055	-0.6679
Timm17a	-0.5164	7.0078	-4.2544	0.0011	0.0055	-1.465
Vps37b	-0.6528	6.5934	-4.2543	0.0011	0.0055	-1.452
Mettl16	-0.7642	6.3322	-4.2535	0.0011	0.0056	-1.4383
Gm16510	-2.2926	-2.6414	-4.2526	0.0011	0.0056	-0.7487
Atf6b	-0.5605	6.8853	-4.2525	0.0011	0.0056	-1.4648
Gm20633	1.7416	-0.1512	4.2504	0.0011	0.0056	-0.6069
Vbp1	-0.762	6.8333	-4.2504	0.0011	0.0056	-1.4644
Agfg1	-0.5663	6.6644	-4.2503	0.0011	0.0056	-1.4617
Phkb	0.748	4.8693	4.2502	0.0011	0.0056	-1.3353
Cideb	3.6521	-1.6248	4.2499	0.0011	0.0056	-0.7306
Abca9	4.1341	-1.3801	4.2494	0.0011	0.0056	-0.7316
Gm7846	-1.914	0.6922	-4.2492	0.0011	0.0056	-0.6256
Dnajc16	0.4246	4.5052	4.2492	0.0011	0.0056	-1.2812
Naa20	-0.8531	5.6059	-4.2462	0.0011	0.0056	-1.3909
Gla	0.4591	5.4652	4.2459	0.0011	0.0056	-1.4085
Rtl5	3.6014	-0.0894	4.2456	0.0011	0.0056	-0.612
BC029722	0.6643	4.8967	4.2455	0.0011	0.0056	-1.3493
Sema6c	2.4235	1.9118	4.2444	0.0011	0.0056	-0.784
Morf4l2	-0.513	8.7212	-4.2438	0.0011	0.0056	-1.5098
Mpzl1	0.7115	6.0865	4.2431	0.0012	0.0056	-1.4582
9530062K07Rik	2.051	-0.8577	4.2427	0.0012	0.0056	-0.6114
Zfand1	1.1062	3.7458	4.2408	0.0012	0.0056	-1.1638
Cdkn2a	0.6007	8.8883	4.2407	0.0012	0.0056	-1.5187
Smim19	0.7188	3.5384	4.2407	0.0012	0.0056	-1.1329
Gm49759	-0.9242	5.9395	-4.2401	0.0012	0.0057	-1.4303
AC122861.1	-1.6882	-0.2959	-4.2397	0.0012	0.0057	-0.6168
Dbn1	1.7719	5.2716	4.2395	0.0012	0.0057	-1.3688
Gpr135	1.1879	1.2032	4.2394	0.0012	0.0057	-0.7368
Naga	1.2782	3.8127	4.2393	0.0012	0.0057	-1.1787
Acads	0.7256	5.7015	4.2374	0.0012	0.0057	-1.4458
0610040B10Rik	1.8608	-0.5979	4.2365	0.0012	0.0057	-0.6211
Nudt19	-0.7066	3.6587	-4.2365	0.0012	0.0057	-1.136
Brca1	-0.9127	6.1801	-4.2363	0.0012	0.0057	-1.4561
Gm26797	3.0873	-1.9928	4.2359	0.0012	0.0057	-0.7134
Prpf38a	-0.4398	5.7106	-4.2355	0.0012	0.0057	-1.436
Il18	2.9339	2.5708	4.2347	0.0012	0.0057	-0.8429
Terf2ip	0.6913	4.6791	4.2335	0.0012	0.0057	-1.334
Zhx3	2.1107	3.1341	4.2317	0.0012	0.0057	-1.0024
Rrp12	-1.227	5.544	-4.2309	0.0012	0.0057	-1.3925

Dhx57	0.578	5.0543	4.2294	0.0012	0.0057	-1.3966
Magi1	-0.6382	5.6137	-4.2272	0.0012	0.0058	-1.4428
Tpi1	-0.5765	9.8634	-4.2271	0.0012	0.0058	-1.5483
Pcdhb5	1.9218	0.2311	4.2267	0.0012	0.0058	-0.6717
Dync1li1	-0.4405	6.5521	-4.2234	0.0012	0.0058	-1.5103
Hist2h3c2	3.6019	-0.8229	4.2231	0.0012	0.0058	-0.7136
Zcchc10	-0.7447	4.122	-4.2229	0.0012	0.0058	-1.2106
Gm7984	-1.328	0.7284	-4.2228	0.0012	0.0058	-0.7049
Snx15	0.6106	4.7599	4.2217	0.0012	0.0058	-1.3757
Spire1	-0.756	6.0992	-4.2216	0.0012	0.0058	-1.4818
Mrps18b	-0.6501	5.0366	-4.2213	0.0012	0.0058	-1.3826
Ttc28	2.4818	3.9816	4.2196	0.0012	0.0058	-1.1485
Dus1l	-0.4353	6.0096	-4.2194	0.0012	0.0058	-1.4896
Cebpg	-0.5706	6.1389	-4.2185	0.0012	0.0058	-1.4977
Fbh1	0.5258	6.1999	4.2182	0.0012	0.0058	-1.5109
Ppp2ca	-0.4629	8.4293	-4.2179	0.0012	0.0058	-1.5541
Gm42918	2.96	-2.7985	4.2174	0.0012	0.0058	-0.8707
Ndufa12	-0.5048	5.321	-4.2174	0.0012	0.0058	-1.4342
Zmym2	0.3482	6.0984	4.2171	0.0012	0.0058	-1.5051
Nr1h2	0.5293	5.4397	4.2167	0.0012	0.0058	-1.4597
Ston1	0.9244	5.707	4.2155	0.0012	0.0058	-1.4815
Zfp28	1.1096	2.5318	4.2153	0.0012	0.0058	-1.0095
Specc1l	0.4171	6.1142	4.2149	0.0012	0.0058	-1.5102
Serhl	1.2379	3.735	4.2147	0.0012	0.0058	-1.2108
Gm6652	1.6271	-1.0191	4.2142	0.0012	0.0059	-0.6572
Heatr5a	-0.5092	6.529	-4.2136	0.0012	0.0059	-1.5237
Gm16000	3.3098	-3.5649	4.2128	0.0012	0.0059	-0.8798
Tob1	0.7281	5.2719	4.2118	0.0012	0.0059	-1.4495
Gm26935	3.0847	-2.5106	4.2115	0.0012	0.0059	-0.8813
Gm30122	3.0317	-3.2673	4.2109	0.0012	0.0059	-0.881
Gm10053	-2.0533	-1.2049	-4.2107	0.0012	0.0059	-0.6973
Omd	5.6172	-1.9155	4.2106	0.0012	0.0059	-0.9867
E230013L22Rik	1.767	-0.8582	4.2075	0.0012	0.0059	-0.6677
Rnaseh1	-0.6158	3.9645	-4.207	0.0012	0.0059	-1.2375
Clpp	-0.528	5.5383	-4.2064	0.0012	0.0059	-1.4775
Tmem50a	0.6621	5.9691	4.2062	0.0012	0.0059	-1.5197
Rhebl1	-0.7041	3.6713	-4.2056	0.0012	0.0059	-1.2047
Rnf166	0.5912	4.2234	4.2047	0.0012	0.0059	-1.3214
Zfp950	0.6911	3.0649	4.2034	0.0012	0.0059	-1.1081
Pi4kb	0.3902	6.1072	4.2032	0.0012	0.0059	-1.5338
Safb	-0.369	6.96	-4.2022	0.0012	0.006	-1.5591
Cchcr1	-0.5867	4.6582	-4.2021	0.0012	0.006	-1.3694
Heatr3	-0.7395	6.8437	-4.2021	0.0012	0.006	-1.5524
Tmem14a	1.7883	-0.0125	4.1996	0.0012	0.006	-0.7012
Magohb	-1.2337	3.4845	-4.1984	0.0012	0.006	-1.1315
Ipo8	-0.4118	6.4094	-4.1968	0.0012	0.006	-1.5525
Pxk	-0.4758	5.7201	-4.1963	0.0013	0.006	-1.512
Ficd	0.8818	2.3878	4.1954	0.0013	0.006	-1.0297
Cep128	-0.9756	4.3218	-4.1953	0.0013	0.006	-1.3078
Trmt61a	-1.121	3.4837	-4.1953	0.0013	0.006	-1.1303
Cops7a	-0.567	6.8653	-4.1941	0.0013	0.006	-1.5708
G930009F23Rik	1.6211	2.5058	4.1937	0.0013	0.006	-0.9925

Rab4a	0.7168	3.7964	4.1926	0.0013	0.006	-1.2786
Gins3	-0.8858	3.9755	-4.1924	0.0013	0.006	-1.2502
Per1	0.9616	6.5643	4.1916	0.0013	0.006	-1.5695
Prepl	0.4081	5.3179	4.1905	0.0013	0.0061	-1.4985
Myo9b	0.5315	6.9202	4.1901	0.0013	0.0061	-1.583
Mbd2	-0.5804	6.7545	-4.1897	0.0013	0.0061	-1.5755
M1ap	-1.5394	0.1477	-4.189	0.0013	0.0061	-0.7163
D930048N14Rik	-1.4894	1.804	-4.1888	0.0013	0.0061	-0.8583
Glt8d1	0.6357	4.5994	4.1887	0.0013	0.0061	-1.4099
Tmbim4	0.678	4.8791	4.1868	0.0013	0.0061	-1.4457
Osbpl11	0.5053	5.0862	4.1867	0.0013	0.0061	-1.4756
Gm6594	-1.3689	0.1101	-4.1865	0.0013	0.0061	-0.7248
Pfn1	-0.5222	9.6653	-4.1862	0.0013	0.0061	-1.6218
Rab40b	2.344	1.4329	4.1858	0.0013	0.0061	-0.7927
Pknox2	3.8799	-2.1143	4.1855	0.0013	0.0061	-0.8459
Rnf123	0.476	5.1534	4.1849	0.0013	0.0061	-1.4882
Anks1b	-3.1079	-1.482	-4.1838	0.0013	0.0061	-0.8229
Vash2	0.7007	5.1114	4.1829	0.0013	0.0061	-1.4891
Bahcc1	0.5956	6.2185	4.1826	0.0013	0.0061	-1.5686
Fam76a	0.7196	5.1943	4.1819	0.0013	0.0061	-1.4975
Zmat1	1.8414	1.5731	4.1811	0.0013	0.0061	-0.8665
Psmg1	-0.5829	5.066	-4.1811	0.0013	0.0061	-1.4649
Mprip	0.483	8.5832	4.1801	0.0013	0.0061	-1.6262
Gm44067	-1.54	-0.1118	-4.1787	0.0013	0.0061	-0.7237
Tgoln1	0.5326	7.5068	4.1785	0.0013	0.0061	-1.6144
Trub1	-0.8202	3.7297	-4.1785	0.0013	0.0061	-1.2454
Phc3	0.7217	5.8594	4.1763	0.0013	0.0062	-1.5616
Trdmt1	-0.8559	1.8176	-4.1762	0.0013	0.0062	-0.931
Ggact	2.2233	2.5313	4.1758	0.0013	0.0062	-0.9979
Slc12a2	0.9804	7.3047	4.1756	0.0013	0.0062	-1.6164
Rab29	0.8671	2.8306	4.1744	0.0013	0.0062	-1.145
Nfatc1	0.7328	4.8605	4.174	0.0013	0.0062	-1.4558
Tpm1	0.6681	8.8777	4.1739	0.0013	0.0062	-1.6399
Psmc6	-0.5038	7.7913	-4.1733	0.0013	0.0062	-1.6262
Sh3gl3	-1.6886	3.1142	-4.1725	0.0013	0.0062	-1.0862
Zfp954	0.9524	2.9643	4.1722	0.0013	0.0062	-1.149
BC006965	2.113	-1.7454	4.1698	0.0013	0.0062	-0.7657
Accs	0.9577	2.4869	4.1693	0.0013	0.0062	-1.0773
Plekho2	0.6585	6.616	4.1685	0.0013	0.0062	-1.6137
Rnf122	1.8172	3.3767	4.1683	0.0013	0.0062	-1.1887
2810021J22Rik	0.6439	2.8695	4.1676	0.0013	0.0062	-1.1683
Jade1	0.8729	6.6908	4.1674	0.0013	0.0062	-1.6173
Ccdc15	-1.2702	1.6411	-4.1658	0.0013	0.0063	-0.9058
Ptpn5	-4.2838	0.8429	-4.1644	0.0013	0.0063	-0.7506
Abt1	-0.5893	4.9134	-4.1637	0.0013	0.0063	-1.475
AC133601.1	-3.4488	-2.2931	-4.1637	0.0013	0.0063	-0.942
Mmab	0.5917	4.3432	4.163	0.0013	0.0063	-1.4207
2810403D21Rik	1.0346	1.2461	4.161	0.0013	0.0063	-0.9134
Bbc3	1.0271	4.0268	4.1603	0.0013	0.0063	-1.3573
Hspe1-rs1	-1.1847	0.8451	-4.1592	0.0013	0.0063	-0.8439
Gtf2i	0.4921	8.2023	4.1592	0.0013	0.0063	-1.6598
Taf4b	-0.7097	4.7042	-4.1568	0.0013	0.0063	-1.4425

Spg20	0.8769	6.6185	4.1561	0.0013	0.0063	-1.6376
Mcrip2	-1.2572	2.7906	-4.1545	0.0013	0.0064	-1.075
Atf6	-0.4916	6.6045	-4.1541	0.0013	0.0064	-1.6387
Tctn1	0.6498	3.3745	4.1522	0.0014	0.0064	-1.2708
N4bp1	0.6588	6.3242	4.151	0.0014	0.0064	-1.6356
Isoc2a	0.7333	3.8794	4.1508	0.0014	0.0064	-1.3663
Pcgf6	-0.7291	4.9468	-4.1507	0.0014	0.0064	-1.4854
Mrpl51	-0.4461	5.8293	-4.1499	0.0014	0.0064	-1.6068
Gm11914	-1.4552	0.786	-4.149	0.0014	0.0064	-0.8482
Acp7	2.351	-3.4782	4.148	0.0014	0.0064	-0.8955
Gm38158	-3.0509	-2.7734	-4.148	0.0014	0.0064	-0.9608
Nrp2	0.8776	8.8802	4.1472	0.0014	0.0064	-1.6887
Lamtor1	0.3995	6.1955	4.1455	0.0014	0.0064	-1.643
Ndufaf2	-0.5694	4.5009	-4.1453	0.0014	0.0064	-1.4508
Gm42819	3.3538	-2.9671	4.1452	0.0014	0.0064	-0.9763
Ppargc1a	3.5338	-3.3764	4.1447	0.0014	0.0064	-0.975
Cdc42ep1	0.6796	6.6529	4.1438	0.0014	0.0065	-1.6614
Elp1	-0.3542	6.3558	-4.1428	0.0014	0.0065	-1.6483
Lancl1	0.4321	5.0267	4.1426	0.0014	0.0065	-1.5539
0610009L18Rik	1.4869	-0.7507	4.142	0.0014	0.0065	-0.7792
Rb1cc1	0.5607	6.7326	4.1415	0.0014	0.0065	-1.6665
Gm6992	-1.1944	0.3187	-4.1411	0.0014	0.0065	-0.8225
Nav1	0.6527	6.8952	4.1405	0.0014	0.0065	-1.6721
Fech	0.5455	5.6786	4.14	0.0014	0.0065	-1.6205
Thsd7a	2.8817	4.072	4.1399	0.0014	0.0065	-1.2693
Nat8f4	1.9894	-0.3903	4.1372	0.0014	0.0065	-0.7845
Nmral1	-0.8656	4.4125	-4.1364	0.0014	0.0065	-1.4306
Tsc22d4	0.6017	6.4627	4.1363	0.0014	0.0065	-1.6702
Ift74	0.5763	5.023	4.1361	0.0014	0.0065	-1.5547
Mturn	2.1044	2.6223	4.1355	0.0014	0.0065	-1.0789
Gm5853	-2.6536	-1.8687	-4.1338	0.0014	0.0065	-0.8523
Gtf2h4	-0.517	4.6486	-4.1334	0.0014	0.0065	-1.4998
Ncln	-0.53	6.5574	-4.1334	0.0014	0.0065	-1.6713
Slc16a1	-1.4629	8.1048	-4.1334	0.0014	0.0065	-1.6985
Mafb	1.6883	2.8427	4.1321	0.0014	0.0066	-1.1627
Snx25	-0.7168	5.8167	-4.1321	0.0014	0.0066	-1.6273
Mapk1ip1	0.9706	2.3894	4.1311	0.0014	0.0066	-1.122
Gas7	1.5971	7.5669	4.1305	0.0014	0.0066	-1.7008
Gm7390	-1.5444	-0.5564	-4.13	0.0014	0.0066	-0.7913
Prkx	0.5878	5.7364	4.1298	0.0014	0.0066	-1.6416
Gm16098	2.2265	-2.3734	4.1294	0.0014	0.0066	-0.9211
Klhdc2	-0.3657	6.9888	-4.1293	0.0014	0.0066	-1.6939
Mex3c	-0.4801	6.7681	-4.1287	0.0014	0.0066	-1.6864
Txnrd1	-0.5303	8.0268	-4.1285	0.0014	0.0066	-1.7116
Gm47284	-2.779	-3.0516	-4.1284	0.0014	0.0066	-0.9873
Mat2b	0.5329	6.3411	4.1274	0.0014	0.0066	-1.6802
Scpep1	0.7406	7.8636	4.1271	0.0014	0.0066	-1.715
Midn	0.3663	6.6373	4.1268	0.0014	0.0066	-1.6916
Mrps2	-0.5009	5.7415	-4.1268	0.0014	0.0066	-1.6328
Armc9	0.5624	4.1868	4.1255	0.0014	0.0066	-1.4539
Ostc	-0.6112	7.2951	-4.125	0.0014	0.0066	-1.7059
Fuk	0.7665	4.1986	4.1245	0.0014	0.0066	-1.4645

Chmp3	0.3868	6.8867	4.1232	0.0014	0.0066	-1.7046
E230015B07Rik	2.4777	-2.5821	4.1226	0.0014	0.0066	-0.9062
Sclt1	-0.8822	4.3436	-4.1221	0.0014	0.0066	-1.4486
Acsl5	-0.7137	7.1233	-4.1221	0.0014	0.0066	-1.7075
Ticam1	0.8641	4.068	4.1215	0.0014	0.0066	-1.4438
Mss51	-1.821	0.0307	-4.1213	0.0014	0.0066	-0.8186
Eif2b4	-0.4847	5.5166	-4.1211	0.0014	0.0066	-1.6308
Pbx4	2.0834	-0.8404	4.1211	0.0014	0.0066	-0.8056
Rad51c	-0.8362	3.3855	-4.1207	0.0014	0.0067	-1.2815
AC158975.2	1.121	0.7808	4.1198	0.0014	0.0067	-0.9219
Osbpl1a	0.8994	4.2017	4.1189	0.0014	0.0067	-1.4709
Igf2bp2	-0.4352	7.5078	-4.1186	0.0014	0.0067	-1.7221
Lrrc24	2.4749	-2.5067	4.1184	0.0014	0.0067	-0.9115
Sdhaf1	0.6203	3.3885	4.1179	0.0014	0.0067	-1.3322
Nup98	-0.4849	7.2878	-4.1134	0.0014	0.0067	-1.7261
Zfp219	0.8491	4.9001	4.1111	0.0015	0.0068	-1.5923
Thada	-0.5435	5.161	-4.1107	0.0015	0.0068	-1.6003
Dhcr24	-0.5688	7.7287	-4.1103	0.0015	0.0068	-1.7402
Nars2	-0.9936	4.0182	-4.1078	0.0015	0.0068	-1.4085
Hgh1	-0.6329	4.4886	-4.1076	0.0015	0.0068	-1.5019
Enpp5	3.1894	1.2514	4.1076	0.0015	0.0068	-0.8727
Vipr2	3.8807	-3.4181	4.1066	0.0015	0.0068	-1.0349
Atxn7	0.5136	4.287	4.1066	0.0015	0.0068	-1.504
Lrpap1	1.2296	4.421	4.1053	0.0015	0.0068	-1.5152
Dst	0.9201	8.3335	4.105	0.0015	0.0068	-1.76
Hoga1	1.4218	1.3725	4.1049	0.0015	0.0068	-1.017
Zfp62	0.4425	5.338	4.1048	0.0015	0.0068	-1.6539
Fastkd5	-0.7815	3.1204	-4.1045	0.0015	0.0068	-1.2675
4930513N10Rik	1.338	0.3741	4.1035	0.0015	0.0068	-0.8931
Plcg1	0.7179	6.8908	4.1034	0.0015	0.0068	-1.7409
Mtrr	-0.5268	5.3258	-4.1027	0.0015	0.0068	-1.6413
Ttll1	0.6912	3.8871	4.1026	0.0015	0.0068	-1.4544
Nus1	-0.5626	7.0811	-4.1017	0.0015	0.0068	-1.744
Dact2	1.9588	2.0953	4.1015	0.0015	0.0068	-1.0998
Kars	-0.5548	8.2926	-4.1013	0.0015	0.0068	-1.7647
Tiprl	-0.4942	6.1831	-4.1008	0.0015	0.0068	-1.716
Scnm1	0.545	4.1982	4.1001	0.0015	0.0068	-1.522
Oas1c	2.0371	-0.6279	4.0998	0.0015	0.0068	-0.8456
Srsf11	-0.4328	7.6309	-4.0992	0.0015	0.0069	-1.7586
Zfp513	0.48	4.7386	4.099	0.0015	0.0069	-1.5858
Wiz	0.4737	6.3867	4.098	0.0015	0.0069	-1.7353
Tubgcp2	-0.3729	5.5221	-4.097	0.0015	0.0069	-1.6751
Ywhaz	-0.5199	8.8346	-4.0968	0.0015	0.0069	-1.7791
Ube2m	-0.5511	6.9243	-4.0965	0.0015	0.0069	-1.7514
Cry1	-0.4928	4.8523	-4.0961	0.0015	0.0069	-1.5949
Nat2	1.1522	1.6817	4.0959	0.0015	0.0069	-1.0621
Elk1	0.3715	5.6597	4.0951	0.0015	0.0069	-1.7012
Tma16	-0.8634	4.5431	-4.0943	0.0015	0.0069	-1.5269
Zfp119a	0.9846	1.6185	4.0938	0.0015	0.0069	-1.0553
4933427E11Rik	2.5521	-2.9124	4.0931	0.0015	0.0069	-1.0072
Lockd	-0.9681	2.6592	-4.0925	0.0015	0.0069	-1.2137
Ippk	-0.4633	4.6338	-4.092	0.0015	0.0069	-1.5698

Zgpat	0.6534	4.5837	4.0917	0.0015	0.0069	-1.5795
Psmd1	-0.5344	8.4417	-4.0914	0.0015	0.0069	-1.7846
Gm43661	-1.5981	-0.2885	-4.0908	0.0015	0.0069	-0.8625
Prickle1	1.5981	3.4516	4.0896	0.0015	0.0069	-1.3585
Ctif	0.806	5.089	4.0893	0.0015	0.0069	-1.649
Gm11847	-1.3943	-0.4764	-4.0889	0.0015	0.0069	-0.8623
Clec16a	0.4863	5.5221	4.0888	0.0015	0.0069	-1.6979
2210408I21Rik	0.8974	2.4931	4.0886	0.0015	0.0069	-1.204
Gm5855	-0.8551	2.1882	-4.0886	0.0015	0.0069	-1.1619
Mmgt1	-0.5191	6.2391	-4.0877	0.0015	0.007	-1.7387
Kcnj2	3.8756	1.5439	4.0877	0.0015	0.007	-0.9123
Rufy2	0.6068	4.3883	4.0869	0.0015	0.007	-1.552
Eci2	0.737	5.82	4.0868	0.0015	0.007	-1.7283
3010003L21Rik	1.1671	0.5302	4.0868	0.0015	0.007	-0.9376
Gm9118	-1.1077	1.39	-4.0853	0.0015	0.007	-1.0318
Ccdc93	0.5408	6.1205	4.0835	0.0015	0.007	-1.7503
Sf1	-0.4583	8.1125	-4.0819	0.0015	0.007	-1.7979
Cc2d1b	0.5374	5.4342	4.0818	0.0015	0.007	-1.7064
Clba1	0.9554	4.3906	4.0815	0.0015	0.007	-1.5701
Gm7628	2.2952	-3.1718	4.0814	0.0015	0.007	-0.9887
Cbr4	0.6667	3.2219	4.0807	0.0015	0.007	-1.3621
Asah1	0.5393	7.4754	4.0797	0.0015	0.007	-1.7949
Sh3bp5	0.8779	2.4688	4.0775	0.0015	0.0071	-1.2426
Fbrs	0.4362	5.7511	4.0773	0.0015	0.0071	-1.7391
Synj2	-0.4733	6.1101	-4.0772	0.0015	0.0071	-1.7562
Osgep	0.5242	4.3143	4.0771	0.0015	0.0071	-1.5845
Mis12	-0.5221	5.6415	-4.0769	0.0015	0.0071	-1.7159
Epc2	0.4895	5.9831	4.0769	0.0015	0.0071	-1.7528
Gbp3	2.7151	3.6167	4.0757	0.0015	0.0071	-1.3205
Zfp809	0.7517	3.3818	4.0755	0.0016	0.0071	-1.3959
Samd4	-0.5822	5.8597	-4.0742	0.0016	0.0071	-1.7396
Cic	0.6226	7.2591	4.074	0.0016	0.0071	-1.8024
Orm3	3.0846	-2.1077	4.0733	0.0016	0.0071	-0.9724
Idua	1.84	2.2181	4.0732	0.0016	0.0071	-1.1724
Dph3	-0.4893	5.196	-4.0726	0.0016	0.0071	-1.6803
Scmh1	-0.5426	4.4867	-4.0723	0.0016	0.0071	-1.5935
Pum3	-0.703	7.1299	-4.0713	0.0016	0.0071	-1.8002
Cstf1	-0.4736	6.0051	-4.0708	0.0016	0.0071	-1.7568
Cmip	-0.4859	6.0482	-4.0698	0.0016	0.0071	-1.7672
Kcnk5	-1.3872	4.3253	-4.0695	0.0016	0.0071	-1.4952
Gm12592	2.086	-0.0309	4.0681	0.0016	0.0071	-0.9128
Lrrc51	1.6615	1.0893	4.0672	0.0016	0.0072	-1.0064
Slc25a28	-0.6203	4.5	-4.0667	0.0016	0.0072	-1.5877
Katnbl1	-0.6993	5.0504	-4.0666	0.0016	0.0072	-1.6552
Gm4130	-0.7763	2.5661	-4.0665	0.0016	0.0072	-1.2582
Psma5	-0.4061	7.991	-4.066	0.0016	0.0072	-1.8263
Zfp131	-0.4771	6.1807	-4.0657	0.0016	0.0072	-1.776
Capzb	0.5078	7.6621	4.0653	0.0016	0.0072	-1.8244
Heatr5b	0.596	6.0874	4.0627	0.0016	0.0072	-1.7865
Ulk4	-1.0263	1.9781	-4.0626	0.0016	0.0072	-1.1564
Ciao2b	-0.3845	5.2792	-4.062	0.0016	0.0072	-1.7216
Gm5131	1.9341	-2.1221	4.0604	0.0016	0.0072	-0.9324

Mycbp2	0.4543	6.4826	4.0601	0.0016	0.0072	-1.8072
Kat8	0.512	4.1371	4.0598	0.0016	0.0072	-1.5853
Aldh3b1	2.0308	3.0798	4.0592	0.0016	0.0072	-1.2895
Srp68	-0.3639	7.1954	-4.0576	0.0016	0.0072	-1.829
Fsbp	-1.9778	-0.4499	-4.0574	0.0016	0.0072	-0.9083
Hadha	0.445	7.1887	4.0573	0.0016	0.0072	-1.8312
Cryl1	1.6582	-0.4416	4.0567	0.0016	0.0073	-0.926
Ipo9	-0.3816	8.0724	-4.0566	0.0016	0.0073	-1.844
Trappc3	0.4272	5.4383	4.0565	0.0016	0.0073	-1.7543
Gm6169	-1.1804	0.3041	-4.0548	0.0016	0.0073	-0.9614
Tnrc6b	0.7128	6.778	4.0546	0.0016	0.0073	-1.8267
Gbp9	3.9322	2.4435	4.0539	0.0016	0.0073	-1.0745
Dock4	1.1959	5.1952	4.0534	0.0016	0.0073	-1.7176
Glud1	-0.4181	7.9845	-4.0529	0.0016	0.0073	-1.8496
Emc9	0.8156	2.5407	4.0526	0.0016	0.0073	-1.3011
Ncoa2	0.5639	5.9881	4.0524	0.0016	0.0073	-1.7978
Gm17251	1.1429	1.0269	4.0518	0.0016	0.0073	-1.0684
Vsig10	0.9299	2.4966	4.0515	0.0016	0.0073	-1.2847
Slc40a1	4.2948	-1.7671	4.0512	0.0016	0.0073	-1.0624
Ptpn2	-0.6604	6.1112	-4.0512	0.0016	0.0073	-1.796
Golga1	0.3759	5.1957	4.051	0.0016	0.0073	-1.7358
Ctnnb1	0.5919	9.8419	4.0495	0.0016	0.0073	-1.8735
Ddx19b	-0.5285	4.4953	-4.0493	0.0016	0.0073	-1.6199
Gpr161	0.6536	5.0489	4.0493	0.0016	0.0073	-1.7158
Pgrmc1	0.4731	6.6965	4.0485	0.0016	0.0073	-1.8364
Zfp160	0.4608	4.7491	4.0482	0.0016	0.0073	-1.6785
Zmym5	0.4166	4.8916	4.0481	0.0016	0.0073	-1.7011
AI413582	-0.5558	4.6064	-4.0476	0.0016	0.0073	-1.65
Gm47283	-0.9435	4.3379	-4.0472	0.0016	0.0073	-1.5832
Cbx7	0.8197	3.7996	4.0468	0.0016	0.0073	-1.5368
Armc3	3.3596	-1.5917	4.0467	0.0016	0.0073	-0.9936
Coq7	-0.6352	4.2843	-4.0466	0.0016	0.0073	-1.5955
Neu3	1.0799	0.42	4.0466	0.0016	0.0073	-1.0162
Vps9d1	0.7437	3.5462	4.0448	0.0016	0.0074	-1.4799
Rwdd2a	1.1443	0.844	4.0442	0.0016	0.0074	-1.0767
E430024P14Rik	2.0575	-2.0267	4.0439	0.0016	0.0074	-0.9728
Arhgef11	0.4741	5.9948	4.0433	0.0016	0.0074	-1.8172
Gbp7	2.2458	4.0038	4.0425	0.0016	0.0074	-1.4862
Cstb	0.7166	7.2453	4.0423	0.0016	0.0074	-1.8606
Csnk2a1	-0.4555	7.1471	-4.0421	0.0016	0.0074	-1.8553
Uimc1	-0.4616	5.3368	-4.0418	0.0016	0.0074	-1.7601
Plekhg2	0.9463	4.3738	4.0413	0.0016	0.0074	-1.6264
Ufc1	0.365	6.5968	4.0411	0.0016	0.0074	-1.8484
Ablim1	3.5233	0.8259	4.0405	0.0017	0.0074	-0.9659
Cacna1a	1.7229	1.7828	4.0405	0.0017	0.0074	-1.1603
Arpc5	0.3415	7.6271	4.0403	0.0017	0.0074	-1.8689
Gm7327	-1.1118	0.0365	-4.0397	0.0017	0.0074	-0.971
Gm14681	-1.0275	2.3726	-4.0397	0.0017	0.0074	-1.2635
Rbm3	-0.5677	8.1617	-4.0397	0.0017	0.0074	-1.8759
Smarca5	-0.6067	8.2449	-4.0366	0.0017	0.0074	-1.8818
Abat	4.2772	-0.1729	4.0362	0.0017	0.0074	-0.9566
Gm7488	-1.2579	0.2795	-4.0352	0.0017	0.0075	-0.9828

Unc13a	-1.5039	2.8206	-4.0345	0.0017	0.0075	-1.3088
Qtrt2	-0.6951	4.8236	-4.0338	0.0017	0.0075	-1.6862
P4ha1	-0.7466	7.4455	-4.0326	0.0017	0.0075	-1.8774
Sybu	3.5851	1.3477	4.0324	0.0017	0.0075	-1.0125
Slc35d2	0.7127	3.2244	4.0323	0.0017	0.0075	-1.4576
Actn1	0.8533	9.2125	4.0308	0.0017	0.0075	-1.9045
Dync1li2	0.545	6.5373	4.0308	0.0017	0.0075	-1.864
P2rx7	0.9233	3.2653	4.0286	0.0017	0.0075	-1.4435
Akr1e1	0.6609	4.8201	4.0281	0.0017	0.0075	-1.7333
Maea	0.4017	7.1043	4.0277	0.0017	0.0075	-1.8838
Abcc5	0.7284	5.2556	4.0274	0.0017	0.0075	-1.7855
Kptn	0.7282	3.1905	4.0262	0.0017	0.0075	-1.4649
Cnot3	-0.4244	6.2488	-4.0251	0.0017	0.0076	-1.8576
Eif3a	-0.4226	8.9845	-4.025	0.0017	0.0076	-1.9124
Cops4	-0.3851	6.974	-4.0214	0.0017	0.0076	-1.8907
Hspa4	-0.5614	8.9225	-4.0209	0.0017	0.0076	-1.919
Fam229b	1.234	0.6747	4.0199	0.0017	0.0076	-1.0651
Evi2a	-1.583	2.4025	-4.0197	0.0017	0.0076	-1.2627
Mlf2	-0.3699	8.0438	-4.0188	0.0017	0.0076	-1.9134
Zfp821	1.0311	3.7478	4.0185	0.0017	0.0076	-1.5656
Mon1a	0.6493	3.3789	4.0183	0.0017	0.0076	-1.52
Mthfr	0.6267	4.0228	4.0165	0.0017	0.0077	-1.6206
Hist1h1c	1.7125	5.8198	4.0161	0.0017	0.0077	-1.8328
Pgk1	-0.6991	9.4224	-4.0144	0.0017	0.0077	-1.9348
C1ra	4.2536	1.9263	4.0142	0.0017	0.0077	-1.057
Ralgds	0.9854	5.7934	4.0142	0.0017	0.0077	-1.8545
Tssk1	3.5309	-3.3232	4.0137	0.0017	0.0077	-1.1507
Pom121	-0.6048	6.6292	-4.013	0.0017	0.0077	-1.893
Trpm4	1.9099	2.1819	4.0128	0.0017	0.0077	-1.2716
Agtpbp1	-0.5597	5.7797	-4.0119	0.0017	0.0077	-1.8472
Jun	-1.0336	7.119	-4.0118	0.0017	0.0077	-1.906
Rufy1	0.4338	4.9584	4.011	0.0017	0.0077	-1.7848
Gid4	-0.6116	5.7822	-4.011	0.0017	0.0077	-1.8513
Gm2788	-2.3193	0.6819	-4.0102	0.0017	0.0077	-1.0104
Dmwd	-0.7775	5.7006	-4.0101	0.0017	0.0077	-1.8456
Slc35b4	0.3887	5.7186	4.0099	0.0017	0.0077	-1.8603
Rhoq	0.9474	6.4275	4.0089	0.0017	0.0077	-1.8975
March3	2.3388	0.7957	4.0075	0.0018	0.0078	-1.064
mt-Nd2	0.6516	10.0049	4.0046	0.0018	0.0078	-1.9563
L3mbtl2	-0.4489	5.3185	-4.0046	0.0018	0.0078	-1.8188
Adgrg6	2.6235	1.2831	4.0043	0.0018	0.0078	-1.1195
Ivl	-4.2291	-0.6581	-4.0039	0.0018	0.0078	-1.0851
Gm37660	1.0529	0.4397	4.0039	0.0018	0.0078	-1.0679
Gm13293	2.9325	-3.829	4.0039	0.0018	0.0078	-1.1566
Ogn	8.0044	1.4366	4.0024	0.0018	0.0078	-1.0646
Ifnar2	0.8388	5.0452	4.0021	0.0018	0.0078	-1.7957
Prr16	4.4488	-2.9772	4.0004	0.0018	0.0078	-1.1937
1810034E14Rik	2.534	-0.5552	3.9997	0.0018	0.0078	-1.0065
Pop4	-0.6941	3.2662	-3.9994	0.0018	0.0078	-1.4992
Fabp4	-1.8852	3.6018	-3.9993	0.0018	0.0078	-1.4264
Apoo	-0.7041	3.7989	-3.9982	0.0018	0.0079	-1.5917
Chmp4c	2.4588	0.2856	3.9973	0.0018	0.0079	-1.0405

Wwc2	0.58	6.2205	3.9968	0.0018	0.0079	-1.9141
Ralgps1	1.5214	2.7914	3.9957	0.0018	0.0079	-1.4133
Osbpl9	0.6866	6.3326	3.9954	0.0018	0.0079	-1.9231
AC166172.4	1.3729	-0.5813	3.9953	0.0018	0.0079	-1.0361
Nelfcd	-0.4567	5.5122	-3.9945	0.0018	0.0079	-1.8592
Napepld	0.9918	2.9657	3.9941	0.0018	0.0079	-1.4675
Riox1	-0.5851	4.6987	-3.9938	0.0018	0.0079	-1.7503
2510016D11Rik	-1.76	1.3261	-3.9937	0.0018	0.0079	-1.1464
Zfp963	1.1792	1.4017	3.9936	0.0018	0.0079	-1.224
Ch25h	3.1011	-3.1558	3.9934	0.0018	0.0079	-1.1722
Dusp27	3.0971	-1.3854	3.9929	0.0018	0.0079	-1.027
Trio	0.5576	6.5617	3.992	0.0018	0.0079	-1.9354
Deptor	-1.0404	6.1981	-3.9919	0.0018	0.0079	-1.8973
Ambra1	0.3935	5.4387	3.9913	0.0018	0.0079	-1.8723
Mboat1	-1.1341	2.6416	-3.9913	0.0018	0.0079	-1.3831
Mtmr14	0.4777	5.4713	3.9907	0.0018	0.0079	-1.8752
Tomm34	-0.4283	5.4571	-3.9891	0.0018	0.008	-1.868
Naaladl1	-1.6158	-0.3705	-3.9874	0.0018	0.008	-1.021
Parn	0.4181	5.3715	3.9871	0.0018	0.008	-1.8762
Fam168a	0.4227	6.3187	3.9868	0.0018	0.008	-1.9375
Map3k8	0.8397	2.8743	3.9854	0.0018	0.008	-1.4629
Trmt12	0.6121	3.5047	3.9844	0.0018	0.008	-1.5873
Srfbp1	-0.4283	5.2737	-3.9842	0.0018	0.008	-1.8552
Mterf2	0.6619	2.4665	3.9836	0.0018	0.008	-1.4263
Cog5	0.5109	5.1105	3.9832	0.0018	0.008	-1.8482
AC165962.1	1.154	0.783	3.9823	0.0018	0.008	-1.162
Hmg20b	0.5464	5.3461	3.982	0.0018	0.008	-1.8821
Ccdc97	0.4062	5.1615	3.9806	0.0018	0.0081	-1.8616
Naxe	0.7677	5.3467	3.98	0.0018	0.0081	-1.8877
Mknk2	0.953	7.4723	3.98	0.0018	0.0081	-1.9788
Slc4a4	1.7035	4.4315	3.9791	0.0018	0.0081	-1.7043
Fmr1	-0.6237	6.9493	-3.9783	0.0018	0.0081	-1.9654
Cdc27	-0.4254	6.7992	-3.9777	0.0018	0.0081	-1.9646
Nipa2	-0.5148	5.2554	-3.9768	0.0019	0.0081	-1.8527
C030014I23Rik	1.8355	-1.434	3.9764	0.0019	0.0081	-1.0387
Gm10125	3.1745	-0.88	3.9751	0.0019	0.0081	-1.0431
Zfp418	1.9056	-0.1011	3.975	0.0019	0.0081	-1.0679
Snrpert	-0.5437	3.6225	-3.9744	0.0019	0.0081	-1.6099
Mrm3	-0.6296	3.265	-3.9742	0.0019	0.0081	-1.5427
Fhod3	1.511	2.6139	3.974	0.0019	0.0081	-1.4025
Rtca	-0.5029	5.7174	-3.974	0.0019	0.0081	-1.9198
Hectd4	0.9075	5.6877	3.9739	0.0019	0.0081	-1.9132
Dap3	-0.3675	7.2818	-3.9737	0.0019	0.0081	-1.9849
Gm32856	-0.8149	1.8544	-3.9735	0.0019	0.0081	-1.2943
Prpf40a	-0.5238	7.7243	-3.9727	0.0019	0.0081	-1.9922
Rmdn3	-0.4829	5.2515	-3.9719	0.0019	0.0081	-1.873
Gm14137	-1.086	3.9399	-3.9717	0.0019	0.0081	-1.6151
Cacnb1	1.5796	1.0352	3.9713	0.0019	0.0081	-1.219
Slc5a5	2.518	0.3169	3.971	0.0019	0.0081	-1.0867
Armc10	0.5628	5.6098	3.9709	0.0019	0.0081	-1.9253
Gtpbp1	-0.478	6.4035	-3.9705	0.0019	0.0082	-1.9658
Gm43058	3.1091	-2.638	3.9701	0.0019	0.0082	-1.169

Cep290	-0.5555	4.6528	-3.9701	0.0019	0.0082	-1.7824
Zfp266	0.5199	6.184	3.9699	0.0019	0.0082	-1.9592
Card19	-0.6963	5.3025	-3.9696	0.0019	0.0082	-1.8878
Tnfaip8	-0.744	5.2857	-3.9692	0.0019	0.0082	-1.8736
Morc4	-1.3925	5.1185	-3.968	0.0019	0.0082	-1.8037
Hyou1	-0.5143	8.776	-3.9677	0.0019	0.0082	-2.0156
Ddx47	-0.3741	6.7264	-3.9677	0.0019	0.0082	-1.9834
Brap	0.467	6.4565	3.9673	0.0019	0.0082	-1.9786
Mid1	1.6257	2.7435	3.9671	0.0019	0.0082	-1.4256
Mex3b	1.4582	3.0188	3.9667	0.0019	0.0082	-1.4972
Spin1	0.4305	7.1728	3.966	0.0019	0.0082	-1.9972
Gm5475	3.1927	-3.3049	3.9652	0.0019	0.0082	-1.2117
Gm10575	1.2547	0.7906	3.9633	0.0019	0.0082	-1.1999
Trappc6a	0.9945	4.1624	3.9631	0.0019	0.0082	-1.749
Cpsf3	-0.4492	6.5655	-3.9629	0.0019	0.0082	-1.9864
Arhgap29	1.7109	2.6977	3.9626	0.0019	0.0082	-1.4341
Irak1	-0.4907	6.9244	-3.961	0.0019	0.0083	-2.0002
Txk	-3.4447	-0.085	-3.9603	0.0019	0.0083	-1.075
Ndufaf4	-0.7148	4.5254	-3.9599	0.0019	0.0083	-1.7655
Micall2	-0.5135	5.9698	-3.9574	0.0019	0.0083	-1.9677
Gm4366	-0.7651	2.6035	-3.9567	0.0019	0.0083	-1.4656
BC003965	-0.6301	4.4167	-3.9565	0.0019	0.0083	-1.7834
Foxj2	0.737	5.1509	3.9564	0.0019	0.0083	-1.904
Sertad4	1.0893	4.168	3.9563	0.0019	0.0083	-1.7374
Dusp10	0.9521	3.8821	3.9562	0.0019	0.0083	-1.6947
Etf1	-0.613	8.685	-3.956	0.0019	0.0083	-2.0358
Rnf34	0.4287	4.9365	3.9538	0.0019	0.0083	-1.8837
Ptpn6	-1.2935	2.6127	-3.9532	0.0019	0.0083	-1.4255
Gm9833	-1.1434	2.096	-3.9531	0.0019	0.0083	-1.347
Borcs8	0.4676	4.5039	3.9529	0.0019	0.0083	-1.8374
Rnpep	0.4095	5.6963	3.9528	0.0019	0.0083	-1.9644
Rnf38	0.6542	5.3118	3.9515	0.0019	0.0084	-1.9248
Wnt9a	2.5946	0.7819	3.9509	0.0019	0.0084	-1.1531
Zfand3	0.5089	6.5457	3.9496	0.0019	0.0084	-2.0143
Ccdc117	-0.3891	5.647	-3.9488	0.0019	0.0084	-1.9574
Rpl18a	0.4975	9.5504	3.9485	0.0019	0.0084	-2.0581
Tppp3	2.1036	-0.0604	3.9485	0.0019	0.0084	-1.1008
Uchl4	-1.8393	-1.5047	-3.9474	0.002	0.0084	-1.0904
Fam110c	-1.8472	4.2235	-3.9468	0.002	0.0084	-1.6418
Plin2	1.237	6.3068	3.9465	0.002	0.0084	-2.0099
Slc12a6	0.9491	4.7747	3.9465	0.002	0.0084	-1.8612
AI480526	1.1009	1.3179	3.9462	0.002	0.0084	-1.2627
Ubxn4	0.3772	7.4297	3.9457	0.002	0.0084	-2.0401
Kif16b	0.5479	5.3358	3.9453	0.002	0.0084	-1.9477
Gm19680	-0.995	1.1825	-3.9432	0.002	0.0085	-1.259
Pdgfra	1.0342	7.1235	3.9417	0.002	0.0085	-2.0402
Hexdc	0.6713	3.8389	3.9414	0.002	0.0085	-1.7284
Lrrc14b	2.418	0.7439	3.9406	0.002	0.0085	-1.1987
Kank3	2.9242	0.9397	3.9389	0.002	0.0085	-1.1568
Sptlc2	0.4277	6.5858	3.9385	0.002	0.0085	-2.0351
Gm44169	2.8509	-3.45	3.9383	0.002	0.0085	-1.2464
Commd4	0.4558	5.2901	3.9375	0.002	0.0085	-1.9563

Gatad2a	-0.4915	7.371	-3.9372	0.002	0.0085	-2.052
4930451G09Rik	1.6118	0.064	3.9369	0.002	0.0085	-1.147
Fkbp5	0.5794	8.4343	3.9362	0.002	0.0085	-2.0718
Pla2g4c	-2.2282	-0.7886	-3.9361	0.002	0.0085	-1.1073
Dgkq	0.6597	3.9239	3.9338	0.002	0.0086	-1.7624
Junb	0.7776	7.5767	3.9336	0.002	0.0086	-2.0633
2810429I04Rik	-1.7729	1.2314	-3.9329	0.002	0.0086	-1.1959
Fetub	-1.6924	2.9948	-3.9328	0.002	0.0086	-1.4757
Erich2os	-1.5105	-0.679	-3.9326	0.002	0.0086	-1.1167
Aldh3a2	0.7576	6.8349	3.9325	0.002	0.0086	-2.0524
Elovl5	-0.4215	7.6243	-3.9325	0.002	0.0086	-2.0652
Rab21	-0.487	6.5651	-3.9307	0.002	0.0086	-2.0443
Ctbs	-0.6004	4.0735	-3.9307	0.002	0.0086	-1.7817
D10Wsu102e	-0.4444	6.1678	-3.9301	0.002	0.0086	-2.0266
Ehbp1	1.3611	5.0955	3.9291	0.002	0.0086	-1.9292
Usp37	-0.3995	5.6979	-3.9272	0.002	0.0087	-1.9969
2310009A05Rik	0.6831	2.4487	3.9267	0.002	0.0087	-1.5204
Odaph	-2.1153	-0.1741	-3.9267	0.002	0.0087	-1.1238
Ppm1h	1.3535	2.6088	3.9252	0.002	0.0087	-1.5085
Tmem38a	0.7329	3.69	3.925	0.002	0.0087	-1.7235
Kxd1	0.5962	4.6066	3.9247	0.002	0.0087	-1.8967
Mtmr11	-0.6287	4.6951	-3.9234	0.002	0.0087	-1.8901
Gm13387	-5.6759	0.5573	-3.9225	0.002	0.0087	-1.2317
Nfatc4	2.4785	1.1058	3.922	0.002	0.0087	-1.2571
Ctnnd1	0.4973	5.4561	3.9218	0.002	0.0087	-2.0005
Itga10	2.0212	3.2307	3.9207	0.002	0.0087	-1.583
E530011L22Rik	2.7514	-1.4879	3.9202	0.002	0.0087	-1.1456
Ccdc189	0.9012	1.0514	3.9199	0.002	0.0087	-1.3329
Psma6	-0.3767	7.747	-3.9193	0.0021	0.0088	-2.0924
Snhg5	-0.7361	4.9186	-3.9192	0.0021	0.0088	-1.9198
Hsd17b10	-0.4557	6.7338	-3.9192	0.0021	0.0088	-2.0739
Pxylp1	1.0812	3.5932	3.9191	0.0021	0.0088	-1.7038
Tmem91	2.5627	-2.5403	3.9184	0.0021	0.0088	-1.1812
Bcl2l2	-0.6766	4.5836	-3.9183	0.0021	0.0088	-1.8819
Kbtbd3	1.2791	1.6374	3.9181	0.0021	0.0088	-1.3863
Zfp429	0.778	2.3358	3.9177	0.0021	0.0088	-1.5215
Slc7a6os	-0.5321	5.1906	-3.9164	0.0021	0.0088	-1.9649
Aff4	0.5917	8.1786	3.9147	0.0021	0.0088	-2.1083
Tnn	4.5056	-1.1444	3.9143	0.0021	0.0088	-1.2299
Jdp2	0.7777	4.0426	3.9124	0.0021	0.0088	-1.8357
Prps2	-0.5525	6.4543	-3.9115	0.0021	0.0089	-2.0729
Pphln1	-0.4941	6.4499	-3.9107	0.0021	0.0089	-2.0761
Hsp90aa1	-0.586	10.3139	-3.9104	0.0021	0.0089	-2.1309
Slc39a10	-0.6571	6.3325	-3.9103	0.0021	0.0089	-2.0662
Bco2	3.284	-1.233	3.9098	0.0021	0.0089	-1.1661
Slc7a11	-2.5375	6.0563	-3.9097	0.0021	0.0089	-1.9249
Ankrd40	0.3697	6.2537	3.9095	0.0021	0.0089	-2.0784
Prr15l	4.6758	-0.1785	3.909	0.0021	0.0089	-1.1839
Gprasp1	0.7228	4.9459	3.9073	0.0021	0.0089	-1.961
Map1b	-0.5591	7.8374	-3.9069	0.0021	0.0089	-2.1144
Pcsk7	0.3934	5.2058	3.9062	0.0021	0.0089	-2.0046
Edem3	0.46	6.2397	3.9058	0.0021	0.0089	-2.0816

Slc9a3r2	0.5916	3.4529	3.9051	0.0021	0.0089	-1.7215
Sys1	-0.7717	4.5051	-3.905	0.0021	0.0089	-1.8617
Syn3	2.9536	-3.5586	3.9045	0.0021	0.0089	-1.293
Kifc2	1.6173	-0.0029	3.9045	0.0021	0.0089	-1.2034
Clip4	2.3633	2.3322	3.9044	0.0021	0.0089	-1.4261
Fyco1	0.629	4.8016	3.9043	0.0021	0.0089	-1.9595
Calr3	-0.8517	2.56	-3.9037	0.0021	0.0089	-1.5408
Ipo4	-0.6311	5.9726	-3.9036	0.0021	0.0089	-2.064
Tceanc2	0.7263	3.1426	3.9025	0.0021	0.009	-1.6894
Slc25a23	1.6297	4.6737	3.9017	0.0021	0.009	-1.8928
Tns2	1.1005	6.348	3.9014	0.0021	0.009	-2.0915
Aktip	0.66	4.6433	3.901	0.0021	0.009	-1.9412
Padi2	-1.977	4.9254	-3.9005	0.0021	0.009	-1.8675
Gm43909	1.7525	-1.7012	3.8991	0.0021	0.009	-1.1654
Klhdc4	-0.6652	4.7428	-3.899	0.0021	0.009	-1.9291
Sptlc1	0.3647	5.4823	3.8983	0.0021	0.009	-2.0421
Zc3h12c	0.6336	4.6006	3.8974	0.0021	0.009	-1.9248
Pctp	1.0402	2.4352	3.8973	0.0021	0.009	-1.5563
Pianp	2.8349	-3.3158	3.8968	0.0021	0.009	-1.3031
Armc7	0.6592	2.4758	3.8961	0.0021	0.009	-1.5807
Ctdspl2	-0.5254	5.2397	-3.8943	0.0021	0.0091	-2.003
Hmbs	-0.4832	5.5951	-3.8942	0.0021	0.0091	-2.0533
Pusl1	-0.7681	3.7239	-3.8934	0.0022	0.0091	-1.7644
Bud13	-0.5309	4.574	-3.8927	0.0022	0.0091	-1.9205
Pcdhb4	4.0648	-2.9944	3.8921	0.0022	0.0091	-1.3281
AU040320	0.4713	5.6904	3.8917	0.0022	0.0091	-2.0778
Spint1	-2.7064	0.9909	-3.8916	0.0022	0.0091	-1.2025
Gm35190	3.2148	-3.5026	3.8908	0.0022	0.0091	-1.314
Srrm4	0.923	1.5128	3.8906	0.0022	0.0091	-1.4322
Ski	0.5332	6.0317	3.8888	0.0022	0.0091	-2.103
Arl11	-2.0762	-1.0753	-3.8886	0.0022	0.0091	-1.1819
Zfp40	0.8981	1.774	3.8882	0.0022	0.0091	-1.4802
Cdkl3	0.844	1.9807	3.8874	0.0022	0.0091	-1.5215
Fkbp15	0.5008	4.601	3.886	0.0022	0.0092	-1.9667
Slc6a7	3.1412	-3.8644	3.8854	0.0022	0.0092	-1.3213
Gm15655	2.4175	-2.4532	3.8847	0.0022	0.0092	-1.2206
Ezr	-0.851	8.2574	-3.8836	0.0022	0.0092	-2.1638
Slc37a4	-0.5602	4.3615	-3.8835	0.0022	0.0092	-1.9084
4732419C18Rik	2.9628	-1.4703	3.8834	0.0022	0.0092	-1.2069
Smarcal1	0.4554	4.7763	3.8824	0.0022	0.0092	-1.9906
Rps7-ps2	-2.9573	-1.7936	-3.8809	0.0022	0.0092	-1.2555
Edem1	-0.5332	7.0007	-3.8809	0.0022	0.0092	-2.1489
2610021A01Rik	-0.5971	4.1412	-3.8807	0.0022	0.0092	-1.8621
Erap1	0.8407	6.557	3.8805	0.0022	0.0092	-2.1378
Gdnf	1.6091	2.7994	3.88	0.0022	0.0092	-1.5934
Smarcad1	0.41	6.0514	3.8799	0.0022	0.0092	-2.12
Aph1b	2.7272	0.0409	3.8796	0.0022	0.0092	-1.2367
Nbas	0.5427	5.3437	3.8786	0.0022	0.0093	-2.0719
Rps12-ps26	-2.5061	-2.5939	-3.8783	0.0022	0.0093	-1.3209
Ttpal	0.48	5.7114	3.878	0.0022	0.0093	-2.0974
Eif6	-0.4129	7.5982	-3.8769	0.0022	0.0093	-2.1677
Spidr	-0.6574	4.8465	-3.8759	0.0022	0.0093	-1.9917

Prl2c2	-2.1677	4.9818	-3.8753	0.0022	0.0093	-1.8814
Tmem35b	1.3747	1.038	3.8746	0.0022	0.0093	-1.3738
Gm16638	2.8346	-0.6651	3.8712	0.0022	0.0094	-1.206
Abi2	-0.5421	5.9177	-3.8703	0.0022	0.0094	-2.1158
Anapc5	-0.4215	8.1714	-3.8701	0.0022	0.0094	-2.189
Rwdd1	-0.5086	5.8755	-3.87	0.0022	0.0094	-2.1184
Znrf1	0.5863	6.0073	3.8693	0.0022	0.0094	-2.138
Mtss1	0.7599	7.283	3.8691	0.0022	0.0094	-2.1792
Tbck	0.4854	4.6437	3.8678	0.0023	0.0094	-2.0055
Pomk	0.7156	4.6653	3.8671	0.0023	0.0094	-1.9987
Ubfd1	-0.4596	6.3496	-3.8658	0.0023	0.0094	-2.155
Cdyl2	-0.7093	3.7851	-3.8655	0.0023	0.0094	-1.8371
Smtnl2	-1.4014	2.184	-3.8651	0.0023	0.0095	-1.5136
Gnaz	1.7738	-0.1402	3.8644	0.0023	0.0095	-1.2408
Uba6	-0.5284	5.9895	-3.864	0.0023	0.0095	-2.1328
Golga5	0.3462	5.589	3.8628	0.0023	0.0095	-2.122
Sohlh1	4.7116	-1.3215	3.862	0.0023	0.0095	-1.2778
Eef1akmt2	-0.5762	4.0295	-3.8612	0.0023	0.0095	-1.8822
Ehf	3.9173	-1.7483	3.8595	0.0023	0.0095	-1.286
Adk	-0.5836	6.1942	-3.8593	0.0023	0.0095	-2.1601
Gm16754	-1.5506	1.7245	-3.8589	0.0023	0.0095	-1.4324
Nanos1	-1.1575	2.6077	-3.8586	0.0023	0.0095	-1.5829
Cebpa	1.0237	3.7702	3.8586	0.0023	0.0095	-1.8411
Exoc7	0.5405	5.5668	3.8585	0.0023	0.0095	-2.1318
Stx17	0.5258	4.2382	3.8573	0.0023	0.0096	-1.9606
5031434O11Rik	1.421	-0.0646	3.8561	0.0023	0.0096	-1.2957
Depdc7	-1.4187	3.7427	-3.8561	0.0023	0.0096	-1.7674
Fmnl2	-0.5587	7.3325	-3.8555	0.0023	0.0096	-2.2009
Gm9796	2.6369	-2.4032	3.8549	0.0023	0.0096	-1.2936
2010001A14Rik	0.9975	0.9971	3.8542	0.0023	0.0096	-1.4218
AW549877	0.6803	5.7762	3.8531	0.0023	0.0096	-2.1483
Bloc1s1	0.6068	4.063	3.853	0.0023	0.0096	-1.9345
Zfp780b	0.5386	3.2876	3.8525	0.0023	0.0096	-1.7843
Zfp949	0.8817	3.1577	3.8523	0.0023	0.0096	-1.7701
Loxl4	2.8702	5.1818	3.8511	0.0023	0.0096	-2.0204
Ghr	2.1272	2.5596	3.8509	0.0023	0.0096	-1.5779
3110001I22Rik	0.7886	1.8663	3.8497	0.0023	0.0097	-1.5378
Zfp408	0.5501	4.0922	3.8491	0.0023	0.0097	-1.9424
Ccn4	2.1924	2.9033	3.8483	0.0023	0.0097	-1.6373
Exoc3l	1.4426	-0.5269	3.8482	0.0023	0.0097	-1.2623
Rae1	-0.48	6.2484	-3.8474	0.0023	0.0097	-2.1853
Exosc5	-0.6664	4.6603	-3.8473	0.0023	0.0097	-2.015
Rbm17	-0.4619	7.2079	-3.8453	0.0023	0.0097	-2.219
Slc4a2	0.481	6.4386	3.8437	0.0024	0.0097	-2.2057
Cdip1	0.4392	5.0223	3.8432	0.0024	0.0098	-2.0937
Abtb1	1.1852	3.9707	3.8425	0.0024	0.0098	-1.9231
Prr14	0.5311	5.0709	3.8422	0.0024	0.0098	-2.1125
Zfp260	0.4412	5.7746	3.841	0.0024	0.0098	-2.1772
D17H6S53E	0.5418	3.6272	3.8406	0.0024	0.0098	-1.8894
Ctu2	-0.6561	4.5469	-3.8399	0.0024	0.0098	-2.0024
Cenpv	-0.6666	4.0524	-3.8398	0.0024	0.0098	-1.9346
C230062I16Rik	2.1062	-2.2402	3.8395	0.0024	0.0098	-1.2869

Gab3	1.5921	0.2414	3.836	0.0024	0.0099	-1.3517
Kctd20	0.4086	6.5644	3.836	0.0024	0.0099	-2.2234
Setd2	0.487	7.0659	3.8355	0.0024	0.0099	-2.2357
Atxn2l	-0.4097	7.6657	-3.8351	0.0024	0.0099	-2.2457
Rad1	-0.9141	3.9777	-3.8346	0.0024	0.0099	-1.9027
Cdc42ep4	0.6944	6.2253	3.8344	0.0024	0.0099	-2.2127
Gm12435	-1.2257	-0.7464	-3.8341	0.0024	0.0099	-1.2841
Rundc3b	-1.3107	3.3291	-3.8333	0.0024	0.0099	-1.7528
Gm15640	4.2878	-2.5229	3.8329	0.0024	0.0099	-1.3883
Nudt18	0.9859	2.6799	3.8325	0.0024	0.0099	-1.7361
Adgrl3	2.4569	1.2256	3.8324	0.0024	0.0099	-1.3945
Wdr54	-0.6119	2.8754	-3.8298	0.0024	0.0099	-1.7608
Csrp1	1.0149	6.4784	3.8298	0.0024	0.0099	-2.2333
Chd6	0.4917	5.708	3.8287	0.0024	0.01	-2.1939
Mindy4	0.7116	2.9245	3.8285	0.0024	0.01	-1.7635
Prdx6	-0.6305	8.5542	-3.8283	0.0024	0.01	-2.2704
Ccni	0.7572	7.9398	3.8282	0.0024	0.01	-2.2647
Gm12905	0.9403	0.0963	3.8278	0.0024	0.01	-1.3707
Osmr	1.4355	4.3876	3.8274	0.0024	0.01	-1.9895
Ewsr1	-0.4236	8.2996	-3.8274	0.0024	0.01	-2.2693
2010110K18Rik	-2.1976	-0.5984	-3.8266	0.0024	0.01	-1.2776
Zfp455	1.3449	0.5487	3.8258	0.0024	0.01	-1.4157
Strn4	-0.4127	6.514	-3.8251	0.0024	0.01	-2.24
Slain2	-0.409	6.8547	-3.8251	0.0024	0.01	-2.2486
Ackr3	1.1531	5.3278	3.8239	0.0024	0.01	-2.1602
Borcs7	0.734	2.6456	3.8236	0.0024	0.01	-1.741
Ica1	-0.7076	5.4788	-3.8232	0.0024	0.01	-2.1668
Hnrnpc	-0.387	8.5072	-3.8228	0.0024	0.01	-2.2807
Rbm19	-0.4664	5.8683	-3.8227	0.0024	0.01	-2.205
Brms1l	-0.3615	5.0122	-3.8215	0.0024	0.0101	-2.124
Kdm6a	0.5387	5.9568	3.8213	0.0024	0.0101	-2.2211
Uqcc2	-0.4163	6.4359	-3.8211	0.0025	0.0101	-2.2452
Ttn	3.5328	-2.4204	3.8195	0.0025	0.0101	-1.39
Tmem220	1.7206	-0.0347	3.8189	0.0025	0.0101	-1.3564
Agrp	3.2175	-3.1938	3.8184	0.0025	0.0101	-1.4178
Fam172a	0.4764	5.5044	3.8179	0.0025	0.0101	-2.1973
Fbxl3	0.4268	5.6546	3.8172	0.0025	0.0101	-2.2095
Tonsl	-0.7007	4.2837	-3.8159	0.0025	0.0101	-1.995
Sbno2	0.5372	6.0903	3.814	0.0025	0.0102	-2.2399
Cyb5d2	0.6101	2.708	3.8134	0.0025	0.0102	-1.7688
Zfp593	-0.8062	4.3694	-3.8125	0.0025	0.0102	-2.0374
Gm13136	-1.4608	-0.5944	-3.8124	0.0025	0.0102	-1.3211
Carmil1	1.094	4.7226	3.8095	0.0025	0.0102	-2.104
Mlh1	-0.6449	4.5931	-3.8086	0.0025	0.0103	-2.0683
Csnk2a2	-0.4325	6.7368	-3.8074	0.0025	0.0103	-2.2781
4930426L09Rik	2.1524	-2.0244	3.8067	0.0025	0.0103	-1.3307
Lrp10	0.5067	6.7176	3.8066	0.0025	0.0103	-2.283
Zfp706	-0.5349	7.4812	-3.8055	0.0025	0.0103	-2.2969
Lsm7	-0.7874	4.3139	-3.8053	0.0025	0.0103	-2.0285
Kcnma1	3.6668	-2.5518	3.8052	0.0025	0.0103	-1.4271
Ppp2r3c	0.5054	5.0275	3.8047	0.0025	0.0103	-2.171
9530082P21Rik	1.4982	2.4139	3.8047	0.0025	0.0103	-1.6983

Mast2	-0.4741	6.4564	-3.8045	0.0025	0.0103	-2.2725
P4htm	2.8035	-3.0919	3.8045	0.0025	0.0103	-1.4308
Atrn	0.4944	5.6928	3.8041	0.0025	0.0103	-2.2358
Ndufa5	-0.4948	5.2926	-3.8041	0.0025	0.0103	-2.1881
Shisa5	0.4756	6.4424	3.8033	0.0025	0.0103	-2.2807
Nbl1	2.6002	2.575	3.8029	0.0025	0.0103	-1.6461
Chsy1	-0.3797	5.9138	-3.8006	0.0025	0.0104	-2.2518
Ldhb	2.0978	3.2936	3.7997	0.0025	0.0104	-1.8201
Gm23301	-1.6146	-1.3604	-3.7994	0.0025	0.0104	-1.3229
Ggct	-0.8528	2.4495	-3.7986	0.0026	0.0104	-1.7071
Hrct1	2.7692	0.0568	3.7974	0.0026	0.0104	-1.3524
Fam207a	-0.4681	5.4226	-3.7973	0.0026	0.0104	-2.2134
Tubb2b	1.5586	4.0826	3.7965	0.0026	0.0104	-2.0283
Rfx1	-0.435	4.4194	-3.7961	0.0026	0.0104	-2.0799
Pdp1	-0.5818	3.863	-3.795	0.0026	0.0105	-1.9785
Zscan26	0.3769	5.3641	3.7948	0.0026	0.0105	-2.2208
Zfp952	0.6412	2.2671	3.7944	0.0026	0.0105	-1.7233
Ntn4	2.812	0.2433	3.7941	0.0026	0.0105	-1.356
Chid1	0.523	5.1571	3.7938	0.0026	0.0105	-2.2027
Entpd6	-0.4652	4.7268	-3.7937	0.0026	0.0105	-2.1321
Fbrsl1	0.3984	4.9399	3.7931	0.0026	0.0105	-2.1806
F11r	0.8173	4.5418	3.7931	0.0026	0.0105	-2.1189
Nab2	-0.7954	5.1068	-3.7917	0.0026	0.0105	-2.1641
Acadsb	0.659	5.6306	3.7914	0.0026	0.0105	-2.2526
2210408F21Rik	1.0567	1.7969	3.7872	0.0026	0.0106	-1.655
AC132253.9	2.7752	-0.9879	3.7871	0.0026	0.0106	-1.342
Twsg1	0.4663	6.9736	3.7847	0.0026	0.0106	-2.3282
Btnl9	2.4881	-3.1799	3.7845	0.0026	0.0106	-1.4558
B130055M24Ril	-0.9389	2.1855	-3.7832	0.0026	0.0107	-1.6807
Nprl2	0.4857	3.8207	3.7829	0.0026	0.0107	-2.0305
Macc1	2.2719	0.921	3.7827	0.0026	0.0107	-1.4753
Ntmt1	-0.5637	5.3945	-3.7822	0.0026	0.0107	-2.2353
Ampd3	2.0647	5.645	3.7822	0.0026	0.0107	-2.2381
9530068E07Rik	0.4041	8.2236	3.7817	0.0026	0.0107	-2.3544
Sidt2	0.5194	7.0379	3.7817	0.0026	0.0107	-2.3363
Arrdc1	0.6434	3.5504	3.7812	0.0026	0.0107	-1.9708
Pot1b	0.617	3.2593	3.7808	0.0026	0.0107	-1.916
Prmt9	0.5008	4.6713	3.7808	0.0026	0.0107	-2.1678
Mafk	-0.4584	4.6744	-3.7805	0.0026	0.0107	-2.1577
Stim2	-0.356	5.8674	-3.78	0.0026	0.0107	-2.287
Cep41	-0.8185	2.8059	-3.779	0.0026	0.0107	-1.7973
Gm7598	2.3036	-2.8787	3.7789	0.0026	0.0107	-1.3878
Fzd8	0.8507	3.1205	3.7782	0.0026	0.0107	-1.8838
Akr1b8	-0.5697	6.7324	-3.7776	0.0027	0.0107	-2.334
Slc18a1	2.0228	-1.7677	3.7772	0.0027	0.0107	-1.3589
Rest	0.4354	6.4599	3.7769	0.0027	0.0107	-2.3292
Eif2ak1	0.4114	5.9939	3.7769	0.0027	0.0107	-2.3071
Cdc42ep2	2.3572	3.2715	3.7762	0.0027	0.0107	-1.8389
Slc25a11	0.3278	6.2873	3.776	0.0027	0.0107	-2.3272
Cox7a2l	0.5457	6.3537	3.7756	0.0027	0.0107	-2.3291
Rab6a	-0.4998	7.268	-3.7754	0.0027	0.0107	-2.3492
Actr6	-0.5288	3.2535	-3.7754	0.0027	0.0107	-1.8971

Rnase4	2.2276	3.2333	3.7753	0.0027	0.0107	-1.8552
Eefsec	-0.5523	4.1957	-3.775	0.0027	0.0107	-2.0836
Crppa	1.1766	1.5933	3.773	0.0027	0.0108	-1.6298
Zfp472	-0.6799	2.6079	-3.773	0.0027	0.0108	-1.7775
4833419F23Rik	1.7993	-1.1072	3.7726	0.0027	0.0108	-1.3656
Prdx2	0.5754	8.2364	3.7721	0.0027	0.0108	-2.3729
Gm43254	1.5933	-0.105	3.7717	0.0027	0.0108	-1.4183
Miip	-0.7753	3.4851	-3.7708	0.0027	0.0108	-1.9463
Gm49359	0.6942	2.0991	3.7708	0.0027	0.0108	-1.7369
2810455O05Rik	-1.9404	-0.1698	-3.7705	0.0027	0.0108	-1.3842
Cavin2	4.4154	2.3074	3.7703	0.0027	0.0108	-1.5233
Oplah	2.0806	3.4096	3.7699	0.0027	0.0108	-1.8834
Dnm1l	-0.5151	6.9131	-3.7694	0.0027	0.0108	-2.3514
Uckl1	0.7002	3.6485	3.7692	0.0027	0.0108	-2.0202
Irx2	0.3676	6.6805	3.7692	0.0027	0.0108	-2.3516
Avl9	0.7758	5.7452	3.7687	0.0027	0.0108	-2.3022
Tmem218	0.7057	2.6884	3.768	0.0027	0.0108	-1.8252
BB123696	3.1645	-3.4745	3.7674	0.0027	0.0109	-1.4874
Tsc2	0.5102	6.5143	3.766	0.0027	0.0109	-2.351
Fabp3	2.9457	-3.6172	3.7653	0.0027	0.0109	-1.4863
Eif3d	-0.517	8.4348	-3.7641	0.0027	0.0109	-2.3881
Lect2	-3.5334	-1.2216	-3.764	0.0027	0.0109	-1.4401
1110004F10Rik	-0.4804	6.8195	-3.764	0.0027	0.0109	-2.3613
Adamts15	3.186	-3.6197	3.7639	0.0027	0.0109	-1.4899
3110039I08Rik	-2.2748	3.6707	-3.7637	0.0027	0.0109	-1.8305
Hnrnph1	-0.373	8.4094	-3.7636	0.0027	0.0109	-2.3888
Mrtfa	0.4742	5.8152	3.7633	0.0027	0.0109	-2.3226
Gm10822	2.6069	-2.8961	3.7632	0.0027	0.0109	-1.4456
Rmnd5b	0.4485	4.3944	3.7628	0.0027	0.0109	-2.1657
Gpx4-ps2	0.7085	3.1019	3.7622	0.0027	0.0109	-1.9562
Llph	-0.599	4.3327	-3.7622	0.0027	0.0109	-2.1123
Tmsb10	0.825	9.008	3.7615	0.0027	0.0109	-2.4002
Hdac4	-0.555	5.1827	-3.7609	0.0027	0.0109	-2.2528
Gm26902	2.5714	-3.4107	3.7607	0.0027	0.011	-1.4898
Bzw1	-0.4884	9.0182	-3.7603	0.0027	0.011	-2.4009
Usp7	-0.3677	7.7302	-3.7601	0.0027	0.011	-2.3855
Zdhhc4	0.5729	3.9972	3.7592	0.0027	0.011	-2.093
Zfp516	0.7094	6.5217	3.7589	0.0027	0.011	-2.3653
Bola3	-0.6412	4.3166	-3.7585	0.0027	0.011	-2.1336
Pabpn1	-0.613	5.6218	-3.7584	0.0027	0.011	-2.2973
Eif2ak3	0.6305	5.2397	3.7582	0.0027	0.011	-2.2818
Rpl8	-0.537	10.4175	-3.7582	0.0027	0.011	-2.4129
Amer1	0.5706	4.859	3.7577	0.0027	0.011	-2.2292
Txndc16	2.4114	3.2277	3.7571	0.0028	0.011	-1.8513
Espnl	3.0167	-1.4882	3.757	0.0028	0.011	-1.3934
Prr12	0.5008	5.8955	3.7567	0.0028	0.011	-2.3374
Creg1	0.9916	4.7259	3.7564	0.0028	0.011	-2.2011
Krt16	2.8189	-0.6495	3.7563	0.0028	0.011	-1.391
Kdm3a	0.5219	7.2179	3.7561	0.0028	0.011	-2.3873
Wdr61	-0.3388	6.2237	-3.7551	0.0028	0.011	-2.3577
Sufu	0.4663	5.6214	3.755	0.0028	0.011	-2.3187
1110059E24Rik	-0.4786	5.0355	-3.7536	0.0028	0.011	-2.2564

Fn3k	2.9097	-1.2157	3.7532	0.0028	0.0111	-1.3951
Glmn	-0.5931	4.143	-3.753	0.0028	0.0111	-2.092
Dynlt1a	-0.6398	2.9039	-3.7518	0.0028	0.0111	-1.8787
Psmc5	-0.4446	7.5124	-3.7518	0.0028	0.0111	-2.3977
Gm31323	2.453	-3.0609	3.7517	0.0028	0.0111	-1.5017
Tsen15	-0.6713	3.9955	-3.751	0.0028	0.0111	-2.0787
Sfxn2	-1.1869	3.2353	-3.7505	0.0028	0.0111	-1.8989
Tpt1	0.5141	11.1209	3.7501	0.0028	0.0111	-2.4306
Gm24727	-1.9903	-0.9503	-3.7501	0.0028	0.0111	-1.4009
4933440N22Rik	1.388	0.332	3.75	0.0028	0.0111	-1.5183
Foxo3	0.6635	7.1896	3.7497	0.0028	0.0111	-2.3979
Fnbp4	-0.3629	6.3317	-3.7496	0.0028	0.0111	-2.3695
Polr3gl	0.7362	3.4262	3.7487	0.0028	0.0111	-1.9937
Glrx3	-0.451	6.9008	-3.7483	0.0028	0.0111	-2.392
Smad7	-1.3382	4.3442	-3.7477	0.0028	0.0111	-2.0805
Zranb3	-0.6571	4.0049	-3.7476	0.0028	0.0111	-2.0912
Psmc4	-0.4347	6.7629	-3.7466	0.0028	0.0111	-2.3924
Cmas	-0.3717	5.5757	-3.7461	0.0028	0.0112	-2.3309
Angel1	0.9455	2.0565	3.7459	0.0028	0.0112	-1.7748
Psmd10	0.4732	5.8739	3.7451	0.0028	0.0112	-2.364
Cd14	-1.0746	4.4018	-3.7445	0.0028	0.0112	-2.143
Ptma	-0.3867	10.0015	-3.7438	0.0028	0.0112	-2.4376
Ppp3cc	-0.8867	3.6515	-3.7428	0.0028	0.0112	-2.0268
Helz2	0.8173	4.0265	3.7419	0.0028	0.0112	-2.1196
Itgb7	-1.7552	6.0813	-3.7416	0.0028	0.0112	-2.3316
Hist1h3g	-1.8498	-1.1944	-3.7414	0.0028	0.0112	-1.4143
Arpc1b	-0.3941	9.0507	-3.741	0.0028	0.0112	-2.4373
Pde7a	0.7962	6.6545	3.7401	0.0028	0.0112	-2.4037
Gm5814	-1.5678	-1.9428	-3.7399	0.0028	0.0112	-1.4209
Ptov1	0.5649	6.511	3.7394	0.0028	0.0113	-2.4017
Apbb3	0.5049	3.3891	3.7391	0.0028	0.0113	-2.032
Api5	-0.391	7.644	-3.7388	0.0028	0.0113	-2.4228
Foxl1	-1.8002	1.106	-3.7388	0.0028	0.0113	-1.5517
Eng	1.2913	0.9269	3.7378	0.0029	0.0113	-1.6089
Armt1	-0.3973	4.5513	-3.737	0.0029	0.0113	-2.2152
Vdr	2.7037	3.5315	3.7359	0.0029	0.0113	-1.9388
Mbd5	0.5949	4.9097	3.7353	0.0029	0.0113	-2.2857
Mrpl57	-0.5284	4.8016	-3.7346	0.0029	0.0113	-2.2578
Mrfap1	-0.4266	8.4168	-3.7344	0.0029	0.0113	-2.443
Zfp867	0.6674	2.7701	3.7343	0.0029	0.0113	-1.9122
Thumpd2	0.8825	1.5247	3.7341	0.0029	0.0113	-1.7199
Aopep	0.9409	3.7862	3.7336	0.0029	0.0113	-2.1008
Neurl4	-0.4282	5.0492	-3.7326	0.0029	0.0114	-2.2949
Sardh	2.0638	3.3234	3.7322	0.0029	0.0114	-1.9185
Gm19276	-2.7749	-1.8169	-3.732	0.0029	0.0114	-1.4998
Traf2	0.4472	5.4359	3.732	0.0029	0.0114	-2.3491
Map3k20	-0.5712	5.8517	-3.7313	0.0029	0.0114	-2.372
Smc5	-0.5476	6.8426	-3.7313	0.0029	0.0114	-2.4209
Gm7666	-0.9633	1.5072	-3.7302	0.0029	0.0114	-1.6767
Ercc5	0.5499	4.6376	3.73	0.0029	0.0114	-2.2624
Abcc10	0.8718	3.2208	3.7282	0.0029	0.0114	-2.0117
Vezf1	0.3451	6.664	3.727	0.0029	0.0115	-2.428

AC131692.1	-2.47	-0.9974	-3.7265	0.0029	0.0115	-1.4415
Timm10	-0.6005	4.1577	-3.7256	0.0029	0.0115	-2.1516
Pms1	-0.5218	3.7377	-3.7253	0.0029	0.0115	-2.0873
Lsm1	-0.6319	5.4633	-3.725	0.0029	0.0115	-2.3469
Pnkd	0.4866	5.2104	3.725	0.0029	0.0115	-2.341
Mfsd8	0.8537	3.2879	3.7246	0.0029	0.0115	-2.0167
Dennd1b	0.6137	4.4697	3.7243	0.0029	0.0115	-2.2265
Plpp1	0.8576	5.6536	3.7215	0.0029	0.0115	-2.3829
Itprid2	0.6067	5.9997	3.7213	0.0029	0.0116	-2.4115
Ubxn11	1.2839	0.3866	3.7209	0.0029	0.0116	-1.5758
Armcx3	0.8645	3.2186	3.7207	0.0029	0.0116	-2.018
Tmed4	0.4478	7.0934	3.7202	0.0029	0.0116	-2.4517
Bptf	0.4363	7.736	3.7199	0.0029	0.0116	-2.4618
Abhd5	0.9605	6.1562	3.7187	0.003	0.0116	-2.4203
Clip1	0.5956	7.2443	3.7185	0.003	0.0116	-2.4569
Zfp830	0.3677	4.3972	3.7163	0.003	0.0116	-2.2472
Rps6kb1	-0.5175	6.6301	-3.7161	0.003	0.0116	-2.4422
Iqsec1	1.4159	6.3532	3.7155	0.003	0.0117	-2.4321
Hpdl	-2.0525	0.829	-3.7154	0.003	0.0117	-1.5292
2610002M06Rik	0.4502	5.4094	3.7151	0.003	0.0117	-2.375
Snx16	0.9564	3.7414	3.7146	0.003	0.0117	-2.1238
Tra2a	-0.3954	7.3051	-3.7145	0.003	0.0117	-2.4627
Tmem238	-1.276	2.7263	-3.714	0.003	0.0117	-1.8642
Zfp319	0.9995	4.3027	3.7125	0.003	0.0117	-2.2278
Mad2l2	0.4588	4.0633	3.7115	0.003	0.0117	-2.1912
Exoc3l4	-1.6441	3.2991	-3.7113	0.003	0.0117	-1.9142
Gm10602	1.7089	0.1399	3.7111	0.003	0.0117	-1.5277
B3gnt8	1.4854	-1.1556	3.7106	0.003	0.0117	-1.4753
Cep104	-0.4822	5.3999	-3.7104	0.003	0.0117	-2.3771
Gm16793	2.7296	-2.6572	3.7103	0.003	0.0117	-1.562
8030462N17Rik	-0.4162	5.5668	-3.7103	0.003	0.0117	-2.3898
Reep6	-0.7407	2.7987	-3.7103	0.003	0.0117	-1.9252
BC021767	3.8808	-3.4883	3.7103	0.003	0.0117	-1.575
Apba1	2.047	-0.8529	3.7098	0.003	0.0117	-1.4874
Tmem80	1.0032	2.0674	3.7091	0.003	0.0117	-1.837
Cfap300	0.7323	3.4329	3.7086	0.003	0.0117	-2.0877
Plekhm3	0.77	4.434	3.7081	0.003	0.0118	-2.2634
Fam174a	0.7953	3.3707	3.7076	0.003	0.0118	-2.0738
Ola1	-0.4976	7.1963	-3.7073	0.003	0.0118	-2.4737
Car12	-2.3269	2.9312	-3.7066	0.003	0.0118	-1.7877
Larp4b	-0.3881	7.4923	-3.7061	0.003	0.0118	-2.4816
Ascc1	0.5526	4.4835	3.7061	0.003	0.0118	-2.2824
Artn	2.0763	2.6203	3.7058	0.003	0.0118	-1.8776
Arnt	0.4394	7.0044	3.7051	0.003	0.0118	-2.4774
P3h2	1.2196	1.9763	3.7049	0.003	0.0118	-1.7962
Zfp692	-1.1934	3.3384	-3.7047	0.003	0.0118	-1.9959
D230017M19Ril	1.3774	0.3337	3.7038	0.003	0.0118	-1.5754
Gstp1	-0.4883	6.5885	-3.7034	0.003	0.0118	-2.4673
Dync1h1	0.9002	8.865	3.7026	0.003	0.0118	-2.5075
Sco1	-0.4861	4.4344	-3.7026	0.003	0.0118	-2.2561
Rbbp9	0.5051	4.9152	3.7026	0.003	0.0118	-2.3431
Adam5	2.6581	-4.0487	3.7009	0.003	0.0119	-1.5759

Zfp865	0.4211	4.7733	3.7005	0.0031	0.0119	-2.3276
Gbp10	2.3863	-0.9697	3.7003	0.0031	0.0119	-1.4993
Myo10	0.7647	7.4913	3.7003	0.0031	0.0119	-2.495
Tmem216	-0.5548	2.9679	-3.7002	0.0031	0.0119	-1.9789
Gramd3	0.8641	4.7652	3.7001	0.0031	0.0119	-2.3284
Rbm15	-0.4027	5.4858	-3.7001	0.0031	0.0119	-2.3965
Mapre1	-0.3915	7.8702	-3.6998	0.0031	0.0119	-2.499
Pcdhb18	2.4745	-0.1468	3.6996	0.0031	0.0119	-1.506
Kif7	0.7652	2.026	3.6994	0.0031	0.0119	-1.8767
Pyurf	0.5398	3.9213	3.6993	0.0031	0.0119	-2.198
Slc25a33	-0.6566	4.4803	-3.6991	0.0031	0.0119	-2.2555
Tmod3	0.4073	7.2593	3.6989	0.0031	0.0119	-2.4928
Nrp1	1.2843	2.7357	3.6989	0.0031	0.0119	-1.9474
Schip1	-1.568	1.3742	-3.6977	0.0031	0.0119	-1.6945
Il24	-5.1618	0.3567	-3.6975	0.0031	0.0119	-1.5099
Eif4enif1	-0.4032	6.9882	-3.6972	0.0031	0.0119	-2.4885
Gm11613	1.4495	2.2208	3.6957	0.0031	0.0119	-1.87
Elof1	-0.4237	5.6262	-3.6951	0.0031	0.0119	-2.4289
Sfr1	-0.4832	7.5079	-3.6948	0.0031	0.012	-2.503
Zfp276	0.6261	3.9453	3.694	0.0031	0.012	-2.2011
Spop	-0.4673	7.5842	-3.694	0.0031	0.012	-2.5061
Smo	0.4696	6.9088	3.6937	0.0031	0.012	-2.4962
Gm44985	-2.2604	-2.2781	-3.6933	0.0031	0.012	-1.5197
Nvl	-0.4476	6.6931	-3.6926	0.0031	0.012	-2.4886
Rbm26	-0.4975	5.7795	-3.6926	0.0031	0.012	-2.4361
Rac3	1.3748	1.8225	3.6917	0.0031	0.012	-1.7961
Rbm25	-0.3834	7.8153	-3.6913	0.0031	0.012	-2.5144
Adpgk	-0.5417	6.0868	-3.6905	0.0031	0.012	-2.4651
Limk1	-0.7022	5.5838	-3.6896	0.0031	0.012	-2.4219
Pacsin2	-0.5686	7.0347	-3.6893	0.0031	0.012	-2.5036
Gm7002	-1.2895	-0.6866	-3.6887	0.0031	0.0121	-1.5229
Rpl36-ps9	-1.7041	-2.5378	-3.6885	0.0031	0.0121	-1.512
Il7	2.9402	-1.8798	3.6881	0.0031	0.0121	-1.526
Riox2	-0.6521	4.3162	-3.688	0.0031	0.0121	-2.2419
Tmem177	0.6259	3.1514	3.6879	0.0031	0.0121	-2.0805
4833438C02Rik	0.6347	2.9191	3.6875	0.0031	0.0121	-2.0384
Sec1	-1.6036	0.6265	-3.6873	0.0031	0.0121	-1.605
Klhl30	4.4408	0.7743	3.6869	0.0031	0.0121	-1.5274
Fam149a	-0.8679	3.8791	-3.6867	0.0031	0.0121	-2.1627
Abcf1	-0.3578	7.8452	-3.6857	0.0031	0.0121	-2.5253
Gm12153	3.9546	-3.401	3.6854	0.0031	0.0121	-1.6117
Ubl5	0.4515	6.115	3.6851	0.0031	0.0121	-2.4867
Gm15484	-1.7053	0.0402	-3.6837	0.0031	0.0121	-1.5592
Hectd2	-1.8318	1.3436	-3.682	0.0032	0.0122	-1.6739
Leprot	0.7325	5.369	3.6807	0.0032	0.0122	-2.4355
Pqlc3	-0.5523	4.2639	-3.68	0.0032	0.0122	-2.2762
Car7	1.7593	-1.0565	3.6795	0.0032	0.0122	-1.5225
3830406C13Rik	0.7406	3.6191	3.6794	0.0032	0.0122	-2.1634
Slc30a5	-0.5874	6.7215	-3.6782	0.0032	0.0122	-2.5157
Thbs3	1.3169	3.7848	3.678	0.0032	0.0122	-2.1767
C130013H08Rik	0.9282	1.3289	3.6779	0.0032	0.0122	-1.7689
Dusp3	0.7126	5.1994	3.6773	0.0032	0.0123	-2.4322

Serpinb9c	3.3205	-3.2203	3.6771	0.0032	0.0123	-1.6173
Pdcd2l	-0.4688	3.7944	-3.6756	0.0032	0.0123	-2.2128
Traf4	0.6074	6.4569	3.6755	0.0032	0.0123	-2.5184
9430015G10Rik	-0.6507	4.185	-3.6752	0.0032	0.0123	-2.2472
Gm4285	1.0038	0.8698	3.6752	0.0032	0.0123	-1.7024
1810037I17Rik	-0.5768	5.401	-3.6748	0.0032	0.0123	-2.4431
Abhd11	-0.4769	4.5285	-3.6745	0.0032	0.0123	-2.3195
Slc25a3	-0.3243	8.3611	-3.6742	0.0032	0.0123	-2.5545
Vps26a	0.3837	6.6406	3.6734	0.0032	0.0123	-2.5276
Prss56	-2.2221	-3.0548	-3.6732	0.0032	0.0123	-1.6096
Bnip3	-0.6215	6.9646	-3.6719	0.0032	0.0123	-2.5349
Nemf	-0.4211	6.6795	-3.6712	0.0032	0.0124	-2.5293
Cep162	0.5595	4.9374	3.671	0.0032	0.0124	-2.4051
Snrnp27	-0.4213	5.8757	-3.6692	0.0032	0.0124	-2.4984
Cir1	0.5559	4.1187	3.6684	0.0032	0.0124	-2.2827
Gpd2	-0.7073	5.9831	-3.668	0.0032	0.0124	-2.4962
Fggy	0.8024	2.9267	3.6674	0.0032	0.0124	-2.0725
Rras	0.8395	5.2494	3.6667	0.0032	0.0124	-2.4577
Fads3	0.7122	6.0989	3.6661	0.0032	0.0125	-2.5187
Dclre1c	0.5796	3.34	3.6651	0.0033	0.0125	-2.1375
BC048644	-2.4084	-2.3854	-3.6651	0.0033	0.0125	-1.5862
Sec22a	0.4565	3.8299	3.6647	0.0033	0.0125	-2.2447
Ints5	-0.4139	5.9749	-3.6642	0.0033	0.0125	-2.5111
Cops2	-0.5646	7.8664	-3.6621	0.0033	0.0125	-2.5679
Orc4	-0.586	5.666	-3.662	0.0033	0.0125	-2.481
Hsf1	-0.51	5.8475	-3.6619	0.0033	0.0125	-2.5011
Trim25	0.4898	6.6194	3.6612	0.0033	0.0125	-2.5487
1190005I06Rik	2.1634	0.677	3.6609	0.0033	0.0125	-1.6651
Rab5if	-0.4587	6.8828	-3.6607	0.0033	0.0125	-2.5547
Eif2b5	-0.3322	6.6529	-3.6605	0.0033	0.0125	-2.5501
Yars2	-0.5817	4.8922	-3.66	0.0033	0.0126	-2.3942
Toe1	-0.4566	4.4573	-3.6586	0.0033	0.0126	-2.3366
Gmpr2	-0.3374	4.9128	-3.6585	0.0033	0.0126	-2.4179
Tmco3	0.6673	5.0702	3.6583	0.0033	0.0126	-2.4508
Pdgfb	2.6242	-1.1718	3.658	0.0033	0.0126	-1.5473
Piezo2	-4.1225	0.9447	-3.6577	0.0033	0.0126	-1.5485
1810043G02Rik	0.6175	3.268	3.6575	0.0033	0.0126	-2.1466
Rnf31	0.4903	4.3246	3.6575	0.0033	0.0126	-2.334
Rbl2	0.6111	5.3527	3.6564	0.0033	0.0126	-2.481
Phyhd1	1.4591	0.2386	3.6561	0.0033	0.0126	-1.6756
Nr1h3	2.0635	1.1732	3.6557	0.0033	0.0126	-1.7065
Suox	1.7664	3.2352	3.6548	0.0033	0.0126	-2.101
Pcdhgb2	2.5543	-1.7346	3.654	0.0033	0.0127	-1.5683
Chpf2	0.3771	5.9122	3.6539	0.0033	0.0127	-2.5309
Rbm47	2.1499	0.2584	3.6534	0.0033	0.0127	-1.6277
Rnf121	-0.4963	4.6651	-3.651	0.0033	0.0127	-2.3821
Srbd1	-0.4418	5.417	-3.651	0.0033	0.0127	-2.483
Cul9	2.6493	-2.6204	3.6498	0.0033	0.0127	-1.632
Nudt3	-0.4192	6.6427	-3.6493	0.0034	0.0128	-2.5687
Pex19	-0.3276	6.3447	-3.6491	0.0034	0.0128	-2.5598
Slc35a1	-0.4658	5.1176	-3.6479	0.0034	0.0128	-2.4514
Micu1	0.4815	5.547	3.6479	0.0034	0.0128	-2.5192

Recql	-0.5032	4.2325	-3.6476	0.0034	0.0128	-2.3099
Osr1	1.9284	4.869	3.6474	0.0034	0.0128	-2.3999
Xdh	0.8488	7.6939	3.6454	0.0034	0.0128	-2.5997
Brdt	0.9448	1.8261	3.6453	0.0034	0.0128	-1.8918
Lpp	0.8917	6.4583	3.6451	0.0034	0.0128	-2.5698
Dnah17	2.5374	-1.5015	3.6447	0.0034	0.0128	-1.5819
Siglec15	-2.9093	0.3276	-3.6445	0.0034	0.0128	-1.5894
Psmd2	-0.373	8.9288	-3.6439	0.0034	0.0128	-2.6165
Mycl	2.9121	-0.3121	3.6436	0.0034	0.0129	-1.5783
Gm43379	1.9963	-1.7526	3.6435	0.0034	0.0129	-1.5846
Ttc38	0.8715	2.8538	3.6434	0.0034	0.0129	-2.0959
Slc12a7	0.8297	6.1593	3.6433	0.0034	0.0129	-2.5615
Saal1	-0.41	4.4923	-3.6419	0.0034	0.0129	-2.3803
Zfp438	0.7271	2.4132	3.6416	0.0034	0.0129	-2.0334
Zkscan6	0.4657	3.6255	3.6407	0.0034	0.0129	-2.2472
A430103D13Rik	-0.9382	0.9639	-3.6404	0.0034	0.0129	-1.7598
Morn5	3.2336	-2.2728	3.6402	0.0034	0.0129	-1.6071
Mknk1	0.4555	4.158	3.64	0.0034	0.0129	-2.351
Gm16589	-0.7328	1.9992	-3.64	0.0034	0.0129	-1.9272
Glipr1	1.1494	4.7515	3.6392	0.0034	0.0129	-2.4214
Rnf4	-0.4613	7.4323	-3.6387	0.0034	0.0129	-2.6056
Rassf7	0.985	3.3448	3.6386	0.0034	0.0129	-2.1974
Gfer	-0.4183	4.8932	-3.6386	0.0034	0.0129	-2.4462
Gm11407	1.7635	-2.0006	3.6378	0.0034	0.0129	-1.5825
Mrc1	-3.3509	-0.5241	-3.6377	0.0034	0.0129	-1.5859
Agpat4	0.4738	5.2791	3.6354	0.0034	0.013	-2.511
Shc4	-0.7667	1.9158	-3.635	0.0034	0.013	-1.9224
Slc25a13	-0.7564	6.0739	-3.6346	0.0034	0.013	-2.5612
Hacl1	0.9602	1.568	3.6341	0.0034	0.013	-1.9068
Wdr11	0.4026	5.1497	3.6338	0.0034	0.013	-2.5067
Sdf4	0.4124	7.0392	3.633	0.0035	0.013	-2.6125
Pcdh12	3.1596	-2.8068	3.633	0.0035	0.013	-1.6409
Pla2g15	0.5128	4.2445	3.6329	0.0035	0.013	-2.3761
Tpcn1	-0.9525	6.9768	-3.6315	0.0035	0.0131	-2.6067
Zfp607b	1.1	0.7792	3.6308	0.0035	0.0131	-1.7714
Zfp64	-0.4507	5.2386	-3.6307	0.0035	0.0131	-2.5043
Tasp1	-0.7101	3.8037	-3.6293	0.0035	0.0131	-2.2679
Gm15920	-0.697	3.5699	-3.629	0.0035	0.0131	-2.2338
Asap3	1.6184	0.9921	3.6289	0.0035	0.0131	-1.7851
Gm26910	1.5205	-0.7178	3.6282	0.0035	0.0131	-1.6126
Gm7435	0.9808	0.7285	3.6281	0.0035	0.0131	-1.783
Krt18	-0.9958	3.6476	-3.6276	0.0035	0.0131	-2.2312
Agpat1	-0.4017	6.346	-3.6274	0.0035	0.0131	-2.6
Gnb1l	-0.5739	2.7829	-3.6266	0.0035	0.0132	-2.0932
Triap1	-0.3992	4.0883	-3.6262	0.0035	0.0132	-2.3452
Mapk3	0.9205	6.523	3.6259	0.0035	0.0132	-2.6135
Gm36696	-3.9382	-0.0322	-3.6256	0.0035	0.0132	-1.6038
Spata5	-0.7522	5.5265	-3.6241	0.0035	0.0132	-2.5324
Zmiz2	0.4883	6.9145	3.6239	0.0035	0.0132	-2.6262
Tbc1d14	0.7684	5.8152	3.6237	0.0035	0.0132	-2.5774
Gm10252	1.0737	0.6612	3.6237	0.0035	0.0132	-1.7843
Gm6563	-0.7132	2.0829	-3.6234	0.0035	0.0132	-1.9733

Stard5	1.0387	2.3576	3.6228	0.0035	0.0132	-2.033
Tm7sf3	0.39	5.2878	3.6227	0.0035	0.0132	-2.5432
Gm47204	1.2648	0.5207	3.6222	0.0035	0.0132	-1.773
Dnajc14	0.3851	6.1464	3.622	0.0035	0.0132	-2.6056
Kdm7a	-0.5214	6.1293	-3.6216	0.0035	0.0132	-2.5969
Nckap1	-0.455	7.6842	-3.6214	0.0035	0.0132	-2.6419
Nhsl2	4.0113	-1.4236	3.6211	0.0035	0.0132	-1.6476
Elf2	0.4245	6.9344	3.621	0.0035	0.0132	-2.6316
Smyd2	-0.5486	6.5593	-3.6207	0.0035	0.0133	-2.6179
Arid4b	0.3927	6.539	3.6206	0.0035	0.0133	-2.6221
Kdm4c	0.4607	4.4699	3.6202	0.0035	0.0133	-2.4312
Cdh13	1.0696	6.5947	3.6194	0.0035	0.0133	-2.6237
Cdsn	5.1886	1.8612	3.6193	0.0035	0.0133	-1.7328
Mfhas1	1.0058	4.7246	3.6182	0.0035	0.0133	-2.4609
Fam92b	2.5463	-3.7654	3.6177	0.0036	0.0133	-1.6912
Plec	-0.4006	9.4594	-3.6177	0.0036	0.0133	-2.6689
Xxylt1	-0.5138	4.9779	-3.6173	0.0036	0.0133	-2.488
Ap1s3	-1.1271	2.5288	-3.617	0.0036	0.0133	-2.0127
Ifit1bl2	2.7308	-1.7534	3.6169	0.0036	0.0133	-1.6167
Spsb1	0.7919	5.1521	3.6161	0.0036	0.0133	-2.52
Ift52	0.4463	5.1596	3.6153	0.0036	0.0133	-2.5416
2310068J16Rik	1.7777	-1.141	3.6148	0.0036	0.0134	-1.6244
Gm46432	-0.9113	1.6298	-3.6145	0.0036	0.0134	-1.9097
mt-Nd1	0.4894	11.1774	3.6144	0.0036	0.0134	-2.6823
Tmub1	0.798	2.9465	3.6143	0.0036	0.0134	-2.1781
Nipsnap3b	0.5058	3.8857	3.6139	0.0036	0.0134	-2.3457
Tprn	1.4755	4.6996	3.6136	0.0036	0.0134	-2.4549
Lgr6	-4.7347	2.9608	-3.6136	0.0036	0.0134	-1.6889
Snora69	-2.9293	-3.1766	-3.6134	0.0036	0.0134	-1.6995
2610017A05Rik	-1.9631	-1.1181	-3.6131	0.0036	0.0134	-1.6196
Pde4dip	0.4553	6.3157	3.6126	0.0036	0.0134	-2.6322
Taf1d	-0.7971	6.775	-3.6116	0.0036	0.0134	-2.6384
Scoc	-0.6134	5.5765	-3.6114	0.0036	0.0134	-2.5708
Btg2	1.3519	3.5417	3.6113	0.0036	0.0134	-2.2453
1700001L19Rik	2.1915	-2.2352	3.6096	0.0036	0.0134	-1.6291
Atf5	-0.4192	5.6297	-3.6092	0.0036	0.0135	-2.5874
Zfp60	0.4473	4.1689	3.6091	0.0036	0.0135	-2.3972
Ttyh2	0.5312	5.141	3.609	0.0036	0.0135	-2.531
1700016L21Rik	2.6638	-3.8552	3.6087	0.0036	0.0135	-1.7062
Chmp2b	0.6473	5.877	3.6087	0.0036	0.0135	-2.6129
2700038G22Rik	-1.2423	2.2738	-3.6075	0.0036	0.0135	-1.968
E130208F15Rik	2.7773	-1.7784	3.6073	0.0036	0.0135	-1.6371
Tmem70	-0.5133	5.3116	-3.6072	0.0036	0.0135	-2.5497
Med16	0.48	4.3247	3.6059	0.0036	0.0135	-2.4287
Erlin1	-0.5666	6.5569	-3.6056	0.0036	0.0135	-2.6434
Kat7	-0.3261	7.1982	-3.6034	0.0036	0.0136	-2.6675
Kif17	-1.2453	-0.1265	-3.6029	0.0036	0.0136	-1.6913
Gm8116	-0.6517	3.275	-3.6023	0.0037	0.0136	-2.211
Retreg1	1.0824	4.197	3.6022	0.0037	0.0136	-2.4129
Atl3	0.6996	7.537	3.6022	0.0037	0.0136	-2.6776
Bcl2	-1.2506	3.6968	-3.6011	0.0037	0.0136	-2.2203
Rapgefl1	0.7264	1.5534	3.6003	0.0037	0.0136	-1.9683

Cnpy2	0.6712	5.5657	3.5996	0.0037	0.0136	-2.6051
Zhx2	0.717	3.6426	3.5995	0.0037	0.0136	-2.3288
Gm13091	2.4946	-2.5778	3.5982	0.0037	0.0137	-1.6858
Tubb4b-ps2	-2.0036	-1.9239	-3.5978	0.0037	0.0137	-1.6472
Polr2b	-0.3693	7.7475	-3.5975	0.0037	0.0137	-2.6876
Higd1a	-0.541	5.7687	-3.5974	0.0037	0.0137	-2.6186
Hnrnpm	-0.2954	8.689	-3.5966	0.0037	0.0137	-2.7022
Gfra1	3.7493	-1.1163	3.5965	0.0037	0.0137	-1.6539
Gm45030	-2.7156	-2.8211	-3.5957	0.0037	0.0137	-1.7199
Herc4	-0.3996	5.764	-3.5955	0.0037	0.0137	-2.6196
Gm12743	2.5179	-1.5009	3.5952	0.0037	0.0137	-1.6476
Prkrip1	-0.4799	4.5121	-3.5936	0.0037	0.0138	-2.4739
Mybpc2	4.4532	-2.8909	3.5933	0.0037	0.0138	-1.7507
Acot6	2.1375	-1.2913	3.5931	0.0037	0.0138	-1.6592
Dnttip2	-0.3775	7.6732	-3.5929	0.0037	0.0138	-2.6948
Sun2	0.6957	6.8474	3.5923	0.0037	0.0138	-2.6836
Cav1	0.7927	9.4534	3.5915	0.0037	0.0138	-2.7179
Hipk3	0.5502	6.2696	3.5911	0.0037	0.0138	-2.6644
Rb1	1.4858	4.8843	3.5907	0.0037	0.0138	-2.5135
Gm17586	0.8933	2.9181	3.5893	0.0037	0.0138	-2.211
Gm14857	-2.9746	-2.1687	-3.5887	0.0037	0.0139	-1.7016
Spast	0.3906	6.0486	3.587	0.0038	0.0139	-2.663
Trmt2b	0.4098	4.6174	3.5869	0.0038	0.0139	-2.519
Atl2	-0.541	6.154	-3.5868	0.0038	0.0139	-2.6596
Map3k5	-1.6532	5.3695	-3.5867	0.0038	0.0139	-2.5142
Gfra4	-2.1922	0.6602	-3.5865	0.0038	0.0139	-1.722
Myh9	0.6505	9.8442	3.5864	0.0038	0.0139	-2.7297
Snx13	0.5064	5.947	3.5862	0.0038	0.0139	-2.6579
Rheb	-0.3778	6.7784	-3.5859	0.0038	0.0139	-2.691
Gm10524	0.989	1.7353	3.5857	0.0038	0.0139	-2.0056
Gm9776	0.6895	1.5568	3.5857	0.0038	0.0139	-1.9819
Map4k3	0.6355	4.9672	3.5851	0.0038	0.0139	-2.5657
Aifm1	-0.3893	6.8715	-3.585	0.0038	0.0139	-2.6949
Tom1l2	0.4351	6.4012	3.5833	0.0038	0.014	-2.6879
Edf1	0.4016	7.0273	3.5831	0.0038	0.014	-2.7049
Erbb3	1.9061	-0.0061	3.5829	0.0038	0.014	-1.7531
Mrps6	-0.6183	5.8154	-3.5812	0.0038	0.014	-2.6491
Zfp65	0.7854	4.0584	3.581	0.0038	0.014	-2.4318
Harbi1	0.7042	3.1824	3.581	0.0038	0.014	-2.2686
Mettl1	-1.3108	3.4824	-3.5806	0.0038	0.014	-2.2463
Pglyrp2	3.67	-3.5047	3.5795	0.0038	0.014	-1.7553
Ranbp3	-0.3479	6.8995	-3.5793	0.0038	0.014	-2.7055
Cryzl1	-0.3843	5.4566	-3.5792	0.0038	0.014	-2.6324
Mmp1a	-4.1362	-2.1062	-3.5788	0.0038	0.014	-1.7593
Prrx2	1.9817	3.1786	3.5788	0.0038	0.014	-2.2102
Cul1	-0.3267	7.5522	-3.5779	0.0038	0.0141	-2.7208
Myh15	2.5768	-3.8753	3.5779	0.0038	0.0141	-1.75
Matn2	1.9255	5.0081	3.5778	0.0038	0.0141	-2.544
Mtx1	-0.3644	5.2613	-3.577	0.0038	0.0141	-2.6093
Immt	-0.412	8.0713	-3.577	0.0038	0.0141	-2.7307
Gm45592	1.7896	-2.0701	3.5756	0.0038	0.0141	-1.6853
Tmem8b	2.1987	0.6652	3.5752	0.0038	0.0141	-1.8227

Dnmt3a	0.7807	5.7824	3.5751	0.0038	0.0141	-2.6641
Veph1	3.4264	-3.0576	3.5744	0.0038	0.0141	-1.7628
Alg8	-0.8426	4.1186	-3.5743	0.0038	0.0141	-2.4076
Gdf5	3.2089	-3.2118	3.5728	0.0039	0.0142	-1.7593
Gfpt1	-0.4631	7.2357	-3.5723	0.0039	0.0142	-2.7257
Ncdn	-0.449	5.158	-3.5719	0.0039	0.0142	-2.5964
Gp5	2.8673	-2.4788	3.5715	0.0039	0.0142	-1.7226
Ripk1	0.4594	6.2404	3.5714	0.0039	0.0142	-2.7019
Wtip	0.7148	3.3885	3.5711	0.0039	0.0142	-2.3161
Cdc23	-0.5196	6.4073	-3.5707	0.0039	0.0142	-2.7055
Naa38	-0.5107	4.7938	-3.5706	0.0039	0.0142	-2.5612
Apcdd1	-1.284	4.5754	-3.5704	0.0039	0.0142	-2.4555
Gm5525	-1.2321	-0.3009	-3.5695	0.0039	0.0142	-1.7468
Fam117b	-0.3338	6.363	-3.5693	0.0039	0.0142	-2.7076
Mapkbp1	0.5833	4.2302	3.5669	0.0039	0.0143	-2.4918
Wipi1	0.7441	6.5233	3.565	0.0039	0.0143	-2.7258
Apip	0.6941	3.5333	3.5649	0.0039	0.0143	-2.3884
Eif5b	-0.3525	8.3054	-3.5627	0.0039	0.0144	-2.7606
Asxl1	0.3861	6.6771	3.5626	0.0039	0.0144	-2.7337
Nr2f6	0.3237	6.0417	3.5625	0.0039	0.0144	-2.7081
Hax1	-0.4287	6.4792	-3.5625	0.0039	0.0144	-2.7276
Ccnh	-0.5353	5.6272	-3.5613	0.0039	0.0144	-2.6681
Styx	-0.4853	3.9071	-3.5605	0.0039	0.0144	-2.4192
Ing4	0.7698	1.1717	3.5604	0.0039	0.0144	-1.9772
Aldh1l2	-1.0161	5.3863	-3.5599	0.0039	0.0144	-2.6445
Csnk1g2	-0.3769	6.9898	-3.559	0.004	0.0145	-2.746
Flnb	0.7933	8.6638	3.5585	0.004	0.0145	-2.7735
Mrpl54	-0.4395	4.7005	-3.5584	0.004	0.0145	-2.5714
Plxnd1	1.3947	5.5384	3.5582	0.004	0.0145	-2.6567
Ift43	0.5893	4.2651	3.5578	0.004	0.0145	-2.5236
Itga11	3.3128	-3.409	3.5567	0.004	0.0145	-1.7833
Tmem9	0.4665	5.7137	3.5555	0.004	0.0145	-2.7025
Eif5al3-ps	-1.2359	0.7791	-3.5551	0.004	0.0145	-1.8719
Olfml3	1.975	4.0602	3.5549	0.004	0.0145	-2.4249
Ing2	-0.4801	4.6176	-3.5545	0.004	0.0145	-2.5564
Aldh4a1	0.9005	3.3742	3.5536	0.004	0.0146	-2.3371
Cacna1i	2.9628	-2.5429	3.5531	0.004	0.0146	-1.7428
Rpe	-0.4555	5.4603	-3.5517	0.004	0.0146	-2.6661
Myl4	1.4098	-0.939	3.5509	0.004	0.0146	-1.7493
Paip2b	-0.3732	6.2143	-3.5508	0.004	0.0146	-2.736
Rab11fip5	-0.4593	6.0342	-3.5495	0.004	0.0147	-2.7282
Zfp628	0.5039	3.8999	3.5493	0.004	0.0147	-2.4486
Pkp4	-0.5473	6.5172	-3.5486	0.004	0.0147	-2.749
Pdcd7	-0.4284	4.648	-3.5482	0.004	0.0147	-2.5801
Ikbke	1.0957	3.8783	3.5481	0.004	0.0147	-2.4652
A630089N07Rik	-1.0054	2.0332	-3.5466	0.004	0.0147	-2.0848
Bend6	-1.1261	4.0606	-3.5458	0.0041	0.0147	-2.4316
4430402I18Rik	2.9302	-3.745	3.5458	0.0041	0.0147	-1.7964
A230028O05Rik	-2.9242	-1.0258	-3.5445	0.0041	0.0148	-1.7328
Atp8b2	-0.4558	7.493	-3.5443	0.0041	0.0148	-2.7823
Qsox1	0.5304	6.4227	3.5436	0.0041	0.0148	-2.7606
Otulinl	2.9317	0.4366	3.5431	0.0041	0.0148	-1.7925

Gm32849	-3.8552	-2.8857	-3.5431	0.0041	0.0148	-1.8052
Lhx6	-0.7244	2.4628	-3.5422	0.0041	0.0148	-2.1709
Rer1	-0.3948	6.3783	-3.5412	0.0041	0.0148	-2.7602
Gpbp1	-0.5026	7.085	-3.5404	0.0041	0.0149	-2.7806
Snd1	-0.3506	8.5487	-3.54	0.0041	0.0149	-2.8061
Nmb	2.2605	-1.8624	3.5397	0.0041	0.0149	-1.752
Maff	-1.0326	4.9681	-3.5397	0.0041	0.0149	-2.6141
Slc6a9	0.6175	4.9931	3.5381	0.0041	0.0149	-2.6614
Ing1	-0.408	6.1377	-3.5379	0.0041	0.0149	-2.7552
Herpud1	0.9875	5.7999	3.5377	0.0041	0.0149	-2.7419
Gm27029	-1.3544	-0.8502	-3.5375	0.0041	0.0149	-1.7707
Atp5g3	-0.4693	7.867	-3.5374	0.0041	0.0149	-2.8008
Pcdhb7	2.2284	-0.5355	3.5371	0.0041	0.0149	-1.7658
Rgs12	1.5977	4.3448	3.5355	0.0041	0.015	-2.5322
Amigo3	0.8075	0.4171	3.5348	0.0041	0.015	-1.9211
Rbpms2	-0.948	5.1826	-3.5345	0.0041	0.015	-2.659
Gm15387	-2.1629	-3.0106	-3.5344	0.0041	0.015	-1.8077
Alg9	-0.4091	5.0929	-3.5338	0.0041	0.015	-2.6705
Frmd6	0.4527	6.6088	3.5327	0.0041	0.015	-2.7865
Gm16538	-2.4202	-2.5972	-3.5323	0.0042	0.015	-1.7817
S1pr1	2.5869	1.9751	3.5321	0.0042	0.015	-2.0166
Atmin	0.4751	4.899	3.5315	0.0042	0.0151	-2.6562
Muc1	2.7931	-1.0497	3.5312	0.0042	0.0151	-1.7497
F3	-1.1618	3.8772	-3.5306	0.0042	0.0151	-2.4089
Ddx60	2.7633	-0.2607	3.5301	0.0042	0.0151	-1.7726
Ift88	0.5619	3.0969	3.5298	0.0042	0.0151	-2.3543
Anxa6	1.0916	6.4713	3.5297	0.0042	0.0151	-2.786
Manf	-0.5071	7.4625	-3.5296	0.0042	0.0151	-2.8093
Ttc8	-0.4803	4.7397	-3.5296	0.0042	0.0151	-2.6232
A930007I19Rik	1.2783	-0.3262	3.5292	0.0042	0.0151	-1.8331
Vcl	0.807	8.1372	3.5291	0.0042	0.0151	-2.8226
Cgnl1	0.5736	6.2647	3.5289	0.0042	0.0151	-2.7815
Pstk	-0.5696	3.965	-3.5288	0.0042	0.0151	-2.4949
Ttc5	0.5694	4.3791	3.5283	0.0042	0.0151	-2.5885
Ccdc91	0.5979	4.2671	3.5277	0.0042	0.0151	-2.5662
Tbc1d13	0.2956	6.0141	3.5276	0.0042	0.0151	-2.7741
Ttll4	-0.4694	6.3757	-3.526	0.0042	0.0152	-2.7839
Insr	0.4561	5.7703	3.5251	0.0042	0.0152	-2.7559
Brpf1	-0.3906	5.8661	-3.525	0.0042	0.0152	-2.7594
Zfp207	-0.3111	7.461	-3.5236	0.0042	0.0152	-2.8204
Gm3608	-0.462	5.8599	-3.5233	0.0042	0.0152	-2.7583
Timm29	0.3926	5.9364	3.5227	0.0042	0.0152	-2.7726
Plxna2	1.4127	4.2583	3.5222	0.0042	0.0152	-2.5542
AU022252	0.5083	4.1207	3.522	0.0042	0.0152	-2.5582
Tcea3	3.0953	-0.588	3.5216	0.0042	0.0153	-1.7736
Chrnb2	1.5342	-0.0176	3.5213	0.0042	0.0153	-1.865
Amigo2	1.6233	4.3514	3.5212	0.0042	0.0153	-2.5258
Cstad	1.9595	-2.2221	3.5212	0.0042	0.0153	-1.7658
Hspb2	2.8984	0.338	3.5204	0.0042	0.0153	-1.8542
Gm10131	-1.3513	0.5663	-3.52	0.0042	0.0153	-1.9022
Xpa	0.6762	2.9613	3.5197	0.0042	0.0153	-2.3541
Zfp770	-0.6374	4.5813	-3.5196	0.0042	0.0153	-2.6036

Gm38150	-2.1007	-1.3413	-3.5192	0.0043	0.0153	-1.7727
Gm16433	-0.8832	0.5441	-3.5188	0.0043	0.0153	-1.9253
Slc35c2	-0.4137	5.052	-3.5179	0.0043	0.0153	-2.6864
Crtc1	0.6358	4.7502	3.5177	0.0043	0.0153	-2.6496
1190007I07Rik	-0.8366	1.7897	-3.5176	0.0043	0.0153	-2.0914
Gm13292	-1.6455	-0.9633	-3.5164	0.0043	0.0154	-1.7937
Rbm27	-0.5017	6.3683	-3.5161	0.0043	0.0154	-2.801
Abhd12	0.4573	5.7129	3.5145	0.0043	0.0154	-2.7773
Sirt5	0.7537	3.1301	3.5129	0.0043	0.0154	-2.3872
Kirrel	0.5836	8.0693	3.5127	0.0043	0.0154	-2.8521
Agk	-0.5428	3.9586	-3.5123	0.0043	0.0155	-2.5281
Jmy	0.5798	6.6895	3.5117	0.0043	0.0155	-2.8287
Ccdc90b	0.8175	3.746	3.5092	0.0043	0.0155	-2.5024
Ikzf2	2.4004	1.2068	3.5091	0.0043	0.0155	-1.977
Ppp1r12b	1.2724	4.9175	3.5088	0.0043	0.0155	-2.6894
Zfp521	2.4258	0.7877	3.5087	0.0043	0.0155	-1.9412
Ttc9c	-0.397	5.9811	-3.5081	0.0043	0.0156	-2.8003
Sp3os	0.7634	1.4871	3.5066	0.0044	0.0156	-2.1279
Cyb561d2	0.7415	3.0116	3.5063	0.0044	0.0156	-2.3736
Scube3	3.8986	-0.0623	3.506	0.0044	0.0156	-1.8129
Gpr179	2.2928	-1.2729	3.5056	0.0044	0.0156	-1.7911
Tmem263	-0.6616	5.3131	-3.5053	0.0044	0.0156	-2.7337
Hebp1	1.1747	4.6347	3.5042	0.0044	0.0157	-2.6711
Arhgap12	0.4898	5.7015	3.5035	0.0044	0.0157	-2.7944
Ddx51	-0.6232	5.2909	-3.5034	0.0044	0.0157	-2.7343
Aars	-0.5013	8.1128	-3.503	0.0044	0.0157	-2.8695
Elf4	0.447	6.1697	3.5027	0.0044	0.0157	-2.8256
Gm43681	1.0299	-0.3266	3.5024	0.0044	0.0157	-1.8649
Zfpl1	0.3673	4.4076	3.5019	0.0044	0.0157	-2.6414
B3gnt2	-0.7495	2.5826	-3.5016	0.0044	0.0157	-2.2907
Nrbp1	0.4907	6.6794	3.5014	0.0044	0.0157	-2.8492
Lrrc73	1.0335	0.9778	3.4993	0.0044	0.0158	-2.0483
Gm14866	2.8062	-2.9212	3.4988	0.0044	0.0158	-1.8125
Gna12	0.4274	6.6234	3.4987	0.0044	0.0158	-2.8502
Tent2	-0.3441	6.0623	-3.4987	0.0044	0.0158	-2.8244
Itga3	-0.6648	8.6778	-3.4985	0.0044	0.0158	-2.8846
Dph5	-0.5509	4.4448	-3.4983	0.0044	0.0158	-2.6366
Luc7l	-0.5025	6.2182	-3.4981	0.0044	0.0158	-2.8291
Lsm14b	-0.4725	6.8785	-3.498	0.0044	0.0158	-2.8564
Cenpj	0.5574	4.4435	3.4971	0.0044	0.0158	-2.6504
Gls2	-0.8345	1.4683	-3.4968	0.0044	0.0158	-2.1071
Hacd4	1.7808	0.0692	3.4968	0.0044	0.0158	-1.8912
Gnpda2	0.5795	4.0418	3.4967	0.0044	0.0158	-2.575
Zfp112	0.8862	1.4094	3.4964	0.0044	0.0158	-2.129
Actr1b	-0.3273	7.3335	-3.495	0.0044	0.0158	-2.8714
9330159M07Rik	-1.2256	0.8333	-3.4922	0.0045	0.0159	-1.9984
Ccl27a	0.8757	0.9285	3.4909	0.0045	0.016	-2.041
Plcxd2	0.9044	2.4759	3.4905	0.0045	0.016	-2.2866
Gch1	-1.0401	3.4584	-3.4905	0.0045	0.016	-2.4245
Gm45515	1.2847	-1.0359	3.49	0.0045	0.016	-1.8421
Elp5	-0.3434	5.7724	-3.49	0.0045	0.016	-2.8221
Dync2h1	0.9089	4.588	3.49	0.0045	0.016	-2.6764

Gm12919	-1.4896	-1.8399	-3.4899	0.0045	0.016	-1.8154
Lrrc17	3.9067	-3.2134	3.4898	0.0045	0.016	-1.8857
Pick1	0.7306	2.8472	3.4879	0.0045	0.016	-2.3813
Cdk19	0.489	5.8455	3.4871	0.0045	0.016	-2.836
Thap12	-0.4532	6.6271	-3.4871	0.0045	0.016	-2.8677
Mpv17l	1.1883	1.8322	3.4867	0.0045	0.016	-2.1813
Il1rl1	-2.3887	-0.0457	-3.486	0.0045	0.0161	-1.8378
Dcst2	2.6501	-2.8978	3.4837	0.0045	0.0161	-1.8556
Mmp19	-1.0138	4.7353	-3.4832	0.0045	0.0161	-2.6748
Ankra2	0.5489	4.0702	3.483	0.0045	0.0161	-2.6158
1700096K18Rik	1.2638	0.0923	3.4827	0.0045	0.0161	-1.957
Commd2	-0.3279	5.8255	-3.4822	0.0046	0.0162	-2.8392
Emsy	-0.2994	5.7002	-3.482	0.0046	0.0162	-2.8293
Disp1	0.5591	4.7296	3.4816	0.0046	0.0162	-2.7342
Casp4	1.632	3.4206	3.4812	0.0046	0.0162	-2.442
Fam71f2	-1.368	-1.335	-3.4801	0.0046	0.0162	-1.8374
Med12l	1.7582	-0.9701	3.4789	0.0046	0.0162	-1.8534
Esyt1	0.453	5.3312	3.4788	0.0046	0.0162	-2.8143
Kmt2c	0.8355	5.9747	3.4785	0.0046	0.0162	-2.8558
Hist1h4j	1.4418	-0.9704	3.4778	0.0046	0.0163	-1.856
Cdk5	0.4202	5.2142	3.4777	0.0046	0.0163	-2.7982
Ebpl	0.6937	2.8913	3.477	0.0046	0.0163	-2.421
Dmtf1	-0.4481	5.8721	-3.4767	0.0046	0.0163	-2.8494
AI987944	0.5549	3.5254	3.4753	0.0046	0.0163	-2.5264
Mtx2	-0.4249	5.9348	-3.4725	0.0046	0.0164	-2.8625
Cpt2	0.5772	4.4236	3.4725	0.0046	0.0164	-2.7035
Mrpl37	-0.3984	5.5067	-3.4721	0.0046	0.0164	-2.8274
Ube2e1	-0.4355	6.0241	-3.4719	0.0046	0.0164	-2.8671
Tenm4	-1.588	5.9919	-3.4713	0.0046	0.0164	-2.8163
Trim41	0.3536	5.0065	3.47	0.0047	0.0165	-2.7913
Cuedc2	0.4662	5.7255	3.4693	0.0047	0.0165	-2.8655
Mrpl1	-0.4721	5.1608	-3.4691	0.0047	0.0165	-2.7901
Ppp1r10	0.3818	6.3799	3.4688	0.0047	0.0165	-2.8969
Gm7862	-0.5317	2.8171	-3.4687	0.0047	0.0165	-2.4089
Slc29a2	-1.1389	3.4418	-3.4687	0.0047	0.0165	-2.4523
Vps26c	0.3719	5.5425	3.4684	0.0047	0.0165	-2.8522
Ppfibp1	-0.3086	8.4499	-3.4683	0.0047	0.0165	-2.9381
Zfp458	0.9528	1.2876	3.4683	0.0047	0.0165	-2.1393
Tmem203	0.6344	2.6877	3.468	0.0047	0.0165	-2.3929
Uqcrfs1	-0.3359	7.2497	-3.4672	0.0047	0.0165	-2.9225
2700054A10Rik	1.6718	-0.562	3.467	0.0047	0.0165	-1.905
Pikfyve	0.5112	5.8038	3.4664	0.0047	0.0165	-2.8687
Ifi204	-1.4868	3.8489	-3.466	0.0047	0.0165	-2.4734
Snrpa	-0.3948	6.5347	-3.4658	0.0047	0.0165	-2.907
Acat1	-0.3995	6.4462	-3.4653	0.0047	0.0165	-2.9064
Uri1	-0.2959	5.9216	-3.4653	0.0047	0.0165	-2.8753
Irf7	2.4079	0.8558	3.4653	0.0047	0.0165	-2.0342
Sdr42e1	2.3741	-2.3075	3.4645	0.0047	0.0166	-1.8665
Il34	0.5454	5.1125	3.4638	0.0047	0.0166	-2.8076
Ccng2	0.6586	6.9414	3.4637	0.0047	0.0166	-2.9265
Klhl2	-0.4085	5.6181	-3.4621	0.0047	0.0166	-2.8562
Trmt1	-0.3954	6.1856	-3.4621	0.0047	0.0166	-2.8981

Tmtc3	-0.5506	5.8644	-3.4619	0.0047	0.0166	-2.8644
Ecpas	-0.4086	7.4014	-3.4619	0.0047	0.0166	-2.9344
Pear1	-1.8432	0.2477	-3.4608	0.0047	0.0167	-1.9649
Gm20186	1.5121	1.9091	3.4605	0.0047	0.0167	-2.2338
Rabggta	-0.6891	4.044	-3.4597	0.0047	0.0167	-2.6369
BC022960	-1.2721	0.9927	-3.4594	0.0047	0.0167	-2.0563
Lig4	0.5578	4.0321	3.4587	0.0048	0.0167	-2.654
Ddost	-0.4457	8.1723	-3.4584	0.0048	0.0167	-2.9529
Il17rc	0.8835	5.4732	3.4578	0.0048	0.0167	-2.8586
Net1	0.9629	7.1316	3.4577	0.0048	0.0167	-2.9391
Zfp827	0.7841	4.5639	3.4562	0.0048	0.0168	-2.7415
Ints1	-0.5979	7.0175	-3.456	0.0048	0.0168	-2.9356
Pak2	-0.3778	7.2656	-3.4549	0.0048	0.0168	-2.9442
Ets2	0.6657	6.8912	3.4528	0.0048	0.0169	-2.9419
Syap1	0.4601	6.1749	3.4526	0.0048	0.0169	-2.9171
Def8	0.3672	5.2985	3.4517	0.0048	0.0169	-2.8513
Fmo5	2.3581	0.2606	3.4513	0.0048	0.0169	-1.9883
Tatdn2	-0.3538	6.7009	-3.4508	0.0048	0.0169	-2.9395
Vwf	2.4787	-1.2647	3.4501	0.0048	0.0169	-1.8811
Mtmr4	0.3575	6.3888	3.4496	0.0048	0.0169	-2.9333
Dhrs3	2.0454	1.7955	3.4492	0.0048	0.017	-2.2015
Gm8129	-1.2412	-0.4172	-3.4491	0.0048	0.017	-1.9423
Snw1	0.4115	7.2577	3.4488	0.0048	0.017	-2.9581
Polr1a	-0.6035	7.5444	-3.4486	0.0048	0.017	-2.9589
Tmem67	0.6804	2.6766	3.4486	0.0048	0.017	-2.4209
Ddx1	-0.4399	8.167	-3.4482	0.0048	0.017	-2.9717
Trak2	-0.3154	6.5141	-3.4468	0.0049	0.017	-2.9404
Fam110a	-0.6764	3.7717	-3.4459	0.0049	0.017	-2.6056
Pde12	-0.5101	5.2237	-3.4457	0.0049	0.017	-2.8368
Vapb	-0.401	6.8358	-3.4451	0.0049	0.017	-2.9534
Rnf114	0.4256	6.035	3.4451	0.0049	0.017	-2.9281
Spcs3	-0.5176	7.2889	-3.4437	0.0049	0.0171	-2.9644
Gm10709	-1.5057	-1.7424	-3.4433	0.0049	0.0171	-1.8891
Gm9892	-0.9638	1.1967	-3.4418	0.0049	0.0171	-2.1587
Evi5l	0.9407	3.6328	3.4412	0.0049	0.0172	-2.6063
Ino80c	-0.5539	5.5558	-3.4411	0.0049	0.0172	-2.8842
E330032C10Rik	-3.7864	-1.9308	-3.441	0.0049	0.0172	-1.9514
Wrn	0.3944	4.922	3.441	0.0049	0.0172	-2.8253
Foxo1	0.5427	5.1569	3.4408	0.0049	0.0172	-2.8501
Fam131a	1.8057	1.0011	3.4407	0.0049	0.0172	-2.0882
Car11	2.0995	-2.0482	3.4405	0.0049	0.0172	-1.9005
Srp72	-0.3886	7.6624	-3.4401	0.0049	0.0172	-2.9791
Cdk15	2.629	-3.5598	3.4397	0.0049	0.0172	-1.9465
Dhrs7	0.5028	4.6996	3.4395	0.0049	0.0172	-2.8185
Kmt2d	0.5524	6.5399	3.4395	0.0049	0.0172	-2.9555
Scnn1a	1.2981	0.7662	3.4392	0.0049	0.0172	-2.105
Hist2h2aa2	3.2036	-3.689	3.4357	0.005	0.0173	-1.9551
Tfcp2	0.5835	4.3191	3.4353	0.005	0.0173	-2.7537
Smpd2	0.7559	3.9979	3.4353	0.005	0.0173	-2.6889
Erlin2	0.5272	6.3398	3.4351	0.005	0.0173	-2.96
Arhgap31	0.6787	5.3874	3.4349	0.005	0.0173	-2.8799
8430423G03Rik	-2.454	-2.959	-3.4341	0.005	0.0173	-1.9414

Zfp729b	0.7452	3.5316	3.4335	0.005	0.0173	-2.5987
Adam33	-3.3816	-2.9115	-3.4334	0.005	0.0173	-1.9579
Lars2	0.5871	9.8765	3.4333	0.005	0.0173	-3.0144
Morn2	0.7013	1.6878	3.4333	0.005	0.0173	-2.2992
Dusp12	-0.4985	4.2405	-3.4332	0.005	0.0173	-2.7256
Nradd	0.7502	4.7134	3.4323	0.005	0.0174	-2.8097
Ppie	-0.4357	5.0175	-3.4319	0.005	0.0174	-2.8489
Cirbp	0.5768	3.5056	3.4316	0.005	0.0174	-2.6345
Nuggc	1.2263	0.0046	3.4315	0.005	0.0174	-2.0606
Rnf170	0.4775	3.8925	3.4314	0.005	0.0174	-2.6753
Rnd3	0.9214	4.6184	3.4314	0.005	0.0174	-2.7675
Lca5l	1.9679	-2.0249	3.4312	0.005	0.0174	-1.9089
Nckap5l	0.7391	4.8113	3.4309	0.005	0.0174	-2.8246
Macf1	0.6543	7.9504	3.4304	0.005	0.0174	-3.0032
Recql5	0.4322	4.4304	3.43	0.005	0.0174	-2.7795
Nedd9	1.104	4.4522	3.4294	0.005	0.0174	-2.7557
Mtus1	3.3178	1.9789	3.4292	0.005	0.0174	-2.1668
AU022754	-1.7892	-0.0722	-3.4289	0.005	0.0174	-1.9633
Aox3	4.8544	-1.437	3.4279	0.005	0.0175	-1.9556
Zfp354a	0.7675	1.9379	3.4261	0.005	0.0175	-2.3246
Ttc27	-0.3785	5.9563	-3.4261	0.005	0.0175	-2.9491
Sh3bgrl3	-0.3494	5.87	-3.4261	0.005	0.0175	-2.9511
Fa2h	-2.0752	-2.4032	-3.4255	0.0051	0.0175	-1.9403
Gm5532	2.9076	-2.8495	3.425	0.0051	0.0175	-1.9443
Shkbp1	0.547	3.8582	3.4242	0.0051	0.0176	-2.6901
Adamts17	3.4419	-2.1441	3.4242	0.0051	0.0176	-1.9507
Ep300	0.5008	7.2899	3.4236	0.0051	0.0176	-3.0047
Gm26610	1.3217	-1.6169	3.4235	0.0051	0.0176	-1.9382
H6pd	0.7781	5.972	3.4231	0.0051	0.0176	-2.9623
Bend4	-0.8013	4.9405	-3.4228	0.0051	0.0176	-2.8284
Rtn2	-0.7397	2.5363	-3.4228	0.0051	0.0176	-2.427
Gm7125	-1.3576	0.5808	-3.4225	0.0051	0.0176	-2.0593
Prkag2	0.4553	4.6012	3.4222	0.0051	0.0176	-2.815
Smtn	-0.8467	7.3144	-3.4216	0.0051	0.0176	-3.0065
Ddx5	0.3165	9.8515	3.4211	0.0051	0.0176	-3.0372
B4galt7	-0.3855	5.0388	-3.4201	0.0051	0.0176	-2.8768
Dbp	1.0383	2.0508	3.4192	0.0051	0.0177	-2.3665
Rufy4	-1.4526	0.0467	-3.419	0.0051	0.0177	-2.0188
Rabac1	0.6628	5.262	3.4188	0.0051	0.0177	-2.9134
Gnas	0.3512	9.8956	3.4187	0.0051	0.0177	-3.0422
Gm6807	-0.8445	0.9125	-3.4179	0.0051	0.0177	-2.1682
Cdr2l	-0.4905	6.395	-3.4174	0.0051	0.0177	-2.9914
Hopx	2.2932	0.8866	3.4165	0.0051	0.0177	-2.0952
1700065D16Rik	1.4022	-1.9252	3.4159	0.0051	0.0178	-1.9322
Lynx1	2.813	2.6456	3.4149	0.0052	0.0178	-2.3321
Smim3	-1.1843	5.4153	-3.4144	0.0052	0.0178	-2.8816
Zswim9	0.5869	3.2456	3.4136	0.0052	0.0178	-2.6047
Gm16374	-1.5852	-1.307	-3.4131	0.0052	0.0178	-1.9427
Hivep1	0.5002	5.8292	3.4114	0.0052	0.0179	-2.974
Pcbp3	-1.7798	2.363	-3.4109	0.0052	0.0179	-2.3169
Gm20721	0.5023	4.4001	3.4103	0.0052	0.0179	-2.8198
4931414P19Rik	0.644	3.2669	3.4099	0.0052	0.0179	-2.6066

Mob3a	-0.4288	5.1983	-3.4078	0.0052	0.018	-2.9169
Snhg20	-1.031	2.8817	-3.4076	0.0052	0.018	-2.4715
Gm5611	-1.0866	1.2975	-3.4074	0.0052	0.018	-2.2226
Tmem42	0.8404	1.4557	3.4068	0.0052	0.018	-2.2821
Snhg12	-0.8989	4.1527	-3.4068	0.0052	0.018	-2.728
Lrrk1	0.7668	5.7257	3.4065	0.0052	0.018	-2.9759
Sik1	0.8907	6.5462	3.4063	0.0052	0.018	-3.0194
4930447K03Rik	1.9407	-3.2	3.4062	0.0052	0.018	-1.9597
Glt8d2	3.7081	-0.6052	3.4058	0.0052	0.018	-1.9496
Ccdc47	-0.3293	7.4067	-3.4054	0.0052	0.018	-3.0395
Edn1	2.6859	-2.1282	3.4053	0.0052	0.018	-1.9502
Adora1	-2.6423	-2.9931	-3.4045	0.0053	0.0181	-1.9944
Gm8909	1.0266	1.5808	3.4042	0.0053	0.0181	-2.3187
Polr3e	-0.8756	5.0049	-3.4037	0.0053	0.0181	-2.8609
Tmem222	0.4118	4.4093	3.4034	0.0053	0.0181	-2.8296
Foxq1	1.8141	2.8622	3.4031	0.0053	0.0181	-2.4847
Phf20l1	0.4439	5.9953	3.403	0.0053	0.0181	-3.0004
Gbp2b	2.8894	-0.0384	3.4024	0.0053	0.0181	-2
Tmcc2	0.7865	4.4905	3.4013	0.0053	0.0181	-2.8361
Nat8f1	1.2425	-0.0016	3.401	0.0053	0.0182	-2.0765
Bbs1	1.5627	0.9219	3.4007	0.0053	0.0182	-2.1819
Plcb3	-0.5219	6.3027	-3.4003	0.0053	0.0182	-3.0184
Arl5c	2.4866	-3.4627	3.4001	0.0053	0.0182	-2.0046
Arhgef10l	0.8502	5.5486	3.3995	0.0053	0.0182	-2.9768
Supt4a	0.4639	5.6403	3.3994	0.0053	0.0182	-2.9891
Galk2	0.5521	5.0973	3.3994	0.0053	0.0182	-2.9287
Ercc8	-0.5822	3.649	-3.399	0.0053	0.0182	-2.6644
Rcc2	-0.4402	8.7302	-3.3989	0.0053	0.0182	-3.07
H2-D1	0.8926	8.4302	3.3987	0.0053	0.0182	-3.0691
Gclm	-0.566	6.7438	-3.3986	0.0053	0.0182	-3.0368
Camk1d	0.7381	6.5573	3.3984	0.0053	0.0182	-3.0331
Sub1	-0.511	7.0129	-3.398	0.0053	0.0182	-3.045
Pip4p2	0.6204	5.4547	3.3974	0.0053	0.0182	-2.9672
Rpgrip1l	0.4414	4.4762	3.3973	0.0053	0.0182	-2.8358
Yy1	-0.2652	7.4477	-3.3972	0.0053	0.0182	-3.0554
Tmem128	-0.4253	5.0326	-3.397	0.0053	0.0182	-2.917
AC131185.2	-1.3474	-1.9219	-3.3953	0.0053	0.0183	-1.966
Gm16576	1.1835	2.162	3.3943	0.0054	0.0183	-2.4229
Il10rb	1.0025	4.1442	3.3936	0.0054	0.0183	-2.7876
Hlf	2.1332	1.0611	3.3932	0.0054	0.0183	-2.189
2810029C07Rik	1.1331	-0.6567	3.3931	0.0054	0.0183	-2.0372
Nelfe	-0.5177	5.3057	-3.393	0.0054	0.0183	-2.95
Tpr	-0.4127	8.8074	-3.3927	0.0054	0.0183	-3.0821
Ccdc120	0.5353	3.9579	3.3926	0.0054	0.0183	-2.7736
Idh2	0.4993	7.72	3.3919	0.0054	0.0184	-3.0727
Nr2f2	0.8233	6.4901	3.3918	0.0054	0.0184	-3.041
Id3	1.5747	5.7518	3.3916	0.0054	0.0184	-2.9968
Yipf3	0.4343	5.6173	3.3911	0.0054	0.0184	-3.0029
Rpain	0.5905	3.6699	3.3909	0.0054	0.0184	-2.7253
Hspb6	0.5058	4.0492	3.3908	0.0054	0.0184	-2.78
Dock11	0.6651	6.5127	3.3905	0.0054	0.0184	-3.0477
Glp2r	2.0169	-2.8139	3.3904	0.0054	0.0184	-1.9837

Ndufa7	0.4769	6.1438	3.3891	0.0054	0.0184	-3.0382
Snora23	-1.7281	-1.8544	-3.3879	0.0054	0.0185	-1.9838
Oaz1	-0.3198	7.2888	-3.387	0.0054	0.0185	-3.0722
Slc25a22	-0.5488	4.304	-3.3865	0.0054	0.0185	-2.8108
Fam13b	0.8338	5.2598	3.3861	0.0054	0.0185	-2.962
Fbxo44	1.1267	2.7574	3.3858	0.0054	0.0185	-2.5241
Ankrd42	0.9163	1.9323	3.3858	0.0054	0.0185	-2.4205
Vamp3	-0.4111	5.8646	-3.3853	0.0054	0.0185	-3.0179
Spryd7	-0.5193	4.2463	-3.3842	0.0055	0.0186	-2.8017
Jtb	0.3899	5.7886	3.3834	0.0055	0.0186	-3.0282
Tdrd3	0.5566	5.0223	3.383	0.0055	0.0186	-2.949
Khk	1.3574	1.7588	3.3828	0.0055	0.0186	-2.3595
Slc30a9	-0.3002	6.8003	-3.3824	0.0055	0.0186	-3.071
Ccl17	3.4953	-2.7269	3.381	0.0055	0.0187	-2.0392
Hira	-0.382	4.792	-3.3805	0.0055	0.0187	-2.9015
Zeb2os	2.67	-0.4685	3.3799	0.0055	0.0187	-2.0224
Ube3a	-0.5545	7.0005	-3.3797	0.0055	0.0187	-3.0768
Abca6	2.2902	-0.7278	3.3795	0.0055	0.0187	-2.0105
Calcoco2	3.0062	-3.6967	3.3789	0.0055	0.0187	-2.0359
Zcchc9	-0.4167	4.7372	-3.3781	0.0055	0.0187	-2.912
Gm45495	1.4195	0.2705	3.3775	0.0055	0.0188	-2.1722
Tanc2	0.7258	6.8844	3.377	0.0055	0.0188	-3.0824
Gm20687	2.6202	-3.1464	3.3766	0.0055	0.0188	-2.0145
Tbl2	-0.525	5.899	-3.3763	0.0055	0.0188	-3.0358
Akap7	0.8957	2.6414	3.3762	0.0055	0.0188	-2.5338
Gm15050	1.2108	0.1294	3.3744	0.0056	0.0188	-2.1429
Gm17837	-2.1335	-1.7965	-3.3743	0.0056	0.0188	-1.9995
Ctnnbip1	0.5095	4.5105	3.3732	0.0056	0.0189	-2.8998
Arhgef19	2.206	2.6477	3.3724	0.0056	0.0189	-2.442
Gm10425	-3.0104	0.3477	-3.3721	0.0056	0.0189	-2.0193
2810004N23Rik	-0.5537	5.9684	-3.3717	0.0056	0.0189	-3.0496
Galnt3	-2.451	2.2294	-3.3716	0.0056	0.0189	-2.2635
Hsdl1	0.5433	5.097	3.3708	0.0056	0.0189	-2.9828
Psmd13	-0.3364	6.8036	-3.3707	0.0056	0.0189	-3.0932
Tmem60	-0.4981	3.9945	-3.3699	0.0056	0.019	-2.7915
Arpc5l	-0.4231	5.8294	-3.369	0.0056	0.019	-3.0493
A630072M18Rik	0.8321	1.6982	3.3685	0.0056	0.019	-2.4023
Wdr73	-0.5114	4.344	-3.3672	0.0056	0.019	-2.8576
Crebzf	-0.4833	5.0489	-3.3672	0.0056	0.019	-2.9651
Gpatch11	0.3613	5.0188	3.3669	0.0056	0.0191	-2.9826
Pex2	0.5043	5.4721	3.3657	0.0056	0.0191	-3.0318
Atp5c1	-0.3628	8.2756	-3.3656	0.0056	0.0191	-3.1272
Slc7a7	1.8035	-0.1905	3.3652	0.0056	0.0191	-2.1261
Atp2a2	-0.4573	8.9202	-3.3649	0.0057	0.0191	-3.1347
Nampt	-0.458	7.5755	-3.3626	0.0057	0.0192	-3.1214
Hes1	-1.8699	2.8983	-3.3625	0.0057	0.0192	-2.4545
Clk3	-0.3458	5.9632	-3.3619	0.0057	0.0192	-3.074
Noxred1	2.141	-1.9252	3.3601	0.0057	0.0193	-2.0207
Hps4	0.8698	4.3801	3.3597	0.0057	0.0193	-2.8922
Thsd4	-2.2527	1.4722	-3.3595	0.0057	0.0193	-2.2109
Zfp185	2.7855	-1.8759	3.3591	0.0057	0.0193	-2.0219
Gm13270	3.2466	-0.6994	3.359	0.0057	0.0193	-2.0325

1810032O08Rik	-0.788	3.3056	-3.359	0.0057	0.0193	-2.6467
Gm5844	-1.2196	0.7156	-3.3588	0.0057	0.0193	-2.2158
Hmbox1	0.4544	4.9019	3.3585	0.0057	0.0193	-2.9752
Gpatch3	0.7323	3.3538	3.3582	0.0057	0.0193	-2.7104
Gm16244	1.1103	-0.8646	3.3582	0.0057	0.0193	-2.0827
Agpat5	-0.658	5.9101	-3.3578	0.0057	0.0193	-3.066
Tmem200a	-3.016	0.1909	-3.3565	0.0057	0.0194	-2.054
Dohh	-0.4703	4.1473	-3.3561	0.0057	0.0194	-2.8431
Polg	-0.3592	6.1221	-3.3561	0.0057	0.0194	-3.0902
Pdcd6	-0.3634	6.7679	-3.3559	0.0057	0.0194	-3.1189
Nat14	1.1534	-0.2813	3.3557	0.0057	0.0194	-2.1137
Gm43072	-2.2015	-2.8269	-3.3555	0.0058	0.0194	-2.0655
E130102H24Rik	1.3583	-0.6517	3.3547	0.0058	0.0194	-2.0847
Arl16	-0.5077	3.9678	-3.3544	0.0058	0.0194	-2.804
Actr3	-0.2865	8.4794	-3.353	0.0058	0.0194	-3.1529
Prr13	1.0178	6.2296	3.3518	0.0058	0.0195	-3.1083
Acy3	1.4806	0.8759	3.3507	0.0058	0.0195	-2.2541
Acot1	2.9593	1.8093	3.3488	0.0058	0.0196	-2.269
Anapc13	0.4776	4.456	3.3484	0.0058	0.0196	-2.9386
Whrn	0.7889	1.7665	3.3483	0.0058	0.0196	-2.425
Pole4	-0.6325	5.0592	-3.3482	0.0058	0.0196	-2.9942
Ppip5k1	0.4561	4.0977	3.3482	0.0058	0.0196	-2.8771
Scaf4	-0.3783	5.6018	-3.3481	0.0058	0.0196	-3.0642
Rab3d	1.37	2.6188	3.3479	0.0058	0.0196	-2.5827
B3galt6	-0.5095	3.5298	-3.3479	0.0058	0.0196	-2.7501
Prrg2	0.5073	3.2705	3.3478	0.0058	0.0196	-2.7356
Pgls	-0.4681	5.7519	-3.3469	0.0058	0.0196	-3.0845
Zbtb12	-0.4978	4.2693	-3.3465	0.0058	0.0196	-2.8846
Gm24187	0.844	5.2281	3.3465	0.0058	0.0196	-3.0223
Serpinf2	2.6676	-3.2413	3.3462	0.0058	0.0196	-2.0722
Emc1	-0.4411	6.0993	-3.346	0.0059	0.0196	-3.1072
Tubb4b-ps1	-1.7677	-1.3632	-3.3456	0.0059	0.0196	-2.047
Prune1	0.5998	5.7935	3.3454	0.0059	0.0197	-3.0987
Atp1a1	-0.37	9.6452	-3.3442	0.0059	0.0197	-3.1787
Alas1	0.3217	5.6371	3.3438	0.0059	0.0197	-3.0893
Trerf1	-0.5426	4.8911	-3.3435	0.0059	0.0197	-2.9863
Rnf214	0.4193	5.2114	3.343	0.0059	0.0197	-3.043
5033417F24Rik	2.0653	-1.1773	3.3428	0.0059	0.0197	-2.0644
Pou6f1	1.0073	2.7025	3.3425	0.0059	0.0197	-2.6196
Dcun1d4	0.7491	4.813	3.3408	0.0059	0.0198	-2.9863
Tle1	0.5034	5.5405	3.3405	0.0059	0.0198	-3.0835
Papss1	-0.2816	7.0062	-3.3396	0.0059	0.0198	-3.1556
Gpi1	-0.5726	9.3657	-3.3393	0.0059	0.0198	-3.1862
Mtpn	-0.4134	8.159	-3.3387	0.0059	0.0199	-3.1745
Kiss1r	1.1464	-0.9576	3.3384	0.0059	0.0199	-2.1216
Hist1h4h	1.7125	-0.0235	3.3382	0.0059	0.0199	-2.1552
Tet3	0.5583	6.329	3.3381	0.0059	0.0199	-3.1383
Gm26738	-2.2838	-2.7087	-3.3376	0.0059	0.0199	-2.0758
R3hdm2	0.3643	6.0852	3.3374	0.0059	0.0199	-3.1307
Btbd7	0.4307	6.9057	3.3367	0.006	0.0199	-3.1587
B930082K07Rik	1.7492	-1.9271	3.3365	0.006	0.0199	-2.068
Prmt3	-0.62	5.7421	-3.3362	0.006	0.0199	-3.0932

Gm3325	-0.6472	2.7985	-3.3362	0.006	0.0199	-2.6156
Slc25a39	-0.3655	7.9607	-3.3356	0.006	0.0199	-3.1788
Gramd1b	0.851	4.0136	3.3355	0.006	0.0199	-2.8759
Chrd	3.9399	-0.3734	3.3355	0.006	0.0199	-2.0718
Fam102a	0.6999	3.3379	3.3351	0.006	0.0199	-2.7497
Mrpl30	-0.2804	6.0388	-3.334	0.006	0.02	-3.1315
Zc3h7a	-0.5733	5.881	-3.3339	0.006	0.02	-3.1106
Apoe	-1.0815	1.1452	-3.3336	0.006	0.02	-2.2995
8430426J06Rik	2.4144	-2.2305	3.3333	0.006	0.02	-2.0626
Mfsd12	0.4799	3.8021	3.3332	0.006	0.02	-2.8518
Taf7	0.6831	2.4997	3.3329	0.006	0.02	-2.583
Crcp	-0.4585	4.4583	-3.3328	0.006	0.02	-2.9433
Vps28	0.5128	5.3893	3.3323	0.006	0.02	-3.0893
Tango6	-0.4937	4.7228	-3.3316	0.006	0.02	-2.9825
Trpv4	2.35	2.9005	3.3316	0.006	0.02	-2.5943
Pkd1l1	1.7146	-0.965	3.3315	0.006	0.02	-2.0933
Usp17la	-1.7335	-0.1353	-3.3314	0.006	0.02	-2.1463
Gm7879	-1.0542	0.6684	-3.3307	0.006	0.02	-2.2586
Tspan3	0.3464	7.2558	3.33	0.006	0.0201	-3.1789
Gm10222	2.0317	-2.6312	3.3297	0.006	0.0201	-2.0832
Josd2	0.66	3.9398	3.3296	0.006	0.0201	-2.8787
Msln	1.4065	5.398	3.3292	0.006	0.0201	-3.0799
D2hgdh	0.5986	2.1317	3.3289	0.006	0.0201	-2.5394
Rhno1	-0.4186	5.2334	-3.3288	0.006	0.0201	-3.0619
Tesmin	1.7009	-1.5441	3.3281	0.006	0.0201	-2.0753
Serp2	-1.1993	1.3716	-3.328	0.006	0.0201	-2.3441
1810021B22Rik	1.7406	-0.2899	3.3279	0.0061	0.0201	-2.1606
Ppil6	2.4085	-1.9354	3.3276	0.0061	0.0201	-2.074
Kank1	1.226	3.6779	3.3274	0.0061	0.0201	-2.8332
Cand2	-0.8607	3.4075	-3.3255	0.0061	0.0202	-2.7504
Atp5a1	-0.3737	10.0112	-3.3253	0.0061	0.0202	-3.216
Ncoa1	0.9965	4.2113	3.3249	0.0061	0.0202	-2.9071
Pcyt1a	0.5451	6.0186	3.3244	0.0061	0.0202	-3.1506
Manba	1.5718	3.5701	3.3244	0.0061	0.0202	-2.7665
Samd1	-0.4745	6.0473	-3.3236	0.0061	0.0202	-3.1441
2700049A03Rik	-0.3823	4.6354	-3.323	0.0061	0.0203	-2.9915
Mthfd2l	-0.4893	4.413	-3.3228	0.0061	0.0203	-2.9512
Hsbp1	0.3954	6.5596	3.3218	0.0061	0.0203	-3.1792
Klhl18	0.5157	3.7723	3.3212	0.0061	0.0203	-2.8728
Hras	-0.3672	5.7895	-3.3205	0.0061	0.0203	-3.1362
Hist1h2bg	1.3944	0.5473	3.3204	0.0061	0.0203	-2.2872
Aplnr	1.3406	-1.2701	3.3202	0.0061	0.0203	-2.13
Gm12758	-1.1353	1.1549	-3.3196	0.0061	0.0204	-2.3624
Samd10	1.1894	1.9389	3.3187	0.0062	0.0204	-2.4958
Stk16	-0.3672	5.7194	-3.3187	0.0062	0.0204	-3.1345
Mllt10	-0.3658	5.8552	-3.3186	0.0062	0.0204	-3.1428
Hao1	-3.8233	0.3199	-3.3182	0.0062	0.0204	-2.0867
Tiparp	-0.5819	7.7182	-3.3176	0.0062	0.0204	-3.2062
Sptbn1	0.6191	8.9252	3.317	0.0062	0.0204	-3.2252
Arhgef1	-0.3529	6.4627	-3.3168	0.0062	0.0204	-3.1837
Sigirr	-1.5778	3.194	-3.3165	0.0062	0.0205	-2.6606
Zfp759	0.9833	1.5268	3.3161	0.0062	0.0205	-2.4588

Rogdi	0.4631	3.3013	3.3161	0.0062	0.0205	-2.7931
Pam16	-0.4606	4.1693	-3.3159	0.0062	0.0205	-2.9376
Gm7332	-0.7948	1.1797	-3.3157	0.0062	0.0205	-2.3832
Pdgfrl	4.5762	1.122	3.3156	0.0062	0.0205	-2.1346
Tctn3	0.8459	2.6697	3.3151	0.0062	0.0205	-2.6529
Crat	0.5868	5.9253	3.3147	0.0062	0.0205	-3.164
G430095P16Rik	1.1079	0.0975	3.3141	0.0062	0.0205	-2.2621
Psmc3ip	-0.4887	4.3105	-3.3137	0.0062	0.0205	-2.951
Dpysl2	-0.4605	6.5141	-3.312	0.0062	0.0206	-3.192
Bckdk	-0.3633	6.1376	-3.3107	0.0062	0.0206	-3.1776
Tmem129	-0.4533	4.5497	-3.3107	0.0062	0.0206	-3.0045
Rsrc2	-0.349	6.2351	-3.3104	0.0062	0.0206	-3.1815
Clstn3	2.2243	-2.8727	3.3103	0.0063	0.0206	-2.1122
Pogz	0.3528	6.2988	3.3098	0.0063	0.0206	-3.1901
Adhfe1	0.8155	2.3292	3.3094	0.0063	0.0207	-2.5901
2700097O09Rik	-0.5071	3.1194	-3.3092	0.0063	0.0207	-2.7399
Mrpl44	-0.3414	4.788	-3.3062	0.0063	0.0208	-3.0536
Pabpc1	-0.3623	11.2511	-3.306	0.0063	0.0208	-3.2561
Ino80	-0.3426	5.6189	-3.3056	0.0063	0.0208	-3.1466
Ier2	-1.1085	5.4879	-3.3054	0.0063	0.0208	-3.0802
Syvn1	0.3626	6.0266	3.3051	0.0063	0.0208	-3.1851
Nploc4	-0.2942	7.7042	-3.305	0.0063	0.0208	-3.2314
Gls	-0.4439	7.607	-3.3032	0.0063	0.0209	-3.2318
Mettl3	-0.487	4.3321	-3.3031	0.0063	0.0209	-2.9799
Stard3nl	-0.3324	5.3612	-3.303	0.0063	0.0209	-3.1338
Zfp426	0.4052	4.0036	3.3026	0.0063	0.0209	-2.9292
Zmynd15	1.9716	-0.5038	3.302	0.0063	0.0209	-2.1795
Gm12184	-1.1905	0.0792	-3.3019	0.0063	0.0209	-2.2543
Otud7b	0.5474	7.6699	3.3015	0.0064	0.0209	-3.2394
Dnajc11	-0.3603	6.3494	-3.3013	0.0064	0.0209	-3.2042
Cln8	0.9515	4.3412	3.3009	0.0064	0.0209	-3.0009
Pef1	0.454	5.6375	3.3004	0.0064	0.0209	-3.1716
Crhbp	-1.7982	-0.8781	-3.3	0.0064	0.0209	-2.1376
Lamc3	2.3336	-1.3605	3.2999	0.0064	0.0209	-2.1286
Gm25238	1.8953	-2.3449	3.2995	0.0064	0.021	-2.1158
Eppk1	4.7622	-1.084	3.2993	0.0064	0.021	-2.1163
Tasor	-0.376	5.0029	-3.2989	0.0064	0.021	-3.0813
Gm16740	0.7856	0.8489	3.2988	0.0064	0.021	-2.406
Gm49503	-1.9911	-0.4519	-3.2981	0.0064	0.021	-2.152
Mrpl40	-0.4761	5.0312	-3.298	0.0064	0.021	-3.0904
Gm21284	1.5635	-2.2986	3.2975	0.0064	0.021	-2.1219
Gm20033	1.2246	-0.1928	3.297	0.0064	0.021	-2.2625
Top3b	0.4811	5.243	3.2965	0.0064	0.021	-3.139
Txn2	-0.5501	7.1489	-3.2962	0.0064	0.0211	-3.2369
Osbpl10	-0.9343	2.4983	-3.2959	0.0064	0.0211	-2.6086
Pcdhgc4	1.0554	0.0749	3.295	0.0064	0.0211	-2.2534
Igfbp6	1.9138	2.5104	3.2946	0.0064	0.0211	-2.6016
Nmnat3	0.987	2.6068	3.2935	0.0064	0.0211	-2.6887
Herc1	0.5756	6.0275	3.2934	0.0064	0.0211	-3.2037
Pik3c2a	0.7809	6.6539	3.2932	0.0065	0.0211	-3.234
4930430F08Rik	-0.5488	3.6331	-3.2925	0.0065	0.0212	-2.8694
Akap9	0.7952	6.0497	3.2925	0.0065	0.0212	-3.208

Nit1	0.4217	4.6328	3.2911	0.0065	0.0212	-3.0662
Zmat2	0.3912	6.4706	3.2904	0.0065	0.0212	-3.2349
Ccn3	-2.1559	3.478	-3.29	0.0065	0.0212	-2.6642
Psca	3.2961	-2.694	3.2897	0.0065	0.0213	-2.168
Unc119b	-0.3032	6.2563	-3.2893	0.0065	0.0213	-3.2252
Flii	0.2992	7.0987	3.2889	0.0065	0.0213	-3.2537
Gm6263	2.021	-2.1166	3.2888	0.0065	0.0213	-2.135
Mbtd1	0.4314	5.8162	3.2885	0.0065	0.0213	-3.1976
Zcrb1	-0.5894	5.9064	-3.2882	0.0065	0.0213	-3.1995
Mir6236	0.8645	7.6021	3.2879	0.0065	0.0213	-3.2637
Slc17a8	1.9883	-2.572	3.2868	0.0065	0.0213	-2.1409
Dynlt3	0.6083	6.8827	3.2867	0.0065	0.0213	-3.2517
Zhx1	0.8535	5.5252	3.2866	0.0065	0.0213	-3.1777
Gm15895	2.5335	-1.6585	3.2866	0.0065	0.0213	-2.1368
Ric1	0.5547	5.5887	3.2862	0.0065	0.0213	-3.1879
Acsbg1	-2.8173	2.7055	-3.2861	0.0065	0.0213	-2.4757
Cwf19l1	-0.5379	4.4723	-3.2861	0.0065	0.0213	-3.0159
Cyp4v3	2.1063	0.8603	3.2841	0.0066	0.0214	-2.3815
Elp6	-0.4762	3.819	-3.2838	0.0066	0.0214	-2.9213
Ifi202b	-1.6081	5.4129	-3.2836	0.0066	0.0214	-3.0732
Gm4462	-2.2251	-1.7655	-3.2827	0.0066	0.0215	-2.1443
Mtmr7	1.7022	-1.0752	3.282	0.0066	0.0215	-2.1653
Cox11	-0.4448	3.6201	-3.2817	0.0066	0.0215	-2.8895
Fcnaos	1.6063	-3.0111	3.2813	0.0066	0.0215	-2.1441
Eif3g	-0.3449	7.3688	-3.281	0.0066	0.0215	-3.2703
Slc27a4	0.5631	5.6264	3.2799	0.0066	0.0216	-3.2044
Impact	0.8648	6.0364	3.278	0.0066	0.0216	-3.2346
Gstt1	2.1853	-0.3059	3.2777	0.0066	0.0216	-2.2149
Zfp36l1-ps	1.8683	-1.0031	3.2777	0.0066	0.0216	-2.1708
Gm5884	1.1346	-1.0026	3.2771	0.0066	0.0217	-2.2287
Gpr35	-2.8652	0.3283	-3.2765	0.0067	0.0217	-2.1952
Mfap1a	0.381	4.6854	3.2763	0.0067	0.0217	-3.1038
Pdcd5	-0.4246	5.6474	-3.2754	0.0067	0.0217	-3.2089
Irak3	2.0404	0.2732	3.2753	0.0067	0.0217	-2.2684
Gm29340	-2.4064	-3.1324	-3.2751	0.0067	0.0217	-2.182
Ankrd35	2.1053	-0.6526	3.2746	0.0067	0.0217	-2.1982
Trmt10a	-0.4543	4.4671	-3.2732	0.0067	0.0218	-3.0587
Mapk11	-1.2694	3.2852	-3.2731	0.0067	0.0218	-2.7735
Atr	-0.5988	5.3415	-3.2729	0.0067	0.0218	-3.1674
Lrrcc1	0.6215	4.2202	3.2719	0.0067	0.0218	-3.0342
Gna11	-0.3807	6.3112	-3.2719	0.0067	0.0218	-3.2581
6430562O15Rik	-2.2838	-2.68	-3.2717	0.0067	0.0218	-2.1796
Ovca2	0.652	2.8461	3.2712	0.0067	0.0218	-2.7766
Elmod3	0.5882	3.8237	3.2711	0.0067	0.0218	-2.9676
Zmiz1os1	1.7125	-0.7017	3.2699	0.0067	0.0219	-2.2305
Mapk14	-0.3248	6.8502	-3.2685	0.0068	0.0219	-3.2823
Mgrn1	0.4468	6.0819	3.2684	0.0068	0.0219	-3.259
Iws1	-0.4765	6.3633	-3.2681	0.0068	0.0219	-3.2657
Ahcyl1	-0.4232	7.8456	-3.2679	0.0068	0.0219	-3.302
Cd300lb	-2.1877	2.0183	-3.2677	0.0068	0.0219	-2.4653
Nucb1	0.3344	7.6245	3.2664	0.0068	0.022	-3.3036
Cox19	0.3997	4.1055	3.2664	0.0068	0.022	-3.024

Rhod	0.5268	4.4007	3.2647	0.0068	0.0221	-3.0784
Prokr1	-1.7598	-0.1592	-3.2645	0.0068	0.0221	-2.2581
Ly6d	2.6808	-3.3904	3.2645	0.0068	0.0221	-2.1999
2610306M01Rik	0.925	1.2425	3.2642	0.0068	0.0221	-2.53
Sec62	0.4648	7.6464	3.2639	0.0068	0.0221	-3.3079
Gm49492	2.2958	-1.5889	3.2634	0.0068	0.0221	-2.1764
Nckipsd	0.6032	3.4158	3.2634	0.0068	0.0221	-2.8981
Hmgcr	-0.7225	8.1825	-3.2631	0.0068	0.0221	-3.315
Arcn1	-0.3581	8.2775	-3.2624	0.0068	0.0221	-3.3187
Dnah2os	-3.021	-1.0681	-3.2622	0.0068	0.0221	-2.1742
Cd63	0.5475	8.7352	3.2621	0.0068	0.0221	-3.3258
Hist1h2ac	1.3464	0.398	3.2612	0.0068	0.0222	-2.3667
Tap2	2.4078	3.5488	3.261	0.0068	0.0222	-2.8011
Ctc1	-0.4509	5.7169	-3.2595	0.0069	0.0222	-3.2419
Gm10093	-0.4836	3.606	-3.2594	0.0069	0.0222	-2.9143
2610044O15Rik	0.4325	3.4695	3.2582	0.0069	0.0223	-2.9093
Fkbp7	0.9604	3.7371	3.2563	0.0069	0.0223	-2.987
Nenf	0.6427	4.4286	3.2557	0.0069	0.0224	-3.1008
Scfd1	0.3596	6.3066	3.2556	0.0069	0.0224	-3.2924
Acsl6	-3.6084	-2.3448	-3.2545	0.0069	0.0224	-2.2172
AA465934	-1.2191	0.368	-3.2545	0.0069	0.0224	-2.3386
Gm13237	-2.2473	-2.36	-3.2538	0.0069	0.0224	-2.1967
Cd68	0.8917	3.0936	3.2538	0.0069	0.0224	-2.8627
Il17rd	2.1893	1.4808	3.2538	0.0069	0.0224	-2.4989
Lrrc28	0.5495	2.9787	3.2521	0.007	0.0225	-2.839
Vwa1	1.3231	2.7048	3.2515	0.007	0.0225	-2.75
Lrrc46	1.5057	-1.2328	3.2513	0.007	0.0225	-2.2239
Etv3	0.4017	5.2103	3.2496	0.007	0.0226	-3.2168
Psenen	0.3982	4.5115	3.2491	0.007	0.0226	-3.1321
Ppfia3	1.9864	0.8708	3.249	0.007	0.0226	-2.443
Ei24	0.3016	6.2781	3.2488	0.007	0.0226	-3.3034
Plod1	0.6529	5.9627	3.2487	0.007	0.0226	-3.2854
Mcub	-1.1402	4.6913	-3.2485	0.007	0.0226	-3.0882
Ess2	0.3663	4.8337	3.2477	0.007	0.0226	-3.1777
Stk24	-0.307	6.4254	-3.2474	0.007	0.0226	-3.3083
Mpdz	0.505	5.9194	3.2468	0.007	0.0226	-3.2864
Taf4	-0.5257	5.3382	-3.2467	0.007	0.0226	-3.2198
Crnde	-0.8796	1.7075	-3.2467	0.007	0.0226	-2.5933
Rab7b	-0.7148	3.9607	-3.2464	0.007	0.0227	-2.9951
Mpp3	-4.1376	-0.9812	-3.2455	0.007	0.0227	-2.2189
Gm17382	2.1707	-3.1928	3.2452	0.0071	0.0227	-2.2268
Gm13010	2.6493	-1.3537	3.2447	0.0071	0.0227	-2.2053
Tmem167b	0.3419	5.957	3.2445	0.0071	0.0227	-3.296
Gmppa	0.5516	5.3184	3.244	0.0071	0.0227	-3.2481
Qtrt1	-0.8232	4.0818	-3.244	0.0071	0.0227	-3.0076
Ubn2	0.4081	6.1965	3.2436	0.0071	0.0227	-3.3056
Smox	-1.0928	5.0278	-3.2434	0.0071	0.0227	-3.1613
Klf2	1.9406	4.835	3.2433	0.0071	0.0227	-3.1471
Ankrd44	1.7826	4.7902	3.242	0.0071	0.0228	-3.124
Pttg1ip	0.5314	7.4741	3.2418	0.0071	0.0228	-3.347
Cdk5r1	0.7659	1.8046	3.2416	0.0071	0.0228	-2.6451
2310015A10Rik	0.798	0.9593	3.2416	0.0071	0.0228	-2.5142

Asah2	0.7094	3.246	3.2414	0.0071	0.0228	-2.9133
Ptpn23	0.3832	6.6838	3.2408	0.0071	0.0228	-3.3326
Bcor	0.412	5.2943	3.2407	0.0071	0.0228	-3.2449
Lamc2	0.7905	4.421	3.2405	0.0071	0.0228	-3.1325
Itga2b	0.8576	1.7633	3.2399	0.0071	0.0229	-2.6403
Fastk	-0.3864	5.757	-3.2394	0.0071	0.0229	-3.2873
Gpr153	2.3383	1.6094	3.2383	0.0071	0.0229	-2.5375
Ell2	0.4714	6.289	3.2379	0.0071	0.0229	-3.3224
Paqr8	1.6926	0.649	3.2379	0.0071	0.0229	-2.4261
Sf3b1	-0.3944	8.9872	-3.2374	0.0072	0.0229	-3.3729
Ndufa6	0.5169	6.4627	3.2374	0.0072	0.0229	-3.334
Cutc	0.7072	2.3805	3.2373	0.0072	0.0229	-2.73
Pi15	2.2916	-1.3162	3.2372	0.0072	0.0229	-2.2407
Gm43566	-0.8382	1.0488	-3.2362	0.0072	0.023	-2.4636
Reps2	2.3194	0.4567	3.2355	0.0072	0.023	-2.3062
Hnrnpa1	-0.3704	9.4579	-3.2349	0.0072	0.023	-3.3809
Entpd2	2.2321	-2.4571	3.2345	0.0072	0.023	-2.2184
Atg2a	0.6426	5.9958	3.234	0.0072	0.023	-3.3143
Gm46633	2.0655	-3.7432	3.2336	0.0072	0.0231	-2.2437
Zfp874b	0.6008	3.2105	3.233	0.0072	0.0231	-2.9294
Ndufv3	0.4818	5.9922	3.2322	0.0072	0.0231	-3.3228
Fkbp1a	-0.4781	8.5842	-3.2322	0.0072	0.0231	-3.3782
Gm35857	-1.8701	-1.6478	-3.2315	0.0072	0.0231	-2.2248
Hspd1-ps5	-1.985	-2.7097	-3.2303	0.0072	0.0232	-2.2324
Nnt	0.798	2.2421	3.2299	0.0073	0.0232	-2.7421
Gm36298	1.6185	-0.059	3.2297	0.0073	0.0232	-2.3367
Usp43	-1.9883	2.3812	-3.2296	0.0073	0.0232	-2.5932
Ap1g1	0.4272	7.4833	3.2296	0.0073	0.0232	-3.3691
Cpox	-0.5249	4.8452	-3.229	0.0073	0.0232	-3.1797
Zdhhc18	-0.4829	5.1935	-3.2287	0.0073	0.0232	-3.2404
E130317F20Rik	0.8069	1.5049	3.2286	0.0073	0.0232	-2.6
Lmod1	2.2718	-2.6146	3.2283	0.0073	0.0232	-2.2273
Fiz1	0.4935	4.8602	3.2282	0.0073	0.0232	-3.2108
Gm5148	2.5242	-2.725	3.228	0.0073	0.0232	-2.245
Rgs2	1.856	3.4728	3.2277	0.0073	0.0232	-2.9199
Vgf	-1.2838	-0.4376	-3.2271	0.0073	0.0233	-2.3287
Fry	3.739	0.3939	3.2267	0.0073	0.0233	-2.308
Apc	0.5773	7.2324	3.2264	0.0073	0.0233	-3.3704
Gm20522	1.3497	-0.6793	3.2261	0.0073	0.0233	-2.327
Cldn23	1.0272	-0.9356	3.2258	0.0073	0.0233	-2.3158
Phka1	0.4424	5.0297	3.2254	0.0073	0.0233	-3.2456
Tmem8	0.7319	1.8404	3.2245	0.0073	0.0234	-2.6979
Mef2b	1.6065	-0.7612	3.2241	0.0073	0.0234	-2.2807
Ino80b	0.4508	4.0787	3.2227	0.0074	0.0234	-3.1079
Lysmd3	-0.7557	5.2993	-3.222	0.0074	0.0234	-3.253
Gm37500	2.263	-3.5183	3.2211	0.0074	0.0235	-2.262
Rprd1a	-0.3396	5.2787	-3.2201	0.0074	0.0235	-3.2646
1110032A03Rik	0.7352	2.8526	3.2199	0.0074	0.0235	-2.8842
Zfp235	0.5494	2.5075	3.2199	0.0074	0.0235	-2.823
Hps3	-0.6031	4.0424	-3.2194	0.0074	0.0235	-3.0809
Gm15500	-0.4864	3.5123	-3.2187	0.0074	0.0236	-2.9938
Gm10120	-1.7196	-1.571	-3.2186	0.0074	0.0236	-2.2567

Brd9	0.4196	5.3243	3.218	0.0074	0.0236	-3.2895
Srp19	-0.4434	6.2439	-3.2179	0.0074	0.0236	-3.3542
Btbd6	0.6379	3.6318	3.2173	0.0074	0.0236	-3.0217
Cep295	-0.7597	5.9027	-3.216	0.0074	0.0237	-3.3245
Smim15	-0.3754	5.7463	-3.2151	0.0075	0.0237	-3.3276
Myo19	-0.8163	4.3364	-3.2151	0.0075	0.0237	-3.0965
Adam8	-1.3396	3.4642	-3.2149	0.0075	0.0237	-2.9142
Sectm1a	2.6669	-1.971	3.2144	0.0075	0.0237	-2.2489
Gm38973	-2.3398	-1.8553	-3.214	0.0075	0.0237	-2.2493
Ephx1	1.9636	3.5164	3.2139	0.0075	0.0237	-2.9416
Pcolce2	-1.4196	5.1233	-3.2138	0.0075	0.0237	-3.1834
Imp4	-0.3718	6.2633	-3.2128	0.0075	0.0238	-3.365
Gm29125	2.3032	-3.2451	3.2127	0.0075	0.0238	-2.2588
2900042K21Rik	-2.5081	-2.4079	-3.2119	0.0075	0.0238	-2.2599
Ascc2	0.3205	6.6872	3.2113	0.0075	0.0238	-3.3877
Klf11	1.2722	3.3316	3.2111	0.0075	0.0238	-2.9664
Gm39318	1.5361	-2.1212	3.211	0.0075	0.0238	-2.2606
Phospho2	0.4928	4.2061	3.2105	0.0075	0.0238	-3.1415
03-sept	0.9467	2.7286	3.2103	0.0075	0.0238	-2.8522
Gm49891	-1.1687	-0.4814	-3.2103	0.0075	0.0238	-2.3699
E430018J23Rik	0.6982	2.1372	3.2102	0.0075	0.0238	-2.7573
Lpcat2	1.4423	0.6926	3.21	0.0075	0.0238	-2.5023
Pus3	-0.6274	4.5687	-3.2099	0.0075	0.0238	-3.1806
Cyb5rl	0.8215	2.2428	3.2093	0.0075	0.0239	-2.7601
Arpc4	-0.3734	7.2813	-3.2084	0.0075	0.0239	-3.4034
AI467606	-1.9768	-1.481	-3.2081	0.0076	0.0239	-2.2621
Atp6v1a	0.6812	7.716	3.2081	0.0076	0.0239	-3.4139
Sppl3	0.3251	6.1762	3.2077	0.0076	0.0239	-3.375
Csrnp2	0.6685	3.8388	3.2075	0.0076	0.0239	-3.0812
Tbp	-0.356	5.0436	-3.2074	0.0076	0.0239	-3.2664
Gm16755	-1.1816	-0.4703	-3.2065	0.0076	0.024	-2.3708
Ccdc61	-0.4913	3.8089	-3.206	0.0076	0.024	-3.0676
Hexim1	0.5547	6.3707	3.2054	0.0076	0.024	-3.387
Gm28902	-2.1206	-2.8216	-3.2048	0.0076	0.024	-2.2786
Lyrm2	0.6789	2.9112	3.2047	0.0076	0.024	-2.9445
Cabyr	2.5779	-1.2201	3.2038	0.0076	0.0241	-2.2826
Cab39l	-0.4696	5.292	-3.2033	0.0076	0.0241	-3.3017
Coq10a	0.5103	3.0772	3.2032	0.0076	0.0241	-2.9591
Top1	-0.3754	8.0295	-3.2032	0.0076	0.0241	-3.4241
Cpd	-0.531	6.9757	-3.2029	0.0076	0.0241	-3.4059
Arap1	0.6476	4.0676	3.2024	0.0076	0.0241	-3.1427
Zfp560	0.6601	3.1934	3.202	0.0076	0.0241	-2.9662
AC131675.2	-1.3854	0.4104	-3.2019	0.0076	0.0241	-2.404
Zfp945	0.5577	3.4831	3.2018	0.0076	0.0241	-3.0226
Stx1a	-0.7028	2.6139	-3.2007	0.0077	0.0242	-2.8165
Dync1i2	0.405	6.9605	3.2006	0.0077	0.0242	-3.4145
Rab17	1.6852	-0.0237	3.1992	0.0077	0.0242	-2.4323
Serpinb6a	-0.5943	7.8649	-3.1991	0.0077	0.0242	-3.4305
N4bp2	0.8104	4.9929	3.1989	0.0077	0.0242	-3.2692
Nedd4l	0.6365	5.4424	3.1987	0.0077	0.0242	-3.3407
Pex1	0.5607	3.9748	3.1981	0.0077	0.0242	-3.1276
Acvr1	0.5188	5.5727	3.198	0.0077	0.0242	-3.3503

Ttll12	-0.4846	6.8301	-3.1971	0.0077	0.0243	-3.414
Faim2	2.4129	-2.2087	3.1971	0.0077	0.0243	-2.2855
Col18a1	-1.1126	9.0581	-3.1966	0.0077	0.0243	-3.447
Tbc1d22a	0.5995	5.088	3.1964	0.0077	0.0243	-3.3097
Gm20300	0.7233	2.1051	3.1958	0.0077	0.0243	-2.8007
Ap4m1	0.5265	4.6776	3.1952	0.0077	0.0243	-3.2562
AA986860	1.0416	0.2092	3.1946	0.0077	0.0244	-2.4908
Pinx1	-0.6988	3.6349	-3.1945	0.0077	0.0244	-3.0397
Fgf18	1.9951	-1.2584	3.1941	0.0078	0.0244	-2.3294
Stx6	0.4856	5.595	3.1933	0.0078	0.0244	-3.3622
Brd2	0.2855	8.6267	3.1923	0.0078	0.0245	-3.4542
Wdr66	2.2396	-1.2404	3.1917	0.0078	0.0245	-2.3107
Gm20036	1.2356	-1.393	3.1916	0.0078	0.0245	-2.3332
Got2-ps1	-1.0809	0.6208	-3.1915	0.0078	0.0245	-2.5256
Gm37254	-0.7993	1.0848	-3.1904	0.0078	0.0245	-2.5774
Gm6579	-2.1855	-1.6609	-3.1903	0.0078	0.0245	-2.2869
Psat1	-0.9341	8.851	-3.1903	0.0078	0.0245	-3.4582
Ptgds	7.0309	0.9574	3.19	0.0078	0.0245	-2.2868
Acot2	1.2036	0.63	3.1892	0.0078	0.0246	-2.5297
Cybc1	0.3392	5.2229	3.1889	0.0078	0.0246	-3.3412
Nudt16	0.7394	2.0497	3.1888	0.0078	0.0246	-2.8142
Eif3c	-0.3454	9.0369	-3.1885	0.0078	0.0246	-3.4644
Sap30	-0.4406	5.5093	-3.1885	0.0078	0.0246	-3.3573
Tesk2	0.8059	2.3931	3.1883	0.0078	0.0246	-2.8541
Lrrfip2	-0.3762	5.6048	-3.1878	0.0078	0.0246	-3.3694
Tnfsfm13	1.821	-0.8157	3.1873	0.0078	0.0246	-2.36
Lrrn4cl	1.728	0.8454	3.1872	0.0078	0.0246	-2.579
Lzic	-0.3806	4.4839	-3.1858	0.0079	0.0247	-3.2202
Gm17383	-1.5567	-0.0386	-3.1854	0.0079	0.0247	-2.3733
Cldn6	-1.2151	-1.3442	-3.185	0.0079	0.0247	-2.3186
Sowahc	-0.8337	3.7325	-3.1839	0.0079	0.0248	-3.0509
Slc25a35	1.2316	1.0306	3.1836	0.0079	0.0248	-2.6015
Ptx3	3.4838	1.4096	3.183	0.0079	0.0248	-2.5091
Cldn12	-0.4585	5.4417	-3.1823	0.0079	0.0248	-3.3513
Chd7	0.5616	5.9942	3.1814	0.0079	0.0248	-3.4098
Gm43011	-1.4197	-1.6438	-3.1813	0.0079	0.0248	-2.3192
Gm11973	1.7967	-0.9797	3.1811	0.0079	0.0249	-2.3507
Gm11874	1.5456	-2.2009	3.1809	0.0079	0.0249	-2.3036
Kif3b	0.4113	5.695	3.1802	0.008	0.0249	-3.3974
Fam162a	-0.8114	7.0604	-3.1799	0.008	0.0249	-3.4501
Suclg1	-0.2877	6.8558	-3.1794	0.008	0.0249	-3.45
Gm7466	-1.1649	-0.6401	-3.1781	0.008	0.025	-2.4113
Cfap20	-0.4594	5.2947	-3.1779	0.008	0.025	-3.3495
Nat9	0.49	3.4504	3.1776	0.008	0.025	-3.0857
2810001G20Rik	0.6463	1.8361	3.1772	0.008	0.025	-2.7628
Ghdc	0.5985	4.051	3.1768	0.008	0.025	-3.1867
Plekhj1	-0.429	4.6545	-3.1756	0.008	0.0251	-3.2635
Sdf2l1	-0.5827	5.5865	-3.1747	0.008	0.0251	-3.385
4930594M22Rik	2.6135	-1.4083	3.1739	0.008	0.0251	-2.3346
Mid2	1.8884	0.9149	3.1734	0.0081	0.0251	-2.5747
Ralgps2	-1.2956	3.9207	-3.1731	0.0081	0.0252	-3.0659
Pdgfrb	3.3501	2.9373	3.1731	0.0081	0.0252	-2.7171

Usp5	-0.2783	7.5738	-3.1726	0.0081	0.0252	-3.4756
Pomp	-0.4339	7.1002	-3.1724	0.0081	0.0252	-3.4666
Sfi1	0.5066	3.3873	3.1722	0.0081	0.0252	-3.0716
Adra1b	1.8546	2.7793	3.1712	0.0081	0.0252	-2.8606
H2afy2	3.166	-2.3656	3.1709	0.0081	0.0252	-2.3275
Gm42928	0.8394	0.6097	3.1705	0.0081	0.0252	-2.6148
Csde1	-0.3015	9.7976	-3.1702	0.0081	0.0253	-3.5029
Eif4a2	-0.3485	7.4138	-3.17	0.0081	0.0253	-3.4771
Gm36283	2.0521	-2.4446	3.17	0.0081	0.0253	-2.3178
Btc	-2.5669	0.6925	-3.1699	0.0081	0.0253	-2.4367
Mfsd7a	1.291	-0.3621	3.1698	0.0081	0.0253	-2.4872
Rida	0.6968	4.4613	3.1696	0.0081	0.0253	-3.2647
Gm13340	0.5073	2.4591	3.1693	0.0081	0.0253	-2.8901
Chchd3	-0.2929	6.5913	-3.1691	0.0081	0.0253	-3.4611
Rfng	0.394	4.4211	3.1684	0.0081	0.0253	-3.2564
Acd	0.4508	5.015	3.1676	0.0081	0.0253	-3.3483
Chmp7	0.4188	5.6586	3.1675	0.0081	0.0253	-3.4163
Wdr53	0.4402	3.3313	3.1675	0.0081	0.0253	-3.0646
Maf1	0.3987	6.474	3.167	0.0081	0.0254	-3.4656
Evpl	2.1133	-1.4155	3.1658	0.0082	0.0254	-2.3345
Fam199x	-0.6813	5.7762	-3.1657	0.0082	0.0254	-3.4016
Ccdc9	-0.3624	4.9495	-3.1656	0.0082	0.0254	-3.337
AC122442.1	1.9588	-0.0832	3.1654	0.0082	0.0254	-2.4565
Idi1	-0.979	6.2969	-3.165	0.0082	0.0254	-3.4404
Gm9517	2.0875	-2.8031	3.1649	0.0082	0.0254	-2.3376
Gm16759	2.907	-2.1209	3.1646	0.0082	0.0254	-2.3283
Taf5l	-0.4289	6.012	-3.1637	0.0082	0.0255	-3.437
Gm12854	0.9649	1.2745	3.1635	0.0082	0.0255	-2.6745
Gm9429	-2.4866	-2.9137	-3.1631	0.0082	0.0255	-2.3456
Ank2	1.8999	2.175	3.1629	0.0082	0.0255	-2.8139
Mpst	0.5849	4.4569	3.1626	0.0082	0.0255	-3.286
Gatb	0.4482	4.2721	3.1621	0.0082	0.0255	-3.2561
Cntnap4	-2.6699	0.5185	-3.1619	0.0082	0.0255	-2.4129
Gstm5	0.5208	5.151	3.1616	0.0082	0.0255	-3.3854
Ptpn12	-0.4212	6.5599	-3.1611	0.0082	0.0256	-3.4721
Rxra	0.5502	6.316	3.1598	0.0083	0.0256	-3.4685
Zbtb33	0.4499	5.5977	3.1597	0.0083	0.0256	-3.4164
Tepsin	0.4859	3.5906	3.1595	0.0083	0.0256	-3.1327
Cops9	0.3605	5.5312	3.1588	0.0083	0.0256	-3.426
Aup1	-0.3704	6.5951	-3.1582	0.0083	0.0257	-3.4812
Rny1	-2.2953	-2.2349	-3.157	0.0083	0.0257	-2.349
Gm37123	-1.1913	0.9149	-3.1566	0.0083	0.0257	-2.6066
Exog	-0.4244	3.9195	-3.156	0.0083	0.0258	-3.171
Mia2	-0.3708	6.6091	-3.1558	0.0083	0.0258	-3.4858
Calr4	2.4814	-0.5242	3.1553	0.0083	0.0258	-2.4225
Dmxl2	0.8544	3.8448	3.1549	0.0083	0.0258	-3.1609
Shroom1	1.5122	2.411	3.1547	0.0083	0.0258	-2.8588
Coq9	0.3352	5.6106	3.1542	0.0083	0.0258	-3.4358
Zfp59	0.631	2.5161	3.154	0.0083	0.0258	-2.9329
Pald1	-0.4069	3.7373	-3.1537	0.0084	0.0258	-3.1254
Med24	-0.3999	6.4579	-3.1535	0.0084	0.0258	-3.4842
Gm22270	-2.5467	-1.8318	-3.1532	0.0084	0.0259	-2.3436

Klhdc10	-0.3325	6.4361	-3.1532	0.0084	0.0259	-3.4842
Zc3h18	-0.41	6.809	-3.1513	0.0084	0.0259	-3.4984
Pon3	1.3702	3.8487	3.1507	0.0084	0.026	-3.1497
Gm9797	-1.158	-0.5974	-3.1505	0.0084	0.026	-2.4118
Fkbp11	-1.4493	3.9744	-3.1499	0.0084	0.026	-3.1126
Taf15	-0.3802	6.877	-3.1497	0.0084	0.026	-3.5034
Amn1	0.4885	3.3673	3.1496	0.0084	0.026	-3.1077
Brcc3	-0.4356	5.3904	-3.1492	0.0084	0.026	-3.4109
Nudt16l1	0.3548	4.9629	3.1484	0.0084	0.026	-3.3802
Egfl7	-0.9458	1.9219	-3.1477	0.0084	0.0261	-2.8009
Frs3	0.7492	1.2683	3.1472	0.0085	0.0261	-2.7287
Ucp2	-0.4334	7.3698	-3.1468	0.0085	0.0261	-3.5197
Gm14277	-1.6679	-1.5211	-3.1468	0.0085	0.0261	-2.3733
Cep295nl	1.4356	0.5797	3.1467	0.0085	0.0261	-2.6094
Igdcc4	1.9614	-0.3865	3.1461	0.0085	0.0261	-2.4352
A930005H10Rik	1.011	2.0834	3.1452	0.0085	0.0262	-2.8664
2300009A05Rik	-0.6382	2.2761	-3.1449	0.0085	0.0262	-2.8709
Ammecr1	-0.7452	5.4053	-3.1448	0.0085	0.0262	-3.4043
Setd1a	-0.4157	5.3571	-3.1448	0.0085	0.0262	-3.4154
Shf	-0.6157	4.124	-3.1447	0.0085	0.0262	-3.2388
Gm12355	-1.032	-1.055	-3.1446	0.0085	0.0262	-2.4288
Gm48194	1.985	-1.5489	3.1444	0.0085	0.0262	-2.3604
Slc35a4	-0.3675	6.5573	-3.1442	0.0085	0.0262	-3.5034
Gm12602	-1.2971	1.2328	-3.1436	0.0085	0.0262	-2.659
Per3	0.6962	2.4942	3.143	0.0085	0.0262	-2.9594
Glrx2	0.3199	4.7954	3.1428	0.0085	0.0262	-3.3645
Coq8a	2.0778	2.0291	3.1426	0.0085	0.0262	-2.7866
Ddx49	-0.3547	5.6482	-3.1424	0.0085	0.0262	-3.4552
Ninl	0.5263	4.6514	3.1423	0.0085	0.0262	-3.339
Champ1	-0.3965	5.9646	-3.1411	0.0086	0.0263	-3.4792
Med23	0.3571	5.2826	3.1411	0.0086	0.0263	-3.428
Kpna4	-0.5724	7.7471	-3.1407	0.0086	0.0263	-3.535
Ubald1	0.4509	5.1174	3.1406	0.0086	0.0263	-3.4085
Hapln4	0.8023	2.146	3.1403	0.0086	0.0263	-2.8719
Capg	-0.4899	7.6839	-3.1389	0.0086	0.0264	-3.5398
Aldoa	-0.3905	10.2647	-3.1388	0.0086	0.0264	-3.5633
Lpcat3	0.7761	5.7627	3.1373	0.0086	0.0264	-3.4804
Fmnl3	-0.6934	6.2974	-3.1364	0.0086	0.0265	-3.5036
Btbd11	-1.5552	3.7607	-3.1357	0.0086	0.0265	-3.0866
Gab2	0.9408	2.6749	3.1341	0.0087	0.0266	-3.0051
Cnnm4	-0.7043	4.4085	-3.1309	0.0087	0.0267	-3.2934
Rps11	-0.4082	9.028	-3.1309	0.0087	0.0267	-3.5712
Ttc14	-0.5811	5.8889	-3.1307	0.0087	0.0267	-3.4856
Ggta1	-0.388	6.1633	-3.1302	0.0087	0.0268	-3.5123
Slc22a4	-0.9658	2.9407	-3.1296	0.0087	0.0268	-3.0033
Gm46447	1.6094	-1.567	3.1294	0.0087	0.0268	-2.4028
Tas1r3	1.3433	-1.4924	3.1292	0.0087	0.0268	-2.4132
Cnp	0.56	4.919	3.1289	0.0087	0.0268	-3.4078
Cyth4	-3.8663	-0.2224	-3.1288	0.0087	0.0268	-2.3853
Hunk	-2.3163	-1.299	-3.1282	0.0088	0.0268	-2.3833
Klhdc8a	2.0445	-0.3936	3.1281	0.0088	0.0268	-2.4792
Il18rap	-2.4568	3.3161	-3.1271	0.0088	0.0269	-2.8933

Tusc2	0.4353	4.0909	3.126	0.0088	0.0269	-3.2878
Adnp	0.5334	3.0422	3.1258	0.0088	0.0269	-3.0671
Pgap2	0.4579	4.115	3.1257	0.0088	0.0269	-3.2761
Dolpp1	-0.4716	3.6988	-3.1256	0.0088	0.0269	-3.1875
Lrp5	-0.3898	6.8979	-3.1252	0.0088	0.027	-3.5495
B230216N24Rik	-1.4267	-0.9133	-3.1252	0.0088	0.027	-2.4311
Cdh24	0.7601	2.1037	3.1248	0.0088	0.027	-2.887
Drc3	0.8333	0.8868	3.1245	0.0088	0.027	-2.7258
Macrod1	0.7401	3.7552	3.1238	0.0088	0.027	-3.2189
Map2	2.2529	2.8245	3.1237	0.0088	0.027	-2.9502
Pex16	0.5168	3.0976	3.1234	0.0088	0.027	-3.103
Igfbp4	-1.7365	8.4755	-3.1233	0.0088	0.027	-3.5742
Uox	-2.978	0.1623	-3.1232	0.0088	0.027	-2.4254
Msantd1	1.7602	-1.6758	3.1231	0.0088	0.027	-2.3983
Gm40578	-2.0691	-2.7935	-3.123	0.0088	0.027	-2.3935
Dchs2	3.3212	-3.3072	3.1226	0.0088	0.027	-2.4087
Ccar2	-0.4273	5.8414	-3.1225	0.0089	0.027	-3.5072
Gemin4	-0.7465	1.1044	-3.1219	0.0089	0.0271	-2.7058
Kat6a	0.4363	6.8945	3.1218	0.0089	0.0271	-3.5569
Gm45718	1.1815	-1.7108	3.1205	0.0089	0.0271	-2.415
Gm16537	2.0938	-1.2131	3.1205	0.0089	0.0271	-2.4301
Rasgrp2	1.7859	-1.543	3.1203	0.0089	0.0271	-2.4264
Hectd1	-0.3802	8.5559	-3.119	0.0089	0.0272	-3.5882
Gm24270	0.7892	4.0113	3.1185	0.0089	0.0272	-3.2711
Gm49741	1.7981	-1.6128	3.1178	0.0089	0.0272	-2.4196
Gm23935	0.7277	9.4029	3.1176	0.0089	0.0272	-3.5983
Nsf	-0.4116	5.9985	-3.1169	0.0089	0.0273	-3.5286
Lage3	-0.3505	4.9905	-3.1164	0.009	0.0273	-3.4314
Scaper	0.5442	4.2654	3.1155	0.009	0.0273	-3.344
Ccdc71	0.2797	5.7723	3.1154	0.009	0.0273	-3.5231
Snrpb2	-0.3729	6.2826	-3.1152	0.009	0.0273	-3.547
C530044C16Rik	-2.9047	-1.6629	-3.1149	0.009	0.0273	-2.4027
AV039307	1.341	-2.0465	3.1147	0.009	0.0273	-2.4224
Arhgap45	-2.2371	0.6366	-3.1145	0.009	0.0274	-2.5601
Kpna3	-0.5241	7.2221	-3.1138	0.009	0.0274	-3.5755
Pdgfa	-0.7402	4.8302	-3.1136	0.009	0.0274	-3.3936
Unc5c	4.6465	-0.6876	3.1128	0.009	0.0274	-2.4109
Snx32	0.739	1.6639	3.1114	0.009	0.0275	-2.8887
Gm43598	2.3283	-2.4368	3.1112	0.009	0.0275	-2.4172
Taf2	0.3469	6.1867	3.1107	0.009	0.0275	-3.5553
Eral1	-0.3742	4.165	-3.1099	0.0091	0.0276	-3.321
Gm20512	-1.8248	-1.1186	-3.1097	0.0091	0.0276	-2.4438
Nlrp12	-2.183	-2.8791	-3.1095	0.0091	0.0276	-2.4159
Rgs4	2.9117	-2.7792	3.1088	0.0091	0.0276	-2.4277
Phykpl	-0.489	3.9485	-3.1086	0.0091	0.0276	-3.2671
Gm48146	-1.3231	-0.9814	-3.1082	0.0091	0.0276	-2.4638
Gnptab	0.3666	5.9686	3.1081	0.0091	0.0276	-3.5476
Zfp704	2.1789	3.2709	3.1081	0.0091	0.0276	-3.0373
Gm45441	1.9984	-2.4862	3.108	0.0091	0.0276	-2.4134
Gm26881	1.3402	-1.5848	3.1072	0.0091	0.0276	-2.4642
Tmem231	0.5062	3.3383	3.1071	0.0091	0.0276	-3.1916
Praf2	0.3215	5.7558	3.107	0.0091	0.0276	-3.5382

Zcchc4	-0.4342	4.556	-3.1067	0.0091	0.0277	-3.3734
Lmo4	0.4085	5.7353	3.1065	0.0091	0.0277	-3.5369
Anapc11	-0.3543	6.2922	-3.106	0.0091	0.0277	-3.5666
Man2a2	0.5994	5.745	3.1058	0.0091	0.0277	-3.5355
Jrk	0.4969	3.8792	3.1053	0.0091	0.0277	-3.2867
Mfn2	0.3967	6.0091	3.1053	0.0091	0.0277	-3.5548
Otud5	0.2933	6.7625	3.1039	0.0092	0.0278	-3.5884
Gm5946	-1.5535	-2.602	-3.1035	0.0092	0.0278	-2.4228
Ctcf	-0.3485	6.794	-3.1029	0.0092	0.0278	-3.5877
Tmem62	0.6473	2.2058	3.1029	0.0092	0.0278	-2.9724
Gdf11	0.7586	4.5358	3.1028	0.0092	0.0278	-3.3989
Xrn1	0.4085	6.0766	3.1011	0.0092	0.0279	-3.5647
Als2cr12	-1.6396	-1.448	-3.1008	0.0092	0.0279	-2.4479
Firre	0.7124	4.1553	3.1007	0.0092	0.0279	-3.3241
Gm22953	0.7897	0.9398	3.1004	0.0092	0.0279	-2.7686
Gm34655	2.003	-2.3207	3.1003	0.0092	0.0279	-2.4256
Tnfrsf1a	-0.3733	6.7648	-3.1002	0.0092	0.0279	-3.5928
Chadl	3.3818	-0.2896	3.1001	0.0092	0.0279	-2.5225
Serpinb1a	3.2605	-2.701	3.0997	0.0092	0.0279	-2.4275
Cacnb2	-1.3282	3.0191	-3.0997	0.0092	0.0279	-3.0355
Mrs2	-0.3913	6.0553	-3.0996	0.0092	0.0279	-3.564
Hacd2	0.3829	5.7004	3.0995	0.0092	0.0279	-3.5422
Gabpb2	-0.3501	7.3574	-3.0987	0.0092	0.028	-3.6073
Alyref2	0.6927	1.6239	3.0982	0.0093	0.028	-2.8645
Slc26a6	0.7763	2.0057	3.0979	0.0093	0.028	-2.9546
Gm15535	2.2278	-2.7432	3.0977	0.0093	0.028	-2.4317
Mks1	1.3213	0.3708	3.0974	0.0093	0.028	-2.6684
Snord17	-1.1326	-1.1206	-3.0961	0.0093	0.0281	-2.4636
2310030G06Rik	2.2945	-2.0548	3.0957	0.0093	0.0281	-2.4474
Gm45133	1.0293	0.9108	3.0956	0.0093	0.0281	-2.7418
Abracl	0.4301	5.2583	3.0952	0.0093	0.0281	-3.5087
Gm44729	-1.4751	-1.7522	-3.0948	0.0093	0.0281	-2.4502
C030015A19Rik	0.7596	1.8582	3.0946	0.0093	0.0281	-2.9187
Abcd3	0.3806	6.1978	3.0945	0.0093	0.0281	-3.5866
Flvcr2	3.5366	0.2472	3.0935	0.0093	0.0282	-2.5062
Gm14488	2.0595	-3.3248	3.0923	0.0094	0.0282	-2.4399
Igtp	1.5639	2.4724	3.0922	0.0094	0.0282	-3.0141
Sccpdh	-0.3789	5.2676	-3.0922	0.0094	0.0282	-3.5119
Rpl18	-0.3834	8.9628	-3.0912	0.0094	0.0283	-3.6441
A530084C06Rik	1.4808	0.0221	3.0908	0.0094	0.0283	-2.6239
Vps36	-0.4026	6.1206	-3.0906	0.0094	0.0283	-3.585
Tmem205	0.8377	2.9118	3.0898	0.0094	0.0283	-3.1369
Mpp2	-1.0169	2.694	-3.0893	0.0094	0.0284	-3.0356
Coprs	0.5891	4.3616	3.0892	0.0094	0.0284	-3.3975
AA467197	-1.4987	2.1479	-3.0884	0.0094	0.0284	-2.9036
Phlda2	-3.6109	1.2687	-3.0884	0.0094	0.0284	-2.5508
Colec11	2.3614	-2.482	3.0884	0.0094	0.0284	-2.4442
BC031181	0.3758	5.4038	3.0879	0.0094	0.0284	-3.5425
Camsap1	0.4687	6.6673	3.0879	0.0094	0.0284	-3.6152
Ptger4	2.3414	-0.7665	3.0874	0.0094	0.0284	-2.5036
Gm48558	2.69	-1.8199	3.087	0.0095	0.0284	-2.4502
Hdac11	1.5078	1.6414	3.0865	0.0095	0.0285	-2.8669

Gm44878	1.3405	-1.606	3.0863	0.0095	0.0285	-2.4714
Col7a1	1.8679	4.1665	3.086	0.0095	0.0285	-3.2845
Gm43136	1.9413	-2.5477	3.0859	0.0095	0.0285	-2.4475
Rpusd1	0.3858	3.8258	3.0854	0.0095	0.0285	-3.3064
Dynlt1-ps1	-1.1262	-0.2654	-3.085	0.0095	0.0285	-2.5743
Rpl36-ps3	-1.573	-1.772	-3.085	0.0095	0.0285	-2.4791
Chpt1	0.7252	4.3692	3.0849	0.0095	0.0285	-3.3853
Cxxc5	-0.8992	4.8651	-3.0847	0.0095	0.0285	-3.4406
Skida1	0.8979	2.3333	3.0843	0.0095	0.0285	-3.0071
Ddah1	-1.3517	3.385	-3.0842	0.0095	0.0285	-3.1086
Fgf7	1.5157	-1.4776	3.0836	0.0095	0.0286	-2.4872
Nod1	1.8486	0.4625	3.0834	0.0095	0.0286	-2.6605
Kbtbd8	-1.1779	2.3593	-3.0833	0.0095	0.0286	-2.9397
5830448L01Rik	0.8124	0.664	3.083	0.0095	0.0286	-2.7372
Eml5	-1.1898	5.0903	-3.0829	0.0095	0.0286	-3.4673
1600012H06Rik	0.3698	5.1658	3.0819	0.0095	0.0286	-3.5241
Psrc1	-0.8993	2.34	-3.0812	0.0096	0.0287	-3.0052
Gm8703	2.365	-1.5973	3.0812	0.0096	0.0287	-2.4647
Gm11870	1.5671	-2.4879	3.0804	0.0096	0.0287	-2.456
Zbtb8os	0.4469	4.1102	3.079	0.0096	0.0288	-3.3773
2410002F23Rik	-0.5293	5.5983	-3.0787	0.0096	0.0288	-3.5574
Snf8	0.2873	5.8147	3.0786	0.0096	0.0288	-3.5969
Yeats4	-0.3568	4.8206	-3.0782	0.0096	0.0288	-3.4726
Gm7389	-1.6754	-1.5167	-3.0776	0.0096	0.0288	-2.4831
Pter	-0.481	4.2807	-3.0763	0.0096	0.0289	-3.3965
Efhd1	2.2513	1.0253	3.0752	0.0097	0.0289	-2.7719
Mmut	0.3312	5.603	3.074	0.0097	0.029	-3.5862
4930519P11Rik	1.6036	-1.5194	3.0738	0.0097	0.029	-2.5024
Pex10	-0.5064	2.6306	-3.0731	0.0097	0.029	-3.0797
0610010K14Rik	-0.8173	1.3356	-3.0724	0.0097	0.0291	-2.8382
Gm26840	2.1168	-2.3118	3.0721	0.0097	0.0291	-2.4708
Pdpr	0.8371	4.5496	3.0721	0.0097	0.0291	-3.4445
Pcnx3	0.3868	6.4492	3.0715	0.0097	0.0291	-3.6392
Trappc13	-0.3961	6.5577	-3.0711	0.0097	0.0291	-3.6379
Rps19bp1	-0.5692	4.9434	-3.0708	0.0097	0.0291	-3.4964
Micu2	0.3858	5.8864	3.0697	0.0098	0.0292	-3.6167
Aar2	0.4943	5.2108	3.0692	0.0098	0.0292	-3.5562
Nipsnap2	0.3775	5.4994	3.0687	0.0098	0.0292	-3.584
Dsn1	-0.5285	4.6471	-3.0684	0.0098	0.0292	-3.4593
Psmc1	-0.3563	8.0055	-3.0682	0.0098	0.0292	-3.6755
Tspan12	1.6714	1.1168	3.0673	0.0098	0.0293	-2.7629
Pdcd11	-0.5288	7.0483	-3.0667	0.0098	0.0293	-3.6585
Ifitm2	0.3701	7.2705	3.066	0.0098	0.0293	-3.6681
Dnah2	-2.7567	-1.8734	-3.0655	0.0098	0.0294	-2.479
Eef1aknmt	-0.3772	5.5467	-3.0652	0.0098	0.0294	-3.5822
Sytl5	3.2192	-1.4718	3.0643	0.0099	0.0294	-2.4885
Fancl	-0.6657	3.2832	-3.0636	0.0099	0.0295	-3.2128
E130311K13Rik	0.6326	3.0582	3.0628	0.0099	0.0295	-3.1936
C77080	-0.3623	6.9988	-3.0626	0.0099	0.0295	-3.6684
Chmp6	-0.5368	5.8152	-3.0618	0.0099	0.0295	-3.6163
5033421B08Rik	1.7858	-1.8707	3.0615	0.0099	0.0295	-2.5009
Cenpo	-0.5403	3.9507	-3.0615	0.0099	0.0295	-3.3474

Hist1h4i	0.8801	2.6451	3.0612	0.0099	0.0296	-3.1032
Slc50a1	0.4095	4.6166	3.0609	0.0099	0.0296	-3.4955
Pcdhga3	2.3371	-2.9529	3.0606	0.0099	0.0296	-2.4923
Ccn5	5.6216	1.1008	3.0597	0.0099	0.0296	-2.5501
AC137605.2	-2.4385	-2.6807	-3.0595	0.0099	0.0296	-2.4908
Arid1a	0.3552	7.021	3.0592	0.01	0.0296	-3.6755
Arhgef12	0.4786	7.4122	3.0589	0.01	0.0296	-3.684
Usp21	0.345	5.3753	3.0574	0.01	0.0297	-3.5964
Mr1	1.1392	3.5948	3.0572	0.01	0.0297	-3.2803
Csf1	1.0961	7.0743	3.0572	0.01	0.0297	-3.6808
Cdc34	-0.3272	5.7133	-3.0571	0.01	0.0297	-3.6196
Ppp1r21	0.3238	5.3505	3.057	0.01	0.0297	-3.5935
Gm8319	-1.4432	-2.0347	-3.0564	0.01	0.0298	-2.5022
Agl	0.6009	5.9969	3.056	0.01	0.0298	-3.6481
Ddx17	0.2924	8.7548	3.0559	0.01	0.0298	-3.708
Smt3h2-ps	-0.7	2.0902	-3.0558	0.01	0.0298	-2.9957
Gm7289	-1.1679	-0.5104	-3.0558	0.01	0.0298	-2.6039
Gdi2	-0.3071	9.0228	-3.0556	0.01	0.0298	-3.7102
Mavs	-0.4507	5.3213	-3.0546	0.01	0.0298	-3.5839
Aif1l	0.5459	5.717	3.0545	0.01	0.0298	-3.6282
Osbpl2	0.5201	5.3258	3.0542	0.01	0.0298	-3.5946
Gm5586	-0.9901	-0.1045	-3.0541	0.01	0.0298	-2.679
Gm32618	1.314	-0.1343	3.054	0.0101	0.0298	-2.6525
P2rx1	2.7049	-2.509	3.0537	0.0101	0.0298	-2.4973
Serpinb6b	3.7655	-0.3139	3.0535	0.0101	0.0299	-2.5247
Pggt1b	0.3501	5.8391	3.0535	0.0101	0.0299	-3.6389
Mboat7	-0.4337	5.2975	-3.0527	0.0101	0.0299	-3.5783
Tpt1-ps3	0.4064	5.1568	3.0524	0.0101	0.0299	-3.5874
Sec31b	1.9049	-1.9241	3.0522	0.0101	0.0299	-2.5205
Slc45a4	0.6392	5.6068	3.0517	0.0101	0.0299	-3.6258
Nfxl1	-0.3934	5.6595	-3.0516	0.0101	0.0299	-3.6222
Atrip	-0.3779	4.4065	-3.0514	0.0101	0.0299	-3.4534
Slc25a27	0.9567	1.0773	3.0513	0.0101	0.0299	-2.8573
Tnfrsf13c	2.418	-1.4831	3.051	0.0101	0.0299	-2.5143
Gm10501	-1.1286	0.005	-3.0504	0.0101	0.03	-2.6701
Snord93	-1.8947	-0.901	-3.0501	0.0101	0.03	-2.5444
Gfra2	0.9745	5.5557	3.0501	0.0101	0.03	-3.6116
Idh3b	-0.2763	7.231	-3.0491	0.0101	0.03	-3.6984
Grap	2.5821	-0.5488	3.0484	0.0102	0.0301	-2.5722
Arl6ip5	0.5083	7.7236	3.0479	0.0102	0.0301	-3.7101
Gm37131	-1.118	-0.0818	-3.047	0.0102	0.0301	-2.6805
3300002I08Rik	0.9572	0.2718	3.0465	0.0102	0.0302	-2.7441
Zyg11b	0.3347	6.1513	3.0459	0.0102	0.0302	-3.6735
Tlcd2	1.6708	-0.6388	3.0452	0.0102	0.0302	-2.6097
Ubr7	-0.3977	5.9958	-3.0449	0.0102	0.0302	-3.658
Zfp395	-0.598	5.951	-3.0446	0.0102	0.0302	-3.6549
Rab31	-0.4179	7.9984	-3.0443	0.0102	0.0303	-3.718
Luc7l3	-0.3914	6.8897	-3.0432	0.0103	0.0303	-3.7
Kctd4	-1.6385	0.0576	-3.0422	0.0103	0.0304	-2.6517
Kctd14	2.7746	-2.6854	3.0418	0.0103	0.0304	-2.5157
Dph2	-0.661	3.9717	-3.0417	0.0103	0.0304	-3.3698
Sox8	2.1852	-2.4868	3.0411	0.0103	0.0304	-2.5189

Myl12b	0.3536	5.9309	3.041	0.0103	0.0304	-3.6729
Gm9790	-1.9645	-2.0953	-3.041	0.0103	0.0304	-2.5197
Pskh1	0.3442	5.0482	3.0409	0.0103	0.0304	-3.5879
Adora2a	2.2565	-3.0929	3.0407	0.0103	0.0304	-2.5177
Gm41442	-1.1762	0.9546	-3.0406	0.0103	0.0304	-2.809
Ubxn8	-0.3828	4.6331	-3.0398	0.0103	0.0304	-3.5075
Pdk3	-0.431	5.9626	-3.0397	0.0103	0.0304	-3.6661
Timm21	-0.4574	4.6172	-3.0396	0.0103	0.0304	-3.5183
Slc38a9	0.7263	3.4974	3.0388	0.0103	0.0305	-3.3183
Tmtc1	2.0911	-1.681	3.0384	0.0103	0.0305	-2.5323
1700087I21Rik	2.1143	-2.3967	3.0384	0.0103	0.0305	-2.5236
Ogfod1	-0.3612	5.4759	-3.038	0.0104	0.0305	-3.6264
Fbxo7	0.4347	3.7132	3.0379	0.0104	0.0305	-3.3815
P3h1	0.3179	5.9134	3.0372	0.0104	0.0306	-3.6755
Foxp4	0.3193	6.591	3.0371	0.0104	0.0306	-3.707
Tulp3	0.3562	6.4469	3.0365	0.0104	0.0306	-3.705
Rcl1	-0.4686	5.9666	-3.0359	0.0104	0.0306	-3.6723
Rnf41	0.3479	5.0899	3.0356	0.0104	0.0306	-3.6042
Zfp110	0.3067	4.8783	3.0356	0.0104	0.0306	-3.5717
Pcid2	-0.4007	5.7042	-3.0356	0.0104	0.0306	-3.6548
Pak3	1.6963	5.5053	3.0354	0.0104	0.0306	-3.6226
Tomm22	-0.4103	7.118	-3.0345	0.0104	0.0307	-3.7222
Pcnp	-0.3962	5.5082	-3.0323	0.0105	0.0308	-3.6379
Kdm8	-0.7832	2.3392	-3.0322	0.0105	0.0308	-3.0713
Cdh1	2.0571	-3.6161	3.0306	0.0105	0.0309	-2.5366
Pten	0.6044	7.4842	3.0302	0.0105	0.0309	-3.7387
F630042J09Rik	3.4932	-3.5168	3.0301	0.0105	0.0309	-2.544
Ergic1	-0.3204	7.2619	-3.029	0.0105	0.0309	-3.7342
Adm2	-1.9335	-1.9636	-3.0288	0.0105	0.031	-2.5509
C430014B12Rik	1.4725	-1.3046	3.0284	0.0105	0.031	-2.6076
Atf3	1.3597	2.9062	3.0284	0.0105	0.031	-3.2202
Ethe1	-0.3971	4.45	-3.0282	0.0105	0.031	-3.5188
Col11a2	1.4462	0.0523	3.0281	0.0105	0.031	-2.7722
Ndufa1	0.3174	5.3326	3.0274	0.0106	0.031	-3.6501
Lrg1	3.3447	-0.6686	3.0266	0.0106	0.0311	-2.5893
Pcdhga11	2.1014	-3.072	3.0262	0.0106	0.0311	-2.5389
D630033O11Ril	-2.6166	-2.28	-3.0254	0.0106	0.0311	-2.5401
Gm11517	1.6628	-1.4295	3.0253	0.0106	0.0311	-2.5884
Arhgap1	0.3071	6.4373	3.025	0.0106	0.0311	-3.7239
Zyx	0.4676	7.8587	3.025	0.0106	0.0311	-3.7546
Golga7	-0.305	6.0818	-3.0248	0.0106	0.0311	-3.7022
Gm10382	1.0235	-0.888	3.0247	0.0106	0.0311	-2.635
C130040N14Ril	-2.6823	-2.7485	-3.0242	0.0106	0.0311	-2.5494
Trim7	1.0804	0.6697	3.0235	0.0106	0.0312	-2.8445
Fam71e1	2.6158	-2.715	3.0232	0.0106	0.0312	-2.5456
Pex13	0.4598	5.6139	3.0231	0.0106	0.0312	-3.678
A430046D13Ril	0.9267	-0.2203	3.0222	0.0107	0.0312	-2.7124
Coro7	0.558	4.1002	3.022	0.0107	0.0312	-3.4834
Tmem131l	0.667	5.8996	3.0215	0.0107	0.0313	-3.6987
Mpnd	0.3893	6.1804	3.0214	0.0107	0.0313	-3.7221
Borcs6	0.467	3.9906	3.0211	0.0107	0.0313	-3.4768
Psmd6	-0.3447	6.8915	-3.0206	0.0107	0.0313	-3.7436

AC241534.1	1.4147	-1.0583	3.0197	0.0107	0.0314	-2.6197
H3f3a-ps1	0.7684	1.3823	3.0192	0.0107	0.0314	-2.9429
Hint3	0.5657	2.4935	3.0179	0.0107	0.0314	-3.1928
Morc3	-0.4053	5.8169	-3.0178	0.0107	0.0314	-3.6949
Gm15564	0.5843	5.8755	3.0176	0.0108	0.0315	-3.7069
Hdac1	-0.3359	6.5534	-3.0175	0.0108	0.0315	-3.7399
Fignl2	1.3028	1.6871	3.0173	0.0108	0.0315	-3.0186
Inpp5j	2.8093	-2.5965	3.016	0.0108	0.0315	-2.5592
R3hcc1	0.5933	4.0469	3.0157	0.0108	0.0315	-3.4763
Kdm4d	1.7411	-1.7197	3.0155	0.0108	0.0315	-2.6155
Sirt7	0.4265	4.9454	3.0151	0.0108	0.0316	-3.6308
Zfp551	0.9676	-0.1258	3.0148	0.0108	0.0316	-2.7674
Clptm1	0.2746	6.8887	3.0147	0.0108	0.0316	-3.7556
Ginm1	0.3545	4.2926	3.0145	0.0108	0.0316	-3.522
Mrgpre	1.3874	0.2537	3.014	0.0108	0.0316	-2.8045
Tmem202	1.3316	-0.5427	3.0137	0.0108	0.0316	-2.6805
Zc3h7b	0.3095	7.4149	3.013	0.0108	0.0317	-3.7686
Daam2	5.9873	-0.8124	3.0127	0.0109	0.0317	-2.5628
Man1c1	-0.9495	4.8891	-3.0123	0.0109	0.0317	-3.5766
Gatm	-1.4679	4.4847	-3.0123	0.0109	0.0317	-3.4584
Nt5c3b	-0.5583	5.4754	-3.0122	0.0109	0.0317	-3.67
Gm5637	-1.0779	0.3247	-3.0105	0.0109	0.0318	-2.8044
Dipk1a	0.8873	4.3615	3.0102	0.0109	0.0318	-3.5363
Zc2hc1c	0.7317	0.8995	3.0099	0.0109	0.0318	-2.9388
Pak6	-1.1276	2.4017	-3.0097	0.0109	0.0318	-3.123
Cd24a	1.8957	6.9779	3.0087	0.0109	0.0319	-3.7665
Elmod2	0.4639	4.522	3.0084	0.0109	0.0319	-3.5719
Cnst	0.4264	4.1379	3.0082	0.0109	0.0319	-3.5059
BC085271	-0.9629	-0.6546	-3.008	0.0109	0.0319	-2.6682
Far1	-0.4728	6.8069	-3.0073	0.011	0.0319	-3.7629
Haus7	-0.5682	4.8878	-3.0061	0.011	0.032	-3.6131
9930004E17Rik	1.967	-2.7665	3.0061	0.011	0.032	-2.5729
Thnsl2	2.6205	-2.6458	3.0058	0.011	0.032	-2.5775
Gm8773	1.3959	-0.9187	3.0054	0.011	0.032	-2.6598
Asb6	-0.3387	5.0889	-3.0053	0.011	0.032	-3.6421
Gm10419	-1.7224	-2.1206	-3.0053	0.011	0.032	-2.5788
Lrrc27	0.7067	1.7221	3.0053	0.011	0.032	-3.0706
Kat2b	0.4867	5.8548	3.0049	0.011	0.032	-3.7316
Pop7	-0.392	3.7617	-3.0042	0.011	0.0321	-3.4297
Lmf1	0.6896	3.719	3.0037	0.011	0.0321	-3.4487
Rock2	-0.3756	7.9015	-3.0037	0.011	0.0321	-3.792
Arhgdia	-0.3017	9.4624	-3.0035	0.011	0.0321	-3.8095
Tollip	0.3464	6.3294	3.0032	0.011	0.0321	-3.7617
Tbk1	0.304	5.4704	3.0027	0.0111	0.0321	-3.7035
Cmtm7	-0.4228	4.9909	-3.0024	0.0111	0.0321	-3.6333
2310058D17Rik	1.1663	-0.4406	3.0019	0.0111	0.0322	-2.7019
6430548M08Rik	1.0158	4.1744	3.0017	0.0111	0.0322	-3.5133
Il23a	-1.7947	-1.2791	-3.0017	0.0111	0.0322	-2.5994
Reep3	0.3743	6.713	3.0015	0.0111	0.0322	-3.7749
Tert	-0.567	2.8332	-3.0012	0.0111	0.0322	-3.253
Rab43	0.8909	1.5463	3.0011	0.0111	0.0322	-3.0434
Adamtsl2	4.105	-3.1928	3.0009	0.0111	0.0322	-2.5896

Nme6	-0.5124	3.1075	-3.0004	0.0111	0.0322	-3.293
Gm7324	-1.0299	-0.494	-3	0.0111	0.0322	-2.719
Spin4	-0.7225	1.7774	-2.9982	0.0111	0.0323	-3.0443
Gm47400	2.3469	-2.873	2.9982	0.0111	0.0323	-2.5818
Gm3336	2.7382	-3.0455	2.9979	0.0112	0.0323	-2.5891
Ang	1.9703	-1.7599	2.9973	0.0112	0.0324	-2.6062
Ints6	-0.4641	5.571	-2.9969	0.0112	0.0324	-3.7109
Ube2i	-0.311	7.4032	-2.9966	0.0112	0.0324	-3.7973
Mapkap1	0.282	6.0521	2.9962	0.0112	0.0324	-3.7584
Trappc4	-0.3746	5.7931	-2.9941	0.0112	0.0325	-3.7433
Phgdh	0.4928	8.672	2.9939	0.0112	0.0325	-3.8225
Qser1	-0.4009	6.5653	-2.993	0.0113	0.0326	-3.7813
Fbxl21	-0.7511	1.974	-2.9918	0.0113	0.0327	-3.087
Nadk	-0.3707	7.4315	-2.9914	0.0113	0.0327	-3.8073
Gm16279	1.8198	-1.929	2.9905	0.0113	0.0327	-2.5948
Zc3hav1l	0.4577	3.3978	2.9903	0.0113	0.0327	-3.3862
Atp5g1	-0.3574	6.3785	-2.9895	0.0113	0.0328	-3.7853
Tmem53	1.875	0.3097	2.989	0.0113	0.0328	-2.848
Josd1	-0.3844	6.551	-2.9881	0.0114	0.0329	-3.7928
Nop10	-0.51	5.93	-2.988	0.0114	0.0329	-3.7596
C9orf72	0.4727	4.5457	2.9878	0.0114	0.0329	-3.6144
C130012C08Rik	1.3073	-1.8238	2.9872	0.0114	0.0329	-2.619
Got1	-0.4137	6.2693	-2.9866	0.0114	0.0329	-3.7862
Rpl14	-0.3485	9.3265	-2.9865	0.0114	0.0329	-3.8401
Rap2b	0.3717	6.1519	2.9859	0.0114	0.033	-3.7811
Homer3	0.6622	5.4944	2.9857	0.0114	0.033	-3.74
Sdc4	-0.8292	8.8997	-2.985	0.0114	0.033	-3.8373
Galnt12	-1.4701	0.7774	-2.9848	0.0114	0.033	-2.8589
Gm9626	2.101	-3.0106	2.9838	0.0114	0.0331	-2.6038
Unk	0.3505	5.0724	2.9834	0.0115	0.0331	-3.6963
Amot	1.1018	5.7457	2.9826	0.0115	0.0331	-3.7475
Setdb2	-0.5049	3.5267	-2.9825	0.0115	0.0331	-3.4129
Pank2	-0.369	5.6223	-2.9823	0.0115	0.0331	-3.7488
Zfp398	0.5842	3.6553	2.981	0.0115	0.0332	-3.456
Sipa1l1	0.3748	6.9978	2.9806	0.0115	0.0332	-3.8211
Mark4	0.3899	5.2627	2.9805	0.0115	0.0332	-3.7268
Eif4g3	0.3426	5.843	2.9805	0.0115	0.0332	-3.7764
S100a6	-0.3227	11.2381	-2.9802	0.0115	0.0332	-3.8597
Mcfd2	-0.4127	6.6703	-2.9802	0.0115	0.0332	-3.8115
Gpt	0.8894	2.2546	2.98	0.0115	0.0332	-3.189
Acbd5	0.4357	5.5792	2.9798	0.0115	0.0332	-3.7512
Ubr4	0.537	8.1016	2.9797	0.0115	0.0332	-3.84
Leng9	0.662	1.1529	2.9796	0.0115	0.0332	-3.041
Tmem123	0.8413	7.9676	2.9789	0.0116	0.0333	-3.8406
Swt1	-0.4008	4.3588	-2.9789	0.0116	0.0333	-3.5773
Rmnd1	0.3719	3.8432	2.9782	0.0116	0.0333	-3.5203
Thoc7	-0.3533	6.02	-2.9781	0.0116	0.0333	-3.7869
Gm39526	-2.1747	-2.4247	-2.9779	0.0116	0.0333	-2.6143
Cyp39a1	1.1903	1.8431	2.9773	0.0116	0.0333	-3.1019
Apex1	-0.4488	7.2244	-2.9773	0.0116	0.0333	-3.8299
Tent4b	-0.5071	6.0466	-2.977	0.0116	0.0334	-3.7833
Ccdc186	0.4658	5.1445	2.9761	0.0116	0.0334	-3.7153

Birc3	1.1196	5.211	2.9756	0.0116	0.0334	-3.7159
1110051M20Rik	0.7801	2.9734	2.9753	0.0116	0.0334	-3.3503
Trmt5	-0.3637	4.31	-2.975	0.0116	0.0335	-3.5859
Cdk20	-0.5057	3.268	-2.975	0.0116	0.0335	-3.3781
Mapk1ip1l	-0.3057	7.1903	-2.9748	0.0116	0.0335	-3.8332
Arrb2	-0.3722	4.9544	-2.9748	0.0116	0.0335	-3.685
Ssc5d	2.3448	0.5967	2.9746	0.0116	0.0335	-2.7954
Arf1	-0.2795	8.9006	-2.9745	0.0116	0.0335	-3.8589
Spata6	0.6605	3.408	2.9744	0.0116	0.0335	-3.4373
Tmem126a	-0.4187	5.3738	-2.9737	0.0117	0.0335	-3.7377
Pfdn5	0.5982	6.4301	2.9733	0.0117	0.0335	-3.8212
5730455P16Rik	0.3335	4.9145	2.9729	0.0117	0.0335	-3.6959
Anxa2	-0.473	10.0512	-2.9723	0.0117	0.0336	-3.87
Abcd2	1.9934	0.047	2.9717	0.0117	0.0336	-2.7916
Nxf1	-0.4112	7.0938	-2.9713	0.0117	0.0336	-3.8382
Zfp277	-0.3093	5.1115	-2.971	0.0117	0.0336	-3.7184
Supt20	-0.2762	6.1126	-2.9705	0.0117	0.0337	-3.8086
Dffa	0.4736	3.9299	2.9694	0.0118	0.0337	-3.5488
Trap1	-0.3578	7.0005	-2.9689	0.0118	0.0337	-3.8408
Tpi-rs10	-1.9619	-2.2139	-2.9686	0.0118	0.0338	-2.6409
Epdr1	-0.5582	5.648	-2.9682	0.0118	0.0338	-3.7717
Letm2	0.8175	0.7439	2.9681	0.0118	0.0338	-2.9457
Gm16061	-1.4221	-1.6134	-2.968	0.0118	0.0338	-2.6826
Prkaca	-0.3356	7.1439	-2.9659	0.0118	0.0339	-3.8487
Il11	-3.6479	0.516	-2.9654	0.0118	0.0339	-2.6996
Gm44250	1.383	3.832	2.9634	0.0119	0.0341	-3.5064
Tigit	-2.4495	0.9251	-2.9618	0.0119	0.0342	-2.848
Pnpla6	0.615	5.6765	2.961	0.0119	0.0342	-3.7961
1810062O18Rik	1.8912	-1.2041	2.9606	0.012	0.0342	-2.7122
Masp1	4.8692	1.9431	2.96	0.012	0.0343	-2.8136
Gm45838	1.6821	-3.2543	2.9598	0.012	0.0343	-2.6417
Gm4813	-2.7431	0.2895	-2.9598	0.012	0.0343	-2.7199
Maml3	0.3622	6.0255	2.9594	0.012	0.0343	-3.8269
Zbtb46	0.883	3.2014	2.9591	0.012	0.0343	-3.4227
Klra2	-1.8816	1.0595	-2.9586	0.012	0.0343	-2.887
Zfp389	1.7365	-2.1433	2.9583	0.012	0.0343	-2.6666
Zfp85	0.7545	1.9395	2.9578	0.012	0.0344	-3.1707
Gm15413	1.6615	-2.9757	2.9572	0.012	0.0344	-2.6587
Stxbp2	0.5479	4.4182	2.9568	0.012	0.0344	-3.6537
Gm7729	-0.6038	2.4174	-2.9565	0.012	0.0344	-3.2505
Pou2af1	-2.7468	-2.617	-2.9556	0.0121	0.0345	-2.648
Rad17	-0.36	5.4966	-2.9554	0.0121	0.0345	-3.7815
Sox9	0.6469	7.598	2.9553	0.0121	0.0345	-3.8802
Gga3	-0.4033	5.2337	-2.9552	0.0121	0.0345	-3.7552
Col20a1	2.3553	-1.3539	2.9548	0.0121	0.0345	-2.6762
Dcaf4	0.4788	4.2434	2.9532	0.0121	0.0346	-3.6348
Usp33	0.3353	6.2767	2.9529	0.0121	0.0346	-3.8504
Dsel	0.8619	3.7971	2.9527	0.0121	0.0346	-3.5073
Ttc12	2.2908	0.4214	2.9524	0.0121	0.0346	-2.8458
Zfp189	0.6159	2.8027	2.9519	0.0121	0.0347	-3.3472
Slc16a6	-2.2552	1.1862	-2.9516	0.0122	0.0347	-2.8768
Rwdd2b	0.4173	3.2243	2.9514	0.0122	0.0347	-3.4324

Csnk2b	-0.3049	6.9578	-2.9512	0.0122	0.0347	-3.8724
Rtp4	2.91	0.3915	2.9511	0.0122	0.0347	-2.8346
Abca7	0.7363	4.6674	2.9511	0.0122	0.0347	-3.6923
Gm11696	1.1725	-0.3089	2.9511	0.0122	0.0347	-2.8593
Grhpr	-0.4941	4.5991	-2.9504	0.0122	0.0347	-3.6859
Gm6286	-1.8337	-1.6426	-2.9504	0.0122	0.0347	-2.679
Zfp790	0.3934	3.5685	2.9501	0.0122	0.0347	-3.5161
Gm40117	-1.9059	-1.03	-2.9492	0.0122	0.0348	-2.6991
Elmo2	-0.4025	6.132	-2.949	0.0122	0.0348	-3.8475
Tmem121	-1.0999	0.0466	-2.9485	0.0122	0.0348	-2.8613
Hnrnpdl	-0.3107	7.4015	-2.9481	0.0122	0.0348	-3.8854
Ash1l	0.394	7.3444	2.948	0.0122	0.0348	-3.8858
Zfp469	2.6018	0.5258	2.9472	0.0123	0.0349	-2.8326
Lrrc14	0.487	4.4962	2.9457	0.0123	0.035	-3.6784
Bnip2	-0.2303	7.4613	-2.9452	0.0123	0.035	-3.8933
Foxred1	-0.3479	4.3719	-2.9449	0.0123	0.035	-3.6501
Meis3	1.1207	2.2267	2.9445	0.0123	0.035	-3.2468
Trmt2a	-0.3972	5.4674	-2.9438	0.0123	0.0351	-3.8032
Sesn2	-0.4361	4.8449	-2.9437	0.0123	0.0351	-3.7294
Dhx33	-0.4107	6.0537	-2.943	0.0123	0.0351	-3.8469
Dvl2	0.325	5.8626	2.9425	0.0124	0.0351	-3.8485
4930480K23Rik	1.2469	-1.3928	2.9424	0.0124	0.0351	-2.7475
Actl6a	-0.534	6.9545	-2.9417	0.0124	0.0352	-3.888
Zfp622	0.4534	5.7964	2.9412	0.0124	0.0352	-3.846
Mff	0.2447	6.3754	2.9409	0.0124	0.0352	-3.8774
Enthd1	-3.2179	-2.8543	-2.9408	0.0124	0.0352	-2.673
Slc25a32	-0.6103	5.0887	-2.9401	0.0124	0.0353	-3.7462
Zfp772	0.6146	2.125	2.94	0.0124	0.0353	-3.2422
Pelo	-0.4171	4.4036	-2.9397	0.0124	0.0353	-3.6684
Tgif1	-0.5401	6.7248	-2.9391	0.0124	0.0353	-3.8884
Mrgprd	2.4866	-3.5738	2.939	0.0124	0.0353	-2.675
Dyrk2	0.3446	5.2025	2.9385	0.0125	0.0353	-3.7864
Dld	-0.3264	7.316	-2.938	0.0125	0.0353	-3.9037
Gm11714	-2.5135	-1.9536	-2.9366	0.0125	0.0354	-2.6802
1110013H19Rik	1.6584	-1.709	2.9357	0.0125	0.0355	-2.7282
Emc2	0.3632	5.4983	2.9354	0.0125	0.0355	-3.8339
Hdac8	0.5028	3.9072	2.934	0.0126	0.0356	-3.6103
Gm11889	-2.0376	-2.234	-2.9337	0.0126	0.0356	-2.6805
Rad52	-0.5361	3.4693	-2.9336	0.0126	0.0356	-3.5033
Fam160a2	-0.3487	5.0462	-2.9334	0.0126	0.0356	-3.7701
Srsf6	-0.3554	8.0551	-2.9333	0.0126	0.0356	-3.923
1700021F05Rik	-0.3707	4.0839	-2.9324	0.0126	0.0357	-3.6209
2900060N12Rik	1.9198	-3.2096	2.9321	0.0126	0.0357	-2.6816
Gm6525	-1.9394	-0.3364	-2.9321	0.0126	0.0357	-2.7691
Sars	-0.2953	8.2813	-2.9319	0.0126	0.0357	-3.9313
Lpgat1	-0.4587	7.4117	-2.9318	0.0126	0.0357	-3.9152
Gm26542	2.5719	-1.5174	2.9312	0.0126	0.0357	-2.7076
Ranbp9	0.3006	6.5908	2.9312	0.0126	0.0357	-3.9011
Ccbe1	-1.7088	3.9818	-2.9307	0.0126	0.0357	-3.4524
Scarf2	0.739	4.9735	2.9306	0.0126	0.0357	-3.7656
Gpr157	1.5829	-1.3558	2.9305	0.0126	0.0357	-2.7508
Rnpc3	-0.4399	4.1913	-2.9304	0.0126	0.0357	-3.6198

Gm26849	2.1652	-3.8552	2.93	0.0126	0.0358	-2.6892
Atn1	0.4949	3.7715	2.9299	0.0127	0.0358	-3.5772
Gm10762	0.6202	1.1028	2.9294	0.0127	0.0358	-3.0925
Icam5	1.0504	1.2077	2.9288	0.0127	0.0358	-3.0556
Rbm3-ps	-1.1768	0.2591	-2.9287	0.0127	0.0358	-2.8897
Gm24967	-1.3525	-2.2893	-2.9286	0.0127	0.0358	-2.6967
Ccdc73	1.0702	-0.536	2.9285	0.0127	0.0358	-2.8515
Gm40309	-1.7255	-1.8439	-2.9281	0.0127	0.0359	-2.6991
Trim63	3.7589	-1.4648	2.9277	0.0127	0.0359	-2.6898
Kpna2-ps	1.2084	-0.8726	2.9268	0.0127	0.0359	-2.8298
Prelid3a	-0.8747	1.444	-2.9259	0.0127	0.036	-3.1265
Mau2	0.4015	6.253	2.9257	0.0128	0.036	-3.9
Hmgn3	0.4157	5.0342	2.9244	0.0128	0.0361	-3.8029
Gm9386	-2.2588	-2.7432	-2.9238	0.0128	0.0361	-2.695
Tor2a	-0.3457	4.8491	-2.9237	0.0128	0.0361	-3.7624
Lpin2	-0.3347	5.6783	-2.9233	0.0128	0.0361	-3.8584
Aspa	1.167	3.0644	2.9222	0.0128	0.0362	-3.4191
Spag9	0.3347	7.5363	2.922	0.0128	0.0362	-3.9385
Afap1l2	2.5869	3.015	2.9201	0.0129	0.0363	-3.2935
Zscan12	0.33	3.951	2.9199	0.0129	0.0363	-3.6328
D6Ertd527e	0.9981	-0.4399	2.9199	0.0129	0.0363	-2.8674
Scarna2	1.8696	-0.8948	2.9196	0.0129	0.0363	-2.7568
Thap4	-0.2752	5.6812	-2.9195	0.0129	0.0363	-3.872
Myo5c	1.9376	-1.9328	2.9181	0.0129	0.0364	-2.7209
St14	1.8485	-0.9814	2.9178	0.0129	0.0364	-2.7598
Tspyl4	1.2349	1.2225	2.9172	0.013	0.0365	-3.1231
Commd8	0.362	5.6948	2.9146	0.013	0.0366	-3.8854
Epop	-0.8764	3.8957	-2.9146	0.013	0.0366	-3.5876
Plet1	-3.2252	0.7821	-2.9139	0.013	0.0367	-2.8309
Exoc6b	0.4377	6.0142	2.9127	0.0131	0.0368	-3.9108
Gm38317	1.5762	-2.4143	2.9125	0.0131	0.0368	-2.7293
Fbxl13	1.9181	-2.7189	2.9125	0.0131	0.0368	-2.7168
Mlx	-0.3987	5.9	-2.9125	0.0131	0.0368	-3.8982
Bbs9	0.6895	4.3252	2.912	0.0131	0.0368	-3.7199
Spata9	2.1336	-2.4297	2.911	0.0131	0.0368	-2.7199
Atp5md	-0.3057	6.5636	-2.9104	0.0131	0.0369	-3.9386
Rpp14	-0.4843	5.0501	-2.9103	0.0131	0.0369	-3.8072
Lmf2	-0.3589	6.5301	-2.91	0.0131	0.0369	-3.9354
Hnrnpul1	-0.2758	7.9485	-2.91	0.0131	0.0369	-3.9655
C2cd3	0.4509	5.5374	2.9091	0.0131	0.0369	-3.8789
Gm567	2.1448	-0.2804	2.909	0.0132	0.0369	-2.8515
Zfp365	-0.7133	3.3212	-2.9089	0.0132	0.0369	-3.5088
Dph6	-0.4057	5.4015	-2.908	0.0132	0.037	-3.8568
Rgs8	-1.812	-2.7816	-2.9075	0.0132	0.037	-2.7187
Gnl3l	-0.4081	7.177	-2.9073	0.0132	0.037	-3.957
Zfp846	0.6025	3.1332	2.9072	0.0132	0.037	-3.5029
Kmt2e	0.5341	6.9609	2.9071	0.0132	0.037	-3.955
1700123O20Rik	0.3447	4.8731	2.9065	0.0132	0.0371	-3.8081
Cemip2	0.4201	6.1142	2.9062	0.0132	0.0371	-3.9267
Tm4sf4	-1.8388	-3.2785	-2.9052	0.0132	0.0371	-2.7247
Igsf9	1.8493	2.0098	2.9051	0.0132	0.0371	-3.2001
Gm8228	-1.245	-1.5892	-2.905	0.0132	0.0371	-2.7842

Naca	-0.3031	8.6745	-2.9048	0.0133	0.0372	-3.9849
Zeb2	0.5153	5.5229	2.9044	0.0133	0.0372	-3.8843
Adam4	1.935	-2.4177	2.9043	0.0133	0.0372	-2.7304
Rpl27	-0.3589	8.0249	-2.9038	0.0133	0.0372	-3.9787
Pmm2	-0.3216	5.2991	-2.9029	0.0133	0.0373	-3.8568
Maoa	0.9481	6.4624	2.9027	0.0133	0.0373	-3.9515
Cdc37	-0.2917	8.4822	-2.9026	0.0133	0.0373	-3.9867
Dusp16	0.6528	5.5981	2.9026	0.0133	0.0373	-3.8966
Hist1h2be	2.3505	0.3189	2.9023	0.0133	0.0373	-2.9096
Cfap100	1.8643	-2.5939	2.9022	0.0133	0.0373	-2.7336
Eif3m	-0.3269	7.4031	-2.9022	0.0133	0.0373	-3.9713
Acox1	0.4685	7.6719	2.9019	0.0133	0.0373	-3.9785
Casc1	1.7285	-0.8382	2.9018	0.0133	0.0373	-2.8415
Mgst2	-0.4112	5.5805	-2.9015	0.0133	0.0373	-3.8936
Syde2	-0.534	4.8117	-2.9008	0.0134	0.0373	-3.7905
Tmem161a	-0.3131	5.1688	-2.9007	0.0134	0.0374	-3.8547
Lacc1	-0.8137	3.8068	-2.9004	0.0134	0.0374	-3.5914
Afap1	1.0224	4.983	2.8996	0.0134	0.0374	-3.8347
Ncor2	0.4339	7.1172	2.8988	0.0134	0.0375	-3.9728
Gm3375	-0.4383	5.1391	-2.8987	0.0134	0.0375	-3.8502
Lrp6	0.2585	6.9332	2.8982	0.0134	0.0375	-3.9701
Cyp2j6	1.4775	1.4604	2.8973	0.0134	0.0375	-3.1702
Ndufs1	-0.2894	7.2213	-2.897	0.0134	0.0376	-3.978
Rab22a	0.4105	6.513	2.8968	0.0135	0.0376	-3.9632
Inpp5f	-0.521	5.0076	-2.8963	0.0135	0.0376	-3.8273
Bnip1	-0.4793	3.5635	-2.8962	0.0135	0.0376	-3.5896
Gm10851	3.2664	-3.2843	2.8955	0.0135	0.0376	-2.7402
Gm16062	-0.6042	1.7073	-2.8955	0.0135	0.0376	-3.2086
Kctd18	0.3664	3.6687	2.8946	0.0135	0.0377	-3.6354
Rorb	1.1835	2.0823	2.8945	0.0135	0.0377	-3.3068
Naip6	1.6192	-2.5515	2.8938	0.0135	0.0377	-2.7533
Arhgap4	0.9694	-0.6838	2.8938	0.0135	0.0377	-2.8944
Higd2a	0.357	5.4001	2.8935	0.0135	0.0377	-3.9019
Tmem40	-2.6647	-1.5275	-2.8933	0.0135	0.0378	-2.7597
Spaar	2.131	-2.8315	2.8928	0.0136	0.0378	-2.7457
Gm27232	2.321	-2.5204	2.8924	0.0136	0.0378	-2.7435
Fam136b-ps	-2.0484	-2.5599	-2.8921	0.0136	0.0378	-2.742
Mtpap	-0.4183	5.2632	-2.8917	0.0136	0.0378	-3.8726
Lpcat4	-0.7173	5.8566	-2.8915	0.0136	0.0378	-3.9183
Shox2	0.8821	3.0596	2.8893	0.0136	0.038	-3.5017
Hist1h2bp	1.9119	-2.5558	2.889	0.0136	0.038	-2.7467
Spink10	1.1902	-0.0694	2.8887	0.0137	0.038	-2.9631
Ndufaf5	-0.4459	3.2595	-2.8883	0.0137	0.0381	-3.5405
Camk1	0.4168	5.9199	2.8878	0.0137	0.0381	-3.9539
Gm11747	1.8333	-3.1866	2.8877	0.0137	0.0381	-2.7486
Anp32b-ps1	-1.3292	-1.1947	-2.8869	0.0137	0.0381	-2.8272
Ifi203-ps	-1.7367	1.1652	-2.8865	0.0137	0.0382	-3.0261
Tmem120b	0.5885	2.5503	2.8859	0.0137	0.0382	-3.4243
Pcdh1	1.4449	3.718	2.8851	0.0137	0.0382	-3.6193
Styk1	-1.7521	1.9074	-2.8846	0.0138	0.0383	-3.1917
Chd9	0.5261	5.5791	2.8839	0.0138	0.0383	-3.9225
Asf1a	-0.3662	4.9611	-2.8839	0.0138	0.0383	-3.8511

Ophn1	0.9152	1.8899	2.8839	0.0138	0.0383	-3.301
Gmpr	1.1938	3.2918	2.8835	0.0138	0.0383	-3.5505
Dcaf5	0.2681	5.7203	2.8834	0.0138	0.0383	-3.946
Ccdc130	0.496	3.1799	2.8833	0.0138	0.0383	-3.5658
Cpsf4l	1.5481	-2.731	2.8822	0.0138	0.0384	-2.7676
Akt1s1	0.3098	6.1226	2.8819	0.0138	0.0384	-3.9732
Ptk7	-0.3673	6.4193	-2.8818	0.0138	0.0384	-3.9851
Dynlrb1	0.3189	6.2417	2.8813	0.0138	0.0384	-3.9823
Lyrm4	-0.4872	3.7054	-2.8812	0.0138	0.0384	-3.6484
Pex5	0.3931	6.2741	2.8805	0.0139	0.0385	-3.9848
Tram1	-0.3389	7.3075	-2.8797	0.0139	0.0385	-4.0096
Mtif3	0.3852	3.7469	2.8796	0.0139	0.0385	-3.6787
Dbndd1	1.22	1.0929	2.8792	0.0139	0.0386	-3.1476
Ggh	1.0206	2.8116	2.8791	0.0139	0.0386	-3.4744
Parvg	-1.1612	-1.0307	-2.879	0.0139	0.0386	-2.871
Ccdc181	0.5198	3.9941	2.8782	0.0139	0.0386	-3.7366
Top3a	-0.3971	4.2116	-2.8776	0.0139	0.0386	-3.7432
Fryl	0.5129	6.1689	2.8775	0.0139	0.0386	-3.9794
AC115752.1	-0.9018	0.9891	-2.8753	0.014	0.0388	-3.1038
Mecp2	0.3913	6.232	2.8753	0.014	0.0388	-3.9897
Cep83os	0.7744	1.3959	2.8748	0.014	0.0388	-3.2342
Erbb2	0.9012	4.2506	2.8739	0.014	0.0389	-3.7548
Slc8b1	0.5324	4.4934	2.8738	0.014	0.0389	-3.8217
Radil	1.0027	0.3892	2.8737	0.014	0.0389	-3.0833
B9d1os	1.3384	-1.5825	2.8731	0.0141	0.0389	-2.8294
Sumo2	-0.3614	6.0836	-2.8719	0.0141	0.039	-3.9859
Usp22	0.4825	7.6081	2.8718	0.0141	0.039	-4.0322
Ppp4r1	-0.3	7.1092	-2.8713	0.0141	0.039	-4.0214
1110059G10Rik	0.5944	3.3216	2.8713	0.0141	0.039	-3.5988
Vnn3	2.3663	-3.859	2.8708	0.0141	0.0391	-2.7754
Mrpl35	-0.2822	5.4779	-2.8703	0.0141	0.0391	-3.9409
Slc5a3	-0.8316	7.3534	-2.8701	0.0141	0.0391	-4.0259
Meiob	-1.5025	-1.8343	-2.8689	0.0142	0.0392	-2.824
Pbld2	1.5145	-0.8996	2.8686	0.0142	0.0392	-2.902
Mettl22	-0.5538	2.4484	-2.8678	0.0142	0.0392	-3.4285
Sh3bgrl	0.541	7.4854	2.8672	0.0142	0.0393	-4.0366
Ccdc9b	1.2425	4.6754	2.8668	0.0142	0.0393	-3.8268
Stk39	-1.1535	4.7198	-2.8667	0.0142	0.0393	-3.7958
4933428G20Rik	1.6327	-2.06	2.8667	0.0142	0.0393	-2.802
Grid1	-2.2098	0.8434	-2.8666	0.0142	0.0393	-3.0026
Mrpl41	-0.5764	4.4204	-2.8661	0.0142	0.0393	-3.8028
Acap1	-2.4703	2.9398	-2.8654	0.0143	0.0394	-3.3094
Gm47270	0.9545	-0.467	2.865	0.0143	0.0394	-2.9708
Gm48789	-1.8688	-2.2301	-2.8649	0.0143	0.0394	-2.7913
Usp53	0.4526	4.302	2.8639	0.0143	0.0395	-3.7899
Cers6	0.4739	5.6192	2.8638	0.0143	0.0395	-3.9646
Ccdc17	1.0539	-0.4831	2.8632	0.0143	0.0395	-2.9532
Luzp1	0.3805	6.43	2.863	0.0143	0.0395	-4.0209
Gm24494	-1.0979	0.1383	-2.863	0.0143	0.0395	-2.9844
Zfp691	0.7127	2.0379	2.8628	0.0143	0.0395	-3.386
Oog1	2.0571	-2.1239	2.8627	0.0143	0.0395	-2.8025
Gabpa	-0.3541	6.5324	-2.8615	0.0144	0.0396	-4.0228

Gm7008	-0.6986	1.0459	-2.8608	0.0144	0.0396	-3.2046
Gm43890	-2.5992	-2.5809	-2.8604	0.0144	0.0397	-2.7891
Ifit3b	3.3457	-1.6699	2.8595	0.0144	0.0397	-2.807
Itfg2	-0.4171	4.1891	-2.8584	0.0144	0.0398	-3.7654
Ncor1	0.2756	7.9639	2.8584	0.0144	0.0398	-4.0611
Phc1	0.4142	5.3531	2.8583	0.0144	0.0398	-3.9585
Asb8	0.5176	5.3558	2.8574	0.0145	0.0399	-3.9561
Gm43759	1.8909	-2.1525	2.8572	0.0145	0.0399	-2.8025
Gm42798	-1.8415	-1.1341	-2.8571	0.0145	0.0399	-2.831
Smim27	0.9476	0.096	2.8561	0.0145	0.0399	-3.0345
Zbtb26	-0.4963	3.6732	-2.8556	0.0145	0.04	-3.6591
Ppargc1b	-0.7905	1.3076	-2.8555	0.0145	0.04	-3.2106
Mrps30	-0.3923	6.3298	-2.8554	0.0145	0.04	-4.0277
Wfikkn1	1.2686	0.3434	2.8542	0.0146	0.04	-3.0729
Mrgbp	-0.4844	4.407	-2.8539	0.0146	0.0401	-3.8187
Zbtb41	-0.3209	6.2471	-2.8526	0.0146	0.0402	-4.0253
Fars2	0.4237	4.142	2.8519	0.0146	0.0402	-3.7941
Cplane1	0.5005	5.9711	2.8515	0.0146	0.0402	-4.0174
Dok4	0.6915	3.0468	2.8514	0.0146	0.0402	-3.5976
Scn1b	1.1065	0.1677	2.8513	0.0146	0.0402	-3.0879
Ercc6l2	-0.329	5.3624	-2.85	0.0147	0.0403	-3.9567
Map2k1	-0.2856	6.8264	-2.8499	0.0147	0.0403	-4.0561
Zfp397	0.2458	5.5202	2.8494	0.0147	0.0403	-3.9874
Gm45447	1.7762	-2.1369	2.8494	0.0147	0.0403	-2.8305
Man1a	0.5842	6.368	2.8492	0.0147	0.0403	-4.0396
Upf3b	-0.3798	5.6926	-2.8492	0.0147	0.0403	-3.9958
Pde4d	2.7758	0.8615	2.8489	0.0147	0.0404	-3.037
Sema3b	1.6312	1.334	2.8488	0.0147	0.0404	-3.2623
Gm39460	-2.2565	-3.6009	-2.8486	0.0147	0.0404	-2.8084
Opa3	-0.3441	5.6271	-2.8484	0.0147	0.0404	-3.9902
Plxnb3	3.45	-2.9809	2.8484	0.0147	0.0404	-2.8092
Draxin	2.7735	-2.0964	2.8482	0.0147	0.0404	-2.8146
Gm5529	-1.188	-0.4665	-2.8481	0.0147	0.0404	-2.9737
Gpcpd1	0.537	4.1218	2.8481	0.0147	0.0404	-3.7937
Acot11	2.6031	-3.2897	2.8479	0.0147	0.0404	-2.8087
Selenok	0.3663	6.3402	2.8475	0.0147	0.0404	-4.0488
9130401M01Rik	-0.3001	5.0119	-2.8474	0.0147	0.0404	-3.9279
Slc44a1	1.0484	3.9165	2.8473	0.0147	0.0404	-3.7371
Hk2	-0.5313	6.9934	-2.8472	0.0147	0.0404	-4.0629
Papln	2.0165	-1.8918	2.847	0.0148	0.0404	-2.8477
Emb	1.4632	7.3861	2.8466	0.0148	0.0404	-4.0739
Gm14471	2.2738	-2.1393	2.8463	0.0148	0.0405	-2.8239
Rnf216	0.3133	6.2222	2.8458	0.0148	0.0405	-4.0427
Atat1	0.8176	3.4361	2.8458	0.0148	0.0405	-3.6571
AW011738	0.9167	-0.2581	2.8453	0.0148	0.0405	-3.0299
Sema4c	0.5182	4.9777	2.845	0.0148	0.0405	-3.9425
D630024D03Rik	2.2371	-1.6891	2.8441	0.0148	0.0406	-2.8608
Tmem159	0.7281	3.0908	2.8441	0.0148	0.0406	-3.6161
Tm9sf3	-0.3639	8.5116	-2.8439	0.0148	0.0406	-4.0936
Tmem94	0.3517	6.1888	2.8434	0.0149	0.0406	-4.0498
Gdpgp1	0.4878	2.7445	2.8433	0.0149	0.0406	-3.5295
BC037032	1.5399	-1.6317	2.8429	0.0149	0.0406	-2.8728

D130017N08Rik	0.9839	-0.3026	2.8423	0.0149	0.0407	-3.0264
Selenok-ps1	2.127	-2.9124	2.8422	0.0149	0.0407	-2.8169
Ppp2r5c	-0.2421	7.2898	-2.8421	0.0149	0.0407	-4.0794
Rsph3a	0.4907	3.4256	2.8419	0.0149	0.0407	-3.6943
Inafm2	-0.4703	4.9031	-2.8415	0.0149	0.0407	-3.9137
Eif3l	-0.3305	8.2799	-2.8415	0.0149	0.0407	-4.0959
Ankrd16	0.4645	3.7477	2.8413	0.0149	0.0407	-3.7424
Sla2	-1.6025	-1.3298	-2.8409	0.0149	0.0407	-2.854
Dnah10	1.6516	-2.7562	2.8405	0.0149	0.0408	-2.8197
Sptbn4	-2.155	-0.7311	-2.84	0.0149	0.0408	-2.8781
Gale	-0.8728	4.7281	-2.8394	0.015	0.0408	-3.8841
Slc48a1	-0.6805	7.5821	-2.8393	0.015	0.0408	-4.0886
Zfp606	0.71	3.3119	2.8392	0.015	0.0408	-3.655
Ubd	5.7539	-2.3449	2.839	0.015	0.0409	-2.8333
Cttn	0.2649	8.494	2.8387	0.015	0.0409	-4.1051
Cmbl	2.3578	4.0471	2.8387	0.015	0.0409	-3.7265
Nabp2	-0.3576	5.3212	-2.8383	0.015	0.0409	-3.9884
Mtm1	0.6338	3.0432	2.8381	0.015	0.0409	-3.6188
Emp3	-0.7049	5.8695	-2.8379	0.015	0.0409	-4.0212
Tagln2	-0.5989	8.808	-2.8377	0.015	0.0409	-4.1082
Zfp459	1.4651	-1.0119	2.8376	0.015	0.0409	-2.9578
Pde4b	1.989	3.4315	2.8374	0.015	0.0409	-3.5845
Atp2c1	0.2692	6.3246	2.8364	0.015	0.041	-4.0639
Btbd2	0.5836	4.9827	2.8359	0.0151	0.041	-3.9595
Zfp385a	0.9903	4.6076	2.8359	0.0151	0.041	-3.8989
Cisd2	-0.3113	5.8752	-2.8356	0.0151	0.041	-4.0351
Tmem161b	-0.4818	4.1599	-2.8355	0.0151	0.041	-3.7962
Cdh17	1.5615	-0.3867	2.8353	0.0151	0.041	-2.9806
Churc1	0.349	4.1401	2.8352	0.0151	0.041	-3.8366
Gm45890	2.0738	-1.6294	2.8349	0.0151	0.0411	-2.8699
Amd2	-0.88	1.1164	-2.8349	0.0151	0.0411	-3.2053
Gm19589	-2.339	-1.5391	-2.8341	0.0151	0.0411	-2.8431
Galnt1	-0.8036	8.126	-2.8336	0.0151	0.0411	-4.1062
Ube3c	-0.4277	7.4197	-2.8334	0.0151	0.0411	-4.0961
Ptpa	-0.3104	7.6968	-2.8331	0.0151	0.0412	-4.1028
Smarcb1	-0.3442	6.0643	-2.8321	0.0152	0.0412	-4.0581
Zfp90	0.8889	3.3695	2.832	0.0152	0.0412	-3.6769
Egfl8	-1.2444	-0.6384	-2.8317	0.0152	0.0413	-2.9745
Gm3650	0.8384	0.616	2.8314	0.0152	0.0413	-3.169
Tpm4	-0.4382	9.356	-2.831	0.0152	0.0413	-4.1251
Gm17825	-2.045	-2.8682	-2.8309	0.0152	0.0413	-2.8378
Tbx15	0.5221	4.0222	2.8308	0.0152	0.0413	-3.8052
Gm3839	-1.6873	-1.9429	-2.8307	0.0152	0.0413	-2.8772
Zfp688	0.4375	2.7121	2.83	0.0152	0.0413	-3.5634
Synj2bp	-0.2806	5.3718	-2.8294	0.0152	0.0414	-3.9983
Gtf2b	0.2902	5.7933	2.8289	0.0153	0.0414	-4.0508
Aif1	1.8285	-2.1005	2.8288	0.0153	0.0414	-2.8691
C230013L11Rik	0.8272	0.8132	2.8286	0.0153	0.0414	-3.2207
Apol7a	1.3609	0.9534	2.8286	0.0153	0.0414	-3.2238
Npas4	1.9556	-2.9213	2.8281	0.0153	0.0414	-2.8455
AC115631.1	0.6606	2.3018	2.8281	0.0153	0.0414	-3.4574
Rnf149	-0.3848	5.6643	-2.828	0.0153	0.0414	-4.0324

Cog7	0.3313	4.8035	2.8277	0.0153	0.0415	-3.9525
Gm42418	0.506	12.7267	2.8274	0.0153	0.0415	-4.1436
Tapt1	-0.4108	5.6633	-2.8274	0.0153	0.0415	-4.0294
C3ar1	-2.2203	-3.2785	-2.8272	0.0153	0.0415	-2.8395
Aqp8	-2.8347	-1.0029	-2.8267	0.0153	0.0415	-2.8719
4930589O11Rik	1.9275	-1.9826	2.8263	0.0153	0.0415	-2.8671
Heg1	1.8054	3.5726	2.8262	0.0153	0.0415	-3.6739
Gdap2	0.4172	4.7763	2.8258	0.0153	0.0416	-3.9447
Galm	0.7232	3.7469	2.8257	0.0153	0.0416	-3.7777
Prdx4	-0.5116	7.175	-2.8256	0.0153	0.0416	-4.1063
Ubl4a	-0.3506	5.6031	-2.8254	0.0154	0.0416	-4.0347
Wnk4	1.934	-0.2479	2.8254	0.0154	0.0416	-3.0065
Washc3	0.3816	4.9185	2.8253	0.0154	0.0416	-3.9728
Arap2	-1.0678	2.7941	-2.8252	0.0154	0.0416	-3.4709
1500011B03Rik	-0.3473	4.2561	-2.8247	0.0154	0.0416	-3.8546
Sumo1	-0.2879	6.9364	-2.8246	0.0154	0.0416	-4.1046
Zfp7	-0.5073	4.6952	-2.824	0.0154	0.0416	-3.9102
Dpp7	2.0824	1.3418	2.8225	0.0154	0.0418	-3.2667
Snapc4	-0.3887	4.9555	-2.8223	0.0154	0.0418	-3.9533
Slc7a2	2.6207	2.4403	2.8213	0.0155	0.0418	-3.3668
Gm15612	2.2448	-2.7665	2.8205	0.0155	0.0419	-2.8535
Rps15a-ps5	-1.0016	-0.2864	-2.8203	0.0155	0.0419	-3.0831
1110002L01Rik	-0.4986	4.458	-2.82	0.0155	0.0419	-3.8877
Tspan8	-1.5592	0.3639	-2.8199	0.0155	0.0419	-3.0503
Pip4k2b	-0.3131	6.6272	-2.8197	0.0155	0.0419	-4.1042
Epas1	1.1935	3.225	2.8197	0.0155	0.0419	-3.6634
Dhx9	-0.2694	8.5953	-2.8189	0.0155	0.042	-4.1405
Gm7589	-0.894	0.0457	-2.8187	0.0155	0.042	-3.1096
Gm10043	-2.1123	-0.8336	-2.8184	0.0156	0.042	-2.9142
Aasdhppt	-0.7231	5.7336	-2.8171	0.0156	0.0421	-4.042
Gulp1	1.6377	1.1761	2.8166	0.0156	0.0421	-3.2715
Atxn7l1	0.598	3.1214	2.8165	0.0156	0.0421	-3.6507
9430037O13Rik	-1.6974	-1.5798	-2.8162	0.0156	0.0421	-2.8746
Gjc2	3.4701	-2.859	2.8158	0.0156	0.0422	-2.8567
Rnaset2a	0.8534	0.9482	2.8151	0.0157	0.0422	-3.256
Arhgap35	0.4023	7.6553	2.815	0.0157	0.0422	-4.1362
Gm15559	1.3729	-1.5834	2.8149	0.0157	0.0422	-2.9334
Rassf3	-0.7605	4.7349	-2.8146	0.0157	0.0422	-3.9224
Dgat1	0.7144	4.1505	2.814	0.0157	0.0423	-3.8616
Mapk8	-0.354	5.2352	-2.8139	0.0157	0.0423	-4.0115
Nsun4	0.4735	3.2152	2.8139	0.0157	0.0423	-3.685
Lrrc75a	0.5002	2.1581	2.8137	0.0157	0.0423	-3.4908
Rnf19a	0.424	5.6881	2.8134	0.0157	0.0423	-4.0706
Zdhhc5	-0.2801	6.8006	-2.8134	0.0157	0.0423	-4.1207
Cib2	2.0568	2.3334	2.813	0.0157	0.0423	-3.4565
Pcdhb14	1.8006	-1.3786	2.8127	0.0157	0.0423	-2.9233
Casd1	0.3639	5.4329	2.8125	0.0157	0.0423	-4.0468
Cdh23	-1.8095	-2.8194	-2.8123	0.0157	0.0423	-2.8634
Zfp874a	0.6732	2.3818	2.8117	0.0158	0.0424	-3.546
Pced1b	1.0003	3.6471	2.8112	0.0158	0.0424	-3.7447
Nxph3	-2.6764	-1.3835	-2.8107	0.0158	0.0425	-2.8938
Stk11	-0.2608	6.4574	-2.81	0.0158	0.0425	-4.1178

Kmt2a	0.4671	6.9327	2.8099	0.0158	0.0425	-4.1308
1700029I15Rik	1.8727	-1.9189	2.8096	0.0158	0.0425	-2.886
Mmp24	1.2676	-1.3028	2.8093	0.0158	0.0425	-2.982
Arl8b	0.3281	7.1479	2.8088	0.0158	0.0426	-4.1387
Mllt3	-0.3127	6.2252	-2.8076	0.0159	0.0427	-4.11
Maml2	1.1201	2.3586	2.8071	0.0159	0.0427	-3.5117
Eif3f	0.3414	8.0341	2.807	0.0159	0.0427	-4.1572
Ptgdr2	2.8531	-2.834	2.8068	0.0159	0.0427	-2.8691
Derl3	2.0252	-1.8541	2.8064	0.0159	0.0427	-2.9165
Gm33023	2.0456	-2.8674	2.8063	0.0159	0.0427	-2.8714
Rtl10	-0.6897	0.9443	-2.8061	0.0159	0.0427	-3.2441
Fth1	-0.539	12.6695	-2.8058	0.0159	0.0427	-4.1827
Triqk	1.922	-1.9558	2.8055	0.0159	0.0428	-2.9032
Ctdspl	-0.5598	4.2057	-2.8055	0.0159	0.0428	-3.8507
Smu1	-0.2472	7.0148	-2.8055	0.0159	0.0428	-4.1406
Stx16	0.356	5.7803	2.8051	0.0159	0.0428	-4.0904
Adck5	0.4387	3.9512	2.8047	0.016	0.0428	-3.8421
Adamts7	0.8176	6.3796	2.8042	0.016	0.0428	-4.1237
Gm45865	1.8908	-3.0212	2.8036	0.016	0.0429	-2.8768
Psmb7	-0.4387	7.8016	-2.8033	0.016	0.0429	-4.1586
Rgs9bp	2.1307	-2.1069	2.8026	0.016	0.0429	-2.8967
Vps13b	0.4341	5.4631	2.8021	0.016	0.043	-4.0673
Slc37a3	0.2989	5.998	2.8021	0.016	0.043	-4.1126
Zfp507	0.2902	5.6148	2.8016	0.016	0.043	-4.0851
Gm44777	-1.2455	-0.0088	-2.8004	0.0161	0.0431	-3.087
Mfap1b	0.3675	3.4896	2.7998	0.0161	0.0431	-3.7593
Tatdn3	0.5609	2.7154	2.7982	0.0161	0.0433	-3.6246
Gm13597	1.6342	-1.9035	2.7979	0.0162	0.0433	-2.9235
Tmem212	-2.1402	-2.603	-2.7978	0.0162	0.0433	-2.882
Bend5	-1.8199	-2.5983	-2.7974	0.0162	0.0433	-2.8999
Akap2	0.7236	0.8328	2.7973	0.0162	0.0433	-3.2668
Gm5452	-1.2713	-0.9297	-2.7971	0.0162	0.0433	-3.0063
4921531C22Rik	0.9647	0.9687	2.7967	0.0162	0.0433	-3.271
Timm10b	-0.3501	3.8235	-2.7963	0.0162	0.0434	-3.8316
Gm26688	1.1343	-0.1469	2.7959	0.0162	0.0434	-3.111
Sh3bp4	0.6259	5.4193	2.7953	0.0162	0.0434	-4.072
Trp53bp2	0.3858	5.2401	2.7951	0.0162	0.0434	-4.062
Hexim2	0.6126	2.1109	2.795	0.0162	0.0434	-3.5269
Pnisr	-0.3395	5.4477	-2.7943	0.0163	0.0435	-4.0701
Gm6793	-0.8406	0.6221	-2.7936	0.0163	0.0435	-3.2222
Gm2027	-0.9776	0.4205	-2.7932	0.0163	0.0436	-3.1408
4632427E13Rik	0.7851	1.0487	2.7932	0.0163	0.0436	-3.3041
4930515G01Rik	0.9211	-0.4073	2.7918	0.0163	0.0437	-3.0997
Supt3	-0.448	3.9954	-2.7911	0.0164	0.0437	-3.8654
Gm45494	1.5049	-1.0575	2.7911	0.0164	0.0437	-2.9813
Ciita	2.3584	-1.4396	2.7909	0.0164	0.0437	-2.9679
Tgtp1	-1.9407	-2.3942	-2.7907	0.0164	0.0437	-2.9156
Hp	3.9108	3.1642	2.7905	0.0164	0.0437	-3.4507
Zbtb39	0.3538	3.718	2.7904	0.0164	0.0437	-3.8281
A630052C17Rik	1.2083	0.0427	2.7898	0.0164	0.0438	-3.1188
Gm47976	2.0707	-2.3764	2.7885	0.0164	0.0439	-2.9142
Adam9	0.464	7.7254	2.7885	0.0164	0.0439	-4.1858

Gm18890	1.5202	-2.1535	2.7882	0.0165	0.0439	-2.9301
Mgst3	0.7446	4.4837	2.7874	0.0165	0.0439	-3.9643
Snord89	-2.2174	-2.6739	-2.7871	0.0165	0.044	-2.8979
Rnf217	-0.3932	4.8521	-2.7868	0.0165	0.044	-4.0034
Inpp5a	0.3111	5.5135	2.7865	0.0165	0.044	-4.1059
Tent5c	2.5453	-0.5931	2.7862	0.0165	0.044	-2.981
Armc1	-0.4246	6.5226	-2.7852	0.0165	0.0441	-4.1624
S100a13	1.1335	4.2493	2.7843	0.0166	0.0441	-3.9233
1110008P14Rik	-0.6156	6.0622	-2.7843	0.0166	0.0441	-4.1397
Nadsyn1	-0.575	2.2819	-2.7843	0.0166	0.0441	-3.534
Intu	0.4082	3.9267	2.7837	0.0166	0.0442	-3.8851
Rnf32	1.5651	-0.0006	2.7835	0.0166	0.0442	-3.1335
Stt3a	-0.3084	8.383	-2.7834	0.0166	0.0442	-4.2028
Ankrd24	0.5264	2.1359	2.7826	0.0166	0.0443	-3.5384
Agap1	0.4884	6.7837	2.781	0.0167	0.0444	-4.1822
Afg1l	0.6727	2.6851	2.7807	0.0167	0.0444	-3.6665
Zfp39	0.5643	2.1287	2.7806	0.0167	0.0444	-3.5211
Grhl1	0.9892	0.0417	2.7805	0.0167	0.0444	-3.1446
1700029J07Rik	1.0676	-0.9573	2.7797	0.0167	0.0445	-3.0335
Cdc5l	-0.2675	6.6715	-2.7796	0.0167	0.0445	-4.1798
Cd53	-2.4962	-3.092	-2.7789	0.0167	0.0445	-2.9098
2810013P06Rik	0.7861	2.3682	2.7781	0.0168	0.0446	-3.5751
Serpini1	1.1955	2.4114	2.7771	0.0168	0.0446	-3.567
Lhx1	2.1871	-3.2241	2.777	0.0168	0.0446	-2.9125
Gm20544	-1.5019	-0.615	-2.7768	0.0168	0.0447	-3.0407
Il12rb1	-1.2612	2.7575	-2.7767	0.0168	0.0447	-3.5713
Zfp251	0.3406	4.89	2.7763	0.0168	0.0447	-4.0454
Tspan5	0.5662	6.0111	2.7763	0.0168	0.0447	-4.1558
Snrnp48	-0.4974	5.2531	-2.7762	0.0168	0.0447	-4.0782
Nkpd1	1.8564	-3.591	2.7757	0.0168	0.0447	-2.9145
Gm15411	-1.9449	-3.0548	-2.7756	0.0168	0.0447	-2.9148
Gm4864	-0.5984	1.7989	-2.7756	0.0168	0.0447	-3.4898
Ppa2	-0.2955	6.1941	-2.7755	0.0168	0.0447	-4.1693
Kcnh5	-2.8411	-1.5212	-2.7744	0.0169	0.0448	-2.9287
Mmp23	2.4816	-3.0808	2.774	0.0169	0.0448	-2.9184
6430531B16Rik	-1.8692	-1.8161	-2.7737	0.0169	0.0448	-2.9499
Mrpl16	-0.3746	5.0541	-2.7732	0.0169	0.0449	-4.0662
Gm47218	-1.3476	-1.1916	-2.7725	0.0169	0.0449	-3.0121
Il17ra	-0.4177	5.0594	-2.7715	0.017	0.045	-4.0608
C330013E15Rik	1.4952	-0.823	2.7714	0.017	0.045	-3.0419
Tpbg	0.4662	6.2017	2.7711	0.017	0.045	-4.1796
Gm6187	-1.771	-2.6726	-2.7699	0.017	0.0451	-2.9383
Rps8	-0.318	9.8745	-2.7699	0.017	0.0451	-4.2399
Aldh3a1	1.3469	5.8874	2.7695	0.017	0.0451	-4.1341
Trib1	-0.6504	6.1915	-2.7691	0.017	0.0452	-4.1684
Arhgap42	1.3301	6.6881	2.768	0.0171	0.0452	-4.1991
Dhrs9	-0.4845	4.3528	-2.7676	0.0171	0.0453	-3.9486
Gm17092	-0.4993	2.2408	-2.7675	0.0171	0.0453	-3.5438
Gpn1	-0.4709	5.3948	-2.7675	0.0171	0.0453	-4.113
Nup50	-0.3616	7.0699	-2.7672	0.0171	0.0453	-4.2101
Thra	0.6996	6.2425	2.7666	0.0171	0.0453	-4.1908
Ap4s1	-0.5109	3.319	-2.7666	0.0171	0.0453	-3.7627

Adam10	0.4319	8.5861	2.7663	0.0171	0.0453	-4.2373
C130060C02Ril	1.5264	-3.1444	2.7663	0.0171	0.0453	-2.9484
Arid3b	-1.0603	0.176	-2.7662	0.0171	0.0453	-3.1989
Phf20	0.389	6.9919	2.7655	0.0172	0.0454	-4.214
Eid1	-0.4312	6.5392	-2.7655	0.0172	0.0454	-4.1975
Gm12482	-1.0886	-1.114	-2.7655	0.0172	0.0454	-3.0829
Rhoa	-0.219	8.7967	-2.7649	0.0172	0.0454	-4.2417
Glyctk	0.9979	0.7743	2.7647	0.0172	0.0454	-3.2988
Cyp1b1	3.2429	2.5063	2.7646	0.0172	0.0454	-3.367
Vdac3	-0.314	7.188	-2.7645	0.0172	0.0454	-4.2189
Nxn	-0.5637	6.2694	-2.7637	0.0172	0.0455	-4.1878
Gm19221	-1.2307	-1.98	-2.7635	0.0172	0.0455	-2.9649
Gm28438	0.656	0.6898	2.7628	0.0172	0.0455	-3.3034
Zfp626	0.4212	3.4777	2.7626	0.0172	0.0456	-3.8243
Ldb3	1.6097	-2.7321	2.7624	0.0173	0.0456	-2.9712
AC124502.4	1.7606	-1.7052	2.7621	0.0173	0.0456	-3.0089
Zup1	0.4533	2.9093	2.7617	0.0173	0.0456	-3.7041
Trmt10b	0.4412	3.1423	2.7612	0.0173	0.0456	-3.7839
Tada1	-0.3054	5.2747	-2.7606	0.0173	0.0457	-4.1202
Gm14853	-1.3299	-1.9682	-2.7605	0.0173	0.0457	-3.0181
H2-Q4	1.2286	4.3174	2.7604	0.0173	0.0457	-3.9746
AU020206	-0.6693	5.0975	-2.7602	0.0173	0.0457	-4.0718
Ntn3	2.2592	-1.5036	2.7597	0.0173	0.0457	-3.0109
Wdr5b	0.7327	1.5785	2.7597	0.0173	0.0457	-3.4683
Gm28231	2.1377	-2.9936	2.7593	0.0174	0.0458	-2.947
Mfsd13b	2.4488	-3.472	2.7588	0.0174	0.0458	-2.9392
Col12a1	5.0747	1.9703	2.7584	0.0174	0.0458	-3.1739
Actr2	-0.3746	8.3498	-2.7583	0.0174	0.0458	-4.2476
Immp2l	0.6297	1.5115	2.7577	0.0174	0.0459	-3.4635
Gm7645	-1.8396	-2.8068	-2.7576	0.0174	0.0459	-2.9492
AC150274.1	-1.2902	-1.5298	-2.7572	0.0174	0.0459	-3.034
C1s2	2.8389	-0.8458	2.7569	0.0174	0.0459	-3.0281
Ripor3	-2.2148	-0.4075	-2.7561	0.0175	0.046	-3.0669
Gm29346	1.4442	-3.1766	2.7546	0.0175	0.0461	-2.9536
Dock3	-1.7081	-2.2192	-2.7546	0.0175	0.0461	-2.9621
Pex12	0.4122	3.6442	2.7543	0.0175	0.0461	-3.8772
Ifi47	1.7786	-0.0529	2.7539	0.0175	0.0461	-3.2518
Fcf1	-0.2759	5.651	-2.7538	0.0175	0.0461	-4.1731
Rwdd4a	-0.3759	5.6234	-2.7532	0.0176	0.0462	-4.1642
Pcdhga12	1.4449	-2.1525	2.7528	0.0176	0.0462	-3.0048
Polr2m	-0.2926	7.8011	-2.7527	0.0176	0.0462	-4.2505
Gm44899	1.9954	-2.173	2.7527	0.0176	0.0462	-2.9556
D130007C19Ril	1.2914	-1.2939	2.752	0.0176	0.0463	-3.0548
Metrn	-0.627	5.4666	-2.7519	0.0176	0.0463	-4.1434
Slc9a6	0.3389	5.272	2.7515	0.0176	0.0463	-4.137
Rnf6	-0.2467	6.6421	-2.7513	0.0176	0.0463	-4.2301
Gm26811	0.9329	-1.0961	2.7512	0.0176	0.0463	-3.1145
Tsc22d1	0.4387	7.7563	2.7503	0.0176	0.0464	-4.2558
Mcpt8	-2.8252	-3.0283	-2.7503	0.0176	0.0464	-2.9516
Izumo4	1.0594	0.5773	2.7501	0.0177	0.0464	-3.317
Armc5	0.3852	4.1341	2.7499	0.0177	0.0464	-3.9822
H2-T24	2.7349	-1.5767	2.7497	0.0177	0.0464	-2.9954

Ddx23	-0.4104	7.5359	-2.7494	0.0177	0.0464	-4.2517
Bag5	0.3149	4.8728	2.7493	0.0177	0.0464	-4.0985
AC090881.3	-0.9323	0.0115	-2.749	0.0177	0.0464	-3.2232
Selenow	0.3145	7.0952	2.7488	0.0177	0.0464	-4.2471
Gm8719	-1.0248	0.511	-2.7488	0.0177	0.0464	-3.2713
4931428F04Rik	0.4474	3.825	2.7482	0.0177	0.0465	-3.9345
Rap1gds1	-0.3127	7.4173	-2.748	0.0177	0.0465	-4.2528
Gm17110	1.5352	-1.7443	2.7478	0.0177	0.0465	-3.0033
Hook3	0.4605	6.357	2.7476	0.0177	0.0465	-4.2272
Atf7ip	0.5878	6.385	2.747	0.0178	0.0465	-4.2305
Tnks1bp1	0.4029	7.3787	2.747	0.0178	0.0465	-4.2554
Fzd1	1.1923	2.8335	2.747	0.0178	0.0465	-3.7215
Gm3788	0.7463	0.8591	2.7468	0.0178	0.0465	-3.3869
Tcf4	0.5366	7.0214	2.7466	0.0178	0.0465	-4.2492
Dexi	0.3976	3.8092	2.7466	0.0178	0.0465	-3.9139
Cisd3	-0.3965	4.2256	-2.7465	0.0178	0.0465	-3.9909
Dop1b	1.1594	3.9548	2.7464	0.0178	0.0465	-3.9307
1700088E04Rik	0.6622	2.0763	2.7464	0.0178	0.0465	-3.6098
Otogl	-1.4803	-1.0088	-2.7459	0.0178	0.0466	-3.0484
Gm6540	-1.206	-1.5807	-2.7456	0.0178	0.0466	-3.0368
Gstp-ps	-0.5579	1.5359	-2.7455	0.0178	0.0466	-3.486
Gm37694	1.9138	-3.4036	2.7453	0.0178	0.0466	-2.9613
Socs6	-0.3639	5.726	-2.7453	0.0178	0.0466	-4.1823
Kif1bp	0.2982	5.3794	2.7448	0.0178	0.0466	-4.171
Soat1	-0.3818	7.071	-2.7445	0.0178	0.0467	-4.2513
Gm49417	2.5698	-2.1045	2.7444	0.0178	0.0467	-2.9732
Dmpk	1.0139	5.2848	2.7441	0.0179	0.0467	-4.1534
Arf4	-0.301	8.2339	-2.7439	0.0179	0.0467	-4.2726
Uck1	-0.2667	5.2819	-2.7437	0.0179	0.0467	-4.1467
Bag1	-0.2452	7.1013	-2.7434	0.0179	0.0467	-4.2557
Mdp1	0.4375	3.8322	2.7422	0.0179	0.0468	-3.9536
Tmem198b	-0.5601	4.1366	-2.7421	0.0179	0.0468	-3.9702
Prdm11	2.908	0.7984	2.7419	0.0179	0.0468	-3.1967
Mrpl58	-0.4415	4.6964	-2.7414	0.0179	0.0469	-4.073
Tlr5	2.0919	1.0655	2.7414	0.0179	0.0469	-3.3492
Plcd1	1.5486	3.3785	2.7409	0.018	0.0469	-3.8076
9530056K15Rik	-1.5118	-1.8117	-2.7409	0.018	0.0469	-3.0051
Ttc32	-0.649	3.1497	-2.7402	0.018	0.0469	-3.7759
Gm5560	-1.1276	-0.4641	-2.7402	0.018	0.0469	-3.1727
Ppp1cc	-0.2967	6.9813	-2.7401	0.018	0.0469	-4.2589
Gm8822	-0.7666	0.4699	-2.7398	0.018	0.0469	-3.3001
Ints2	-0.464	5.8301	-2.7396	0.018	0.047	-4.2008
Zfp667	0.5391	2.13	2.7396	0.018	0.047	-3.6082
Gm50037	-1.5275	-1.9147	-2.7395	0.018	0.047	-3.0055
Oscp1	0.4019	2.5265	2.7383	0.018	0.047	-3.7043
Dnaja2	-0.3404	7.8887	-2.7383	0.018	0.047	-4.2774
Tekt2	2.3598	-2.5106	2.7377	0.0181	0.0471	-2.9882
Cpne5	-2.222	-2.4174	-2.7374	0.0181	0.0471	-2.9732
Dusp28	0.5114	2.003	2.7373	0.0181	0.0471	-3.6003
Psmb2	-0.3366	6.968	-2.737	0.0181	0.0471	-4.2645
G6pdx	-0.453	7.7884	-2.737	0.0181	0.0471	-4.2791
Ifitm6	-2.5761	-2.2428	-2.7365	0.0181	0.0472	-2.9816

Rexo2	-0.341	6.5599	-2.7361	0.0181	0.0472	-4.2546
6330562C20Rik	-0.8591	0.9126	-2.7357	0.0181	0.0472	-3.3512
Gm32742	-2.4008	-3.6947	-2.7355	0.0181	0.0472	-2.9733
Fbln7	3.2799	0.4149	2.7352	0.0181	0.0472	-3.1524
Rab7	0.2197	8.2025	2.735	0.0182	0.0473	-4.2897
Setd3	-0.2606	6.4779	-2.7343	0.0182	0.0473	-4.256
Gm37249	-1.4357	-1.951	-2.7343	0.0182	0.0473	-3.0207
Supt7l	0.3395	4.7472	2.7337	0.0182	0.0473	-4.101
Mdfi	-1.3324	0.5838	-2.7332	0.0182	0.0474	-3.2683
4930520O04Rik	-1.2165	0.0519	-2.733	0.0182	0.0474	-3.1816
Bloc1s5	0.3976	4.1017	2.7329	0.0182	0.0474	-4.002
Klrg2	-0.897	4.3192	-2.7326	0.0182	0.0474	-4.0045
Gm20400	-2.4955	-3.4176	-2.7323	0.0182	0.0474	-2.9779
Cep126	-1.7913	-0.7971	-2.7319	0.0183	0.0475	-3.0756
Amd-ps3	-1.036	-0.6125	-2.7296	0.0183	0.0477	-3.1358
Lamtor2	0.3081	5.5878	2.7287	0.0184	0.0477	-4.2186
Gcfc2	-0.3869	3.7224	-2.7284	0.0184	0.0477	-3.9097
Med9os	1.2946	-1.8957	2.7276	0.0184	0.0478	-3.0413
Mapk7	0.3177	5.2453	2.7268	0.0184	0.0479	-4.1808
Ifit1	2.3004	2.2273	2.7268	0.0184	0.0479	-3.5354
Aga	0.7793	3.4143	2.7266	0.0184	0.0479	-3.8886
Gm16536	0.5664	2.6964	2.7263	0.0184	0.0479	-3.7659
Unc13b	3.0966	-1.7442	2.7258	0.0185	0.0479	-3.0224
Eef2kmt	-0.6783	4.4404	-2.7257	0.0185	0.0479	-4.0463
Mboat2	0.4882	4.5997	2.7244	0.0185	0.048	-4.1006
AU021092	2.2226	-2.3773	2.724	0.0185	0.0481	-3.0168
Zmynd8	0.4524	6.3482	2.7231	0.0186	0.0481	-4.2742
Ppp4r1l-ps	0.6844	2.5852	2.7222	0.0186	0.0482	-3.7243
Gm16183	1.6744	-3.1697	2.7221	0.0186	0.0482	-3.0083
Gm44873	1.4615	-1.6824	2.7219	0.0186	0.0482	-3.0688
Lca5	-0.5567	3.5644	-2.7219	0.0186	0.0482	-3.8987
Brox	0.286	5.5784	2.7219	0.0186	0.0482	-4.2285
Plekhh3	-0.5016	4.7589	-2.7216	0.0186	0.0482	-4.1124
Ankrd45	1.9539	-3.1556	2.7213	0.0186	0.0482	-2.9953
Gm45667	0.6038	1.0181	2.7211	0.0186	0.0483	-3.4364
Ggt6	2.745	-2.684	2.7206	0.0186	0.0483	-3.0065
Sec24a	-0.3237	5.6245	-2.72	0.0187	0.0483	-4.221
Gm4609	-1.2476	-1.9226	-2.7194	0.0187	0.0484	-3.044
Ptrh1	0.5956	3.6828	2.719	0.0187	0.0484	-3.9471
C78859	-1.7998	-2.8013	-2.7189	0.0187	0.0484	-3.0006
Mmaa	0.4969	3.4303	2.7177	0.0187	0.0485	-3.9099
Aldh16a1	-0.3157	4.843	-2.7176	0.0187	0.0485	-4.1365
Rnmt	-0.3426	6.475	-2.7175	0.0188	0.0485	-4.2843
Rnf151	-1.4407	-1.1853	-2.717	0.0188	0.0486	-3.0748
Gpr137c	2.7793	-0.3525	2.717	0.0188	0.0486	-3.1298
Ppbp	-1.5238	-0.486	-2.7158	0.0188	0.0487	-3.1502
Selenbp2	2.5321	-3.8348	2.7156	0.0188	0.0487	-3.0023
Ube2d3	-0.3773	8.6461	-2.7145	0.0189	0.0488	-4.3303
Togaram2	0.7967	1.6751	2.7144	0.0189	0.0488	-3.5654
Btg1	0.3298	7.6024	2.7143	0.0189	0.0488	-4.3182
Gigyf1	0.3777	5.4144	2.7141	0.0189	0.0488	-4.2245
Pcif1	-0.3344	5.572	-2.714	0.0189	0.0488	-4.2379

Efhc1	1.074	0.5333	2.7136	0.0189	0.0488	-3.3894
Ppp1r15b	-0.373	7.1433	-2.7134	0.0189	0.0488	-4.3092
Pcx	0.7882	5.56	2.7133	0.0189	0.0488	-4.2396
Chchd10	1.8331	-0.1763	2.7132	0.0189	0.0488	-3.225
Gm17655	1.9361	-2.2016	2.7127	0.0189	0.0489	-3.0214
A530072M11Ril	2.5675	-2.2509	2.7125	0.0189	0.0489	-3.0305
Fam222a	3.3201	-1.561	2.7118	0.0189	0.0489	-3.0501
Atp13a4	-1.1959	2.7354	-2.7116	0.019	0.0489	-3.6576
Map11	-0.4818	3.7191	-2.7114	0.019	0.0489	-3.9507
Pdk1	-0.4969	6.4806	-2.711	0.019	0.049	-4.2941
Suclg2	-0.5206	6.2005	-2.7109	0.019	0.049	-4.2802
Nol6	-0.4123	6.3946	-2.7106	0.019	0.049	-4.2906
Lztr1	0.2989	6.4737	2.7101	0.019	0.049	-4.3015
Eea1	-0.3826	5.743	-2.7098	0.019	0.049	-4.2522
Wwp1	-0.303	5.2289	-2.7097	0.019	0.049	-4.1976
Commd6	0.4597	4.3451	2.7091	0.019	0.0491	-4.1026
Prss22	-3.2237	-0.4106	-2.709	0.019	0.0491	-3.0683
Tax1bp1	0.2927	7.746	2.7088	0.0191	0.0491	-4.3304
Txndc5	-0.3789	8.5406	-2.7076	0.0191	0.0492	-4.3422
Rcor1	-0.2785	6.7238	-2.7069	0.0191	0.0493	-4.3117
Kcnd1	0.569	3.2319	2.7067	0.0191	0.0493	-3.8865
Anxa7	-0.2988	6.7113	-2.7067	0.0191	0.0493	-4.3145
Gm6352	1.9606	-2.6919	2.7065	0.0191	0.0493	-3.0325
Cbr1	0.4288	4.3877	2.7064	0.0191	0.0493	-4.1008
Gm17108	-0.9221	1.1934	-2.7062	0.0191	0.0493	-3.4516
Gripap1	0.2453	5.6176	2.7061	0.0191	0.0493	-4.2597
AC129773.1	-0.9961	-0.5981	-2.706	0.0192	0.0493	-3.2246
Mterf3	-0.3097	4.7445	-2.706	0.0192	0.0493	-4.1444
C87436	0.3658	4.0418	2.706	0.0192	0.0493	-4.0436
Ankrd61	1.6259	-1.1724	2.7059	0.0192	0.0493	-3.0822
Lrp2bp	1.7178	-2.6717	2.7059	0.0192	0.0493	-3.0362
Zfp644	0.3131	5.6684	2.7058	0.0192	0.0493	-4.2611
Ocln	-2.1168	-1.6547	-2.7057	0.0192	0.0493	-3.0457
D130020L05Rik	0.7511	0.5438	2.7056	0.0192	0.0493	-3.4221
Arv1	-0.6692	2.8055	-2.7054	0.0192	0.0493	-3.7747
Tpd52l1	1.3332	-0.9187	2.7048	0.0192	0.0493	-3.175
Gm9432	-1.747	-2.3401	-2.7046	0.0192	0.0494	-3.0391
Ggnbp1	0.6383	0.9415	2.7042	0.0192	0.0494	-3.4607
9430037G07Rik	-1.8008	-1.9153	-2.7041	0.0192	0.0494	-3.069
Dmp1	-2.5282	-2.9528	-2.7041	0.0192	0.0494	-3.0191
Yod1	-0.5218	3.5751	-2.7039	0.0192	0.0494	-3.919
Gad1	2.2547	-2.1575	2.7037	0.0192	0.0494	-3.0367
Spty2d1	-0.4327	5.3046	-2.7035	0.0192	0.0494	-4.2145
Gm6421	0.9754	0.1609	2.7034	0.0192	0.0494	-3.317
Eaf2	0.6861	1.3044	2.7031	0.0193	0.0494	-3.5566
Rbm6	0.2439	6.4844	2.7028	0.0193	0.0495	-4.3136
Fastkd1	-0.4984	3.4834	-2.7027	0.0193	0.0495	-3.9194
Alg2	0.3354	5.1856	2.7024	0.0193	0.0495	-4.2215
Gm10635	2.41	-2.656	2.7024	0.0193	0.0495	-3.0254
Tsen34	-0.2934	6.0497	-2.702	0.0193	0.0495	-4.2939
H3f3a-ps2	1.3768	-1.5143	2.7007	0.0193	0.0496	-3.1036
Rbmx	-0.3879	5.0161	-2.7006	0.0193	0.0496	-4.1958

Rrp7a	-0.4749	6.4942	-2.7004	0.0194	0.0496	-4.3164
Rflna	2.5247	-3.568	2.7003	0.0194	0.0496	-3.0246
Prl2c5	-2.0627	-1.8963	-2.7002	0.0194	0.0496	-3.0574
AC132253.6	-0.7166	0.6422	-2.6997	0.0194	0.0497	-3.3406
Lpcat1	-0.5562	6.3437	-2.6995	0.0194	0.0497	-4.3066
AC121821.2	-0.6944	-0.1332	-2.6978	0.0194	0.0498	-3.2933
Gm6257	-1.1381	-2.3298	-2.6968	0.0195	0.0499	-3.0863
Pard6g	1.4552	1.5561	2.6962	0.0195	0.05	-3.5545
Dennd6a	0.422	5.6905	2.6956	0.0195	0.05	-4.2794
Dnajc17	-0.5205	2.8429	-2.6955	0.0195	0.05	-3.8171
Amfr	0.2792	7.4259	2.6955	0.0195	0.05	-4.3489
Gstm2	2.1108	2.5803	2.6952	0.0195	0.05	-3.7091
Gm49257	2.8928	-3.5497	2.6948	0.0196	0.0501	-3.0327
Klhl33	3.2837	-2.2069	2.6941	0.0196	0.0501	-3.0411
Rasip1	-0.9048	0.9196	-2.6938	0.0196	0.0501	-3.4231
Dhtkd1	0.9299	2.246	2.6935	0.0196	0.0501	-3.7268
Kansl3	-0.2708	6.1086	-2.6934	0.0196	0.0502	-4.3114
Gm48632	-1.8399	-3.5163	-2.693	0.0196	0.0502	-3.0355
Med8	-0.3203	5.0532	-2.6928	0.0196	0.0502	-4.2164
Gm20430	1.6764	-3.0548	2.6922	0.0196	0.0502	-3.0366
Iars2	-0.2479	7.0574	-2.6917	0.0197	0.0503	-4.3481
Prpsap1	-0.2816	6.5969	-2.6911	0.0197	0.0503	-4.3381
Zfp768	0.4118	4.2515	2.691	0.0197	0.0503	-4.1096
Gm45631	1.7864	-3.0688	2.691	0.0197	0.0503	-3.0564
Mylpf	-0.8057	0.4951	-2.6901	0.0197	0.0504	-3.3851
Nalcn	2.3222	-2.9408	2.6897	0.0197	0.0504	-3.0528
Pex6	0.3654	5.4238	2.6893	0.0198	0.0505	-4.274
Gm45399	-1.857	-3.085	-2.6888	0.0198	0.0505	-3.0416
Fyttd1	-0.2946	6.9866	-2.6885	0.0198	0.0505	-4.3513
Tigd5	0.5419	1.5142	2.6873	0.0198	0.0506	-3.5757
Shc2	2.6896	-1.7596	2.6872	0.0198	0.0506	-3.0749
AU021063	3.0564	-2.805	2.6871	0.0198	0.0506	-3.0438
Alkbh6	0.4807	3.3541	2.6869	0.0198	0.0506	-3.9291
2210016F16Rik	0.4454	3.2838	2.6865	0.0199	0.0507	-3.9395
C920021L13Rik	1.9953	-2.1276	2.6863	0.0199	0.0507	-3.0563
Shld1	1.2927	0.0304	2.6859	0.0199	0.0507	-3.3079
Alg11	0.317	5.1359	2.6856	0.0199	0.0507	-4.2448
Clybl	0.6881	2.9244	2.6848	0.0199	0.0508	-3.8902
Ccdc39	1.8619	-0.9663	2.6843	0.0199	0.0508	-3.1843
Myrfl	1.6651	-3.346	2.684	0.0199	0.0509	-3.0616
Xist	-0.5139	6.8494	-2.6836	0.02	0.0509	-4.353
Gm13669	-1.9137	-2.5627	-2.6834	0.02	0.0509	-3.0725
Atp5d	-0.2933	7.1182	-2.6833	0.02	0.0509	-4.3652
Nudt1	-0.3444	3.6475	-2.6829	0.02	0.0509	-3.9919
Adgra3	0.3277	6.3717	2.6827	0.02	0.0509	-4.3472
Slc26a11	0.7424	2.4288	2.6826	0.02	0.0509	-3.7755
A330035P11Rik	1.1227	-0.0584	2.681	0.0201	0.0511	-3.3224
9930012K11Rik	0.6413	1.9259	2.6807	0.0201	0.0511	-3.6535
Pomt1	-0.4086	4.4161	-2.6802	0.0201	0.0511	-4.1298
Pde5a	1.5254	1.9691	2.6798	0.0201	0.0512	-3.6568
Mir1905	2.057	-2.8269	2.6787	0.0201	0.0513	-3.0609
L3mbtl3	0.375	4.0684	2.6784	0.0202	0.0513	-4.1036

C2cd2	0.6007	3.6111	2.6775	0.0202	0.0514	-3.9925
Gm11185	-1.1408	-1.3092	-2.6774	0.0202	0.0514	-3.176
Slc6a2	3.9209	-0.2349	2.6774	0.0202	0.0514	-3.1172
Map3k13	1.1172	1.1957	2.6773	0.0202	0.0514	-3.5455
2610020C07Rik	-1.0799	0.7586	-2.6771	0.0202	0.0514	-3.4053
Rngtt	-0.3909	4.9356	-2.677	0.0202	0.0514	-4.2087
Gm14327	1.9654	-3.0345	2.6767	0.0202	0.0514	-3.0728
E130307A14Rik	0.8842	1.3398	2.6766	0.0202	0.0514	-3.6049
Pip5k1a	0.2552	7.0082	2.6766	0.0202	0.0514	-4.3759
Prss16	-1.2966	-0.5535	-2.6764	0.0202	0.0514	-3.2458
Vps39	0.3287	4.8737	2.6748	0.0203	0.0515	-4.2376
Gm17334	2.8352	-1.1339	2.6746	0.0203	0.0516	-3.1185
Kirrel3	2.1756	-1.5553	2.6744	0.0203	0.0516	-3.1052
Acsf3	0.3145	3.7658	2.6739	0.0203	0.0516	-4.0432
Cdc26	-0.2801	4.6824	-2.6735	0.0203	0.0516	-4.1878
Lgals3	0.7333	7.9039	2.6732	0.0203	0.0517	-4.3993
Camk2a	4.1576	-1.4741	2.6732	0.0203	0.0517	-3.0715
Tle2	0.9213	2.8461	2.6726	0.0204	0.0517	-3.8412
Hps5	0.3742	4.8823	2.6725	0.0204	0.0517	-4.2365
Gm9484	2.0124	-3.0003	2.6725	0.0204	0.0517	-3.0792
Mex3a	0.8168	6.1953	2.6712	0.0204	0.0518	-4.3595
Fam45a	-0.2898	4.5195	-2.6707	0.0204	0.0519	-4.1704
Isg15	1.6111	2.1168	2.6706	0.0204	0.0519	-3.7023
Zfp236	0.3457	5.8359	2.6702	0.0205	0.0519	-4.3393
Aldh1l1	3.5874	-1.4209	2.6701	0.0205	0.0519	-3.0877
Syt8	1.5678	0.4459	2.6697	0.0205	0.0519	-3.4387
Tmem158	-1.0445	2.7259	-2.6693	0.0205	0.0519	-3.7772
Aacs	-0.3369	6.8889	-2.669	0.0205	0.052	-4.3848
Impad1	0.3741	7.3062	2.6686	0.0205	0.052	-4.395
Gm11688	-1.0759	-0.8295	-2.668	0.0205	0.0521	-3.241
Atg16l2	0.4945	2.6779	2.6679	0.0205	0.0521	-3.8676
Gm43621	-2.6206	-2.4864	-2.6677	0.0206	0.0521	-3.0745
Trim13	0.5623	1.8625	2.6662	0.0206	0.0522	-3.6858
Fbxl18	0.3585	4.8968	2.6654	0.0206	0.0523	-4.2432
Prkab2	0.4425	3.7155	2.6651	0.0207	0.0523	-4.0483
Uso1	-0.2947	7.3392	-2.665	0.0207	0.0523	-4.4015
Adprm	0.35	3.2654	2.6643	0.0207	0.0523	-3.9828
Chst11	0.4788	5.4206	2.6643	0.0207	0.0523	-4.3116
Gm37174	1.7644	-2.0599	2.6639	0.0207	0.0524	-3.128
Usp30	0.3852	4.9349	2.6638	0.0207	0.0524	-4.2639
Ifi211	-1.1024	2.4436	-2.6637	0.0207	0.0524	-3.7154
Ptprq	-1.5405	-0.7427	-2.6635	0.0207	0.0524	-3.1765
Akirin2	-0.3036	5.9461	-2.6633	0.0207	0.0524	-4.3569
Gm10463	1.6929	-1.4503	2.6622	0.0208	0.0525	-3.1547
Btbd19	-0.7796	2.778	-2.6621	0.0208	0.0525	-3.8185
Rnf157	0.7899	4.6882	2.6619	0.0208	0.0525	-4.2254
Mrm1	-0.3885	3.0318	-2.6615	0.0208	0.0525	-3.9238
Zfp953	0.7863	2.0323	2.6611	0.0208	0.0526	-3.7135
Zfp143	-0.2961	4.85	-2.6605	0.0208	0.0526	-4.2407
Gm12976	-1.2507	-2.0617	-2.6603	0.0208	0.0526	-3.1668
Gm11814	-2.0081	-1.2907	-2.6603	0.0208	0.0526	-3.1631
Fam210a	-0.3786	6.7705	-2.6601	0.0208	0.0526	-4.3966

Pi4ka	0.4341	6.2934	2.6596	0.0209	0.0527	-4.3844
Bpgm	0.3882	4.2045	2.6586	0.0209	0.0528	-4.163
Gpr156	1.4712	-2.7724	2.658	0.0209	0.0528	-3.1005
Fgr	-2.3225	-2.712	-2.6579	0.0209	0.0528	-3.0869
Taok3	0.5627	4.9273	2.6577	0.0209	0.0528	-4.2774
Serpinb1b	1.7936	-1.987	2.6574	0.0209	0.0529	-3.1326
Plvap	-0.988	1.4424	-2.6568	0.021	0.0529	-3.5663
Gm8483	-1.4664	-0.2534	-2.6563	0.021	0.053	-3.3034
Gm10516	1.0138	-0.3714	2.6562	0.021	0.053	-3.3234
Ptges2	-0.3473	5.1135	-2.6561	0.021	0.053	-4.2793
Tpp2	-0.3359	7.4835	-2.6559	0.021	0.053	-4.4196
Atg3	0.3391	5.4494	2.6556	0.021	0.053	-4.3344
Fkbp14	0.7259	4.5557	2.6555	0.021	0.053	-4.208
Ephb3	1.053	3.1374	2.6549	0.021	0.053	-3.9471
S100a10	-0.3272	9.4502	-2.6538	0.0211	0.0531	-4.4475
Mapk1	-0.311	7.9643	-2.6524	0.0211	0.0533	-4.4339
Exosc4	-0.4576	5.1467	-2.6524	0.0211	0.0533	-4.2971
08-sept	0.4631	6.7052	2.6519	0.0212	0.0533	-4.4133
Rnf13	0.5247	6.598	2.6516	0.0212	0.0533	-4.4116
Gm17690	0.5919	1.5388	2.6516	0.0212	0.0533	-3.6708
Trappc11	0.3885	5.8782	2.6516	0.0212	0.0533	-4.3745
Mettl26	-0.4213	3.9683	-2.6515	0.0212	0.0533	-4.1018
Tmx1	-0.3903	6.8628	-2.6514	0.0212	0.0533	-4.4134
Blvra	-0.3923	4.6422	-2.6506	0.0212	0.0534	-4.2289
2310061I04Rik	-0.3403	4.3476	-2.6499	0.0212	0.0534	-4.1826
Pcdhb12	2.3067	-2.9588	2.6495	0.0213	0.0535	-3.1069
Lcor	-0.4964	5.0995	-2.6482	0.0213	0.0536	-4.2907
Abhd10	-0.3948	3.5726	-2.6474	0.0213	0.0537	-4.0392
Dus4l	-0.4933	2.909	-2.6465	0.0214	0.0537	-3.9037
Hibch	0.3896	4.5872	2.6456	0.0214	0.0538	-4.2405
Stk38l	0.5166	4.9292	2.6456	0.0214	0.0538	-4.2807
Psmb6	-0.2656	7.3748	-2.6451	0.0214	0.0539	-4.4391
Gm13470	1.3149	0.2419	2.6449	0.0214	0.0539	-3.378
Col14a1	1.3115	0.641	2.6447	0.0214	0.0539	-3.5044
Cdk16	-0.3057	7.4023	-2.6446	0.0214	0.0539	-4.4391
Creb3l4	1.2781	0.0349	2.644	0.0215	0.0539	-3.4311
Fbxl19	0.2867	5.4948	2.6433	0.0215	0.054	-4.36
Gm15975	-0.5483	2.1097	-2.6431	0.0215	0.054	-3.7393
Rnf14	0.236	6.5601	2.6425	0.0215	0.0541	-4.4265
Fbxl22	1.2548	0.9705	2.6422	0.0215	0.0541	-3.5562
Nr1h4	2.4468	-1.2458	2.6417	0.0216	0.0541	-3.1902
Coq4	-0.5133	3.703	-2.6416	0.0216	0.0541	-4.0628
Fgfr2	3.7797	-2.5347	2.6413	0.0216	0.0542	-3.1095
Acaa1b	0.6807	0.5887	2.6406	0.0216	0.0542	-3.5369
Rnasek	0.9702	1.2185	2.6403	0.0216	0.0542	-3.6005
Gm6366	-1.5672	-2.8177	-2.64	0.0216	0.0543	-3.1432
4833427G06Rik	-2.0813	-2.9217	-2.6394	0.0217	0.0543	-3.1163
Gm30794	1.4056	-1.2416	2.6391	0.0217	0.0543	-3.2671
Dda1	-0.233	6.2069	-2.6388	0.0217	0.0544	-4.417
Itgae	2.4461	-2.8226	2.6386	0.0217	0.0544	-3.123
Tbc1d22b	0.2953	4.5885	2.6382	0.0217	0.0544	-4.2569
Tceal1	2.6886	-0.9492	2.6381	0.0217	0.0544	-3.1823

Gm37303	1.8772	-2.0826	2.6378	0.0217	0.0544	-3.1379
Hk1os	1.6566	-1.0393	2.6374	0.0217	0.0545	-3.2738
Sash1	0.5073	5.2683	2.6373	0.0217	0.0545	-4.3439
Apool	0.4865	4.4575	2.6371	0.0218	0.0545	-4.2427
Gm44164	-1.7577	-1.9535	-2.6369	0.0218	0.0545	-3.1475
Iscu	0.3019	4.7257	2.6367	0.0218	0.0545	-4.2805
Ddx41	-0.4688	6.4885	-2.6357	0.0218	0.0546	-4.4313
Gm42984	-1.5382	-1.0608	-2.6354	0.0218	0.0546	-3.2597
Pitpnm2	0.5998	5.3443	2.6345	0.0219	0.0547	-4.3533
Setmar	1.4046	0.3221	2.6344	0.0219	0.0547	-3.4407
Rmdn1	0.653	1.6543	2.6337	0.0219	0.0548	-3.7352
Gm44292	2.2593	-3.0699	2.6333	0.0219	0.0548	-3.1226
Gpsm2	-0.5595	7.0691	-2.6331	0.0219	0.0548	-4.4517
1700016C15Rik	1.9584	-1.1731	2.633	0.0219	0.0548	-3.257
St7l	0.3002	4.439	2.6329	0.0219	0.0548	-4.2379
B230208H11Rik	-0.8676	0.3944	-2.6329	0.0219	0.0548	-3.4816
Gm14270	-0.9757	0.2417	-2.6327	0.0219	0.0548	-3.4495
Atg9b	-0.9791	2.9299	-2.6321	0.022	0.0549	-3.89
Bmp4	2.4721	-0.161	2.6321	0.022	0.0549	-3.2784
C1s1	3.4635	1.8379	2.6319	0.022	0.0549	-3.5746
Smim7	-0.3069	5.8972	-2.6313	0.022	0.0549	-4.4115
Gys1	-0.4329	6.4537	-2.631	0.022	0.0549	-4.4382
Cnot10	-0.3268	5.949	-2.6308	0.022	0.055	-4.4127
2500002B13Rik	-0.5874	1.9299	-2.6303	0.022	0.055	-3.7472
Zfp341	-0.4706	2.2648	-2.6303	0.022	0.055	-3.8223
Ndufaf3	0.5031	3.0441	2.6299	0.022	0.055	-4.004
Zfp119b	0.7215	1.52	2.6297	0.022	0.055	-3.689
Bdkrb2	-2.6392	1.0344	-2.6296	0.0221	0.055	-3.4152
Gm7334	-0.9584	0.1546	-2.629	0.0221	0.0551	-3.4207
Stk10	0.7315	5.2241	2.6289	0.0221	0.0551	-4.356
Catsperd	1.5181	-2.0706	2.6282	0.0221	0.0552	-3.1776
Prl2c3	-1.9709	3.453	-2.6282	0.0221	0.0552	-3.912
Gm27241	1.4574	-2.852	2.6274	0.0221	0.0552	-3.1562
Rasl11b	2.1772	0.4727	2.6267	0.0222	0.0553	-3.4466
Epha8	-2.3871	0.4395	-2.6262	0.0222	0.0553	-3.3202
Gm45242	1.6541	-2.5282	2.6257	0.0222	0.0554	-3.1612
Hyal1	0.9944	2.0819	2.6255	0.0222	0.0554	-3.8212
Cd3eap	-0.4168	5.3	-2.6254	0.0222	0.0554	-4.3533
Gm15832	-1.5676	-1.1389	-2.6237	0.0223	0.0556	-3.2341
Mrps28	-0.5738	4.368	-2.6229	0.0223	0.0556	-4.2257
Gm27326	-1.1788	-1.3895	-2.6215	0.0224	0.0558	-3.2691
Gm5436	-0.444	2.483	-2.6213	0.0224	0.0558	-3.8826
Asb4	3.3967	-2.9323	2.6212	0.0224	0.0558	-3.1389
Gm49494	1.3169	-1.3893	2.6212	0.0224	0.0558	-3.252
Fastkd3	-0.3955	4.4255	-2.6204	0.0224	0.0558	-4.2385
Dcn	1.715	3.9553	2.6202	0.0224	0.0559	-4.0915
Enkur	1.8542	-1.5789	2.6202	0.0224	0.0559	-3.2171
Vash1	1.8173	-2.0149	2.62	0.0224	0.0559	-3.2153
Styxl1	1.3773	-2.3785	2.6199	0.0225	0.0559	-3.1953
Slc12a9	0.5223	4.1652	2.6198	0.0225	0.0559	-4.1986
Txndc17	-0.2473	6.626	-2.6197	0.0225	0.0559	-4.4676
Gm49883	-0.7675	-0.3114	-2.6196	0.0225	0.0559	-3.378

Mydgf	-0.3072	6.503	-2.6184	0.0225	0.056	-4.4656
Eif3e	-0.3412	8.3455	-2.6177	0.0225	0.0561	-4.502
Gm6288	2.1107	-1.6425	2.6175	0.0225	0.0561	-3.1913
Prss23os	3.1359	-3.0053	2.6174	0.0226	0.0561	-3.147
Tgtp2	-1.7886	-2.32	-2.6167	0.0226	0.0561	-3.1904
Dock1	0.5798	4.9274	2.6165	0.0226	0.0561	-4.3382
Fkbp9	0.2614	7.8323	2.6156	0.0226	0.0562	-4.4996
Tbx2	1.9345	-3.5646	2.6152	0.0226	0.0563	-3.149
Pyroxd1	0.3116	4.2943	2.615	0.0227	0.0563	-4.2537
Mrps7	-0.2645	6.3866	-2.6149	0.0227	0.0563	-4.4667
C2	-1.5392	-1.3827	-2.6148	0.0227	0.0563	-3.2436
Tspan14	-0.3118	6.0515	-2.6147	0.0227	0.0563	-4.4521
Gmip	-0.4567	4.7811	-2.6138	0.0227	0.0564	-4.3074
Lrba	0.8663	4.8931	2.6135	0.0227	0.0564	-4.3356
Rpl35-ps1	-1.2946	-1.6368	-2.6131	0.0227	0.0564	-3.2681
Zfp677	0.7608	2.0913	2.6123	0.0228	0.0565	-3.8126
Dnah12	1.7523	-2.6464	2.612	0.0228	0.0565	-3.1772
Abcb1a	-0.889	4.0854	-2.612	0.0228	0.0565	-4.1502
Snord43	-1.3951	-1.771	-2.6119	0.0228	0.0565	-3.2052
Ptprs	0.3969	9.1685	2.6118	0.0228	0.0565	-4.5214
Kif1c	0.2521	7.8262	2.6118	0.0228	0.0565	-4.5066
Orm2	3.7717	-2.3401	2.6114	0.0228	0.0565	-3.1583
Gm10643	1.3867	-0.8783	2.611	0.0228	0.0566	-3.3291
Cbfa2t3	1.7076	-1.7026	2.6108	0.0228	0.0566	-3.2546
Wdr93	1.8737	-2.1249	2.6107	0.0228	0.0566	-3.199
Gng13	-1.6382	-2.7423	-2.61	0.0229	0.0567	-3.1724
Pisd-ps2	0.8029	-0.4004	2.6098	0.0229	0.0567	-3.4104
Mfsd4b3-ps	1.5175	-1.6292	2.6095	0.0229	0.0567	-3.2933
Mfsd9	0.7233	2.5619	2.6085	0.0229	0.0568	-3.9242
1190001M18Rik	-1.4154	-1.359	-2.6082	0.0229	0.0568	-3.235
Zcwpw1	0.7144	3.0961	2.6081	0.0229	0.0568	-4.0275
Gm14377	-2.6809	-2.1077	-2.6075	0.023	0.0569	-3.1696
Gpnmb	2.4442	0.8187	2.6073	0.023	0.0569	-3.5328
Ppip5k2	-0.3372	6.6558	-2.6071	0.023	0.0569	-4.4876
Emd	-0.2703	6.3828	-2.6069	0.023	0.0569	-4.4823
Gm6028	2.1294	-2.5697	2.6067	0.023	0.0569	-3.1853
0610030E20Rik	-0.319	4.5001	-2.6065	0.023	0.0569	-4.29
Timm44	-0.2524	6.1249	-2.6061	0.023	0.057	-4.4718
Dock6	0.9972	2.5718	2.6056	0.023	0.057	-3.9252
Bloc1s3	0.5358	3.1138	2.605	0.0231	0.0571	-4.0591
Zcchc24	0.3271	6.9849	2.605	0.0231	0.0571	-4.5028
Kcna4	2.0535	-1.0034	2.6049	0.0231	0.0571	-3.2988
B930036N10Rik	-1.4691	0.7301	-2.6047	0.0231	0.0571	-3.4892
Zfp689	-0.6887	1.943	-2.6041	0.0231	0.0571	-3.79
AA386476	-1.6847	-2.0508	-2.6037	0.0231	0.0572	-3.2029
Dixdc1	0.8387	4.9869	2.6033	0.0231	0.0572	-4.3704
Gpat3	0.6474	3.364	2.6029	0.0232	0.0572	-4.1005
Gm5514	1.6312	0.3747	2.6028	0.0232	0.0572	-3.5037
Pxmp2	0.5714	1.2524	2.6027	0.0232	0.0572	-3.7041
Crem	0.8126	3.3676	2.6022	0.0232	0.0573	-4.0831
Zfp467	1.1514	1.2675	2.6018	0.0232	0.0573	-3.6821
Glg1	0.3104	8.7551	2.6018	0.0232	0.0573	-4.5362

Crnkl1	0.3175	5.9126	2.601	0.0232	0.0574	-4.4726
1700055D18Rik	1.2075	-1.6554	2.6006	0.0233	0.0574	-3.3045
Avpi1	0.6106	5.7874	2.6006	0.0233	0.0574	-4.469
Trpc1	-0.6516	1.1939	-2.6005	0.0233	0.0574	-3.6558
Emc6	-0.2884	5.8821	-2.6	0.0233	0.0575	-4.4653
Sfxn1	-0.3745	7.1517	-2.5997	0.0233	0.0575	-4.5141
Ggt1	1.6952	-2.3358	2.5986	0.0233	0.0576	-3.2184
Gm17354	-0.7143	0.4486	-2.5985	0.0234	0.0576	-3.5492
Pex11a	0.4258	2.822	2.5976	0.0234	0.0577	-3.9912
Mdm2	-0.2212	5.8579	-2.5976	0.0234	0.0577	-4.4704
Gm15672	-0.9381	-1.159	-2.5975	0.0234	0.0577	-3.3211
Eps8l2	1.3669	4.996	2.5973	0.0234	0.0577	-4.3707
Gm29642	2.1695	-3.8994	2.5972	0.0234	0.0577	-3.1752
Eml4	-0.4069	6.2523	-2.5972	0.0234	0.0577	-4.4863
Ddhd1	-0.4572	4.4468	-2.5968	0.0234	0.0577	-4.291
4930579C12Rik	-2.1218	-1.1024	-2.596	0.0235	0.0578	-3.261
Tmem268	0.4672	2.1926	2.5956	0.0235	0.0578	-3.8836
AC132148.1	-1.1323	1.0843	-2.5956	0.0235	0.0578	-3.5916
Tlnrd1	-0.5637	4.7856	-2.5952	0.0235	0.0579	-4.3372
Tmem98	1.1997	2.1955	2.5944	0.0235	0.0579	-3.8519
Spsb3	0.2732	4.1609	2.5932	0.0236	0.058	-4.2647
Sav1	-0.3813	7.0924	-2.5932	0.0236	0.058	-4.5248
Gm16124	1.0218	-0.9921	2.5928	0.0236	0.0581	-3.3779
Fbxo46	0.3261	3.7419	2.5926	0.0236	0.0581	-4.1904
Casp6	0.5342	5.6028	2.5919	0.0236	0.0581	-4.4629
Sft2d1	-0.3946	4.0485	-2.5917	0.0236	0.0582	-4.228
Fam71f1	-2.4274	-2.3098	-2.5909	0.0237	0.0582	-3.1949
Atp5f1	-0.2659	8.5112	-2.5905	0.0237	0.0583	-4.5531
Arf2	0.3254	5.0031	2.5903	0.0237	0.0583	-4.4019
Ston2	1.4101	2.0653	2.5903	0.0237	0.0583	-3.7873
Padi3	-3.0234	1.2263	-2.5892	0.0238	0.0584	-3.4466
Maats1	-1.2006	-0.0113	-2.5891	0.0238	0.0584	-3.4314
Rbck1	-0.2793	6.645	-2.589	0.0238	0.0584	-4.5238
Stradb	0.4718	3.8922	2.5889	0.0238	0.0584	-4.2194
Gm4799	-1.292	-1.5276	-2.5882	0.0238	0.0585	-3.293
Zfp668	0.3485	3.9477	2.5874	0.0238	0.0585	-4.2307
Rpl6	-0.3422	9.5061	-2.5869	0.0239	0.0586	-4.5678
Ppdpf	0.4633	4.897	2.5866	0.0239	0.0586	-4.393
Tomm20	-0.2897	7.5556	-2.5857	0.0239	0.0587	-4.5479
Gm12335	-2.5189	-3.5093	-2.5857	0.0239	0.0587	-3.1912
Tle4	1.4231	1.9752	2.5854	0.0239	0.0587	-3.8293
4932438A13Rik	0.705	6.4246	2.585	0.0239	0.0588	-4.5211
Hnrnpa0	-0.3472	8.655	-2.5845	0.024	0.0588	-4.5648
Gm8141	-1.1908	-0.8337	-2.5845	0.024	0.0588	-3.3905
Fam20a	0.8365	1.813	2.5835	0.024	0.0589	-3.8123
Gm4518	-0.5941	1.5905	-2.5834	0.024	0.0589	-3.7836
Rtn4rl1	1.3838	-1.9347	2.5833	0.024	0.0589	-3.2719
Serf1	0.5505	4.6233	2.583	0.024	0.0589	-4.3616
Ttc7b	0.3551	4.9765	2.5822	0.0241	0.059	-4.4124
Lingo3	-1.8484	-3.4549	-2.5821	0.0241	0.059	-3.195
Arid2	0.3734	6.6724	2.5819	0.0241	0.059	-4.5372
Rab8b	0.4541	5.3269	2.5818	0.0241	0.059	-4.4506

Apobr	-0.557	6.0365	-2.5814	0.0241	0.059	-4.5058
Nif3l1	-0.3591	5.3501	-2.581	0.0241	0.0591	-4.4481
5033406O09Rik	-1.4846	-2.2885	-2.5809	0.0241	0.0591	-3.2495
Ppcdc	-0.4767	3.7882	-2.5793	0.0242	0.0592	-4.1898
Rraga	0.3042	5.9568	2.5789	0.0242	0.0593	-4.5125
Rgs19	-0.4537	5.2008	-2.5788	0.0242	0.0593	-4.4244
Msl3	-0.313	6.5323	-2.5785	0.0242	0.0593	-4.5362
Zbtb34	0.404	3.6436	2.5782	0.0242	0.0593	-4.1768
Zfand2b	0.526	3.8814	2.578	0.0243	0.0594	-4.235
Wdr78	0.6389	1.1847	2.5773	0.0243	0.0594	-3.7269
Dstn	0.3053	8.0868	2.577	0.0243	0.0594	-4.5724
Card10	0.613	5.4108	2.5769	0.0243	0.0594	-4.4705
Snhg3	-0.7157	3.6862	-2.5766	0.0243	0.0595	-4.1525
Smg1	0.3931	7.1887	2.5764	0.0243	0.0595	-4.5574
Slc39a7	-0.3328	4.868	-2.576	0.0243	0.0595	-4.3947
Cox4i1	0.2372	8.3767	2.5754	0.0244	0.0596	-4.5796
Adm	-2.7507	0.0722	-2.5752	0.0244	0.0596	-3.3877
Rnf181	0.3976	6.0733	2.5749	0.0244	0.0596	-4.5297
Vcpip1	0.3634	6.2998	2.5746	0.0244	0.0596	-4.535
Casp9	0.4302	4.2093	2.5745	0.0244	0.0596	-4.3119
Gcnt4	-0.906	3.5386	-2.5735	0.0245	0.0597	-4.0921
Tssc4	-0.3041	4.5628	-2.573	0.0245	0.0598	-4.3579
Ranbp10	0.289	5.8929	2.5721	0.0245	0.0599	-4.5207
Gm5857	1.3093	-1.5576	2.5714	0.0245	0.0599	-3.3033
Ssna1	-0.3234	5.2909	-2.5704	0.0246	0.06	-4.4681
Top2b	0.2941	7.8439	2.5694	0.0246	0.0601	-4.5823
Ptk2	-0.3568	7.2391	-2.5692	0.0246	0.0602	-4.5713
Gm5815	1.9205	-3.6376	2.5688	0.0247	0.0602	-3.215
Magt1	-0.3048	7.4231	-2.5677	0.0247	0.0603	-4.5772
Plcb4	1.1231	2.0584	2.567	0.0247	0.0604	-3.8915
Tpmt	0.7028	3.4131	2.5667	0.0248	0.0604	-4.1687
Gm807	-1.4059	1.8062	-2.5662	0.0248	0.0605	-3.7633
1700020D05Rik	1.8411	-2.0928	2.5656	0.0248	0.0605	-3.2842
Vkorc1l1	-0.2842	6.0137	-2.5654	0.0248	0.0605	-4.5329
Eid2b	0.7297	0.6475	2.5642	0.0249	0.0606	-3.6461
Tmem68	-0.465	4.3928	-2.564	0.0249	0.0606	-4.3269
Pla2g3	2.3726	-1.1806	2.564	0.0249	0.0606	-3.3108
Pgap1	0.6897	3.862	2.564	0.0249	0.0606	-4.2486
Serinc2	-1.865	0.0223	-2.5636	0.0249	0.0607	-3.4497
Fam219a	1.2406	1.8903	2.5633	0.0249	0.0607	-3.8753
Bcl2l15	1.3993	-2.4003	2.5632	0.0249	0.0607	-3.2499
Gm35315	-0.832	1.8988	-2.563	0.0249	0.0607	-3.821
Cdv3-ps	-1.4376	-1.2763	-2.5622	0.025	0.0608	-3.334
Kctd15	0.3932	5.2806	2.5622	0.025	0.0608	-4.4852
Ccdc32	0.3861	3.632	2.5621	0.025	0.0608	-4.2287
Gm47767	1.1278	-0.7304	2.5616	0.025	0.0608	-3.4591
Gmppb	0.3257	4.1836	2.5613	0.025	0.0609	-4.3175
Gpr3	-2.7179	-2.5938	-2.5611	0.025	0.0609	-3.2351
Slc43a2	1.7095	4.1616	2.5593	0.0251	0.0611	-4.2439
Mical2	-0.8231	5.3417	-2.5592	0.0251	0.0611	-4.4593
Rph3al	-2.0816	-1.035	-2.5588	0.0251	0.0611	-3.3254
Gm35330	2.595	-1.6839	2.5585	0.0251	0.0611	-3.2777

Chd2	0.356	6.0031	2.5584	0.0251	0.0611	-4.5538
Gm10157	1.1361	-1.0912	2.5584	0.0251	0.0611	-3.3971
5430405H02Rik	0.5696	1.5228	2.558	0.0252	0.0612	-3.8311
Whamm	0.2738	3.7989	2.5575	0.0252	0.0612	-4.2721
Greb1	1.2599	1.4179	2.5571	0.0252	0.0613	-3.7966
Itprip	-0.5354	5.0529	-2.5571	0.0252	0.0613	-4.4526
Gm5617	0.6797	0.2965	2.5567	0.0252	0.0613	-3.628
Ccdc43	-0.328	5.7301	-2.5559	0.0253	0.0614	-4.5291
Tspo	0.979	6.5986	2.5557	0.0253	0.0614	-4.5838
Zfp623	0.3276	4.1041	2.5556	0.0253	0.0614	-4.3122
Gm7143	-0.9428	0.466	-2.5556	0.0253	0.0614	-3.625
Golm1	-0.36	6.2767	-2.5556	0.0253	0.0614	-4.563
Upp2	0.8052	0.7948	2.5548	0.0253	0.0614	-3.7156
Arhgap10	0.4244	6.607	2.5548	0.0253	0.0614	-4.5849
Ctdnep1	-0.2188	6.8374	-2.5538	0.0254	0.0615	-4.5908
Spcs1	0.2303	6.795	2.5535	0.0254	0.0616	-4.5915
Nrde2	0.2883	5.0099	2.5531	0.0254	0.0616	-4.4699
Gm16229	-1.8399	-1.8541	-2.5531	0.0254	0.0616	-3.3014
Eps15l1	0.2609	5.8854	2.5528	0.0254	0.0616	-4.5546
Fam102b	0.2998	7.4147	2.5525	0.0254	0.0616	-4.6056
Hist1h3c	-1.5361	-1.4505	-2.5517	0.0254	0.0617	-3.3442
Prag1	0.4415	5.5007	2.5516	0.0255	0.0617	-4.5198
4833445I07Rik	1.1406	-1.5759	2.5516	0.0255	0.0617	-3.3743
Efemp2	0.4763	6.7736	2.5511	0.0255	0.0618	-4.5959
Ndufv2	-0.2703	6.8266	-2.5507	0.0255	0.0618	-4.5966
Fer	0.4171	4.9904	2.5507	0.0255	0.0618	-4.4745
Med13l	0.3195	7.2641	2.5507	0.0255	0.0618	-4.6062
Apobec1	0.7235	2.4768	2.5503	0.0255	0.0618	-3.994
Ice2	-0.3073	4.5375	-2.5502	0.0255	0.0618	-4.3862
Tmem106c	0.5774	4.6379	2.5501	0.0255	0.0618	-4.4215
Gm23723	-1.9925	-3.7938	-2.5499	0.0255	0.0618	-3.2418
Dpp3	0.3113	6.4165	2.5494	0.0256	0.0619	-4.5902
Gramd1a	0.4074	5.3441	2.5488	0.0256	0.062	-4.5199
Rabgap1l	0.562	4.7222	2.5482	0.0256	0.062	-4.43
Wdr27	0.94	-0.3781	2.5479	0.0256	0.062	-3.5034
Bco1	1.6384	-3.1866	2.5476	0.0256	0.0621	-3.2749
Abhd6	0.6088	2.5828	2.5474	0.0256	0.0621	-4.028
Mir3091	-1.1681	-1.8379	-2.5465	0.0257	0.0622	-3.3238
Pold4	-0.716	4.4545	-2.5462	0.0257	0.0622	-4.3727
Pkig	-0.4876	4.1012	-2.546	0.0257	0.0622	-4.3133
Scand1	0.4274	5.251	2.5459	0.0257	0.0622	-4.5151
Ctdsp1	0.2735	6.7658	2.5456	0.0257	0.0622	-4.6044
Pcm1	-0.4498	7.5423	-2.5454	0.0257	0.0623	-4.6179
Ube2e3	0.2507	6.1084	2.5452	0.0258	0.0623	-4.5804
Zfhx2os	1.6231	-2.026	2.5452	0.0258	0.0623	-3.3275
Gt(ROSA)26Sor	-0.6285	2.6987	-2.5451	0.0258	0.0623	-4.0088
Pitpnm1	-0.3176	5.5965	-2.5449	0.0258	0.0623	-4.5399
Cpsf2	-0.3227	7.3162	-2.5449	0.0258	0.0623	-4.6156
Gm10509	1.0386	-0.2761	2.5448	0.0258	0.0623	-3.5081
Ppp4r3a	-0.3198	6.793	-2.5447	0.0258	0.0623	-4.6033
Fnbp1l	0.4307	6.8092	2.5445	0.0258	0.0623	-4.6084
Gm4202	-0.4641	1.7339	-2.5441	0.0258	0.0623	-3.8543

Thoc1	-0.4445	6.212	-2.5435	0.0258	0.0624	-4.5809
P4ha3	1.8596	0.4792	2.5432	0.0259	0.0624	-3.6227
Cnot8	0.24	6.2669	2.5431	0.0259	0.0624	-4.5942
Ift20	0.3564	4.8028	2.5429	0.0259	0.0624	-4.4585
Slc25a29	-0.606	3.9431	-2.5429	0.0259	0.0624	-4.2757
Lrpprc	-0.3124	7.3657	-2.5425	0.0259	0.0624	-4.6209
Tnfaip6	2.0406	0.4014	2.5422	0.0259	0.0625	-3.555
Gm26850	1.1919	-0.9658	2.5421	0.0259	0.0625	-3.4282
Ccdc80	3.4601	0.0082	2.542	0.0259	0.0625	-3.4045
Gm2467	-1.7713	-2.2343	-2.5419	0.0259	0.0625	-3.3132
Nfe2l3	2.4224	-2.1515	2.5418	0.0259	0.0625	-3.3013
Cystm1	0.5124	3.9614	2.5417	0.0259	0.0625	-4.3288
Gm43328	-1.1126	-1.1261	-2.5416	0.0259	0.0625	-3.3936
Dnajb4	0.617	5.6928	2.5413	0.0259	0.0625	-4.5614
Zfp74	0.3676	3.5255	2.541	0.026	0.0625	-4.2393
Nop53	-0.2682	6.7176	-2.5408	0.026	0.0626	-4.612
Zfp866	0.3754	3.9238	2.54	0.026	0.0626	-4.3161
4930478M13Rik	1.3681	-0.8187	2.5399	0.026	0.0627	-3.481
Slc12a5	1.0598	0.0074	2.5397	0.026	0.0627	-3.6059
Acbd3	-0.2839	6.2757	-2.5394	0.026	0.0627	-4.5965
Utp14a	-0.2836	6.012	-2.5392	0.026	0.0627	-4.5807
Gng11	1.0549	3.3565	2.5391	0.026	0.0627	-4.2049
Ccdc106	1.9123	-3.0981	2.539	0.0261	0.0627	-3.2639
Gm17134	-1.5828	-2.6078	-2.5374	0.0261	0.0629	-3.2949
Laptm4b	0.478	6.2209	2.5374	0.0261	0.0629	-4.5998
Dedd2	0.4735	3.7972	2.5371	0.0261	0.0629	-4.2963
Cdk9	-0.2591	6.3655	-2.5366	0.0262	0.0629	-4.6049
Zfp808	-0.6516	2.4842	-2.5364	0.0262	0.063	-3.9876
Gm44423	1.3847	-2.506	2.5347	0.0263	0.0631	-3.3127
Fan1	0.449	2.4534	2.5344	0.0263	0.0632	-4.0374
Sos2	-0.3437	5.2657	-2.5344	0.0263	0.0632	-4.5177
Apoo-ps	-1.0694	-0.8682	-2.5343	0.0263	0.0632	-3.4469
Zfp324	0.4616	3.2691	2.534	0.0263	0.0632	-4.2068
Tmem219	0.4972	3.0549	2.5317	0.0264	0.0635	-4.1733
Clvs1	-1.6126	-2.0469	-2.5316	0.0264	0.0635	-3.3614
Atg9a	0.4266	5.0068	2.5314	0.0264	0.0635	-4.5074
Wwox	0.4748	3.5003	2.5313	0.0264	0.0635	-4.2482
Zfp707	0.4313	2.7492	2.5306	0.0265	0.0635	-4.0973
Taf1a	-0.441	3.8445	-2.5306	0.0265	0.0635	-4.2878
H2afj	-0.4124	5.8235	-2.5305	0.0265	0.0635	-4.5838
Rn7sk	0.7867	2.8147	2.5302	0.0265	0.0636	-4.0874
Gm5801	-0.6098	1.564	-2.5294	0.0265	0.0636	-3.8465
Taf9	-0.5435	1.644	-2.5289	0.0265	0.0637	-3.8564
Itih2	1.2613	2.3405	2.5289	0.0265	0.0637	-3.9431
Gm37084	1.5167	-2.2915	2.5287	0.0265	0.0637	-3.3411
Adtrp	3.7689	-2.3282	2.5286	0.0266	0.0637	-3.2712
Poglut1	0.2275	6.0148	2.5284	0.0266	0.0637	-4.603
Ecm2	1.9446	-2.3029	2.5283	0.0266	0.0637	-3.3108
Tfcp2l1	2.1322	-2.2731	2.5273	0.0266	0.0638	-3.2989
Cfap53	1.761	-2.2059	2.5272	0.0266	0.0638	-3.3123
1300017J02Rik	1.565	-2.3093	2.5272	0.0266	0.0638	-3.3592
Cx3cl1	1.1897	5.4487	2.5268	0.0266	0.0639	-4.5539

Tmem101	0.2944	4.5187	2.5249	0.0267	0.0641	-4.4509
Gm18541	-1.2092	-2.2278	-2.5247	0.0267	0.0641	-3.3626
Cd248	2.1498	0.7825	2.5247	0.0267	0.0641	-3.6198
Tlk2	-0.2051	6.6538	-2.5245	0.0268	0.0641	-4.6378
Gm17509	1.4155	-0.9196	2.5245	0.0268	0.0641	-3.4948
Zfp800	-0.3537	4.5318	-2.5243	0.0268	0.0641	-4.4175
BC005624	0.253	5.4823	2.5242	0.0268	0.0641	-4.5708
Ramp1	1.4428	0.1204	2.5238	0.0268	0.0641	-3.6107
Cdc37l1	0.4561	5.4238	2.523	0.0268	0.0642	-4.5651
Zfp180	-0.2919	4.6518	-2.5229	0.0268	0.0642	-4.4581
09-sept	-0.3729	9.0871	-2.5228	0.0268	0.0642	-4.6788
Ndufb8	-0.245	7.0111	-2.5228	0.0268	0.0642	-4.6509
Gm4262	0.7144	0.6558	2.5224	0.0269	0.0643	-3.7093
Aldh3b2	1.8516	-0.9375	2.5223	0.0269	0.0643	-3.4987
Ly6g	-2.8832	-3.6559	-2.522	0.0269	0.0643	-3.2837
Ap3b1	0.3373	6.9657	2.5219	0.0269	0.0643	-4.6522
Gm9755	-0.8336	-0.41	-2.5219	0.0269	0.0643	-3.5739
Gm22826	-1.9945	-3.7975	-2.5217	0.0269	0.0643	-3.2824
Srek1ip1	0.3052	4.8947	2.5213	0.0269	0.0643	-4.5069
Dtd1	0.4175	3.6023	2.5209	0.0269	0.0644	-4.2886
Gm15590	1.7406	-1.9262	2.5206	0.0269	0.0644	-3.3601
Canx	-0.2855	9.8499	-2.5205	0.027	0.0644	-4.6878
Gm2962	-1.3659	-1.9864	-2.5194	0.027	0.0645	-3.3788
Gm10254	-1.0814	-0.4641	-2.5193	0.027	0.0645	-3.5181
2510039O18Rik	0.2571	5.8753	2.5184	0.0271	0.0646	-4.6151
Golga2	0.3254	6.6452	2.5177	0.0271	0.0647	-4.6519
Tsr3	-0.2669	4.9393	-2.5174	0.0271	0.0647	-4.5223
Cand1	-0.2186	7.6844	-2.517	0.0271	0.0648	-4.6713
Mccc2	0.3109	5.0811	2.5167	0.0271	0.0648	-4.5425
Gm49745	1.374	-2.7772	2.5166	0.0271	0.0648	-3.3041
Actn4	-0.3599	8.8889	-2.5163	0.0272	0.0648	-4.689
Asprv1	1.8627	-3.2753	2.5162	0.0272	0.0648	-3.2927
Phf8	0.7959	5.6491	2.5161	0.0272	0.0648	-4.6001
Med21	-0.3413	4.9875	-2.5161	0.0272	0.0648	-4.5173
Sypl	-0.2966	6.333	-2.5159	0.0272	0.0648	-4.6402
Ipmk	-0.4777	6.3838	-2.5158	0.0272	0.0648	-4.6409
Rps27l	-0.3264	6.8946	-2.5157	0.0272	0.0648	-4.6598
Slx1b	-0.5215	2.2504	-2.5151	0.0272	0.0649	-4.0117
Rps4x-ps	-0.709	0.3101	-2.5146	0.0272	0.065	-3.6962
Cep19	0.3298	3.8909	2.5144	0.0273	0.065	-4.3442
Paqr7	0.7274	2.8279	2.5142	0.0273	0.065	-4.1341
Gtpbp8	0.5388	3.0683	2.5142	0.0273	0.065	-4.1816
Ndufab1-ps	-1.5371	-2.4013	-2.5139	0.0273	0.065	-3.344
Slfn8	0.5771	2.6683	2.5137	0.0273	0.065	-4.0962
Cox7a1	1.3819	-0.7906	2.5132	0.0273	0.0651	-3.4811
Ppp1r7	-0.2975	6.2928	-2.5109	0.0274	0.0653	-4.6472
A730063M14Rik	0.8484	-0.6419	2.5107	0.0274	0.0653	-3.5571
Stim1	0.4132	5.1153	2.5105	0.0274	0.0654	-4.552
Snora73b	-1.0158	0.2658	-2.5104	0.0275	0.0654	-3.6504
Cpne3	0.3436	6.2017	2.5095	0.0275	0.0655	-4.647
Gm42788	1.9696	-1.5764	2.5086	0.0275	0.0656	-3.4353
Lamb3	2.7555	0.7946	2.5085	0.0275	0.0656	-3.6277

Aox1	0.8877	4.2293	2.5085	0.0276	0.0656	-4.4107
2010315B03Rik	0.5909	2.7366	2.5084	0.0276	0.0656	-4.1292
Zfp974	0.8163	1.1407	2.5084	0.0276	0.0656	-3.859
Gm29668	-1.8732	-0.8795	-2.5082	0.0276	0.0656	-3.4354
Pdia4	-0.3105	8.1918	-2.5081	0.0276	0.0656	-4.6958
Ppm1b	-0.2618	5.9896	-2.5079	0.0276	0.0656	-4.6373
Gm49327	0.7783	0.6127	2.5079	0.0276	0.0656	-3.7664
Ltbr	0.2705	6.278	2.5073	0.0276	0.0656	-4.6584
Mapkapk5	-0.5021	2.3958	-2.5067	0.0276	0.0657	-4.0457
Pigx	0.3571	4.3709	2.5061	0.0277	0.0658	-4.4516
9130019O22Rik	0.7803	0.7686	2.5057	0.0277	0.0658	-3.76
Erf	-0.2969	6.1819	-2.5056	0.0277	0.0658	-4.6498
Cep97	0.3893	3.6014	2.5054	0.0277	0.0658	-4.3119
Gm5113	0.5727	1.8843	2.5044	0.0278	0.0659	-4
Gm1821	0.4823	2.0929	2.5044	0.0278	0.0659	-4.0286
Fxn	-0.4581	3.7202	-2.504	0.0278	0.066	-4.3123
Gm17315	1.534	-3.4036	2.5036	0.0278	0.066	-3.3336
Mrpl36	0.3209	5.0944	2.5032	0.0278	0.066	-4.5622
Tnfaip1	-0.2459	7.1018	-2.5029	0.0278	0.0661	-4.6873
Agps	-0.3824	6.3525	-2.5027	0.0278	0.0661	-4.6628
Dram2	0.3913	4.8959	2.5024	0.0279	0.0661	-4.5397
Krt15	1.8916	-1.8244	2.5021	0.0279	0.0661	-3.4235
Gm41396	-1.3611	-1.759	-2.5016	0.0279	0.0662	-3.3805
Pfdn1	-0.3182	6.7805	-2.5015	0.0279	0.0662	-4.6827
Eya3	-0.2388	5.8576	-2.5014	0.0279	0.0662	-4.6383
Wdr91	0.4691	3.7089	2.5011	0.0279	0.0662	-4.3431
Sntb1	3.1082	-0.7631	2.501	0.0279	0.0662	-3.4376
Dlec1	2.2395	-2.9701	2.5004	0.028	0.0663	-3.3204
Tnks	0.4246	7.3047	2.5002	0.028	0.0663	-4.6962
Zkscan8	0.3092	4.6655	2.5001	0.028	0.0663	-4.5028
Sptan1	0.3305	8.7822	2.4999	0.028	0.0663	-4.7174
Gm43375	-1.4866	-0.9703	-2.4993	0.028	0.0664	-3.4503
Hspg2	0.6324	8.786	2.4993	0.028	0.0664	-4.718
Fam114a2	0.2831	6.3433	2.4989	0.028	0.0664	-4.675
Bok	-1.0931	4.565	-2.4982	0.0281	0.0665	-4.421
Dnajb1	0.2818	5.9512	2.4981	0.0281	0.0665	-4.6551
Uspl1	-0.3407	5.4196	-2.4974	0.0281	0.0666	-4.599
Sh2b1	0.2653	5.8946	2.497	0.0281	0.0666	-4.6556
Chd1l	-0.4325	5.2757	-2.497	0.0281	0.0666	-4.5859
Pofut1	-0.2918	5.6979	-2.4964	0.0282	0.0667	-4.6352
Gm4335	-1.1052	-1.1208	-2.4962	0.0282	0.0667	-3.4847
Snip1	-0.2985	4.8617	-2.4958	0.0282	0.0667	-4.5369
Pja1	-0.3832	6.5686	-2.4955	0.0282	0.0668	-4.6857
Zmym6	0.5734	3.4651	2.4949	0.0282	0.0668	-4.2895
Pcnx	0.4349	5.8402	2.4944	0.0283	0.0669	-4.6489
Gm11539	-0.7622	-0.5223	-2.4937	0.0283	0.0669	-3.571
Gm28818	2.1078	-1.8877	2.4929	0.0283	0.067	-3.4066
Gm5847	1.3877	-2.6575	2.4928	0.0284	0.067	-3.3616
Gm11218	2.495	-4.0909	2.4918	0.0284	0.0672	-3.3273
Abitram	-0.3121	5.0056	-2.4916	0.0284	0.0672	-4.5595
Sult2b1	-1.276	0.2786	-2.4913	0.0284	0.0672	-3.6688
Abcb7	-0.3651	6.3163	-2.4908	0.0285	0.0672	-4.6805

Gm45833	-1.116	-1.7128	-2.4907	0.0285	0.0673	-3.4814
Tmem185a	0.2695	5.1665	2.4906	0.0285	0.0673	-4.6031
Pde4a	-0.5327	5.3769	-2.4901	0.0285	0.0673	-4.6017
Tex26	1.4655	-2.2874	2.4899	0.0285	0.0673	-3.4336
Pts	0.4838	3.9721	2.4895	0.0285	0.0674	-4.417
Mansc1	2.0589	-1.6523	2.4893	0.0285	0.0674	-3.4125
Gm14633	-1.1477	-0.5971	-2.4892	0.0285	0.0674	-3.5451
Pkn2	-0.2625	7.6388	-2.489	0.0286	0.0674	-4.7201
Gm7591	-1.838	-2.2359	-2.4889	0.0286	0.0674	-3.3699
Ier5	-0.5548	6.6362	-2.4886	0.0286	0.0674	-4.6955
Snx3	-0.246	7.0332	-2.4885	0.0286	0.0674	-4.7115
Msh2	-0.3198	6.6807	-2.4875	0.0286	0.0675	-4.7031
Ulk3	-0.5269	3.2532	-2.4869	0.0287	0.0676	-4.2576
Hspb11	0.4551	3.3145	2.4867	0.0287	0.0676	-4.301
Gm20548	1.031	-2.339	2.4863	0.0287	0.0677	-3.4004
Slc37a2	0.7745	0.9086	2.4862	0.0287	0.0677	-3.8396
Gm45222	1.8589	-3.4185	2.4861	0.0287	0.0677	-3.3361
Halr1	4.4196	-2.7396	2.4857	0.0287	0.0677	-3.3303
Catsperg1	2.0139	-3.0083	2.4849	0.0288	0.0678	-3.3526
Ttbk1	2.0044	-3.0214	2.4846	0.0288	0.0678	-3.3435
Zfp444	0.2582	4.2843	2.4845	0.0288	0.0678	-4.477
Best2	1.6078	-3.0198	2.4836	0.0288	0.0679	-3.3444
Eif4a1	-0.3301	10.2547	-2.4831	0.0289	0.068	-4.756
Gm6722	-1.7565	-2.6102	-2.483	0.0289	0.068	-3.3644
Pak1	0.5706	5.3702	2.4829	0.0289	0.068	-4.6393
B230206H07Rik	2.1279	-1.7575	2.4828	0.0289	0.068	-3.4316
Rrm2b	0.3865	4.5153	2.4826	0.0289	0.068	-4.5234
Lrrc8a	0.3178	4.5371	2.4825	0.0289	0.068	-4.5198
Mecom	0.8902	1.8477	2.4824	0.0289	0.068	-3.967
C1rl	3.2357	-0.2694	2.4819	0.0289	0.0681	-3.5115
CR555305.2	-1.1476	-0.1786	-2.4817	0.0289	0.0681	-3.633
Gm8623	-1.3312	-1.2313	-2.4817	0.0289	0.0681	-3.508
Bclaf1	-0.2668	7.8153	-2.4806	0.029	0.0682	-4.7375
Chkb	0.4105	3.079	2.4805	0.029	0.0682	-4.2572
Slc35b2	0.312	6.4062	2.4799	0.029	0.0683	-4.7103
Ccdc59	-0.3861	5.08	-2.4798	0.029	0.0683	-4.5939
Zfp994	0.5983	3.0978	2.4794	0.0291	0.0683	-4.248
Gipc1	0.3459	5.7456	2.4793	0.0291	0.0683	-4.678
Naa40	-0.3836	5.6101	-2.4793	0.0291	0.0683	-4.6502
Ankrd54	-0.3678	4.9972	-2.4792	0.0291	0.0683	-4.5892
Rab27b	-0.5235	2.8542	-2.4788	0.0291	0.0683	-4.199
Unc13c	-1.3494	-1.9023	-2.4783	0.0291	0.0684	-3.3969
Cmpk2	2.482	-0.3742	2.4782	0.0291	0.0684	-3.546
Faf2	0.2686	7.0073	2.4775	0.0292	0.0685	-4.7307
Gm14168	1.9794	-1.959	2.477	0.0292	0.0685	-3.396
Ppp1r35	-0.32	3.2641	-2.4757	0.0293	0.0687	-4.3049
Zfp958	-0.6119	3.3839	-2.4755	0.0293	0.0687	-4.2765
Cdc73	-0.3537	6.5487	-2.4751	0.0293	0.0688	-4.7183
Hist1h2af	-1.513	-2.13	-2.4749	0.0293	0.0688	-3.42
Frmd4b	1.4836	0.145	2.4748	0.0293	0.0688	-3.6677
1810012K08Rik	1.3195	-2.5405	2.4747	0.0293	0.0688	-3.4154
Tada3	0.25	5.4593	2.4744	0.0293	0.0688	-4.6573

Dnajc5	0.3087	7.0914	2.4742	0.0293	0.0688	-4.7386
Gm4610	-2.0328	0.5041	-2.4742	0.0293	0.0688	-3.6973
Vps37d	0.6187	0.7161	2.4734	0.0294	0.0689	-3.8245
Rrad	0.5379	3.8279	2.4734	0.0294	0.0689	-4.427
Ccdc127	-0.2474	6.508	-2.4733	0.0294	0.0689	-4.7231
Sppl2b	-0.2629	4.4406	-2.4732	0.0294	0.0689	-4.5057
Gm45220	1.903	-3.0111	2.4729	0.0294	0.0689	-3.3703
Gm47233	-1.2853	-2.1812	-2.4729	0.0294	0.0689	-3.4237
Pou2f2	-2.4131	-2.6645	-2.4728	0.0294	0.0689	-3.3705
Gm11946	-0.9416	-0.0887	-2.4728	0.0294	0.0689	-3.6633
Emp2	-0.6663	6.8144	-2.4723	0.0294	0.069	-4.7331
Evi5	-0.2897	6.647	-2.4723	0.0294	0.069	-4.7288
Mirt1	-1.0776	0.321	-2.4721	0.0294	0.069	-3.7212
Sacs	-0.5189	5.6527	-2.472	0.0295	0.069	-4.659
Spns3	2.3844	-1.4761	2.4714	0.0295	0.069	-3.4767
Commd7	0.3342	4.359	2.4697	0.0296	0.0692	-4.5182
Ptdss1	0.2609	6.5924	2.4695	0.0296	0.0693	-4.7348
Abhd14b	0.56	3.3124	2.4686	0.0296	0.0694	-4.3236
Gm11960	-2.0132	-2.9256	-2.4684	0.0296	0.0694	-3.3723
Gpaa1	-0.401	5.23	-2.4676	0.0297	0.0695	-4.6286
Uprt	0.2661	4.7152	2.4668	0.0297	0.0696	-4.5738
Papss2	2.6504	-0.5139	2.4667	0.0297	0.0696	-3.5226
Gm42743	1.2909	-0.7286	2.4666	0.0297	0.0696	-3.6032
R3hdm1	-0.253	6.9288	-2.4653	0.0298	0.0697	-4.7481
Slc25a43	-1.3716	-0.3828	-2.4649	0.0298	0.0698	-3.5744
Cptp	-0.2714	4.517	-2.4644	0.0299	0.0698	-4.5445
G3bp2	-0.2507	8.0182	-2.4643	0.0299	0.0698	-4.7696
Tmed9	-0.309	7.7132	-2.4637	0.0299	0.0699	-4.7663
Rybp	-0.2547	5.2719	-2.4637	0.0299	0.0699	-4.6476
Samm50	-0.3384	7.0221	-2.4637	0.0299	0.0699	-4.7544
Anpep	1.22	2.904	2.4632	0.0299	0.0699	-4.2456
Gm10293	-0.765	0.0851	-2.4631	0.0299	0.0699	-3.71
Gm9828	0.6832	1.4317	2.4628	0.03	0.07	-3.9824
Ephx2	2.2772	-0.42	2.4625	0.03	0.07	-3.6315
P2ry6	-1.9321	-1.4608	-2.4619	0.03	0.0701	-3.4445
Slc24a3	3.182	-2.1132	2.4614	0.03	0.0701	-3.4109
Fam192a	0.2561	5.7861	2.461	0.0301	0.0702	-4.7118
Dleu2	0.7164	2.2331	2.4606	0.0301	0.0702	-4.1363
Rnase1	3.1751	-3.803	2.4604	0.0301	0.0702	-3.372
Amacr	0.5828	3.4184	2.4601	0.0301	0.0702	-4.3437
Pim2	0.4078	3.9682	2.4598	0.0301	0.0703	-4.4621
Dcbld2	-0.3851	6.5914	-2.4598	0.0301	0.0703	-4.7491
Gm5874	-0.6383	1.1514	-2.4596	0.0301	0.0703	-3.876
Sapcd1	-1.7135	-1.4211	-2.4594	0.0301	0.0703	-3.4759
Bola2	-0.316	5.0405	-2.4594	0.0301	0.0703	-4.63
Kremen1	0.3413	5.3043	2.4592	0.0302	0.0703	-4.6719
Gm38037	-0.9896	-0.1956	-2.4585	0.0302	0.0704	-3.627
Ilf3	-0.2607	7.2114	-2.458	0.0302	0.0704	-4.7678
Gorasp2	-0.2729	7.3553	-2.4579	0.0302	0.0704	-4.7707
Zfp605	0.5216	4.358	2.4575	0.0302	0.0705	-4.5434
Khdc4	-0.3122	6.051	-2.4569	0.0303	0.0706	-4.7253
Bbs5	-0.6173	2.3083	-2.4568	0.0303	0.0706	-4.1278

Hipk2	0.496	6.0233	2.4565	0.0303	0.0706	-4.7289
Cherp	-0.3433	6.9518	-2.4559	0.0303	0.0707	-4.7644
Gm12583	-1.321	-1.9126	-2.4553	0.0304	0.0707	-3.491
Smim10l1	0.4491	5.7819	2.4553	0.0304	0.0707	-4.7164
Bpifb4	-1.1728	1.1908	-2.4552	0.0304	0.0707	-3.8875
Rps6ka2	0.4554	4.4037	2.4551	0.0304	0.0707	-4.5577
Vps51	0.2703	5.2082	2.4549	0.0304	0.0707	-4.6702
Gm6254	-1.2676	-0.6307	-2.4546	0.0304	0.0708	-3.5853
Cd74	5.3936	-1.3069	2.4543	0.0304	0.0708	-3.3744
Gdf9	1.0619	-1.8619	2.4542	0.0304	0.0708	-3.5475
Hadh	0.2474	6.815	2.454	0.0304	0.0708	-4.7686
Tln1	0.3023	8.3768	2.4537	0.0305	0.0708	-4.7948
Tnni2	1.6745	-0.8021	2.4536	0.0305	0.0708	-3.5762
Vkorc1	0.341	4.0401	2.4533	0.0305	0.0709	-4.4836
Smap1	0.2795	6.1568	2.4533	0.0305	0.0709	-4.7478
Vti1a	0.3402	4.5774	2.4532	0.0305	0.0709	-4.5809
Chd3	0.5627	6.7442	2.4529	0.0305	0.0709	-4.7689
Henmt1	1.4953	-1.5159	2.4528	0.0305	0.0709	-3.5206
Prkce	0.4353	4.3228	2.4526	0.0305	0.0709	-4.537
Rdx	-0.2982	8.1916	-2.4521	0.0305	0.071	-4.7937
Cdpf1	0.457	3.01	2.4518	0.0306	0.071	-4.29
Fmo1	6.5333	-1.1112	2.4517	0.0306	0.071	-3.3739
Itgbl1	5.7731	-0.7089	2.4516	0.0306	0.071	-3.3986
Synm	3.1283	-0.5806	2.4513	0.0306	0.071	-3.5202
2310011J03Rik	0.3826	4.3372	2.4508	0.0306	0.0711	-4.5466
1810008I18Rik	-1.676	-2.5356	-2.4506	0.0306	0.0711	-3.4062
Gm11772	1.349	-1.5566	2.4504	0.0306	0.0711	-3.4759
Brip1os	-0.3444	6.6191	-2.4503	0.0306	0.0711	-4.7661
Olfr920	2.4094	-3.1794	2.4503	0.0306	0.0711	-3.3985
2210011K15Rik	2.034	-2.5941	2.4497	0.0307	0.0712	-3.4303
Gosr1	0.2226	6.0091	2.4497	0.0307	0.0712	-4.7444
Fam3a	0.4842	4.5416	2.4494	0.0307	0.0712	-4.578
Slx4ip	0.509	3.1615	2.4489	0.0307	0.0713	-4.3205
Celf5	1.9463	-2.3697	2.4489	0.0307	0.0713	-3.4507
Ppp1r3f	-0.7674	0.2998	-2.4486	0.0307	0.0713	-3.7709
Btrc	0.3909	4.3444	2.4484	0.0308	0.0713	-4.5514
Gm45237	-1.2455	-2.315	-2.4483	0.0308	0.0713	-3.4598
Gnai3	-0.2198	8.0407	-2.4472	0.0308	0.0714	-4.8004
1810026B05Rik	0.4336	3.1976	2.4465	0.0309	0.0715	-4.3195
CT010467.1	0.6212	5.2316	2.4461	0.0309	0.0716	-4.6877
Tnfsf9	-1.649	1.2512	-2.4455	0.0309	0.0716	-3.8278
Abhd8	0.3368	5.6752	2.4444	0.031	0.0718	-4.7283
Ddt	0.4141	4.0395	2.4439	0.031	0.0718	-4.5137
Gm6649	1.5804	-2.5799	2.4438	0.031	0.0718	-3.4647
Gm6548	0.4695	2.9651	2.4435	0.031	0.0719	-4.2976
Glis3	-0.4194	6.1338	-2.4434	0.031	0.0719	-4.7541
Cpe	0.7223	9.1102	2.4428	0.0311	0.0719	-4.8211
Snord58b	-1.8307	-1.6987	-2.442	0.0311	0.072	-3.4683
Calr-ps	1.8257	-2.3703	2.4419	0.0311	0.072	-3.4478
Cxcl16	1.3827	2.7691	2.4419	0.0311	0.072	-4.2273
Map4	0.2786	7.8346	2.4417	0.0311	0.072	-4.8087
Sec11a	-0.2992	6.1342	-2.4412	0.0312	0.0721	-4.7622

Sec14l1	0.4269	7.7999	2.4405	0.0312	0.0722	-4.8104
Arl5a	-0.382	6.5812	-2.44	0.0312	0.0722	-4.7812
Fam83f	2.0208	-3.6294	2.4399	0.0312	0.0722	-3.4009
Ndufa13	0.2602	6.3075	2.4396	0.0312	0.0722	-4.7802
Aldh9a1	-0.2615	6.7016	-2.4396	0.0312	0.0722	-4.7897
Uxt	-0.3861	3.6011	-2.4393	0.0313	0.0723	-4.4225
Dgkh	0.8752	3.3385	2.4393	0.0313	0.0723	-4.3257
Hmgxb4	-0.3065	4.9019	-2.4385	0.0313	0.0724	-4.6479
Mtfp1	-0.8264	2.2949	-2.4385	0.0313	0.0724	-4.1385
S100a8	-3.3128	2.3826	-2.4382	0.0313	0.0724	-3.8292
Mgarp	-1.2118	0.3832	-2.4379	0.0313	0.0724	-3.7312
Galt	0.5226	3.1024	2.4378	0.0314	0.0724	-4.3387
Gm10734	-0.8557	-0.2703	-2.4376	0.0314	0.0724	-3.6611
Mepce	0.2747	5.9655	2.4375	0.0314	0.0724	-4.7629
Zbtb18	0.3284	5.576	2.4366	0.0314	0.0725	-4.7346
March9	0.9447	2.5666	2.4365	0.0314	0.0725	-4.2404
Bckdhb	0.5129	4.351	2.4354	0.0315	0.0727	-4.5739
Hspa1a	0.7688	2.6425	2.4346	0.0315	0.0728	-4.2505
Gm42890	-0.9276	-0.6618	-2.4336	0.0316	0.0729	-3.6358
Aldh3b3	1.4881	-1.015	2.4332	0.0316	0.073	-3.6203
Mterf4	0.2643	4.1824	2.4325	0.0317	0.073	-4.5564
Polb	0.345	4.6507	2.4323	0.0317	0.073	-4.6374
Zfp964	1.0479	1.4367	2.4323	0.0317	0.073	-4.0046
A430035B10Rik	1.4185	-1.8792	2.4309	0.0318	0.0732	-3.5206
Gm37855	1.0989	-0.6317	2.4298	0.0318	0.0734	-3.6648
Gm15787	1.1483	-0.7547	2.4297	0.0318	0.0734	-3.6058
Rbm41	-0.2859	4.0341	-2.4295	0.0318	0.0734	-4.5143
Tcf12	0.2507	7.4674	2.4295	0.0318	0.0734	-4.8236
Gm16278	1.022	-2.0818	2.4293	0.0318	0.0734	-3.5361
Cap1	-0.2928	8.5688	-2.4286	0.0319	0.0735	-4.8408
Pum1	0.2459	6.7477	2.4286	0.0319	0.0735	-4.8101
Ddx55	-0.3526	4.1043	-2.4285	0.0319	0.0735	-4.5289
Gm10130	-0.5612	2.1531	-2.4282	0.0319	0.0735	-4.1347
Dhrs7b	-0.3209	4.8641	-2.4279	0.0319	0.0735	-4.6596
Lrfn4	0.3842	5.5268	2.4276	0.0319	0.0736	-4.7465
Serp1	-0.3589	8.0381	-2.4275	0.0319	0.0736	-4.8343
Ppp2cb	-0.2547	6.8362	-2.4273	0.032	0.0736	-4.8137
Timmdc1	-0.2508	4.7553	-2.4273	0.032	0.0736	-4.6515
Usp48	-0.2085	6.159	-2.4271	0.032	0.0736	-4.7906
Clk2	-0.3289	5.1104	-2.4265	0.032	0.0736	-4.6954
Sil1	0.5301	5.1143	2.4265	0.032	0.0736	-4.71
5930420M18Rik	-0.8084	0.254	-2.4264	0.032	0.0736	-3.7963
Orai1	0.4219	2.6584	2.4257	0.0321	0.0737	-4.2795
Nqo1	-0.8326	5.4905	-2.4255	0.0321	0.0737	-4.7233
Pde1b	2.0625	-1.2228	2.4254	0.0321	0.0737	-3.5553
Ass1	1.4472	1.6894	2.425	0.0321	0.0738	-4.0005
1810059H22Rik	0.7772	0.2436	2.4249	0.0321	0.0738	-3.7923
Alg12	0.5526	3.628	2.4246	0.0321	0.0738	-4.4624
Gm43797	2.1927	-2.8472	2.4242	0.0321	0.0739	-3.4421
4933411K16Rik	1.6282	-2.1268	2.4229	0.0322	0.074	-3.5635
Adh1	2.2894	-2.8825	2.4223	0.0323	0.0741	-3.4341
Gm49356	-1.5112	-2.8994	-2.4222	0.0323	0.0741	-3.4504

Prcp	0.4994	4.6401	2.4219	0.0323	0.0741	-4.6447
Tirap	-0.359	5.1264	-2.4212	0.0323	0.0742	-4.6933
Zfp451	-0.2313	6.228	-2.421	0.0323	0.0742	-4.7999
Hs3st3b1	1.4682	3.7255	2.4201	0.0324	0.0743	-4.4407
Trim54	-0.5222	3.2559	-2.42	0.0324	0.0744	-4.395
Git2	0.3238	5.8456	2.4199	0.0324	0.0744	-4.7844
Gtf2h1	-0.3285	6.8424	-2.4197	0.0324	0.0744	-4.8265
Nutf2-ps1	-1.2324	-1.4009	-2.4193	0.0324	0.0744	-3.5611
Orai2	-0.3612	3.4753	-2.4192	0.0324	0.0744	-4.4272
Tmem38b	0.414	4.0017	2.4175	0.0325	0.0747	-4.5321
Mfsd14b	-0.2936	6.0633	-2.417	0.0326	0.0747	-4.7981
Rps6kb2	0.4252	3.7687	2.4165	0.0326	0.0748	-4.5107
Disp2	-2.5913	1.6077	-2.4159	0.0326	0.0748	-3.8628
Nbdy	0.3544	4.3656	2.4159	0.0326	0.0748	-4.6102
Tpcn2	0.5693	3.0378	2.4159	0.0326	0.0748	-4.3601
Fstl1	3.3552	0.6215	2.4152	0.0327	0.0749	-3.7054
Gapdh	-0.4647	10.5275	-2.415	0.0327	0.0749	-4.8767
Stk25	0.2722	6.2911	2.4139	0.0328	0.0751	-4.822
Cog1	0.2456	5.5079	2.4138	0.0328	0.0751	-4.765
Tmem189	-0.2847	5.4726	-2.4137	0.0328	0.0751	-4.7611
Ccm2	-0.2216	6.05	-2.4136	0.0328	0.0751	-4.8059
Oasl2	1.943	2.1205	2.4136	0.0328	0.0751	-4.132
Gm6035	-1.574	-2.851	-2.4135	0.0328	0.0751	-3.481
Spata2	0.3919	5.1717	2.413	0.0328	0.0751	-4.7287
Nabp1	-0.602	5.3135	-2.4128	0.0328	0.0751	-4.7314
Aven	-0.3424	4.7516	-2.4128	0.0328	0.0751	-4.6599
Mir22hg	0.6012	2.5539	2.4125	0.0328	0.0752	-4.2634
Etfb	0.4648	3.6068	2.4121	0.0329	0.0752	-4.4782
Ddr2	0.3758	8.3153	2.4117	0.0329	0.0752	-4.8681
Arid5a	-0.5021	4.3233	-2.4116	0.0329	0.0753	-4.5871
Gm37170	0.884	-0.1081	2.4113	0.0329	0.0753	-3.8058
Egfr	1.7311	0.6799	2.4107	0.0329	0.0754	-3.8955
Awat2	-2.1341	-1.7652	-2.4104	0.033	0.0754	-3.4772
Popdc2	2.0534	-3.2827	2.4102	0.033	0.0754	-3.4518
Ccdc151	1.3851	-2.1856	2.4096	0.033	0.0755	-3.5335
Dapp1	0.5635	3.5707	2.4085	0.0331	0.0756	-4.4918
Pmpcb	-0.2314	6.7588	-2.4084	0.0331	0.0756	-4.8461
Cfap97	0.2889	5.2975	2.4082	0.0331	0.0756	-4.7538
Gm37986	-1.7864	-2.2987	-2.4081	0.0331	0.0756	-3.5195
Gm5873	-0.8875	-1.5302	-2.4081	0.0331	0.0756	-3.628
Zbed5	0.5693	2.1678	2.4081	0.0331	0.0756	-4.1925
Necap2	0.2488	5.3692	2.4076	0.0331	0.0757	-4.7711
Tmem160	0.4223	4.4347	2.4071	0.0332	0.0757	-4.6462
Trp63	2.6129	-1.1154	2.4071	0.0332	0.0757	-3.58
Med10	-0.4167	5.8458	-2.4071	0.0332	0.0757	-4.8023
Llph-ps2	-1.2373	-0.8639	-2.4069	0.0332	0.0757	-3.6254
Wscd2	2.4587	-3.6419	2.4065	0.0332	0.0758	-3.4498
Gapvd1	-0.2359	7.1644	-2.4063	0.0332	0.0758	-4.8572
Tnfsf10	2.694	-2.5003	2.4059	0.0332	0.0758	-3.4622
Zfp820	0.5611	1.6375	2.4055	0.0333	0.0759	-4.0842
Rpl19-ps9	-1.6464	-2.5596	-2.4054	0.0333	0.0759	-3.4949
Dennd2d	1.7306	-2.8431	2.4054	0.0333	0.0759	-3.4643

Itgb2	-1.5014	1.8588	-2.4054	0.0333	0.0759	-4.0306
Bbs7	0.4745	4.4131	2.4043	0.0333	0.076	-4.6543
Chd4	0.2156	9.3074	2.404	0.0333	0.0761	-4.8904
Gm28404	-2.1487	-3.1788	-2.4036	0.0334	0.0761	-3.4676
Chn1os3	-1.8208	-2.4613	-2.4033	0.0334	0.0761	-3.4807
Islr	4.9524	-1.9892	2.403	0.0334	0.0762	-3.4468
Mmp1b	-3.4031	-1.8635	-2.4027	0.0334	0.0762	-3.4597
Gm11258	-1.7354	-1.9531	-2.4024	0.0334	0.0762	-3.5372
Nid1	-0.6044	7.6872	-2.4023	0.0334	0.0762	-4.8719
B230369F24Rik	1.0369	-0.5879	2.4019	0.0335	0.0763	-3.6908
Cnot7	-0.3167	6.6269	-2.4013	0.0335	0.0763	-4.8525
Dnajc4	0.4687	2.7723	2.4009	0.0335	0.0764	-4.336
Scamp5	0.7588	3.0767	2.4007	0.0335	0.0764	-4.3876
Kctd3	-0.2282	5.7198	-2.4002	0.0336	0.0765	-4.8066
Tmem221	1.79	-1.2331	2.3999	0.0336	0.0765	-3.5909
4933408J17Rik	-1.6034	-1.7687	-2.3993	0.0336	0.0766	-3.5615
Atxn1l	0.3402	6.2024	2.3986	0.0337	0.0767	-4.8434
Nek8	0.5245	2.7373	2.3985	0.0337	0.0767	-4.3305
Trpt1	0.5603	1.5456	2.3983	0.0337	0.0767	-4.0975
Itga7	1.0229	2.6609	2.3982	0.0337	0.0767	-4.285
Pacsin3	0.547	4.0965	2.3982	0.0337	0.0767	-4.6037
Gm20707	1.1191	-0.2763	2.3981	0.0337	0.0767	-3.7887
Gm42986	-1.4249	-1.4704	-2.3974	0.0337	0.0768	-3.5511
Arc	1.2507	3.319	2.397	0.0338	0.0768	-4.406
Dr1	-0.4257	6.045	-2.397	0.0338	0.0768	-4.8259
Spag6	1.9526	-1.8721	2.3967	0.0338	0.0768	-3.5613
Dbt	-0.3093	5.2922	-2.3961	0.0338	0.0769	-4.77
Adamts1	1.2727	5.9128	2.396	0.0338	0.0769	-4.8245
Ribc1	-0.4288	2.1343	-2.3958	0.0338	0.0769	-4.2254
Gm38079	1.7525	-2.5472	2.3954	0.0339	0.077	-3.5235
Ypel4	1.3923	-0.1317	2.395	0.0339	0.077	-3.7875
Fgfrl1	0.6999	5.0688	2.3949	0.0339	0.077	-4.7636
Pon2	0.637	5.1695	2.3942	0.0339	0.0771	-4.7631
Arhgap39	0.2759	4.434	2.3939	0.034	0.0771	-4.6607
Gid8	0.3605	5.2946	2.3939	0.034	0.0771	-4.7786
Arhgef28	0.5991	3.1733	2.3938	0.034	0.0771	-4.4334
B230118H07Rik	0.3728	4.0446	2.3936	0.034	0.0772	-4.6051
Gm20744	-2.079	-2.3956	-2.3935	0.034	0.0772	-3.4873
Gm4134	-1.2379	-1.4371	-2.3934	0.034	0.0772	-3.5689
Cd2ap	-0.3697	6.9411	-2.3934	0.034	0.0772	-4.8737
Arhgdib	-1.3985	3.0371	-2.3925	0.0341	0.0773	-4.3067
Acyp2	0.6072	1.8796	2.3921	0.0341	0.0773	-4.1797
Gm43863	1.4591	-2.6527	2.392	0.0341	0.0773	-3.5142
Ubap2l	0.244	8.6545	2.3918	0.0341	0.0773	-4.9064
Lmx1a	-3.2644	-0.1777	-2.3918	0.0341	0.0773	-3.551
Gm31105	2.0755	-2.2546	2.3909	0.0342	0.0774	-3.5032
Rgs10	-0.6574	0.914	-2.3909	0.0342	0.0774	-3.9822
Lgals3bp	1.5344	4.3973	2.3906	0.0342	0.0775	-4.6186
Hsp90ab1	-0.2231	11.729	-2.3903	0.0342	0.0775	-4.9235
Ilk	0.2396	7.2263	2.3903	0.0342	0.0775	-4.8887
Rdh10	0.8833	4.1223	2.3899	0.0342	0.0775	-4.5882
Fbxo45	-0.2652	5.1895	-2.3889	0.0343	0.0777	-4.7649

Nfat5	-0.3729	7.765	-2.3885	0.0343	0.0777	-4.8974
Naip1	-4.4516	-2.4895	-2.3883	0.0343	0.0777	-3.4709
Arpin	-0.2695	5.3076	-2.3878	0.0343	0.0778	-4.7881
Ogfod2	0.4298	3.809	2.3875	0.0344	0.0778	-4.5728
Gm7384	-1.2819	-2.0136	-2.3863	0.0344	0.078	-3.577
Psmb5	-0.3582	6.1871	-2.3851	0.0345	0.0781	-4.8649
Msmo1	-0.5672	7.6607	-2.3848	0.0345	0.0782	-4.9031
Hdhd3	0.6027	2.0572	2.3843	0.0346	0.0782	-4.1915
Ltn1	-0.3111	6.6812	-2.3843	0.0346	0.0782	-4.8834
Pth2r	1.6512	-3.2844	2.3843	0.0346	0.0782	-3.5003
Patz1	0.5755	5.44	2.384	0.0346	0.0782	-4.8076
Fdxacb1	-0.4523	2.7794	-2.3839	0.0346	0.0782	-4.3475
Tbkbp1	-1.0632	3.2994	-2.3839	0.0346	0.0782	-4.387
CAAA01147332	0.4529	5.3357	2.3838	0.0346	0.0782	-4.8053
Plekhg3	-0.3038	5.8231	-2.3838	0.0346	0.0782	-4.8432
Capn1	-0.4327	6.9494	-2.3833	0.0346	0.0783	-4.8944
Pard3	0.3463	6.771	2.383	0.0346	0.0783	-4.8912
Lsm10	0.4573	3.6986	2.3827	0.0347	0.0784	-4.5605
Klhl28	0.5189	3.4673	2.3824	0.0347	0.0784	-4.4871
2700046G09Rik	0.9145	-0.6419	2.3824	0.0347	0.0784	-3.7783
Dnm1	-0.4202	6.5115	-2.3822	0.0347	0.0784	-4.8808
Hmgb1-ps3	-0.7704	-0.2016	-2.3822	0.0347	0.0784	-3.7674
Raet1d	-1.1118	-0.2868	-2.3819	0.0347	0.0784	-3.7858
Gm8705	-1.2372	-2.0095	-2.3815	0.0347	0.0785	-3.592
Gm7901	0.6729	1.4262	2.3811	0.0348	0.0785	-4.1045
G6pc3	-0.3386	4.9081	-2.381	0.0348	0.0785	-4.7438
D230025D16Rik	0.2764	4.6554	2.3808	0.0348	0.0785	-4.7135
Mcu	-0.3961	4.6938	-2.3805	0.0348	0.0786	-4.7097
Kdelc2	0.4043	5.6171	2.3804	0.0348	0.0786	-4.8349
Asb5	1.7907	-2.8269	2.38	0.0348	0.0786	-3.4887
Fcgrt	3.0428	0.8066	2.3797	0.0349	0.0787	-3.7761
Gm37475	1.7083	-3.0051	2.3791	0.0349	0.0787	-3.5205
Krtcap2	-0.3483	6.3144	-2.3787	0.0349	0.0788	-4.881
Gm12020	-2.0304	-2.2713	-2.3786	0.0349	0.0788	-3.546
B230398E01Rik	1.9469	-2.3762	2.3778	0.035	0.0789	-3.5582
Golga4	0.3017	6.6965	2.3775	0.035	0.0789	-4.8997
Slc39a9	0.2459	5.9058	2.3771	0.035	0.079	-4.8614
Frrs1	0.4357	6.3933	2.377	0.035	0.079	-4.8891
Asb7	0.2759	4.2301	2.3766	0.0351	0.079	-4.6582
Tbc1d10a	-0.2672	5.27	-2.3757	0.0351	0.0791	-4.8047
Fads1	0.3101	7.7235	2.3746	0.0352	0.0793	-4.9248
Sik3	-0.2363	6.939	-2.3741	0.0352	0.0793	-4.9095
1700109H08Rik	-0.8801	0.2779	-2.374	0.0352	0.0793	-3.8785
4933433G15Rik	-1.3184	-2.4944	-2.3739	0.0352	0.0794	-3.5232
Minpp1	-0.3556	6.0792	-2.3738	0.0352	0.0794	-4.8751
Rpl14-ps1	-0.3822	3.0486	-2.3737	0.0352	0.0794	-4.4213
Lrrc71	1.1635	-2.4743	2.3735	0.0353	0.0794	-3.5762
Crk	-0.2372	7.4469	-2.373	0.0353	0.0794	-4.9204
AF357399	-1.739	-1.8375	-2.3728	0.0353	0.0795	-3.5815
Hsd17b14	2.2366	-3.3115	2.3727	0.0353	0.0795	-3.4995
Gm4793	1.416	-2.4944	2.3723	0.0353	0.0795	-3.5539
Jade2	-0.6445	6.4828	-2.3719	0.0354	0.0796	-4.8898

Tmem25	1.6419	-1.23	2.3717	0.0354	0.0796	-3.7139
Uqcrh	0.3059	6.8421	2.3714	0.0354	0.0796	-4.9144
Gm10800	1.6864	-3.2096	2.3708	0.0354	0.0797	-3.5202
Gm25117	-1.7004	-2.8883	-2.3708	0.0354	0.0797	-3.5192
Mettl5	-0.4815	3.1533	-2.3705	0.0354	0.0797	-4.4331
Cntnap5a	1.6732	-1.7002	2.3702	0.0355	0.0797	-3.6197
Lrriq3	1.1385	0.2179	2.3701	0.0355	0.0797	-3.9188
Dcaf10	0.3601	5.1232	2.37	0.0355	0.0797	-4.7995
Trim32	0.2364	5.1868	2.3691	0.0355	0.0799	-4.8088
Gcdh	0.3649	4.4558	2.3687	0.0356	0.0799	-4.7088
Mettl25	-0.3745	2.9785	-2.3683	0.0356	0.08	-4.4151
Wfs1	0.4405	4.6522	2.3683	0.0356	0.08	-4.7478
Sema3c	1.8702	3.64	2.3681	0.0356	0.08	-4.4985
Bdh2	3.5591	-0.1474	2.3681	0.0356	0.08	-3.7074
4930478K11Rik	-1.7129	-1.9823	-2.3668	0.0357	0.0801	-3.5777
Abl2	-0.2855	7.4511	-2.3664	0.0357	0.0802	-4.9316
Gm6477	-0.52	2.6891	-2.3662	0.0357	0.0802	-4.3647
Ppp3ca	0.4649	8.4967	2.3641	0.0359	0.0805	-4.9522
Gm15784	-1.2837	-1.6223	-2.3633	0.0359	0.0806	-3.5989
Gm7444	1.2461	-2.317	2.3632	0.0359	0.0806	-3.6129
Cdc42bpa	0.2658	6.7617	2.3628	0.0359	0.0807	-4.9255
Romo1	0.2863	5.1227	2.3627	0.0359	0.0807	-4.8207
Polr2f	-0.376	6.4561	-2.3623	0.036	0.0807	-4.9152
Usp4	0.2007	7.074	2.3619	0.036	0.0808	-4.935
Bcl2l12	-0.4412	3.0241	-2.3616	0.036	0.0808	-4.4342
Nsmce1	-0.3213	5.184	-2.3616	0.036	0.0808	-4.8171
Med9	-0.3242	4.7408	-2.3612	0.036	0.0808	-4.7562
Flt3	-1.6205	-1.7177	-2.3608	0.0361	0.0809	-3.6081
Sft2d3	0.4943	1.6553	2.3604	0.0361	0.0809	-4.1869
Gm10275	-0.361	2.9074	-2.3602	0.0361	0.081	-4.4193
Pnpla7	0.4909	3.8152	2.36	0.0361	0.081	-4.6195
Gm12017	-1.2014	-2.6078	-2.3599	0.0361	0.081	-3.5867
Arfgef1	-0.2519	7.4125	-2.3598	0.0361	0.081	-4.9428
Nexn	2.4557	-0.7447	2.3597	0.0361	0.081	-3.6657
Gm12504	-0.6921	-0.3062	-2.3596	0.0361	0.081	-3.8163
Gm7292	0.3918	3.6177	2.3596	0.0362	0.081	-4.5764
Gm5619	1.0176	-1.1279	2.3591	0.0362	0.081	-3.711
Hhex	-1.4339	-1.5148	-2.359	0.0362	0.081	-3.6168
Zfhx2	0.9562	3.1247	2.3589	0.0362	0.081	-4.4372
Gm49490	1.7445	-2.4165	2.3583	0.0362	0.0811	-3.6107
Cisd1	-0.3812	5.3247	-2.3582	0.0362	0.0811	-4.8392
Cldn15	1.414	-1.992	2.3582	0.0362	0.0811	-3.5834
Gm29054	-1.2937	-2.6405	-2.3582	0.0362	0.0811	-3.5975
Arhgap21	0.2511	6.6035	2.3576	0.0363	0.0812	-4.9297
Gm12258	0.7148	1.1968	2.3573	0.0363	0.0812	-4.0692
Vwa7	1.1149	-1.6041	2.3572	0.0363	0.0812	-3.6622
Fkbp8	0.1822	7.4245	2.3571	0.0363	0.0812	-4.9498
Plac1	-1.6234	-0.1085	-2.3571	0.0363	0.0812	-3.7899
Csnk1d	0.1964	7.9931	2.3571	0.0363	0.0812	-4.9585
Pdcd2	-0.2663	5.0741	-2.357	0.0363	0.0812	-4.8126
Sv2c	-1.6629	-2.8349	-2.3568	0.0363	0.0812	-3.5396
AC166779.2	-0.7993	0.3025	-2.3564	0.0364	0.0813	-3.95

Rras2	-0.4073	5.7159	-2.3561	0.0364	0.0813	-4.8767
Ppl	0.9069	6.2182	2.356	0.0364	0.0813	-4.9084
Ttc21b	-0.2817	5.8385	-2.3558	0.0364	0.0813	-4.8953
Tsn	-0.2444	7.419	-2.3557	0.0364	0.0813	-4.9499
Gm15495	-1.7581	-3.9608	-2.3553	0.0364	0.0814	-3.5182
Myh14	2.3934	-1.8525	2.3547	0.0365	0.0815	-3.6098
Calhm5	2.7578	-3.7897	2.3545	0.0365	0.0815	-3.5242
Rlim	-0.3163	7.7618	-2.3543	0.0365	0.0815	-4.9579
BC004004	0.3537	5.443	2.3538	0.0365	0.0816	-4.8693
Sox12	-0.4136	5.2953	-2.3525	0.0366	0.0817	-4.8389
March5	0.2975	6.6197	2.3522	0.0366	0.0818	-4.9405
1700113A16Rik	1.1146	0.095	2.3521	0.0366	0.0818	-3.9163
Cacng4	-2.2472	-2.2868	-2.3518	0.0367	0.0818	-3.5577
Plcd4	1.9461	-3.2785	2.351	0.0367	0.0819	-3.5274
Apol10b	2.8761	-0.9018	2.3504	0.0368	0.082	-3.7016
Cox7b	-0.3041	7.6204	-2.3501	0.0368	0.082	-4.9634
Cnot11	-0.2163	5.698	-2.3498	0.0368	0.0821	-4.8897
Fundc1	0.2704	5.4088	2.3495	0.0368	0.0821	-4.8719
Fam114a1	0.5147	5.2851	2.3489	0.0369	0.0822	-4.8588
Sbsn	1.5609	0.9086	2.3487	0.0369	0.0822	-3.9936
Ep400	0.369	7.2304	2.3483	0.0369	0.0823	-4.9597
Ckb	0.959	2.1495	2.3483	0.0369	0.0823	-4.288
Kctd2	0.3368	4.2514	2.3482	0.0369	0.0823	-4.7059
Ago2	-0.3176	7.6491	-2.3478	0.0369	0.0823	-4.967
Zfp26	0.3301	4.3713	2.347	0.037	0.0824	-4.7214
Taco1os	1.2823	-1.5008	2.3468	0.037	0.0824	-3.6999
Gm15536	-1.041	-1.1577	-2.3468	0.037	0.0824	-3.6958
Gstm2-ps1	1.0572	-1.5757	2.3458	0.0371	0.0826	-3.6791
Gigyf2	0.2564	6.9311	2.3454	0.0371	0.0826	-4.9597
Polr2i	-0.2838	4.0916	-2.3453	0.0371	0.0826	-4.6776
Mosmo	-0.3112	5.2821	-2.345	0.0371	0.0826	-4.8479
Mtmr10	-0.4202	4.0484	-2.3446	0.0371	0.0827	-4.6629
Atox1	0.3462	6.5263	2.3445	0.0372	0.0827	-4.9536
Dpp9	-0.2395	6.5337	-2.3444	0.0372	0.0827	-4.9504
Mrps31	-0.3178	4.7477	-2.3444	0.0372	0.0827	-4.7955
Gm12216	2.7666	-1.3861	2.3441	0.0372	0.0827	-3.6508
Cep57	-0.5529	6.2976	-2.3438	0.0372	0.0828	-4.9366
Bbs2	0.3301	3.8445	2.3436	0.0372	0.0828	-4.6475
Dlgap2	2.6414	-3.4728	2.3435	0.0372	0.0828	-3.5399
Atg7	0.2743	4.5544	2.3431	0.0373	0.0828	-4.7735
Becn1	0.2254	6.5649	2.3431	0.0373	0.0828	-4.9565
Baz1b	-0.2777	8.1373	-2.3429	0.0373	0.0828	-4.983
Agap2	1.3657	-2.6757	2.3427	0.0373	0.0829	-3.6065
4732491K20Rik	0.6712	-0.4436	2.3425	0.0373	0.0829	-3.8768
Sytl2	3.5041	-1.5749	2.3424	0.0373	0.0829	-3.5944
Ankhd1	0.4454	6.6388	2.3419	0.0373	0.0829	-4.959
Gm5540	1.2585	-1.9086	2.3413	0.0374	0.083	-3.689
Gm15697	1.016	-1.5642	2.3399	0.0375	0.0832	-3.7243
Klhl9	0.2385	6.4397	2.3392	0.0375	0.0833	-4.9566
Gm8210	-0.2922	4.9145	-2.3391	0.0375	0.0833	-4.8232
Ephb4	0.4577	6.9606	2.3385	0.0376	0.0834	-4.9736
Serpina3i	4.9378	-0.8528	2.338	0.0376	0.0835	-3.5929

Ptprg	-0.3427	5.7782	-2.3372	0.0376	0.0836	-4.9141
Gtpbp3	-0.348	5.4862	-2.3369	0.0377	0.0836	-4.8909
Psma4	-0.2984	7.628	-2.3369	0.0377	0.0836	-4.9868
Trit1	-0.4333	3.8213	-2.3367	0.0377	0.0836	-4.6383
Esrp2	1.9996	-1.0089	2.3354	0.0378	0.0838	-3.7172
Sytl1	1.4056	-1.2831	2.3347	0.0378	0.0839	-3.7409
Pgam1-ps2	-0.6901	0.0192	-2.3345	0.0378	0.0839	-3.9346
Tmem131	0.3755	7.4184	2.3342	0.0379	0.084	-4.9883
Elobl	4.0299	-2.892	2.3342	0.0379	0.084	-3.5565
L1cam	-1.1095	-0.7197	-2.334	0.0379	0.084	-3.7928
Tmem79	-0.4884	1.8979	-2.3339	0.0379	0.084	-4.2482
Tm9sf4	0.1982	6.518	2.3338	0.0379	0.084	-4.9686
Gabpb1	-0.3224	5.1928	-2.3336	0.0379	0.084	-4.8672
Gm26695	1.7797	-2.7837	2.3335	0.0379	0.084	-3.5751
Ufsp2	-0.33	5.6536	-2.3335	0.0379	0.084	-4.9201
Ergic2	-0.2247	6.7972	-2.3334	0.0379	0.084	-4.9757
Csnk1g3	-0.2895	6.5242	-2.3325	0.038	0.0841	-4.9689
Selenop	3.3964	0.1335	2.3323	0.038	0.0841	-3.8275
Hgs	0.2429	6.664	2.3319	0.038	0.0842	-4.9782
Rhd	1.1878	0.2061	2.3319	0.038	0.0842	-3.9675
Sppl2a	0.2963	6.5559	2.3316	0.038	0.0842	-4.9741
Serpina3f	2.7149	-3.5678	2.3313	0.0381	0.0843	-3.5572
Camk2n2	-0.8154	2.2801	-2.3311	0.0381	0.0843	-4.3072
Gm17430	1.7949	-3.2343	2.331	0.0381	0.0843	-3.5729
Gm22027	-1.7951	-2.3831	-2.3305	0.0381	0.0843	-3.5915
Hyal2	0.4865	5.5841	2.3303	0.0381	0.0844	-4.9198
B4galt5	0.512	6.8931	2.33	0.0381	0.0844	-4.9861
Tacc2	1.0778	6.3125	2.33	0.0381	0.0844	-4.968
Gm6382	-1.2569	-1.588	-2.3293	0.0382	0.0845	-3.6888
Fam210b	0.6002	4.5545	2.3288	0.0382	0.0845	-4.7994
Gm35551	1.0875	-2.485	2.3287	0.0382	0.0846	-3.6432
Castor1	-0.3532	3.0199	-2.3285	0.0382	0.0846	-4.5102
Col6a4	1.4203	-1.492	2.3283	0.0383	0.0846	-3.7311
Tnfrsf26	-1.6514	-1.1213	-2.328	0.0383	0.0846	-3.6836
Prmt2	0.5697	5.1026	2.3272	0.0383	0.0847	-4.8851
E2f2	-0.8159	3.7451	-2.3272	0.0383	0.0847	-4.5837
Ovgp1	-0.7708	1.0623	-2.3271	0.0383	0.0847	-4.0648
Adar	0.257	5.5625	2.3269	0.0384	0.0847	-4.9254
Ppil3	-0.2409	4.8293	-2.3268	0.0384	0.0848	-4.8343
Nek3	0.6192	2.9779	2.3268	0.0384	0.0848	-4.504
Rbm11	2.8954	-3.3523	2.3265	0.0384	0.0848	-3.5637
Ube2f	0.2786	5.9185	2.3263	0.0384	0.0848	-4.9566
Med20	-0.2907	4.8141	-2.325	0.0385	0.085	-4.8278
Ddx59	0.3602	3.7532	2.3247	0.0385	0.085	-4.6643
Wdr83os	-0.2635	4.8756	-2.3245	0.0385	0.0851	-4.8427
Dhx16	-0.2327	6.3146	-2.3242	0.0385	0.0851	-4.977
Cdan1	-0.3189	4.3386	-2.324	0.0386	0.0851	-4.7537
Paox	-0.6087	3.2066	-2.3236	0.0386	0.0852	-4.5467
Tmed7	-0.288	7.6406	-2.3235	0.0386	0.0852	-5.0088
Mns1	-0.6021	3.3175	-2.3233	0.0386	0.0852	-4.5581
Gm33378	1.0142	-1.5614	2.3229	0.0386	0.0852	-3.6906
Pogk	0.379	5.3706	2.3224	0.0387	0.0853	-4.9131

Ybx1-ps2	-0.9248	-0.2187	-2.322	0.0387	0.0853	-3.8923
Yme1l1	-0.3003	7.6451	-2.322	0.0387	0.0853	-5.0117
Chtop	-0.2531	7.2111	-2.3218	0.0387	0.0853	-5.0044
Tmed2	-0.2074	6.8107	-2.3214	0.0387	0.0854	-4.9974
Blzf1	-0.2863	5.1068	-2.3208	0.0388	0.0855	-4.8754
Mtfr1l	0.3267	5.8173	2.3207	0.0388	0.0855	-4.9567
Snhg9	-1.0943	-0.8975	-2.3206	0.0388	0.0855	-3.8373
Atp13a3	0.5834	7.8289	2.3202	0.0388	0.0856	-5.0199
Gm17066	-0.6746	2.076	-2.3199	0.0388	0.0856	-4.2446
Zscan25	0.3666	3.9518	2.3199	0.0389	0.0856	-4.6988
Gemin8	-0.3237	3.3906	-2.3198	0.0389	0.0856	-4.5834
Purg	0.5964	2.2799	2.3198	0.0389	0.0856	-4.3732
Gm6394	-1.3584	-1.6774	-2.3195	0.0389	0.0856	-3.7214
Mrpl23-ps1	-1.9778	-2.1082	-2.3193	0.0389	0.0856	-3.647
Zscan4-ps2	-1.9471	-3.459	-2.3191	0.0389	0.0856	-3.576
Col3a1	1.8807	7.057	2.319	0.0389	0.0856	-5.0028
Scd4	2.3545	-1.9352	2.3184	0.039	0.0857	-3.6437
Adamts12	1.3209	2.2295	2.3183	0.039	0.0857	-4.3323
Zbtb7c	3.0993	-1.082	2.318	0.039	0.0858	-3.6589
Gm31166	1.5045	-1.9722	2.3179	0.039	0.0858	-3.6886
F420014N23Rik	1.4066	-2.192	2.3177	0.039	0.0858	-3.6881
Rnf8	-0.393	3.2653	-2.3174	0.039	0.0858	-4.5443
Rcbtb2	0.3513	5.5052	2.3171	0.039	0.0859	-4.9356
Zfp3	0.5968	1.6275	2.3167	0.0391	0.0859	-4.2556
Mical3	0.3274	5.539	2.3165	0.0391	0.0859	-4.9364
Gm4787	1.412	-2.0661	2.3161	0.0391	0.086	-3.6795
Xpr1	0.2908	6.1375	2.3155	0.0392	0.0861	-4.9812
Lman1	-0.3489	8.2298	-2.3145	0.0392	0.0862	-5.034
Gm10080	1.2662	-1.1974	2.313	0.0393	0.0864	-3.7578
Pex14	-0.28	4.9792	-2.3129	0.0393	0.0864	-4.8811
Gm15163	-0.9521	0.3561	-2.3122	0.0394	0.0865	-3.991
Relch	0.2335	5.1543	2.3113	0.0395	0.0867	-4.9031
5430430B14Rik	1.0261	0.4497	2.3112	0.0395	0.0867	-4.018
Kpna6	-0.2345	6.8476	-2.3104	0.0395	0.0868	-5.0177
Mc3r	-1.8847	-3.4892	-2.3099	0.0396	0.0869	-3.5889
Malsu1	-0.3722	4.4723	-2.3096	0.0396	0.0869	-4.8077
Gpr176	1.8659	-2.2055	2.3096	0.0396	0.0869	-3.6712
Smg7	-0.2157	6.6577	-2.3091	0.0396	0.0869	-5.0135
Rgl3	0.7761	2.847	2.309	0.0396	0.087	-4.4832
Gm44171	2.4551	-3.2616	2.3083	0.0397	0.087	-3.5911
Acrbp	0.8072	0.2712	2.3071	0.0398	0.0872	-4.0318
Gm9315	-0.9726	-1.7303	-2.3068	0.0398	0.0873	-3.7416
Zfp868	0.3963	4.2915	2.3068	0.0398	0.0873	-4.7808
1700003E16Rik	0.7192	0.4272	2.3067	0.0398	0.0873	-4.058
Cdk17	-0.4621	5.1082	-2.3063	0.0398	0.0873	-4.8975
Pnpla8	0.355	5.8651	2.306	0.0398	0.0874	-4.9855
Tubgcp3	-0.268	5.9218	-2.3046	0.0399	0.0876	-4.986
Gm16436	-1.8403	-3.3158	-2.3043	0.04	0.0876	-3.5908
Cxcr2	-1.7991	-3.0769	-2.3042	0.04	0.0876	-3.5963
Xkr5	-1.7654	-1.3615	-2.3036	0.04	0.0877	-3.7536
Gm24245	0.6216	2.4235	2.3032	0.04	0.0877	-4.3808
Strn3	-0.2358	6.495	-2.3028	0.0401	0.0878	-5.0203

Wdr24	0.2999	4.6534	2.3023	0.0401	0.0879	-4.8636
Slc38a4	2.0435	-2.6248	2.3016	0.0402	0.088	-3.6533
Tspan4	-0.3942	6.6017	-2.3013	0.0402	0.088	-5.0246
2310014F06Rik	1.0196	-1.1685	2.3012	0.0402	0.088	-3.8329
Lgalsl	0.4956	4.7981	2.3005	0.0402	0.0881	-4.8846
Cbx2	0.6118	4.2877	2.3002	0.0403	0.0881	-4.7913
Islr2	1.5634	-2.5687	2.3	0.0403	0.0882	-3.6754
Gm5846	-0.9115	-0.9064	-2.2996	0.0403	0.0882	-3.8282
Gm28294	-2.0485	-3.0818	-2.2991	0.0403	0.0883	-3.6118
C230035I16Rik	0.7378	-0.3855	2.2989	0.0404	0.0883	-3.8931
Unc50	0.2516	5.0502	2.2985	0.0404	0.0883	-4.9148
Vps25	0.6126	0.6554	2.2983	0.0404	0.0884	-4.1453
Mfsd10	-0.3487	5.7158	-2.2979	0.0404	0.0884	-4.9769
Lipe	0.4955	3.5664	2.2969	0.0405	0.0886	-4.6769
Ptprf	1.2828	4.6859	2.2969	0.0405	0.0886	-4.8317
Camta1	-0.4177	2.5887	-2.2965	0.0405	0.0886	-4.4578
Elp2	-0.2758	7.3252	-2.2962	0.0405	0.0886	-5.0513
Gm4895	-1.5275	-2.7019	-2.2961	0.0406	0.0887	-3.6268
Aimp1	-0.253	6.8381	-2.296	0.0406	0.0887	-5.0429
Cry2	0.4088	3.0428	2.2959	0.0406	0.0887	-4.5624
Mtf2	-0.2915	5.8122	-2.2957	0.0406	0.0887	-4.9904
Gm12350	0.6524	-0.2195	2.2957	0.0406	0.0887	-3.9635
Gm14820	-1.7882	-2.0104	-2.2954	0.0406	0.0887	-3.6915
Vmn2r87	2.3506	-3.3491	2.295	0.0406	0.0888	-3.6101
Scrn2	0.447	3.186	2.2942	0.0407	0.0889	-4.594
Nfrkb	0.2721	5.7386	2.2935	0.0407	0.089	-4.9944
Gm10167	-0.5749	1.4058	-2.2934	0.0408	0.089	-4.2283
Ndufb1-ps	0.2501	6.041	2.2932	0.0408	0.089	-5.0215
Gm9727	-0.9531	-1.014	-2.2928	0.0408	0.0891	-3.8336
Ube2d-ps	-0.2446	4.2045	-2.2921	0.0409	0.0892	-4.7974
Rfesd	0.4037	3.0122	2.2921	0.0409	0.0892	-4.5683
Ppp6c	-0.3616	5.8174	-2.292	0.0409	0.0892	-4.9989
Cdkl5	0.8124	4.1577	2.2917	0.0409	0.0892	-4.7745
Gm49733	-2.1238	-2.7874	-2.2914	0.0409	0.0892	-3.6366
Gm4890	1.523	-0.7449	2.2911	0.0409	0.0893	-3.8531
Cmtm8	1.1512	-0.5843	2.2906	0.041	0.0893	-3.9101
Gm12276	1.2658	-1.9529	2.2905	0.041	0.0893	-3.7158
Rasl11a	-1.0996	0.1706	-2.2903	0.041	0.0894	-3.951
Gm38394	0.5599	3.122	2.2902	0.041	0.0894	-4.5562
Eda2r	0.5498	2.7745	2.2887	0.0411	0.0896	-4.4953
Qrfp	-2.0982	-3.7324	-2.2883	0.0411	0.0897	-3.6157
Rbm12b2	-0.3884	2.7669	-2.2881	0.0412	0.0897	-4.5196
Adgrb2	-0.7047	0.6305	-2.2875	0.0412	0.0898	-4.0935
Gm9824	-1.0199	-0.8584	-2.2874	0.0412	0.0898	-3.8757
Zfp322a	0.295	4.7652	2.2864	0.0413	0.0899	-4.895
Ccdc125	1.6575	-2.8922	2.2863	0.0413	0.0899	-3.6755
Vps16	-0.2683	5.255	-2.2862	0.0413	0.0899	-4.9536
Lrrc8d	0.226	6.3638	2.2861	0.0413	0.0899	-5.045
Dpysl3	1.2903	5.433	2.2856	0.0413	0.09	-4.9701
Abhd16a	0.3443	5.0262	2.2853	0.0414	0.09	-4.933
Rnf2	-0.2989	6.3652	-2.2853	0.0414	0.09	-5.0426
Mlph	1.4007	-0.2176	2.2851	0.0414	0.09	-3.917

Nol4	1.1636	0.9934	2.2851	0.0414	0.09	-4.181
Snapc5	0.6583	3.1125	2.285	0.0414	0.09	-4.6016
Cep78	-0.4657	3.9609	-2.2848	0.0414	0.0901	-4.7517
Gatc	-0.3415	4.6642	-2.2847	0.0414	0.0901	-4.8834
Ik	0.2446	7.3131	2.2835	0.0415	0.0902	-5.0753
Gm4707	-1.0175	-2.1121	-2.2833	0.0415	0.0903	-3.699
Cyp26b1	2.6027	2.4631	2.2832	0.0415	0.0903	-4.3148
Gm44075	1.4063	-2.5064	2.283	0.0415	0.0903	-3.7038
Tmem179	-1.8019	-3.1688	-2.283	0.0415	0.0903	-3.6341
Gm5865	1.1693	-1.7707	2.2825	0.0416	0.0904	-3.7076
Angptl4	5.235	1.85	2.2822	0.0416	0.0904	-3.9953
Lap3	-0.3851	7.7284	-2.2818	0.0416	0.0904	-5.0827
Lrif1	0.4199	4.44	2.2818	0.0416	0.0904	-4.8495
Tlr7	-1.0955	1.564	-2.2807	0.0417	0.0906	-4.2267
Gm4705	-0.8847	-1.8009	-2.2802	0.0417	0.0907	-3.7893
Gm42659	-1.2738	-1.1171	-2.28	0.0418	0.0907	-3.7825
Eif4a-ps4	-0.3982	3.4229	-2.2796	0.0418	0.0907	-4.664
Tead4	-0.3997	6.1637	-2.2795	0.0418	0.0908	-5.0455
Zfp597	0.3435	3.8184	2.2794	0.0418	0.0908	-4.7349
Xbp1	-0.3854	6.7236	-2.2789	0.0418	0.0908	-5.0682
Arfgap3	0.4905	5.8499	2.2784	0.0419	0.0909	-5.0332
Nol4l	-0.6992	4.1465	-2.2773	0.042	0.0911	-4.7836
Tmem88b	1.493	-2.5023	2.277	0.042	0.0911	-3.6885
Snora41	-0.9407	-1.2367	-2.2769	0.042	0.0911	-3.8091
Fam13c	1.3618	0.4807	2.2769	0.042	0.0911	-4.1029
Gm13502	-1.3002	-0.7002	-2.2768	0.042	0.0911	-3.8709
Strip2	1.3392	-2.0259	2.2765	0.042	0.0911	-3.7811
Cul5	-0.2738	6.6422	-2.2765	0.042	0.0911	-5.0694
Cep89	-0.3055	4.233	-2.2753	0.0421	0.0913	-4.8182
Vrk3	0.2438	4.9638	2.2753	0.0421	0.0913	-4.9509
Hnrnph3	-0.2672	4.5465	-2.2752	0.0421	0.0913	-4.8836
Slamf8	2.8598	-2.422	2.2752	0.0421	0.0913	-3.6665
Tfpi2	-1.9814	-3.8753	-2.2747	0.0422	0.0914	-3.6336
Fibp	0.2766	4.6694	2.2747	0.0422	0.0914	-4.9112
Dnajc13	0.2791	7.7086	2.2747	0.0422	0.0914	-5.096
Gtdc1	-0.346	3.6028	-2.2738	0.0422	0.0915	-4.702
Dgcr2	0.2202	6.27	2.2734	0.0423	0.0915	-5.0624
Gm44200	-1.0095	-1.7595	-2.2725	0.0423	0.0917	-3.7701
Gm38329	-1.8992	-2.2886	-2.2724	0.0423	0.0917	-3.6779
Slc25a53	0.417	2.3906	2.2724	0.0423	0.0917	-4.4789
Gm10327	-0.89	1.028	-2.2718	0.0424	0.0918	-4.2178
Gm20457	0.702	-0.0268	2.2716	0.0424	0.0918	-4.0439
Palm2	1.7017	-3.5055	2.2704	0.0425	0.092	-3.6667
Tafa2	-1.9229	-1.4997	-2.2701	0.0425	0.092	-3.7632
Snx2	0.272	6.6696	2.27	0.0425	0.092	-5.0848
Tbc1d23	0.3128	5.4186	2.2696	0.0426	0.0921	-5.0119
Eepd1	0.5697	2.8215	2.2694	0.0426	0.0921	-4.5473
Vtcn1	2.6649	-2.3015	2.2694	0.0426	0.0921	-3.6975
Gm5165	-0.5131	2.5642	-2.2693	0.0426	0.0921	-4.4836
Ppp2r5d	-0.2392	7.0393	-2.2693	0.0426	0.0921	-5.0923
Gm7224	0.661	0.1193	2.2692	0.0426	0.0921	-4.0654
Tmem269	1.6157	-2.8343	2.2684	0.0426	0.0922	-3.6833

F730043M19Rik	-0.9391	1.3959	-2.2682	0.0427	0.0922	-4.2224
Gm5297	-1.501	-2.3035	-2.2677	0.0427	0.0923	-3.7186
Aatk	-1.4945	-0.6895	-2.2676	0.0427	0.0923	-3.897
Cep44	-0.2579	4.1359	-2.2673	0.0427	0.0923	-4.8085
Pcdh19	2.5116	2.7807	2.267	0.0427	0.0924	-4.3682
Cog6	0.2783	5.6792	2.2669	0.0428	0.0924	-5.0412
Gm27252	1.8712	-3.0947	2.2669	0.0428	0.0924	-3.6495
Klf15	2.4169	-3.2381	2.2666	0.0428	0.0924	-3.6581
Stk35	-0.323	5.3088	-2.2665	0.0428	0.0924	-4.9932
Wdr33	-0.2812	6.8799	-2.266	0.0428	0.0925	-5.0944
Anapc7	-0.2031	5.7996	-2.2659	0.0428	0.0925	-5.0442
1110025M09Rik	0.9147	-0.9867	2.2651	0.0429	0.0926	-3.8566
Cacfd1	0.3345	4.945	2.2645	0.0429	0.0927	-4.9595
Phtf2	-0.4794	5.109	-2.2641	0.043	0.0927	-4.9631
Gm43268	0.96	-0.8811	2.264	0.043	0.0927	-3.8585
Scaf11	-0.3273	8.3818	-2.264	0.043	0.0927	-5.1224
Gm5913	-0.8267	0.6178	-2.2639	0.043	0.0927	-4.1313
Ifih1	1.6329	0.9866	2.2636	0.043	0.0928	-4.1882
Clec3b	2.0447	-2.6417	2.2634	0.043	0.0928	-3.728
Gpank1	-0.3656	3.5299	-2.2634	0.043	0.0928	-4.6994
Gm10241	-0.7847	-1.1437	-2.2633	0.043	0.0928	-3.901
Nupr1l	1.2566	-1.3891	2.2623	0.0431	0.093	-3.8666
Gm12166	-0.7461	0.5196	-2.2621	0.0431	0.093	-4.1408
Slit2	1.5981	3.9726	2.2617	0.0432	0.093	-4.7275
Dhdh	-1.454	-0.9494	-2.2616	0.0432	0.093	-3.8533
Fam83h	0.8137	2.8042	2.2614	0.0432	0.0931	-4.5691
Dennd4c	0.2979	5.3345	2.2614	0.0432	0.0931	-5.008
Gm49345	1.6736	-2.2879	2.2612	0.0432	0.0931	-3.7311
Mylk3	2.6074	-3.3115	2.2605	0.0433	0.0932	-3.6581
Dzip3	-0.3526	5.4967	-2.2593	0.0433	0.0934	-5.0186
Ehd3	2.7333	-2.9841	2.2588	0.0434	0.0934	-3.6604
Gatad2b	0.2311	7.2535	2.2581	0.0434	0.0935	-5.1163
Glrp1	-2.2223	-2.7332	-2.2569	0.0435	0.0937	-3.6804
Gpr137b-ps	0.4099	4.3905	2.2567	0.0436	0.0937	-4.889
Ccdc170	1.3436	-2.3915	2.2566	0.0436	0.0937	-3.7268
Rexo4	-0.3152	5.4498	-2.2566	0.0436	0.0937	-5.0244
Fxr2	-0.2437	7.1584	-2.2566	0.0436	0.0937	-5.1172
Gstz1	0.4044	4.4111	2.2562	0.0436	0.0938	-4.9012
Zbtb2	0.2915	4.2231	2.2559	0.0436	0.0938	-4.8536
Vps54	-0.2362	5.7337	-2.2557	0.0436	0.0939	-5.0547
Cmtm4	0.3637	6.4776	2.2553	0.0437	0.0939	-5.103
Gm4928	-2.1575	-2.5417	-2.2551	0.0437	0.0939	-3.683
Ddx4	2.4093	-3.7734	2.2546	0.0437	0.094	-3.6655
Rps13-ps4	-1.0867	-1.3811	-2.2541	0.0438	0.0941	-3.8453
Gm7353	0.8482	-0.3548	2.2538	0.0438	0.0941	-3.9614
Gm14325	0.6008	2.0469	2.2537	0.0438	0.0941	-4.4306
Gm45902	-0.4937	2.9116	-2.2535	0.0438	0.0942	-4.5515
Oaz3	-1.2444	-1.969	-2.253	0.0438	0.0942	-3.8344
Slc25a14	0.3303	3.2951	2.253	0.0438	0.0942	-4.7052
Acap2	-0.2994	6.8226	-2.2529	0.0438	0.0942	-5.114
Gm38067	-0.9693	-1.6206	-2.2524	0.0439	0.0943	-3.7722
Focad	-0.334	4.951	-2.2524	0.0439	0.0943	-4.9678

Phlda1	-0.6055	5.3011	-2.2521	0.0439	0.0943	-5.0014
A830035O19Rik	0.776	-0.3498	2.2513	0.044	0.0944	-4.0375
Gpatch8	0.2672	6.3175	2.251	0.044	0.0945	-5.1044
Gm12481	0.5846	0.9301	2.2503	0.0441	0.0946	-4.1951
Mbd6	0.3017	5.1512	2.25	0.0441	0.0946	-5.0105
Gm17202	1.514	-3.1971	2.2486	0.0442	0.0949	-3.712
Ddx54	-0.3146	6.8402	-2.2484	0.0442	0.0949	-5.123
Oas1b	1.2465	-0.5332	2.2483	0.0442	0.0949	-3.9436
Hrc	2.6789	-2.7397	2.2482	0.0442	0.0949	-3.6965
Gm10689	-0.9417	-0.8093	-2.2481	0.0442	0.0949	-3.965
Gadd45a	0.5646	3.706	2.2481	0.0442	0.0949	-4.8024
Sap18	-0.2877	6.1514	-2.2478	0.0443	0.0949	-5.0984
Cript	0.2651	5.3491	2.2478	0.0443	0.0949	-5.0433
Sp1	-0.3176	7.2356	-2.2474	0.0443	0.095	-5.1325
H2-Q1	1.535	-0.805	2.2471	0.0443	0.095	-3.9796
Cdk3	-0.969	0.056	-2.2464	0.0444	0.0951	-4.0351
B430305J03Rik	0.7815	0.102	2.2464	0.0444	0.0951	-4.0838
Dennd5a	0.2569	7.1078	2.2462	0.0444	0.0951	-5.1345
Mrpl46	-0.2433	4.162	-2.2453	0.0444	0.0953	-4.867
Gm15246	-0.962	-0.6487	-2.2453	0.0445	0.0953	-3.9375
Abi3	1.2105	-1.5147	2.2452	0.0445	0.0953	-3.8737
Bicc1	1.5867	3.0672	2.2443	0.0445	0.0954	-4.5645
Rbm38	-0.3332	5.4625	-2.2442	0.0445	0.0954	-5.0432
Rpl36	-0.3158	8.7043	-2.2442	0.0445	0.0954	-5.1614
Nfil3	0.2642	6.3168	2.2439	0.0446	0.0954	-5.1161
Flnc	0.4275	7.4807	2.2436	0.0446	0.0955	-5.1448
Ddx6	0.1976	8.2071	2.2434	0.0446	0.0955	-5.1563
Apopt1	0.391	3.9878	2.2432	0.0446	0.0955	-4.837
Osbpl3	0.3607	5.3254	2.2432	0.0446	0.0955	-5.0506
Rps13	-0.268	8.6501	-2.2432	0.0446	0.0955	-5.1622
Zfp771	0.3439	4.2511	2.2428	0.0447	0.0956	-4.8892
Tcam1	-1.0523	1.9863	-2.2426	0.0447	0.0956	-4.3802
Gm39822	-1.8333	-2.4922	-2.2426	0.0447	0.0956	-3.7209
Pik3cd	-0.7237	0.6814	-2.2418	0.0447	0.0957	-4.1873
Zfp608	-0.2673	6.2805	-2.2416	0.0448	0.0957	-5.1152
Mafa	1.3526	-0.448	2.2411	0.0448	0.0958	-4.0218
Gm42997	1.3927	-2.1903	2.2405	0.0448	0.0959	-3.8029
Use1	0.3488	5.286	2.2403	0.0449	0.0959	-5.0523
4930519F16Rik	-0.6343	1.9701	-2.2391	0.045	0.0961	-4.3975
Cryz	0.3816	5.5426	2.2388	0.045	0.0961	-5.08
Srd5a1	-0.4898	4.0753	-2.2388	0.045	0.0961	-4.8248
Sgms1	-0.5146	5.4694	-2.2386	0.045	0.0961	-5.0452
Hddc3	0.8821	2.1043	2.2382	0.045	0.0962	-4.4997
Pxdn	0.5601	8.2324	2.2379	0.045	0.0962	-5.1666
Sox5	-0.9043	2.7327	-2.2379	0.045	0.0962	-4.5371
Mrps14	-0.2393	5.7398	-2.2377	0.0451	0.0962	-5.0898
Gm9449	-1.2652	-2.0079	-2.2375	0.0451	0.0963	-3.805
Edrf1	-0.3559	4.6501	-2.237	0.0451	0.0963	-4.9551
Fam151b	0.8286	-0.4742	2.2369	0.0451	0.0964	-4.0196
Ifi27l2a	2.4077	-2.1902	2.2367	0.0451	0.0964	-3.7486
Foxo4	0.3826	5.7262	2.2366	0.0452	0.0964	-5.0942
Nmrk1	0.4397	2.5827	2.2365	0.0452	0.0964	-4.5858

Lst1	1.1108	-1.706	2.2364	0.0452	0.0964	-3.8497
Galnt11	0.4867	3.7408	2.2354	0.0452	0.0965	-4.8013
Gm47509	-1.337	1.0289	-2.2353	0.0453	0.0966	-4.2488
Gm7198	-1.2195	-1.7209	-2.2348	0.0453	0.0966	-3.8491
Stfa2	-2.1907	-3.6919	-2.2348	0.0453	0.0966	-3.6914
Mettl4	-0.2469	4.7426	-2.2348	0.0453	0.0966	-4.9689
Acod1	3.2757	-1.281	2.2346	0.0453	0.0966	-3.7624
Pigg	-0.3656	4.2565	-2.2341	0.0454	0.0967	-4.8913
BC034090	2.185	-1.7881	2.2337	0.0454	0.0968	-3.8318
C330011M18Ril	1.0381	-0.7808	2.2335	0.0454	0.0968	-3.9482
A730071L15Rik	1.5449	-2.658	2.2335	0.0454	0.0968	-3.7506
Galnt9	3.2753	-1.5753	2.2332	0.0454	0.0968	-3.7813
Gm13421	-0.7656	0.5938	-2.2332	0.0454	0.0968	-4.1538
Map2k5	0.2293	5.2169	2.233	0.0454	0.0968	-5.0532
Gm42600	-0.8312	-0.2967	-2.2329	0.0455	0.0968	-4.0267
1700034P13Rik	-0.9959	-2.0204	-2.2326	0.0455	0.0969	-3.8471
Mtdh	-0.2858	8.4602	-2.2325	0.0455	0.0969	-5.1773
Gm5176	1.1174	-1.3823	2.2323	0.0455	0.0969	-3.8793
Gm26648	0.7187	-0.3769	2.2316	0.0456	0.097	-4.0653
A130051J06Rik	0.892	-0.5549	2.2315	0.0456	0.097	-3.9876
BC037039	0.51	2.154	2.2311	0.0456	0.0971	-4.5035
Eci3	2.0599	-2.2443	2.231	0.0456	0.0971	-3.7811
Snord72	-1.4103	-1.6315	-2.2307	0.0456	0.0971	-3.7671
Gpkow	-0.2332	6.1709	-2.2301	0.0457	0.0972	-5.1303
Abi1	0.235	6.5874	2.23	0.0457	0.0972	-5.1482
Usp47	-0.231	7.5993	-2.2295	0.0457	0.0973	-5.1696
9030407P20Rik	0.8494	0.2191	2.2284	0.0458	0.0975	-4.1151
Ccdc12	-0.3899	4.9136	-2.2284	0.0458	0.0975	-5.0102
Smim20	0.2884	4.3794	2.2281	0.0458	0.0975	-4.9425
Dnajc24	-0.3575	3.6823	-2.228	0.0459	0.0975	-4.7986
Gm47708	-1.4814	-2.2603	-2.228	0.0459	0.0975	-3.7586
Gm6368	-0.5109	2.4199	-2.2277	0.0459	0.0975	-4.5557
AC120797.1	-2.3749	-3.2484	-2.2276	0.0459	0.0975	-3.7065
Lrfn1	-0.5882	2.0824	-2.2274	0.0459	0.0976	-4.4448
Npr1	1.2008	2.0857	2.2264	0.046	0.0977	-4.4517
Sbds	-0.1976	5.7444	-2.2262	0.046	0.0977	-5.1121
Gm43693	-1.5408	-2.8177	-2.2258	0.046	0.0978	-3.7282
Cilp2	2.6773	-1.2376	2.2255	0.0461	0.0979	-3.8175
Eif4e2	-0.2025	6.9239	-2.2252	0.0461	0.0979	-5.1648
Gm43360	-1.9516	-2.7059	-2.225	0.0461	0.0979	-3.7257
4933429H19Rik	0.9698	-1.0016	2.2248	0.0461	0.0979	-3.9648
5530601H04Rik	-0.3028	3.6413	-2.224	0.0462	0.0981	-4.7887
Mmp28	1.2051	1.6385	2.2238	0.0462	0.0981	-4.366
Spef1	-0.5128	2.2537	-2.2237	0.0462	0.0981	-4.4968
Msx3	-1.6572	-0.7868	-2.2228	0.0463	0.0982	-3.9617
Nek1	-0.3351	5.067	-2.2227	0.0463	0.0982	-5.0373
Glce	-0.2328	5.6994	-2.222	0.0464	0.0984	-5.1141
Mtif2	-0.2526	5.4596	-2.2219	0.0464	0.0984	-5.0904
Gm26558	0.8712	-0.6709	2.2214	0.0464	0.0984	-4.0126
Nlrc3	-1.2601	0.5675	-2.2212	0.0464	0.0985	-4.1502
1810008B01Rik	-0.8916	-0.9649	-2.22	0.0465	0.0987	-3.9778
Kif13a	0.5008	5.4885	2.2193	0.0466	0.0988	-5.1007

Plat	0.5493	6.1248	2.2193	0.0466	0.0988	-5.1462
Dnaaf3	0.7648	1.2313	2.2193	0.0466	0.0988	-4.3286
Syce2	-1.1704	2.2394	-2.2181	0.0467	0.0989	-4.4628
Plekhn1	0.2906	4.4331	2.2176	0.0467	0.099	-4.9665
Ecscr	1.556	2.6326	2.2171	0.0468	0.0991	-4.5664
Amd-ps4	-1.6025	-2.0015	-2.2166	0.0468	0.0992	-3.7903
Exd1	0.6495	1.8742	2.2157	0.0469	0.0993	-4.4858
Mrps15	-0.2553	5.2803	-2.2157	0.0469	0.0993	-5.0823
9330136K24Rik	-1.5111	-2.0388	-2.2156	0.0469	0.0993	-3.8326
Lss	-0.6039	6.6346	-2.2156	0.0469	0.0993	-5.1706
Spib	6.4492	-0.7466	2.2155	0.0469	0.0993	-3.7096
Gm13056	-1.4139	-0.0847	-2.2154	0.0469	0.0993	-4.0285
Ankrd13c	-0.2525	7.1987	-2.215	0.0469	0.0994	-5.1872
Hmx2	-0.8831	0.8415	-2.214	0.047	0.0995	-4.239
Gm49395	-1.0973	-2.306	-2.2137	0.047	0.0996	-3.8483
Gm43672	0.8908	1.1836	2.2131	0.0471	0.0997	-4.3477
Galnt4	0.4121	4.2523	2.2125	0.0472	0.0998	-4.9298
Fubp3	-0.1948	6.8297	-2.2123	0.0472	0.0998	-5.184
Gm5526	0.9889	-0.6301	2.2118	0.0472	0.0999	-4.0558
Lilr4b	-1.7679	-3.1496	-2.2117	0.0472	0.0999	-3.7268
Pura	-0.2936	5.9406	-2.2111	0.0473	0.1	-5.1451
Ppard	-0.3877	6.5065	-2.2109	0.0473	0.1	-5.1762
Trp53	-0.6075	2.2556	-2.2103	0.0473	0.1001	-4.538
Orm1	4.582	-0.2301	2.21	0.0474	0.1001	-3.8239
Nme5	1.0813	-1.9482	2.2098	0.0474	0.1002	-3.8592
Cldn1	4.0433	-2.017	2.2097	0.0474	0.1002	-3.7503
Hhip	-3.6194	2.0579	-2.2097	0.0474	0.1002	-4.1159
C030006K11Rik	0.4069	3.5592	2.2096	0.0474	0.1002	-4.8302
Dpagt1	-0.2561	4.9214	-2.2095	0.0474	0.1002	-5.0472
Atad3aos	1.5021	-2.3531	2.2091	0.0474	0.1002	-3.8409
Txnrd2	-0.4165	3.6198	-2.2087	0.0475	0.1003	-4.8219
Snai1	-0.7215	5.3223	-2.208	0.0475	0.1004	-5.0743
Thap1	-0.3805	4.0653	-2.2078	0.0476	0.1004	-4.8872
Gm8756	-1.3192	-1.319	-2.2077	0.0476	0.1004	-3.9352
Kdm1b	0.4046	4.6798	2.2077	0.0476	0.1004	-5.0147
Hspb7	2.0954	0.245	2.2071	0.0476	0.1005	-4.0492
Scap	0.2379	6.193	2.2069	0.0476	0.1005	-5.1717
Gucd1	0.2954	4.0696	2.2065	0.0477	0.1006	-4.9199
Fbxo2	1.1768	3.0607	2.2063	0.0477	0.1006	-4.7058
Gm6556	0.8817	-0.4182	2.2049	0.0478	0.1009	-4.0739
Fah	-0.9455	4.0048	-2.2042	0.0479	0.101	-4.8504
Nck1	0.3316	5.3656	2.2042	0.0479	0.101	-5.1138
Slc6a8	-0.2734	6.4821	-2.2041	0.0479	0.101	-5.1877
Gm6653	-1.4704	-1.6212	-2.2041	0.0479	0.101	-3.8886
Ppt2	-0.4092	4.7883	-2.2035	0.0479	0.101	-5.0347
Smyd3	0.4007	3.9764	2.2033	0.0479	0.1011	-4.9072
Sfn	1.2822	-1.8134	2.2022	0.048	0.1013	-3.8636
Slc1a7	-2.0486	-3.1886	-2.2021	0.048	0.1013	-3.7417
Ralgapa2	0.3883	5.039	2.2021	0.048	0.1013	-5.0723
Cnep1r1	-0.2725	5.5385	-2.2016	0.0481	0.1013	-5.1262
D830036C21Rik	-2.039	-3.1172	-2.2015	0.0481	0.1013	-3.7423
Mmp7	3.1159	-3.5098	2.2014	0.0481	0.1014	-3.7397

Lypd2	2.3562	-3.0911	2.2013	0.0481	0.1014	-3.7595
P4ha2	-0.8525	5.6435	-2.2011	0.0481	0.1014	-5.1229
Arhgdig	-1.0576	-1.1896	-2.2006	0.0482	0.1015	-3.8989
Mnat1	0.204	5.6272	2.2005	0.0482	0.1015	-5.1481
Wrb	-0.3775	4.7982	-2.2004	0.0482	0.1015	-5.0299
Gm9247	-0.9052	-0.2302	-2.2001	0.0482	0.1015	-4.0577
Kctd9	-0.362	5.3295	-2.1996	0.0483	0.1016	-5.1087
Slc38a10	0.2745	7.9144	2.199	0.0483	0.1017	-5.228
Ppp1r13b	0.4852	3.6162	2.1983	0.0484	0.1018	-4.8532
Morf4l1	0.1682	7.9595	2.1983	0.0484	0.1018	-5.2299
Nt5dc2	-0.3761	7.163	-2.198	0.0484	0.1018	-5.2157
Prtn3	2.0579	-0.9779	2.1965	0.0485	0.1021	-3.9702
Sec13	-0.2265	7.0833	-2.1963	0.0485	0.1021	-5.2172
Zfp661	0.4931	1.97	2.1961	0.0486	0.1021	-4.534
Tubgcp5	-0.285	4.5679	-2.1961	0.0486	0.1021	-5.015
Notch3	1.1005	0.5403	2.1961	0.0486	0.1021	-4.1884
Ptcd1	0.2629	4.9173	2.1958	0.0486	0.1022	-5.0751
Stamos	1.0953	-1.196	2.1955	0.0486	0.1022	-4.0481
Zdhhc6	-0.3267	5.7173	-2.1952	0.0486	0.1022	-5.1562
Ppp1r27	2.1681	-2.8371	2.1946	0.0487	0.1023	-3.7916
Gm37738	-1.5121	-2.5939	-2.1933	0.0488	0.1026	-3.7901
Mccc1os	1.2393	-1.5984	2.1931	0.0488	0.1026	-3.9361
Aqp1	-1.1856	3.778	-2.1929	0.0488	0.1026	-4.7575
Tbcb	0.2877	5.4895	2.1928	0.0488	0.1026	-5.1475
Wnt6	3.0069	1.6097	2.192	0.0489	0.1027	-4.2327
Gm26714	1.2604	-0.6684	2.1918	0.0489	0.1028	-4.0557
Zfp619	0.5981	0.6836	2.1917	0.0489	0.1028	-4.3225
Osbpl8	0.3988	5.5164	2.1917	0.0489	0.1028	-5.1484
Yif1a	0.3431	4.514	2.1913	0.049	0.1028	-5.0355
04-sept	1.8626	-1.9095	2.191	0.049	0.1029	-3.8359
Gm4117	0.8585	0.3291	2.1899	0.0491	0.1031	-4.2049
Ccdc114	-1.1611	0.618	-2.1898	0.0491	0.1031	-4.1952
Gm26826	1.3819	-2.1456	2.1894	0.0491	0.1031	-3.7971
Kif19a	1.3948	-1.9314	2.1894	0.0491	0.1031	-3.9033
Akap6	1.6232	-1.0054	2.1888	0.0492	0.1032	-3.9456
Gm43062	1.2667	-0.6478	2.1887	0.0492	0.1032	-4.0391
Serpina3h	4.9701	0.0341	2.1886	0.0492	0.1032	-3.9008
Gas2l1	-0.2833	6.5977	-2.1886	0.0492	0.1032	-5.2181
Gm44639	-1.1157	-1.2105	-2.1884	0.0492	0.1032	-3.9185
Fjx1	-1.3462	0.1071	-2.1882	0.0492	0.1033	-4.1113
Necab2	-2.4563	-1.9516	-2.1876	0.0493	0.1033	-3.7864
Hmgb1-ps5	-1.8182	-2.2836	-2.1876	0.0493	0.1033	-3.8279
Gm7407	-1.579	-2.8489	-2.1875	0.0493	0.1034	-3.8383
Gm37940	-1.7793	-3.1059	-2.1868	0.0494	0.1035	-3.7791
Saa3	4.5883	3.809	2.1867	0.0494	0.1035	-4.5352
Rhpn1	1.414	-0.3279	2.1866	0.0494	0.1035	-4.0518
Ddx19a	-0.277	5.8348	-2.1858	0.0495	0.1036	-5.1818
Mroh2a	2.0507	-2.5475	2.1857	0.0495	0.1036	-3.7891
Clip2	-0.3558	5.9993	-2.1855	0.0495	0.1036	-5.1938
Ush1g	1.9358	-3.3289	2.1849	0.0495	0.1037	-3.7902
Abcb8	0.2552	4.9018	2.1848	0.0495	0.1038	-5.0891
Slc16a9	2.8272	-3.4497	2.1847	0.0496	0.1038	-3.7636

Akr1c18	-3.1981	-0.2658	-2.1846	0.0496	0.1038	-3.9092
Xrra1	1.1372	-1.9265	2.1845	0.0496	0.1038	-3.9238
Gm22884	-1.7093	-1.9854	-2.1845	0.0496	0.1038	-3.8517
Lin7c	-0.4006	6.9361	-2.1834	0.0497	0.1039	-5.2332
Arg2	-1.4288	2.1929	-2.1828	0.0497	0.1041	-4.5185
Ccnk	-0.2735	6.0662	-2.1824	0.0498	0.1041	-5.2036
Oga	0.2992	7.5183	2.1818	0.0498	0.1042	-5.2504
Gm12091	-1.631	-2.6441	-2.1817	0.0498	0.1042	-3.8476
C230038L03Rik	-0.8904	-2.2372	-2.1815	0.0498	0.1042	-3.8958
Ercc2	-0.2753	4.8125	-2.1814	0.0499	0.1042	-5.0721
Prdx1	-0.3244	9.2351	-2.1814	0.0499	0.1042	-5.2717
Gm37145	-1.5679	-2.9245	-2.1811	0.0499	0.1043	-3.7923
Anks3	0.3567	4.1265	2.1809	0.0499	0.1043	-4.9733
Gm32219	1.0505	-0.7203	2.1803	0.0499	0.1044	-4.0696
Gm4045	-0.7124	0.2193	-2.1801	0.05	0.1044	-4.2034
Cfdp1	-0.2676	7.2645	-2.1801	0.05	0.1044	-5.2481
Zfpm1	-0.7684	3.453	-2.1799	0.05	0.1044	-4.8

Supplementary Table 4. Antibody dilutions

Antibody (clone)	Supplier	Catalogue Number	Dilution (application)
pMET Y1234/5 (D26) XP	Cell Signaling	3077	1:1000 (IB)
Met	R&D System	AF527	1:500 (IB)
MET (SP44)	Spring Bioscience	M3442	1:50 (IF)
pAKT S473 (D7F10) XP	Cell Signaling	9018	1:500 (IB)
AKT (40D4)	Cell Signaling	2920	1:1000 (IB)
pERK1/2 T202/Y204	Cell Signaling	9101	1:1000 (IB)
ERK1/2 (3A7)	Cell Signaling	9107	1:1000 (IB)
pFRS2 Y196	Cell Signaling	3864	1:500 (IB)
FRS2 (H-91)	Santa Cruz	sc-8318	1:500 (IB)
FGFR1 (D8E4) XP	Cell Signaling	9740	1:1000 (IB)
FGFR1 (D8E4) XP	Cell Signaling	9740	1:200 (IHC)
FGFR1 (D8E4) XP	Cell Signaling	9740	1:200 (IF)
Claudin1 (2H10D10)	Thermo Fisher	37-4900	1:1000 (IB)
Cleaved-Caspase-3 (Asp175)	Cell Signaling	9661	1:1000 (IB)
Beta-Actin (AC-15)	Sigma	A5441	1:10000 (IB)
AAD Viability Stain	eBioscience	00-6993	1:20
CD24-PE	BD Biosciences	555428	1:10 (FC)
CD44-FITC	BD Biosciences	555478	1:10 (FC)

Supplementary Table 5. Primers sequences

Gene name	Forward	Reverse
FGFR1	ATGGTTGACCGTTCTGGAAG	GGAAGTCGCTCTTCTTGGTG
FGFR2	GGATCAAGCACGTGGAAAAG	TATTCCCCAGCATCCATCTC
FGFR3	ATCGACAAGGACCGTACTGC	CCCCAGCAGGTTAATGATGT
HPRT	GCCCCAAAATGGTTAAGGTT	CAAGGGCATATCCAACAACA
RPL13	AAGGCCAAGATGCACTATCG	GAGTCCGTTGGTCTTGAGGA
ALDH1L1	CACCATGGATGCTTCAGAGA	ACTTTCCCACCATTGAGTGC
HES1	TACACCTGGAGGGGCTGTAA	CGCCTCTTCTCCATGATAGG
TWIST1	GCTCAGCTACGCCTTCTCC	CCTTCTCTGGAAACAATGACATC
CD44	TCCAGAGGCGACTAGATCC	GCGGCAGGTTACATTCAAA
MYBL2	CTGGAAGTTCTTGGCCAGTC	AACAAGGTCGGGATTCAAAA
HMGA2	AAGGCAGCAAAAACAAGAGC	AACAAGGTCGGGATTCAAAA
GDPD2	CAGAGACTCAATGCTGGAACC	TGCTGGTATGGTCTGATTCTCA
OGN	CCATCATTACCAACCAAGAAAGA	GGTGGTACAGCATCAATGTCA
