# Supplementary material for: Co-dependency for MET and FGFR1 in basal triple-negative breast cancers
Source: NPJ Breast Cancer. 2021 Mar 26;7:36. doi: 10.1038/s41523-021-00238-4 (PMC7997957; doi:10.1038/s41523-021-00238-4)
Supplement: Supplementary file 2 — Reporting Summary Checklist [file 41523_2021_238_MOESM2_ESM.pdf]

nature research

Corresponding author(s): DBPR-NPJCANCER00625

Last updated by author(s): Sep 28, 2020

Reporting Summary

Nature Research wishes to improve the reproducibility of the work that we publish. This form provides structure for consistency and transparency in reporting. For further information on Nature Research policies, see our Editorial Policies and the Editorial Policy Checklist.

Statistics

For all statistical analyses, confirm that the following items are present in the figure legend, table legend, main text, or Methods section.

n/a	Confirmed	
☐	☒	The exact sample size (n) for each experimental group/condition, given as a discrete number and unit of measurement
☐	☒	A statement on whether measurements were taken from distinct samples or whether the same sample was measured repeatedly
☐	☒	The statistical test(s) used AND whether they are one- or two-sided<br>*Only common tests should be described solely by name; describe more complex techniques in the Methods section.*
☒	☐	A description of all covariates tested
☒	☐	A description of any assumptions or corrections, such as tests of normality and adjustment for multiple comparisons
☐	☒	A full description of the statistical parameters including central tendency (e.g. means) or other basic estimates (e.g. regression coefficient) AND variation (e.g. standard deviation) or associated estimates of uncertainty (e.g. confidence intervals)
☐	☒	For null hypothesis testing, the test statistic (e.g. F, t, r) with confidence intervals, effect sizes, degrees of freedom and P value noted<br>*Give P values as exact values whenever suitable.*
☒	☐	For Bayesian analysis, information on the choice of priors and Markov chain Monte Carlo settings
☒	☐	For hierarchical and complex designs, identification of the appropriate level for tests and full reporting of outcomes
☒	☐	Estimates of effect sizes (e.g. Cohen's d, Pearson's r), indicating how they were calculated

Our web collection on statistics for biologists contains articles on many of the points above.

Software and code

Policy information about availability of computer code

Data collection	A description of the software and code has been included in the Methods. For RNA-Seq analysis, reads were mapped to human genome version hg19 using Spliced Transcripts Alignment to a Reference (STAR). Reads counts were normalized using mean-centered and log-transformed. Differentially expressed genes among groups were identified using the R packages DESeq2 and Lima.
Data analysis	Microsoft Office (Excel), Prism Graphpad, AxioVision (Carl Zeiss), Zen software (Zeiss), FlowJo, GSEA 4.0, Partek, Extreme Limiting Dilution Analysis (ELDA) online software (http://bioinf.wehi.edu.au/software/elda/), and for the gene expression-based outcome for breast cancer (GOBO) online tool (http://co.bmc.lu.se/gobo/)

For manuscripts utilizing custom algorithms or software that are central to the research but not yet described in published literature, software must be made available to editors and reviewers. We strongly encourage code deposition in a community repository (e.g. GitHub). See the Nature Research guidelines for submitting code & software for further information.

Data

Policy information about availability of data

All manuscripts must include a data availability statement. This statement should provide the following information, where applicable:

- Accession codes, unique identifiers, or web links for publicly available datasets
- A list of figures that have associated raw data
- A description of any restrictions on data availability

Accession codes will be available before publication. RNA-Sequencing analysis are presented in Figure 4. GSE XXXXXX. Deposit process ongoing

Field-specific reporting

Please select the one below that is the best fit for your research. If you are not sure, read the appropriate sections before making your selection.

☒ Life sciences ☐ Behavioural & social sciences ☐ Ecological, evolutionary & environmental sciences

For a reference copy of the document with all sections, see nature.com/documents/nr-reporting-summary-flat.pdf

Life sciences study design

All studies must disclose on these points even when the disclosure is negative.

Sample size	No sample size calculations were performed. The sample size (n) of each experiment is provided in the corresponding figure captions in the main manuscript and supplementary information files. Sample sizes were chosen to support meaningful conclusions.
Data exclusions	No data was excluded from the analyses.
Replication	All in vitro experiments were replicated successfully 3 times. In vivo tumour measurements were taken 2-3 times week and groups sizes were chosen to support meaningful conclusions.
Randomization	In the reported experiments, each group consisted of identically engineered samples. The work does not involve participant groups. Therefore, randomization was not relevant the present study.
Blinding	Methods for group allocation, data collection and all related analyses were predetermined. Furthermore, the work does not involve participant groups. Therefore, blinding was not relevant to the study.

Reporting for specific materials, systems and methods

We require information from authors about some types of materials, experimental systems and methods used in many studies. Here, indicate whether each material, system or method listed is relevant to your study. If you are not sure if a list item applies to your research, read the appropriate section before selecting a response.

Materials & experimental systems

n/a	Involved in the study	
☐	☒	Antibodies
☐	☒	Eukaryotic cell lines
☒	☐	Palaeontology and archaeology
☐	☒	Animals and other organisms
☒	☐	Human research participants
☒	☐	Clinical data
☒	☐	Dual use research of concern

Methods

n/a	Involved in the study	
☒	☐	ChIP-seq
☐	☒	Flow cytometry
☒	☐	MRI-based neuroimaging

Antibodies

Antibodies used

Antibody (clone), Supplier (Catalogue Number), Dilution (application)
pMET Y1234/5 (D26) XP, Cell Signaling (3077), 1:1000 (IB)
Met, R&D System (AF527), 1:500 (IB)
MET, In-house (148), 1:1000 (IB)
MET (SP44), Spring Bioscience (M3442), 1:50 (IF)
pAKT S473 (D7F10) XP, Cell Signaling (9018), 1:500 (IB)
AKT (40D4), Cell Signaling (2920), 1:1000 (IB)
pERK1/2 T202/Y204, Cell Signaling (9101), 1:1000 (IB)
ERK1/2 (3A7), Cell Signaling (9107), 1:1000 (IB)
pFRS2 Y196, Cell Signaling (3864), 1:500 (IB)
pFRS2 Y436, Cell Signaling (3861), 1:500 (IB)
FRS2 (H-91), Santa Cruz (sc-8318), 1:500 (IB)
FGFR1 (D8E4) XP, Cell Signaling (9740), 1:1000 (IB), 1:200 (IHC), 1:200 (IF)
Claudin1 (2H10D10), Thermo Fisher (37-4900), 1:1000 (IB)
Cleaved-Caspase-3 (Asp175), Cell Signaling (9661), 1:1000 (IB)
Beta-Actin (AC-15), Sigma (A5441), 1:10000 (IB)
Beta-tubulin, Cell Signaling (2148), 1:1000 (IB)
AAD Viability Stain, eBioscience (00-6993) 1:20 (FC)
CD24-PE, BD Biosciences (555428), 1:10 (FC)
CD44-FITC, BD Biosciences (555478), 1:10 (FC)

Validation	All the commercial antibodies were used as specified by the manufactures protocol. For the anti-Met (in house), validation has been performed in cells derived from our transgenic mouse model MMTV-Metmt;Trp53fl/+;Cre previously described in Knight et al, PNAS, 2013.

Eukaryotic cell lines

Policy information about cell lines

Cell line source(s)	The BT-20, HCC70, HCC1937, HCC1954, HCC1395, MDA-MB-468, MDA-MB-436, MDA-MB-157, MDA-MB-231, BT-549, and Hs578T cells used in this work were purchased from ATCC. The primary mouse cell lines were established by dissociation of MMTV-Metmt, Trp53fl/+;Cre, and MMTV-Metmt;Trp53fl/+;Cre mammary tumours as previously described in Knight et al, PNAS, 2013.
Authentication	The BT-20, HCC70, HCC1937, HCC1954, HCC1395, MDA-MB-468, MDA-MB-436, MDA-MB-157, MDA-MB-231, BT-549, and Hs578T cells used in this work were not autentificated as they were purchased from ATCC and used in the first few passages.
Mycoplasma contamination	The cell lines were tested for mycoplasma contamination by PCR. Results were negative for all cell lines in the time these studies were performed.
Commonly misidentified lines (See ICLAC register)	No commonly misidentified cell lines were used.

Animals and other organisms

Policy information about studies involving animals; ARRIVE guidelines recommended for reporting animal research

Laboratory animals	Female athymic mice aged between 6-8 weeks were purchased from Taconic Farms.
Wild animals	n/a
Field-collected samples	n/a
Ethics oversight	All in vivo experiments were approved and performed in agreement with the McGill University Animal Ethics Committee.

Note that full information on the approval of the study protocol must also be provided in the manuscript.

Flow Cytometry

Plots

Confirm that:

☒ The axis labels state the marker and fluorochrome used (e.g. CD4-FITC).

☒ The axis scales are clearly visible. Include numbers along axes only for bottom left plot of group (a 'group' is an analysis of identical markers).

☒ All plots are contour plots with outliers or pseudocolor plots.

☒ A numerical value for number of cells or percentage (with statistics) is provided.

Methodology

Sample preparation	Single cells were stained with fluorophore-conjugated antibodies in 100µl of PBS with 2% FBS for 30 minutes on ice protected from light. In brief, 1 x 10E6 cells were washed twice then resuspended in PBS with 2% FBS, and viability dye 7-AAD (eBioscience) was added to each sample. For dissociated PDX tumours, murine stromal cells were removed using a Mouse Cell Depletion Kit (Miltenyi) prior to staining.
Instrument	Multi-colour flow cytometry was performed on a FACS CantoII (BD Biosciences).
Software	Flow cytometry data were collected with BD FACS Diva software and analyzed with FlowJo.
Cell population abundance	After gating for live and single cells, the remaining population for analysis was typically 60-70% of total. No cell sorting was performed. See supplementary figure 8.
Gating strategy	Cell were gated on general population (SSC/FSC), single cells (FSC-W/FSC-H), live cells (7-AAD negative), and finally divided into 4 quadrants to evaluate FITC/PE levels. Compensation and gating for positive/negative cells were based on control samples that were unstained and single-stained with each fluorophore used.

☒ Tick this box to confirm that a figure exemplifying the gating strategy is provided in the Supplementary Information.
